# The Magnetic Electron Ion Spectrometer: A Review of On-Orbit Sensor Performance, Data, Operations, and Science

**DOI:** 10.1007/s11214-021-00855-2

**Published:** 2021-10-28

**Authors:** S. G. Claudepierre, J. B. Blake, A. J. Boyd, J. H. Clemmons, J. F. Fennell, C. Gabrielse, M. D. Looper, J. E. Mazur, T. P. O’Brien, G. D. Reeves, J. L. Roeder, H. E. Spence, D. L. Turner

**Affiliations:** 1 Space Science Applications Laboratory, The Aerospace Corporation, El Segundo, CA USA; 2 Department of Atmospheric and Oceanic Sciences, UCLA, Los Angeles, CA USA; 3 Department of Physics and Astronomy, University of New Hampshire, Durham, NH USA; 4 Space and Atmospheric Sciences Group, Los Alamos National Laboratory, Los Alamos, NM USA; 5 Space Exploration Sector, The Johns Hopkins University Applied Physics Laboratory, Laurel, MD USA

**Keywords:** Relativistic electron sensors, Energetic magnetospheric particles, Acceleration, transport, and loss of radiation belt particles, Particle instrument operation

## Abstract

Measurements from NASA’s Van Allen Probes have transformed our understanding of the dynamics of Earth’s geomagnetically-trapped, charged particle radiation. The Van Allen Probes were equipped with the Magnetic Electron Ion Spectrometers (MagEIS) that measured energetic and relativistic electrons, along with energetic ions, in the radiation belts. Accurate and routine measurement of these particles was of fundamental importance towards achieving the scientific goals of the mission. We provide a comprehensive review of the MagEIS suite’s on-orbit performance, operation, and data products, along with a summary of scientific results. The purpose of this review is to serve as a complement to the MagEIS instrument paper, which was largely completed before flight and thus focused on pre-flight design and performance characteristics. As is the case with all space-borne instrumentation, the anticipated sensor performance was found to be different once on orbit. Our intention is to provide sufficient detail on the MagEIS instruments so that future generations of researchers can understand the subtleties of the sensors, profit from these unique measurements, and continue to unlock the mysteries of the near-Earth space radiation environment.

**Supplementary Information:**

The online version contains supplementary material available at 10.1007/s11214-021-00855-2.

## Introduction

We begin this chapter with a brief refresher on the MagEIS instruments and measurement techniques. In Sect. 2, we discuss some of the scientific achievements in which MagEIS data played a central role. We then proceed to describe the MagEIS data products in detail in Sect. 3, followed by a description of revised calibrations that were undertaken once on-orbit, guided by computer simulations of the instrument response (Sect. 4). Data validation and intercalibrations are discussed in Sect. 5. Important data caveats, of which the end user should be aware, are discussed in Sect. 6, followed by a short section on instrument anomalies (Sect. 7). In Sect. 8, we discuss lessons learned from the on-orbit operations and sensor performance, along with a number of aspects of the design that could be improved upon in future iterations of MagEIS-like instruments. We note that scientific results and instrument cross-calibrations at the ECT suite level, and specifically those studies where the analysis required using multiple instruments from the suite, are discussed in the Reeves et al. (this book) chapter.

### Instrument Synopsis

The Van Allen Probes were launched into geostationary transfer orbits (GTO) on 30 August 2012 near the maximum of solar cycle 24, a weak period of solar activity relative to previous cycles. The Van Allen Probes were spinning spacecraft (period $\sim11~\text{s}$) with their spin axes nominally sun-pointing. There were four MagEIS units on each Probe: one low energy unit (“LOW”), two medium energy units (“M35” and “M75”), and one high energy unit (“HIGH”). The LOW, M75, and HIGH units were all mounted with their look directions oriented 75 degrees with respect to the spacecraft spin axis, biased in the anti-sunward direction. The fourth unit, M35, had its look direction oriented 35 degrees with respect to the spin axis, pointing out of the aft deck (anti-sunward). The LOW and M75 units were mounted on the aft deck on one side of the spacecraft, while the M35 and HIGH units were on the opposite side. Each Probe carried two medium energy units to maximize angular sampling in the 200 keV to 1 MeV energy range, though this was not realized on-orbit (see Sect. 8.9). Throughout this chapter, when it is not necessary to distinguish between the two medium energy units, we will generally refer to them as “MED.” The LOW and MED magnetic electron spectrometer units only measured electrons, while the HIGH electron spectrometer unit also housed an ion telescope.

### MagEIS Electron Spectrometers

**Fig. 1:**
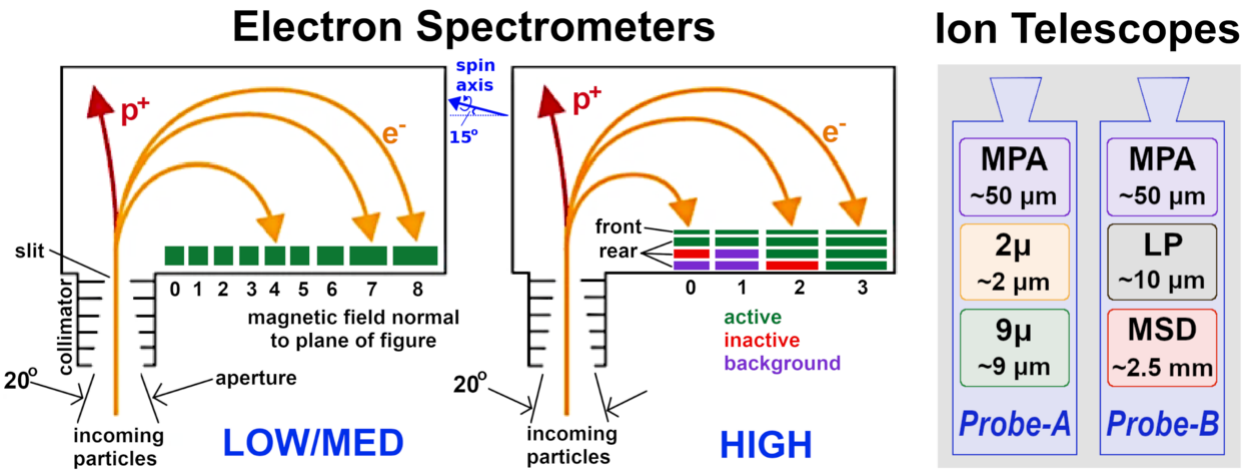
(Left & middle) MagEIS electron spectrometer schematics for the low/medium energy units (left) and the high energy unit (middle). Individual pixel numbers are indicated (starting from 0) and active detectors are shown in green. Some HIGH unit rear detectors were inactive (red), while others were used to monitor background (purple). The orientation of the spacecraft spin axis is shown in blue for the LOW/M75/HIGH units; it points in the opposite direction (to the right) and at a different angle ($55^{\circ }$ below the horizontal line) for the M35 unit. The $20^{ \circ }$ acceptance in the plane of the page is indicated. The $10^{ \circ }$ acceptance (out of the plane) edge sweeps as the spacecraft spins. After Blake et al. (2013). (Right) MagEIS ion telescope detector layout on both Probes. Detector names and thicknesses are indicated

MagEIS used a magnetic filtering technique to measure electrons, where a roughly uniform magnetic field is maintained within the instrument chamber or “yoke.” This measurement technique is illustrated schematically in Fig. 1. With a sensor of this design, charged particles enter through the instrument aperture (field-of-view: $20^{\circ }$ in the plane, $10^{\circ }$ out of the plane) and are collimated before reaching the chamber. Positively charged ions are deflected by the internal field and strike the back or side walls. Conversely, electrons are deflected onto the detectors, or “pixels,” that are mounted in an array on the front wall of the chamber. Momentum selection by the magnetic field ensures that lower energy electrons strike the lower numbered pixels, while higher energy electrons travel farther down the array to the higher numbered pixels. Thus, in an ideal sensor configuration free from penetrating background particles, scattering, and other processes, only electrons over a narrow energy range strike an individual pixel. Note that we define an explicit distinction between pixels and detectors: A detector is a single active silicon element or combination of multiple elements that produces an electronic signal indicating energy deposited by an ionizing particle. A pixel is one detector or multiple detectors working in combination to indicate the position at which a particle traverses a sensor.

In a sensor like MagEIS, design considerations must balance the chamber magnetic field strength with the detector thicknesses that are required to stop electrons within a pixel, all in a reasonably sized sensor package that is able to meet flight requirements. To do so, the pixel arrays in the MagEIS LOW/MED units consisted of 9 individual pixels in a single detector strip. The LOW unit strip was fabricated from a single piece of silicon that measured 0.5 mm in thickness. The MED units had two planes each of which measured 1.5 mm in thickness, with the corresponding pixels in each ganged together to function as a single detector. The HIGH unit arrays consisted of 4 individual pixels, where each pixel stack was composed of two detectors: a thin (0.3 mm) front detector, followed by a thick rear detector that consisted of 3 pairs of detectors each of which was 1.5 mm thick, for a total rear detector thickness of 9 mm. In all MagEIS units, the detector sizes increased with increasing pixel number down the array, as illustrated schematically in Fig. 1. This was done in an attempt to maintain uniform count rates across all pixels in a unit, compensating for the combined effects of a falling spectrum and the decrease of the geometry factor with increasing energy. For a fixed chamber magnetic field strength and a pixel array with uniform thickness, the overall instrument size constraints ensure that an individual magnetic spectrometer can measure electrons that span an energy range of roughly one order of magnitude. This energy range is reduced for the relativistic energies measured in the HIGH unit. The nominal electron energy ranges measured by each MagEIS unit were: $\text{LOW}\sim20\text{--}200~\text{keV}$, $\text{MED}\sim200\text{--}1000~\text{keV}$, and $\text{HIGH}\sim1\text{--}4~\text{MeV}$.

With a sensor of this design, when an electron strikes a pixel, a current pulse is generated in the detector that is measured by the onboard electronic processing and digitized into a pulse height. This pulse height is proportional to the energy deposited by the electron as it passes through the silicon. In an ideal situation, the electron deposits all of its incident energy and completely stops within the detector, in which case the pulse-height-measured energy deposit is equal to the incident energy. However, a number of factors can lead to non-ideal energy collection in the detector, such as backscatter, which we define as any electron that leaves the detector before depositing all of its energy (e.g., through the sides or out the front). Moreover, as discussed in greater detail in Sect. 4.1, electrons can lose some of their incident energy before striking the pixel (e.g., through scattering off of the chamber walls). Nevertheless, to first order, we can assume that the energy deposited in a pixel is roughly equal to the incident electron energy. Thus, the magnetic spectrometer technique provides two independent measures of incident electron energy: momentum selection by the chamber magnetic field and pulse-height analysis. This two-parameter measurement of incident electron energy has tremendous value, in that it allows for quantifiable background estimation and rejection in post-processing. Due to the thicker detectors needed to stop electrons, the HIGH unit was more susceptible to spurious counts from penetrating particles. Thus, to further mitigate background contamination, the HIGH unit used coincidence between the front and rear detectors, where a threshold deposit event had to be registered on both detectors within a specified time window to be considered valid.

### MagEIS Ion Telescopes

Each MagEIS HIGH unit also housed an ion telescope that contained three detectors arranged in a stack. The detector configuration was different in the two telescopes and is illustrated schematically in Fig. 1. Both telescopes carried identical front detectors, which we refer to as the “MPA” detectors (the name used by the detector manufacturer). These annular detectors, nominally 50 μm thick, were used to obtain the primary MagEIS ion measurement, protons over the $\sim60\text{--}1000~\text{keV}$ energy range. Unfortunately, these detectors became noisy early on in the mission and the proton measurements quickly became unreliable and ultimately unusable after $\sim1$ year on orbit (see Sect. 6.7). Pulse height analysis was the only (single parameter) measure of incident particle energy in the ion telescopes and a sweeping magnet was used at the entrance aperture to shield electrons away from the stack. There was no coincidence between any of the detectors in the ion telescope system and all ions detected from the MPA detectors were assumed to be protons. Throughout this chapter, when we refer to MagEIS proton data, we mean the $\sim60\text{--}1000~\text{keV}$ proton data from the front MPA detectors, unless otherwise stated.

The ion telescope on Probe-A carried additional rear detectors that nominally measured energetic proton, helium, and oxygen ions, while the telescope on Probe-B carried an additional rear detector that nominally measured $\sim1\text{--}20~\text{MeV}$ protons. (The middle “LP” detector on Probe-B was inactive.) These rear detector measurements were not part of the primary science requirements for MagEIS and were not extensively calibrated pre-flight. As calibration work is ongoing at the time of writing, these rear-detector telescope measurements are not discussed further in this chapter. We note that the remainder of this chapter focuses largely on the MagEIS electron measurements, since the primary ion measurement from the MPA detectors degraded quickly.

### Detector Bias

**Fig. 2:**
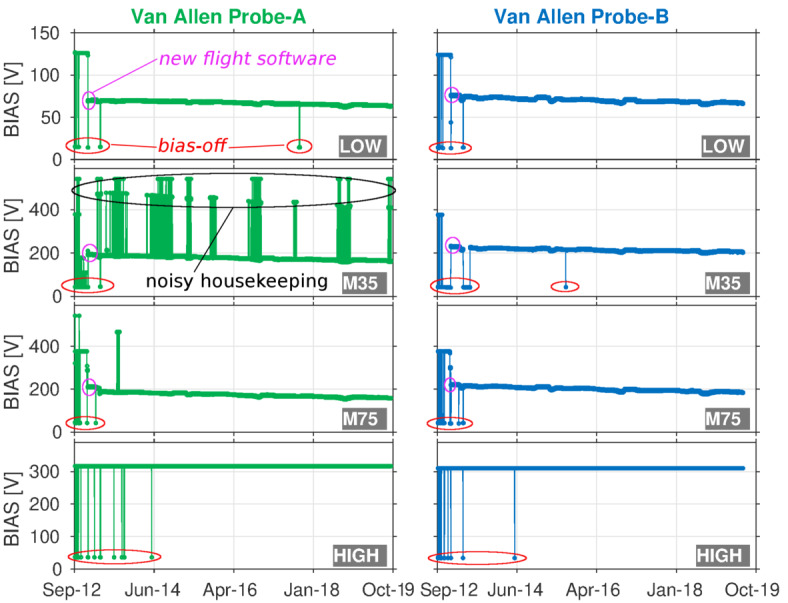
Time histories of the MagEIS bias voltages. The raw instrument housekeeping data have been interpolated to a 5-minute time cadence using a nearest neighbor approach. New flight software uploads are indicated with magenta ellipses and instances where the bias was disabled are shown with red ellipses. The M35-A unit had considerable noise in its housekeeping measurements (see Sect. 6.15)

In the silicon solid-state detectors of an instrument like MagEIS, current pulses are generated when ionizing radiation strikes a detector creating free charge, which migrates through the semiconductor when a sufficient voltage is applied across the detector. The current pulses are collected and read out from an anode on the detector surface. The applied voltage, known as the bias voltage, was set to a fixed value at launch for each MagEIS electron spectrometer and the bias was enabled via ground command to either a “bias-on” or “bias-off” state. In the bias-off state, the bias voltages were set to a low value ($\sim20~\text{V}$ for LOW and $\sim40~\text{V}$ for MED/HIGH). In the bias-on state, the bias voltages were set to $\sim125~\text{V}$, $\sim375~\text{V}$, $\sim310~\text{V}$, for the LOW, MED and HIGH units, respectively, at the beginning of the mission. In an effort to investigate and potentially mitigate the source of noise in LOW/MED pixel 0 and pixel 1 (see Sect. 6.1), new flight software was uploaded to the LOW/MED units in December of 2012 that allowed this fixed bias voltage to be adjusted and lowered (see Sect. 8.3). After a short tuning period, the bias voltages were lowered on the six LOW/MED units, though this did not completely mitigate the noise in pixels 0 and 1, which remained throughout the mission. The LOW units were lowered to $\sim75~\text{V}$ and the MED units were lowered to $\sim210~\text{V}$. Figure 2 shows time histories of the bias voltages for all 8 MagEIS electron spectrometer units over the course of the mission, indicating when the LOW/MED voltages were adjusted. Intervals in the bias-off state are also noted and during these rare instances the MagEIS data should not be used (see Sect. 6.5).

New flight software was not uploaded to the HIGH units and their pre-flight-specified bias voltages were never adjusted on-orbit. We note that when the HIGH units were in the bias-off state, the electron bias was disabled on both the front and rear detectors in each pixel. This bias was also used for the thick MSD detector in the ion telescope on Probe-B. It was not possible to disable the bias on the front MPA detectors in the ion telescopes, nor on the thin rear detectors in Probe-A’s telescope. The MPA bias was simply supplied by the 5 V monitor.

### Thermal Control System

**Fig. 3:**
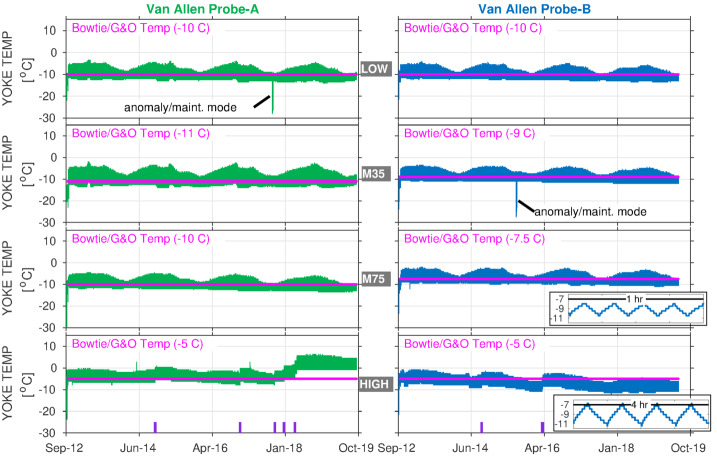
Magnetic spectrometer yoke temperatures for all 8 MagEIS units plotted over the duration of the mission. The fixed temperatures assumed for the gain and offsets used in the bowtie analysis (Sect. 4.2) are indicated in magenta. The spurious (noisy) housekeeping values in M35-A and M75-A (e.g., Fig. 2) have been filtered out in these panels. The times when LOW-A and M35-B were rebooted into maintenance mode are noted, as are the times when the HIGH unit’s heater set point was changed (with vertical purple hashes in the bottom panels). The last two panels for Probe B show insets of the short term variability in yoke temperature due to the thermal control system ($\sim15~\text{min}$ periodicity for LOW/MED; $\sim60~\text{min}$ for HIGH)

The MagEIS electron spectrometers utilized an active thermal control system to maintain the ambient temperature inside the yoke within a specified tolerance of $10~^{\circ }\text{C}$. The yoke, made from a high-cobalt steel alloy (Hiperco-50), completely enclosed the detector array (see Fig. 28 below for an example from a similar sensor). The operational heaters could be enabled or disabled via ground command and were usually enabled while on-orbit. When enabled, the heater was triggered on when the locally-sampled temperature inside the yoke dropped below a prescribed heater set point, which could be adjusted via ground command to allow for fine control over the temperature range for the yoke and associated electronics. This yoke temperature is plotted in Fig. 3 for all 8 magnetic spectrometers over the course of the mission. There are long-term orbital variations visible ($\sim18$ months), along with shorter-term variations (∼minutes-to-hours) due to orbital motion and the action of the thermal control system. The period of these shorter-term variations was $\sim60~\text{min}$ for the HIGH unit and $\sim15~\text{min}$ for the LOW/MED units (the different periods were due to different heater rates and thermal mass). As we learned on orbit, the electron spectrometer measurements were quite sensitive to temperature (see Sect. 6.2), particularly the HIGH-A unit, and several adjustments were made to its heater set point over the course of the mission, as indicated in the figure.

## Scientific Results

We now provide an overview of the key scientific results from the Van Allen Probes mission in which MagEIS data played a central role. Of course, it would not be possible to review all such works here and the presentation that follows is limited to a few select studies. Similarly, there were a number of important results that were obtained only when the MagEIS data were combined with the complementary measurements from the ECT suite at lower (HOPE) and higher (REPT) energies. These key results, where the full energy coverage of the suite was necessary for the analysis, are described in the Reeves et al. (this book) chapter.

### Inner Zone Electrons

**Fig. 4:**
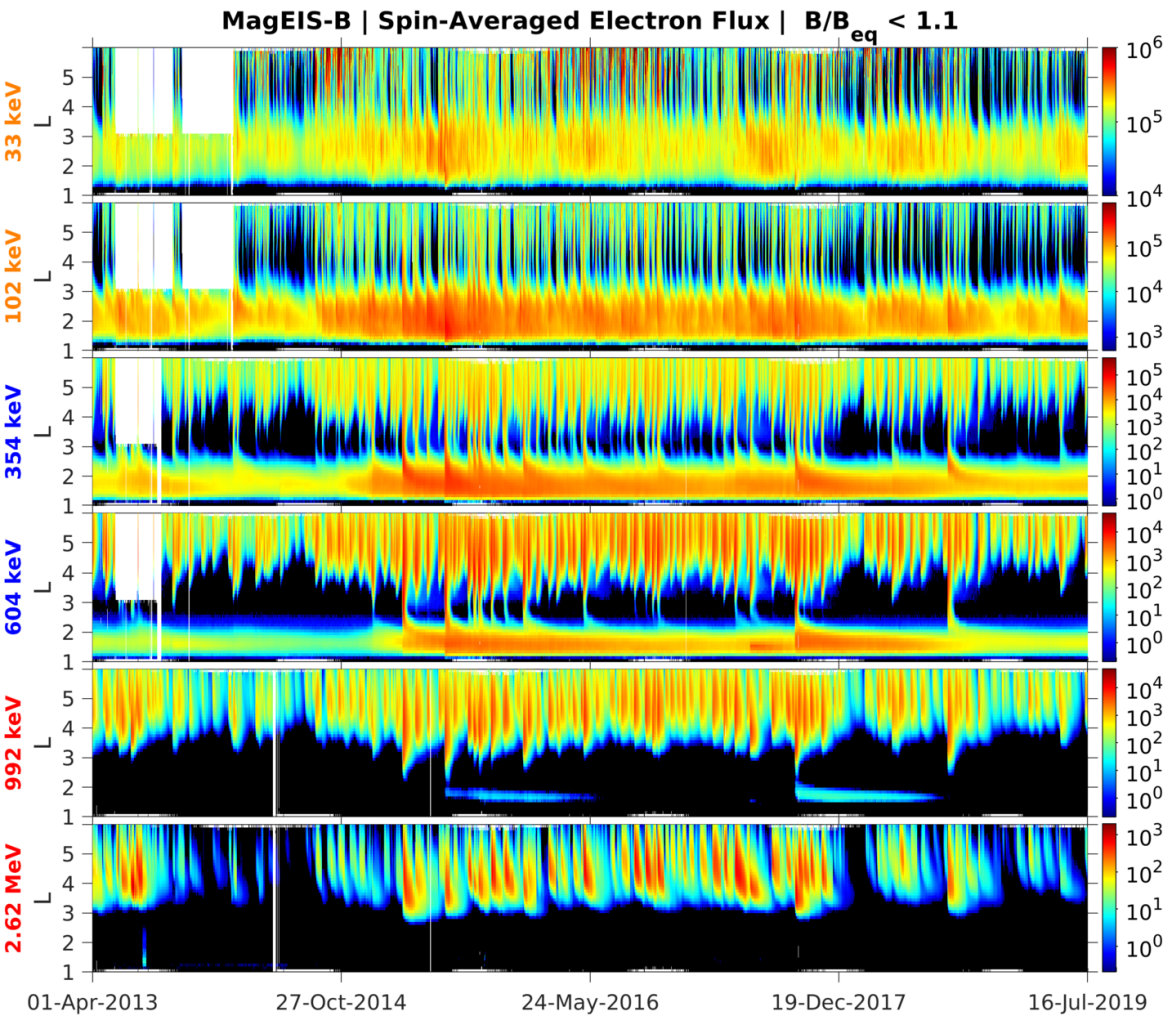
Summary of background-corrected MagEIS electron flux measurements from Probe-B. Two channels are shown from each unit, LOW (orange labels), M75 (blue), and HIGH (red). The spin-averaged fluxes were obtained when the spacecraft was in close proximity to the magnetic equator, as measured by the ratio of the local magnetic field strength ($B$) to the equatorial field ($B_{eq}$). The data are presented as daily averages in $L$ bins of $0.1L$ width. The long data gaps early in the time interval at $L > 3$ are due to the unavailability of histogram data, which are required for the background corrections as detailed in Sect. 3.2.4

Prior to the Van Allen Probes mission, most studies of MeV electrons in the inner zone were subject to considerable uncertainty due to measurement contamination from the very energetic inner proton belt. The quantifiable estimates of detector background levels enabled by the MagEIS electron spectrometer design (see Sect. 3.2.4) revealed many never-before-seen features of the inner radiation belt. Figure 4 shows an overview of these background-corrected electron measurements in six energy channels from Probe-B, organized by the McIlwain $L$ parameter. Unless otherwise stated, the Olson and Pfitzer (1977) quiet magnetic field model is used throughout this chapter for computing the global magnetic field and derived quantities.

One of the surprising early findings from the mission was that the inner zone contained relatively few electrons with kinetic energies in excess of 1 MeV. Fennell et al. (2015) investigated a quiet interval in late February of 2013 and placed an upper limit on 1 MeV electron flux in the inner zone at the low intensity of $\sim0.1~\text{electrons}/(\text{cm}^{2}\,\text{s}\,\text{sr}\,\text{keV})$. This was in stark contrast to prior measurements and state-of-the-art empirical models, which suggested much higher inner zone intensities at energies $>1~\text{MeV}$ (e.g., Ginet et al. 2013). Follow-on work (Claudepierre et al. 2017, 2019) confirmed these initial results and also demonstrated that strong geomagnetic storms could inject new MeV populations into the inner zone, forming long-lasting ($\sim1$ year), transient inner electron belts. Instances of these injections can be seen in Fig. 4 at $\sim1~\text{MeV}$ following the June 2015 and September 2017 storms. Claudepierre et al. (2017, 2019) also used an alternative correction algorithm to reveal that very-low-intensity MeV electron populations were in fact present in the inner zone prior to the two major enhancements noted above. We emphasize that these MeV populations were only revealed when the MagEIS data were analyzed with the alternative correction algorithm and were below the sensitivity threshold of the algorithm used in Fennell et al. (2015). These quantitative measurements of the inner electron belt have motivated a revisiting of historical inner zone electron data, in order to scrutinize the cleanliness of earlier measurements (e.g., Selesnick 2015; Boscher et al. 2018).

**Fig. 5:**
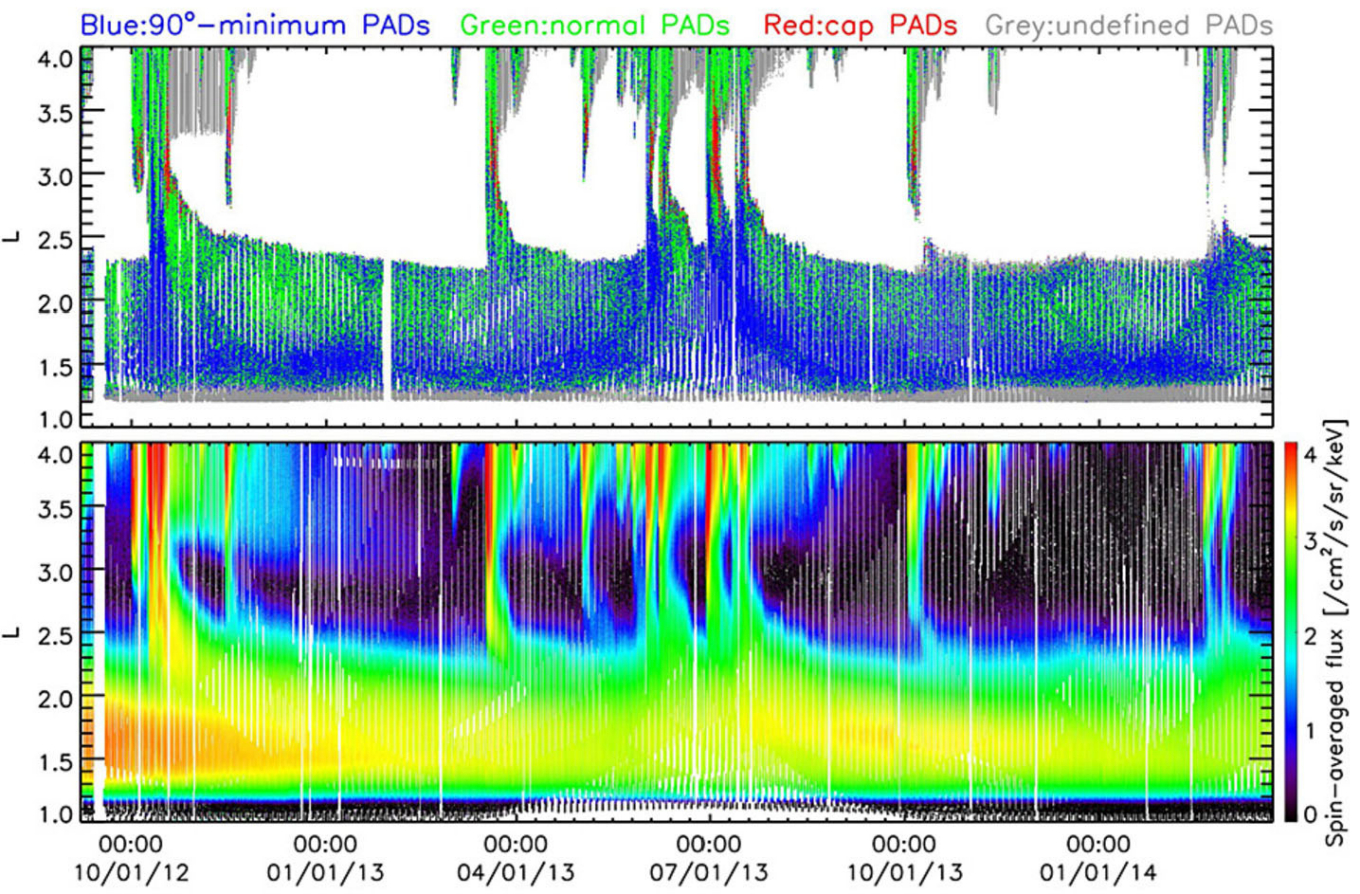
(Top) Pitch angle distribution classification for $\sim450~\text{keV}$ electrons from MagEIS-A. At the outer portion of the inner belt, electrons associated with inward radial transport are largely peaked at $90^{\circ }$ (green), while in the heart of the inner belt the distributions have a local minimum at $90^{\circ }$ (blue). The “cap” or “flat-top” distributions (red) are associated with rapid scattering by waves in the slot region. (Bottom) Spin-averaged flux of $\sim 450$ electrons from MagEIS-A. From Zhao et al. (2014) ©The American Geophysical Union

These surprising features of the inner electron belt were not solely found in the relativistic population. At lower energies, sporadic injections were frequently observed, at times penetrating through the slot region into the inner zone. Turner et al. (2017a) investigated the energy dependence of these low $L$ injections as a potential source for the inner belt. The authors argued that these advective injections from higher $L$ are the dominant source for inner belt electrons at energies $<200~\text{keV}$, rather than a source from inward radial diffusion (e.g., Lyons and Thorne 1973; Schulz and Lanzerotti 1974). The authors also statistically analyzed $\sim3.5$ years of data and found that injections into the inner zone occurred frequently, on the order of 2–3 per month at 200 keV, and more frequently at energies $<200~\text{keV}$. Related work by Zhao et al. (2014) examined the angular distributions of these inner zone electrons. Their analysis revealed that the bulk of the inner zone population at 100s keV energy exhibited angular distributions with a local minimum at $90^{\circ }$ (see Fig. 5). This rather unexpected result has been interpreted as the combined effect of energy and pitch angle diffusion (Albert et al. 2016), though there is no general consensus regarding the formation and sustainment of such distributions.

The clean inner zone electron measurements from MagEIS have also contributed to investigations of the electric field at low-$L$. For example, Selesnick et al. (2016) demonstrated magnetic local time (MLT) asymmetries in $\sim100\text{s}~\text{keV}$ electron flux at low $L$, which suggested electron drift trajectories that were not consistent with standard empirical electric field models (see also Su et al. 2016). The authors argued that a uniform convection electric field ($\sim0.5~\text{mV/m}$), in addition to the standard corotation and convection potentials, was required to explain the observations. O’Brien et al. (2016) used MagEIS data to estimate the radial diffusion coefficient at $L<3$ and demonstrated that the values were consistent with diffusion due to impulsive electrostatic fluctuations. The authors of these studies were only able to quantify the electric-field effects on the particles because the inner zone electron measurements could be used confidently. On the whole, MagEIS measurements have completely transformed our understanding of the inner radiation zone.

### Direct Observations of Wave-Particle Interactions

**Fig. 6:**
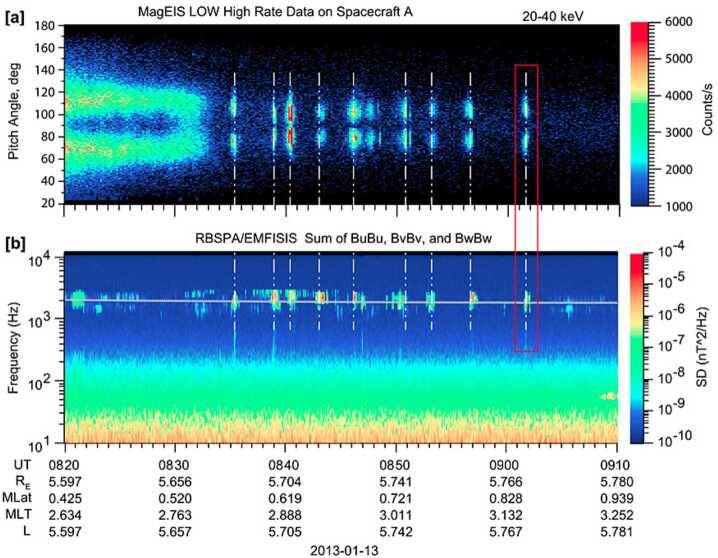
(**a**) Local pitch angle distribution from LOW-A in high-rate mode showing quasi-periodic electron flux bursts at 20–40 keV. (**b**) Bursty chorus wave emissions from the EMFISIS instrument (Kletzing et al. 2013) showing a nearly one-to-one correspondence with the flux bursts. The grey horizontal line indicates half the electron cyclotron frequency. From Fennell et al. (2014) ©The American Geophysical Union

The measurement capabilities of the MagEIS instrument have provided direct observations of radiation belt wave-particle interactions with unrivaled detail. Figure 6 shows quasi-periodic electron flux bursts in close association with simultaneous bursts of chorus wave emissions. The electron measurements were obtained from the LOW-A unit when it was in a special, “high-rate” mode (Sect. 3.2.5). Using the observed parameters, the cyclotron resonant energy was calculated as $\sim20\text{--}40~\text{keV}$, consistent with the range of electron energies over which the bursts were observed. Importantly, the very fine angular resolution in high-rate mode permitted a tight estimate of the resonant energy range; a similar calculation using angular distributions obtained from the normal mode data would only provide a much coarser energy bound of $\sim10\text{--}100~\text{keV}$. MagEIS high-rate data were also used by Shumko et al. (2018) to reveal the first observation of the electron microburst process near the high-altitude generation region (i.e., outside of low-Earth orbit).

Direct observations of electron interactions with lower frequency magnetospheric waves have been demonstrated as well. In a case study, Maldonado et al. (2016) used MagEIS high-rate mode data to demonstrate a one-to-one correspondence between equatorial magnetosonic noise (ion Bernstein-mode waves) and modulations in $\sim100~\text{keV}$ electron flux. Their test-particle simulations suggested that electron bounce resonance with the waves was responsible for the rapid flux modulations and the formation of butterfly angular distributions (see also Li et al. 2016). We also note the very recent work of Zhu et al. (2020), who showed direct modulation of MeV electron flux near the loss cone in an event study using Van Allen Probes REPT data. While MagEIS observations were not the primary data set used in this study, they provided important constraints on the lower energy bound of the observed flux modulations.

**Fig. 7:**
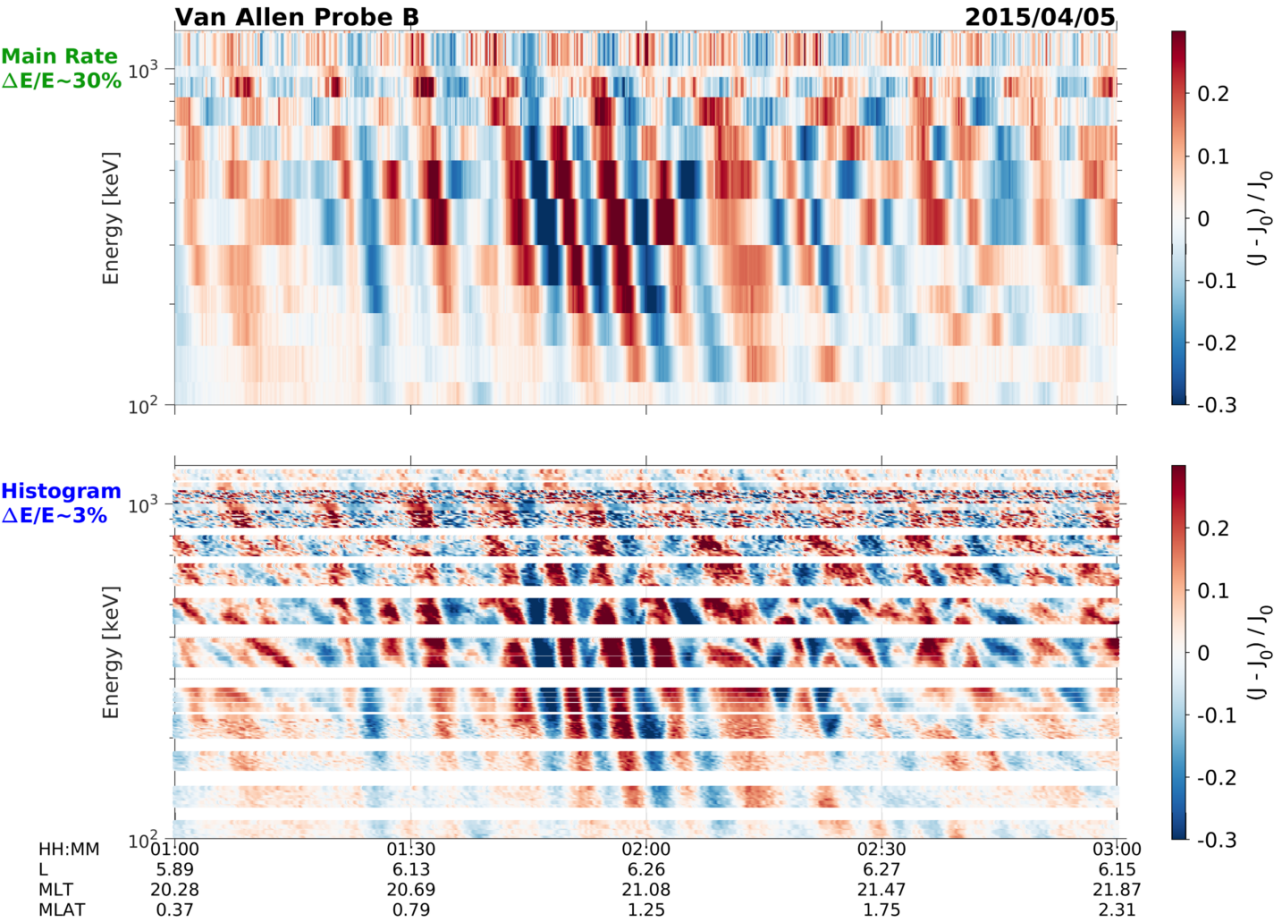
MagEIS residual flux (perturbations from the mean) oscillations plotted versus time (horizontal) and energy (vertical). (Top) ULF drift-resonance signature in the main channel data. (Bottom) The same signature in the high-energy resolution MagEIS histogram data, where each main channel is further subdivided into $\sim5\text{--}10$ energy channels. Adapted from Hartinger et al. (2018) ©The American Geophysical Union, but shown here with the fully-calibrated histogram data (see Sect. 4.2)

The fine energy resolution of the MagEIS instrument (i.e., $\Delta E/E \lesssim 30\%$) has enabled the direct observation of a large number of drift-resonance events between magnetospheric particles and ultralow frequency (ULF) waves. Early in the mission, Claudepierre et al. (2013) and Dai et al. (2013) demonstrated drift resonance with $\sim100~\text{keV}$ electrons and protons, respectively, using MagEIS data. Since then, a large number of studies have uncovered similar electron drift-resonance signatures in the MagEIS data (e.g., Hao et al. 2014; Foster et al. 2015; Hao et al. 2017; Chen et al. 2017; Tang et al. 2018; Korotova et al. 2018; Hao et al. 2019; Teramoto et al. 2019). There have also been a large number of reports of drift-bounce resonance in Van Allen Probes data, with several case studies using MagEIS proton measurements (Korotova et al. 2015; Takahashi et al. 2018; Wang et al. 2018). We highlight recent work (Hartinger et al. 2018) that used the very fine energy resolution (i.e., $\Delta E/E \lesssim 3\%$) MagEIS histogram data (Sect. 3.2.4) to identify signatures of ULF/electron drift resonance that were not apparent in the main-rate data (see Fig. 7). The highly detailed ULF drift-resonance signatures revealed by MagEIS in the aforementioned studies have spurred a renewed theoretical interest in this area, which has been extended to incorporate nonlinear (Li et al. 2018) and wave growth/damping effects (Zhou et al. 2015, 2016). Perhaps one of the most interesting findings regarding electron drift resonance in the inner magnetosphere is that it is very often observed to be spatially localized (Claudepierre et al. 2013; Teramoto et al. 2019). Such a finding would not have been possible without high-resolution measurements from multiple spacecraft.

### Particle Acceleration and Transport

One of the most important findings of the Van Allen Probes mission is the crucial role that “seed” electrons ($\sim100\text{s}~\text{keV}$) play in multi-MeV enhancement events. Jaynes et al. (2015) analyzed an event from September of 2014 where the conditions that are usually associated with multi-MeV enhancements were observed (e.g., chorus/ULF wave activity and high solar wind speed), but in which the MeV fluxes stayed low. The authors used MagEIS data to demonstrate that 100s of keV electrons were not present at sufficient intensities to be further accelerated to MeV energies. Thus, an important link in the electron acceleration chain was broken for this event. A number of other studies have used MagEIS measurements to demonstrate this fundamental relationship between seed and MeV electrons (e.g., the statistical work of Boyd et al. 2014 and the modeling work of Foster et al. 2015 and Li et al. 2014).

Seed electrons are typically assumed to originate from higher $L$ and in association with substorm injections, dipolarizations, and other nightside phenomena. Turner et al. (2017b) used MagEIS data along with numerous other in-situ measurements to examine a complex series of injections observed during a relatively quiet interval (maximum $\text{AE}<300~\text{nT}$). The authors used a drift-mapping technique, the accuracy of which is directly tied to the MagEIS energy resolution, to determine the injection boundary location in $L$, MLT, and time. They demonstrated that at least 5 of the observed injections were localized (narrow) in MLT, while one large scale injection was also observed across the entire nightside. These results concerning the scale size of the injection region are important towards quantifying the role that injections of seed electrons contribute to the overall formation of the MeV radiation belt.

MagEIS measurements have also provided important information on the global, energy-dependent radiation belt response to different interplanetary drivers. For example, Shen et al. (2017) examined the influence of coronal mass ejections (CMEs) and stream interaction regions (SIRs) on the outer radiation belt and found that CME driving was more effective at enhancing the belts at lower $L$ ($L\sim 3$), while SIR effects were mainly confined to higher $L$ ($L\gtrsim 5$). Similar statistical work was presented by Turner et al. (2019a), who demonstrated a remarkably repeatable feature where $\sim90\%$ of storms analyzed showed seed electron enhancements at lower $L$ ($L\approx3\text{--}4$). This work also delineated important distinctions between different CME types (e.g., sheaths and/or ejecta), with storms driven by only sheaths or only ejecta most likely to result in radiation belt dropouts, while those driven by full CMEs ($\text{sheath}+\text{ejecta}$) and SIRs were most likely to result in belt enhancements (see also Kilpua et al. 2019). It has also become clear that associating radiation belt enhancements with geomagnetic storms/activity is not the best way to organize statistical surveys (e.g., Zhao et al. 2019a), since the belts can be significantly enhanced during non-storm intervals (Schiller et al. 2014). A number of studies using MagEIS data have also demonstrated the prevalence of drifting flux “dropouts” or “negative” drift echoes associated with interplanetary shock impacts (e.g., Hao et al. 2016; Liu et al. 2019). These appear to be related to radial gradients in phase space density and their dependence on energy (e.g., Boyd et al. 2014).

### Particle Loss

Of course, radiation belt enhancements cannot be studied without a careful consideration of the loss processes that influence the rate and extent of electron enhancement. The Van Allen Probes have shed considerable light on this topic and there is now strong evidence that the large scale, rapid flux dropouts observed at higher $L$ during storm main phase are largely due to loss to the magnetopause (Li and Hudson 2019, and references therein). However, electron loss due to resonant wave-particle scattering also plays an important role in global losses from the radiation belts, particularly at lower $L$. For example, Turner et al. (2014) used MagEIS data to study a storm-time dropout and acceleration event and demonstrated that the dropout above $L=4$ was due to magnetopause loss, which was confirmed in modeling work (Hudson et al. 2014). While wave-driven electron acceleration was observed in the event, the authors demonstrated that it was not sufficient to overcome the losses, so that the overall belt response was depletion. The authors also demonstrated that some additional process, other than loss to the magnetopause, was required to explain the depletion observed at lower $L$. Electromagnetic ion cyclotron (EMIC) waves were suggested, which, due to their large amplitudes, can produce rapid scattering of electrons. Statistical work using MagEIS data (Xiang et al. 2018) demonstrated that EMIC wave scattering is the dominant dropout mechanism at low $L$ ($<4.5$), while magnetopause loss dominates at higher $L$ ($>4.5$).

EMIC waves alone cannot produce the rapid, global depletion observed during storm main phase, since they do not resonate with electrons near $90^{\circ}$ pitch angle. Thus, other wave modes, such as chorus and plasmaspheric hiss, must be involved in the resonant-scattering losses. In an event study, Miyoshi et al. (2015) used MagEIS data, ground radar measurements of precipitation, and simulations to demonstrate strong-diffusion scattering by chorus waves. The detailed MagEIS energy spectrum observed near the loss cone was crucial towards conclusively identifying the scattering mechanism and linking the ground measurements to space. Inside of the plasmasphere, enhanced hiss waves also contribute to the electron losses. One important finding from the Van Allen Probes was the discovery of so-called “low-frequency” hiss. Li et al. (2013) demonstrated that these waves are amplified in the outer plasmasphere due to injected energetic ($\sim100~\text{keV}$) electrons. The authors used MagEIS observations to show that the upper energy of the injected electrons was in close agreement with the minimum cyclotron resonant energy calculated from hiss, again making use of the high resolution afforded by MagEIS. Ni et al. (2014) used MagEIS data and simulations to show that this newly-revealed hiss population can have a significant impact on electron loss timescales.

In addition to the studies of electron loss during storm main phase, MagEIS data has also played a central role in a number of investigations into the quiet-time structure of the radiation belts. Reeves et al. (2016) examined the energy dependence of the injection, acceleration, and loss of electrons in the MagEIS energy range. They showed the development of a “wave-like” or “S-shaped” spectrum several days after storm main phase, which was attributed to hiss-wave scattering and confirmed using simulations (Ripoll et al. 2016). Related work (Zhao et al. 2019b) used background-corrected MagEIS data to show that deep local minima were observed in the energy spectrum between 100 keV and 1 MeV, termed “bump-on-tail” or “reversed” energy spectra. These features are prevalent in the MagEIS data following storms and develop due to the energy dependence of the electron decay timescales from hiss waves. Claudepierre et al. (2020a,b) used MagEIS measurements to conduct a large-scale statistical analysis of these decay timescales, building on the earlier work of O’Brien et al. (2014) and linking the morphological features noted above to the action of pitch-angle diffusion from a variety of mechanisms (Coulomb scattering, hiss waves, VLF transmitter waves, and EMIC waves).

### Applications of MagEIS Data

High-accuracy, low-background observations from sensors like MagEIS, in a well-chosen orbit like that of the Van Allen Probes, are designed to provide a variety of opportunities to do highly impactful science. However, they also provide many opportunities to address applied problems in space science. These applications come in three basic categories: space environmental assessment, space climatology modeling, and space weather modeling.

The electrons that MagEIS measured can cause internal charging and gradual degradation of electronic components on spacecraft. MagEIS’s proton and electron measurements also covered the important energy ranges for solar cell degradation. As one would expect, MagEIS data were invaluable for technical assessments of recurring satellite anomalies and degradations in medium Earth orbiting (MEO) satellites, although details of those investigations cannot be shared here. In addition, MagEIS electron data were used to understand scientific sensor responses. For example, MagEIS data were used to investigate the RAPID/IES instrument response on Cluster (Kronberg et al. 2016), and to validate electron fluxes obtained from sensors on GPS satellites (Morley et al. 2016). A new technique was developed with MagEIS data to use drift echoes to assist in sensor cross-calibration between MagEIS and other sensors on the Van Allen Probes (O’Brien et al. 2015a). MagEIS data were also used as part of the assessment of climatology models of the radiation belts (de Soria-Santacruz Pich et al. 2017).

**Fig. 8:**
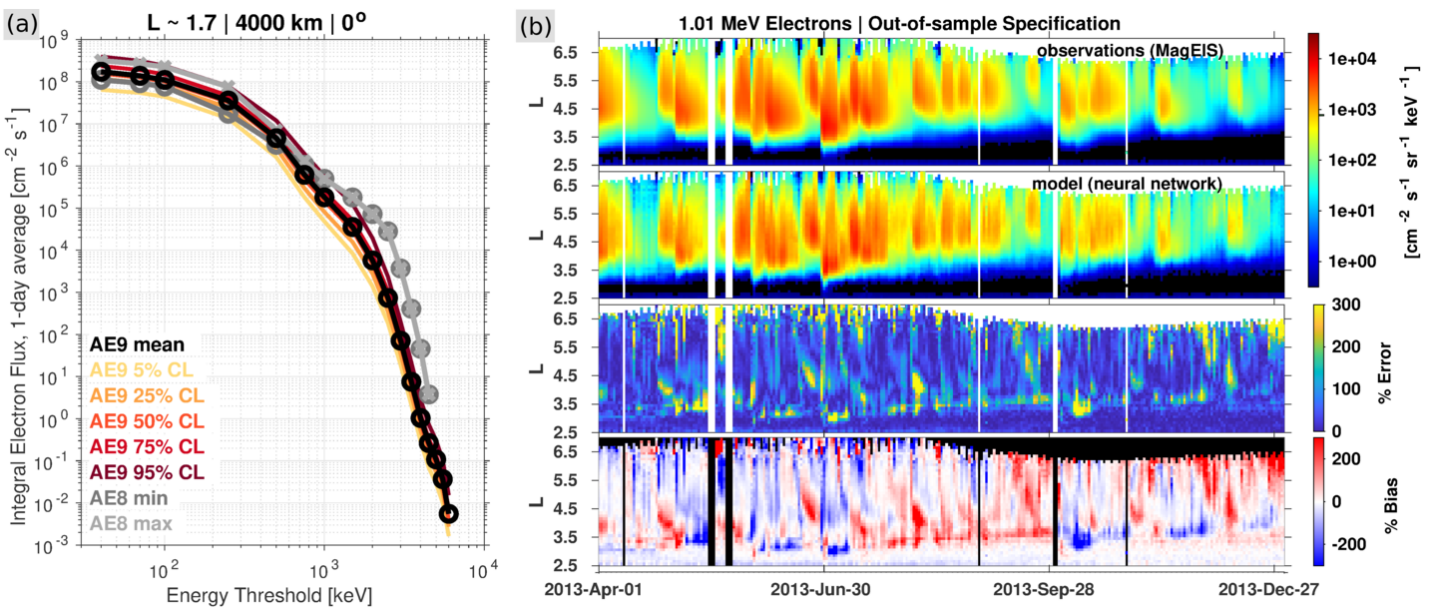
(**a**) Illustration of the effect that MagEIS data inclusion has on the AE9 climatology model, relative to the legacy AE8 model, for the orbital parameters indicated. The AE8 model is shown in both solar minimum and maximum states. The AE9 model is shown in its mean state, along with confidence levels (CL) from 100 perturbed mean scenarios. (**b**) Summary of the SHELLS neural network model performance for an out-of-sample specification of 1 MeV electron flux. The four panels show MagEIS observations, the SHELLS model (nowcast), and the percent error and bias between the model and the observations. After Claudepierre and O’Brien (2020) ©The American Geophysical Union

MagEIS was designed to make especially clean measurements of inner zone electrons in the presence of penetrating proton background. Therefore, MagEIS data are ideal for use in the development of space climatology models needed for satellite design (O’Brien et al. 2018; Papadimitriou et al. 2018). The most prominent of these climatology models is the International Radiation Environment Near Earth (AE9/AP9-IRENE; Ginet et al. 2013; Johnston et al. 2015), which incorporated MagEIS data into version 1.5 of the model. Figure 8a shows the legacy model, AE8 (Vette 1991), in its solar minimum and solar maximum states, as well as the AE9 part of the IRENE model, evaluated along a $0^{\circ }$-inclination (equatorial), 4000 km altitude orbit ($L \sim 1.7$). The introduction of MagEIS data into v1.5 of the model has brought the inner zone electron fluxes down relative to AE8 at energies $>700~\text{keV}$, where MagEIS showed the inner zone rarely contains any appreciable flux (Fennell et al. 2015; Claudepierre et al. 2017). While AE9 also includes Monte Carlo scenarios to capture the statistics of space weather variations around the mean environment, for day-to-day operational use a different set of tools and models are needed, and MagEIS contributed to those as well.

The Van Allen Probes were designed with a real-time space weather broadcast, which transmitted a subset of data to the ground that enabled monitoring of the radiation belts between ground contacts (Kessel et al. 2013). This made possible the incorporation of MagEIS data into real-time data assimilation models, such as DREAM (Walker and Morley 2018) and VERB (Shprits et al. 2019). These models could then be run further into the future to provide a global forecast. The VERB model continues to run today using data from ongoing missions at https://isdc.gfz-potsdam.de/data-assimilative-radiation-belt-forecast/. These data assimilative models can also be run for long-term retrospective studies, which can aid in specific anomaly investigations or in development of climatology models (e.g., Bourdarie et al. 2009). For example, the VERB model has been used for long-term runs with MagEIS data for the radiation belts (Cervantes et al. 2020) and ring current (Aseev et al. 2019).

Exploiting both the space weather broadcast and the archived Van Allen Probe science data, the Applied Physics Lab and NOAA’s Space Weather Prediction Center developed an experimental data product to display a synoptic view of MagEIS electron flux $L$ profiles (Singer et al. 2018). These observations, and additional views, complemented the traditional views available from NOAA’s GOES satellite in geosynchronous orbit. In the future, such products would be especially useful for vehicles operating in MEO or those performing solar-electric orbit raising through the heart of the outer belt.

Recognizing that the Van Allen Probe mission would eventually come to an end, two different groups attempted to develop models that could specify the high-altitude radiation belt state given data from longer-term and ongoing low altitude and geostationary observations. Chen et al. (2019) developed the PreMevE model that used a set of analytical expressions to nowcast and forecast the high-altitude fluxes that would be observed by MagEIS. Claudepierre and O’Brien (2020) employed a neural network model called SHELLS (Specifying High-Altitude Electrons Using Low-Altitude LEO Systems) to achieve similar ends (see Fig. 8b). Because they do not use Van Allen Probes data as input, both models can be used now, and in the future, and can also be used for climatological studies in years prior to the launch of the Probes, so long as the low altitude and geostationary input data are available. Overall, it is evident that models like SHELLS and PreMevE, based largely on low-altitude observations, can capture the large-scale day-to-day variation in high altitude outer zone fluxes, facilitating anomaly assessments and situational awareness for satellite operations.

## Instrument Modes, Data Products, and Data Processing

We now describe the MagEIS data in detail, which is intended to serve as a guide for end users on how to properly use and interpret the data.

### Instrument Modes

Each MagEIS instrument was operated in two independent modes, a “maintenance” mode and a “science” mode. Maintenance mode was used sparingly on orbit, typically only for diagnostic proposes to investigate instrument anomalies and issues, and for flight software and lookup table (LUT) uploads. In maintenance mode, the detector biases and operational heaters were disabled and only housekeeping and instrument status data were recorded and telemetered.

**Fig. 9:**
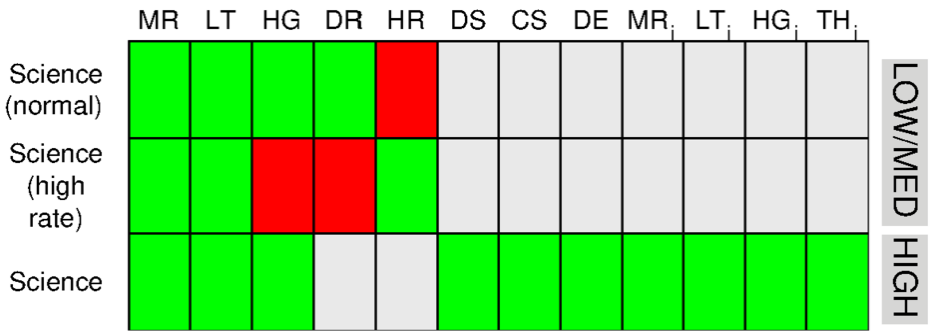
Summary of the data products available in science mode. Green color indicates that the data product is available for the given mode and unit (LOW/MED or HIGH), red indicates that it is not, and grey indicates that it does not exist for this unit/mode. We note that the LOW unit on Probe-A was generally operated in high rate mode above $L=4$ on each orbit. Key: $\text{MR} = \text{main rates}$; $\text{LT} = \text{livetime}$; $\text{HG} = \text{histogram}$; $\text{DR} = \text{derived rates}$; $\text{HR} = \text{high rate}$; $\text{DS} = \text{detector singles}$; $\text{CS} = \text{coincidence singles}$; $\text{DE} = \text{direct events}$; $\text{MR}_{i} = \text{ion main rates}$; $\text{LT}_{i} = \text{ion livetime}$; $\text{HG}_{i} = \text{ion histogram}$; and $\text{TH}_{i} = \text{ion threshold channels}$

In MagEIS science mode, there were two independent modes, “normal” science mode and “high-rate” science mode. Normal science mode, which we simply refer to as science mode, telemetered down the primary set of MagEIS science data products: main rates, detector livetimes, and histograms, along with all of the standard status and housekeeping data. The LOW and MED units could be commanded into a special high-rate mode, where a high-time/high-angular resolution science data product was recorded, in addition to the standard main rate and livetime data products. In high-rate mode, also referred to as “sample” mode or “burst-rate” mode, the histogram data and derived rate data were not recorded in favor of the high-rate data. This is important because in high-rate mode when histogram data were not recorded, background corrections could not be performed. The HIGH unit only operated in the “normal” science mode and did not have a high-rate or derived-rate data product. We now describe these data products in greater detail, with their availability in the various science modes illustrated in Fig. 9.

### Data Products

#### Electron Main Rate Data

**Fig. 10:**
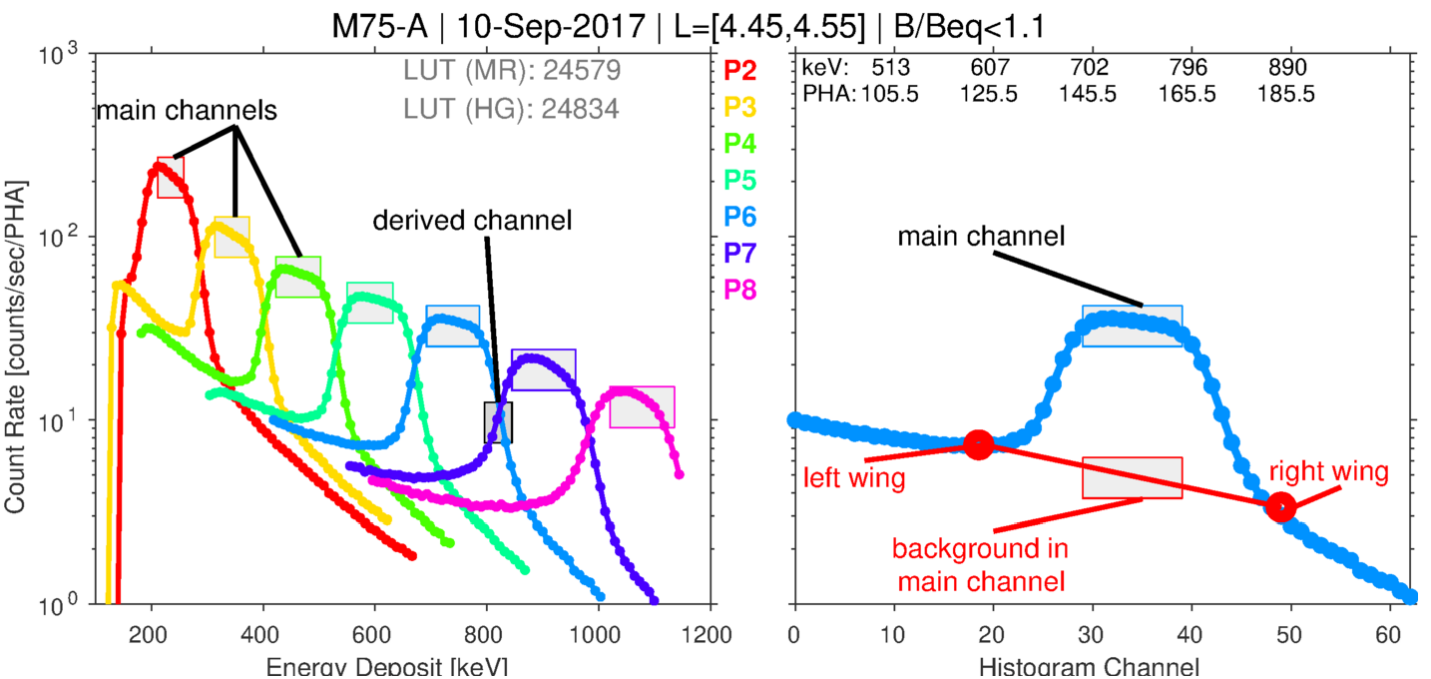
Left: Spin-averaged histogram data from pixels 2–8 on the M75-A unit averaged in the indicated $L$ and $B/B_{eq}$ range on 10 Sep 2017. The main rate channel boundaries, defined via the main rate LUT for each pixel, are indicated with a shaded box (the height of each box is arbitrarily chosen). One of the 6 derived channels, between P6 and P7, is also indicated. Right: One of the pixels from the left panel (P6), now plotted versus histogram channel number, 0–63, with the corresponding PHA channels and energy deposit values indicated along the top horizontal scale. Various parameters used in the background corrections are also labeled (red)

On-orbit, each spacecraft spin was subdivided into a fixed number of angular sectors by the MagEIS flight software. The primary measurement recorded on an individual detector in an electron spectrometer unit was the pulse height spectrum of particle counts in each angular sector. Figure 10 (left) shows examples of these spectra from 7 pixels on the M75-A unit, taken from flight data. Each pixel’s pre-flight-determined gain and offset have been used to convert the pulse-height analyzer (PHA) channel into an equivalent energy deposit, which is plotted along the horizontal scale. In fact, the pulse-height spectra were not telemetered to the ground at the full 256 PHA channel resolution due to telemetry constraints. The data shown in the figure have been downsampled in energy, as described in Sect. 3.2.4. Before this downsampling, on-board LUTs that defined the main channel passband were used to sum the counts centered near the peak (flat-top) response of each pixel, as illustrated in Fig. 10. These counts, which we refer to as the “main channel rates” or the “main rates,” were reported in the telemetry as the primary science data product.

For nearly all of the flight data, one main channel was defined for each pixel on the LOW/MED units, giving 9 total main rate channels from the 9 pixels, P0–P8. As discussed below in Sect. 6.1, the first two of these pixels (P0 and P1) were typically noisy and excluded from the primary (i.e., LOW, M75, and HIGH merged together) data products. Thus, there were typically 7 valid (noise-free) main rate channels for a given LOW/MED unit. For the wide pixels on the HIGH unit, one main channel was defined for the first pixel (P0), while on the subsequent three pixels (P1–P3), each peak response region was subdivided into two main channels, giving seven total main rate channels. There was an interval (∼ Oct 2012 through Feb 2013), after we realized that LOW/MED P0 and P1 were noisy, where such a channel-subdivision was implemented on P2 and P3 on the MED units (creating 4 main channels from these two pixels). However, this approach was abandoned, as the two narrow channels from a single MED pixel were virtually indistinguishable from one another, and we reverted to defining one main rate (MR) channel from each pixel on the MED units (retaining the two noisy main rate channels obtained from pixels 0 and 1).

#### Electron Derived Rate Data

On the LOW/MED units, in addition to the main rate channels, interstitial or “derived rate” channels were defined via the main rate LUT, generally for the 6 overlapping pixel pairs from P2 to P8. An example of such a channel is indicated in Fig. 10. These channels were introduced in order to increase the energy resolution by a factor of $\sim2$ when the derived channels were incorporated into the main channel spectrum. However, the calibration and validation of the derived rate channels have not been extensively analyzed or scrutinized at the time of writing. This was largely due to the fact that the calibration of the histogram data took higher priority.

#### Livetime Data

All MagEIS units reported a livetime data product for every angular sector of every spin from all detectors. The raw livetime per any given sector provided a count of time that the sector’s data was being taken (i.e., the complement of deadtime). Each raw livetime count represented 32 μsec, which was summed over the sector duration and converted to a percentage for each sector before further downstream processing. The percent livetime thus represented the livetime counts per sector interval compared to the maximum possible livetime for that interval. The livetime clock in the instrument was 32 MHz, so the maximum livetime count was $32 \times 10^{6}$ multiplied by the sector duration, in seconds.

The MagEIS livetime percent values were generally quite high on-orbit (i.e., low deadtime), typically in the $\sim95\text{--}100\%$ range on all of the units. As the highest count rates were usually observed at the lowest energies, the LOW unit saw the most significant deadtime effects, where percent livetime values could decrease to $\sim75\%$ during short intervals of very high fluxes (e.g., during substorm injections). Thus, the livetime corrections described below as part of the standard data processing were typically minimal (i.e., an appreciable livetime of 50% only amounts to a factor of 2 correction on the rate). On the HIGH unit, the rear detector livetimes were used for these corrections. The data compression algorithm used on the raw instrument telemetry introduced a $\sim3\%$ error into the livetimes (Blake et al. 2013), such that the value could exceed 100% by a few percent at a given time. This was handled by defining an effective maximum livetime percentage for each unit, based on trending of on-orbit data, and using this as the reference value for scaling all reported lifetimes. These maximum values were $\sim103\text{--}105\%$.

#### Electron Histogram Data

Telemetry constraints prohibited the full, 256-channel PHA spectrum recorded on each electron spectrometer pixel from being sent to the ground. Recognizing the large potential value in these data, the MagEIS team devised a downsampling strategy where a subset of the full PHA spectrum was retained. These “histogram” channels for each unit were built up onboard the spacecraft from varying combinations of the 256 raw PHA channels for each pixel, using the “histogram” LUTs. The histogram LUT mapped a subset of the full 256 PHA channels into 64 histogram channels. Typically, two adjacent PHA channels were combined into one histogram channel across the main channel passband, with the same, or a coarser, resolution used outside of the main response region (e.g., a 4-to-1 downsampling).

Figure 10 (right) shows histogram data from a single pixel on M75-A, plotted versus histogram channel number with the corresponding PHA channels and energy deposit values listed along the top horizontal scale. Note that within this main channel passband, there are roughly 10 histogram channels (channels 30–40) and $\sim20$ PHA channels (channels 145–165), i.e., the 2-to-1 mapping in the passband noted above. These electron histogram data demonstrate the two-parameter MagEIS measurement technique: the action of the chamber magnetic field leads to the narrow peak centered on $\sim750~\text{keV}$, which provides one estimate of incident energy, while the PHA conversion to energy provides a second estimate. One key benefit of this approach is that the histogram counts outside of the main passband region can be considered background counts, which provides a means for quantifying background levels in each pixel throughout the orbit. We note that HIGH unit electron histograms were coincidence events measured on the rear detectors.

As described in greater detail in Claudepierre et al. (2015), fitting a straight line from the “left wing” region to the “right wing” region provides an estimate of the background level within the main channel passband (see also Claudepierre et al. 2017, 2019). This can then be subtracted from the main channel count rate to provide a more accurate, background-corrected estimate of the true foreground rate. An important consideration here is the delicate trade off between preserving foreground signal while simultaneously removing noise. Figure 10 suggests that the straight line fit might also remove part of the foreground signal, i.e., the right and left wing points are specified where the peak is already rising. We have attempted to mitigate this as much as possible (e.g., by accounting for backscatter in the left wing), but we ultimately adopted a conservative approach that may result in the loss of signal when backgrounds are large and foregrounds are low. Note that, in the example shown in Fig. 10, the background rate within the main channel is roughly $15\%$ of the main rate, a low but not-insignificant level of contamination. In the data calibration and validation discussions that follow, it is important to note that background corrections using this algorithm are not possible when the LOW/MED units are in high-rate mode because histogram data are not recorded. In addition, they are not possible on P8 on the LOW/MED units (because the right wing is undefined), nor on P1 on the LOW/MED units (because the left wing is undefined). Plots analogous to Fig. 10 are provided in the Electronic Supplementary Material for all 8 electron spectrometer units.

It should also be clear that histogram data can provide an additional data product with very fine energy resolution. For example, the MagEIS main channels have a nominal resolution of $\Delta E/E \leq 30\%$. With 10 histogram channels within a main channel passband, this amounts to roughly an order of magnitude increase in energy resolution (i.e., $\Delta E/E \lesssim 3\%$). The histogram channels were not calibrated pre-flight and thus the Geant4/bowtie calibration techniques described in Sect. 4.1 and Sect. 4.2 were used to convert these data into physical units. These flux conversion factors and differential energy channel assignments (i.e., in terms of incident energy) are provided in the Electronic Supplementary Material for each histogram LUT, along with a brief description of the histogram flux conversion procedure, data product, and known caveats.

#### Electron High-Rate Data

MagEIS high-rate mode data is a high-angular/high-time resolution electron data product available from the LOW/MED units (see Fig. 9). As described below in Sect. 3.3, MagEIS main rate data were sampled at anywhere between 8 and 64 angular sectors per spacecraft spin, where the number of sectors was configurable via ground command. The high-rate (HR) data were sampled at anywhere between 8 and 2048 sectors per spin, again set by ground command. Thus, for a nominal spin period of 11 sec, the maximum achievable time resolution for the main rates was 172 msec (64 sectors), versus 5.4 msec for the HR data (2048 sectors). We note that we typically operated the HR mode with 500–1000 sectors, however, to maintain sufficient counting statistics per sample.

Similarly, for a nominal $0^{\circ }$–$180^{\circ }$ local pitch angle range, the maximum achievable angular resolution in normal mode was $\sim3^{\circ }$ (64 sectors), versus $\sim0.1^{\circ }$ in HR mode (2048 sectors). However, due to the orientation of the local magnetic field relative to the spacecraft spin axis, MagEIS frequently did not observe the full $180^{ \circ }$ range of local pitch-angles on each spin, so that these angular resolution values are somewhat approximate. Moreover, considerations of the instrument field-of-view ($10^{\circ } \times 20^{\circ }$) and the variable sector response must also be taken into account to properly define the maximum achievable pitch-angle resolution. The MagEIS team has not performed a pitch-angle deconvolution analysis (e.g., Selesnick and Blake 2000) that would be necessary to fully characterize the angular response, though such an analysis is possible using the information provided in Sect. 4 below and in the Electronic Supplementary Material.

To define the HR energy channels, an additional lookup table was used, different from the main rate LUT. The general idea was similar, where the primary passband response for each pixel was defined in PHA channel space. Early on in the mission, we used HR LUTs that combined the passband response from several pixels into one HR channel. This is in contrast to the main rate data, where multiple pixels were never combined to produce a single main channel. For example, up until early April 2013, the HR LUT that was used on LOW-A defined 3 HR channels: the first was obtained solely from P2, while the second combined P3 and P4 into one HR channel, and the third combined the four pixels P5–P8. The energy channel calibration factors obtained for these three channels are $E_{0}=[36,62,128]~\text{keV}$ with $\Delta E/E=[47, 79, 110]\%$, i.e., a significantly coarser resolution than the main channels. The HR channels were originally defined in this way to increase the counting statistics at the expense of energy resolution, in an attempt to achieve roughly same counting rates in each of the HR channels. At later times in the mission, after we gained experience with the HR data, we used HR LUTs that defined only one HR channel from one pixel. The maximum possible number of HR channels was hardcoded to 8, with typically 3, 5, or 7 defined via the HR LUT. Like the histogram data, the Geant4/bowtie calibration methods (Sect. 4.1 and Sect. 4.2) were used exclusively to determine the HR flux conversion factors, since the HR channels were not calibrated pre-flight. The conversion factors for each HR LUT are provided in the Electronic Supplementary Material.

**Fig. 11:**
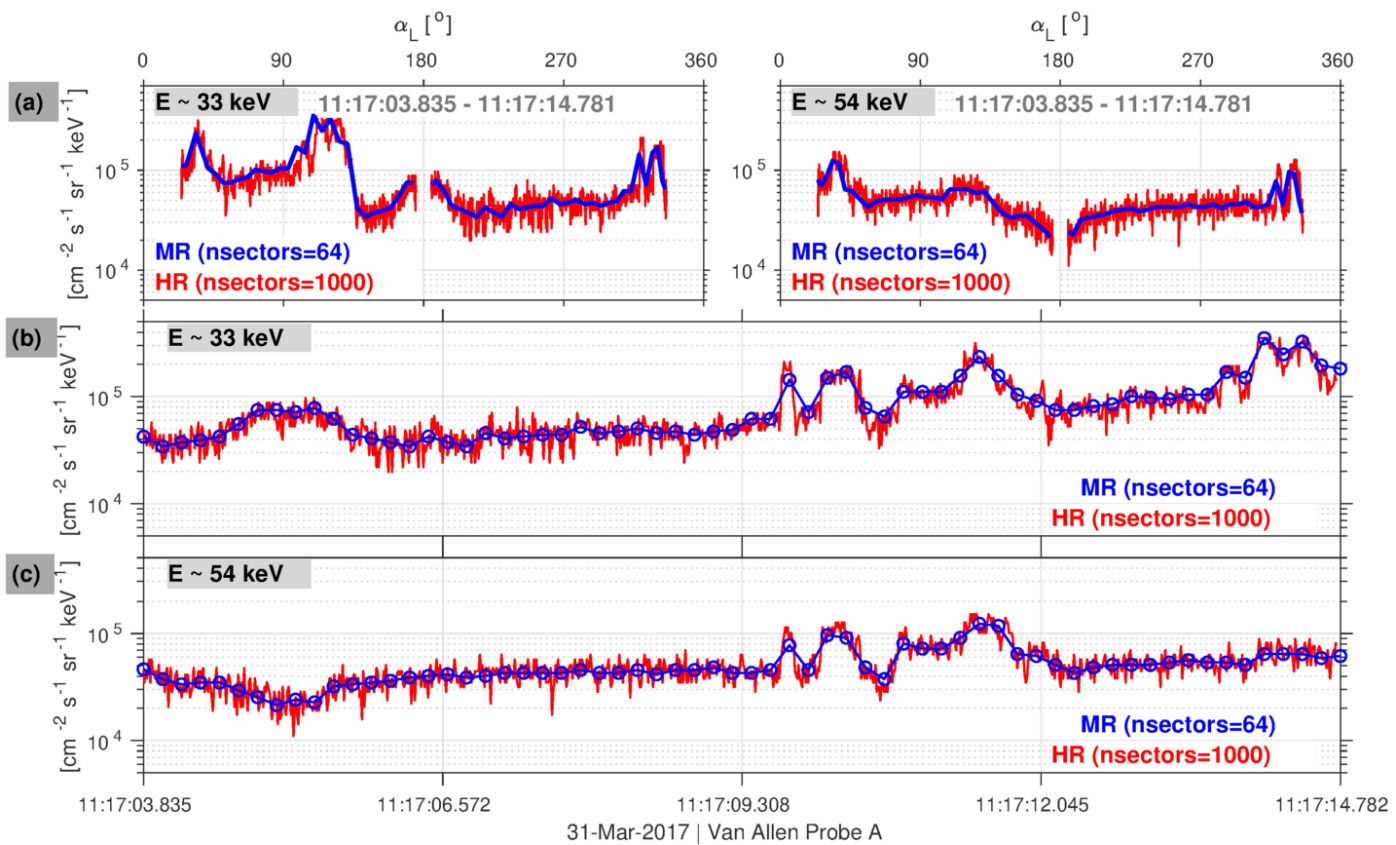
Comparison of the main rate (MR) and high-rate (HR) data products from the LOW-A unit. (**a**) Raw angular distributions (in local pitch angle, $\alpha _{L}$) obtained over one full spacecraft spin ($\sim11~\text{sec}$) for $\sim33~\text{keV}$ (left) and $\sim54~\text{keV}$ electrons (right). (**b**) & (**c**) The same $\sim11~\text{sec}$ of data but now plotted versus time, for $\sim33~\text{keV}$ and $\sim54~\text{keV}$, respectively

The primary goal of the HR data was to detect the effects of wave bursts on the particle distributions, such as those thought to be the cause of electron microbursts. For example, Fig. 11 compares main-rate and high-rate data from LOW-A for the event analyzed in detail in Shumko et al. (2018) and noted in Sect. 2. The authors identified the flux pulses observed just after 11:17:09.308 UTC as microbursts. Note that the time duration of the first pulse ($\sim200~\text{msec}$) is only accurately measured in HR mode (e.g., panel (b)). It is also clear from the figure that the HR data suffers from increased noise due to Poisson counting statistics error, relative to the main-rate data. This is generally true for the HR data taken throughout the mission. We also note that the HR data used a different compression scheme from the main rate data (see Sect. 3.4) and that compression effects are at times noticeable in the HR measurements. Section 2 describes a number of other studies that used MagEIS HR data to obtain high-resolution angular distributions, revealing in detail wave-particle interactions that were previously impossible to observe.

#### Additional HIGH Unit Electron Data Products

The MagEIS HIGH unit electron spectrometers provided additional data products beyond the standard science-mode products (main rates, livetimes, and histograms). These secondary data products were primarily used for trending and evaluating instrument performance and only retained to level 1 in the data files. Two types of singles rates were recorded, the raw “detector singles” rates from each of the 10 detectors, and the “coincidence singles” rates for the 4 pixels (detector pairs) in coincidence in each stack (see Fig. 1). Singles rates consisted of the counts summed across all PHA channels, not just those in the main channel passband (i.e., the main rates). “Direct event” data were also telemetered from both the front and rear detectors in each pixel stack, as described in greater detail in the Electronic Supplementary Material. The direct event data were used extensively in early operations to optimize sensor performance, especially to guide threshold adjustments.

#### Ion Data Products

The primary data product from the HIGH unit ion telescopes was the main rate, similar to the electron main rates. Here, an ion main rate LUT was used to define 32 main channels across the three detectors. In all of the ion MR LUTs used on orbit, the first 21 channels were obtained from the front (MPA) detectors, providing $\sim60~\text{keV}\text{--}1~\text{MeV}$ proton flux in 21 differential energy channels. On telescope A, the 10 remaining main channels were subdivided equally between the two thin rear detectors, with 5 main channels obtained from each of the 2μ and 9μ detectors (the 32nd channel was used as a catch-all, “junk” accumulation bin). On telescope B, the 10 remaining main channels were obtained from the thick rear MSD detector. An additional LUT was used to produce an ion histogram data product from the telescope detectors, providing a subsampling of the full 256 PHA channel space (see the Electronic Supplementary Material for further details). Each detector in the ion telescopes reported a livetime data product, analogous to the electron livetimes described above. Blake et al. (2013) also described 4 ion integral threshold channels, which have not been analyzed in any fashion at the time of writing. We emphasize that the MPA detectors became noisy early on in the mission and consequently these data suffer from considerable quality issues, to the point that they should not be used beyond mid 2013 (see Sect. 6.7).

### Data Sampling: Sectoring and Spin-Accumulation

For all of the science data products described above, each spacecraft spin was subdivided into a fixed number of angular sectors. This parameter, which we refer to as “nsectors,” was configurable via ground command. The main rate sectoring on each unit could be set to its own integer value between 8 and 64, independent of the values used on the other units. The same was true for the histogram sectoring, where possible values were integers from 8 to 32. The livetime sectoring was not configurable and was locked to the main rate sectoring. Similarly, on the LOW/MED units, the derived rate sectoring was locked to the main rates, while on the HIGH unit, both singles rates and the direct event sectoring were fixed to the main rate sectoring. Our general best-practice was to either use the same value for the main rate and histogram sectoring on a given unit, or to differ them by a factor of two (e.g., 64 main rate sectors and 32 histogram sectors). This type of flexibility is advantageous in that it can allow for improved counting statistics on count-poor data products (like the high-energy resolution histogram data), but can present challenges when simultaneously using two data products that have different sectoring parameters (e.g., when using the histogram data to background-correct the main rates).

In addition to the sectoring parameter, each of the science data products could be accumulated in a given sector over one or multiple spacecraft spin periods. This parameter, which we refer to as “nspins,” was also set via ground command and every data product could be set to its own integer value, independent of the others. Again, this allowed for maximum flexibility in optimizing sensor performance, but complicated the downstream data processing. Note that, since the natural time cadence of the MagEIS science data was tied to the spacecraft spin period, the time cadence was variable and not uniform/fixed. Housekeeping and status data were output at a fixed time cadence, typically 10 sec.

Table 1 shows the major changes that were made to the electron main rate and histogram accumulation parameters during the mission. Aside from a few brief instances of $\text{nspins} = 2$ on LOW-A and HIGH-A during the very early commissioning phase, the main rates were accumulated over 1 spin for the duration of the mission. In October 2013, a new compression scheme was implemented on the Probe’s solid-state recorder that was used to store data between downlinks. This enabled instrument teams to increase their telemetry throughput and ultimately downlink more data from the instruments. The MagEIS sampling parameters thus underwent a significant change between 04 and 10 Oct 2013 to make use of this increased allocation. Additional telemetry became available in June 2014 and a second round of major changes to the various sampling parameters occurred on 27 Jun 2014. Table 2 shows the sectoring parameters for the high-rate data from the LOW/MED units. As noted above, high-rate data were obtained most frequently on the LOW-A unit (above $L = 4$), with sparser availability on the other units.

**Table 1:** Major Changes^a^ to the Electron Main Rate (MR) and Histogram (HG) Accumulation Parameters (nsectors and nspins^b^)

	nsectors (MR)		nsectors (HG)		nspins (HG)
	LOW/MED	HIGH	LOW/MED	HIGH	LOW/MED/HIGH
04 Oct 2012^c^	20→27	20→16	20→27	20→16	8→12
04–10 Oct 2013^d^	27→50	16→32	27→25	(16)	12→6
17 Jan 2014	(50)	32→50	(25)	16→25	(6)
27 Jun 2014	50→64	50→32	25→32	25→32	6→2

^a^There were several brief intervals of deviations from the displayed values, due to unintended commanding

^b^Main rate nspins values are not shown (nspins = 1 for the duration of the mission)

^c^There were many parameter changes prior to this date during commissioning (not shown)

^d^The parameters were adjusted throughout this time interval

**Table 2:** Changes to the Electron High-Rate Sectoring Parameter^a^

Date	Unit	nsectors
2012/10/07	LOW-B, M75-B	100→300
2012/12/13	LOW-B	300→1800
2013/01/04	LOW-A	300→1000
	LOW-B	1800→1000
2013/07/13	LOW-B	1000→360
	M75-B	300→180
2013/08/16	LOW-A	1000→360
	M75-A	300→180
2013/12/20	LOW-A, LOW-B	360→500
2015/09/17	M35-A, M35-B	300→180
2015/10/06	M35-A, M35-B	180→2048
2015/10/15	M35-A, M35-B	2048→1960
2016/05/16	LOW-A	500→1000
2017/07/30	M35-A	1960→1000
	M75-A	180→1000
2017/08/05	M35-B	1960→180

^a^High-rate data were always accumulated over 1 spin

The histogram data were by far the largest consumer of the MagEIS science data telemetry allocation, constituting roughly 90% of the allocation for a given unit. Thus, it was not possible to lower the accumulation time to 1 spin and stay within telemetry constraints. As such, the histogram data were generally obtained at reduced angular and temporal resolution relative to the main rates. As a side note, if the electron PHA spectra were telemetered at full resolution (256 PHA channels in 64 sectors at 1 spin cadence), this would have resulted in a roughly order-of-magnitude increase in telemetry requirements, all other things being equal.

It is important to consider how the low-resolution histogram data obtained early in the mission impacted the quality of the background-corrected main rate data (see also Claudepierre et al. 2015, 2019). For example, with a $\sim2~\text{min}$ accumulation time ($\text{nspins} = 12$), any steep radial gradients in flux were smoothed over, relative to what was measured in the main rates. Between $L = 1$ to 4, where the spacecraft were crossing $L$ the fastest, 2 minutes was roughly equivalent to a $\Delta L = 0.05$ to 0.1, a small but not insignificant constraint. More impactful were changes in the local pitch angle in a fixed sector as the spacecraft moved through the low $L$ region, where the rapid motion in $L$ and the steep gradients in the magnetic field conspired. Here, with a histogram accumulation time of $\sim2~\text{min}$ and $\text{nsectors} = 16$ (as was the case in the early portion of the mission) the local pitch angle could change by as much as $\sim5^{\circ }$ in a fixed sector over the 2 minutes. This is important because the low $L$ region is where the histogram data are the most useful, enabling the quantification and removal backgrounds from the intense inner proton belt. These sampling impacts were mitigated as the mission progressed by increasing the histogram sectoring while reducing the accumulation time interval. This, of course, came at the expense of increased counting statistics error in the histogram data.

### Data Processing

MagEIS has 4 formal data levels, level 0 through level 3. The following sections describe the level-to-level processing of the sensor data.

#### Level 0 to Level 1

Raw data from each MagEIS unit was organized into binary “payload telemetry packet” (PTP) level 0 data files on each mission day. Each MagEIS data type (see Fig. 9) was organized into a separate PTP file and assigned a unique “Application Identifier” (APID). A level 0 file consisted of a series of packets, each of which contained header information (packet length, spacecraft ID, etc.) followed by the telemetry data. The primary task in this first level of data processing was to “unpack” the raw binary data and combine similar data types in daily level 1 files organized by UTC day. The general philosophy was to process the data as little as possible in this step and not to use LUTs in any of the level 0 to level 1 processing. One important part of this unpacking was to arrange and reorder all of the level 1 data arrays with respect to increasing energy channel/pixel number. This was necessary since some of the level 0 data types were organized using hardware/electronics numbering schemes that did not necessarily correspond to the more natural arrangement in terms of increasing pixel/channel number.

In order to conserve telemetry, all of the level 0 MagEIS data were compressed using floating-point compression except for the HIGH unit direct event data, which was not compressed. Thus, the first step was to decompress the data. The data counters were 24 bits in depth and were compressed to 10 bits for all of the data types except the high-rate data, where a 16-to-8 bit scheme was used. All of the decompressed particle counts data were then converted into raw count rates in each energy bin, using the duration of each spin sector. No livetime (or other timing) corrections were performed in the level 0 to level 1 processing. The raw instrument time variable (mission elapsed time) was converted into UTC/Epoch time at this step and a spin-averaged rate was computed for each of the count rate data products (e.g., main rate, histogram). We refer to this as a “spin-set” average, since some data products were accumulated over multiple spin “sets” (e.g., when $\text{nspins}>1$). The UTC/Epoch time tag corresponds to the start of the spin that begins an accumulation interval of nspins. The raw counts were also retained in the level 1 files, so that Poisson (counting statistic) errors could be computed in the downstream processing.

All of the converted level 0 data were organized into daily (UTC) level 1 “Common Data Format” (CDF) files for each unit. For each unit, there were two primary level 1 CDF data files, one that contained the science data on the time base of the spacecraft spin-period, and another that contained the housekeeping and status data, which were output on a fixed time cadence (typically 10 sec) unrelated to the spacecraft spin period. The direct event data, taken only on the HIGH unit, was organized into a separate level 1 CDF file, as was the high-rate data, which was taken only on the LOW/MED units. We note that each unit had its own time base, independent of the other units. Moreover, in the HIGH unit data files, the electron and ion data products were on separate time bases. No attempt was made to merge together any of the electron data products from the various units at level 1.

#### Level 1 to Level 2

The high-level steps in this stage of the processing were to: (1) apply livetime corrections to the raw count rates; (2) apply background corrections to the livetime corrected count rates (electrons only); (3) convert the livetime- and background-corrected count rates into physical flux units; and (4) assign energy channel centroids in physical units. All of these conversions and processing steps were done on a sector-by-sector basis. Calibration files were generated for each main rate LUT for the flux conversions and energy channel assignments (see Sect. 4). A spin-averaged flux data product was also produced by averaging the sector-resolved fluxes over the spin. These fluxes and support data products were then organized into daily level 2 CDF files, one for each unit, which we refer to as the “unit-by-unit” level 2 files.

As described in Sect. 1, the LOW, M75, and HIGH units were all positioned at $75^{\circ }$ with respect to the spacecraft spin axis so that they were commensurate in their mutual angular coverage. Thus, an additional data file was created, the “merged” level 2 file, which combined electron data from these three units onto a common time base. This merged level 2 file does not contain the sector resolved electron fluxes, only the spin-averaged. Since the different MagEIS units were mounted at different locations on the spacecraft and thus at different locations in spin phase, the MagEIS team determined that it was not productive to attempt to combine the three units into a merged level 2 flux data product aligned on spin-phase angle. This merging is more naturally done at level 3, where the sector-resolved fluxes for each unit are first converted into pitch-angle-resolved fluxes. We do note, however, that the sector-resolved electron fluxes are retained and available in the unit-by-unit level 2 files.

Note that since the ion telescopes were housed in a single MagEIS unit (the HIGH unit), there is no merging of the ion flux data. Both the unit-by-unit HIGH unit level 2 file and the merged level 2 file contain identical copies of the spin-averaged and sector-resolved ion flux data products. In addition, data from the M35 unit were not included in the merged data files, as they were generally redundant with the M75 data (see Sect. 8.9). The primary data variables in the level 2 files are described in greater detail in the Electronic Supplementary Material.

#### Level 2 to Level 3

The primary task in the level 2 to level 3 processing was to convert the sector angle (i.e., spin-phase angle) into local pitch angle. Again, as for the level 2 data files, there are both unit-by-unit level 3 files and a merged level 3 file. In the merged file, the fluxes are binned into a fixed number of local pitch-angle bins, $N$, such that $d\alpha = 180^{\circ }/N$ with the pitch-angle bin edges given by $\alpha _{i}=[ (i-1) \cdot d\alpha , i \cdot d\alpha ) $ for $i=1,2,\ldots ,N$. The two half spins (pitch angles $0\text{--}180^{\circ }$ and $180\text{--}360^{\circ }$) were combined and binned into the $0\text{--}180^{\circ }$ local pitch angle range. For the electron fluxes, $N=11$, and for the ion fluxes, $N=15$, in the merged level 3 data files. The primary data variables in the level 3 files are described in greater detail in the Electronic Supplementary Material.

Importantly, the unit-by-unit level 3 data files also contain the “unbinned” pitch-angle data, where the instantaneous sector angle was converted to local pitch angle. Here, the pitch-angle value assigned to each sector corresponds to the pitch angle at the center of the sector. Half-spin and full-spin unbinned angular distribution variables are available in these data files. These unbinned/full-spin pitch-angle data are useful if end users wish to construct their own pitch-angle binning, to examine non-gyrotropic effects, etc. Omnidirectional data products are not included in any of the MagEIS level 3 data files, given the assumptions that must be made regarding the portions of the angular distributions that were not sampled (e.g., the loss cone). A masking procedure was also applied to the ion telescope data at this stage to remove light contamination (see Sect. 6.8).

### Electron High-Rate Mode Operation and Coordinated Campaigns

We generally operated the MagEIS suite such that only one unit on one Probe was in high-rate mode at any given time. This was typically the LOW-A unit, where the concept of operation was to command it into HR mode at higher $L$ on each orbit, so that histogram data were recorded at lower $L$ to enable background corrections in the inner zone. We initially set this threshold $L$ value to 3 but we changed it to $L=4$ on 03 Apr 2014 due to the significant amount of bremsstrahlung background contamination that can be present in the $L=3\text{--}4$ region. Until 15 May 2016, these LOW-A HR data were acquired in 500 sectors, after which time we increased the sampling to 1000 sectors (see Table 2). This commanding was handled through automated scripts; the MagEIS suite did not have an HR-mode trigger. These scripted commanding procedures failed occasionally for various reasons, which left the LOW-A unit in HR mode continuously, at times for multiple days. The availability of such data can be found in the Electronic Supplementary Material where we provide summary plots showing the MagEIS instrument mode over the duration of the mission. In addition to the LOW-A unit, other LOW/MED instruments were operated in HR mode at various times throughout the mission (see the instrument mode summary plots in the Electronic Supplementary Material for availability).

There were several intervals of note where HR-mode data were taken to support coordinated campaigns. For the first BARREL balloon campaign (Millan et al. 2013), the MagEIS team commanded the LOW and M75 units on both Probes into HR mode above $L = 4$ between 05 Jan 2013 and 26 Feb 2013. We also supported the second BARREL campaign (21 Dec 2013 to 21 Feb 2014), but only the two LOW units were commanded into HR mode and the turn-on point was lowered to $L = 3$.

The MagEIS team also participated in two Van Allen Probes coordinated burst mode campaigns to take high resolution measurements during close encounters of the two Probes. The first occurred on 02 Feb 2015, when the LOW-B unit was briefly commanded into HR mode during the close approach (LOW-A was already in HR mode as part of its normal operations). The second occurred between 08–10 Apr 2015, where LOW-B was commanded into HR mode at $L>4$ on every orbit for these 3 days (i.e., to coincide with LOW-A HR mode). In addition, in August 2017, a coordinated burst mode campaign was undertaken between the Van Allen Probes and Arase/ERG (JAXA) mission science teams to take advantage of physical conjunctions between the spacecraft. The MagEIS team supported this by commanding the LOW, M35, and M75 units on Probe-A into HR mode above $L = 4$ between 30 Jul 2017 and 30 Aug 2017. There were also brief instances ($\sim30$ minutes) of HR mode data taken on Probe-B on 13 and 22 Aug 2017 during the anticipated closest approaches of the two Probes (i.e., all 6 LOW/MED units in HR mode).

The MagEIS team initiated a campaign where HR mode data was taken for several months (17 Sep 2015 to 10 Dec 2015) over the entire orbit on both M35 units. Various sectoring configurations were experimented with during this time (see Table 2). One item of note here is that during this campaign, we uncovered a timing issue in the flight software when the maximum possible HR sectoring (2048 sectors per spin) was used. As described in the Electronic Supplementary Material, this led to spurious count accumulation in the first $\sim100$ sectors of each spin, which impacted these sectors for the first $\sim10$ days of M35 HR data taken during this campaign before the issue was resolved.

## Data Calibration

The calibration of the MagEIS data proceeded in several stages as the mission progressed. Extensive pre-flight calibrations were conducted by exposing the instruments to radioactive sources in the laboratory, as described in Blake et al. (2013). The PHA main channel boundaries for each pixel were determined from these tests over the range of temperatures expected on orbit. These channel boundaries were progressively optimized in the early part of the mission using on-orbit histogram data, which led to several revisions of the main rate LUTs. The histogram LUTs were also revised early on, once we had experience with the on-orbit data, though these changes were less frequent than for the main-channel LUTs. The HIGH unit was considerably more complex than the LOW/MED units, since it used coincidence between the two detector planes. Optimizing its performance took longer and was more involved, particularly because front detector histograms were not available (unless the unit was specifically commanded into such a state, in which case science data was unavailable). Thus, we relied heavily on the direct event data to optimize the performance of the HIGH unit, which resulted in several revisions to the instrument threshold settings. But most important of all for the calibrations were the Geant4 computer simulations of the instruments’ response, which were used to obtain flux conversion factors through a “bowtie” analysis as described below.

### Sensor Response: Geant4 Simulations

The schematic diagram of MagEIS electron spectrometer operation (Fig. 1) shows how to calculate an approximation to the response of each unit: the geometry of the slit and the collimator will give an angular response for electrons entering the chamber, and the distance from the slit to each pixel combined with the strength of the magnetic field will give the energy response for the electrons that are steered by the chamber field to each pixel. However, in the actual sensors as flown, departures from this idealized representation had significant effects on the response. Characterization of this began before launch with simulation of trajectories through a more realistic model of the magnetic field, using the LORENTZ magnetic-circuit code (Yildir et al. 1993; Asi 2001) to calculate the fields in the magnets, yoke, and chamber, and the SIMION code (Dahl 2000) to trace electrons through this field in the chamber (see Blake et al. 2013).

In order to account for the scattering of electrons off solid parts of the sensor and for non-ideal collection of electron energy in the detectors, we used the Geant4 Monte Carlo radiation transport code (Allison et al. 2016). This code package transports energetic particles through an arbitrary, user-defined 3D geometry, simulating the trajectories of individual particles through vacuum or different materials with or without an electromagnetic field. Stochastic processes, such as scattering, generation of secondary particles, energy loss fluctuations, etc., are represented by sampling from the relevant probability distribution functions. The end product is a set of simulated particle histories that realistically represent the behavior of an ensemble of incident particles.

**Fig. 12:**
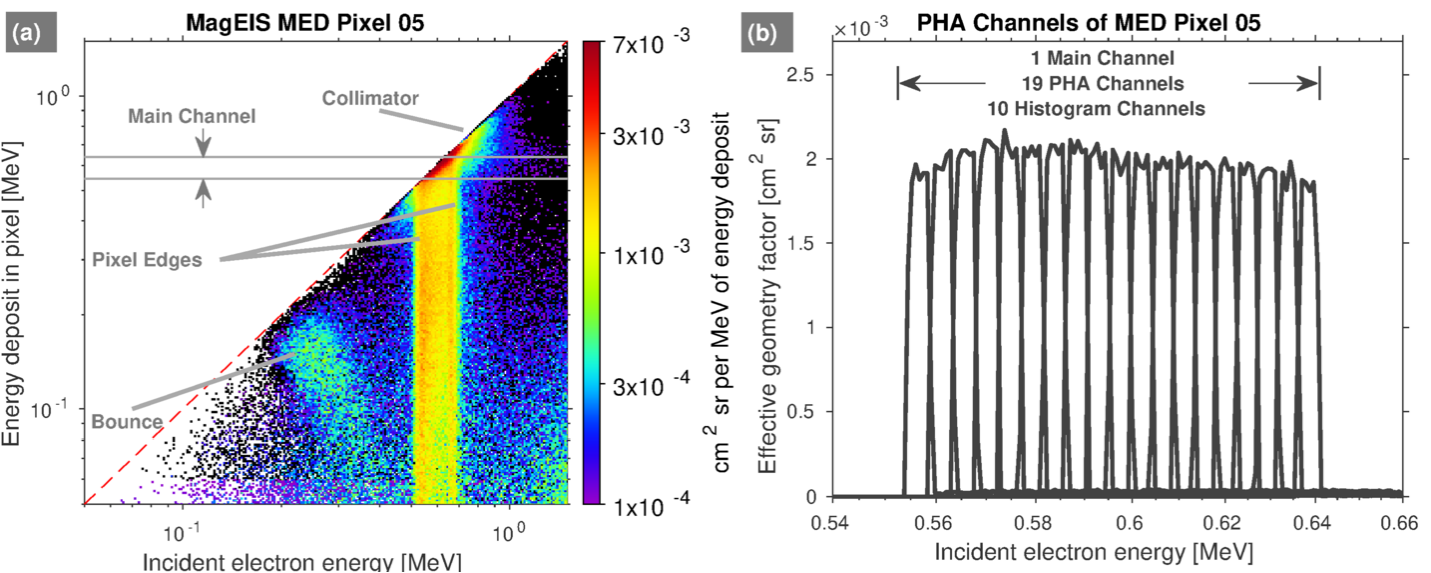
(**a**) Energy deposit in a single MED pixel as a function of incident electron energy, calculated with the full Geant4 model. The main channel boundaries (cuts on the energy deposit) are labeled, along with non-ideal contributions to the energy deposit (bounce, collimator, pixel edges – see text). (**b**) The response of the individual PHA channels that constitute the main channel delineated in panel (**a**), showing the maximum energy resolution and separation achievable by the sensor

**Fig. 13:**
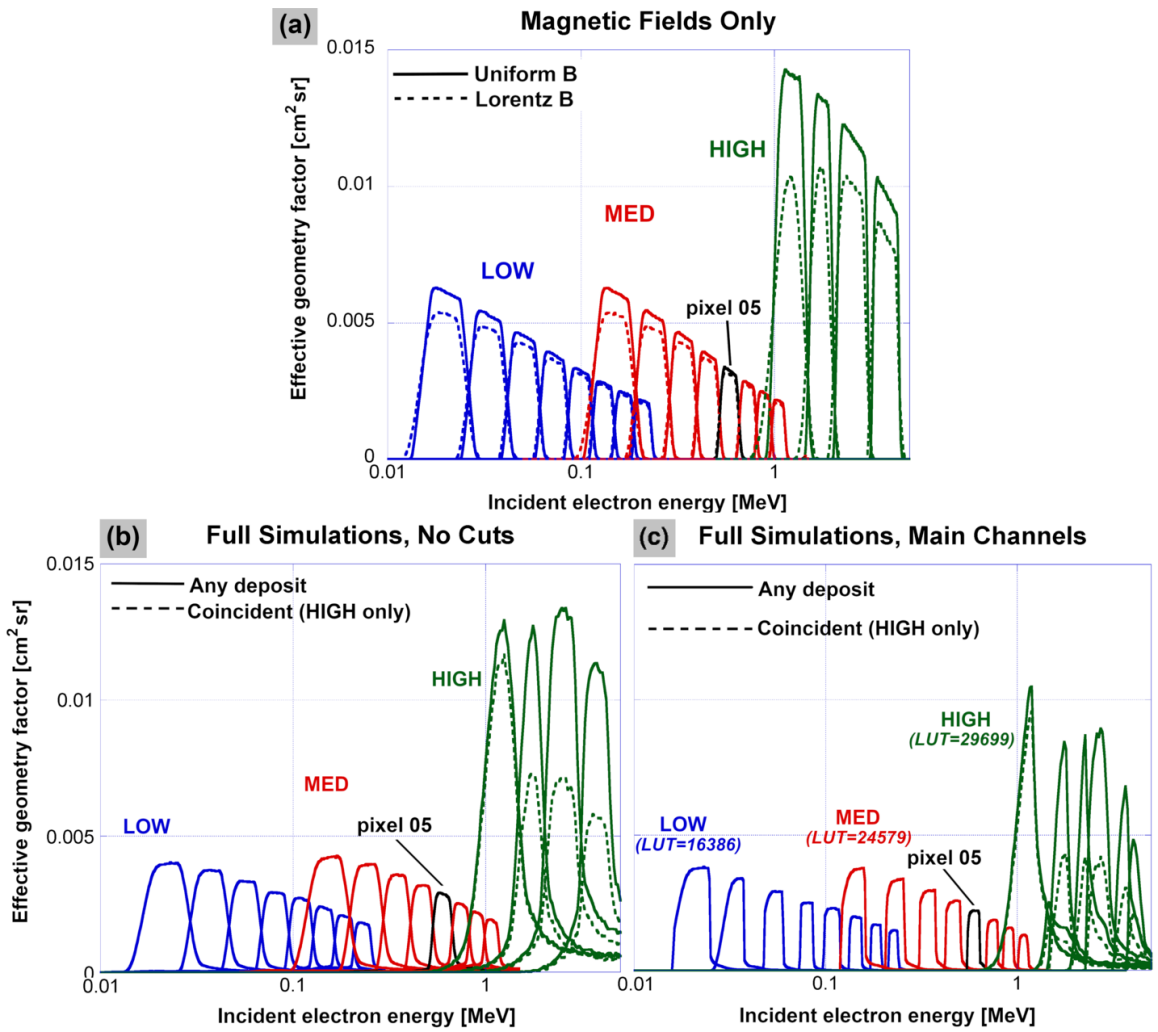
(**a**) Simulated response of each pixel for the three units, considering only transport from the slit to the detector plane through the magnetic field in the chamber. Pixels P1–P8 are shown for LOW and MED (P0 omitted) and P0–P3 are shown for HIGH; MED P5 (e.g., Fig. 12a) is shown in black rather than red. Solid lines are for simulations conducted with a uniform chamber magnetic field of 550, 1600, and 4800 Gauss for LOW, MED, and HIGH, respectively. Dashed lines are for simulations conducted with the LORENTZ field as modeled before launch for each unit and scaled to those three mean values. (**b**) Same as in (**a**), but with full realism of the simulation, including the LORENTZ field and non-ideal effects (e.g., scattering). Solid lines represent response to electrons depositing any amount of energy in the pixels. For the HIGH unit, the dashed lines show the effect of the coincidence requirement. (**c**) Same as in (**b**), but with a set of main rate LUTs applied to the simulation results

In the simulations reported in this section, electrons covering the range of energies appropriate to each unit (LOW, MED, HIGH) were distributed isotropically over a rectangle encompassing the aperture of each collimator. Very energetic electrons or protons might penetrate the spacecraft body and the yoke of each unit and also deposit energy, but for the purposes of the response calculations summarized in this section (i.e., Fig. 12 and Fig. 13) we only consider “foreground” electrons arriving in and near the aperture. The bowtie analysis discussed in Sect. 4.2 is based on these same simulation results for the LOW and MED units. For HIGH, we based that analysis on simulations of electrons up to 10 MeV illuminating the entire volume of the sensor from all directions, to approximate the contributions of penetrating electrons to the response, but this made little difference to the bowtie parameters calculated and so the HIGH unit curves in Fig. 13 that are discussed below are representative of that sensor’s bowtie input as well. We rely on background correction as discussed in Sect. 3.2.4 to isolate the foreground signal, from which we extract the incident flux using the calculated responses.

Figure 12a shows the result of a Geant4 simulation for one pixel from a MED unit. A vertical cut through the plot represents the response as a function of energy deposit for the given incident energy. As outlined in Sect. 1.2, in an ideal magnetic spectrometer one would expect to see nonzero response only along the diagonal where energy deposit equals incident energy, and only for those energies that would be steered from the slit to that pixel by the magnetic field. This is indeed where the most intense response (red colors along the diagonal) is seen. Horizontal lines labeled “main channel” are the energy-deposit limits used to isolate the cleanest possible sample of electrons traveling to the pixel along nominal trajectories through the chamber, via the main rate LUT. However, non-ideal response is seen in several parts of the plot, as labeled (bounce, collimator, pixel edges).

The “bounce” portion of the response in Fig. 12a represents electrons of lower energy that strike the detector plane at a pixel closer to the slit, but that then bounce off with some fraction of their incident energy and make another arc through the chamber to reach this pixel. This manifests as a faint diagonal band, extending down and to the right, which is expected because a higher-energy primary electron will first strike the detector plane farther from the slit, and so the bouncing electron that reaches the pixel plotted here must have lower energy in order not to overshoot it.

The “collimator” portion of the response represents higher-energy incident electrons from outside the clear field of view that glance off the collimator and enter the slit at a steeper angle that carries them to this pixel instead of one farther from the slit.

The “pixel edges” show the deficit in energy deposit for electrons that strike near the edges of the pixel, so that parts of the paths inside the detector of these electrons and their secondary particles leak out of the sensitive volume of the pixel’s silicon. Such “edge-cutting” events result from primary electrons with energies at the upper and lower limits of the nominal pixel energy range that strike near the physical edges of the rectangular pixel farthest from and closest to the slit respectively, and with all energies in between near the other two edges.

The remainder of this section builds up the Geant4 simulation with increasing levels of realism and shows the effects of such changes on the response integrated over all energy deposits as a function of incident electron energy.

Figure 13a shows how the three units (LOW, MED, HIGH) would respond with no scattering of electrons either upstream of the slit in the collimator or downstream in the chamber: that is, collimation is taken to be perfect into the slit and, in the chamber, electrons move through the magnetic field until they either strike a pixel, or miss and are discarded from the simulation. Note that, for the HIGH unit, the relevant target for a strike is the smaller front detector in a pixel stack, not the larger outline of the rear detector. In Fig. 13a, for each unit and a uniform field, the decline of response with increasing incident energy is expected because the “beam” from the slit inside the chamber spreads out as it travels farther from the slit, and so a smaller fraction of electrons entering the slit will actually fall on the more distant (higher-energy) pixels. In the realistic LORENTZ model, the field flares somewhat toward the edges of the rectangular magnets, and the component not perpendicular to the magnet faces can steer some electrons out of the beam, reducing response. Since lower-energy electrons spend more of their trajectories near the edges of the magnets, they are more affected by this fringe effect, and the less-uniform field of the HIGH unit compared to the LOW/ MED (5% vs. 0.5% per Blake et al. 2013) means the effect is greater in the HIGH unit. All of this is consistent with the trends in Fig. 13a.

Figure 13b shows the changes in response with the addition of realistic interactions with the solid material of the units, including all of the effects of scattering and non-ideal energy deposit that were called out in Fig. 12a. Besides a general reduction in response due mainly to collisions with the baffles inside each chamber, there is an increase rather than a decrease in response as a function of incident energy for most pixels. This is due to higher-energy electrons from slightly outside the ideal field of view that scatter off the collimator elements and then enter the slit at an angle that takes them to the given pixel. An additional complication for the HIGH unit is the use of coincidence, where both the front and rear detectors in each pixel stack must be triggered for an event to be tabulated. In order to suppress electronic noise in the detectors, the discriminator thresholds of the front detectors (see Table 3) had to be set not far below the most probable energy deposit for electrons penetrating to the rear detector, resulting in the loss of some valid events. This significantly reduces the response in the HIGH pixels, with the effect of the thresholds (using the HIGH-A unit values valid after 17 Oct 2013) shown by dashed lines in Fig. 13b (only a minimal front-detector energy deposit, $>30~\text{keV}$, is required for the solid lines). The reduction in response due to application of the front-detector threshold to calculate the dotted curves in Fig. 13b and Fig. 13c is less for pixel 0 than for the other pixels of HIGH-A mainly because, as can be seen in Table 3, its threshold is lower than the others.

**Table 3:** HIGH Electron Spectrometer Front-Detector Thresholds for Each Pixel (P0–P3), in keV

Unit	Valid dates^a^	P0	P1	P2	P3
HIGH-A	2012/09/06–2013/07/03	114	131	114	93
HIGH-A	2013/10/17–2019/10/14	81	93	93	93
HIGH-B	2012/09/06–2013/07/03	67	52	121	112
HIGH-B	2013/08/03–2019/07/16	62	52	82	68

^a^For each HIGH unit, there was a time interval in which there were frequent changes to the thresholds (see Sect. 6.4); values are not provided during these intervals

In addition, we found it necessary to adjust the intensity of the LORENTZ magnetic field used in the Geant4 models, as indicated in Table 4, in order to make the simulations line up at the energy deposits measured for each pixel (with both laboratory and flight data). This scaling of the magnetic field is done on a pixel-by-pixel basis, which leads to a “hybrid” response function for each unit, i.e., a separate response function is produced for each pixel and then merged together with all pixels from a given unit – see the Electronic Supplementary Material for more details. We emphasize that we adopted the hybrid response function approach between MagEIS data release 04 (“rel04”) and the “final” release; prior to and including data release 04, a single field scaling was used for all pixels in a given unit. The necessity for this scaling of the LORENTZ magnetic field may be explained by differences in the on-orbit thermal environment versus what was assumed in the pre-flight testing/modeling, and/or non-uniformities in the chamber magnetic field (see Sect. 8.6). For definiteness, we note that fixed chamber field scaling values of 106.5%, 102%, and 100% were used for all pixels in Fig. 12 and Fig. 13 for LOW, MED, and HIGH units, respectively. On the scale of Fig. 13, these fixed field scalings would be indistinguishable from the hybrid, pixel-by-pixel scalings, if the latter were shown instead.

**Table 4:** Parameters Used in Electron Sensor Response and Bowtie Analysis

	B-field scaling^a^ [%]	Temp. [^∘^C]	Power law indices	Exponential energies [MeV]
LOW-A	102 to 106.5	−10	−0.5,−1,−1.5,−2,−2.5,−3,−3.5,−4	0.02,0.04,0.06,0.08,0.1
LOW-B	102 to 106.5	−10	“ ”	“ ”
M35-A	100 to 100	−11	0,−0.5,−1,−1.5,−2,−2.5,−3,−3.5,−4	0.1,0.2,0.3,0.4,0.5,0.6,0.7,0.8,0.9,1.0
M35-B	101 to 103	−9	“ ”	“ ”
M75-A	101 to 102	−10	“ ”	“ ”
M75-B	101 to 103	−7.5	“ ”	“ ”
HIGH-A	94 to 111.5	−5	−5,−6,−7,−8	0.25,0.34,0.43,0.51,0.60
HIGH-B	94 to 110	−5	“ ”	“ ”

^a^For each unit, a different scaling is used for each pixel in the “hybrid” response function (see Electronic Supplementary Material). The values shown here indicate the range of scalings used

The primary data product from MagEIS, reported at the highest temporal and angular resolution, is the set of main channels from each unit. Figure 13c shows the response when a set of main rate LUTs for the LOW-A, M75-A, and HIGH-A units are applied to the simulated response of each sensor. These response curves are used to define the conversions from main-channel count rate to flux using the bowtie analysis described in Sect. 4.2, though we retained the full energy and angular response for each pixel from the Geant4 simulations for use in further analyses, as needed. These MagEIS electron spectrometer 3D (energy-angle-angle) response files are available in a data repository at the link provided in the Acknowledgments. The angular response, as determined by Geant4, is described in greater detail in the Electronic Supplementary Material, where we find that the response is well-approximated by the nominal $20^{\circ }\times10^{\circ }$ field-of-view. However, there are subtleties with regard to the sensors’ angular response and how it depends on the incident particle energy. For example, in Fig. 13, the overall decline of the response with energy when integrated over all incidence angles is primarily an angular-response effect.

As described in Sect. 3.2.4, the MagEIS units output the additional histogram data product with very fine energy-deposit resolution. Figure 12b shows the simulated response of the individual PHA channels within the main-channel passband of pixel 5 of the M75-A unit (same pixel as in Fig. 12a), of which adjacent channels are combined into a histogram channel using the histogram LUT. The very fine slicing of incident energy necessary here reduces statistics so that the flattop responses are somewhat noisy, but the width and separation are clearly visible in the figure.

### Sensor Response: Bowtie Analysis

The Geant4 simulations described above are tabulated into 3D (energy-angle-angle) response functions according to the draft guidelines provided by the COSPAR Panel on Radiation Belt Environment Modeling (O’Brien and Bourdarie 2011; also available at https://prbem.github.io/). Essentially, these guidelines specify that the response is provided as a table of effective area ($\text{cm}^{2}$) on a grid in energy, polar angle, and azimuthal angle for each output channel of the sensor. We only provided responses for incident electrons, though we note that the proton response was also simulated for the HIGH electron spectrometer unit. This proton response was only used to better understand the shape of the HIGH unit histograms in the inner zone when the measurements were contaminated by high-energy protons (cf., Claudepierre et al. 2019). For the electron response, the tabulation of the Geant4 simulations is done in two stages. First, the tabulation is performed in terms of PHA channels, using gain and offset values that convert from deposited energy to PHA channels on a 0–255 discrete scale. Then, using the main-rate LUTs, the 3D tables for the many pulse height channels are summed into 3D tables for the main-rate channels, which are those that were telemetered to the ground. This summation of the 3D tables is functionally equivalent to the channel summation performed in the on-board electronics via the LUTs. The largest uncertainty is in the gain and offset that relate the simulated energy deposits to the PHA channel.

As described by Blake et al. (2013), the gain and offset derived for each MagEIS detector are sensitive to the ambient temperature in the yoke, which is plotted in Fig. 3 for all 8 magnetic spectrometers. Note the large ($\sim10~^{\circ }\text{C}$) variations in temperature. Clearly, one would not want to attempt to account for these temperature variations in the MagEIS flux conversions, as this would lead to complicated, time-dependent flux conversions and energy channel definitions. Thus, a fixed yoke temperature is assumed for each unit (indicated in Fig. 3 and Table 4) and the pre-flight gain and offset obtained at this temperature is assumed in the bowtie analysis.

For many practical purposes, the 3D tabulated response is unwieldy and it is more convenient to assign a nominal energy centroid, energy width, and flux conversion factor to each channel. To accomplish this, we perform a bowtie analysis (Selesnick and Blake 2000; O’Brien et al. 2015b). Specifically, we determine the effective energy centroid ($E_{0}$) and flux conversion factor ($G_{0} \Delta E$) that approximate the true isotropic channel response as an ideal differential channel with a delta-function response in energy. Here, $G_{0}$ is the effective geometric factor, which accounts for both the geometry factor and the efficiency of detection (i.e., $G_{0} \sim \epsilon G$), and $\Delta E$ is the energy channel width. The isotropic response $\bar{R}(E)$ is:

1 $$ \bar{R}(E)=\int _{0}^{\pi } \int _{0}^{2\pi } A(E,\theta ,\phi ) d \phi \sin \theta d \theta $$

where $A(E,\theta ,\phi )$ is the effective area in $\text{cm}^{2}$, $\phi $ is the azimuthal angle, and $\theta $ is the polar angle. The bowtie analysis then finds values of $E_{0}$ and $G_{0} \Delta E$ that approximate:

2 $$ j(E_{0}) G_{0} \Delta E \approx \int _{0}^{\infty } \bar{R}(E) j(E) dE $$

for a range of likely isotropic spectra, $j(E)$. Procedurally, we start by choosing a set of candidate spectra by inspection of MagEIS data using nominal pre-flight flux conversion factors based on simplified response functions. Observed spectra from each unit (LOW, MED, and HIGH) are then fit with exponentials and falling power laws, with the obtained fitting parameters shown in Table 4. For example, for the LOW-A unit, there are 8 assumed power law spectra and 5 exponential spectra. We then have 13 spectrum choices $j_{i}(E)$ for $i =1,2, \ldots 13$, each of which has its own result when integrated with the sensor response:

3 $$ y_{i} \approx \int _{0}^{\infty } \bar{R}(E) j_{i}(E) dE $$

We next choose the $E_{0}$ that minimizes the root-mean-square (RMS) spread of $y_{i}/j_{i}(E_{0})$ over the 13 spectra. We adopt as $G_{0} \Delta E$ the mean of $y_{i}/j_{i}(E_{0})$ at the selected $E_{0}$. We can then use $G_{0} \Delta E$ to convert an observed count rate $r(t)$ into an isotropic flux at $j(E_{0};t)$:

4 $$ r(t) \approx \int _{0}^{\infty } \bar{R}(E) j(E;t) dE \Rightarrow j(E_{0};t) \approx \frac{r(t)}{G_{0} \Delta E} $$

Finally, we retain the RMS spread of $y_{i}/j_{i}(E_{0})$ as an indicator of the error in flux computed this way; we usually express this as a relative flux error (e.g., as $\Delta G_{0}/G_{0}$). Note that this flux error does not account for errors in the assumption of isotropy, errors in the sensor model, errors in the family of spectra chosen, nor temperature variation in the gain, offset, or field strength.

**Fig. 14:**
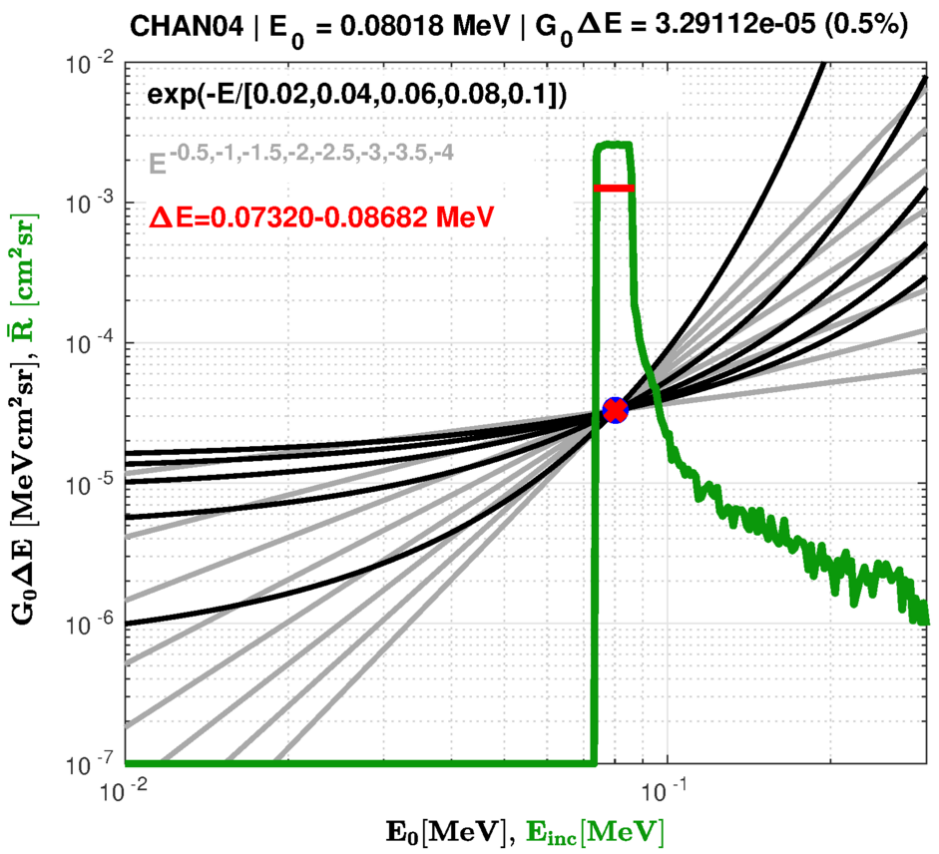
Example of the bowtie analysis for a single main rate channel ($E_{0} \approx 80~\text{keV}$) from LOW-A (LUT 16386). The green curve is the simulated isotropic pixel response and the black and grey curves are the candidate exponential and power law bowtie spectra ($= y_{i}/j_{i}(E_{0})$), respectively. The red line marks the energy channel width and the red dot marks the energy channel centroid, $E_{0}$. Note that the labels on the horizontal and vertical scales are different for the isotropic response and the candidate spectra. The response curve is the simulated isotropic response ($\bar{R}$) plotted against incident electron energy ($E_{\mathrm{inc}}$), while the candidate spectra are bowtie response parameter ($G_{0} \Delta E$) vs. bowtie energy centroid ($E_{0}$)

Figure 14 shows the final result of the entire bowtie analysis process for one main rate channel, LOW-A channel 04 ($\sim80~\text{keV}$) obtained from pixel 04. The green curve is the simulated isotropic pixel response. The 5 black curves are $y_{i}/j_{i}(E_{0})$ for the exponential spectra and the 8 grey curves are the same for the power laws (as in Table 4). The red dot indicates that $E_{0}$ of 80 keV minimizes the spread in $y_{i}/j_{i}(E_{0})$, at a value for $G_{0} \Delta E$ of $3.29 \times 10^{-5}~\text{MeV}\,\text{cm}^{2}\,\text{sr}$, with an RMS spread of 0.5%. A horizontal red bar spanning from 73 to 87 keV indicates the full-width-half-maximum width of the main passband of the channel, which is an independent estimate of $\Delta E$ (here, 14 keV). One can then derive an effective geometric factor, $G_{0}$, of $2.351 \times 10^{-3}~\text{cm}^{2}\,\text{sr}$ for this channel.

**Fig. 15:**
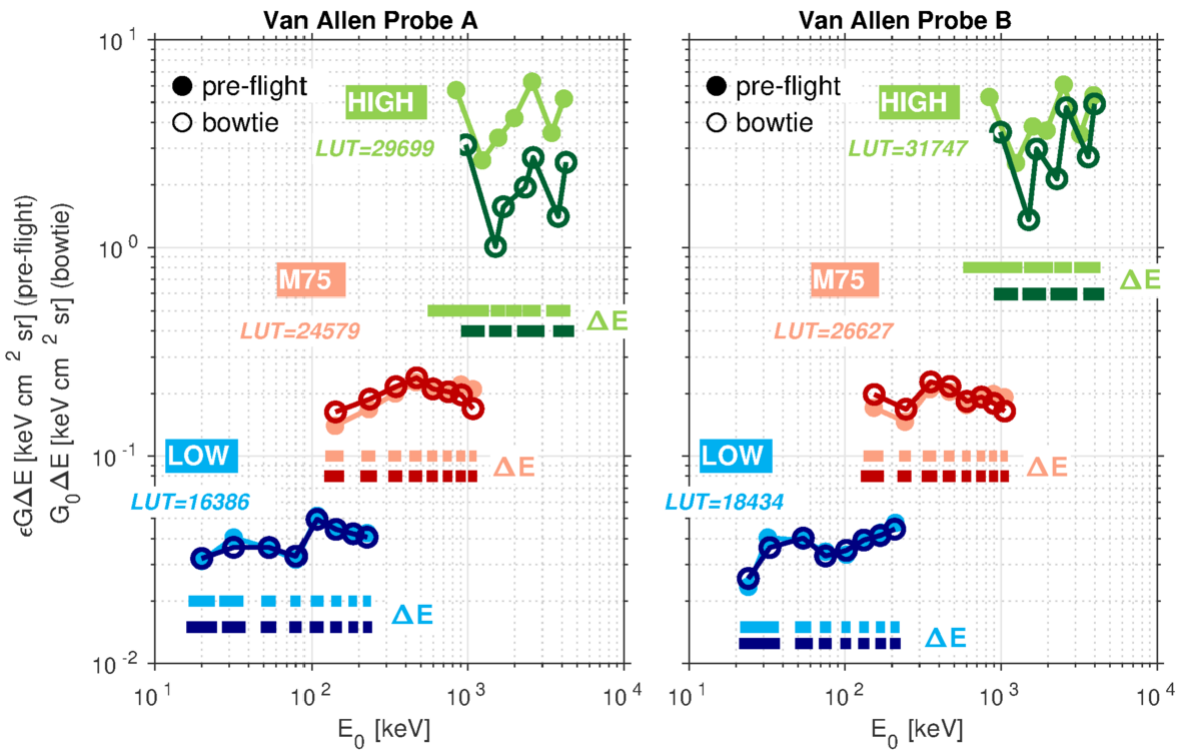
Comparison of the flux conversion factors obtained from pre-flight lab calibrations with the Geant4/bowtie derived values. The values are shown for LOW, M75, and HIGH on both Probes (left and right). The main rate LUT IDs are indicated and the channels widths ($\Delta E$) are shown below the flux conversion factors. For the pre-flight conversion factors ($=\epsilon G \Delta E$), $\epsilon $ is the efficiency (assumed to be unity), $G$ is the geometric factor from Blake et al. (2013), and $\Delta E$ is the channel width obtained in pre-flight calibrations

We perform this same bowtie analysis for all the main rate channels on all 8 electron spectrometers using the spectral parameters shown in Table 4. A separate bowtie analysis is performed for each main rate LUT that was used on orbit, which results in a unique set of bowtie-derived flux conversion factors for each main rate LUT. Since different LUTs were used at different times on the various units, this resulted in a somewhat complicated time-dependence to the flux conversions and energy channel definitions, as described in Sect. 6.6. The bowtie flux conversion factors that were used for the merged data from 03 Aug 2013 until the end of the mission are provided in Table 5 and Table 6, while those derived from earlier LUT combinations are provided in the Electronic Supplementary Material. We emphasize that these bowtie factors are somewhat different from the calibration values obtained in pre-flight lab testing (as reported in Blake et al. 2013), particularly for the HIGH unit, as shown in Fig. 15. The pre-flight calibration factors were phased out in favor of the Geant4/bowtie factors in a previous release of the MagEIS data and are not discussed further in this chapter.

**Table 5:** Energy/Flux Calibration Factors for the Merged Electron Channels on Probe A. Valid from 03 Aug 2013 to 14 Oct 2019. Main rate LUT IDs (LOW/M75/HIGH): 16386/24579/29699

CH #	UNIT-PIX	*E* [keV]	Δ*E* ($E_{lo},E_{hi}$) [keV]	Δ*E*/*E* [%]	$G_{0}\Delta E$ [cm^2^ sr keV]	$G_{0}$ [cm^2^ sr]	$\Delta G_{0}/G_{0}$ [%]
0	LOW-P0	X	X	X	X	X	X
1	LOW-P1	20	9 (16,25)	45	3.210E-02	3.567E-03	1.5
2	LOW-P2	32	11 (27,38)	34	3.630E-02	3.300E-03	1.2
3	LOW-P3	54	12 (48,60)	22	3.625E-02	3.021E-03	0.6
4	LOW-P4	80	14 (73,87)	17	3.291E-02	2.420E-03	0.5
5	LOW-P5	108	22 (97,119)	20	4.953E-02	2.282E-03	0.6
6	M75-P0	X	X	X	X	X	X
7	M75-P1	142	41 (120,161)	29	1.630E-01	3.976E-03	2.2
8	LOW-P6	143	23 (132,155)	16	4.430E-02	1.926E-03	0.5
9	LOW-P7	184	25 (172,197)	14	4.220E-02	1.688E-03	1.3
10	LOW-P8	226	29 (213,242)	13	4.064E-02	1.401E-03	0.8
11	M75-P2	235	55 (205,260)	23	1.880E-01	3.418E-03	1.3
12	M75-P3	346	70 (309,379)	20	2.158E-01	3.083E-03	0.9
13	M75-P4	470	89 (420,509)	19	2.381E-01	2.675E-03	0.9
14	M75-P5	597	94 (545,639)	16	2.104E-01	2.238E-03	0.7
15	M75-P6	749	101 (692,793)	13	2.040E-01	2.020E-03	1.1
16	M75-P7	909	123 (840,963)	14	1.970E-01	1.602E-03	1.3
17	HIGH-P0	970	374 (905,1279)	39	3.120E+00	8.342E-03	6.1
18	M75-P8	1079	135 (1007,1142)	13	1.690E-01	1.252E-03	1.0
19	HIGH-P1	1504	317 (1371,1688)	21	1.010E+00	3.186E-03	2.7
20	HIGH-P1	1688	391 (1504,1895)	23	1.570E+00	4.015E-03	1.7
21	HIGH-P2	2333	422 (2078,2500)	18	1.950E+00	4.621E-03	2.7
22	HIGH-P2	2619	745 (2333,3078)	28	2.710E+00	3.638E-03	2.8
23	HIGH-P3	3790	621 (3536,4157)	16	1.410E+00	2.271E-03	5.3
24	HIGH-P3	4254	897 (3878,4775)	21	2.570E+00	2.865E-03	5.9

**Table 6:** Energy/Flux Calibration Factors for the Merged Electron Channels on Probe B. Valid from 03 Aug 2013 to 16 Jul 2019. Main rate LUT IDs (LOW/M75/HIGH): 18434/26627/31747

CH #	UNIT-PIX	*E* [keV]	Δ*E* ($E_{lo},E_{hi}$) [keV]	Δ*E*/*E* [%]	$G_{0}\Delta E$ [cm^2^ sr keV]	$G_{0}$ [cm^2^ sr]	$\Delta G_{0}/G_{0}$ [%]
0	LOW-P0	X	X	X	X	X	X
1	LOW-P1	24	7 (21,28)	29	2.570E-02	3.671E-03	1.0
2	LOW-P2	33	10 (28,38)	30	3.620E-02	3.620E-03	1.2
3	LOW-P3	54	14 (48,62)	26	4.022E-02	2.873E-03	0.8
4	LOW-P4	75	14 (68,82)	18	3.298E-02	2.425E-03	0.8
5	LOW-P5	102	15 (94,109)	15	3.511E-02	2.341E-03	0.4
6	M75-P0	X	X	X	X	X	X
7	LOW-P6	132	21 (122,143)	16	3.936E-02	1.874E-03	0.4
8	M75-P1	154	50 (127,177)	32	1.980E-01	3.960E-03	2.2
9	LOW-P7	168	25 (157,182)	15	4.140E-02	1.656E-03	1.2
10	LOW-P8	208	31 (195,226)	15	4.442E-02	1.433E-03	0.9
11	M75-P2	246	49 (217,266)	20	1.680E-01	3.429E-03	1.2
12	M75-P3	354	75 (312,387)	21	2.274E-01	3.032E-03	0.9
13	M75-P4	470	85 (424,509)	18	2.164E-01	2.546E-03	0.9
14	M75-P5	604	81 (558,639)	13	1.834E-01	2.264E-03	0.8
15	M75-P6	749	101 (692,793)	13	1.923E-01	1.904E-03	0.9
16	M75-P7	899	112 (840,952)	12	1.793E-01	1.601E-03	0.8
17	HIGH-P0	992	374 (905,1279)	38	3.590E+00	9.599E-03	5.2
18	M75-P8	1054	120 (996,1116)	11	1.646E-01	1.372E-03	0.7
19	HIGH-P1	1504	241 (1371,1612)	16	1.360E+00	5.643E-03	3.3
20	HIGH-P1	1688	400 (1539,1939)	24	2.980E+00	7.450E-03	1.6
21	HIGH-P2	2280	365 (2078,2443)	16	2.140E+00	5.863E-03	1.9
22	HIGH-P2	2619	745 (2333,3078)	28	4.710E+00	6.322E-03	2.1
23	HIGH-P3	3618	502 (3376,3878)	14	2.730E+00	5.438E-03	3.8
24	HIGH-P3	3969	941 (3618,4559)	24	4.910E+00	5.218E-03	4.1

We note that the bowtie analysis does not handle flat spectra very well, which is important to acknowledge since the observed spectrum in the LOW/MED energy range can be flat at times, or even inverted (e.g., Zhao et al. 2019b). The use of falling candidate spectra in the bowtie analysis does not, however, preclude MagEIS from detecting inverted spectra (see, e.g., Fig. 17 below). The bowtie analysis relies on the assumption of smoothness in both the foreground and background signals. However, in MagEIS, each pixel has roughly the same background response, but a different foreground response. Thus, MagEIS can observe inverted spectra since it has many channels that span the energy range of the spectral inversion. We do note, however, that the channel center energies may be inaccurate by a small amount in such cases, since they are shifted to slightly higher energies for inverted spectra, which are not included in the bowtie candidate spectra.

The calibration procedures described above were also used to obtain the flux conversion factors for the high-rate and histogram data. The histogram calibration in particular requires careful modeling of the instrument due to non-ideal sensor response. This, coupled in with background effects and the very narrow histogram channels, means that a simple flux conversion using the $\Delta E$ obtained from the energy deposit/gain conversion and known instrument geometric factor is not sufficiently accurate. Thus, the bowtie analysis is critical in converting the histogram data into calibrated physical units. At the time of writing, the fluxes obtained from the histogram data are a very new data product and have been subjected to only a minimal amount of validation and scrutiny. We refer the reader to the Electronic Supplementary Material for additional details on the conversion of histogram data to fluxes. We note that Fig. 7 shows an example of the fully-calibrated histogram fluxes.

There is one important caveat here for the histogram data that is different from the main rate calculations. Here, we introduce a power-law tail to counteract the nonphysical effects of extrapolating very flat candidate spectral shapes that are reasonable near the low energy threshold to much higher energies that penetrate the shielding off-axis. This is done for $>1~\text{MeV}$ electrons because we determined that the flatter spectra common near $\sim100~\text{keV}$ (e.g., Zhao et al. 2019b) are not appropriately extrapolated to the $>1~\text{MeV}$ regime. The use of a $>1~\text{MeV}$ power-law tail that scales as $E^{-5}$ suppresses the influence of this penetrating response, based on the observation that, for electrons, flat spectra in the vicinity of $\sim100~\text{keV}$ rarely extend to $>1~\text{MeV}$. The imposition of the power-law tail above $>1~\text{MeV}$ for the bowtie analysis de-emphasizes these penetrating particles. In fact, this $E^{-5}$ power-law tail at $>1~\text{MeV}$ is only used in the bowtie analysis for MED unit histogram data and only for pixels 0–6. This is because the HIGH unit analysis already includes this candidate spectrum (Table 4) and the LOW unit simulations stop at 300 keV, so that extrapolation at $>1~\text{MeV}$ does not impact the results. We note that this power-law tail modification is not necessary for the main rate channels, since the wide in-band response for these channels dominates the influence of the higher-energy penetrating response, in contrast to the narrower histogram channels where the effect is more pronounced.

The MagEIS ion telescope response functions were also obtained with Geant4 modeling and a similar bowtie analysis procedure. However, for the front MPA detectors, the proton responses were not significantly different from the pre-flight lab calibrations, so that the bowtie factors were never incorporated into the proton flux data products. Specifically, we found that the bowtie energy channel centroids were nearly identical to the lab calibrations and that the flux conversion factors obtained were different by less than a factor of 2 across all energies. Geant4/bowtie calibration of the MSD thick detector on telescope B and the two thin detectors (2μ, 9μ) on telescope A is ongoing work and will be described in a future publication.

As a final note, we emphasize that we consider the bowtie analysis to be a 1st-order flux conversion and other methods (e.g., multi-channel inverse methods; Park et al. 2021) may provide superior accuracy, especially for complex spectra. We note, however, that multi-channel inverse methods have several shortcomings that have been addressed in the literature (e.g., Sandberg et al. 2012; Cyamukungu et al. 2014). Specifically, the response matrices can be singular and, even once regularized (e.g., via the use of a pseudoinverse), they can produce negative fluxes. On the other hand, the bowtie method is positive definite and does not require the use of multiple channels simultaneously. As noted above, its biggest weakness is that it may not produce accurate fluxes when the true spectrum is very different from the shapes assumed for the candidate spectra. We chose the bowtie method since it has been widely used in the calibration of radiation belt particle measurements (e.g., Van Allen et al. 1974; Selesnick and Blake 2000; Boudouridis et al. 2020), including those from other instruments on the Van Allen Probes (Baker et al. 2021). In line with previous works (e.g., Yando et al. 2011; Morley et al. 2016; Selesnick et al. 2018; Carver et al. 2018), we provide the sensor response functions so that end users can experiment with other calibration methods, should they so choose.

## Data Validation

In this section we present inter-comparisons of the MagEIS electron main channel data during close conjunctions of the two Probes. We find good agreement between Probe-A and Probe-B, and also in the energy-overlap regions between LOW/MED and MED/HIGH.

**Fig. 16:**
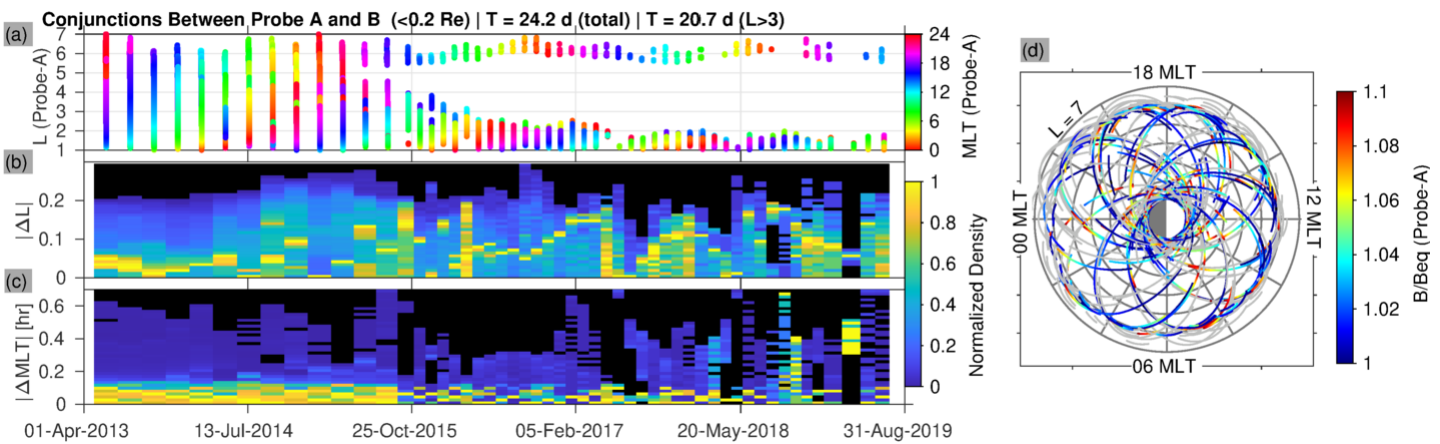
Summary of Van Allen Probe conjunctions when the inter-Probe separation was less than $0.2~R_{E}$. (**a**) The $L$ value of Probe-A during each conjunction, plotted versus time with MLT on the color scale. There were 53 clusters of conjunctions during the time interval displayed that encompassed $\sim24$ d of total observing time. The duration of each conjunction cluster was on the order of a few days. The increase in conjunction-cluster frequency and the decrease in $L$-coverage with time is an orbital effect (see text). (**b**) Histograms of the inter-Probe separations in $L$ during each cluster, normalized by the maximum value in each cluster. (**c**) Same as (**b**), but for MLT. (**d**) The location of Probe-A in $L$ and MLT during each conjunction with $B/B_{eq}$ plotted on the color scale (values greater than 1.1 are shown in grey)

Figure 16 shows a summary of close conjunctions between Probes A and B from 01 Apr 2013 through 01 Sep 2019. Conjunctions were identified using orbital position data at a 1 min time cadence with the criteria that the inter-Probe separation was less than $0.2~R_{E}$. Panel (a) shows all such conjunctions identified, as a function of $L$ and time with MLT on the color scale. The conjunctions occurred in clusters (53 in total during the time interval displayed) due to the orbital phasing of the two Probes, which lapped one another in roughly the same orbit every $\sim60$ days in the early portion of the mission and every $\sim30~\text{days}$ beginning in ∼Oct 2015. The change in frequency from 60 to 30 days was the result of orbital maneuvers that were conducted in the extended mission to increase the lapping rate, which also led to a gradual separation of the orbital “petals” (i.e., the Probes’ MLT at apogee drifted apart with time). Note that while this increased the frequency of the lappings and thus the frequency of conjunctions, the gradual separation of the petals in MLT ensured that conjunctions in the later portion of the mission were restricted to low and high $L$, with a gap in conjunctions between $L\approx 3$ and $L\approx 5.5$ after ∼Oct 2015. Panels (b) and (c) show histograms of the inter-Probe separations in $L$ and MLT, respectively, during each cluster, with the histogram for each cluster normalized to its maximum value. Early in the time interval, $\Delta L$ was typically less than 0.1 in each cluster, and generally less than 0.2 throughout. Similarly, $\Delta\text{MLT}$ was typically less than 0.2 hr. Panel (d) shows the location of Probe A in $L$ and MLT during each conjunction identified, indicating good coverage in both. In what follows, we use this database to compare MagEIS-A with MagEIS-B during close conjunctions.

### MagEIS-A vs. MagEIS-B

**Fig. 17:**
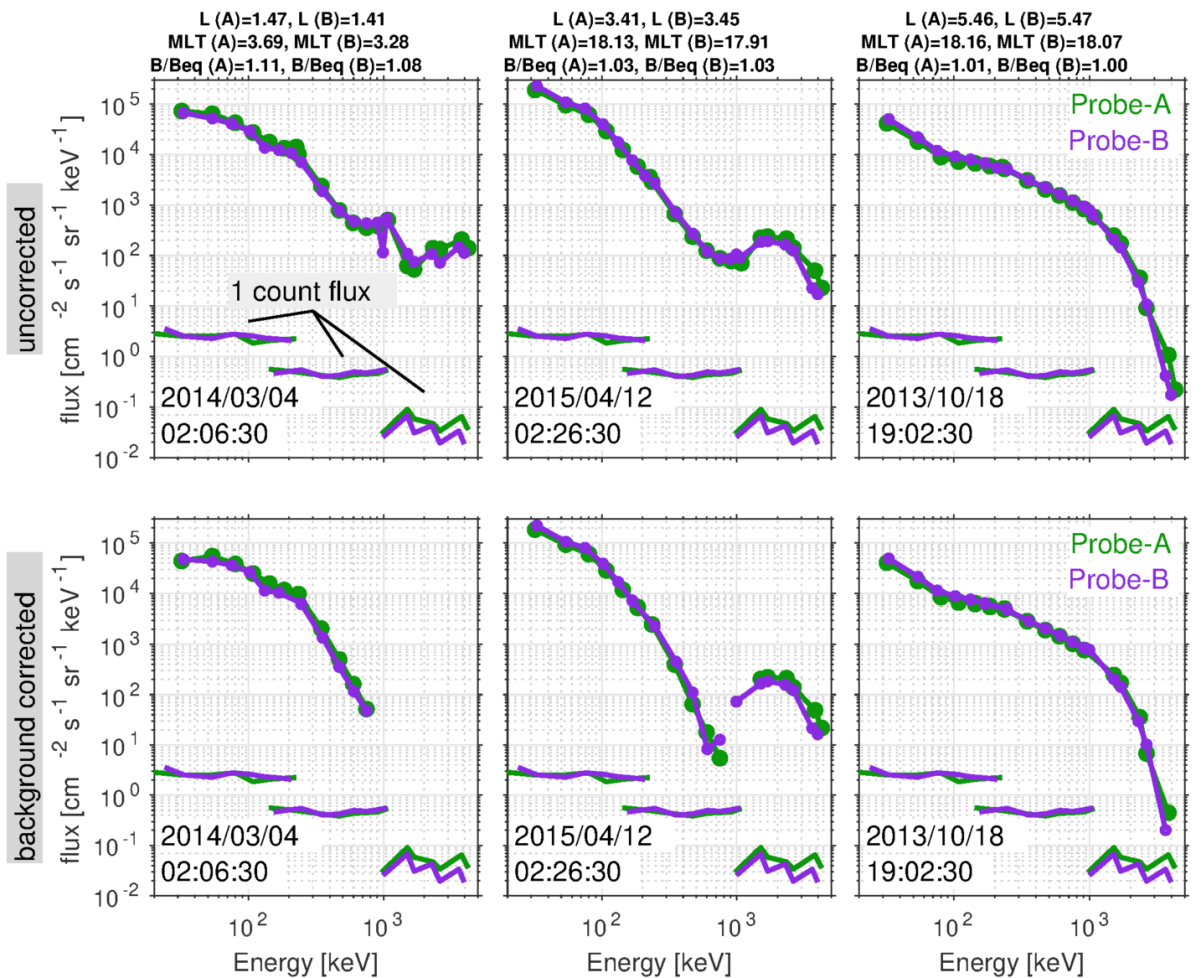
Spectral comparisons between MagEIS-A and -B at three different $L$ values (columns) for uncorrected and background-corrected data (rows). These spin-averaged fluxes were obtained at the indicated conjunction times (the $L$, MLT, and $B/B_{eq}$ values for both Probes are shown at the top). The one count flux levels are shown (the flux level that corresponds to one count per spin); the staircasing is due to the different geometric factors/responses for the three units, LOW, M75, and HIGH

Figure 17 shows spectral comparisons between MagEIS-A and MagEIS-B at three different $L$ values (1.4, 3.4, and 5.5) for both uncorrected and background-corrected flux. These three conjunction intervals were chosen randomly from the conjunction database described above, with the additional requirement that $|\Delta L|<0.1$. We see that the fluxes from Probe-A and Probe-B agree well with one another, generally within a factor of two on these days and for these $L$ values. Note the significant differences between the uncorrected and background corrected fluxes in the inner zone (left column). The flattening and turn up in the uncorrected spectrum at energies $>1~\text{MeV}$ is due to background contamination from the inner proton belt. This contamination is removed in the corrected fluxes, which reveal a steeply falling spectrum in the inner zone. Note also the differences between the uncorrected and corrected fluxes at $L=3.45$ (middle column), where bremsstrahlung x-rays from multi-MeV electrons contaminate the fluxes between $\sim500~\text{keV}$ and $\sim1~\text{MeV}$, leading to a nearly order-of-magnitude correction at 700 keV. We emphasize that the background correction here is more substantial in the M75 channels ($\lesssim 1~\text{MeV}$) relative to the HIGH channels ($\gtrsim 1~\text{MeV}$). This is because the coincidence requirement in the HIGH unit mitigates the effects of background counts, so that a more significant correction is required for the LOW/MED units (Claudepierre et al. 2015, 2019).

**Fig. 18:**
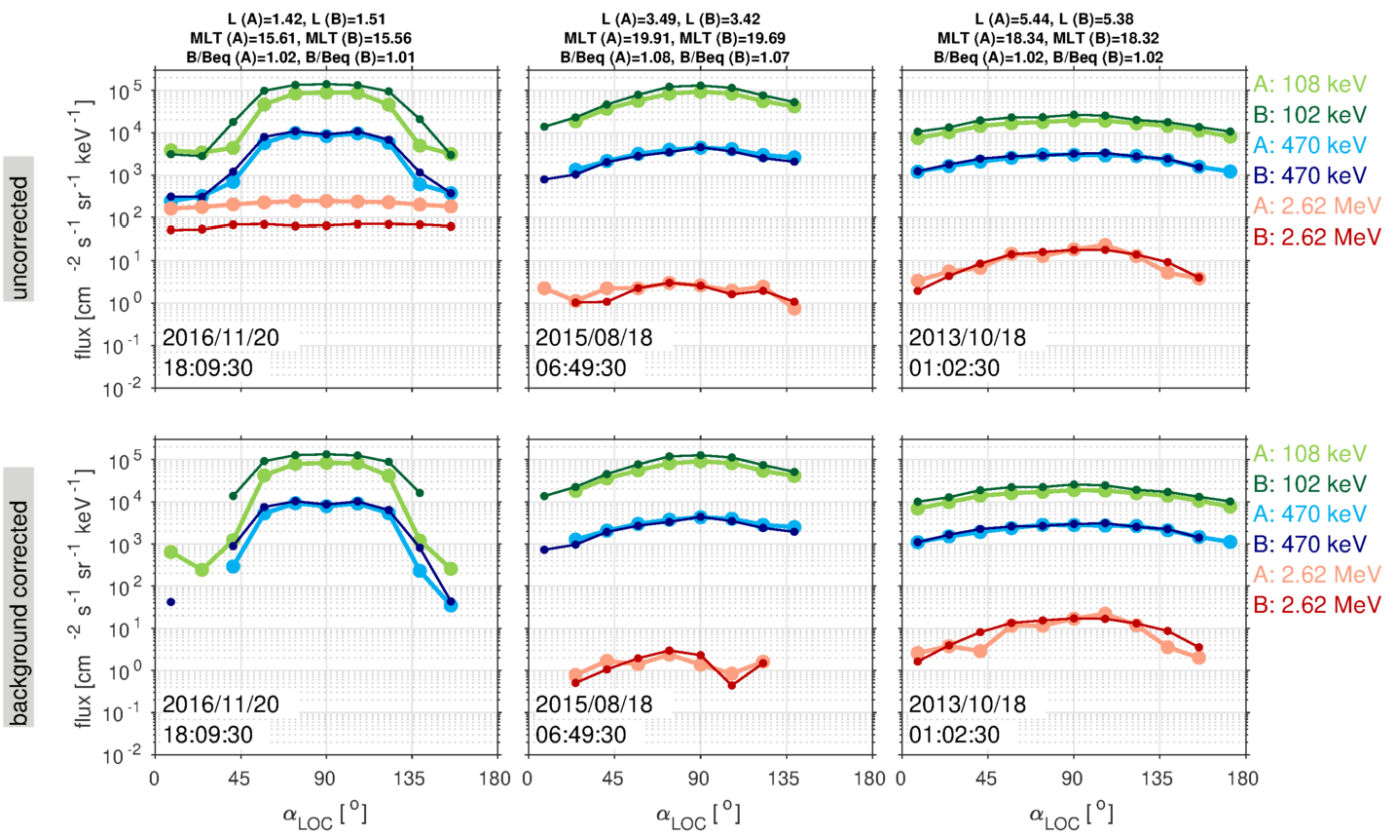
Angular distribution comparisons between MagEIS-A and -B at three different $L$ values (columns) for uncorrected and background-corrected data (rows) during conjunctions. These data are plotted versus local pitch angle for three selected energy channels, one each from LOW, M75, and HIGH. We indicate particles observed moving opposite to the local magnetic field direction with the supplement of their local pitch angles, $\alpha_{loc}>90^{\circ }$

Figure 18 compares the MagEIS angular distributions between the two Probes at the same three $L$ values but during different conjunctions (again with $|\Delta L|<0.1$). One energy channel is selected from each of the LOW, M75, and HIGH units. For this comparison, the $L=5.4$ conjunction was not chosen at random but rather picked specifically to ensure that background corrected data was available from LOW-A for comparison. Again, we see good agreement between Probe-A and Probe-B fluxes, generally within a factor of 2 and with similar shapes. Note that the angular distributions in the inner zone at $\sim100~\text{keV}$ and $\sim500~\text{keV}$ are left largely unchanged by the correction algorithm near $90^{\circ }$ local pitch angle, while the fluxes at pitch angles closer to the loss cone are corrected by a significant amount (more than an order of magnitude). At these pitch angles, the uncorrected angular distributions flatten, suggesting large uncertainties (i.e., low foreground rates in the presence of high background levels).

**Fig. 19:**
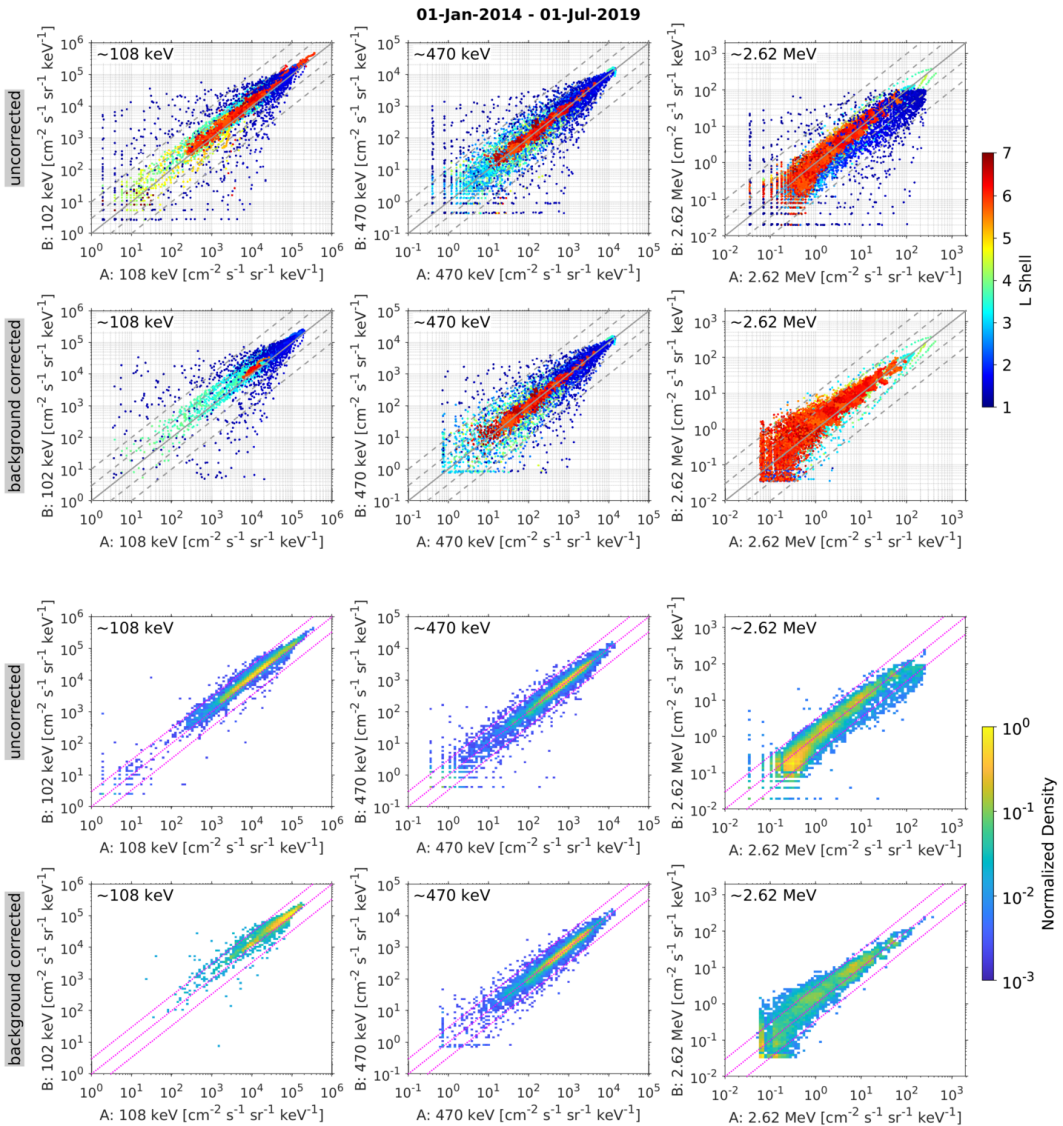
Scatter plot comparisons between MagEIS-A and -B at three selected energy channels from LOW, M75, and HIGH (columns) for uncorrected and background-corrected data (rows). These spin-averaged fluxes are shown for all conjunctions identified between 01 Jan 2014 and 01 Jul 2019. In the upper set of 6 panels, each point is color-coded by $L$ value and guidelines are shown indicating factors of 1, 3, and 10 difference. In the lower set of 6 panels, a 2D, normalized point-density is shown using a different color map, with only the factors of 1 and 3 guidelines shown (in magenta). The point-density is normalized by the maximum value in the domain and values that correspond to $\leq1$ point per bin are omitted

Figure 19 provides a more comprehensive comparison between Probe-A and Probe-B at the same three energy channels that were used in the angular distribution comparisons. These scatter plots show data from all conjunctions identified between 01 Jan 2014 and 01 Jul 2019 with no additional restriction on $|\Delta L|$. We see a good linear relationship between the flux levels observed on both Probes, with the differences generally lying within the factor of 3 guidelines, most clearly seen in the “normalized density” panels. In the top row at $\sim2.62~\text{MeV}$, a region of disagreement is noted as a cluster of blue points near the factor of 1/3 line. This is merely reflective of the different responses of the two HIGH units to inner belt proton contamination (e.g., compare with the corresponding panel in the second row where the disagreement is not seen because those points have been removed via background correction). It is also clear that the background corrections introduce some error into the flux levels, with more scatter relative to the uncorrected data, especially at the lower flux levels. This is to be expected when foreground signals are low relative to background levels, due to the uncertainties introduced when removing background contamination (see Claudepierre et al. 2015).

To summarize, we find that the discrepancies between Probe-A and Probe-B are generally within a factor of 2–3. We have performed these analyses at other energies and find similarly good agreement, and we do not find any systematic $L$, angular, or temporal dependence to these small offsets between MagEIS-A and MagEIS-B (not shown here).

### Energy Overlap Between LOW/MED and MED/HIGH

**Fig. 20:**
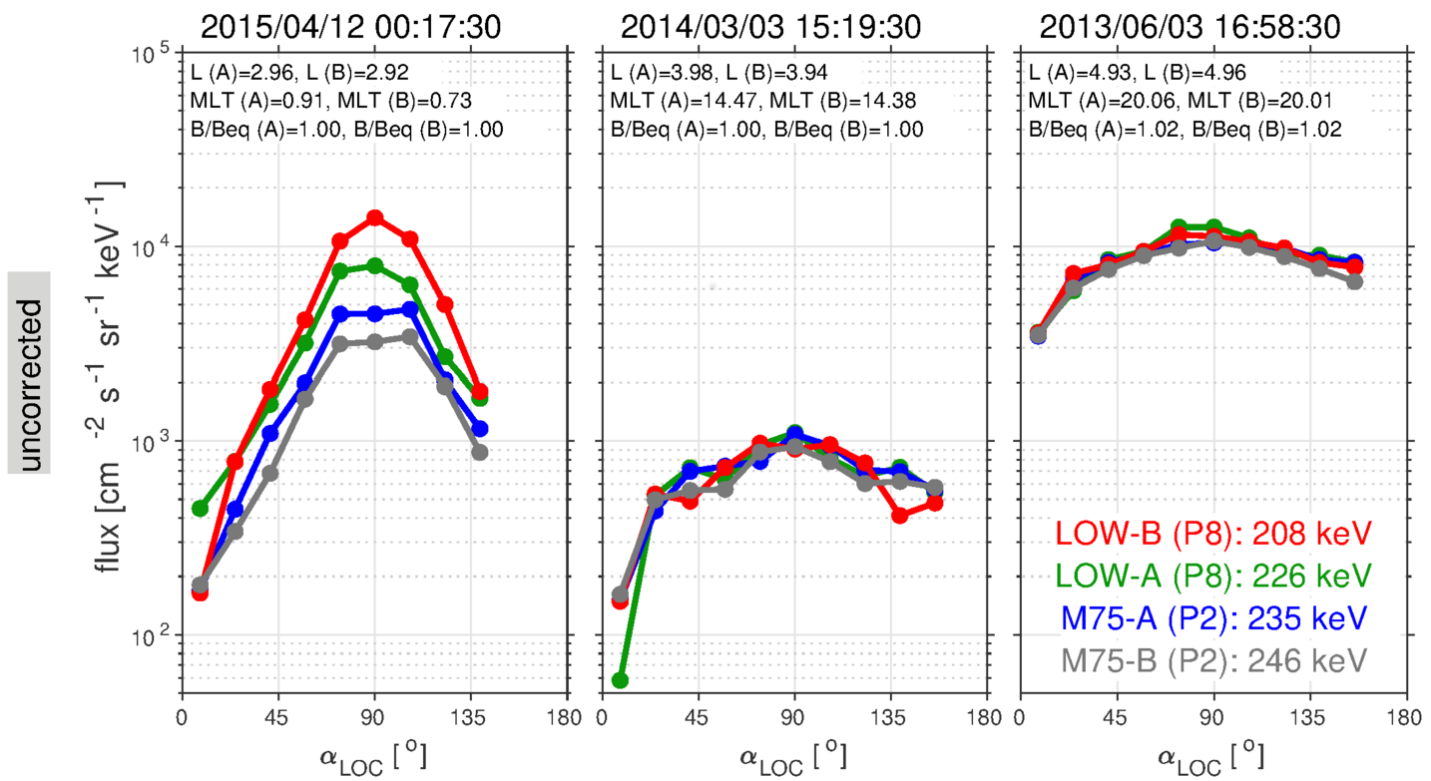
Angular distribution comparisons between LOW and M75 on both Probes at three different $L$ values (columns) during conjunctions. The four channels that span the 208–246 keV energy range are plotted (two channels from each Probe) versus local pitch angle and only uncorrected data are shown

We now consider the four energy channels that span the 208–246 keV energy range in the overlap region between LOW and M75. Note that the spectral comparisons presented above in Fig. 17 suggest that these channels agree well with one another, for the $L$ and conjunction times selected in that figure. Figure 20 shows local pitch angle distributions for these four channels at three different $L$ values. Again, these three conjunction intervals were chosen at random but subject to the additional requirement that $|\Delta L|<0.1$. Note that only uncorrected data are shown in these comparisons, as background corrections are not possible on LOW P8 (see Sect. 3.2.4). At the two higher $L$ values shown in the figure, we see that the distributions all agree well with one another, within a factor of $\sim2$ across all pitch angles. However, at the lowest $L$ value, larger differences are seen. We attribute this to a steeply falling spectrum rather than an intercalibration issue between the units, since the differences are ordered by energy and the agreement is good at higher $L$ where spectra are generally flatter.

**Fig. 21:**
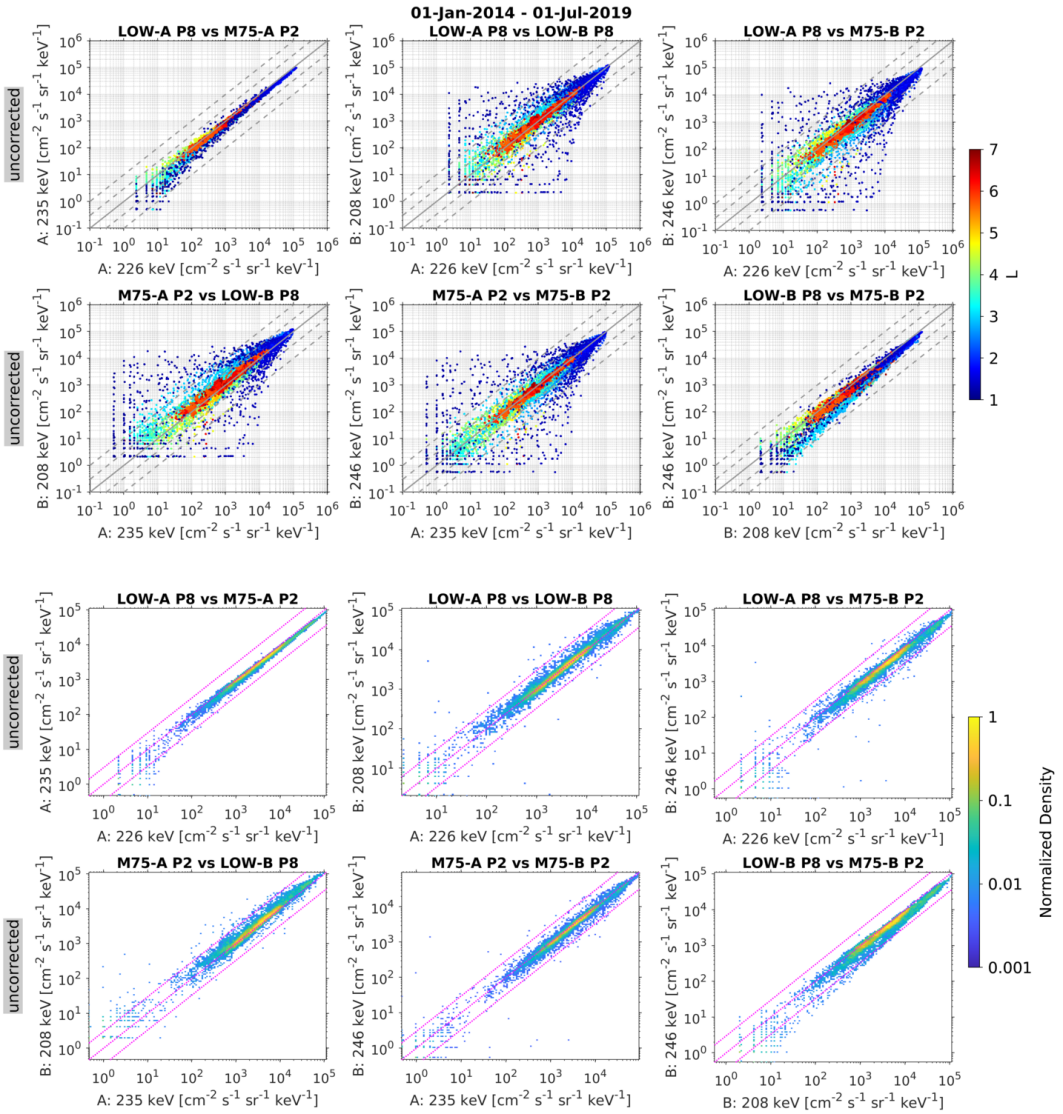
Scatter plot comparisons between LOW and M75 on both Probes in the same format as Fig. 19. The six different combinations of the four channels that span the 208–246 keV energy range are shown (two channels from each Probe). These spin-averaged fluxes are shown for all conjunctions identified between 01 Jan 2014 and 01 Jul 2019 and only uncorrected data are shown

Figure 21 provides scatter plot comparisons for these same four LOW/M75 channels, similar to the scatter plots presented above. Here, the six different permutations of the 4 energy channels are shown. Again, we see very good agreement between the channels, generally within a factor of $\sim2\text{--}3$, and the tightest agreement between the channel pairs on the same spacecraft. The scatter between channels pairs on different spacecraft can be reduced by placing additional criteria on the conjunctions (e.g., $|\Delta L|<0.1$ or proximity to the magnetic equator; not shown here). Note that the instances where the disagreement is worse than a factor 3 are typically at $L<4$, again suggesting that spectral shape may contribute to the differences. Background contamination from bremsstrahlung x-rays may also contribute to the differences here, since this is typically where multi-MeV electrons are the most intense and each unit/pixel responds differently to background contamination. In this regard, we note that the M75 P2 channels are physically wider and wider in energy response relative to the LOW P8 channels, and their energy-angle response factors are larger by a factor of $\sim4$ (see Table 5 and Table 6).

**Fig. 22:**
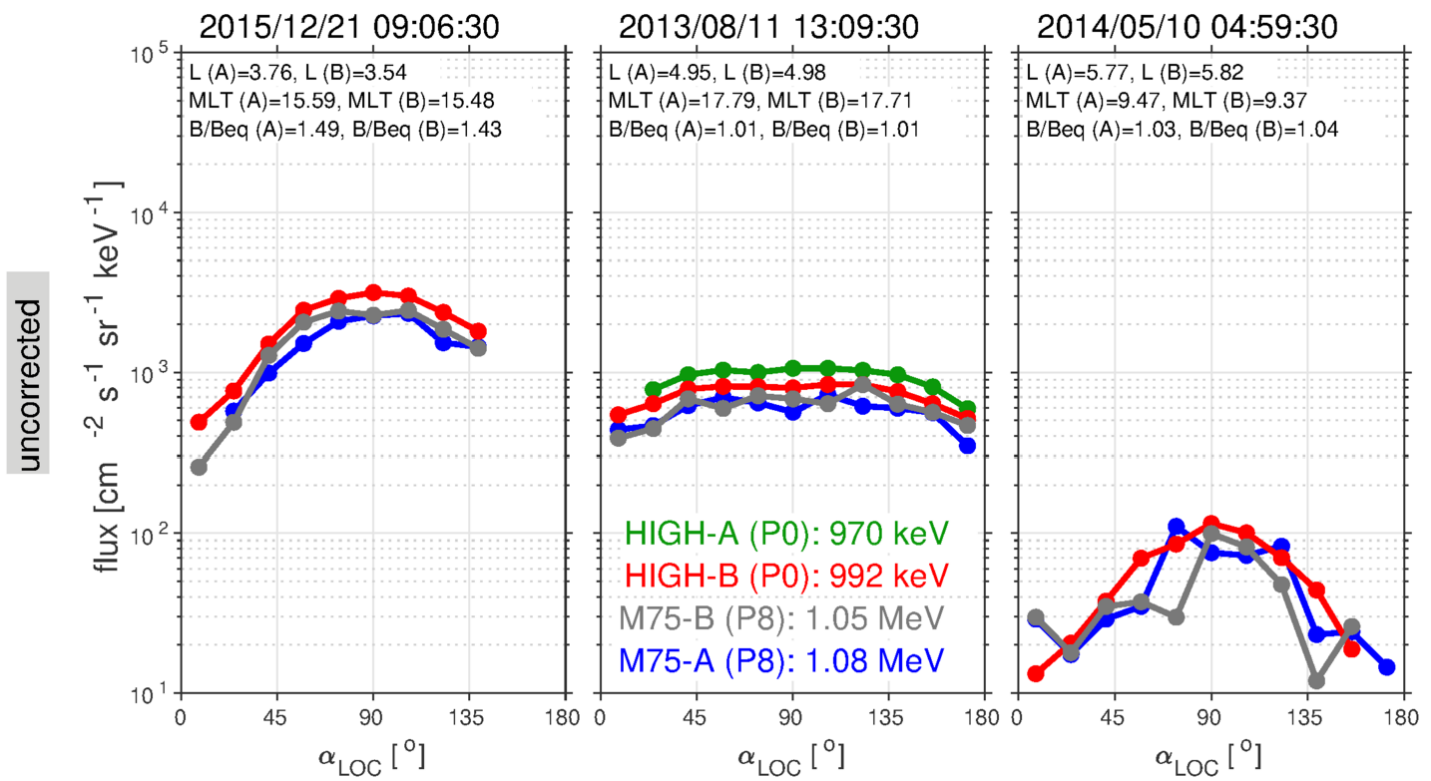
Angular distribution comparisons between M75 and HIGH on both Probes at three different $L$ values (columns) during conjunctions. The four channels that span the 970–1079 keV energy range are plotted (two channels from each Probe) versus local pitch angle and only uncorrected data are shown. HIGH-A P0 failed in Oct 2013 so that only the middle panel contains these data

We now turn our attention to the overlap region between M75 and HIGH, which corresponds to the 970–1079 keV energy range. Figure 22 shows angular distributions for these four channels in a similar format to what was used above for the LOW/MED comparisons. Again, only uncorrected data are used in these comparisons since background corrections are not possible on M75-P8. Here, we find that the distributions are generally within a factor of $\sim3$ of one another with similar shapes, suggesting a good intercalibration. However, we note that the discrepancies are similar at all three $L$ values shown and that the entire energy range considered is narrow, indicating that there may be a more systematic offset between M75-P8 and HIGH-P0, i.e., one that is not entirely related to spectral steepness. We note that the conjunction times for these M75/HIGH comparisons were not chosen at random for a number of reasons. The comparison at $L=5$ (middle) was specifically chosen to show a conjunction that occurred before HIGH-A P0 failed in Oct 2013. In addition, 1 MeV fluxes can often be quite low, so we searched for conjunction times during which the fluxes were high at $L\approx 3.6$ and loosened the restriction on $|\Delta L|$ from 0.1 to 0.3 in this case.

**Fig. 23:**
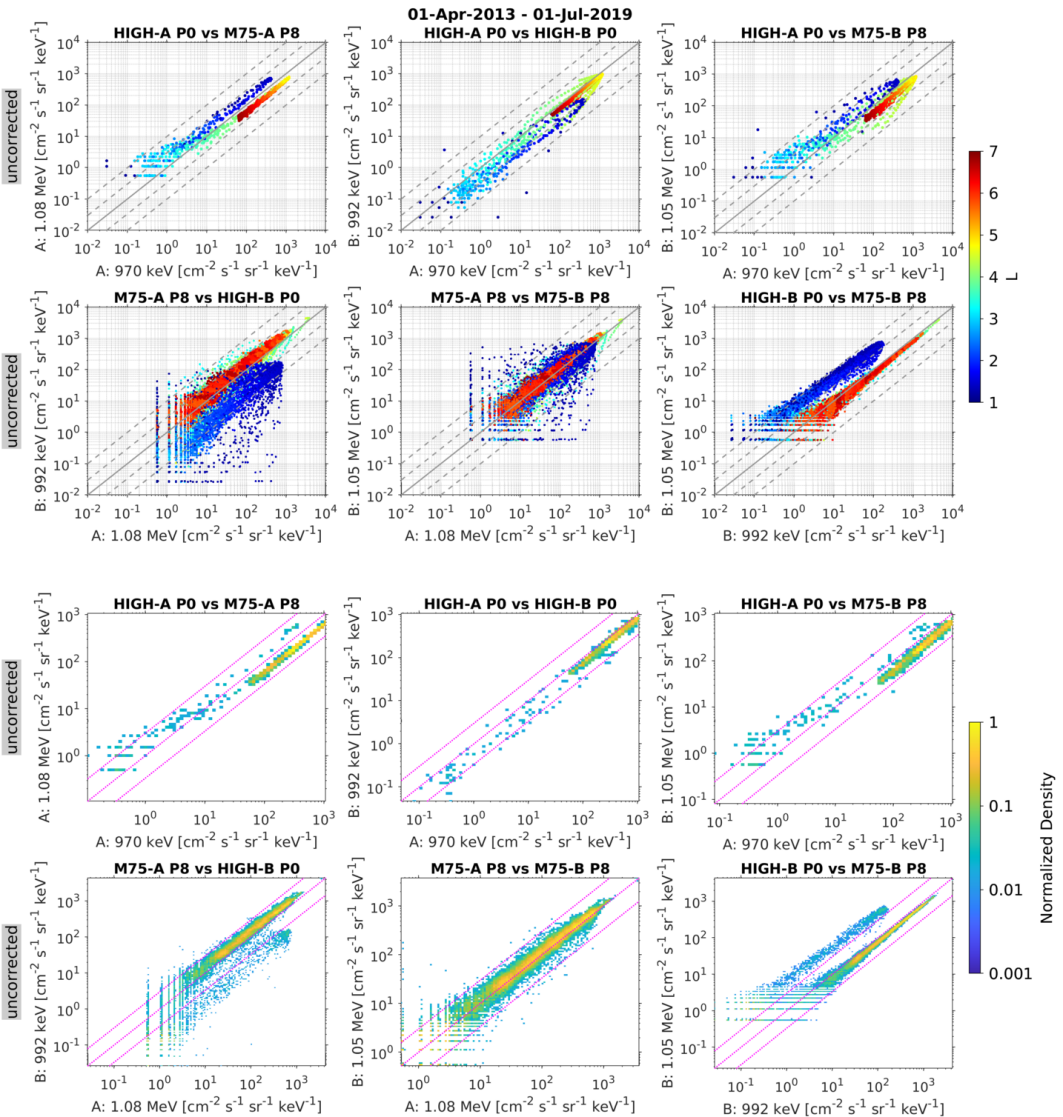
Same as Fig. 21 for the four M75 and HIGH channels that span the 970–1079 keV energy range. We find a systematic intercalibration offset of a factor $\sim2\text{--}3$ between M75-P8 and HIGH-P0. The significant offsets in the inner zone (blue hues in the upper 6 panels) should be ignored as they are largely due to background contamination at these energies. Note that HIGH-A P0 failed in Oct 2013, so that there is considerably less data in the panels that compare this pixel (top row in each set of 6 panels)

Figure 23 shows scatter plot comparisons for the six permutations of the same four M75/HIGH channels that span the 970–1079 keV energy range. As these comparisons are only possible for the uncorrected data, the out of family points in the inner zone (blue hues in the 6 upper panels) should be ignored as they are largely due to background contamination at these energies. We also emphasize that the comparisons in the top row show considerably less data than those in the second row, due to the failure of HIGH-A P0 in Oct 2013. In Fig. 23, we see that there is a systematic intercalibration offset of a factor $\sim2$ between M75-P8 and HIGH-P0, most clearly seen in the “normalized density” panels. There does not appear to be an $L$ dependence to these differences, which suggests that it is not related to spectral shape as was the case for the LOW/MED units. In this regard, we note that the instrument responses of M75-P8 and HIGH-P0 are different due to a number of factors. For example, the HIGH unit uses coincidence between front and rear detectors, while M75 does not have a coincidence system. Moreover, the response factors for HIGH-P0 are larger than M75-P8 by a factor of $\sim20$ (see Table 5 and Table 6): HIGH-P0 responds to a wider energy range of electrons (905–1279 keV) relative to M75-P8 (1007–1142 keV) with a much larger effective geometric factor ($\sim0.01$ vs. $\sim0.001~\text{cm}^{2}\,\text{sr}$).

In summary, the MagEIS electron measurements agree well with one another when compared between the two Probes and when compared in the overlap energy range between two units. Cross-calibration work at the ECT suite level shows good intercalibration between HOPE and MagEIS near 30–50 keV and between MagEIS and REPT near 2–4 MeV (Boyd et al. 2019; Reeves et al. this book). We also note that the MagEIS electron measurements agree well with the energetic particle measurements from the ERG/Arase mission (Szabo-Roberts et al. 2021; Miyoshi et al. this book) and the Cluster mission (Smirnov et al. 2019).

## Data Caveats

This section briefly describes a number of important caveats that impact the use and interpretation of MagEIS data. Many of these points are expanded upon in the Electronic Supplementary Material.

### Noise in LOW/MED Pixels 0 and 1

**Fig. 24:**
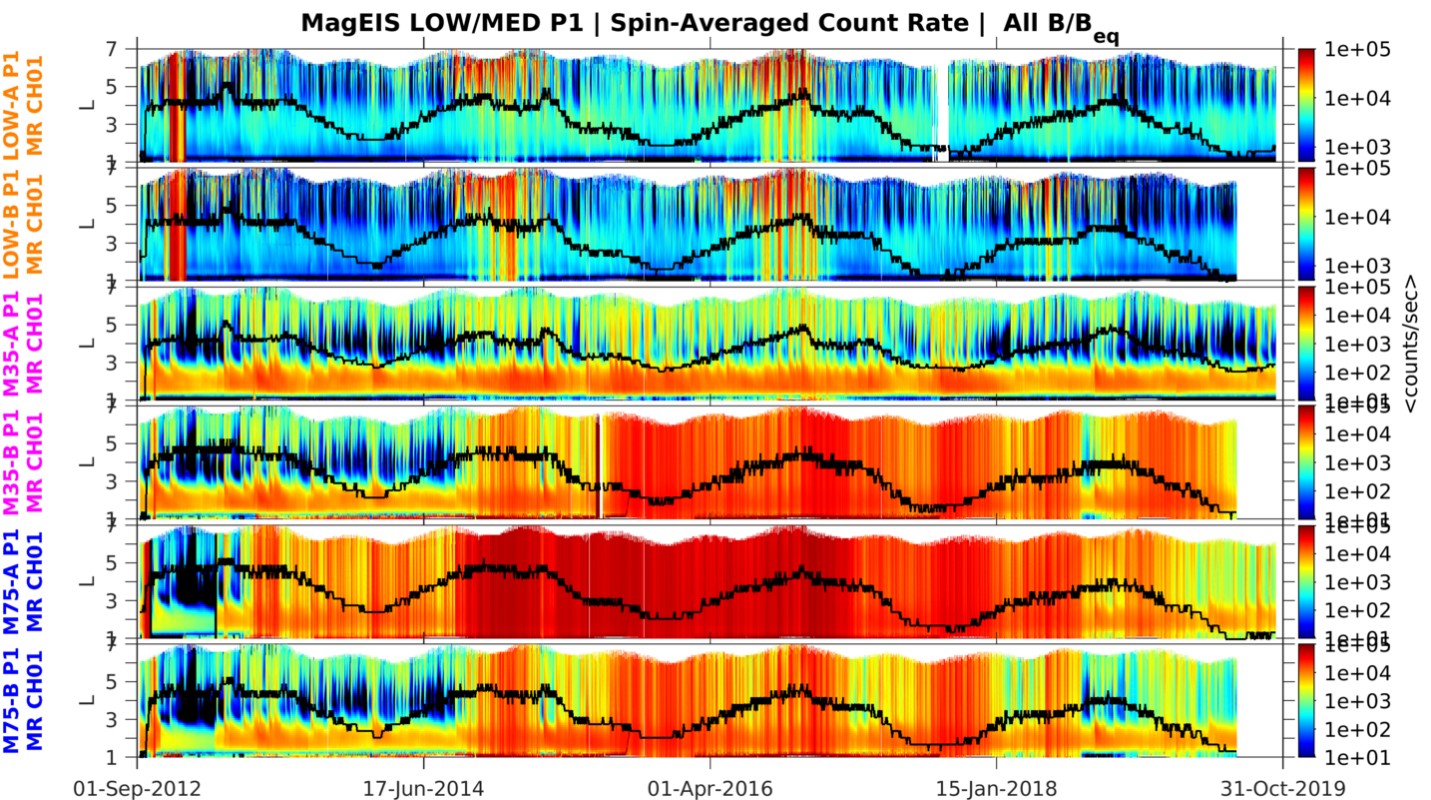
Noise in LOW/MED P1. The raw, spin-averaged count rates are daily-averaged and binned in $L$. The daily maximum yoke temperature is overplotted in black, with the units arbitrarily scaled to the $L$ range of the plot window (the peak-to-peak range is $\sim5~^{\circ }\text{C}$)

Pixel 0 and pixel 1 on the six LOW/MED units were known to be noisy, which manifested as very high count rates in these two pixels, at or near saturation levels. Figure 24 shows $L$-sorted plots of P1 data from all six LOW/MED units; an analogous plot for P0 data is shown in the Electronic Supplementary Material. (We note that P0 was partially shielded and was intended to serve as a background monitor, so that it did not provide scientifically useful electron fluxes irrespective of any noise considerations.) All four MED units displayed noise characteristics in P0 and P1 almost immediately after turn on and became swamped with noise during the 08 Oct 2012 storm, though these early effects are not discernible on the time scale used in the figure. The noise subsided for a time and then returned again in all of the units except M35-A, which appears to have relatively noise free P1 data throughout the mission. We note that P0 on M35-A did display some noise signatures, in contrast to P1. P0 and P1 on the LOW units also displayed noise characteristics early on and became totally saturated with noise following the 14 Nov 2012 storm. They remained saturated until the biases were lowered in late December 2012, an effect which is clearly seen in the figure. The noise in the LOW units returned (seen as a saturation in rates across all $L$ shells) at later times, though not to the sustained levels observed in the MED units.

While the P0/P1 noise was believed to be primarily due to leakage current and enhanced during high flux intervals (see Sect. 8.1), the noise also appeared to be influenced by the ambient yoke temperature, which is overplotted in each panel in Fig. 24. We note that the timing of the four local maxima in the yoke temperatures (period $\sim18$ months) was related to the Probes’ orbital motion, occurring when the Probes had their apogees at $\sim00$ MLT. In addition to the long-term relationship with temperature, when the P0 and P1 detectors became noisy, they displayed periodic oscillations in the count rates at the thermal control period ($\sim15~\text{min}$ – see Fig. 3). This further indicates a sensitivity to temperature.

All data from LOW/MED pixel 0 and pixel 1 have been set to fill values in the merged level 2 and level 3 data files. Thus, end users of these higher-level data products should not see any of the noise and thermal oscillations signatures. However, we emphasize that fluxes from LOW/MED P1 have been retained in the unit-by-unit level 2 and level 3 files, as there are times when these data may be suitable for scientific analysis, as can be seen in portions of Fig. 24. The end user should nevertheless exercise an abundance of caution when using LOW/MED P1 data.

### Thermal Oscillation Noise in HIGH Electron Data

When the yoke temperature on the HIGH electron spectrometer got too low, the count rates would drop out and were not valid. The reason for this is not fully understood, though it appears that the individual detector thresholds varied with temperature. It also appears to have affected only some, but not all, of the HIGH unit detectors, which was likely related to the different threshold settings on the individual detectors. Due to changes in the ambient thermal environment in various portions of the Van Allen Probes orbit over the course of the mission, there have been time intervals when the yoke temperature dropped below the nominal range. This led to instances of this thermal oscillation noise, which manifested as brief ($\sim5$ minute) flux dropouts recurring at the thermal control period ($\sim60$ minutes – see Fig. 3). The effect was mitigated by periodically changing the heater set point that was used to initiate the thermal control system (see Fig. 3). There were two long time intervals in 2014 and additional shorter intervals throughout the mission (detailed in the Electronic Supplementary Material) where this effect was particularly problematic (especially for pixel 1 on HIGH-A) and was not correctable. The fluxes from these time intervals have been set to fill values for the affected pixels.

### Data Quality Issues Before January 2013

The MagEIS units were powered on both Probes on 06 Sep 2012 during the first week of instrument commissioning. Science data were generally available from this time, but due to the large number of configuration changes early in the mission and uncertainties regarding absolute calibrations, electron measurements obtained prior to ∼Jan 2013 were of reduced quality. After the bias adjustments were made on the LOW/MED units in mid-December 2012 (see Sect. 6.5), these data were of much better quality and their intercalibrations were improved. The HIGH unit underwent further reconfiguration until ∼Sep 2013 (see next section) and its data should be used only qualitatively prior to this time. Thus, the MagEIS-REPT electron cross-calibrations were poor during this early period (pre Sep 2013).

### Major Changes in HIGH Electron Spectrometer Logic

Extensive cross-calibration work by the MagEIS and REPT teams led to a major reconfiguration of onboard logic in the MagEIS HIGH electron units. These changes occurred between 03 Jul 2013 and 26 Sep 2013, with the specific dates given in the Electronic Supplementary Material. MagEIS HIGH electron data acquired prior to the reconfiguration (before 03 Jul 2013) should be used only for qualitative purposes. Data acquired between 03 Jul 2013 and 26 Sep 2013 should be used with an abundance of caution since extensive tuning was done during this time interval. Any step-function changes in flux or other peculiarities on these days were likely instrumental (non-physical) effects. These instrumental tunings led to a significant improvement in the MagEIS/REPT cross-calibration. In addition, we note that a variety of instrument tests were performed in late May and early June of 2014 on the MagEIS HIGH electron units. These were simply instrument diagnostics and did not improve the quality of the data like the major changes in Jul-Sep 2013. However, there are some data gaps between May and November 2014 due to these diagnostic tests and related commanding (see Electronic Supplementary Material).

### Detector Bias

The electron spectrometers were occasionally operated in the “bias-off” configuration for varying periods of time, which corresponded to the low-bias state of $\sim20\text{--}40~\text{V}$ (see Sect. 1.4). When the bias was off, the flux intensities were affected, but the fluxes have not been set to fill values in the data files during these times, as the data can still be used for qualitative purposes. Nearly all of these instances occurred early on in the mission (see Fig. 2), but the end user should be aware of this and, if there is concern, should check the bias state, which is contained in a status variable in the level 1 and level 2 unit-by-unit files. The bias voltages from the instrument housekeeping data are available in the level 1 files and a listing of specific time intervals in the low-bias state is provided in the Electronic Supplementary Material.

### Time-Varying Energy Channels and Sampling Parameters (Sectoring and Accumulation Times)

One of the challenges that comes with having an instrument that is highly configurable in-flight is that parameter changes and fine tunings can have a negative impact on the ease of usability of the scientific data. To this end, the end user should be aware of the time dependencies of both the energy channel definitions and the sampling parameters, and of the influence that they can have on the measurements, especially when analyzing long time-intervals of data (e.g., months to years). For example, the normal science mode data were output at the highest temporal and angular resolutions beginning on 27 Jun 2014 and at coarser resolution prior to this time, with frequent changes (see Sect. 3.3). Moreover, when a new MR LUT was uploaded to a given MagEIS unit, this changed the energy centroids, energy widths, and flux conversions factors for this unit. Thus, there was not a fixed set of energy channels for MagEIS for the duration of the mission. Moreover, LUT uploads were done at different times on different units, which resulted in somewhat complicated time dependence of the flux conversions and energy channel definitions for the merged data product. We emphasize that within a given daily CDF file, there is a fixed set of (non-time-varying) energy channels. The energy channels may change from day to day when new LUTs were uploaded, but for a given day the channel definitions are fixed. Also, after 04 Oct 2012, all MR LUTs were changed at UTC day boundaries. The final revisions to the MR LUTs, energy channel definitions, and flux conversion factors were not made until ∼Aug 2013, after which time these values remained fixed for the duration of the mission. The dates of all of the LUT changes and the associated calibration factors are provided in the Electronic Supplementary Material.

### Noise and Efficiency-of-Detection Issues in the Proton Channels

The front MPA detector in the ion telescopes on both Probes began to display noise characteristics early in the mission in March 2013. A low level of noise was observed in the lowest-energy channels on both spacecraft (channels 00–02, $\sim60\text{--}80~\text{keV}$ protons) and this noise increased over time in a very similar manner on both telescopes. A detailed analysis revealed that the noise first appeared in channel 00, then progressed into channel 01, and subsequently into channel 02 and worsened with time from mid-March 2013 onward. By October 2014, the noise had progressed into the first six (00–05) proton channels, at which time the proton thresholds were raised to suppress these noisy lowest-energy channels. These first six proton channels ($\sim60\text{--}140~\text{keV}$) are set to fill values after 15 Oct 2014. It is believed that the noise was due to radiation damage (ion implantation) on the surface of the front detector (see Sect. 8.2).

In addition to the noise due to ion implantation damage, the higher-energy channels ($\sim 700\text{--}1200~\text{keV}$) from the same MPA detectors exhibited a decrease in detection efficiency. We believe that this may have been related to the ion implantation damage. The decrease in detection efficiency progressed over the entire mission on both spacecraft, where a slow steady decrease in proton intensity was observed in the higher-energy channels. We do not believe that this slow decrease in flux was a real geophysical effect.

Figures are provided in the Electronic Supplementary Material that summarize the noise and detection-efficiency signatures in the MagEIS proton data from the MPA detectors. In general, the proton data from the MPA detectors should only be used qualitatively and with extreme care, and only until mid-2013. We recommend using RBSPICE proton measurements instead, which nominally cover the same energy range ($\sim60~\text{keV}\text{--}1~\text{MeV}$).

### Light Contamination in the Ion Telescopes

When the ion telescopes viewed the sunlit Earth, the front detectors saturated and the fluxes would drop to zero. This would typically occur at low $L$ (e.g., $L<2.5$) and only over a portion of a spacecraft rotation. As noted above, the level 2 to level 3 processing corrects for this issue by masking and removing these light-contaminated portions of the spin/orbit. While this contamination has been masked out in the level 3 proton data, it remains in the level 2 data and leads to an absence of valid data over a significant portion of the angular distribution when it occurs. End users should be aware of this when looking at angular-resolved (and spin-averaged) proton data. We emphasize that only the front MPA detectors in the ion telescopes suffered from this light contamination. The rear MSD detector on Probe-B was shielded by the inert 10 μm LP detector, and the 2μ and 9μ detectors on Probe-A had high thresholds. Further details on the light contamination signatures are provided in the Electronic Supplementary Material.

### Background Contamination and Correction Algorithm

Electron measurements from the MagEIS suite have been corrected for background when possible (see Sect. 3.2.4), using the histogram data and the automated routine of Claudepierre et al. (2015). Background arises from several sources, both external and internal to the instruments. Penetrating radiation that reaches the focal planes of the instruments will cause an “event” in the electronics system. This penetrating radiation includes galactic cosmic rays, energetic solar particles, inner zone protons, and bremsstrahlung from the interaction of energetic electrons with the spacecraft. Internal background results from electron backscatter from the silicon focal plane and other scattering events within the magnetic spectrometer itself.

The level of instrumental background depends strongly upon several factors, including the intensity of the energetic particles in the radiation belts at a given time and the location of the Probes within the radiation belts. A good rule-of-thumb is that above about 1 MeV, electron data in the inner zone is highly suspect and likely predominantly contaminated by high-energy protons. However, background influences all of the MagEIS data at various locations along the orbit. In particular, bremsstrahlung appears to be a major source of contamination in the LOW/MED units, at energies $\sim30\text{--}700~\text{keV}$, in regions of space where intense multi-MeV electrons are present. It is important to be aware of the complexity of the background removal process and that the MagEIS data can never be used blindly. We strongly encourage the end user to consult Claudepierre et al. (2015) before using background-corrected MagEIS data and to be aware of the various caveats therein. The background-corrected variables in the level 2 and level 3 data files are described further in the Electronic Supplementary Material. Note that the MagEIS ion telescopes provide a single parameter measurement that cannot be corrected for background.

### Eclipse Effects

MagEIS data should always be handled with care when the spacecraft enters, exits, and is in eclipse (i.e., it is in the Earth’s shadow), as the angular accumulation of particle counts over the spacecraft spin is prone to timing errors during eclipse. For example, in normal operations, the instrument receives a “sun pulse” from the spacecraft indicating the beginning of a new spacecraft spin. However, in eclipse, that timing indicator is no longer available, so the instrument uses an internally generated timing based on a hardware timer set at eclipse entry. Moreover, during eclipse, the spin rate of the spacecraft increases due to changes in the thermal environment and the conservation of angular momentum. These effects conspire to produce a mismatch between the instrument accumulation time (e.g., 1 spin) and the true spacecraft spin period. In geophysical terms, this means that the mapping between particle pitch angle and instrument sector number (spin-phase angle) changes much more dramatically than during normal, non-eclipse times. When in eclipse, the fluxes can appear to change rapidly and periodically at very low pitch angles; flux spikes/dips are produced at eclipse exit. These effects are illustrated in the Electronic Supplementary Material. All MagEIS units, including the ion telescopes, are subject to these effects.

### Rate Saturation in LOW Pixels 2 and 3

**Fig. 25:**
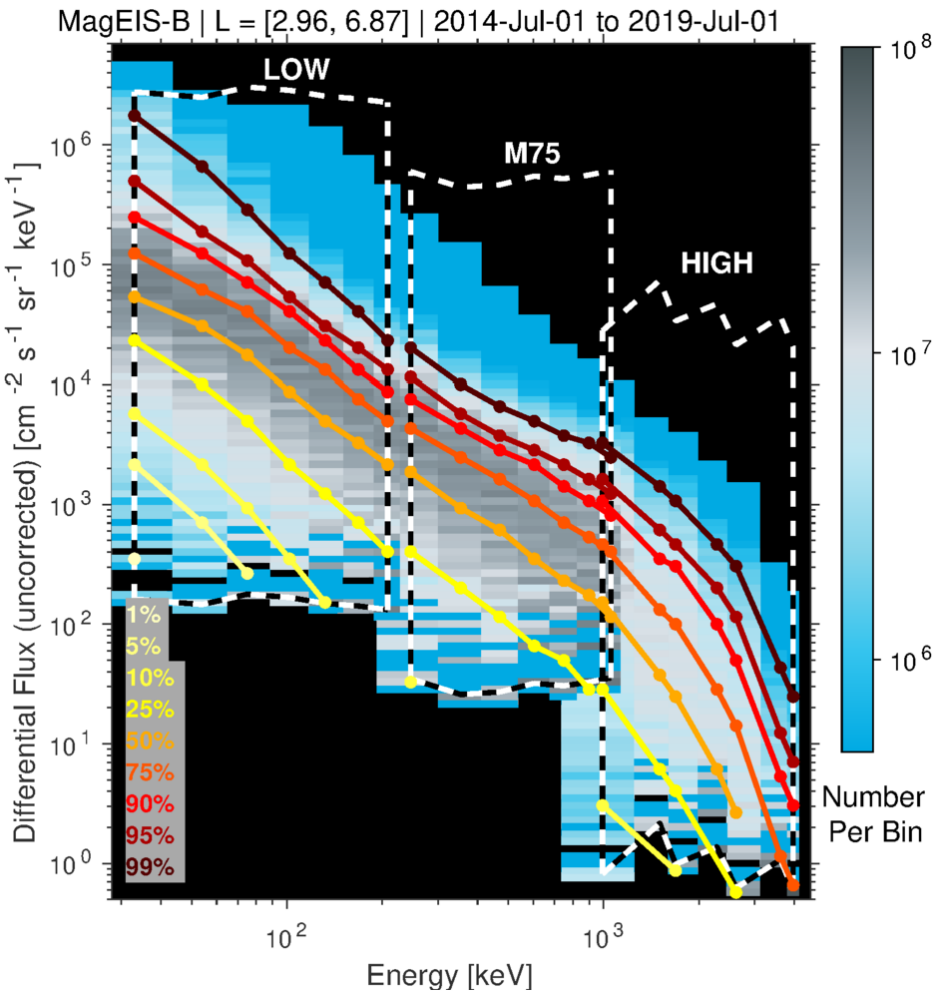
Sensitivity of the MagEIS electron instruments on Probe B, as flown (compare with Fig. 5 of Blake et al. 2013). The upper bound of each dashed-boxed region marks the flux level at the electronics saturation count rate of $10^{5}$ counts/s. The lower bound shows the one-count-per-sector flux level where a nominal spin period of 10.9 s has been used along with 64 sectors/spin for LOW/MED and 32 sectors/spin for HIGH. The width of each boxed region demarcates the centroids of the lower and upper energy range covered by each unit (note that only non-noisy LOW/MED channels are shown, i.e., the channels from pixels 2–8). The blue-grey color scale shows statistics on the energy-flux spectrum accumulated over the indicated $L$ and time range, along with intensity levels at various percentiles (yellow-through-red lines). The analogous figure for Probe A looks similar (not shown)

Extensive cross-calibration work by the ECT HOPE and MagEIS teams revealed that the lowest energy electron channels on MagEIS LOW suffered from occasional pileup and rate saturation. While this was an infrequent occurrence, comparisons between the common $\sim 30~\text{keV}$ channels on MagEIS and HOPE show that during intervals of very high fluxes, the MagEIS intensities can plateau while the HOPE intensities continue to rise (not shown here). This occurred when the MagEIS rates approached the electronics saturation level of $\sim10^{5}$ counts/s. These intense rates were only observed in the channels from LOW P2 ($\sim30~\text{keV}$) and LOW P3 ($\sim50~\text{keV}$). The effect is illustrated in Fig. 25, where we see that the lowest two MagEIS channels displayed (those from LOW P2 and P3) occasionally reached this saturation level. A percentiles analysis, similar to that shown in the figure, has revealed that instances of such rate saturation in MagEIS LOW were very rare, amounting to roughly $<0.1\%$ and $<0.01\%$ of the mission time for LOW P2 and LOW P3, respectively. As a conservative estimate, end users should exercise caution when MagEIS count rates were above $\sim 7.5 \times 10^{4}$ counts/s. Note that this rate threshold can be converted to more practical flux levels for a given energy channel using the $G_{0}\Delta E$ values provided in Table 5 and Table 6 (or from the tables provided in the Electronic Supplementary Material for earlier time intervals). The flux threshold maximums shown in the figure are computed in this manner, using the electronics saturation level of $\sim10^{5}$ counts/s. Note that these saturation flux levels can also be used as a proxy for when it may be safe to use data from the noisy LOW/MED pixel 1.

### Error Due to Counting Statistics

When particle count rates are low there is significant statistical uncertainty in the MagEIS measurements. There is a variable included in the level 2 and level 3 data files that can be used to quantify this counting-statistics error for both spin-averaged and angular-resolved fluxes. This percent error due to counting statistics is computed as (see Claudepierre et al. 2015):

5 $$ \textrm{error} = 100 \times \frac{\delta C}{C} = 100 \times \frac{\sqrt{1+C}}{C} $$

where $C$ is the counts accumulated over the integration time and the error is expressed as a percentage. Thus, the one count level over the integration time corresponds to 141% error in the uncorrected error variables. Note that these percent error variables are computed from the uncorrected data and are solely the error associated with counting statistics. They should not be confused with the additional percent error variables that are provided, which are defined and computed for the background-corrected electron data. We emphasize that these background-corrected errors are a combination of Poisson counting error for both the foreground and background counts, along with error terms due to background contamination itself. Thus, these errors can be considerably larger than the counting-statistics error computed solely from the uncorrected counts. To estimate the total uncertainty in the MagEIS measurements, one can combine these RMS error estimates with the bowtie uncertainties provided above in the root-sum-of-squares sense. However, the bowtie uncertainties are typically negligible when compared with the Poisson and/or background contamination error terms (e.g., $10^{4}$ counts must be achieved for a 1% Poisson error). Finally, we note that the error measure data products are spin-averaged from the angular-resolved error measures in the RMS sense:

6 $$ < \textrm{error}> = 100 \times \frac{\sqrt{< \delta C^{2}>}}{< C>} $$

where <> denotes the spin-averaging process.

### Data Quality Flags

The level 2 MagEIS data files contain a data quality flag that the end user should consult when undertaking any study using MagEIS data. We emphasize that the level 3 data files do not contain this data quality flag, though the level 2 data quality flag can be used as a guide when interpreting or analyzing level 3 data. The philosophy is to provide a simple quality flag that can take on one of three possible values (i.e., a red, yellow, green flag). These flag values are first determined on a sector-by-sector basis in each spin. The spin-averaged quality flag is then determined by taking the most frequently occurring value of the sectored quality flag in a given spin. For example, if there are 4 sectors per spin with corresponding $\text{flag values} = [0,0,1,2]$, then spin-averaged quality flag is 0 for that spin. Further details on the quality flags are provided in the Electronic Supplementary Material.

### Ion Telescope Threshold and Gain Settings

On 28 Sep 2012, we discovered that a reference voltage (“VREF_A”) used for the MPA detectors on the ion telescopes had been set incorrectly since launch. This setting rendered the detector gains and thresholds incorrect, meaning that all data taken from launch until this time were invalid when converted to energy. During this investigation, we also discovered that the thresholds of all detectors were set too high on the ion telescopes and these values were adjusted on 28 Sep 2012. Thus, MagEIS ion telescope data should not be used prior to 29 Sep 2012.

### Noise in Housekeeping Data

The DPU housekeeping circuits on the M35-A and, to a lesser extent, M75-A units were known to be noisy. This noise was related to the DPU temperature and worsened at higher temperatures. When the issue occurred, it produced anomalously large values for the yoke temperature (see Fig. 3) and anomalous values for the other housekeeping parameters (see Fig. 2). This behavior was observed pre-flight and it did not impact the quality of the MagEIS science data in any way. The root cause was believed to be incompatible logic levels between the FPGA and analog multiplexers. The issue never occurred on the other units.

## Instrument Anomalies

### Pixel 0 Failure on HIGH-A

At ∼11:25:00 UTC on 02 Oct 2013, the HIGH-A magnetic electron spectrometer experienced a permanent failure in the MAPPER channel attached to the rear detector in the pixel 0 stack. (The MAPPER was a custom multi-chip module that provided analog and digital processing in MagEIS – see Blake et al. 2013.) In the $\sim6$ months leading up to the failure, the noise in pixel 0’s rear detector had gradually increased by nearly an order of magnitude while all other detectors remained stable. The root cause of the noise build-up was never determined, though we note that the same HIGH unit experienced a nearly identical failure during pre-flight thermal-vacuum testing. This suggests that the root cause was not addressed by the repairs made prior to instrument delivery. Immediately following the failure, increased noise was observed in the front detectors in all four pixel stacks. This was a temporary anomaly attributed to an increase in cross-talk resulting from the chattering of the failed channel discriminator. This effect was corrected on 22 Oct 2013, when the threshold to the pixel 0 discriminator was commanded sufficiently high to prohibit further rate saturation. Also, at this time, the noise baseline in the front detector in pixel 0 returned to pre-failure levels. The other three rear detector pixels, in addition to the buried detectors, were not impacted by the failure and continued to produce nominal pulse-height resolution.

The failure of HIGH-A pixel 0 resulted in the total loss of data from the $\sim1~\text{MeV}$ channel on HIGH-A after this time. We note that this channel was redundant with the highest energy channel on the MED units, so that the impact was somewhat mitigated. However, background corrections are not possible on P8 on the MED units, so that after this time there is a significant gap in background corrected flux near this energy on Probe-A (i.e., between $\sim900~\text{keV}$ and $\sim1.5~\text{MeV}$).

We also note that both the rear detector and the buried detector in the HIGH-A pixel 1 stack exhibited some noise characteristics similar to what was observed on pixel 0 rear before it failed. However, these noise signatures were less pronounced and more transient relative to that of pixel 0 rear and did not impact the quality of the science data from HIGH-A P1 (see Electronic Supplementary Material).

### Suspected SEU on M35-B

At ∼02:00:00 UTC on 15 Jul 2015, the MagEIS M35-B unit suffered an anomaly while the Probe was entering the inner proton belt. The instrument registered very high count rates ($\sim10^{6}$ counts/s) in all 9 pixels and all 9 pixels were affected in the same way, suggesting a potential issue with the DPU. On 23 Jul 2015, the instrument was (soft) rebooted into maintenance mode and remained in maintenance mode until 30 Jul 2015, at which time it was rebooted into science mode. The anomaly was not seen when the instrument was rebooted into science mode and it continued to operate normally for the remainder of the mission. The suspected root-cause was a single-event upset (SEU). The lasting impact is the unavailability of science mode data from M35-B between 15 and 30 Jul 2015. Note that the reboot into maintenance mode can be seen in Fig. 2 as the temporary drop in bias voltage into the bias-off state ($\sim40~\text{V}$) around this time.

### Suspected SEU on LOW-A

At ∼02:20:00 UTC on 21 Aug 2017, the MagEIS LOW-A unit suffered an anomaly that prevented proper commanding of the unit. Some commands that were sent to the unit executed properly, while others did not, even consecutive commands within the same command script. The housekeeping and digital status data were nominal throughout the interval and no commanding errors were reported in the instrument command echo. The likely root cause was believed to be an SEU that led to a bit flip in the instrument’s RAM. This is consistent with the fact that some commands were received and executed, while others were reported as received and executed in the command echo but were not actually executed. The anomaly was ultimately cleared by soft rebooting the instrument into maintenance mode (on 19 Sep 2017) and then back into science mode (26 Sep 2017). We note that this same action corrected the earlier anomaly on M35-B in July 2015 noted above, which was also suspected to be an SEU. The lasting impact of the anomaly is a total loss of the science mode data (main rate, histogram, livetime) from 05 Sep 2017 through 25 Sep 2017, unfortunately during one of the largest geomagnetic storms of the Van Allen Probes era. We do note, however, that there is nearly continuous high-rate mode data available from LOW-A from 30 Aug 2017 through 19 Sep 2017 and intermittent (but sparse) science mode data from 21 Aug 2017 through 04 Sep 2017. Note that the reboot into maintenance mode can be seen in Fig. 2 as the temporary drop in bias voltage into the bias-off state around this time.

## Lessons Learned and Future Design Considerations

### Noise in LOW/MED Pixels 0 and 1

**Fig. 26:**
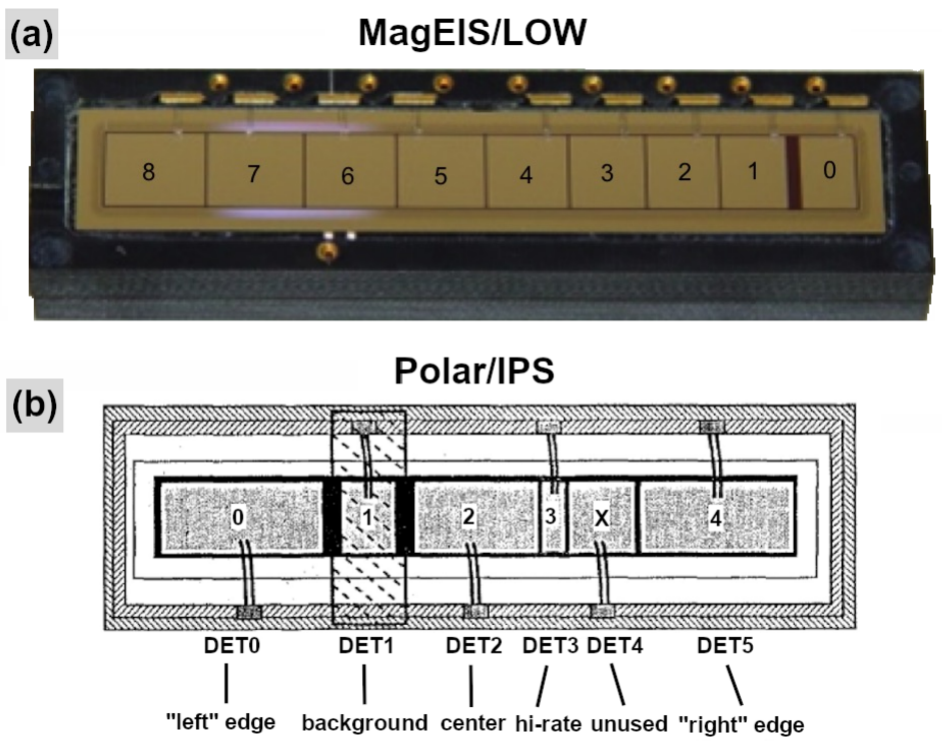
(**a**) MagEIS LOW detector array in black mount showing the large gap between pixel 0 and pixel 1. (**b**) Scale drawing of a Polar/IPS detector array showing the six pixels surrounded by a guard ring (black strip around all pixels). The detector was supplied by Micron Semiconductor Limited

As we learned, after observing noise in the LOW/MED pixels 0 and 1 (Sect. 6.1), the gaps between the pixels need to be kept small to reduce field changes from charge buried in the unbiased silicon. In the LOW/MED sensors, the gap between pixel 0 and pixel 1 was large by design (see Fig. 26a) to allow space for a background shield to be placed over pixel 0 without impeding electron access to pixel 1. However, the positioning of the shield was not as expected, resulting in part of pixel 0 and the gap between pixel 0 and pixel 1 being illuminated by electrons. The electrons that buried themselves in the gap modified the electric fields near the two pixels, resulting in a leakage current that created noise in these pixels. The effect maximized following intense magnetic storm disturbances and high flux events. During intervals of low fluxes and quiet times, the noise level decayed to low levels, allowing the pixel 1 data to be usable some of the time on some of the units (see Fig. 24).

In follow-on magnetic spectrometer units, we eliminated the wide gap between pixel 0 and pixel 1. As noted, this noise problem was not obvious in the case of low-to-modest electron intensities. Our laboratory beta spectrometer has sufficient intensity to perform calibrations in a timely fashion but does not have intense beams. There is the issue of personal safety and one is loath to “blast” flight hardware with extreme fluxes. In retrospect, we could have exposed just the focal-plane detector in The Aerospace Corporation’s Cobalt-60 irradiator that is used for determining the radiation hardness of electronic components.

It is also possible to have guard strips surround the individual pixels, as was done for the Imaging Electron Spectrometer (IES) and Imaging Proton Spectrometer (IPS) telescopes in the Polar/CEPPAD sensors (Blake et al. 1995). Some of these pixels had large gaps between them but the individual detector elements were surrounded by guard strips (see Fig. 26b). No inter-pixel noise was observed in these sensors. The MagEIS detectors had a guard strip around the array of pixels but not around the individual pixels.

### Noise Development in the Ion Telescope MPA Detectors

The detectors used in the MagEIS ion telescopes were ion implanted, thin deadlayer, silicon detectors from Micron Semiconductor Limited. Foils were not placed in front of the MPA detectors, so they were exposed directly to the space plasma. After the sensors had been on orbit for $\sim6$ months, we observed that the lowest energy channels were becoming noisy (see Sect. 6.7). As time on orbit increased, so too did the noise, which slowly affected higher energy channels. It was concluded that the normal plasma energy ions were implanting into the deadlayer of the front MPA detectors of both the A and B ion telescopes, modifying them and causing the noise. Once on orbit, there was nothing we could do to reverse the process. We also believe that this led to the decrease in efficiency of detection in the highest energy channels noted above, though it may be more appropriate to regard this as a mislabeling of the energy channels. The ion implantation in the front side of the detector increases the deadlayer, through which the incident particles must traverse, depositing unmeasured energy in the process. This effectively increases the lower energy threshold of the instrument, which results in higher energy particles being labeled as if they were at lower incident energies. This manifests as a decrease in intensity over time (i.e., as time progressed, the detector was measuring higher and higher energy ions but calling them the same, incorrect energy).

The ion telescope had a strong sweeping magnet in the entrance collimator for the purpose of sweeping away electrons up to relativistic energies. This “broom” magnet was tested in the laboratory with radioactive electron sources and a beta spectrometer and found to be highly effective, keeping out up to 1.8 MeV electrons. It should have also kept out up to 2 keV protons. At the time, we did not have ready access to a low-energy proton source ($\sim\text{keV}$) and assumed that blocking the low energy ($\leq2~\text{keV}$) proton access, as well as relativistic electrons, would be sufficient to protect the detectors. Why this was not the case still is not understood. Similar ion detector degradation was also observed in the Solid State Telescope (SST) ion instruments on NASA’s THEMIS mission, which also used a sweeping magnet and had no foils to reject incident electrons; the same ion implantation also might have affected those instruments. The lesson learned was that one needs to protect detectors like the MPA from direct contact with somewhat higher energy plasma ions than 2 keV. A thin, light-tight foil that stopped $\sim10~\text{keV}$ protons might have provided better protection while still allowing the telescope to measure $>50~\text{keV}$ protons. Such a design consideration is important for future ion instruments, given both the ion implantation damage and the light contamination observed in the MagEIS ion telescopes. For example, a 0.2 μm, light-tight nickel foil was used for the micro-Charged Particle Telescope on the AeroCube-10 CubeSats (Turner et al. 2018) to eliminate both of these problems with the same, easy-to-implement solution. We note that a thin carbon foil will mitigate the deadlayer growth from ion implantation, but it will not help with light contamination. On the other hand, a thin nickel foil attenuates light from the Sun with a factor of $>10^{4}$ for the thermal through EUV portions of the spectrum, which we demonstrated in testing at The Aerospace Corporation’s Sun simulator facility (Turner et al. 2018, 2019b).

### Modified Electron Detector Bias

The MagEIS electron detectors, as launched, were over-biased by a factor of two in accordance with the recommendations from the detector supplier. Once on orbit it was found that the detector noise levels in LOW/MED pixels 0 and 1 would rise following very high flux events. Some laboratory testing and discussions with the detector supplier indicated this issue might be eliminated or reduced if the detector over-bias levels were reduced. However, the units as launched did not have the ability to change bias levels by command. Discussion with the bias supply designer and the software engineer indicated it could be possible to reduce the bias levels by changing the supply’s operating frequency by turning it on and off at a high rate such that the duty cycle was reduced. Laboratory testing showed that this could be done without damaging the supplies or affecting the performance of the rest of the system. This technique was implemented by uploading new software to the LOW/MED units that allowed the duty cycle of the supplies to be set by ground command. The technique performed well and was successfully used throughout the rest of the mission on the LOW/MED units. This is a technique that can be built into any new units. However, this lowering of the biases did not eliminate the noise in P0 and P1, as discussed above.

Figure 2 shows that the detector biases on the LOW/MED units gradually declined over the course of the mission, likely due to increased leakage currents. While we do not believe that this decrease impacted the quality of the science data, it is interesting to note that this decline was not observed on the HIGH units. This could perhaps be related to the pixel 0/1 noise on the LOW/MED units. For example, the increased leakage current through the pixel 0 gap (see Sect. 8.1) could draw enough current to cause a voltage drop through the bias resistor. The HIGH unit had a different detector design that did not have the pixel 0 gap.

### Importance of Histogram Data

Claudepierre et al. (2015) demonstrated the critical importance of how the MagEIS magnetic spectrometry technique, combined with full histograms of counts as a function of energy on each pixel, can be employed to effectively identify and remove background contamination from the true target signals. In key regions, such as the slot region and inner radiation belt, this background removal technique resulted in corrections of errors on the order of several hundred percent and higher. Such errors went uncorrected in past (and most present) observations of high-intensity radiation environments at Earth and other systems in the Heliosphere. This contamination previously obscured the true energy spectra in Earth’s electron radiation belts, which was reliably recovered in the background-corrected MagEIS data. Thus, it is critical that any future iterations of MagEIS (or any spectrometers for that matter) employ a strategy for effective background removal with lessons learned from MagEIS (e.g., Claudepierre et al. 2015, 2017, 2019). In an ideal, telemetry-rich scenario, one would obtain only histogram-like data on-orbit and produce main channels and derived products in post-processing on the ground.

### HIGH Unit Electron Coincidence Assessment

The coincidence processing for the MagEIS HIGH electron spectrometer was not done by hardwired electronics. Instead, the timing of the above-threshold events for the front and rear detectors were compared digitally to see if they fell within a coincidence time window. This window was set by command and initially, at launch, was fixed at 20 μsec. Examination of the early flight data suggested that this caused the acceptance of too many front and rear detector events that were spurious coincidences of separate particles in each detector. We systematically changed and tuned the coincidence window until we settled on a value of 5 μsec. That gave good rejection of spurious coincidences without unreasonable reduction in detection efficiency for actual coincidences due to a single particle. Importantly, the coincidence requirement improved the background rejection on the HIGH units relative to the similar energy pixels on the MED units, which saw comparatively more background in the inner zone despite being physically smaller.

### The Value of Geant4 and LORENTZ Simulations

The Geant4 simulations described in Sect. 4.1 were an important part of the MagEIS calibration effort. Sensor simulation technology has now reached the level of sophistication and stability that it is likely productive to engage in a high-fidelity simulation as part of the calibration campaign rather than, as has been prior custom, to wait until the sensor is on orbit to use the most advanced simulation possible. Such simulations can help guide the calibration campaign and may even aid in minor design and flight software modifications. However, one must balance resources devoted to such efforts with the sophistication and complexity of the instrument (e.g., a Geant4 simulation of a range telescope is much simpler than a magnetic spectrometer). Similarly, the level of maturity and capabilities of Geant4 may be insufficient for certain instruments or subsystems in the design phase, as was the case for the Cosmic Ray Telescope for the Effects of Radiation (CRaTER) instrument on the Lunar Reconnaissance Orbiter (e.g., Looper et al. 2013).

The Geant4 simulations and other studies of the response of the MagEIS spectrometer sensor revealed several aspects of the instrument design and operation that were not optimal. For example, the orientation of the antiscattering baffles within the yoke chamber, which were arranged in a fan configuration that was centered on the slit, was found to be non optimal. In subsequent calibration work for a MagEIS-like follow-on instrument (see Sect. 8.11), it was determined that a better orientation for the baffles was to point directly to the spot between the slit and the first pixel and placed so that they are approximately orthogonal to the electron trajectories (see Fig. 28 below). In addition, the Geant4 simulations revealed that the HIGH unit response was quite sensitive to the front detector thresholds, which were high enough to reject some valid events with slightly lower energy deposits in the front detector for electrons penetrating to the rear detector (see Sect. 4.1). This meant that the response of the sensor was strongly dependent on the exact value of the thresholds, and thus to errors in our knowledge of those thresholds (for example, compare the reduction in the coincident response of HIGH pixel 0 in Fig. 13b relative to that of pixels 1–3). We also stress the importance of performing a simulation of the instrument response to omni-directional particles (i.e., not simply those that come in parallel to the axis of the instrument aperture), as we found these results to be substantially different from narrow beam simulations.

For the MagEIS spectrometer, the LORENTZ simulation of the yoke magnetic fields was also crucially important, both in evaluating design configurations for the magnetic circuitry and in calibration efforts (when combined with the Geant4 response). For example, using the LORENTZ field versus the nominal, uniform field made a significant difference in the bowtie calibration factors that were obtained and used for the flux conversions (see Sect. 4). We note that the only pre-flight measurement of the yoke field was taken at room temperature, while the on-orbit field was most certainly different due to temperature dependencies in the magnets. For example, the magnets used in the HIGH unit (NdFeB) had a coefficient of temperature of $\sim -0.13\%/\text{C}$, which was substantially higher than those of the LOW/MED units (SmCo; $\sim -0.04\%/\text{C}$). This may explain why it was necessary to scale the yoke chamber magnetic fields in order to bring the Geant4/bowtie results into alignment with the on-orbit data, as noted in Sect. 4.1 and described in greater detail in the Electronic Supplementary Material. A more significant difference between the nominal field and the LORENTZ field was that the latter “flares” toward the edges of the magnets, which can steer electrons away from the detectors. All of these effects and the impact they had on calibrations were only appreciated once the simulations had been conducted and analyzed in detail.

It is significant that a full, pre-flight mapping of the yoke magnetic field was not performed and the temperature dependence of the magnetic field was not measured. The magnetic circuits were required to be tight (minimal leakage fields) and efficient (minimal usage of heavy materials). These requirements prevented us from mapping the field within the magnetic units thoroughly with a probe. Rather, the approach taken was to reference a single point within the field that was accessible with a Hall probe. The measured field at that point was used to adjust the amount of saturation of the permanent magnets such that the measured field at that point matched the modeled field at that point. From that juncture forward, the modeled field was taken to be sufficiently accurate to be used in subsequent Geant4 simulations, which is a testament to the accuracy of state-of-the-art modeling tools such as LORENTZ. Similarly, the dependence of the magnetic field on temperature was not measured, as a Hall probe has a significant response to temperature. Again, the philosophy was that modeling was the most accurate way to understand how the field varied with temperature. In this regard, the magnetic circuits were required to be maintained within a fairly narrow temperature range to keep the amount of variation on orbit low.

### Calibration Test Pulser Sweeps

For much of the mission, the calibration pulser in the MagEIS magnetic spectrometers was run at perigee nearly every orbit. Perigee was chosen since, on the majority of passes, the Van Allen Probes were below the radiation belts there. In hindsight, this frequency ($\sim2\text{--}3$ times per day) was more often than needed and contaminated real data taken near perigee. On 01 Nov 2017 the cadence of the calibration pulser operations at perigee was reduced to once per month to allow electron data to be taken at most perigees.

The perigee pulser calibrations showed that the electron spectrometer performance varied little over the mission and the frequency of pulser tests could have been significantly reduced early on. The pulser calibrations were also useful in defining the ion telescope gain responses for the very thin (2μ, 9μ) and thick MSD detectors for the on-orbit temperatures achieved. These gains were different from what was observed pre-flight in the laboratory, where only very limited data were taken as a function of temperature. The final calibrations of these ion detectors are still somewhat uncertain; we would have greatly benefited from more time spent measuring the temperature-dependent gains for the ion telescope system.

### Telemetry Flexibility

We developed MagEIS to have significant flexibility in generating output telemetry. While that capability was initially little used, it became very valuable later in the mission. The primary negative impact of the telemetry-constrained operations early in the mission was that histogram data could not be recorded at high time resolution. Once on-board data compression was developed and implemented in the satellite data system, MagEIS was able to quickly take advantage by increasing the number of angular sectors per spin period and reducing the accumulation time intervals. The sample rates and sectoring for the histogram data were increased such that it became a very useful data set for background corrections and for providing high energy resolution spectra. The flexibility that MagEIS had provided a great ability to use increased telemetry as it became available. This should be kept in mind for any future missions. Some telemetry flexibility is good, though it can complicate both instrument operation and downstream data processing/continuity as remarked in Sect. 6.6.

### Pitch Angle Coverage

MagEIS was limited in its pitch angle coverage by the angle between the spin axis, the direction of the magnetic field vector, and the mounting of the MagEIS units on the satellites. The mounting offset from perpendicular to the spin axis was chosen based on experience with the CRRES mission, which was also in a GTO orbit. However, the angular coverage achieved on orbit was compromised by the tilt of the spacecraft spin axis relative to the sun line, which was imposed to keep the axial electric field boom tips illuminated at all times. As a result, the $75^{\circ }$-pointing MagEIS units (LOW, M75, and HIGH) rarely viewed close to the field line direction and the minimum pitch angle sampled was often $>10\text{--}20$ degrees. In hindsight, it would have been closer to optimal to mount the MagEIS units with their fields-of-view perpendicular to the spin axis, given the tilt requirement. This also would have simplified the mounting since offset mounting brackets would have been unnecessary.

**Fig. 27:**
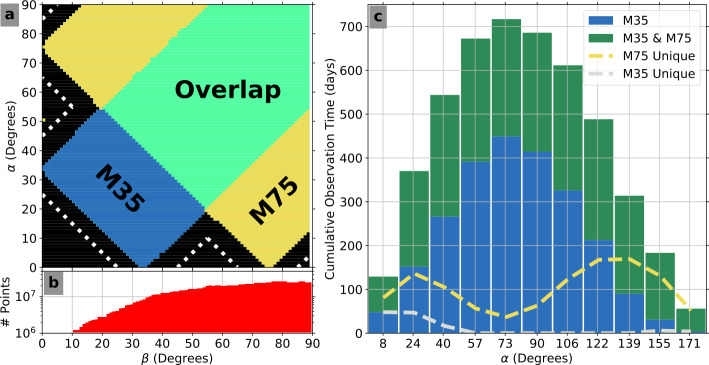
Summary of M35 vs. M75 pitch angle coverage from Probe-B. (**a**) The 0–90 degree local pitch angle ($\alpha $) coverage vs. the angle between the spin axis and the magnetic field ($\beta $). Regions only observed by M35 are shown in blue, only by M75 in yellow, and observed by both in green. Dashed lines indicate the original, anticipated (pre-flight) boundaries from Fig. 12 of Blake et al. (2013). (**b**) The occurrence frequency of $\beta $ over the entire mission. (**c**) The cumulative observation time in each pitch angle bin measured using M35 only (blue) and using both M75 and M35 (green). The dashed lines show only the unique observations for M35 (grey) and M75 (yellow)

As described in Sect. 1, MagEIS featured two medium energy units to enhance pitch angle coverage when the magnetic field was highly stretched. However, the final orientation of the spacecraft noted above compromised this design feature as well. Figure 27a shows the pitch angle coverage of the M35 and M75 units as a function of the angle between the spin axis and the magnetic field, $\beta $. As shown with the blue region, the M35 detector did offer some additional coverage when the magnetic field was highly stretched (small $\beta $ angles). However, this improvement was very limited and did not meet pre-flight expectations (e.g., Blake et al. 2013) due to the imposed spacecraft tilt. Figure 27c shows the cumulative observation time in each pitch-angle bin for both detectors. While M35 did offer a considerable number of observations across different pitch angle bins, most of these overlapped with M75. The unique M35 observations (i.e., times when only M35 observed a pitch angle bin) were largely confined to the pitch angle bins closest to 0 degrees, which only represent 3.1% of total observations. Given these results and the challenges inherent in combining the M75 and M35 datasets (for example, they do not share a common set of energy channels), the MagEIS team decided not to produce a merged M35/M75 data product.

### Higher Density Detector Arrays and Increased Energy Resolution

As described above, the MagEIS LOW and MED units used 9-element detector arrays, and from those, only 8 pixels were used for science data products. However, the MAPPER ASIC-hybrid chip that was used for the front-end detector signal processing in the electronics can handle inputs from up to 10 detectors. For future iterations of MagEIS-like magnetic spectrometers, science output can be optimized by using 10-element detector arrays per MAPPER chip. It is now possible, with advances in high density electronics, to put many more pixels in the spectrometer focal plane. The pixel width could be the width of the momentum resolution of the magnet. One advantage would be reduced sensitivity to pileup and saturation as the total count rate would be distributed over many more channels. Rather than pulse height analysis on each pixel to generate histograms, one could have as many pixels as MagEIS had histogram channels (64, though ideally 60 or 70 considering MAPPER inputs). Electronically, it would be very simple: each pixel would have a narrow passband window in energy. However, one would have to have a greater-density amplifier ASIC, and possibly require higher power and greater telemetry bandwidth to accommodate such data, if nominal 1/64 of a spin ($\sim8~\text{msec}$) temporal sampling was desired. Geometric factors and expected counting rates must also be considered for such an approach. In addition, narrower-width pixels will experience an increased loss of electrons out of the sides via scattering, distorting the pulse height distribution (like the “pixel edges” in Fig. 12). Such an effect must be considered if one is to replace histograms with large numbers of narrow pixels.

### Measuring Protons/Ions in the Magnetic Chamber

**Fig. 28:**
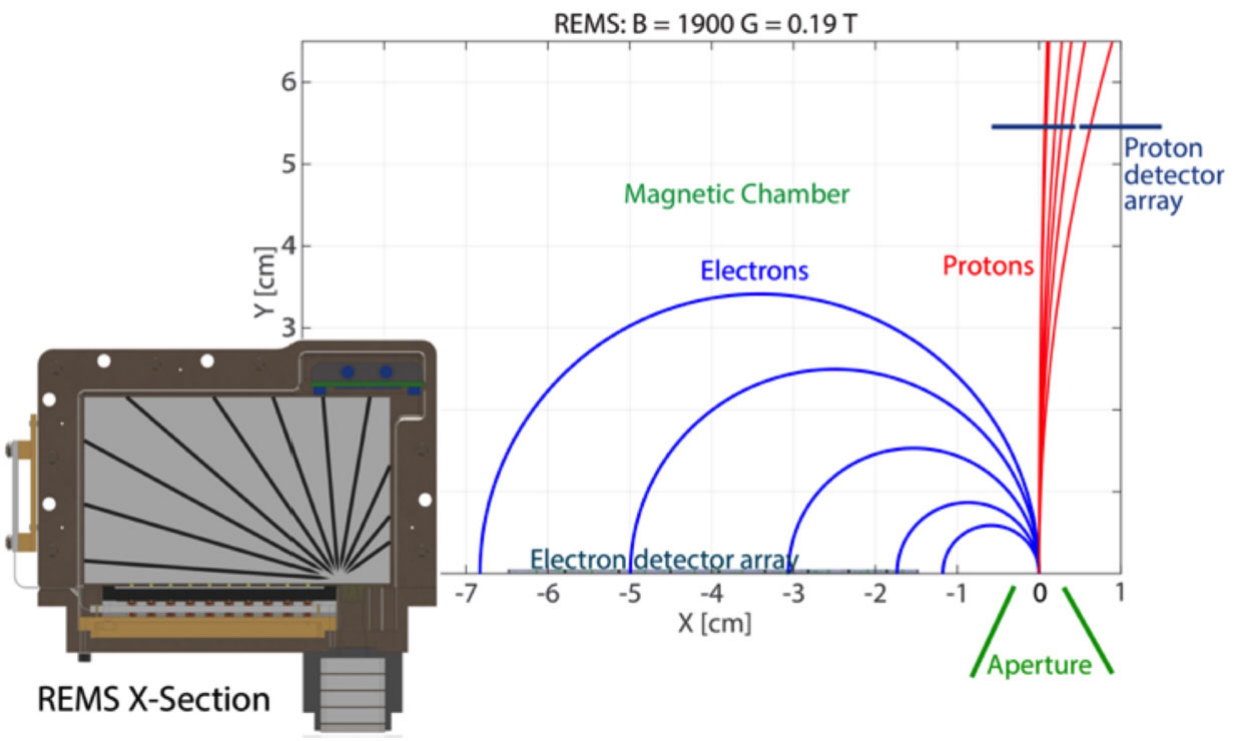
The REMS sensor, a next-generation instrument derived from MagEIS MED, to be flown on the GTOSat CubeSat. Particle trajectories (coming in straight along the aperture boresight) for 100 keV to 2 MeV electrons (blue traces) and 150 keV to 10 MeV protons (red traces) are shown, obtained from test-particle simulations in a uniform, 1900 G chamber magnetic field (oriented in the $+Z$ direction). The electron detector array is plotted along the $X$-axis and the proton detector array is shown in the upper right. The inset image shows a cross-section of the magnetic chamber in this same $XY$ plane. After Blum et al. (2020) ©Society of Photographic Instrumentation Engineers

The magnetic spectrometer measurement technique can also be employed for a clean measure of energetic ions, as was shown in the S3-3 magnetic electron-proton spectrometer (Cattell 1982; Vampola 1996). Because of the electron-to-proton mass ratio and opposite charge, electrons up to very high energies (several MeV) are steered away from the corner of the magnetic chamber across from the instrument aperture (see Fig. 28). Due to their much larger gyroradii, energetic (100s keV to several MeV) protons and other positively charged ions, however, are guided directly into the corner of the chamber by the same magnetic field. As Fig. 28 shows, detectors (or stacks of detectors) can be placed in the back corner of a magnetic spectrometer chamber to provide a clean (i.e., largely free of incident electrons, though still susceptible to penetrating-particle background) measure of energetic ions. A new version of such a unit, the Relativistic Electron Magnetic Spectrometer (REMS) instrument, is currently in development for NASA’s GTOSat mission (Blum et al. 2020), a 6U CubeSat mission to study Earth’s radiation belts from GTO. REMS incorporates this ion measurement strategy using an additional pair of detectors to measure fluxes of $\sim100~\text{keV}$ to $\sim8~\text{MeV}$ protons and other heavier ions in the Earth’s ring current and inner radiation belt. Such a strategy can be employed in future iterations of MagEIS-like spectrometers to enhance the scientific return from these instruments.

## Supplementary Information

Below are the links to the electronic supplementary material.

The primary document that contains various figures and text that expand upon many of the points described above. This document also contains tables of times of LUT changes and detailed descriptions of the variables in the level 2 and level 3 data files (PDF 8.8 MB)

Tables of the calibration (energy channel definitions/flux conversion) factors for all of the LUTs used on orbit for the electron high rates (PDF 91 kB)

Tables of the calibration (energy channel definitions/flux conversion) factors for all of the LUTs used on orbit for the electron histograms (PDF 218 kB)

Tables of the calibration (energy channel definitions/flux conversion) factors for all of the LUTs used on orbit for the electron main rates (PDF 107 kB)

Tables of the calibration (energy channel definitions/flux conversion) factors for all of the LUTs used on orbit for the ion main rates (PDF 72 kB)

Figures indicating the instrument mode (e.g., science, high-rate, or maintenance mode) for all 8 units over the course of the mission (PDF 3.0 MB)

Figures indicating the availability of the daily MagEIS CDF data files (levels 1, 2, and 3) for the entire mission (PDF 10.9 MB)

A chronological listing of configuration changes (e.g., LUT changes, accumulation parameter changes) and known instrumental/spacecraft issues that led to missing data files and/or large data gaps (PDF 60 kB)

## Data Availability

All MagEIS data used in this manuscript are from the “final” data release and are available in the public domain at NASA’s Space Physics Data Facility (SPDF). The MagEIS electron spectrometer 3D response files are available in the Dryad data repository at the following link: https://doi.org/10.5068/D10M4S.
