# Supplementary material for: The Magnetic Electron Ion Spectrometer: A Review of On-Orbit Sensor Performance, Data, Operations, and Science
Source: Space Sci Rev. 2021 Oct 28;217(8):80. doi: 10.1007/s11214-021-00855-2 (PMC8553741; doi:10.1007/s11214-021-00855-2)
Supplement: Supplementary file 1 — The primary document that contains various figures and text that expand upon many of the points described above. This document also contains tables of times of LUT changes and detailed descriptions of the variables in the level 2 and level 3 data files (PDF 8.8 MB) [file 11214_2021_855_MOESM1_ESM.pdf]

Electronic Supplementary Material for *The Magnetic Electron Ion Spectrometer: A Review of On-Orbit Sensor Performance, Data, Operations, and Science*

S. G. Claudepierre, J. B. Blake, A. J. Boyd, J. H. Clemmons, J. F. Fennell, C. Gabrielse, M. D. Looper, J. E. Mazur, T. P. O'Brien, G. D. Reeves, J. L. Roeder, H. E. Spence, and D. L. Turner

October 26, 2021

1 Introduction

This document provides additional supporting information and material relevant to the topics presented in the main manuscript. Sect. 2 provides further details on a number of the "Data Caveats" described in the manuscript. MagEIS lookup table (LUT) changes are documented here (Sect. 2.3) for all 8 units and all LUT types (main rate, histogram, and high rate). The descriptions of several of the data products introduced in the manuscript are expanded upon in Sect. 3, including the conversion of histogram data to flux. In Sect. 4, we discuss several points related to how LUT and instrument configuration changes impacted the calibration factors. Sect. 5 examines the MagEIS electron spectrometer's angular response as simulated by Geant4. In Sect. 6, we provide additional details on the scaling of the chamber magnetic field that was necessary to bring the Geant4 simulations into alignment with the flight histogram data. Finally, Sect. 7 describes the contents and variables in the level 2 and level 3 MagEIS data files.

In addition to the information provided in the sections below in this document, the following documents are also provided as part of the Electronic Supplementary Material:

1. Tables of the calibration factors for all of the LUTs used on orbit (see Sect. 2.3) for the electron main rates, electron histograms, electron high rates, and ion main rates. A few additional details are provided in Sect. 4.

2. Figures indicating the instrument mode (e.g., science, high-rate, or maintenance mode) for all 8 units over the course of the mission. Note that these summary plots can be scanned, for example, to look for instances of high-rate mode data taken continuously over multiple days. Each page in the PDF displays one month of data so that there are 86 pages in the document.

3. Figures indicating the availability of the daily MagEIS CDF data files (levels 1, 2, and 3) for the entire mission. The plots are presented in stoplight red/green format, where green indicates that the data file exists, and red indicates that it does not. Each page in the PDF displays one month of files so that there are 86 pages in the document.

4. A chronological listing of configuration changes (e.g., LUT changes, accumulation parameter changes) and known instrumental/spacecraft issues that led to missing data files and/or large data gaps.

2 Data Caveats

2.1 Noise in LOW/MED Pixels 0 and 1

As described in the main manuscript, pixels 0 (P0) and 1 (P1) on the LOW/MED units experienced noise. Figure 1 shows L sorted plots of P0 data from all six LOW/MED units, analogous to the plot for P1 data

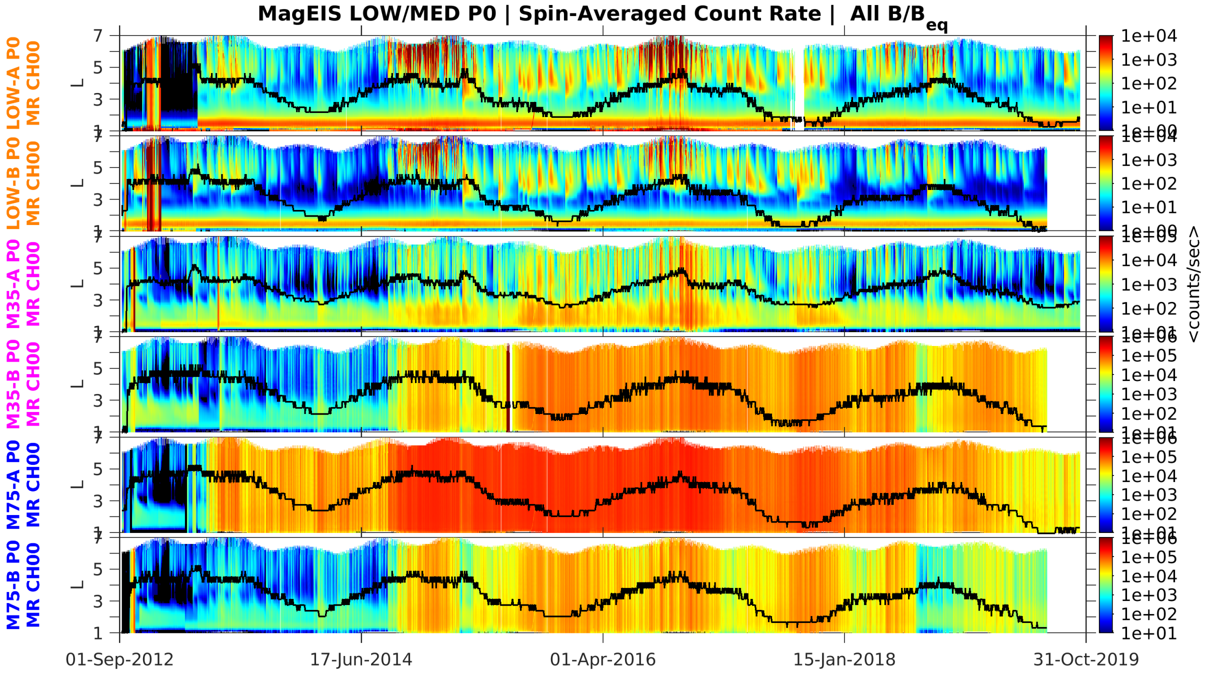

Figure 1: Noise in LOW/MED P0. The raw, spin-averaged count rates are daily-averaged and binned in L. The daily maximum yoke temperature is overplotted in black, where the units are arbitrarily scaled to the L range of the plot window (the peak-to-peak range is $\sim 5^\circ$ C).

shown in the main manuscript. We note that P0 was partially shielded and was intended to serve as a background monitor, so that it did not provide scientifically useful electron fluxes irrespective of any noise considerations.

2.2 Major Changes in HIGH Electron Spectrometer Logic

Table 1 lists the major changes and reconfigurations of the HIGH unit, along with intervals where thermal oscillations were observed. The significance of these points is described in the main manuscript. The end user should be aware of these issues with the HIGH unit electron data, again emphasizing that the data should only be used qualitatively prior to 26 Sep 2013.

2.3 Time-varying Energy Channels due to Lookup Table Uploads/Changes

When a new main rate LUT was uploaded or implemented on a given MagEIS unit, this changed the energy channel definitions and flux conversion factors. We note that within a given daily CDF file, there is a fixed set of (non time-varying) energy channels. The energy channels may change from day-to-day when new LUTs were uploaded, but for a given day the channel definitions are fixed (e.g., the energy channel variables are not time-varying in an individual daily file).

Table 2 through Table 6 list the dates and times (in UTC) of LUT table changes for the electron main rates, electron histograms, ion main rates, ion histograms, and electron high rates. Note that the dates and times indicated can be either the date/time of the LUT upload or the date/time of a table switch, or both. In Table 2 through Table 6, an entry of “NONE” indicates that there was not a LUT uploaded to the instrument prior to that time.

In Table 2 through Table 6, an entry of “ENG” indicates an engineering LUT, which was used for instrument diagnostics and did not produce science-quality data. In addition to their use early on in the mission, these ENG tables were also often used during the test pulser calibrations performed at perigee (see main manuscript). The following LUT IDs are of ENG type and data taken when these LUTs were in use

should not be considered valid: (65520, 65521, 65522, 65523, 65524, 65525, 65526, 16144, 16149, 16160, 16165, 16176, 16181, 16192, 13824,13825, 13826, 65284). In addition, the following science-grade LUTs were deemed to be invalid for various reasons and science data taken with these LUTs should be discarded: (16640, 20736, 24832).

2.4 Time-varying Sampling Parameters (Sectoring and Accumulation Times)

In addition to instrument reconfigurations via LUT uploads/changes, sensor performance was also optimized on-orbit via several parameters that were configurable via ground-command. As described in the main manuscript, two of the most important were the number of spacecraft spins over which data were accumulated (nspins) and the number of angular sectors per spacecraft spin (nsectors). We emphasize that these values were changed frequently during instrument commissioning (Sep and Oct 2012) and early operations, but settled into relatively stable values as the mission progressed. We note that the pitch-angle binned data in the level 3 data products are somewhat insensitive to the sectoring changes. This is because a fixed number of pitch-angle bins are used for the entire mission, regardless of the specific sectoring on a given unit at a given time. Of course, early in the mission when the angular sectoring was coarse, some pitch angle bins may have lacked coverage relative to later times when the sector resolution increased.

2.5 Noise in HIGH Electron Detectors

As described in the main manuscript, on 02 Oct 2013 the HIGH-A magnetic electron spectrometer experienced a permanent failure in the MAPPER channel attached to the rear detector in the pixel 0 (P0) stack. In the ∼6 months leading up to the failure, the noise in P0's rear detector had gradually increased by nearly an order of magnitude while all other detectors remained stable. At later times in the mission, both the rear detector and the buried detector in the HIGH-A pixel 1 (P1) stack exhibited some noise characteristics similar to what was observed on P0 rear before it failed. However, these noise signatures were less pronounced and more transient relative to that of P0 rear and did not impact the quality of the science data from HIGH-A P1. Since the buried detectors were not used, HIGH-A P1 buried was disabled (threshold raise) by ground command in Nov 2017. HIGH-A P1 rear began to exhibit noise signatures in the detector singles data in Jul 2017, which was monitored until the end of the mission. However, this noise was transient, at times going away completely, and did not steadily increase with time as was the case for HIGH-A P0 rear before it failed. More importantly, the noise in HIGH-A P1 rear was less severe than that in P0 rear and was not observed in the front+rear coincidence data, so that the impact on the science data was minimal.

2.6 High-Rate Data Issue With Maximum Sectoring (2048 Sectors)

In Oct 2015, we commanded the M35-A and M35-B units into high-rate (HR) mode with the maximum possible sectoring of 2048 sectors per spin. Upon analyzing the data, it was determined that there was a timing issue when the maximum possible sectoring was used. Specifically, we determined that there was double counting (twice the true number of counts) in the first 10s of sectors in each spin. This was due to a mismatch between the actual sector time and that was computed in the flight software, which was coarse due to the 1/4 msec time resolution on the sector time.

For example, with an assumed spin rate of 5.481 RPM (a 10.947 sec spin period) and 2048 sectors (the parameters on Probe B in Oct 2015), the true sector time is ∼5.35 msec, which is resolved by the flight software to be 5.25 msec due to the 1/4 msec time resolution. Thus, the spin period computed by the flight software for the next spin is 10.752 sec (5.25 msec $\times$ 2048). This mismatch between the true spin period and that computed by the flight software leads to 195 msec of rollover at the end of each spin, resulting in double counting in the first ∼37 sectors of the next spin ($37 \approx 195/5.25$). A similar analysis for M35 on Probe-A, where the where spin rate was ∼5.4 RPM, showed 68 bad sectors at the beginning of each spin. Thus, we analyzed the spin rates for both Probes in the Sep-Oct 2015 time frame to determine that the minimum spin rate was 5.385 msec (Probe A). In this case, there are 390 msec of bad sectors, or ∼75 bad sectors. To allow for this worst case, the M35 sectoring was lowered from 2048 to 1960 sectors to mitigate this timing issue, allowing for some margin. We note that there were ∼10 days in Oct 2015 where the M35 sectoring was set to 2048 before it was lowered to 1960, so that the end user should be aware of this issue

if analyzing data from this time interval. When the sectoring was set to 2048, the data from the first 100 sectors in each spin is set to fill value.

This timing mismatch was not an issue for the main rate data, which had a maximum possible sectoring of 64 sectors per spin. This is because the maximum possible duration of the rollover due to the 1/4 msec time resolution ($\sim$16 msec) was much less than the duration of the sector time ($\sim$171 msec), for 64 sectors and nominal values of the spin period.

2.7 Noise and Efficiency-of-Detection Issues in Proton Channels

Figure 2 illustrates the development of the noise and efficiency-of-detection issues in the MagEIS MPA proton data on both Probes. Prior to mid-2013, the detectors were producing valid data, which can be seen most clearly in the inset panels. In mid-2013, the lowest 5 or 6 energy channels began to display noise, while the higher-energy channels began to gradually decline in intensity. By the end of the mission, the count rates in the highest energy channels had decreased by roughly 3 orders of magnitude. Note that the count rate in the lowest energy channel was initially different in the inner and outer zone (black vs. red), but then reached the same value, indicating noise development. The two inner zone traces (black and grey) in particular suggest that the issue was instrumental. In the inner zone, the MPA proton measurement was largely dominated by inner belt proton contamination. As the very energetic inner belt protons that cause this contamination were relatively steady over the time scale displayed, one would expect these count-rate profiles to be relatively constant in time throughout the mission. They were at first, but the lowest energy channel (black) then began to display noise in mid-2013, while at the same time the highest energy channel (grey) began to decrease precipitously, falling by nearly 3 orders of magnitude in count rate by the end of the interval. Both of these issues were suspected to be related to ion implant damage in the from MPA detector, as described in the main manuscript. Figure 3 illustrates these same issues on the MPA detector in Probe B relative to complementary measurements from the RBSPICE instrument on Probe B.

2.8 Light Contamination in the Ion Telescopes

The MPA detectors in the ion telescopes were subject to considerable light contamination when they viewed the sunlit Earth (see main manuscript). Figure 4 shows an example of this contamination in the sector-resolved level 2 data, along with the level 3 data where a mask has been applied.

2.9 Eclipse Effects

We now expand on the timing effects experienced in eclipse that were described briefly in the main manuscript. Such effects are illustrated in Figure 5 for the LOW-A unit, though all MagEIS units, including the ion telescopes, are subject to these timing issues in and around eclipse. Panel (a) shows the angular distribution (in local pitch angle) of 54 keV electron flux plotted versus time during a pass through the inner zone down towards perigee, which is at the far right of the panel. Note that once the spacecraft enters eclipse, the flux appears to change rapidly and periodically at very low pitch angle ($\alpha_L \approx 5°$). Panel (b) shows this effect in time series format for the lowest two pitch-angle bins and panel (c) shows it in an energy spectrogram in the lowest pitch-angle bin, indicating that that the effect occurs in all energy channels.

To emphasize that these effects are non-physical and related to binning and timing effects in eclipse, we also show the "unbinned" pitch-angle data, where the center of each angular sector is assigned an instantaneous pitch angle. Panel (d) shows angular distributions for two consecutive spins in eclipse using the unbinned data. The two distributions lie nearly on top of one another and are virtually indistinguishable, indicating that the angular distribution has not evolved significantly over the $\sim$22 s accumulation interval. Despite this, the binned time series shown in panels (a)-(c) at α_L=8.2° suggest that the flux changes rapidly between these two spins. This binning effect can be seen in panel (e) where a zoom in of the α_L =[0°, 35°] range is shown, which encompasses the first two pitch angle bins in the binned data. Four additional points are shown with X's, where the value of the binned flux is marked at the center of each of the two pitch angle bins. Note that the unbinned fluxes near α_L=16° straddle the bin boundary, with the flux from the first spin (07:20:10) belonging to bin 0, but the flux from the next spin (07:20:21) belonging to bin 1. The influence that this has on the binned data is clear: In the first spin, the binned flux in bin 0 is the average

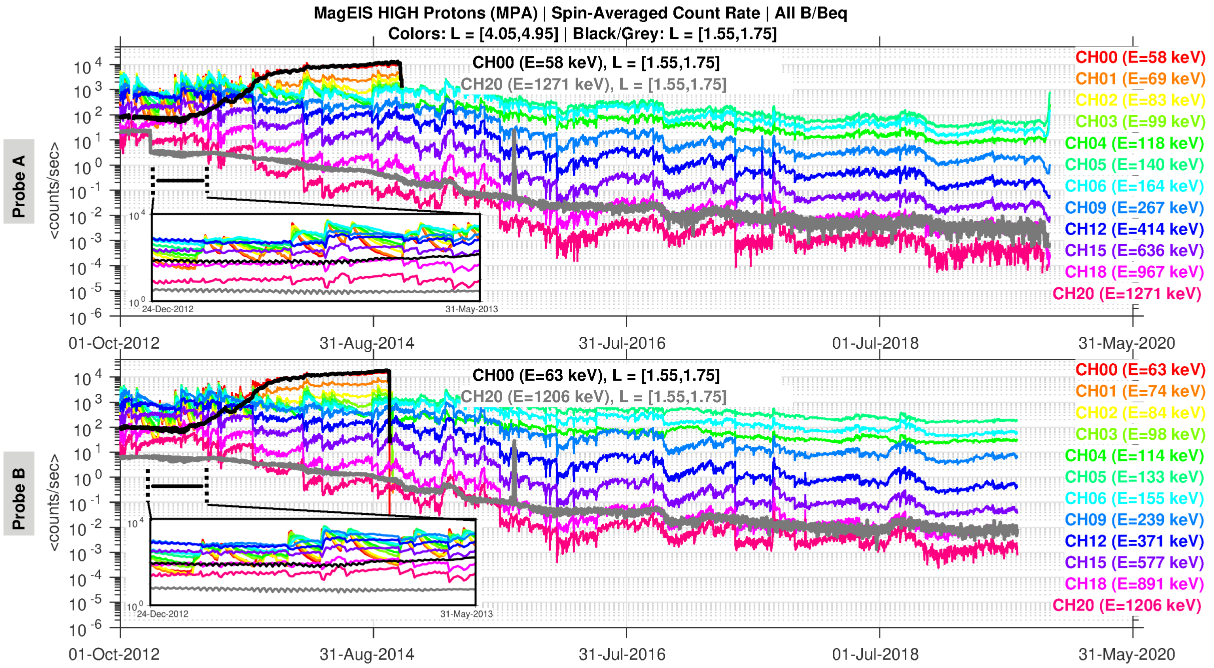

Figure 2: Development of noise and decrease in efficiency-of-detection in the MagEIS front MPA detectors in the ion telescopes on both Probes. Spin-averaged fluxes, averaged between L = 4-5, are shown in color for a subset of energy channels between ∼60 keV and ∼1.3 MeV. The black and grey traces show the lowest and highest energy channels (∼60 keV and ∼1.3 MeV) averaged in the inner zone (L = 1.5-1.8) where the measurement is dominated by background contamination. Portions of the spacecraft spin where the telescopes were subject to light contamination have been removed before the spin-averaging. The inset panels show a zoom of the early time interval when the detectors were not noisy and producing valid data. The detectors thresholds were raised in Oct 2014 to zero out the six lowest-energy channels. Note that the step-function decrease very early in the time interval in the grey line for Probe A (top) was due to a LUT change.

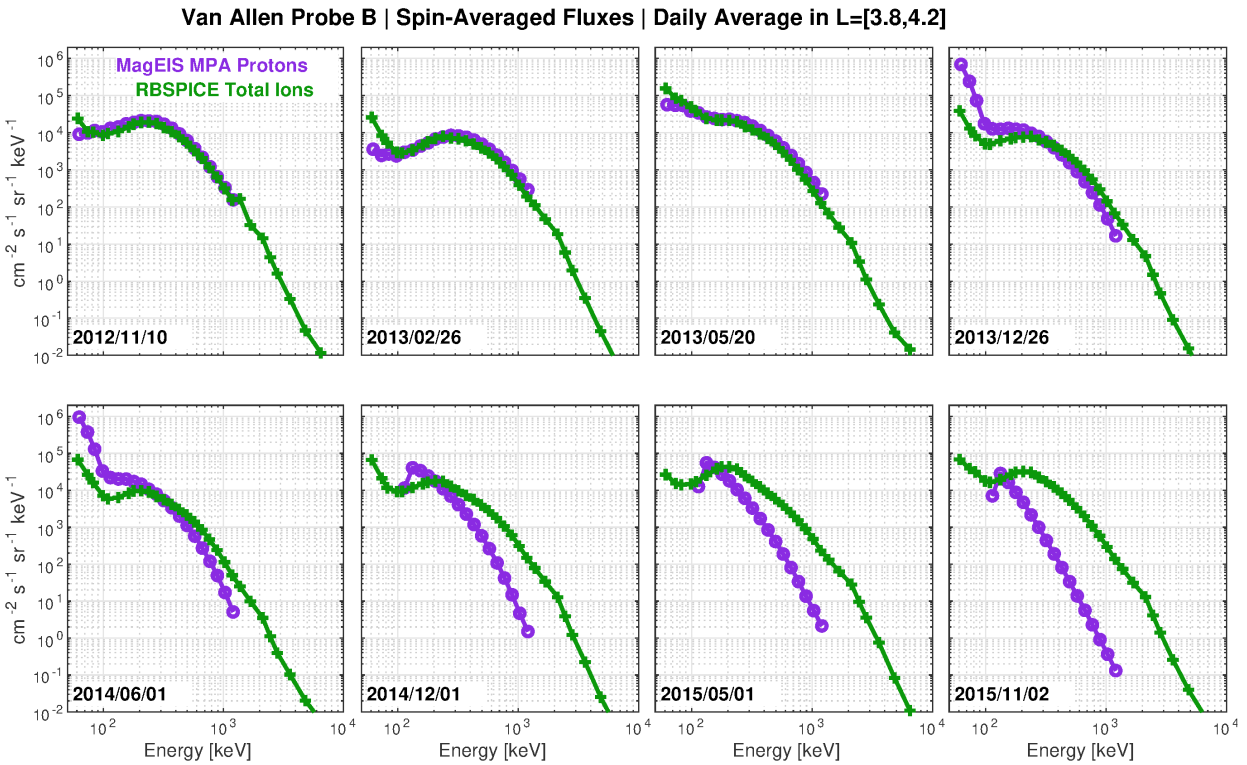

Figure 3: Development of noise and decrease in efficiency-of-detection in the MagEIS front MPA detector, here illustrated for the ion telescope on Probe B. The MagEIS data are compared with a complementary measurement from the Van Allen Probe's RBSPICE instrument. The two agreed well until late 2013 after which time considerable noise developed in the lower energy MagEIS channels; these channels were zeroed out by raising the detector threshold in Oct 2014. Note also that at the higher energies, the MagEIS fluxes were reduced relative to RBSPICE, a discrepancy that increased over time.

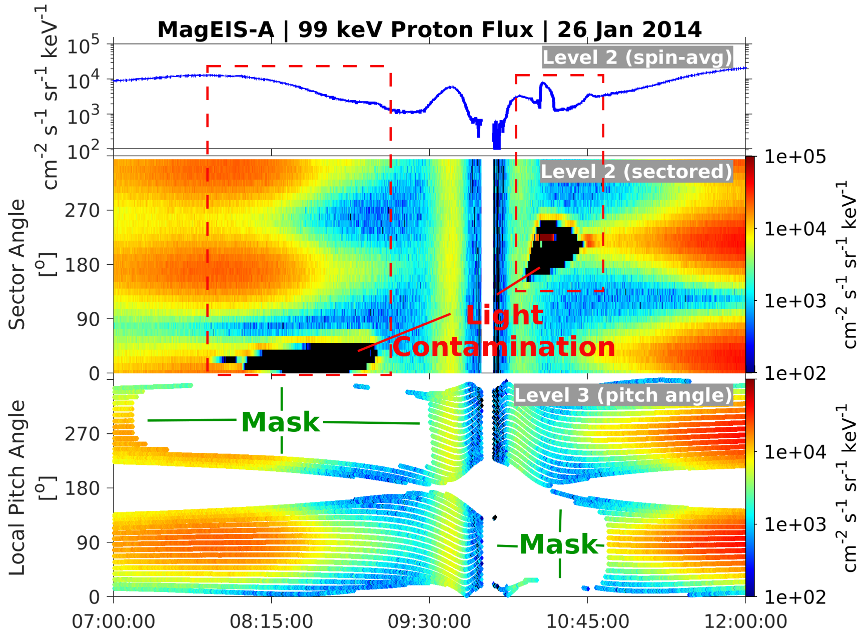

Figure 4: Example of light contamination in the 100 keV proton channel from the front (MPA) detector in the ion telescope on Probe-A. The top panel shows level 2 spin-averaged flux through perigee (∼10:00). The time interval indicated by the red boxes shows light contamination effects in the spin-averaged data. The second panel shows the level 2 angular distribution with the light contamination indicated as regions of 0 counts. The bottom panel shows the level 3 angular distribution where a mask has been applied to remove the portions of the spin that contain light contamination.

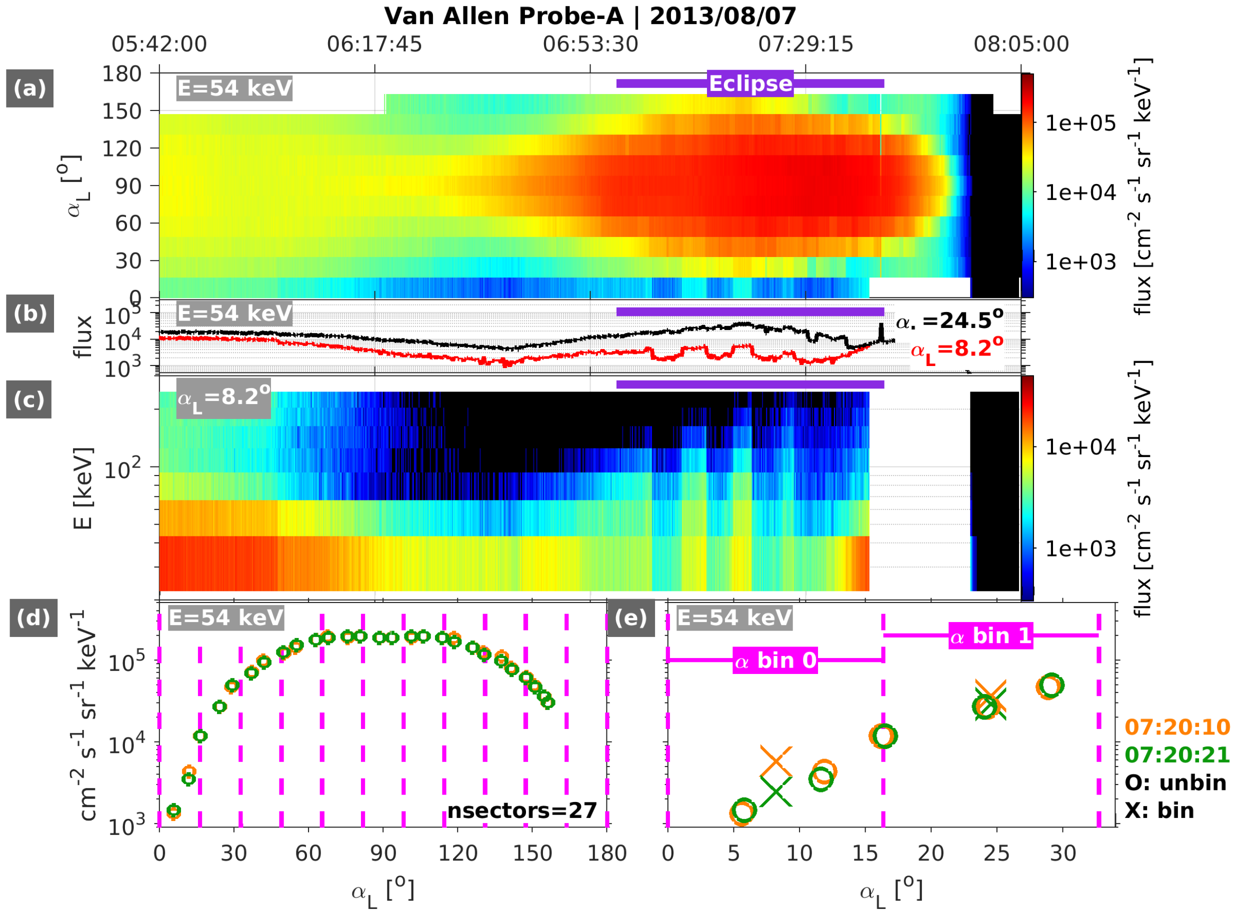

Figure 5: Van Allen Probe A pass through the inner belt down towards perigee, highlighting the impact that eclipse intervals (purple) have on the data. In panels (a)-(c), the data presented have been binned in local pitch angle (α_L). Note the rapid changes in flux that occur in the α=8.2° bin during eclipse. These are non-physical artifacts that arise due to timing errors when the spacecraft are in eclipse. Panel (d) shows unbinned angular distributions for two consecutive spacecraft spins and panel (e) shows a zoomed-in version of panel (d). In panel (e), the binned data are also shown in each bin, for comparison.

of 3 unbinned values, while in the second spin, it is the average only two binned values, hence the factor of ∼5 difference in the binned flux values in bin 0 between the two consecutive spins, despite the fact that the underlying (e.g., unbinned) angular distribution has not changed significantly between the two spins.

To mitigate this effect, end users can linearly interpolate (in logarithmic space) the unbinned data to a finer angular resolution before binning. We note that this issue is also mitigated when the MagEIS sectoring increased to larger values. The issue occurs in all pitch-angle bins, but is most apparent in the near-parallel bins ($\alpha_L \approx 0°, 180°$), since the flux gradient is usually very steep there. We note that this is a generic problem with pitch angle binning that perhaps goes unnoticed unless explicitly accounted for and corrected. It may impact the other particle measurements on the Van Allen Probes, if their angular sampling is too coarse.

A related effect occurs during eclipse exits, which are particularly prone to errors due to the abrupt change and mismatch between the true and assumed spin period. The spacecraft spins up in eclipse and the spin period decreases, while at the same time the MagEIS sensor is using an internally generated sun pulse at a fixed period. When the vehicle exits eclipse into sunlight, the MagEIS system re-syncs to the true sun pulse and the spin period rapidly returns to normal, but not immediately. This produces a short interval of changing spin periods until the vehicle settles out. Thus, the last spin before eclipse exit is ended abruptly and a new spin begins, which can produce spikes and other artifacts in flux in the times surrounding eclipse exit. This phase shift accumulation effect can be noted as "blips" in flux at eclipse exit in panels (a) and (b) in Figure 5. Van Allen Probe eclipse entrance/exit times are provided by the EMFISIS team and are available in their data archive.

2.10 Livetime Data

There are several caveats regarding the livetime data of which the end user should be aware. For example, the eclipse effects described in Sect. 2.9 can also influence the livetime values when the spacecraft enter/exit and are in eclipse. When the spin pulse re-syncs with that computed by the flight software at eclipse exit, spurious livetime values are reported (e.g., 0%, >200%). When such values are encountered in the ground data processing software, they are assumed to indicate a timing problem and fluxes are set to fill values in such instances.

Another caveat is the forced deadtime at the beginning of each spin caused by the readouts of the data from the hardware. For the LOW/MED units, there are 13 msec of deadtime at the beginning of every spin, while for HIGH it is 6 msec. For normal science mode data (e.g., main rate, histograms) this forced deadtime always occurs in the first sector for nominal values of the spin period (i.e., the minimum possible sector time is ∼172 msec = 11 sec / 64 sectors). However, for the high rate data with a large number of sectors (e.g., 1000), this deadtime can be comparable to or exceed the sector time of the first sector. In either case, it must be accounted for when using the livetime data to correct the rates. In the MagEIS data processing, this "short sector" correction is accounted for in the level 1 to level 2 processing; the livetimes reported in the level 1 data files do not account for this effect.

An additional caveat on the livetime data is an issue with the flight software when the maximum possible sectoring is used for the livetime data (64 sectors). In this case, there is rollover in the livetime counts similar to the count rollover described in Sect. 2.6 for the high-rate data when the maximum possible sectoring is used. Because the spin pulse and sectoring are accurate only to 1/4 msec, the last sector in each spin can be either longer or shorter than the true sector time, depending on the preciseness of the spin period. When this occurs, any extra livetime is written to the following sector, so that when there are 64 sectors, the counter rolls over and all extra livetime is written to sector 0 of the next spin. Compression error can also influence and enhance this effect. To mitigate this issue in the ground data processing, we replace the livetime in sector 0 with the livetime from sector 1 when 64 sectors are used.

2.11 Calibration Test Pulser Sweeps

For much of the mission, the calibration pulser in the MagEIS magnetic spectrometers was run at perigee nearly every orbit using a specialized "engineering" LUT. Perigee was chosen since, on the majority of passes, the Van Allen Probes were below the radiation belts there. Test pulser runs introduced non-physical features into the measurements taken in the ∼5 minutes around perigee when the pulser was active and thus fluxes

at/near perigee should generally be ignored. The level 1 to level 2 processing sets the fluxes to fill when the pulser is on, but there may be artifacts that are not completely removed. The test pulser calibrations were quite useful for tracking sensor performance but were used excessively in the early years. Thus, on 01 Nov 2017 the cadence of the calibration pulser operations at perigee was reduced to once per month (on the 1st orbit of the month) to allow electron data to be taken at most perigees.

3 Further Details on Data Products

3.1 Electron Histogram Data

As described in the main manuscript, the electron histogram data provided an invaluable measure of background contamination in each pixel. The figure presented in the manuscript was for M75-A histogram data. Figure 6 presents an analogous set of histogram plots for all 8 electron spectrometer units. There are several features to note. We see that P8 in the LOW/MED units does not have a "right wing" so that background corrections using the algorithm described in the main manuscript are not possible on these pixels. Similarly, P2 for the LOW/MED units has very little "left wing" for several of the units, impacting the quality of the background corrections for P2. In these cases, the background is estimated solely from the right wing level, which is generally an underestimate of the true background level in the main channel passband. We also call attention the "bump" just below the passband in P8 on both M35 units. The reason for this is unknown but was a persistent feature of the M35 units; note that such a feature is not seen in the same energy pixels on the M75.

There are two important points regarding the histogram data and how it is used in the level 1 to level 2 processing for background corrections. First, the histogram count rates provided in the level 1 files are in units of counts per second per histogram channel. To properly use these rates in processing and analysis, they must be recast in counts per second per PHA channel using the histogram LUT. Second, because both main rate LUTs and histogram LUTs were used to organize the data in-flight, it was necessary to use them together when processing the main rate and histogram simultaneously (e.g., when using the histograms to do background corrections on the main rates). Thus, the processing required a unique set of background-correction parameters (e.g., left/right wings) to be defined for each different combination of main rate and histogram LUT that was used on orbit, which complicated the processing considerably.

The histogram data also provided a high resolution data product, with energy channels much narrower ($\Delta E/E \sim 3\%$) than the main channels ($\Delta E/E \sim 30\%$). Like the main rate channels, we used the Geant4 simulations and bowtie machinery to obtain the calibration factors necessary to convert the histogram counts into fluxes. However, the narrow histogram channels complicated the conversions somewhat and stressed the limits and assumptions of the bowtie analysis.

A rough overview of the procedure used is as follows. First, for each histogram LUT used on orbit, we calculated the bowtie parameters in the same manner as described in the main manuscript for the main channels. Next, we defined a passband region for each pixel, similar to the main channel definitions (e.g., Figure 6). However, these passband regions defined for the histograms were not identical to the main channel definitions because we found it necessary in some cases to reduce the number of histogram channels selected. This is because near the edges of the passband region, small changes in the Geant4-simulated response can conspire with the steep gradients in the histogram counts on either side of the passband to produce artifacts in the histogram flux spectrum. We then calculated the bowtie parameters within this histogram passband region and converted the count rates to flux in the usual way for all histogram channels within the defined passband. Note that because we defined a separate set of passbands for the histogram data, the conversion of the histogram data to flux is independent of the main rate channel definitions/LUTs and main rate data are not used in the histogram flux conversions.

For the histogram flux data product obtained in this manner and provided in the level 2 and level 3 files, there are several caveats that should be considered:

Energy channel centroids and bounds/widths. For the bowtie-derived energy channel centroids, E_0, and channel bounds, E_{lo} and E_{hi}, there are several items of note. For example, occasionally there is a repeated E_0 in adjacent histogram channels, likely due to the resolution of the grid used in the Geant4 simulation. When this occurs, we construct a linear fit to the bowtie-derived E_0's within the passband and relabel the E_0's using the fitted channels. We note that this relabeling results in only a small change to the

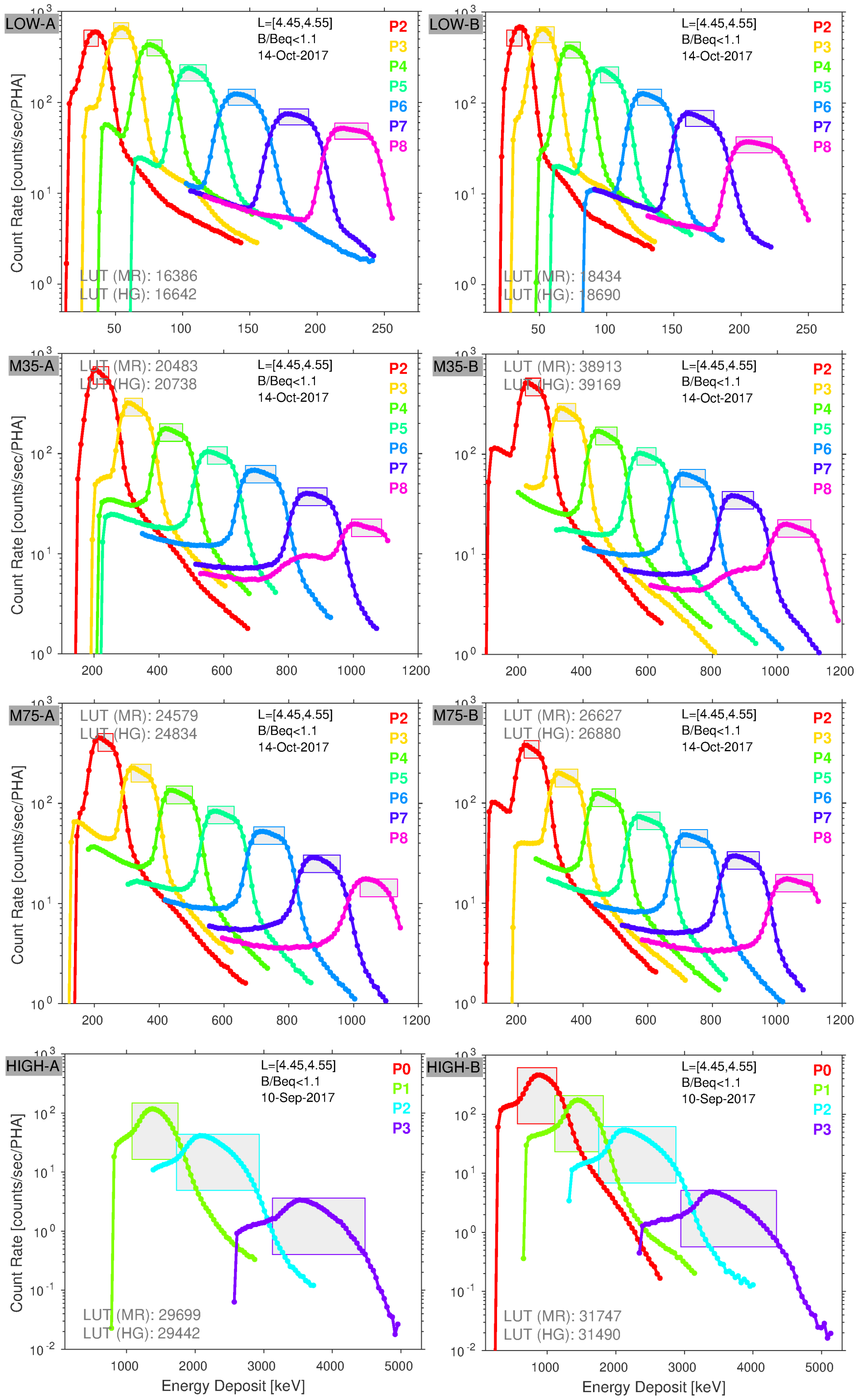

Figure 6: MagEIS histogram data from all 8 electron spectrometer units. The LOW/MED data were obtained on 14 Oct 2017 and the HIGH unit data were obtained on 10 Sep 2017. All data displayed are spin-averaged histogram count rates averaged between $L = [4.45, 4.55]$ near the magnetic equator $(B/B_{eq} < 1.1)$ over the indicated day. The main channel passband widths are shown for each pixel for the indicated LUTs (the height of the rectangles is arbitrary). Note that these main passbands are those defined by the MR LUT in PHA 0-255 space, mapped into histogram channel space using the HG LUT, and then converted to energy deposit via the gain and offset for each pixel; they are not the channel widths, ΔE, obtained from the Geant4/bowtie analysis.

E_0 values (<1%) and is done to avoid repeated energy channels. In addition, the Geant4/bowtie analysis sometimes results in E_0 values that are the same as E_{hi}. This is a feature of the narrow histogram channel response, where the large out-of-band response at higher energies "pulls" E_0 towards the higher-energy edge. An extreme case arises when the bowtie produces an E_0 that is strictly greater than E_{hi}. When this pathological case is encountered, we adjust E_{lo} and E_{hi} by setting $E_{hi} = E_0$ and then adjust E_{lo} to preserve the ΔE (full-width at half-max) obtained from the bowtie analysis. In either of these scenarios, if one desires an E_0 that lies at the center of the ΔE or one that is strictly bounded by E_{lo} and E_{hi}, then E_0 can be relabeled to the arithmetic mean, $0.5(E_{lo} + E_{hi})$, or geometric mean, $\sqrt{(E_{lo} \cdot E_{hi})}$.

Time cadence/averaging in the merged level 2 and level 3 files. As noted in the main manuscript, the histogram data were typically output at a coarser time resolution than the main rate channels. Beginning in June 2014, the histogram data were accumulated over two spacecraft spins (∼22 s) for the duration of the mission, with a coarser time resolution used prior to this time (see Table 1 in the main manuscript). An unfortunate consequence of this multi-spin accumulation for the histograms was that the histogram data from different units could be out of phase in time. Because of this, the merged level 2 and level 3 histogram flux data products are averaged into 2-min time bins. We note that there is no time averaging in the unit-by-unit level 2 and level 3 histogram data files, where the fluxes are retained at their native time cadence. This time averaging also helps in boosting the counting statistics for the histogram data, which are generally worse than the main rate channels due to the narrower energy channels.

Background corrections. The histogram data were corrected for background contamination in a manner analogous to that used for the main channels, where the procedure described in the Appendix of Claudepierre et al. (2015) was modified and tailored to the histogram data. One item of note is that we explored the influence that background subtractions have on the Geant4/bowtie-derived response since background removal is expected to change the response. This effect is also expected to be more pronounced for the narrow histogram channels and thus may result in appreciable differences in the calibration factors relative to those obtained for uncorrected data. Specifically, we examined the total response of each pixel based on the PHA channels (energy deposits) to define a background-subtracted response. We determined the incident energy passband for each pixel by calculating the isotropic response from the "hybrid" response function, integrated over all PHA channels. We then zeroed out the response everywhere outside of this passband region and computed the bowtie parameters. We found that the bowtie-derived calibration factors (e.g., E_0, G_0dE) from these "clipped" responses did not differ significantly from those obtained without accounting for background removal (the differences were typically ∼5% or less). Very often the errors from counting statistics are much larger than this and we felt it could be confusing for the end user to provide different energy channel definitions for the uncorrected and background-corrected data products. Thus, we chose to accept this level of discrepancy and used the same calibration factors for both the uncorrected and background-corrected histogram flux conversions.

Issues related to detector biasing. The histogram data were more sensitive to changes in the bias settings than the main channels. For this reason, when a unit was in the low-bias state, the histogram fluxes are not reported in the level 2 and level 3 data files (for example, between 02 Oct 2012 and 20 Dec 2012 for the M35-A unit). The time intervals when the units were in the low-bias state are provided in Table 7 and Table 8. In addition, when the M75-A unit was biased with the very high bias setting early in the mission (∼400 V - see Figure 2 in the main manuscript), the histogram data were rendered inaccurate. Thus, there are no histogram fluxes from this unit before 14 Dec 2012, after which time the bias was lowered to the nominal level of ∼200 V.

Unknown issue on HIGH-A, pixel 1. There was an unknown issue with the bowtie-derived flux conversion factors for pixel 1 on the HIGH-A unit when the histogram data were recorded with LUT 29442 prior to 02 Jul 2013. The quality of the histogram flux data from this pixel is greatly reduced prior to this time and should only be used qualitatively.

3.2 HIGH Unit Electron Direct Event Data

The MagEIS HIGH unit electron spectrometers provided additional data products beyond the standard science-mode products (main rates, livetimes, and histograms), as described in the main manuscript. The HIGH unit "direct event" data were used extensively in early operations to optimize sensor performance, especially to adjust the threshold settings (e.g., Table 1). These event data consisted of the PHA channel

registered in each count event, with a hard maximum of 8 events per sector for each sector in a spin. That is, if an event was registered within the coincidence window in both the front and rear detectors in a pixel stack, the PHA channel for that event was recorded for each detector. Due to telemetry constraints, only the last 8 events in each sector were retained, so that it is not possible to compute the true count rate (or flux) from the direct event data.

Examples of direct event data are shown in Figure 7 before and after the threshold tunings. The impact that the threshold changes had on the sensor performance is clear. For example, in the first panel in the top row (HIGH-A, P0, 2013/07/02), one can see that the front detector threshold setting was not optimal, as it "chopped out" a large portion of the pixel response. Compare this with the second panel in the top row (HIGH-A, P0, 2013/10/01) that shows data after the threshold tunings, and we see that more of the full pixel response is captured. With regard to these threshold changes shown in Table 1, we note that there was an additional threshold change on 16 Oct 2013 on P0 rear that is not listed in the table. This is because this pixel failed on 02 Oct 2013, so that the last significant threshold change to HIGH P0 was on 26 Sep 2013.

3.3 Ion Histogram Data

In addition to the ion MR LUT, an additional LUT was used to produce an ion histogram data product from the telescope detectors, providing a subsampling of the full 256 PHA channel space from each of the three detectors. For a given telescope, these histogram LUTs produced 256 histogram channels total, which were allocated between the various detectors as follows. On both telescopes, the primary histogram LUTs used on orbit mapped the 256 PHA channel space for the MPA detector into 128 ion histogram channels (2-to-1 mapping). On telescope A, the 256 PHA channel space for each of the two thin rear detectors was mapped into 64 ion histogram channels each (4-to-1 mapping), giving a total of 256 ion histogram channels from the 3 detectors. On Telescope B, the 256 PHA channels from the MSD detector were mapped into 128 ion histogram channels, again giving 256 total histogram channels. These specific ion histogram LUTs were the ones used primarily on orbit, though the histogram LUTs were changed more frequently than the main rate LUTs (i.e., additional LUTs were used that gave higher resolution to various detectors at various times). The ion histogram data have been useful in ongoing calibration efforts for the rear detectors, as they were not extensively calibrated pre-flight.

4 Calibration Factors: Energy Channel Definitions and Flux Conversion Factors

The supporting documents noted in item (1) in Sect. 1 provide tables of the calibration factors for all of the LUTs described in Sect. 2.3 (electron main rate, electron histograms, electron high rate, and ion main rate). There are a few items to note:

Electron Main Rate Data In these tables, we provide the calibration factors for the merged electron main rates. The factors shown in the tables represent the values for a fixed time interval that is defined by a unique combination of three separate MR LUTs (LOW, M75, and HIGH). The tables provided represent all of the unique combinations of LOW, M75 and HIGH unit LUTs that were used throughout the mission. There is one subtle point here. The last upload of MR LUTs to the LOW, M75, and HIGH electron spectrometers occurred on 31 Mar 2013. However, the HIGH unit instrument tunings that took place between 03 Jul 2013 and 26 Sep 2013 (Sect. 2.2) essentially resulted in a unique set of calibration factors each time a threshold setting changed. Since there were many such changes during this interval, the MagEIS team decided to set one fixed date within this interval (03 Aug 2013) as the "nominal" time for all of the changes, rather than have multiple different calibration factors within the interval. Thus, the HIGH unit electrons have a separate set of calibration factors from 31 Mar 2013 to 03 Aug 2013 despite there being no MR LUT changes after 31 Mar 2013. Thus, the HIGH unit electron calibration factors should be considered somewhat notional and approximate from 31 Mar 2013 to 26 Sep 2013, which is the date of the final change to the HIGH unit threshold settings. We also provide the analogous set of tables for the M35 unit, which was not used in the merged product.

Electron High Rate Data The energy channels and flux conversion factors for the high-rate data were obtained solely using the Geant4/bowtie analysis described in the main manuscript (these channels were

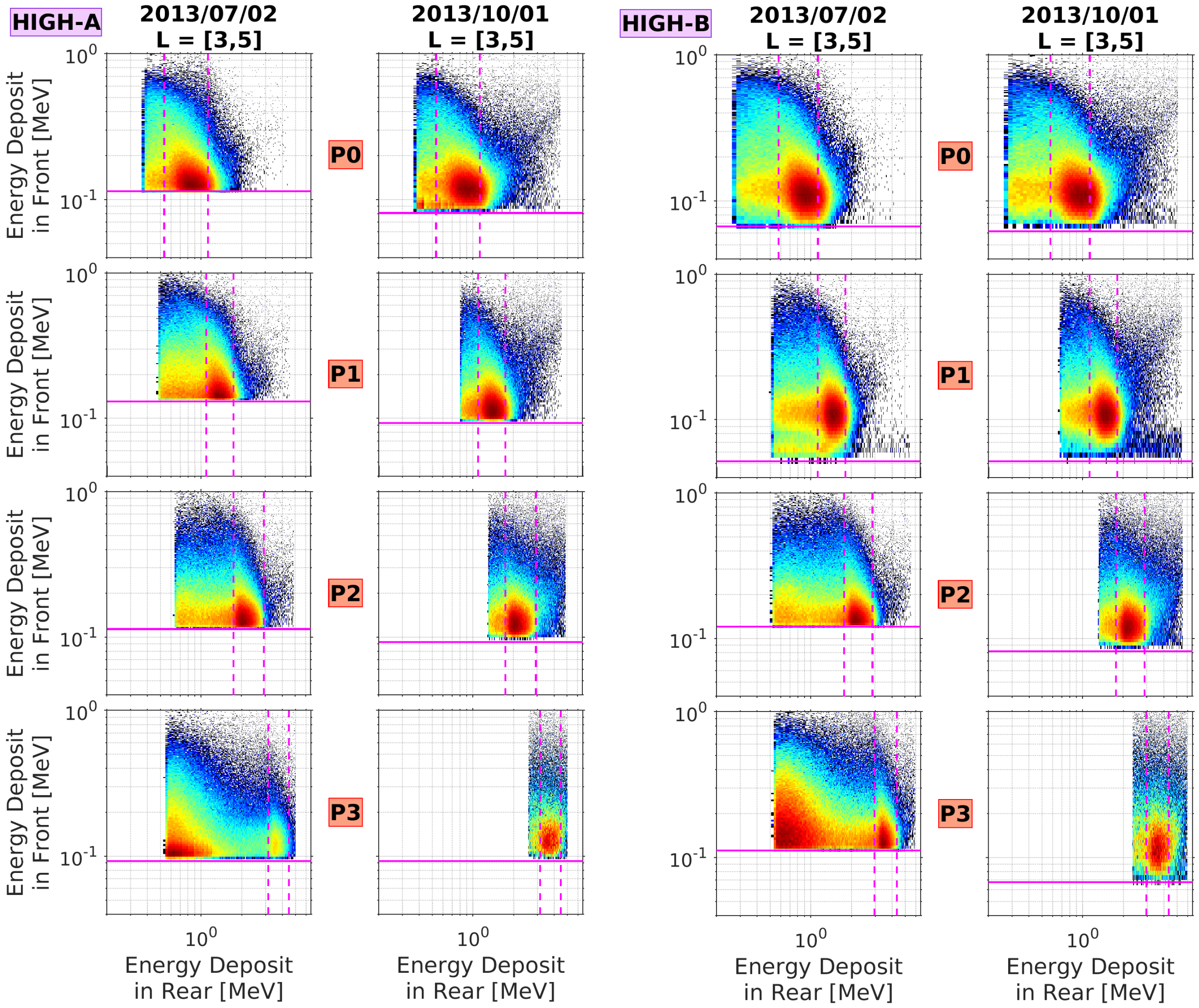

Figure 7: MagEIS HIGH unit direct event data before and after the threshold tunings. Each row corresponds to one HIGH unit pixel. The first two columns are for HIGH-A before and after the tunings, while the third and fourth columns are for HIGH-B before and after the tunings. The main channel boundaries are shown with vertical dashed magenta lines, while the front thresholds are indicated with a horizontal magenta line (rear thresholds are not indicated). We note that main channels were only defined on the rear detectors (i.e., there was no capability to define a similar main channel response region on the front detectors). The color scales are somewhat arbitrary and not shown, since the direct events can saturate during high rates. The lower value on the color scale indicates a count of 1 in a given front and rear PHA channel bin and the maximum value of the color scale is set to the maximum value within the domain.

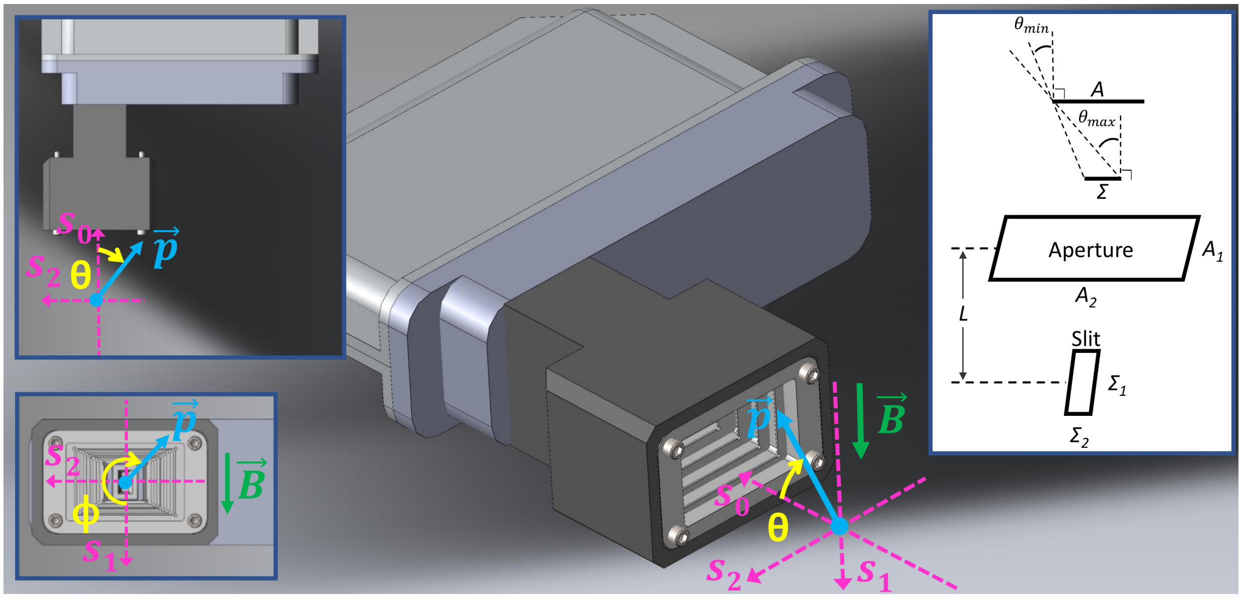

Figure 8: The sensor coordinate system, $s_0 s_1 s_2$, that is used to describe the MagEIS electron spectrometer's angular response. The main panel and two inset panels on the left define the two angles θ and ϕ (yellow) and indicate the direction of the particle's momentum vector, $\vec{p}$ (cyan), and yoke magnetic field, $\vec{B}$ (green). Note that ϕ lies in the $s_1 s_2$-plane while θ lies in the plane that contains the s_0 axis and the momentum vector. The inset panel at the right illustrates the geometry of the aperture (A) and slit (Σ) with the relevant parameters defined in Table 9. Note that in the right inset panel θ is schematically represented in a plane (e.g., when $\phi = 0°$, θ lies in the $s_0 s_1$-plane).

not calibrated pre-flight). One important caveat with respect to the HR data and LUTs is that the date on which a new LUT was uploaded (Table 6) does not necessarily mean that HR data was obtained on that date (since the instrument had to also be commanded into HR mode). Moreover, at times, we switched back and forth between the various HR LUTs over the course of the mission, to optimize performance. The end user should be aware of these changes and, if needed, consult the HR LUT ID variable in the data files to determine which LUT was used on a given day.

Electron Histogram Data Like the high rate data, the energy channels and flux conversion factors for the electron histogram data were obtained using the Geant4/bowtie analysis described in the main manuscript and in Sect. 3.1 above. The tables in the supporting PDF document provide the bowtie parameters for each histogram LUT used on orbit. The histogram LUT ID number can be found in the "LUT_ID_HG" variable in the unit-by-unit level 2 histogram data files.

Ion Main Rate Data As described in the main manuscript, Geant4 modeling of the ion telescope response was performed and flux conversion factors from bowtie analysis were computed for the front MPA detectors. However, these were not significantly different from those obtained in the pre-flight lab calibrations, so they were never implemented in the proton data processing. The tables provided thus show the energy channel definitions and flux conversion factors from the pre-flight lab calibration. Note that far fewer MR LUTs were uploaded to the ion telescopes (e.g., Table 4) compared to the electron spectrometers. During commissioning, on 29 Sep 2012, a revised set of ion main rate LUTs were uploaded to both telescopes. For telescope B, this ion main rate LUT was used for the remainder of the mission, while a second (and final) ion main rate LUT was uploaded to telescope A on 31 Mar 2013.

5 MagEIS Angular Response

Figure 8 illustrates the sensor coordinate system, $s_0 s_1 s_2$, that is used to describe the MagEIS electron spectrometer's angular response. The particle's momentum vector, $\vec{p}$, is resolved in this coordinate system

as:

$$\begin{aligned} p_0 &= p\cos\theta \\ p_1 &= p\sin\theta\cos\phi \\ p_2 &= p\sin\theta\sin\phi \end{aligned} \tag{1}$$

where the two angles, θ and ϕ, are defined as:

$$\begin{aligned} \tan\phi &= \frac{p_2}{p_1} \\ \cos\theta &= \frac{p_0}{p} \end{aligned} \tag{2}$$

and the 4-quadrant arctangent (atan2) must be used when computing ϕ. We note that as the sensor spins with the spacecraft, the direction of sweep (the direction that the boresight vector rotates in inertial space) is in the s_1 direction.

The inset panel at the right of Figure 8 illustrates the geometry of the MagEIS aperture (the entrance of the collimator) and slit (the end of the collimator). The relevant dimensions and angles are provided in Table 9 with the following definitions of θ_{min} and θ_{max}:

$$\left.\begin{aligned} \tan\theta_{max,i} &= \frac{A_i + \Sigma_i}{2L} \\ \tan\theta_{min,i} &= \frac{A_i - \Sigma_i}{2L} \end{aligned}\right\} \quad \text{for } i = 1, 2 \tag{3}$$

Here, for each of the two dimensions, θ_{min} is the largest angle where the whole slit is exposed through the collimator, and θ_{max} is the largest angle where any of it is exposed. In Table 9 and Figure 8, the subscript 2 (the s_2-direction) corresponds to the short dimension of the slit and the subscript 1 (the s_1-direction) corresponds to the slit's long dimension (and vice versa for the aperture).

Figure 9 shows a representative example of the MagEIS electron spectrometer's angular response as obtained from the Geant4 simulations described in the main manuscript. Here, we show the response for the LOW-B unit with the main channels defined by main rate LUT 18434, with the color scale indicating the effective area of that channel's response to particles arriving from each direction. The two rows of panels in the figure present the responses of main channels 2 and 8 for four incident electron energies, represented by the columns: the entire passband for the respective channel from the bowtie analysis (see main manuscript), and the energies at the lower limit, centroid, and upper limit of that passband. In each panel, the angular response is plotted on the color scale as a function of the two angles, θ and ϕ. The response is depicted in a 2D polar coordinate plot, where the ϕ angle is plotted as the azimuthal (clock) angle from 0 to 2π (clockwise sense with ϕ=0° at the bottom of the panel) and the θ angle is plotted as the radius. Two representative θ values are shown with magenta circles. In addition, two reference rectangles are shown indicating the nominal field-of-view (FOV) of the sensor, where the inner rectangle corresponds to the FOV defined by θ_{min} and the outer corresponds to that defined by θ_{max}. We note that, in some panels, apparent concentric rings may be visible and are an artifact of the binning of the simulation results. In addition, in the Geant4 simulations conducted for LOW/MED, only θ values in the range [0°, 90°] were considered, while for the HIGH unit simulations, θ values over [0°, 180°] were simulated.

The view of each panel in Figure 9 can be considered as looking into the sensor with the same perspective as in the lower-left inset in Figure 8, although the locations in each panel of Figure 9 represent directions of arrival, not positions. We emphasize that the primary response is approximately a rectangle. In such a graphical representation, a non-ideal sensor response would manifest as non-rectangular distortions or twists in the response. Also, in this representation, we note that asymmetry towards ϕ=270° indicates particles that are already moving slightly towards the pixel focal plan when they enter the collimator. The pattern of the intensities in these panels reflects two main dependencies of the angular response on the particle energy.

First, the "beam" of electrons traveling from the slit to the pixel has more room to spread out along the magnetic field for particles that make longer arcs through the magnet chamber, and so the subset of

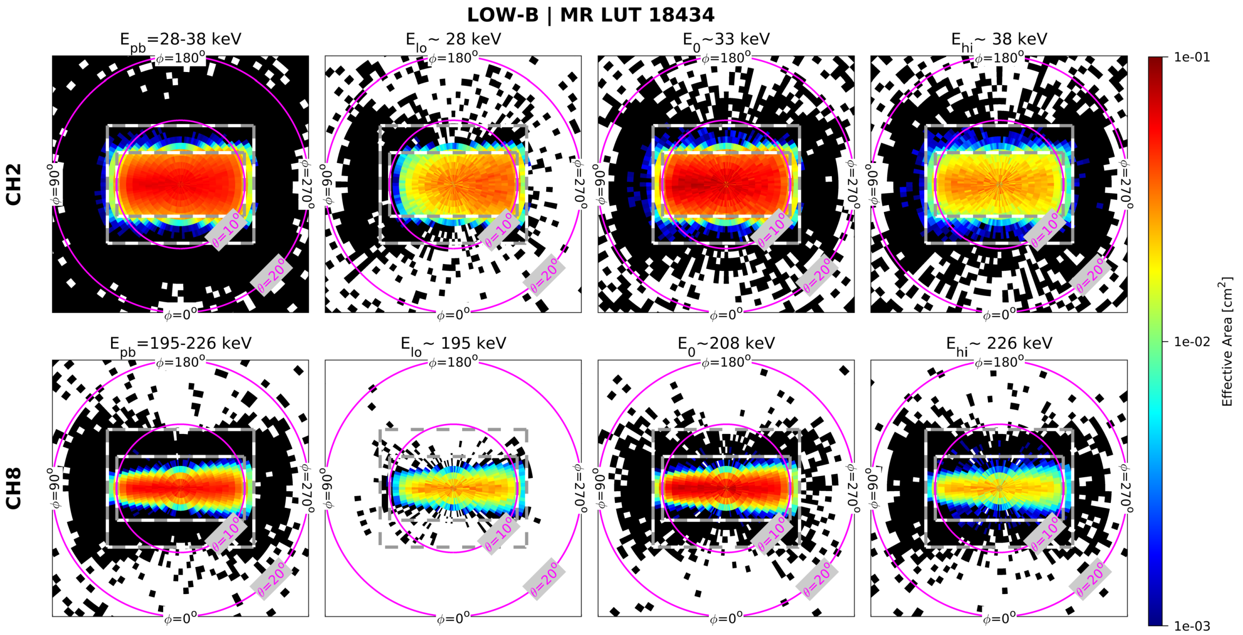

Figure 9: Effective areas for the response of two LOW-B main-rate channels (rows) as a function of electron incidence direction, for each channel's entire passband (first column) and for energies at the low end, centroid, and high end of the passband (second through fourth columns).

such particles that actually reach the detector plane rather than running into the magnets or baffles will be narrower in the s_1 direction than for particles that make shorter arcs. Since higher-energy electrons travel farther to reach the higher-numbered pixels, we can see in Figure 9 that the response for channel 8 is narrower in the vertical direction than is that for channel 2. This is the primary cause of the overall decline with energy of the response integrated over all incidence angles and plotted in Figure 13 of the main text. Likewise, at a given energy a particle arriving from a direction with ϕ between 0° and 180° will trace out a larger arc than will a particle with ϕ between 180° and 360°, going deeper into the chamber before circling back to the detector as in Figure 7 of Blake et al. (2013). Thus, again, these particles will spread out more in the direction along the magnetic field and more of them will strike the magnets or the chamber walls before reaching the detector, and therefore the response shown in each panel of Figure 9 is narrower in the vertical direction at the left of the panel, toward $\phi = 90°$, especially in the lower row of plots for channel 8.

Second, electrons of the same energy that arrive from different directions may also be imperfectly focused by the chamber magnetic field, so that they strike the detector plane at slightly different distances from the slit. The effects of this were shown by Blake et al. (2013) in their Figures 7 and 8; the Geant4 sensor models described in the main manuscript used the same realistic magnetic field configurations modeled in this earlier work using the LORENTZ magnetic-circuit code, so we see the same effects in the present simulations. Looking closely at Figure 7 of Blake et al. (2013), we see that an electron arriving from a direction with $\phi = 90°$ will strike the detector plane slightly closer to the slit than will an electron with the same energy that arrives with $\phi = 270°$. This means that, for an energy right at the boundary between the passbands of adjacent pixels, an electron with $\phi = 90°$ may arrive at the lower-numbered pixel while one with $\phi = 270°$ arrives at the higher-numbered pixel. This is seen here in the second column of Figure 9, where the response at the low-limit energy fades toward the left ($\phi = 90°$), and in the fourth column, where the response at the high-limit energy fades toward the right ($\phi = 270°$), as electrons arriving from these directions at these energies end up in an adjacent pixel. It is also the cause of the non-rectilinear shape of the energy-angle response plot in Figure 8 of Blake et al. (2013).

The details of these effects will vary from pixel to pixel and sensor to sensor, depending on the details of the geometry of the structures and the magnetic field. Figure 10 shows the same set of directional response plots as in Figure 9, but now for channels 0 and 3 of the HIGH-B unit with LUT 31747. The vertical

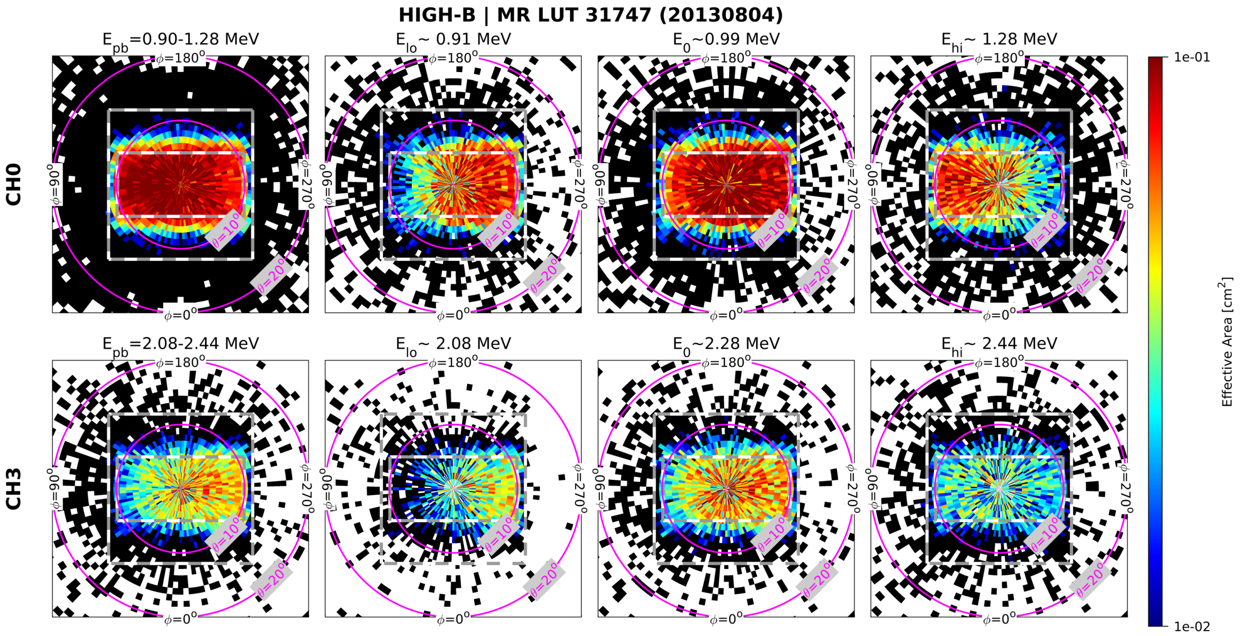

Figure 10: Same format as Figure 9 but here for two main-rate channels from the HIGH-B unit.

narrowing of response with energy is less than in Figure 9 because the chamber is wider in proportion to the length of the detector plane, but the fading toward $\phi = 90°$ of the response in the second column at the low-energy limits of the channels' passbands is more pronounced than in Figure 9. The fading of response toward $\phi = 270°$ in the fourth column at the high-energy limits is visible in the first row but not the second; this is due to the fact that channel 0 in the first row covers the whole energy range of electrons reaching pixel 0, whereas channel 3 covers only the lower part (channel 4 takes the higher part in this split pixel) of the energy range falling in pixel 2. Thus, the fourth column at the high-energy limit of channel 3 represents electrons arriving in the middle of the physical pixel, where they will not be tipped into a different pixel depending on their trajectories.

The response files provided in the Dryad data repository (link provided in the acknowledgments in the main manuscript) contain all the information that a user of the MagEIS data would need to tease out the details of the electron sensors' response, for purposes such as extraction of the detailed shape of pitch-angle distributions vs. energy via deconvolution.

6 Chamber Magnetic Field Scaling

As described in the main manuscript, a careful comparison of the flight histogram data relative to the Geant4-simulated responses revealed an offset between the two. We attributed this mismatch to a discrepancy between the nominal chamber magnetic field provided by the LORENTZ simulation and the true magnetic field within each unit. This effect is illustrated in Figure 11 for the LOW, M75, and HIGH units on Probe B. Each panel shows histogram data from the indicated pixel in grey, where the data have been averaged between $L = 3 - 5$ on the indicated day and the counts are normalized to lie between 0 and 1. The nominal simulated response is shown in red, where the isotropic response has been convolved with either an exponential (LOW-B and HIGH-B) or power law spectrum (M75-B) with the indicated parameters. These candidate spectra were determined by fitting the observed main channel spectrum within each unit's energy range (averaged over the same L region on the same day) and are thus representative of the observed spectral shape for the displayed histogram data. Comparing the nominal response (red) with the observed response (grey) reveals that there can be a mismatch between the two. For example, LOW-B (top row) pixels 2-4 show a simulated response that is shifted to the left relative to the observed response.

We corrected for this mismatch by performing additional Geant4 simulations for each unit but with the

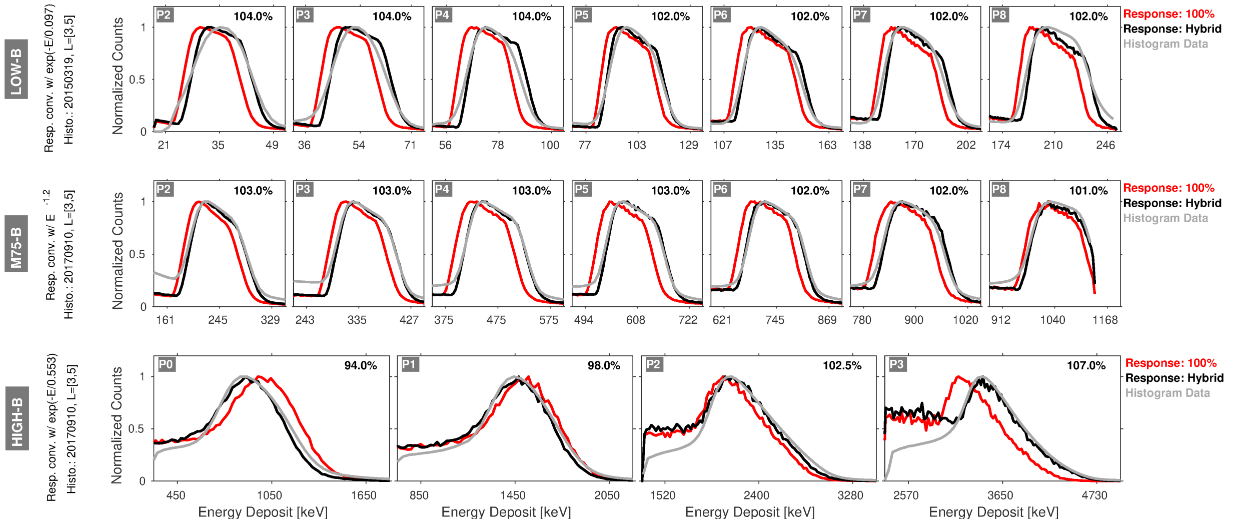

Figure 11: A comparison of histogram data (grey) with the Geant4-derived response, obtained using the nominal (unadjusted) chamber magnetic field (red) and the scaled field (black). The top row shows the LOW-B unit, the middle row shows the M75-B unit, and the bottom row shows the HIGH-B unit. The profiles in each column show normalized counts vs. energy deposit for the the indicated pixel. See text for further details.

LORENTZ-derived chamber magnetic field scaled by various percentages. The black line in each panel in Figure 11 shows the results from these simulations with the magnetic field scaling value indicated in the panel. For example, for LOW-B pixels 2-4, we see that a 104% scaling of the field is sufficient to bring the simulation results into good agreement with the observed response, while a lower field scaling is needed for the higher-energy pixels. For the M75-B unit, similar levels of scaling are required, while for the HIGH-B unit more substantial scalings are necessary (∼95-110%). We thus adopted the philosophy of choosing the optimal magnetic field scaling for each pixel in every unit, and then merging all of the pixel-specific responses together from a given unit to produce a "hybrid" response function for that unit. The magnetic field scaling values that were determined for each pixel on each unit are provided in Table 10.

We note that up through MagEIS data release 04 ("rel04"), we used a single magnetic-field scaling for all pixels in a given unit (i.e., this hybrid approach was not adopted until the final data release). The values used were as follows: LOW-A: 106.5%, LOW-B: 102%, M35-A: 100%, M35-B: 103%, M75-A: 102%, M75-B: 103%, HIGH-A: 100%, HIGH-B: 100%. For the relatively wide main channels ($\Delta E/E \sim 30\%$) and the small field-scaling corrections necessary for the LOW and MED units (see Table 10), the bowtie-derived main channel energy centroids and flux conversion factors did not change significantly when we adopted the hybrid response functions (typically by ∼5% or less). However, since we did not originally scale the HIGH unit magnetic field and since the field-scaling corrections for the HIGH unit turned out to be quite large (see Table 10), the bowtie-derived main channel energy centroids and flux conversion factors (and thus the overall flux intensities) did change somewhat between release 04 and the final MagEIS data release (∼5-10% for the energy centroids and ∼20-40% for the flux conversion factors). We emphasize that these revised main channel flux calibrations using the hybrid response functions improved the HIGH unit cross-calibration with the REPT instrument, and also the intercalibration with the MagEIS MED unit. Moreover, adopting the hybrid response functions was a crucial step in obtaining accurate flux conversions for the histogram data product, which has narrow energy channels ($\Delta E/E \sim 3\%$) and is very sensitive to the detailed profile of the simulated response. This is because, at a simplified level, converting the histogram counts to fluxes essentially amounts to dividing the histogram data (Figure 11, grey) by the simulated responses (Figure 11, red/black). When the simulated response is offset relative to the histogram data, artifacts are introduced into the histogram flux spectrum.

As a final note on this topic, Figure 12 shows the nominal chamber magnetic fields obtained from the LORENTZ simulation that were used in the Geant4 simulations. The cuts shown in each panel are through

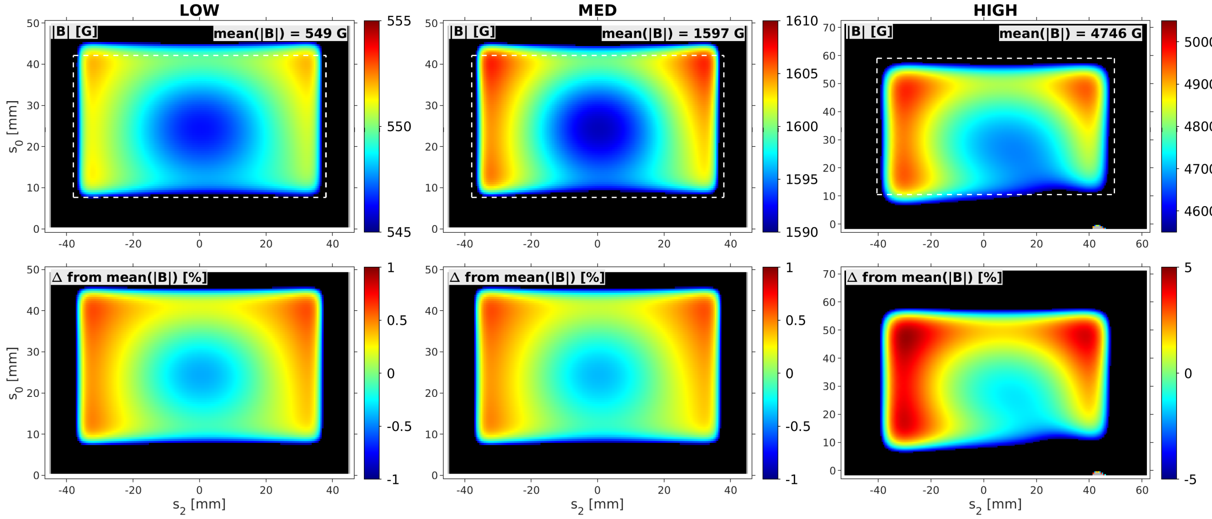

Figure 12: Cuts through the LORENTZ-simulated chamber magnetic field at the center of the chamber for each unit (columns). The top row shows the absolute field intensity while the bottom row shows the deviation from the mean field (averaged over the dashed box region indicated in the top panels). The chamber field is directed out of the page (in the s_1 direction), the instrument aperture is near the bottom right corner, and the pixel array is located near s_0=0 along the s_2 dimension (see also Figure 8).

the center of the chamber in the short dimension, s_1 (see Figure 8), which is directed out of the page and is parallel to the chamber magnetic field. The top row shows the absolute intensity of the chamber field for each unit, while the bottom row shows the percent deviation from the mean field. Here, we see that there is nonuniformity in the chamber field on the order of 1% for the LOW/MED units and $\sim$5% for the HIGH unit. Moreover, as electrons enter through the aperture (bottom right in each panel) and are deflected by the chamber magnetic field, they will sample regions with differing field intensities as they are steered onto the the pixel array (located near the s_0=0 line). Note that electrons with different incident energies will sample different regions of field intensity. These features in the LORENTZ-simulated field and the impact that they have on the energy-dependent electron trajectories through the chamber may explain why it was necessary to scale the chamber fields by the varying levels shown in Table 10.

7 Primary Variables in MagEIS Data Files

The following subsections describe the primary variables in the level 2 (L2) and level 3 (L3) MagEIS data files, both for the unit-by-unit files and for the merged (LOW + M75 + HIGH) files. There is an important caveat for the LOW/MED unit-by-unit L2 and L3 files. As described in the main manuscript, both pixels 0 and 1 on the LOW/MED electron spectrometers are noisy and should not be used, in general. However, the noisy channel from pixel 1 is retained in the unit-by-unit LOW/MED L3 data files because the channel from pixel 1 is sometimes free of noise, as described in the main manuscript (fluxes from pixel 0 are always set to fill in the L2 and L3 files). The end user should remember this caveat when using the electron fluxes from the unit-by-unit LOW/MED L2 and L3 files to ensure that they are not analyzing noisy P1 data.

In what follows, the "spin average" of a quantity J is defined as:

$$< J >= \frac{1}{N}\sum_{i=1}^{N} J_i \tag{4}$$

where N is the number of sectors in one spacecraft spin. All spin-averaged count rates, fluxes, and livetimes are computed in this way, though the error measure data products are spin-averaged in the root-mean-square sense (see main manuscript). Note that, for MagEIS data products that are accumulated over multiple

spacecraft spins (like the histogram data), computing a "spin average" via the above equation is technically an average over multiple consecutive spins (a "spin set"). Thus, in the MagEIS data files, any variables referred to as "spin averaged" should be interpreted in this context when they are accumulated over multiple spins.

7.1 Level 1 Data Files

Level 1 data files consist of the raw, uncalibrated count rates and ancillary data from the MagEIS instruments and are generally not well-suited for scientific analysis. Thus, a full description of the contents of these data files is not provided here, apart from the file naming convention:

- **File name (example):** rbspa_int_ect-mageisLOW-sp-L1_20141231_v8.0.0.cdf
- **File name (convention):** <source>_<type>_<descriptor>_<date>_<version>.cdf
  - **<source>:** 'rbspa' or 'rbspb'
  - **<type>:** 'int' or 'relNN' or 'final.' 'int' is an internally-held version of the data file that is not part of the public archive. The 'NN' in 'relNN' is the two-digit integer release number and 'final' represents the final release.
  - **<descriptor>:** Format of ect-mageisXXX-yy-L1. Here 'XXX' is the MagEIS unit (LOW, M35, M75, or HIGH) and 'yy' is the level 1 data type, which can be one of four values: 'sp' (spin-based, i.e., science data), 'ns' (non-spin based i.e., housekeeping/status data), 'hr' (high-rate data; LOW/MED only), 'de' (direct-event data; HIGH only).
  - **<date>:** UTC day in yyyymmdd format
  - **<version>:** Data version number

7.2 Level 2 Data Files

This section describes the primary data variables available in the level 2 data files. Note that some variables are only available/defined in the unit-by-unit files, as noted. The file naming convention is that same as for the level 1 files shown above, aside from the 'descriptor' field:

- **File name (example):** rbspa_int_ect-mageisM75-L2_20141231_v8.1.0.cdf
- **File name (convention):** <source>_<type>_<descriptor>_<date>_<version>.cdf
  - **<descriptor>:** Format of ect-mageisXXX-L2. Here 'XXX' is the MagEIS unit (LOW, M35, M75, or HIGH) for the unit-by-unit files and is omitted (i.e., mageis-L2) for the merged files.
  - **<source>, <type>, <date>, <version>:** Same as level 1 files

Primary data variables:

- **Epoch/Epoch_prot** Time base for the electron/proton fluxes. The epoch time corresponds to the start of a spin (i.e., the start time of the first sector in a spin).
- **FESA/FPSA** Spin-averaged electron/proton flux.
- **FEDU/FPDU** Differential, unidirectional electron/proton flux. These data are resolved by spacecraft spin-phase angle ("F*DU_Sector_Angle"), ranging from 0-360 degrees. FEDU is only available in the unit-by-unit level 2 data files (it is not in the merged level 2 file).
- **FESA_CORR** Spin-averaged electron flux, background corrected
- **FEDU_CORR** Differential, unidirectional electron flux, background corrected. These data are resolved by spacecraft spin-phase angle ("F*DU_Sector_Angle"), ranging from 0-360 degrees. FEDU_CORR is only available in the unit-by-unit level 2 data files (it is not in the merged level 2 file).
- **FEDU_Energy/FPDU_Energy** Energy channel centroids for electron/proton flux (i.e., the variables: FESA, FESA_CORR, FEDU, FEDU_CORR; FPSA, FPDU)
- **FEDU_Energy_DELTA_plus/FEDU_Energy_DELTA_minus** Upper and lower bounds for the electron energy channels FEDU_Energy. Can be used to compute the channel widths.
- **FPDU_Energy_DELTA_plus/FPDU_Energy_DELTA_minus** Upper and lower bounds for the proton energy channels FPDU_Energy. Can be used to compute the channel widths.

- **FESA_ERROR, FEDU_ERROR** Percent error (due to counting statistics) in the uncorrected electron data (spin-averaged, sector-angle resolved).
- **FPSA_ERROR, FPDU_ERROR** Percent error (due to counting statistics) in the proton data (spin-averaged, sector-angle resolved).
- **FESA_CORR_ERROR, FEDU_CORR_ERROR** Percent error (due to counting statistics and/or contamination) in the background-corrected electron data (spin-averaged, sector-angle resolved).
- **FESA_Quality/FPSA_Quality** Three-value quality flag (spin-averaged electrons/protons): 0=green, 1=yellow, 2=red. See Sect. 7.7.
- **FEDU_Quality/FPDU_Quality** Three-value quality flag (sector-angle resolved electrons/protons): 0=green, 1=yellow, 2=red. See Sect. 7.7.

The following housekeeping/status variables are only available in the unit-by-unit level 2 data files:

- **LUT_ID_MAIN** Lookup table (LUT) identifier (ID) for the main rate LUT.
- **BIAS_MODE** Flag to indicate whether or not the electron detectors are in the normal bias (bias-on) or bias-off state (0=bias off; 1=normal bias). In the bias-off state, the electron fluxes should only be used qualitatively and with an abundance of caution.
- **INSTRUMENT_MODE** Flag to indicate the mode of the MagEIS instruments (0=maintenance; 1=science; 2=high-rate). Note that only the LOW and MED units can go into high rate mode; the HIGH unit can only be in maintenance or science mode. We note that the most common reason why background corrections cannot be done is when the LOW/MED units are in high-rate mode.
- **COINCIDENCE_MODE** Flag to indicate whether or not the HIGH electron coincidence is enabled (0=disabled; 1=enabled). This variable is only valid/defined for the HIGH unit, as this is the only MagEIS unit with coincidence. In the disabled coincidence state, the electron fluxes should only be used qualitatively and with an abundance of caution.

The following variables are associated with the ion flux measurements from the two rear detectors in each of the ion telescopes. Here, “pix1” refers to the middle detector in each telescope: the 2μ detector on Probe A and the inactive ‘LP’ detector on Probe B. Similarly, “pix2” refers to the rear detector in each telescope: the 9μ detector on Probe A and the MSD detector on Probe B. At the time of writing, these measurements have not been properly calibrated:

- **FPSA_pix1, FPSA_pix2** Spin-averaged ion flux.

- **FPDU_pix1, FPDU_pix2** Differential, unidirectional ion flux. These data are resolved by spacecraft spin-phase angle (“FPDU_Sector_Angle”), ranging from 0-360 degrees.

7.3 Level 3 Data Files

This section describes the primary data variables available in the level 3 data files. Note that some variables are only available/defined in the unit-by-unit files, as noted. The file naming convention is the same as for the level 2 files above, aside from the ‘descriptor’ field where the ‘L2’ is replaced by an ‘L3.’

- **Epoch** Time base for the electron fluxes and is the start of the spin. Note that in the merged L3 files, Epoch is the coarsest time cadence of the 3 units, LOW, M75, and HIGH (the fluxes are interpolated to this common time cadence). The unit-by-unit L3 files are retained at their native time cadence.
- **Epoch_prot** Time base for the proton fluxes (unit-by-unit files) and is the start of the spin.
- **FPDU_Epoch** Time base for the proton fluxes (merged files) and is the start of the spin.
- **FEDU/FPDU** Differential, unidirectional electron/proton flux. These data are resolved into bins in local pitch angle.
- **FEDU_CORR** Differential, unidirectional electron flux, background corrected. These data are resolved into bins in local pitch angle.
- **FEDU_Energy/FPDU_Energy** Energy channel centroids for electron/proton flux (FEDU, FEDU_CORR/FPDU).
- **FEDU_Energy_DELTA_plus/FEDU_Energy_DELTA_minus** Midpoint (in log10(Energy)) between the electron energy channel centroids, FEDU_Energy. Note that these are not the upper and lower bounds for the electron energy channel centroids, as is the case in the level 2 files, and thus

they cannot be used to compute the channel widths (use the corresponding values in the level 2 files instead).

- **FPDU_Energy_DELTA_plus/FPDU_Energy_DELTA_minus** Same as previous line, for protons.
- **FEDU_ERROR,FPDU_ERROR** Percent error (due to counting statistics) in the uncorrected electron and proton flux data (pitch-angle resolved).
- **FEDU_CORR_ERROR** Percent error (due to counting statistics and/or contamination) in the background-corrected electron data (pitch-angle resolved). .
- **FEDU_Quality/FPDU_Quality** Three-value quality flag (pitch-angle resolved electrons/protons): 0=green, 1=yellow, 2=red. See Sect. 7.7.

In the unit-by-unit level 3 data files, the raw, "unbinned" pitch angle resolved fluxes are also provided, where the instantaneous sector angle is converted to pitch angle. The flux variable names are similar to the binned pitch angle variables described above (e.g., FEDU), but also include the "Unbinned" tag in the variable name. Both half-spin ("*_0to180") and full-spin ("*_0to360") flux data variables are provided. For these unbinned data, the pitch-angle value for each sector corresponds to the pitch-angle at the center of each sector, and is contained in the variable "FEDU_Unbinned_Alpha360" (full spin) and "FEDU_Unbinned_Alpha180" (half spin). These unbinned/full-spin pitch-angle data are useful if one wishes to construct one's own pitch-angle binning, examine non-gyrotropic effects, etc. Note that the unbinned pitch angle data is time tagged with Epoch, which not entirely consistent. Epoch is the start of the spin but the unbinned pitch angle variable is the pitch angle at the center of each sector (i.e., the unbinned data should really be time tagged with Epoch plus half the sector time).

Also, as in the level 2 data files, in the level 3 files there are variables associated with the ion flux measurements from the two rear detectors in each of the ion telescopes (i.e., FPDU_pix1, FPDU_pix2; see above).

7.4 Level 2 High Rate (HR) Data Files

This section describes the primary data variables available in the level 2 high-rate data files. Note that there is no merged high-rate data product, only unit-by-unit files. The file naming convention is the same as the level 1 files above, aside from the 'descriptor' field:

- **File name (example):** rbspa_int_ect-mageisLOW-hr-L2_20141231_v8.0.0.cdf
- **File name (convention):** <source>_<type>_<descriptor>_<date>_<version>.cdf
  - **<descriptor>:** Format of ect-mageisXXX-hr-L2. Here 'XXX' is the MagEIS unit (LOW, M35, or M75).
  - **<source>, <type>, <date>, <version>:** Same as level 1 files

Primary data variables:

- **HighRate** Differential, unidirectional electron flux. These data are resolved by energy channel and spacecraft spin-phase angle ("FEDU_Sector_Angle"). The array dimensions are ntime x 2048 x 8 (e.g., ntime x nsectors_max x nchannels_max). If fewer than 8 channels are recorded, then the fluxes from the unused channels are set to fill value. If fewer than 2048 sectors are recorded, then the fluxes from the unused sectors are set to fill value.
- **FEDU_Sector_Angle** Instantaneous, spin-phase (sector) angle, 0-360 degrees. The angle is the value at the start of the sector. There are 2048 possible HR sectors but typically fewer than 2048 sectors are recorded (e.g., 500, 1000, or 2000). The array dimensions are ntime x 2048 so that, for a fixed time index, the first value corresponds to sector angle at the start of the first sector in the spin, the second value corresponds to sector angle at the start of the second sector in the spin, and so on.
- **FEDU_Energy** Energy channel centroids for electron flux (HighRate). There are 8 possible HR channels but typically fewer than 8 channels are recorded (e.g., 3, 5, or 7).
- **FEDU_Energy_DELTA_plus/FEDU_Energy_DELTA_minus** Upper and lower bounds for the electron energy channels FEDU_Energy. Can be used to compute the channel widths.
- **HighRate_ERROR** Percent error (due to counting statistics) in the HighRate electron flux data.
- **LUT_ID_HighRate** Lookup table (LUT) identifier (ID) for the high rate LUT.

7.5 Level 3 Highrate (HR) Data Files

This section describes the primary data variables available in the level 3 high-rate data files. Note that there is no merged high-rate data product, only unit-by-unit files. The file naming convention is the same as in the level 2 high-rate files above, aside from the 'descriptor' field where the 'L2' is replaced by an 'L3.'

- **HighRate** Differential, unidirectional electron flux. These data are resolved by energy channel and local pitch angle in each angular sector. The array dimensions are ntime x 2048 x 8 (e.g., ntime x nsectors_max x nchannels_max). If less than 8 channels are recorded, then the fluxes from the unused channels are set to fill value. If less than 2048 sectors are recorded, then the fluxes from the unused sectors are set to fill value.
- **HighRate_Alpha360** Instantaneous, full-spin (0-360 degrees), local pitch angle for each sector. There are 2048 possible HR sectors but typically fewer than 2048 sectors are recorded (e.g., 500, 1000, or 2000). The pitch angle is given at the center of each angular sector. The array dimensions are ntime x 2048 so that, for a fixed time index, the first value corresponds to pitch angle at the center of the first sector in the spin, the second value corresponds to pitch angle at the center of the second sector in the spin, and so on.
- **FEDU_Energy** Energy channel centroids for electron flux (HighRate). There are 8 possible HR channels but typically fewer than 8 channels are recorded (e.g., 3, 5, or 7).
- **HighRate_ERROR** Percent error (due to counting statistics) in the HighRate electron flux data.

7.6 Level 2 and Level 3 Histogram (HG) Data Files

The high-energy-resolution electron histogram fluxes are provided in the level 2 (L2) and level 3 (L3) HG data files. Their contents/variables are nearly identical to the regular (main rate channel) L2 and L3 files (Sect. 7.2 and Sect. 7.3) and so, for brevity, are not described in detail here. Like the regular L2 and L3 files, there are both unit-by-unit and merged L2 and L3 files. For the unit-by-unit HG files, the file naming convention follows the format used above for the high rate data files, but with the "hr" tag replaced with an "hg." For the merged HG files, the descriptor field is in the format of "ect-mageis-hg-L2." The important differences relative to the regular L2 and L3 are as follows:

- The energy dimension is larger in the HG files to accommodate the increased energy resolution.
- The maximum number of sectors is 32 in the HG files vs. 64 in the regular files.
- In the merged L2 HG files, the fluxes are time-averaged on a 2-minute time cadence and the Epoch time is the center of the time bin. Note that this is only the case for the merged L2 HG files; the unit-by-unit L2 HG files contain the data at the native histogram time cadence (see Table 1 in the main manuscript) where the Epoch variable is the beginning of the accumulation period (i.e., the start of a block of NSPINS spins). This time averaging in the merged L2 HG files is done to improve counting statistics and to provide a uniform time base for the histogram data combined from the 3 units, LOW+M75+HIGH, since there is no guarantee that the individual unit-by-unit L2 HG files are all on the same time cadence (histograms are accumulated over multiple spins and units can be out-of-phase with one another in time).
- In the merged L3 HG files, the fluxes are interpolated to a common time cadence, the coarsest time cadence of the 3 units, LOW, M75, and HIGH. The unit-by-unit L3 HG files are retained at their native time cadence and the Epoch variable is the start of a block of NSPINS spins. To compute pitch angles in the unit-by-unit files, the magnetic field at the time consistent with the central spin of the NSPINS-spin block is used.
- Data quality flag variables (e.g., FESA_Quality) an some of the other support variables are not included in the HG files. The end user can examine the data quality flags in the regular L2 files as a proxy. Note, however, that errors from counting statistics (i.e., FESA_ERROR) and background contamination (i.e., FESA_CORR_ERROR) are provided in the HG files.

7.7 Data Quality Flags

Data quality flags are provided in the regular level 2 and level 3 data files (i.e., not in the high rate or histogram data files). The data quality flags provide a simple flag that can take on one of three possible

values (i.e., a red, yellow, green flag):

0. (green): There are no known issues with the data.

1. (yellow): The data should be used with caution. Examples include: background corrections could not be performed (electrons only); low counts, large backgrounds (electrons only), or both; detector coincidence was disabled (HIGH electrons only); deadtime correction was not performed; calibration tunings.

2. (red): The data are highly suspect and should be used with extreme care. Examples include: very low counts, very large backgrounds (electrons only), or both; detector bias was disabled (i.e., in the bias-off state); large deadtimes (>40%); noisy channels.

These flag values are first determined on a sector-by-sector basis in each spin. The spin-averaged quality flag is then determined by taking the most frequently occurring value of the sectored quality flag in a given spin. For example, if there are 4 sectors per spin with corresponding flag values = [0,0,1,2], then spin-averaged quality flag is 0 for that spin.

Table 1: HIGH Electron Spectrometer Coincidence/Threshold Changes, Testing Intervals, and Thermal Oscillation (TO) Noise Intervals

	Date	Unit	Type
Coinc./Thresh. Changes	24 Oct 2012	HIGH-A, -B	Coincidence window: 20 μs to 10 μs
	21 Dec 2012	HIGH-A	Coincidence window: 10 μs to 20 μs
	22 Dec 2012	HIGH-B	Coincidence window: 10 μs to 20 μs
	31 Mar 2013	HIGH-A, -B	Coincidence window: 20 μs to 10 μs
	04 Apr 2013	HIGH-B	Coincidence window: 10 μs to 5 μs
	03 Jul 2013	HIGH-A, -B	Threshold changes
	18 Jul 2013	HIGH-A, -B	Threshold changes
	25 Jul 2013	HIGH-B	Threshold changes
	26 Jul 2013	HIGH-A	Threshold changes
	03 Aug 2013	HIGH-A, -B	Threshold changes
	14 Aug 2013	HIGH-A	Threshold changes
	14 Aug 2013	HIGH-A	Coincidence window: 10 μs to 5 μs
	21 Aug 2013	HIGH-A	Threshold changes
	26 Sep 2013	HIGH-A	Threshold changes
Testing	23 May 2014	HIGH-A, -B	Coincidence testing; turned off until 5/28
	28 May 2014	HIGH-A, -B	Coincidence turned back on
	01 Jun 2014	HIGH-A, -B	Coincidence testing
	04 Jun 2014	HIGH-A	Coincidence testing
	05 Jun 2014	HIGH-A, -B	Coincidence testing
Thermal Oscillation Noise	29 May 2014	HIGH-A	Incorrect P0 threshold $\rightarrow$ cross-talk noise in all pixels until corrected on 5/31
	29 May 2014	HIGH-B	Incorrect heater set point $\rightarrow$ TO in P3 until 9/18[a]
	31 May 2014	HIGH-A	Correct thresholds restored
	06 Aug 2014	HIGH-A	TO noise in P1. Not corrected until 11/07[b]
	18 Sep 2014	HIGH-B	Incorrect heater set point corrected
	07 Nov 2014	HIGH-A	Heater set point lowered. Corrected TO noise in P1
	11 Mar 2016	HIGH-B	Heater set point lowered (preemptive)
	18 Mar 2016	HIGH-B	Heater set point lowered (preemptive)
	26 Nov 2016	HIGH-A	TO noise in P1. Not corrected until 12/06[c]
	06 Dec 2016	HIGH-A	Heater set point lowered. Corrected TO noise in P1
	04 Oct 2017	HIGH-A	TO noise in P1. Not corrected until 10/12[d]
	12 Oct 2017	HIGH-A	Heater set point lowered. Corrected TO noise in P1
	27 Dec 2017	HIGH-A	TO noise in P1. Not corrected until 01/03[e]
	03 Jan 2018	HIGH-A	Heater set point lowered. Corrected TO noise in P1
	24 Mar 2018	HIGH-A	TO noise in P1. Not corrected until 04/11[f]
	11 Apr 2018	HIGH-A	Heater set point lowered. Corrected TO noise in P1

[a]HIGH-B P3 data is set to fill value from 29 May 2014 to 18 Sep 2014
[b]HIGH-A P1 data is set to fill value from 06 Aug 2014 to 07 Nov 2014
[c]HIGH-A P1 data is set to fill value from 26 Nov 2016 to 06 Dec 2016
[d]HIGH-A P1 data is set to fill value from 04 Oct 2017 to 12 Oct 2017
[e]HIGH-A P1 data is set to fill value from 27 Dec 2017 to 03 Jan 2018
[f]HIGH-A P1 data is set to fill value from 24 Mar 2018 to 11 Apr 2018

Table 2: Dates and times of LUT table changes for the electron main rates.

Time	Unit-Probe	LUT ID
2012/09/25 05:30:00	HIGH-A	29696>29697
2012/09/25 05:30:00	M35-A	20481>20482
2012/09/28 15:42:00	HIGH-B	31744>31745
2012/09/28 15:42:00	M75-B	25600>25601
2012/10/04 16:20:00	HIGH-B	31745>31746
2012/10/04 19:00:00	HIGH-A	29697>29698
2012/10/04 18:55:00	M75-A	24577>24578
2012/10/17 00:00:00	LOW-B	17408>18433
2012/10/24 00:00:00	M75-B	25601>26626
2013/02/24 00:00:00	M75-A	24578>24577
2013/02/24 00:00:00	M75-B	26626>25601
2013/03/31 00:00:00	LOW-A	16385>16386
2013/03/31 00:00:00	LOW-B	18433>18434
2013/03/31 00:00:00	M35-A	20482>38913[a]
2013/03/31 00:00:00	M75-A	24577>24579
2013/03/31 00:00:00	M75-B	25601>26627
2013/03/31 00:00:00	HIGH-A	29698>29699
2013/03/31 00:00:00	HIGH-B	31746>31747
2013/04/03 00:00:00	M35-A	38913>20483
2013/04/03 00:00:00	M35-B	38912>38913

[a] 38913 was a M35-B LUT inadvertently uploaded to M35-A (corrected on 2013/04/03).

Table 3: Dates and times of LUT table changes for the electron histograms.

Time	Unit-Probe	LUT ID
2012/09/19 21:35:00	HIGH-A	29441>ENG
2012/09/21 01:12:00	HIGH-B	31489>ENG
2012/10/02 17:29:00	LOW-A	16641>16642
2012/10/02 17:29:00	M35-A	20737>20738
2012/10/02 17:29:00	M75-A	24833>24834
2012/10/02 17:57:00	LOW-B	17664>18689
2012/10/02 17:57:00	M35-B	39168>39169
2012/10/02 17:57:00	M75-B	25856>26880
2012/10/04 19:00:00	LOW-A	16642>16641
2012/10/04 19:00:00	M35-A	20738>20737
2012/10/04 19:00:00	M75-A	24834>24833
2012/10/04 16:20:00	LOW-B	18689>17664
2012/10/04 16:20:00	M35-B	39169>39168
2012/10/04 16:20:00	M75-B	26880>25856
2012/10/04 19:00:00	HIGH-A	ENG>29442
2012/10/04 16:20:00	HIGH-B	ENG>31490
2012/10/18 00:00:00	M75-A	24833>24834
2012/10/18 06:40:00	M75-B	25856>26880
2012/10/18 00:30:00	M35-A	20737>20738
2012/10/18 06:40:00	M35-B	39168>39169
2012/10/25 03:20:00	LOW-A	16641>16642
2012/10/25 03:20:00	LOW-B	17664>18690

Table 4: Dates and times of LUT table changes for the ion main rates.

Time	Unit-Probe	LUT ID
2012/09/28 15:42:00	HIGH-B	32000>32001
2012/12/23 00:00:00	HIGH-A	29952>29953
2013/03/31 00:00:00	HIGH-A	29953>29954

Table 5: Dates and times of LUT table changes for the ion histograms.

Time	Unit-Probe	LUT ID
2012/09/28 15:42:00	HIGH-B	32257>ENG
2012/11/28 00:00:00	HIGH-B	32257>32258
2013/02/13 00:00:00	HIGH-A	30208>30209
2013/04/04 00:00:00	HIGH-A	30209>30210
2013/07/24 00:00:00	HIGH-A	30210>30211
2013/09/26 00:00:00	HIGH-A	30211>30209

Table 6: Dates and times of LUT table changes for the electron high rates.

Time	Unit-Probe	LUT ID
2012/10/04 19:00:00	M75-A	NONE>25088
2012/10/09 06:30:00	LOW-B	ENG>18944
2012/10/09 06:30:00	M75-B	ENG>27136
2012/10/09 11:10:00	LOW-A	ENG>16896
2012/10/09 11:10:00	M75-A	ENG>25089
2012/12/23 00:00:00	LOW-A	16896>16897
2012/12/23 00:00:00	LOW-B	18944>18945
2012/12/23 00:00:00	M35-A	NONE>20992
2012/12/23 00:00:00	M75-B	27136>27137
2013/02/24 00:00:00	M75-A	25089>25088
2013/02/24 00:00:00	M75-B	27137>27136
2013/04/04 00:00:00	LOW-A	16897>16898
2013/04/04 00:00:00	LOW-B	18945>18946
2013/04/04 00:00:00	M35-A	20992>20993
2013/04/04 00:00:00	M35-B	39424>39425
2013/04/04 00:00:00	M75-A	25088>25090
2013/04/04 00:00:00	M75-B	27136>27138
2013/07/12 00:00:00	LOW-B	18946>18947
2013/07/12 00:00:00	LOW-A	16898>16899
2013/07/13 00:00:00	M75-B	27138>27139
2013/07/14 00:00:00	M75-A	25090>25091
2015/09/16 00:00:00	M35-A	20993>20994
2015/09/16 00:00:00	M35-B	39425>39426
2017/07/31 00:00:00	M75-A	25091>25092
2017/07/31 00:00:00	M75-B	27139>27140

Table 7: Start and stop times for the low-bias state, Probe A. The times indicated are accurate to 5 min. Nearly all of these instances fall into 3 categories (1) Instrument turn-on in early Sep 2012; (2) Instrument reboots (e.g., for LUT uploads); (3) Instrument anomaly investigations.

Unit	Start UTC	Stop UTC	Duration
LOW-A	06-Sep-2012 17:17:30	06-Sep-2012 17:47:30	30 min
LOW-A	07-Sep-2012 20:47:30	11-Sep-2012 22:57:30	4.1 day
LOW-A	17-Sep-2012 22:27:30	17-Sep-2012 22:32:30	< 5 min
LOW-A	02-Oct-2012 17:52:30	02-Oct-2012 17:57:30	< 5 min
LOW-A	07-Oct-2012 04:02:30	07-Oct-2012 04:07:30	< 5 min
LOW-A	20-Dec-2012 16:22:30	20-Dec-2012 16:27:30	< 5 min
LOW-A	23-Dec-2012 00:02:30	23-Dec-2012 00:07:30	< 5 min
LOW-A	31-Mar-2013 00:02:30	31-Mar-2013 00:07:30	< 5 min
LOW-A	04-Apr-2013 00:02:30	04-Apr-2013 00:07:30	< 5 min
LOW-A	19-Sep-2017 15:32:30	19-Sep-2017 23:57:30	8.4 hr
LOW-A	22-Sep-2017 00:02:30	26-Sep-2017 18:07:30	4.8 day
M35-A	06-Sep-2012 17:17:30	06-Sep-2012 17:57:30	40 min
M35-A	07-Sep-2012 21:07:30	11-Sep-2012 05:47:30	3.4 day
M35-A	17-Sep-2012 22:47:30	17-Sep-2012 22:57:30	10 min
M35-A	25-Sep-2012 05:27:30	28-Sep-2012 10:02:30	3.2 day
M35-A	02-Oct-2012 18:02:30	20-Dec-2012 16:42:30	78.9 day
M35-A	23-Dec-2012 00:02:30	23-Dec-2012 00:07:30	< 5 min
M35-A	31-Mar-2013 00:02:30	31-Mar-2013 00:07:30	< 5 min
M35-A	03-Apr-2013 00:02:30	03-Apr-2013 00:07:30	< 5 min
M35-A	04-Apr-2013 00:02:30	04-Apr-2013 00:07:30	< 5 min
M75-A	06-Sep-2012 17:17:30	11-Sep-2012 05:47:30	4.5 day
M75-A	11-Sep-2012 23:12:30	11-Sep-2012 23:17:30	< 5 min
M75-A	17-Sep-2012 22:47:30	17-Sep-2012 22:57:30	10 min
M75-A	28-Sep-2012 19:02:30	28-Sep-2012 19:07:30	< 5 min
M75-A	02-Oct-2012 18:07:30	02-Oct-2012 18:12:30	< 5 min
M75-A	04-Oct-2012 19:02:30	04-Oct-2012 19:07:30	< 5 min
M75-A	12-Oct-2012 11:27:30	16-Oct-2012 04:02:30	3.7 day
M75-A	18-Oct-2012 00:02:30	18-Oct-2012 00:12:30	10 min
M75-A	14-Dec-2012 14:52:30	14-Dec-2012 14:57:30	< 5 min
M75-A	24-Feb-2013 00:02:30	24-Feb-2013 00:07:30	< 5 min
HIGH-A	06-Sep-2012 17:22:30	06-Sep-2012 18:17:30	55 min
HIGH-A	07-Sep-2012 21:17:30	11-Sep-2012 23:17:30	4.1 day
HIGH-A	17-Sep-2012 22:52:30	18-Sep-2012 11:57:30	13.1 hr
HIGH-A	19-Sep-2012 21:57:30	19-Sep-2012 22:02:30	< 5 min
HIGH-A	25-Sep-2012 05:27:30	28-Sep-2012 19:47:30	3.6 day
HIGH-A	04-Oct-2012 18:47:30	04-Oct-2012 18:52:30	< 5 min
HIGH-A	25-Oct-2012 03:22:30	25-Oct-2012 23:57:30	20.6 hr
HIGH-A	20-Dec-2012 16:52:30	20-Dec-2012 17:02:30	10 min
HIGH-A	23-Dec-2012 00:02:30	23-Dec-2012 00:07:30	< 5 min
HIGH-A	13-Feb-2013 00:02:30	13-Feb-2013 00:07:30	< 5 min
HIGH-A	31-Mar-2013 00:02:30	31-Mar-2013 00:07:30	< 5 min
HIGH-A	04-Apr-2013 00:02:30	04-Apr-2013 00:07:30	< 5 min
HIGH-A	24-Jul-2013 00:02:30	24-Jul-2013 00:07:30	< 5 min
HIGH-A	26-Sep-2013 00:02:30	26-Sep-2013 00:07:30	< 5 min
HIGH-A	11-Oct-2013 22:12:30	11-Oct-2013 23:57:30	1.7 hr
HIGH-A	16-Oct-2013 19:17:30	16-Oct-2013 23:57:30	4.7 hr
HIGH-A	29-May-2014 00:02:30	29-May-2014 00:07:30	< 5 min

Table 8: Start and stop times for the low-bias state, Probe B. The times indicated are accurate to 5 min. Nearly all of these instances fall into 3 categories (1) Instrument turn-on in early Sep 2012; (2) Instrument reboots (e.g., for LUT uploads); (3) Instrument anomaly investigations.

Unit	Start UTC	Stop UTC	Duration
LOW-B	06-Sep-2012 18:37:30	11-Sep-2012 10:57:30	4.7 day
LOW-B	12-Sep-2012 00:57:30	12-Sep-2012 01:02:30	$<$ 5 min
LOW-B	18-Sep-2012 01:47:30	18-Sep-2012 01:52:30	$<$ 5 min
LOW-B	02-Oct-2012 18:37:30	02-Oct-2012 18:42:30	$<$ 5 min
LOW-B	17-Oct-2012 00:02:30	17-Oct-2012 00:07:30	$<$ 5 min
LOW-B	21-Dec-2012 15:22:30	21-Dec-2012 15:27:30	$<$ 5 min
LOW-B	23-Dec-2012 00:02:30	23-Dec-2012 00:07:30	$<$ 5 min
LOW-B	31-Mar-2013 00:02:30	31-Mar-2013 00:07:30	$<$ 5 min
LOW-B	04-Apr-2013 00:02:30	04-Apr-2013 00:07:30	$<$ 5 min
M35-B	06-Sep-2012 18:37:30	06-Sep-2012 18:57:30	20 min
M35-B	07-Sep-2012 19:52:30	11-Sep-2012 10:57:30	3.6 day
M35-B	12-Sep-2012 01:02:30	18-Sep-2012 01:57:30	6.0 day
M35-B	18-Sep-2012 02:02:30	18-Sep-2012 02:07:30	$<$ 5 min
M35-B	02-Oct-2012 18:42:30	02-Oct-2012 18:47:30	$<$ 5 min
M35-B	12-Oct-2012 06:57:30	21-Dec-2012 15:42:30	70.4 day
M35-B	03-Apr-2013 00:02:30	29-May-2013 00:37:30	56.0 day
M35-B	23-Jul-2015 19:12:30	23-Jul-2015 23:57:30	4.7 hr
M75-B	06-Sep-2012 18:37:30	06-Sep-2012 19:07:30	30 min
M75-B	07-Sep-2012 21:47:30	11-Sep-2012 11:02:30	3.6 day
M75-B	12-Sep-2012 01:07:30	12-Sep-2012 01:12:30	$<$ 5 min
M75-B	18-Sep-2012 02:07:30	18-Sep-2012 02:12:30	$<$ 5 min
M75-B	28-Sep-2012 16:07:30	28-Sep-2012 16:12:30	$<$ 5 min
M75-B	02-Oct-2012 18:47:30	02-Oct-2012 18:52:30	$<$ 5 min
M75-B	12-Oct-2012 06:57:30	30-Oct-2012 15:12:30	18.3 day
M75-B	13-Dec-2012 16:27:30	13-Dec-2012 16:32:30	$<$ 5 min
M75-B	23-Dec-2012 00:02:30	23-Dec-2012 00:07:30	$<$ 5 min
M75-B	24-Feb-2013 00:02:30	24-Feb-2013 00:07:30	$<$ 5 min
M75-B	31-Mar-2013 00:02:30	31-Mar-2013 00:07:30	$<$ 5 min
M75-B	04-Apr-2013 00:02:30	04-Apr-2013 00:07:30	$<$ 5 min
HIGH-B	06-Sep-2012 18:42:30	06-Sep-2012 19:32:30	50 min
HIGH-B	07-Sep-2012 19:57:30	11-Sep-2012 10:57:30	3.6 day
HIGH-B	12-Sep-2012 01:12:30	12-Sep-2012 01:17:30	$<$ 5 min
HIGH-B	21-Sep-2012 01:42:30	21-Sep-2012 01:52:30	10 min
HIGH-B	28-Sep-2012 15:57:30	28-Sep-2012 16:02:30	$<$ 5 min
HIGH-B	04-Oct-2012 15:52:30	04-Oct-2012 16:27:30	35 min
HIGH-B	25-Oct-2012 03:22:30	25-Oct-2012 23:57:30	20.6 hr
HIGH-B	30-Oct-2012 00:02:30	30-Oct-2012 16:27:30	16.4 hr
HIGH-B	28-Nov-2012 00:02:30	28-Nov-2012 00:07:30	$<$ 5 min
HIGH-B	21-Dec-2012 16:02:30	21-Dec-2012 16:07:30	$<$ 5 min
HIGH-B	31-Mar-2013 00:02:30	31-Mar-2013 00:07:30	$<$ 5 min
HIGH-B	29-May-2014 00:02:30	29-May-2014 00:07:30	$<$ 5 min

Table 9: MagEIS Aperture and Slit Dimensions and Angles (see Figure 8, right inset)

UNIT	L, cm	A_2, cm	A_1, cm	Σ_2, cm	Σ_1, cm	$\theta_{min,2}$	$\theta_{max,2}$	$\theta_{min,1}$	$\theta_{max,1}$
LOW/MED	6.6040	2.4917	1.6383	0.1753	0.4928	9.95°	11.42°	4.96°	9.17°
HIGH	8.3808	3.1293	2.4536	0.2000	1.0000	9.91°	11.23°	4.96°	11.64°

Table 10: Chamber magnetic field scalings for each pixel used in the hybrid responses

Unit[a]	P0 [%]	P1 [%]	P2 [%]	P3 [%]	P4 [%]	P5 [%]	P6 [%]	P7 [%]	P8 [%]
LOW-A	106.5	106.5	102	106.5	106.5	106.5	106.5	106.5	106.5
LOW-B	106.5	106.5	104	104	104	102	102	102	102
M35-A	100	100	100	100	100	100	100	100	100
M35-B	104	104	104	104	103	103	101	101	101
M75-A	101	101	101	101	102	102	102	102	101
M75-B	103	103	103	103	103	103	102	102	101
HIGH-A (before 03 Aug 2013)	96	98	100	110	X	X	X	X	X
HIGH-A (after 03 Aug 2013)	94	96	102.5	111.5	X	X	X	X	X
HIGH-B (before 03 Aug 2013)	94	98	105	110	X	X	X	X	X
HIGH-B (after 03 Aug 2013)	94	98	102.5	107	X	X	X	X	X

[a]Each HIGH unit has two separate scalings to account for the threshold changes that took place between 03 Jul 2013 and 26 Sep 2013 (see Sect. 2.2)

References

Blake JB, Carranza PA, Claudepierre SG, Clemmons JH, Crain WR, Dotan Y, Fennell JF, Fuentes FH, Galvan RM, George JS, Henderson MG, Lalic M, Lin AY, Looper MD, Mabry DJ, Mazur JE, McCarthy B, Nguyen CQ, O'Brien TP, Perez MA, Redding MT, Roeder JL, Salvaggio DJ, Sorensen GA, Spence HE, Yi S, Zakrzewski MP (2013) The Magnetic Electron Ion Spectrometer (MagEIS) Instruments Aboard the Radiation Belt Storm Probes (RBSP) Spacecraft. Space Sci Rev 179:383–421, DOI 10.1007/s11214-013-9991-8

Claudepierre SG, O'Brien TP, Blake JB, Fennell JF, Roeder JL, Clemmons JH, Looper MD, Mazur JE, Mulligan TM, Spence HE, Reeves GD, Friedel RHW, Henderson MG, Larsen BA (2015) A background correction algorithm for Van Allen Probes MagEIS electron flux measurements. J Geophys Res 120:5703–5727, DOI 10.1002/2015JA021171
