# Supplementary material for: The Magnetic Electron Ion Spectrometer: A Review of On-Orbit Sensor Performance, Data, Operations, and Science
Source: Space Sci Rev. 2021 Oct 28;217(8):80. doi: 10.1007/s11214-021-00855-2 (PMC8553741; doi:10.1007/s11214-021-00855-2)
Supplement: Supplementary file 2 — Tables of the calibration (energy channel definitions/flux conversion) factors for all of the LUTs used on orbit for the electron high rates (PDF 91 kB) [file 11214_2021_855_MOESM2_ESM.pdf]

MagEIS Calibration Factors: Electron High Rates

June 18, 2021

Table 1 through Table 24 provide energy channel definitions and flux conversion factors for the high rate electron channels on both Probes.

Probe A (LOW)

Table 1: Energy/Flux Calibration Factors for the High Rate Electron Channels. High rate LUT ID (LOW-A): 16896.

CH #	PIX	E [keV]	ΔE (E_{lo},E_{hi}) [keV]	$\Delta E/E$ [%]	$G_0\Delta E$ [cm^2 sr keV]	G_0 [cm^2 sr]	$\Delta G_0/G_0$ [%]
0	P1,P2	26	24 (17,41)	92	9.400E-02	3.917E-03	4.5
1	P3,P4	63	49 (46,95)	78	1.300E-01	2.653E-03	3.4
2	P5,P6,P7	126	109 (95,204)	87	1.990E-01	1.826E-03	5.4

Table 2: Energy/Flux Calibration Factors for the High Rate Electron Channels. High rate LUT ID (LOW-A): 16897.

CH #	PIX	E [keV]	ΔE (E_{lo},E_{hi}) [keV]	$\Delta E/E$ [%]	$G_0\Delta E$ [cm^2 sr keV]	G_0 [cm^2 sr]	$\Delta G_0/G_0$ [%]
0	P2	33	16 (27,43)	48	5.470E-02	3.419E-03	2.0
1	P3,P4	62	49 (46,95)	79	1.200E-01	2.449E-03	3.3
2	P5,P6,P7,P8	128	141 (95,236)	110	1.900E-01	1.348E-03	7.3

Table 3: Energy/Flux Calibration Factors for the High Rate Electron Channels. High rate LUT ID (LOW-A): 16898.

CH #	PIX	E [keV]	ΔE (E_{lo},E_{hi}) [keV]	$\Delta E/E$ [%]	$G_0\Delta E$ [cm^2 sr keV]	G_0 [cm^2 sr]	$\Delta G_0/G_0$ [%]
0	P2	32	13 (27,40)	41	4.490E-02	3.454E-03	1.4
1	P3,P4	62	46 (46,92)	74	1.120E-01	2.435E-03	3.1
2	P5,P6,P7,P8	129	152 (95,247)	118	2.080E-01	1.368E-03	8.0

Table 4: Energy/Flux Calibration Factors for the High Rate Electron Channels. High rate LUT ID (LOW-A): 16899.

CH #	PIX	E [keV]	ΔE (E_{lo},E_{hi}) [keV]	$\Delta E/E$ [%]	$G_0\Delta E$ [cm^2 sr keV]	G_0 [cm^2 sr]	$\Delta G_0/G_0$ [%]
0	P2	33	14 (27,41)	42	4.800E-02	3.429E-03	1.5
1	P3	54	20 (46,66)	37	5.730E-02	2.865E-03	1.2
2	P4	78	24 (68,92)	31	6.056E-02	2.523E-03	0.9
3	P5	108	31 (95,126)	29	6.880E-02	2.219E-03	1.1
4	P6	143	38 (126,164)	27	7.350E-02	1.934E-03	1.3
5	P7	182	40 (164,204)	22	6.900E-02	1.725E-03	1.5
6	P8	223	41 (206,247)	18	5.980E-02	1.459E-03	1.9

Probe A (M35)

Table 5: Energy/Flux Calibration Factors for the High Rate Electron Channels. High rate LUT ID (M35-A): 20992.

CH #	PIX	E [keV]	ΔE (E_{lo},E_{hi}) [keV]	$\Delta E/E$ [%]	$G_0\Delta E$ [cm^2 sr keV]	G_0 [cm^2 sr]	$\Delta G_0/G_0$ [%]
0	P1	146	77 (112,189)	53	3.190E-01	4.143E-03	3.7
1	P2,P3,P4	288	326 (189,515)	113	9.670E-01	2.966E-03	6.7
2	P5,P6,P7,P8	684	601 (515,1116)	88	9.440E-01	1.571E-03	6.7

Table 6: Energy/Flux Calibration Factors for the High Rate Electron Channels. High rate LUT ID (M35-A): 20993.

CH #	PIX	E [keV]	ΔE (E_{lo},E_{hi}) [keV]	$\Delta E/E$ [%]	$G_0\Delta E$ [cm^2 sr keV]	G_0 [cm^2 sr]	$\Delta G_0/G_0$ [%]
0	P2,P3	260	198 (189,387)	76	6.060E-01	3.061E-03	4.1
1	P4,P5	492	254 (392,646)	52	6.070E-01	2.390E-03	2.3
2	P6,P7,P8	802	462 (654,1116)	58	6.830E-01	1.478E-03	4.7

Table 7: Energy/Flux Calibration Factors for the High Rate Electron Channels. High rate LUT ID (M35-A): 20994.

CH #	PIX	E [keV]	ΔE (E_{lo},E_{hi}) [keV]	$\Delta E/E$ [%]	$G_0\Delta E$ [cm^2 sr keV]	G_0 [cm^2 sr]	$\Delta G_0/G_0$ [%]
0	P2	230	86 (189,275)	37	3.080E-01	3.581E-03	2.0
1	P3	334	102 (285,387)	31	3.220E-01	3.157E-03	1.3
2	P4	454	123 (392,515)	27	3.240E-01	2.634E-03	1.2
3	P5	584	139 (515,654)	24	3.070E-01	2.209E-03	1.1
4	P6	724	148 (654,802)	20	2.835E-01	1.916E-03	1.0
5	P7	879	161 (802,963)	18	2.520E-01	1.565E-03	1.0
6	P8	1031	153 (963,1116)	15	2.020E-01	1.320E-03	1.5

Probe A (M75)

Table 8: Energy/Flux Calibration Factors for the High Rate Electron Channels. High rate LUT ID (M75-A): 25088.

CH #	PIX	E [keV]	ΔE (E_{lo},E_{hi}) [keV]	$\Delta E/E$ [%]	$G_0 \Delta E$ [cm^2 sr keV]	G_0 [cm^2 sr]	$\Delta G_0/G_0$ [%]
0	P0,P1	127	109 (85,194)	86	3.680E-01	3.376E-03	6.4
1	P2,P3,P4	295	333 (194,527)	113	9.720E-01	2.919E-03	6.7
2	P5,P6,P7,P8	700	596 (533,1129)	85	9.810E-01	1.646E-03	6.6

Table 9: Energy/Flux Calibration Factors for the High Rate Electron Channels. High rate LUT ID (M75-A): 25089.

CH #	PIX	E [keV]	ΔE (E_{lo},E_{hi}) [keV]	$\Delta E/E$ [%]	$G_0 \Delta E$ [cm^2 sr keV]	G_0 [cm^2 sr]	$\Delta G_0/G_0$ [%]
0	P2,P3	269	202 (194,396)	75	6.860E-01	3.396E-03	4.3
1	P4,P5	509	264 (405,669)	52	6.470E-01	2.451E-03	2.3
2	P6,P7,P8	821	460 (669,1129)	56	7.220E-01	1.570E-03	4.8

Table 10: Energy/Flux Calibration Factors for the High Rate Electron Channels. High rate LUT ID (M75-A): 25090.

CH #	PIX	E [keV]	ΔE (E_{lo},E_{hi}) [keV]	$\Delta E/E$ [%]	$G_0 \Delta E$ [cm^2 sr keV]	G_0 [cm^2 sr]	$\Delta G_0/G_0$ [%]
0	P2,P3	266	202 (194,396)	76	6.350E-01	3.144E-03	4.1
1	P4,P5	509	256 (405,661)	50	6.160E-01	2.406E-03	2.2
2	P6,P7,P8	821	473 (669,1142)	58	6.840E-01	1.446E-03	4.9

Table 11: Energy/Flux Calibration Factors for the High Rate Electron Channels. High rate LUT ID (M75-A): 25091.

CH #	PIX	E [keV]	ΔE (E_{lo},E_{hi}) [keV]	$\Delta E/E$ [%]	$G_0 \Delta E$ [cm^2 sr keV]	G_0 [cm^2 sr]	$\Delta G_0/G_0$ [%]
0	P2	235	91 (194,285)	39	3.260E-01	3.582E-03	2.1
1	P3	342	108 (288,396)	32	3.270E-01	3.028E-03	1.3
2	P4	465	128 (405,533)	28	3.280E-01	2.563E-03	1.1
3	P5,P6	646	288 (533,821)	45	5.710E-01	1.983E-03	2.5
4	P7,P8	941	321 (821,1142)	34	4.440E-01	1.383E-03	3.0

Table 12: Energy/Flux Calibration Factors for the High Rate Electron Channels. High rate LUT ID (M75-A): 25092.

CH #	PIX	E [keV]	ΔE (E_{lo},E_{hi}) [keV]	$\Delta E/E$ [%]	$G_0 \Delta E$ [cm^2 sr keV]	G_0 [cm^2 sr]	$\Delta G_0/G_0$ [%]
0	P2	235	91 (194,285)	39	3.260E-01	3.582E-03	2.1
1	P3	342	108 (288,396)	32	3.270E-01	3.028E-03	1.3
2	P4	465	128 (405,533)	28	3.280E-01	2.563E-03	1.1
3	P5	597	136 (533,669)	23	3.020E-01	2.221E-03	1.0
4	P6	741	152 (669,821)	21	2.810E-01	1.849E-03	1.1
5	P7	899	164 (821,985)	18	2.500E-01	1.524E-03	1.1
6	P8	1054	168 (974,1142)	16	2.130E-01	1.268E-03	1.1

Probe B (LOW)

Table 13: Energy/Flux Calibration Factors for the High Rate Electron Channels. High rate LUT ID (LOW-B): 18944.

CH #	PIX	E [keV]	ΔE (E_{lo},E_{hi}) [keV]	$\Delta E/E$ [%]	$G_0\Delta E$ [cm^2 sr keV]	G_0 [cm^2 sr]	$\Delta G_0/G_0$ [%]
0	P1,P2	28	24 (20,44)	86	9.070E-02	3.779E-03	3.9
1	P3,P4	61	47 (44,91)	77	1.220E-01	2.596E-03	3.2
2	P5,P6,P7	117	102 (88,190)	87	1.860E-01	1.824E-03	5.1

Table 14: Energy/Flux Calibration Factors for the High Rate Electron Channels. High rate LUT ID (LOW-B): 18945.

H #	PIX	E [keV]	ΔE (E_{lo},E_{hi}) [keV]	$\Delta E/E$ [%]	$G_0\Delta E$ [cm^2 sr keV]	G_0 [cm^2 sr]	$\Delta G_0/G_0$ [%]
0	P2	35	16 (28,44)	46	5.000E-02	3.125E-03	1.4
1	P3,P4	60	43 (45,88)	72	1.060E-01	2.465E-03	2.9
2	P5,P6,P7,P8	117	120 (88,208)	103	1.650E-01	1.375E-03	5.2

Table 15: Energy/Flux Calibration Factors for the High Rate Electron Channels. High rate LUT ID (LOW-B): 18946.

CH #	PIX	E [keV]	ΔE (E_{lo},E_{hi}) [keV]	$\Delta E/E$ [%]	$G_0\Delta E$ [cm^2 sr keV]	G_0 [cm^2 sr]	$\Delta G_0/G_0$ [%]
0	P2	33	12 (28,40)	36	4.280E-02	3.567E-03	1.4
1	P3,P4	59	44 (44,88)	75	1.080E-01	2.455E-03	3.0
2	P5,P6,P7,P8	121	143 (88,231)	118	2.020E-01	1.413E-03	7.3

Table 16: Energy/Flux Calibration Factors for the High Rate Electron Channels. High rate LUT ID (LOW-B): 18947.

CH #	PIX	E [keV]	ΔE (E_{lo},E_{hi}) [keV]	$\Delta E/E$ [%]	$G_0\Delta E$ [cm^2 sr keV]	G_0 [cm^2 sr]	$\Delta G_0/G_0$ [%]
0	P2	33	11 (28,39)	33	3.960E-02	3.600E-03	1.4
1	P3	53	19 (44,63)	36	5.470E-02	2.879E-03	1.0
2	P4	76	24 (65,89)	32	5.928E-02	2.470E-03	0.8
3	P5	101	29 (88,117)	29	6.605E-02	2.278E-03	0.8
4	P6	132	33 (117,150)	25	6.460E-02	1.958E-03	0.9
5	P7	168	36 (152,188)	21	6.230E-02	1.731E-03	1.2
6	P8	208	38 (193,231)	18	5.960E-02	1.568E-03	1.4

Probe B (M35)

Table 17: Energy/Flux Calibration Factors for the High Rate Electron Channels. High rate LUT ID (M35-B): 39424.

CH #	PIX	E [keV]	ΔE (E_{lo},E_{hi}) [keV]	$\Delta E/E$ [%]	$G_0 \Delta E$ [cm^2 sr keV]	G_0 [cm^2 sr]	$\Delta G_0/G_0$ [%]
0	P0,P1	136	115 (90,205)	85	4.110E-01	3.574E-03	6.0
1	P2,P3,P4	305	330 (203,533)	108	1.040E+00	3.152E-03	6.3
2	P5,P6,P7,P8	708	571 (545,1116)	81	1.020E+00	1.786E-03	6.4

Table 18: Energy/Flux Calibration Factors for the High Rate Electron Channels. High rate LUT ID (M35-B): 39425.

CH #	PIX	E [keV]	ΔE (E_{lo},E_{hi}) [keV]	$\Delta E/E$ [%]	$G_0 \Delta E$ [cm^2 sr keV]	G_0 [cm^2 sr]	$\Delta G_0/G_0$ [%]
0	P2,P3	282	207 (203,410)	73	6.830E-01	3.300E-03	4.0
1	P4,P5	515	262 (415,677)	51	6.120E-01	2.336E-03	2.4
2	P6,P7,P8	821	473 (669,1142)	58	6.720E-01	1.421E-03	4.9

Table 19: Energy/Flux Calibration Factors for the High Rate Electron Channels. High rate LUT ID (M35-B): 39426.

CH #	PIX	E [keV]	ΔE (E_{lo},E_{hi}) [keV]	$\Delta E/E$ [%]	$G_0 \Delta E$ [cm^2 sr keV]	G_0 [cm^2 sr]	$\Delta G_0/G_0$ [%]
0	P2	249	95 (203,298)	38	3.510E-01	3.695E-03	2.0
1	P3	358	110 (305,415)	31	3.550E-01	3.227E-03	1.4
2	P4	475	124 (415,539)	26	3.250E-01	2.621E-03	1.1
3	P5	604	138 (539,677)	23	3.090E-01	2.239E-03	1.2
4	P6	741	143 (669,812)	19	2.670E-01	1.867E-03	1.0
5	P7	889	162 (812,974)	18	2.470E-01	1.525E-03	1.1
6	P8	1054	168 (974,1142)	16	2.160E-01	1.286E-03	1.0

Probe B (M75)

Table 20: Energy/Flux Calibration Factors for the High Rate Electron Channels. High rate LUT ID (M75-B): 27136.

CH #	PIX	E [keV]	ΔE (E_{lo},E_{hi}) [keV]	$\Delta E/E$ [%]	$G_0\Delta E$ [cm^2 sr keV]	G_0 [cm^2 sr]	$\Delta G_0/G_0$ [%]
0	P0,P1	141	110 (90,200)	78	3.670E-01	3.336E-03	5.2
1	P2,P3,P4	302	327 (200,527)	108	9.640E-01	2.948E-03	6.3
2	P5,P6,P7,P8	700	564 (539,1103)	81	9.470E-01	1.679E-03	6.0

Table 21: Energy/Flux Calibration Factors for the High Rate Electron Channels. High rate LUT ID (M75-B): 27137.

CH #	PIX	E [keV]	ΔE (E_{lo},E_{hi}) [keV]	$\Delta E/E$ [%]	$G_0\Delta E$ [cm^2 sr keV]	G_0 [cm^2 sr]	$\Delta G_0/G_0$ [%]
0	P2,P3	275	210 (200,410)	76	6.790E-01	3.233E-03	4.2
1	P4,P5	515	267 (410,677)	52	6.380E-01	2.390E-03	2.4
2	P6,P7,P8	812	434 (669,1103)	53	6.690E-01	1.541E-03	4.6

Table 22: Energy/Flux Calibration Factors for the High Rate Electron Channels. High rate LUT ID (M75-B): 27138.

CH #	PIX	E [keV]	ΔE (E_{lo},E_{hi}) [keV]	$\Delta E/E$ [%]	$G_0\Delta E$ [cm^2 sr keV]	G_0 [cm^2 sr]	$\Delta G_0/G_0$ [%]
0	P2,P3	275	205 (200,405)	75	6.270E-01	3.059E-03	3.9
1	P4,P5	515	254 (415,669)	49	6.170E-01	2.429E-03	2.2
2	P6,P7,P8	821	447 (669,1116)	54	6.590E-01	1.474E-03	4.6

Table 23: Energy/Flux Calibration Factors for the High Rate Electron Channels. High rate LUT ID (M75-B): 27139.

CH #	PIX	E [keV]	ΔE (E_{lo},E_{hi}) [keV]	$\Delta E/E$ [%]	$G_0\Delta E$ [cm^2 sr keV]	G_0 [cm^2 sr]	$\Delta G_0/G_0$ [%]
0	P2	243	92 (200,292)	38	3.300E-01	3.587E-03	2.1
1	P3	350	107 (298,405)	31	3.330E-01	3.112E-03	1.4
2	P4	470	118 (415,533)	25	3.210E-01	2.720E-03	1.2
3	P5,P6	646	282 (539,821)	44	5.580E-01	1.979E-03	2.4
4	P7,P8	941	295 (821,1116)	31	4.240E-01	1.437E-03	2.5

Table 24: Energy/Flux Calibration Factors for the High Rate Electron Channels. High rate LUT ID (M75-B): 27140.

CH #	PIX	E [keV]	ΔE (E_{lo},E_{hi}) [keV]	$\Delta E/E$ [%]	$G_0\Delta E$ [cm^2 sr keV]	G_0 [cm^2 sr]	$\Delta G_0/G_0$ [%]
0	P2	243	92 (200,292)	38	3.300E-01	3.587E-03	2.1
1	P3	350	107 (298,405)	31	3.330E-01	3.112E-03	1.4
2	P4	470	118 (415,533)	25	3.210E-01	2.720E-03	1.2
3	P5	604	130 (539,669)	22	3.009E-01	2.315E-03	1.0
4	P6	741	152 (669,821)	21	2.760E-01	1.816E-03	1.1
5	P7	899	164 (821,985)	18	2.470E-01	1.506E-03	1.0
6	P8	1054	142 (974,1116)	13	1.930E-01	1.359E-03	1.5
