# Supplementary material for: The Magnetic Electron Ion Spectrometer: A Review of On-Orbit Sensor Performance, Data, Operations, and Science
Source: Space Sci Rev. 2021 Oct 28;217(8):80. doi: 10.1007/s11214-021-00855-2 (PMC8553741; doi:10.1007/s11214-021-00855-2)
Supplement: Supplementary file 3 — Tables of the calibration (energy channel definitions/flux conversion) factors for all of the LUTs used on orbit for the electron histograms (PDF 218 kB) [file 11214_2021_855_MOESM3_ESM.pdf]

# MagEIS Calibration Factors: Electron Histograms

June 18, 2021

Table 1 through Table 16 provide energy channel definitions and flux conversion factors for the histogram electron channels on both Probes.

## Probe A (LOW)

Table 1: Energy/Flux Calibration Factors for the Histogram Electron Channels. Histogram LUT ID (LOW-A): 16641.

| CH # | PIX | $E$ [keV] | $\Delta E$ ($E_{lo}$,$E_{hi}$) [keV] | $\Delta E/E$ [%] | $G_0 \Delta E$ [cm$^2$ sr keV] | $G_0$ [cm$^2$ sr] | $\Delta G_0/G_0$ [%] |
|---|---|---|---|---|---|---|---|
| 0 | P1 | 21.2 | 1.5 (20.2,21.7) | 7.1 | 4.383E-03 | 2.922E-03 | 0.8 |
| 1 | P1 | 22.4 | 1.3 (21.4,22.7) | 5.8 | 4.412E-03 | 3.394E-03 | 0.5 |
| 2 | P1 | 23.5 | 1.6 (22.4,24.0) | 6.8 | 4.366E-03 | 2.729E-03 | 0.5 |
| 3 | P1 | 24.6 | 1.6 (23.5,25.1) | 6.5 | 4.337E-03 | 2.711E-03 | 0.5 |
| 4 | P1 | 25.7 | 1.4 (24.6,26.0) | 5.4 | 4.254E-03 | 3.039E-03 | 0.5 |
| 5 | P2 | 30.8 | 1.4 (29.8,31.2) | 4.5 | 3.798E-03 | 2.713E-03 | 0.5 |
| 6 | P2 | 31.9 | 1.5 (30.8,32.3) | 4.7 | 3.872E-03 | 2.581E-03 | 0.5 |
| 7 | P2 | 33.0 | 1.5 (31.9,33.4) | 4.5 | 3.889E-03 | 2.593E-03 | 0.5 |
| 8 | P2 | 34.2 | 1.6 (33.0,34.6) | 4.7 | 3.855E-03 | 2.409E-03 | 0.7 |
| 9 | P2 | 34.9 | 1.1 (34.2,35.3) | 3.2 | 3.805E-03 | 3.459E-03 | 0.6 |
| 10 | P2 | 36.2 | 1.7 (34.9,36.6) | 4.7 | 3.798E-03 | 2.234E-03 | 0.4 |
| 11 | P2 | 37.4 | 1.6 (36.2,37.8) | 4.3 | 3.823E-03 | 2.389E-03 | 0.6 |
| 12 | P2 | 38.3 | 1.7 (37.0,38.7) | 4.4 | 3.701E-03 | 2.177E-03 | 0.4 |
| 13 | P3 | 52.0 | 1.7 (50.9,52.6) | 3.3 | 3.394E-03 | 1.996E-03 | 0.3 |
| 14 | P3 | 53.2 | 1.8 (52.0,53.8) | 3.4 | 3.483E-03 | 1.935E-03 | 0.3 |
| 15 | P3 | 54.5 | 1.3 (53.2,54.5) | 2.4 | 3.491E-03 | 2.685E-03 | 0.5 |
| 16 | P3 | 55.1 | 1.9 (53.8,55.7) | 3.4 | 3.410E-03 | 1.795E-03 | 0.7 |
| 17 | P3 | 56.4 | 1.9 (55.1,57.0) | 3.4 | 3.401E-03 | 1.790E-03 | 0.4 |
| 18 | P3 | 57.6 | 1.9 (56.4,58.3) | 3.3 | 3.422E-03 | 1.801E-03 | 0.2 |
| 19 | P3 | 59.0 | 1.4 (57.6,59.0) | 2.4 | 3.433E-03 | 2.452E-03 | 0.6 |
| 20 | P3 | 59.7 | 2.0 (58.3,60.3) | 3.4 | 3.359E-03 | 1.680E-03 | 0.5 |
| 21 | P3 | 61.0 | 1.3 (59.7,61.0) | 2.1 | 3.347E-03 | 2.575E-03 | 0.2 |
| 22 | P3 | 61.7 | 2.1 (60.3,62.4) | 3.4 | 3.305E-03 | 1.574E-03 | 0.7 |
| 23 | P3 | 63.1 | 2.2 (61.7,63.9) | 3.5 | 3.359E-03 | 1.527E-03 | 0.3 |
| 24 | P4 | 74.0 | 2.5 (72.4,74.9) | 3.4 | 5.791E-03 | 2.316E-03 | 0.3 |
| 25 | P4 | 76.6 | 3.5 (74.0,77.5) | 4.6 | 6.050E-03 | 1.729E-03 | 0.4 |
| 26 | P4 | 78.4 | 2.7 (76.6,79.3) | 3.4 | 5.993E-03 | 2.220E-03 | 0.5 |
| 27 | P4 | 81.1 | 3.6 (78.4,82.0) | 4.4 | 6.031E-03 | 1.675E-03 | 0.4 |
| 28 | P4 | 83.0 | 2.8 (81.1,83.9) | 3.4 | 5.883E-03 | 2.101E-03 | 0.4 |
| 29 | P4 | 85.8 | 3.8 (83.0,86.8) | 4.4 | 5.958E-03 | 1.568E-03 | 0.6 |
| 30 | P4 | 87.8 | 3.0 (85.8,88.8) | 3.4 | 5.854E-03 | 1.951E-03 | 0.3 |
| 31 | P4 | 89.8 | 3.1 (87.8,90.9) | 3.5 | 5.771E-03 | 1.862E-03 | 0.2 |
| 32 | P5 | 102.0 | 3.5 (99.5,103.0) | 3.4 | 5.227E-03 | 1.493E-03 | 0.2 |
| 33 | P5 | 104.0 | 3.0 (102.0,105.0) | 2.9 | 5.467E-03 | 1.822E-03 | 0.2 |
| 34 | P5 | 107.0 | 4.0 (104.0,108.0) | 3.7 | 5.465E-03 | 1.366E-03 | 0.5 |
| 35 | P5 | 109.0 | 2.0 (107.0,109.0) | 1.8 | 5.476E-03 | 2.738E-03 | 0.7 |
| 36 | P5 | 110.0 | 4.0 (108.0,112.0) | 3.6 | 5.413E-03 | 1.353E-03 | 0.5 |
| 37 | P5 | 113.0 | 4.0 (110.0,114.0) | 3.5 | 5.330E-03 | 1.332E-03 | 0.2 |
| 38 | P5 | 115.0 | 4.0 (113.0,117.0) | 3.5 | 5.287E-03 | 1.322E-03 | 0.4 |
| 39 | P6 | 137.0 | 4.0 (134.0,138.0) | 2.9 | 4.759E-03 | 1.190E-03 | 0.2 |
| 40 | P6 | 140.0 | 3.0 (137.0,140.0) | 2.1 | 4.920E-03 | 1.640E-03 | 0.9 |
| 41 | P6 | 142.0 | 5.0 (138.0,143.0) | 3.5 | 4.786E-03 | 9.572E-04 | 0.2 |
| 42 | P6 | 143.0 | 3.0 (142.0,145.0) | 2.1 | 4.772E-03 | 1.591E-03 | 0.5 |
| 43 | P6 | 146.5 | 3.3 (143.2,146.5) | 2.3 | 4.773E-03 | 1.446E-03 | 0.6 |
| 44 | P6 | 148.0 | 5.0 (145.0,150.0) | 3.4 | 4.693E-03 | 9.386E-04 | 0.2 |
| 45 | P7 | 175.8 | 4.0 (171.8,175.8) | 2.3 | 4.090E-03 | 1.022E-03 | 1.1 |
| 46 | P7 | 178.0 | 4.0 (174.0,178.0) | 2.2 | 4.103E-03 | 1.026E-03 | 0.9 |
| 47 | P7 | 180.0 | 6.0 (176.0,182.0) | 3.3 | 4.132E-03 | 6.887E-04 | 0.7 |
| 48 | P7 | 182.0 | 6.0 (178.0,184.0) | 3.3 | 4.076E-03 | 6.793E-04 | 0.6 |
| 49 | P7 | 184.0 | 6.0 (180.0,186.0) | 3.3 | 4.055E-03 | 6.758E-04 | 0.4 |
| 50 | P7 | 186.0 | 6.0 (182.0,188.0) | 3.2 | 3.991E-03 | 6.652E-04 | 0.4 |
| 51 | P7 | 188.0 | 6.0 (184.0,190.0) | 3.2 | 3.976E-03 | 6.627E-04 | 0.2 |
| 52 | P7 | 190.0 | 7.0 (186.0,193.0) | 3.7 | 3.959E-03 | 5.656E-04 | 0.2 |
| 53 | P7 | 193.0 | 7.0 (188.0,195.0) | 3.6 | 3.880E-03 | 5.543E-04 | 0.2 |
| 54 | P7 | 195.0 | 7.0 (190.0,197.0) | 3.6 | 3.998E-03 | 5.711E-04 | 0.3 |
| 55 | P8 | 214.9 | 6.8 (211.2,218.0) | 3.2 | 3.485E-03 | 5.125E-04 | 0.9 |
| 56 | P8 | 217.1 | 4.7 (213.5,218.2) | 2.2 | 3.550E-03 | 7.553E-04 | 1.2 |
| 57 | P8 | 219.4 | 5.2 (215.8,221.0) | 2.4 | 3.480E-03 | 6.692E-04 | 1.5 |
| 58 | P8 | 221.6 | 5.0 (218.0,223.0) | 2.3 | 3.590E-03 | 7.180E-04 | 1.2 |
| 59 | P8 | 223.9 | 5.7 (220.3,226.0) | 2.5 | 3.587E-03 | 6.293E-04 | 1.0 |
| 60 | P8 | 226.1 | 5.4 (222.6,228.0) | 2.4 | 3.623E-03 | 6.709E-04 | 0.9 |
| 61 | P8 | 228.4 | 6.2 (224.8,231.0) | 2.7 | 3.619E-03 | 5.837E-04 | 0.4 |

| | | | | | | | |
|---|---|---|---|---|---|---|---|
| 62 | P8 | 230.6 | 6.9 (227.1,234.0) | 3.0 | 3.617E-03 | 5.242E-04 | 0.3 |
| 63 | P8 | 232.9 | 6.7 (229.3,236.0) | 2.9 | 3.634E-03 | 5.424E-04 | 0.8 |
| 64 | P8 | 235.1 | 4.7 (231.6,236.3) | 2.0 | 3.580E-03 | 7.617E-04 | 1.3 |
| 65 | P8 | 237.4 | 5.1 (233.9,239.0) | 2.1 | 3.370E-03 | 6.608E-04 | 1.4 |
| 66 | P8 | 239.6 | 5.9 (236.1,242.0) | 2.5 | 3.461E-03 | 5.866E-04 | 0.9 |
| 67 | P8 | 241.9 | 5.6 (238.4,244.0) | 2.3 | 3.452E-03 | 6.164E-04 | 0.6 |
| 68 | P8 | 244.1 | 6.3 (240.7,247.0) | 2.6 | 3.481E-03 | 5.525E-04 | 0.3 |
| 69 | P8 | 246.4 | 7.1 (242.9,250.0) | 2.9 | 3.490E-03 | 4.915E-04 | 1.1 |

Table 2: Energy/Flux Calibration Factors for the Histogram Electron Channels. Histogram LUT ID (LOW-A): 16642.

| CH # | PIX | $E$ [keV] | $\Delta E$ ($E_{lo}$,$E_{hi}$) [keV] | $\Delta E/E$ [%] | $G_0\Delta E$ [cm$^2$ sr keV] | $G_0$ [cm$^2$ sr] | $\Delta G_0/G_0$ [%] |
|---|---|---|---|---|---|---|---|
| 0 | P1 | 19.6 | 2.4 (18.1,20.5) | 12.2 | 8.578E-03 | 3.574E-03 | 0.8 |
| 1 | P1 | 21.7 | 2.5 (20.2,22.7) | 11.5 | 8.717E-03 | 3.487E-03 | 0.9 |
| 2 | P1 | 24.0 | 2.7 (22.4,25.1) | 11.3 | 8.715E-03 | 3.228E-03 | 0.5 |
| 3 | P1 | 26.3 | 2.6 (24.6,27.2) | 9.9 | 8.134E-03 | 3.128E-03 | 0.7 |
| 4 | P2 | 31.2 | 2.5 (29.8,32.3) | 8.0 | 7.595E-03 | 3.038E-03 | 0.5 |
| 5 | P2 | 33.4 | 2.7 (31.9,34.6) | 8.1 | 7.689E-03 | 2.848E-03 | 0.4 |
| 6 | P2 | 35.8 | 2.4 (34.2,36.6) | 6.7 | 7.671E-03 | 3.196E-03 | 0.7 |
| 7 | P2 | 37.8 | 2.5 (36.2,38.7) | 6.6 | 7.535E-03 | 3.014E-03 | 0.4 |
| 8 | P3 | 51.5 | 2.9 (49.7,52.6) | 5.6 | 6.668E-03 | 2.299E-03 | 0.3 |
| 9 | P3 | 53.8 | 2.5 (52.0,54.5) | 4.6 | 6.974E-03 | 2.790E-03 | 0.4 |
| 10 | P3 | 55.7 | 3.2 (53.8,57.0) | 5.7 | 6.812E-03 | 2.129E-03 | 0.5 |
| 11 | P3 | 58.3 | 2.6 (56.4,59.0) | 4.5 | 6.856E-03 | 2.637E-03 | 0.4 |
| 12 | P3 | 60.3 | 3.4 (58.3,61.7) | 5.6 | 6.711E-03 | 1.974E-03 | 0.3 |
| 13 | P3 | 62.4 | 3.6 (60.3,63.9) | 5.8 | 6.664E-03 | 1.851E-03 | 0.5 |
| 14 | P4 | 74.0 | 2.5 (72.4,74.9) | 3.4 | 5.791E-03 | 2.316E-03 | 0.3 |
| 15 | P4 | 76.6 | 3.5 (74.0,77.5) | 4.6 | 6.050E-03 | 1.729E-03 | 0.4 |
| 16 | P4 | 78.4 | 2.7 (76.6,79.3) | 3.4 | 5.993E-03 | 2.220E-03 | 0.5 |
| 17 | P4 | 81.1 | 3.6 (78.4,82.0) | 4.4 | 6.031E-03 | 1.675E-03 | 0.4 |
| 18 | P4 | 83.0 | 2.8 (81.1,83.9) | 3.4 | 5.883E-03 | 2.101E-03 | 0.4 |
| 19 | P4 | 85.8 | 3.8 (83.0,86.8) | 4.4 | 5.958E-03 | 1.568E-03 | 0.6 |
| 20 | P4 | 87.8 | 3.0 (85.8,88.8) | 3.4 | 5.854E-03 | 1.951E-03 | 0.3 |
| 21 | P4 | 89.8 | 3.1 (87.8,90.9) | 3.5 | 5.771E-03 | 1.862E-03 | 0.2 |
| 22 | P5 | 103.0 | 3.0 (101.0,104.0) | 2.9 | 5.352E-03 | 1.784E-03 | 0.2 |
| 23 | P5 | 105.0 | 4.0 (103.0,107.0) | 3.8 | 5.458E-03 | 1.365E-03 | 0.3 |
| 24 | P5 | 108.0 | 4.0 (105.0,109.0) | 3.7 | 5.501E-03 | 1.375E-03 | 0.6 |
| 25 | P5 | 109.0 | 3.0 (107.0,110.0) | 2.8 | 5.378E-03 | 1.793E-03 | 0.7 |
| 26 | P5 | 112.0 | 4.0 (109.0,113.0) | 3.6 | 5.383E-03 | 1.346E-03 | 0.3 |
| 27 | P5 | 114.0 | 3.0 (112.0,115.0) | 2.6 | 5.295E-03 | 1.765E-03 | 0.2 |
| 28 | P5 | 116.7 | 2.6 (114.1,116.7) | 2.2 | 5.282E-03 | 2.032E-03 | 0.7 |
| 29 | P6 | 137.0 | 4.0 (134.0,138.0) | 2.9 | 4.759E-03 | 1.190E-03 | 0.2 |
| 30 | P6 | 140.0 | 3.0 (137.0,140.0) | 2.1 | 4.920E-03 | 1.640E-03 | 0.9 |
| 31 | P6 | 142.0 | 5.0 (138.0,143.0) | 3.5 | 4.786E-03 | 9.572E-04 | 0.2 |
| 32 | P6 | 143.0 | 3.0 (142.0,145.0) | 2.1 | 4.772E-03 | 1.591E-03 | 0.5 |
| 33 | P6 | 146.5 | 3.3 (143.2,146.5) | 2.3 | 4.773E-03 | 1.446E-03 | 0.6 |
| 34 | P6 | 148.0 | 5.0 (145.0,150.0) | 3.4 | 4.693E-03 | 9.386E-04 | 0.2 |
| 35 | P6 | 150.0 | 4.0 (148.0,152.0) | 2.7 | 4.554E-03 | 1.138E-03 | 0.5 |
| 36 | P6 | 153.0 | 3.0 (150.0,153.0) | 2.0 | 4.646E-03 | 1.549E-03 | 0.7 |
| 37 | P6 | 155.0 | 5.0 (152.0,157.0) | 3.2 | 4.575E-03 | 9.150E-04 | 0.3 |
| 38 | P7 | 175.8 | 4.0 (171.8,175.8) | 2.3 | 4.090E-03 | 1.022E-03 | 1.1 |
| 39 | P7 | 178.0 | 4.0 (174.0,178.0) | 2.2 | 4.103E-03 | 1.026E-03 | 0.9 |
| 40 | P7 | 180.0 | 6.0 (176.0,182.0) | 3.3 | 4.132E-03 | 6.887E-04 | 0.7 |
| 41 | P7 | 182.0 | 6.0 (178.0,184.0) | 3.3 | 4.076E-03 | 6.793E-04 | 0.6 |
| 42 | P7 | 184.0 | 6.0 (180.0,186.0) | 3.3 | 4.055E-03 | 6.758E-04 | 0.4 |
| 43 | P7 | 186.0 | 6.0 (182.0,188.0) | 3.2 | 3.991E-03 | 6.652E-04 | 0.4 |
| 44 | P7 | 188.0 | 6.0 (184.0,190.0) | 3.2 | 3.976E-03 | 6.627E-04 | 0.2 |
| 45 | P7 | 190.0 | 7.0 (186.0,193.0) | 3.7 | 3.959E-03 | 5.656E-04 | 0.2 |
| 46 | P7 | 193.0 | 7.0 (188.0,195.0) | 3.6 | 3.880E-03 | 5.543E-04 | 0.2 |
| 47 | P7 | 195.0 | 7.0 (190.0,197.0) | 3.6 | 3.998E-03 | 5.711E-04 | 0.3 |
| 48 | P8 | 216.0 | 5.2 (213.0,218.2) | 2.4 | 3.502E-03 | 6.735E-04 | 0.7 |
| 49 | P8 | 218.3 | 5.4 (215.1,220.5) | 2.5 | 3.479E-03 | 6.443E-04 | 0.4 |
| 50 | P8 | 220.5 | 5.5 (217.3,222.8) | 2.5 | 3.599E-03 | 6.544E-04 | 0.2 |
| 51 | P8 | 222.8 | 5.7 (219.4,225.1) | 2.6 | 3.662E-03 | 6.425E-04 | 0.4 |

| | | | | | | | |
|---|---|---|---|---|---|---|---|
| 52 | P8 | 225.1 | 5.8 (221.6,227.4) | 2.6 | 3.665E-03 | 6.319E-04 | 0.7 |
| 53 | P8 | 227.4 | 5.9 (223.8,229.7) | 2.6 | 3.650E-03 | 6.186E-04 | 1.2 |
| 54 | P8 | 229.6 | 6.1 (225.9,232.0) | 2.7 | 3.570E-03 | 5.852E-04 | 1.4 |
| 55 | P8 | 231.9 | 6.2 (228.1,234.3) | 2.7 | 3.579E-03 | 5.773E-04 | 1.0 |
| 56 | P8 | 234.2 | 6.4 (230.2,236.6) | 2.7 | 3.568E-03 | 5.575E-04 | 0.9 |
| 57 | P8 | 236.5 | 6.5 (232.4,238.9) | 2.7 | 3.472E-03 | 5.342E-04 | 0.4 |
| 58 | P8 | 238.7 | 6.7 (234.5,241.2) | 2.8 | 3.422E-03 | 5.107E-04 | 0.4 |
| 59 | P8 | 241.0 | 6.8 (236.7,243.5) | 2.8 | 3.540E-03 | 5.206E-04 | 1.1 |

## Probe A (M35)

Table 3: Energy/Flux Calibration Factors for the Histogram Electron Channels. Histogram LUT ID (M35-A): 20737.

| CH # | PIX | $E$ [keV] | $\Delta E$ ($E_{lo}$,$E_{hi}$) [keV] | $\Delta E/E$ [%] | $G_0\Delta E$ [cm$^2$ sr keV] | $G_0$ [cm$^2$ sr] | $\Delta G_0/G_0$ [%] |
|---|---|---|---|---|---|---|---|
| 0 | P1 | 131 | 5 (126,131) | 3.8 | 1.880E-02 | 3.760E-03 | 1.5 |
| 1 | P1 | 136 | 6 (130,136) | 4.4 | 1.940E-02 | 3.233E-03 | 1.4 |
| 2 | P1 | 141 | 6 (134,141) | 4.5 | 1.960E-02 | 3.111E-03 | 1.3 |
| 3 | P1 | 146 | 7 (139,146) | 4.8 | 1.960E-02 | 2.800E-03 | 1.2 |
| 4 | P1 | 151 | 7 (144,151) | 4.4 | 1.960E-02 | 2.925E-03 | 1.2 |
| 5 | P1 | 156 | 7 (149,156) | 4.5 | 1.950E-02 | 2.786E-03 | 1.2 |
| 6 | P1 | 160 | 6 (154,160) | 3.8 | 1.940E-02 | 3.233E-03 | 1.2 |
| 7 | P1 | 165 | 7 (158,165) | 4.2 | 1.920E-02 | 2.743E-03 | 1.1 |
| 8 | P1 | 169 | 6 (163,169) | 3.6 | 1.890E-02 | 3.150E-03 | 1.2 |
| 9 | P1 | 175 | 8 (167,175) | 4.6 | 1.820E-02 | 2.275E-03 | 1.1 |
| 10 | P2 | 210 | 8 (202,210) | 3.7 | 1.641E-02 | 2.131E-03 | 0.9 |
| 11 | P2 | 214 | 7 (207,214) | 3.3 | 1.735E-02 | 2.444E-03 | 0.9 |
| 12 | P2 | 219 | 7 (212,219) | 3.4 | 1.793E-02 | 2.423E-03 | 0.9 |
| 13 | P2 | 224 | 8 (216,224) | 3.5 | 1.798E-02 | 2.305E-03 | 1.0 |
| 14 | P2 | 227 | 6 (221,227) | 2.7 | 1.790E-02 | 2.934E-03 | 1.0 |
| 15 | P2 | 232 | 7 (226,232) | 2.9 | 1.794E-02 | 2.678E-03 | 0.9 |
| 16 | P2 | 238 | 8 (230,238) | 3.3 | 1.774E-02 | 2.274E-03 | 0.8 |
| 17 | P2 | 243 | 8 (235,243) | 3.4 | 1.792E-02 | 2.185E-03 | 0.8 |
| 18 | P2 | 249 | 10 (240,249) | 3.8 | 1.768E-02 | 1.861E-03 | 1.0 |
| 19 | P2 | 251 | 7 (244,251) | 2.7 | 1.756E-02 | 2.545E-03 | 0.8 |
| 20 | P2 | 257 | 8 (249,257) | 3.2 | 1.751E-02 | 2.135E-03 | 0.7 |
| 21 | P2 | 260 | 7 (253,260) | 2.5 | 1.705E-02 | 2.583E-03 | 1.0 |
| 22 | P2 | 266 | 8 (258,266) | 3.0 | 1.646E-02 | 2.084E-03 | 0.7 |
| 23 | P3 | 309 | 9 (300,309) | 3.0 | 1.393E-02 | 1.514E-03 | 0.8 |
| 24 | P3 | 312 | 8 (304,312) | 2.5 | 1.451E-02 | 1.837E-03 | 0.6 |
| 25 | P3 | 316 | 8 (308,316) | 2.4 | 1.496E-02 | 1.995E-03 | 0.6 |
| 26 | P3 | 319 | 6 (313,319) | 1.9 | 1.508E-02 | 2.432E-03 | 0.7 |
| 27 | P3 | 327 | 10 (317,327) | 3.0 | 1.527E-02 | 1.542E-03 | 0.7 |
| 28 | P3 | 330 | 9 (321,330) | 2.7 | 1.535E-02 | 1.706E-03 | 0.5 |
| 29 | P3 | 334 | 8 (326,334) | 2.5 | 1.511E-02 | 1.820E-03 | 0.5 |
| 30 | P3 | 338 | 8 (330,338) | 2.4 | 1.513E-02 | 1.891E-03 | 0.6 |
| 31 | P3 | 342 | 8 (334,342) | 2.2 | 1.510E-02 | 1.987E-03 | 0.8 |
| 32 | P3 | 350 | 11 (339,350) | 3.2 | 1.500E-02 | 1.327E-03 | 0.7 |
| 33 | P3 | 354 | 11 (343,354) | 3.1 | 1.495E-02 | 1.359E-03 | 0.6 |
| 34 | P3 | 358 | 11 (347,358) | 3.0 | 1.484E-02 | 1.387E-03 | 0.5 |
| 35 | P3 | 362 | 10 (352,362) | 2.9 | 1.485E-02 | 1.428E-03 | 0.5 |
| 36 | P3 | 366 | 10 (356,366) | 2.7 | 1.473E-02 | 1.473E-03 | 0.5 |
| 37 | P3 | 370 | 10 (360,370) | 2.6 | 1.469E-02 | 1.514E-03 | 0.5 |
| 38 | P4 | 429 | 14 (415,429) | 3.3 | 2.527E-02 | 1.805E-03 | 0.8 |
| 39 | P4 | 434 | 10 (424,434) | 2.3 | 2.558E-02 | 2.558E-03 | 0.6 |
| 40 | P4 | 444 | 15 (429,444) | 3.4 | 2.600E-02 | 1.733E-03 | 0.4 |
| 41 | P4 | 454 | 15 (439,454) | 3.3 | 2.580E-02 | 1.720E-03 | 0.5 |
| 42 | P4 | 465 | 16 (449,465) | 3.4 | 2.553E-02 | 1.596E-03 | 0.8 |
| 43 | P4 | 470 | 10 (460,470) | 2.1 | 2.511E-02 | 2.511E-03 | 0.5 |
| 44 | P4 | 481 | 16 (465,481) | 3.3 | 2.526E-02 | 1.579E-03 | 0.5 |
| 45 | P4 | 486 | 17 (475,492) | 3.5 | 2.438E-02 | 1.434E-03 | 0.8 |
| 46 | P5 | 551 | 18 (533,551) | 3.3 | 2.084E-02 | 1.158E-03 | 0.8 |
| 47 | P5 | 558 | 13 (545,558) | 2.3 | 2.211E-02 | 1.701E-03 | 0.5 |
| 48 | P5 | 564 | 13 (551,564) | 2.3 | 2.180E-02 | 1.677E-03 | 0.5 |
| 49 | P5 | 577 | 19 (558,577) | 3.3 | 2.240E-02 | 1.179E-03 | 0.9 |
| 50 | P5 | 584 | 14 (570,584) | 2.4 | 2.220E-02 | 1.586E-03 | 0.5 |
| 51 | P5 | 590 | 13 (577,590) | 2.2 | 2.173E-02 | 1.672E-03 | 0.5 |
| 52 | P5 | 597 | 20 (584,604) | 3.4 | 2.119E-02 | 1.060E-03 | 0.8 |
| 53 | P5 | 611 | 14 (597,611) | 2.3 | 2.141E-02 | 1.529E-03 | 0.6 |
| 54 | P5 | 618 | 14 (604,618) | 2.3 | 2.101E-02 | 1.501E-03 | 0.3 |
| 55 | P5 | 625 | 14 (611,625) | 2.2 | 2.041E-02 | 1.458E-03 | 0.5 |
| 56 | P6 | 684 | 15 (669,684) | 2.2 | 1.855E-02 | 1.237E-03 | 0.3 |
| 57 | P6 | 692 | 15 (677,692) | 2.2 | 1.964E-02 | 1.309E-03 | 0.4 |
| 58 | P6 | 700 | 24 (684,708) | 3.4 | 1.960E-02 | 8.167E-04 | 0.6 |
| 59 | P6 | 708 | 24 (692,716) | 3.4 | 1.999E-02 | 8.329E-04 | 0.8 |
| 60 | P6 | 724 | 16 (708,724) | 2.2 | 2.038E-02 | 1.274E-03 | 0.9 |
| 61 | P6 | 733 | 17 (716,733) | 2.3 | 1.987E-02 | 1.169E-03 | 0.7 |

| | | | | | | | |
|---|---|---|---|---|---|---|---|
| 62 | P6 | 741 | 17 (724,741) | 2.3 | 1.985E-02 | 1.168E-03 | 0.4 |
| 63 | P6 | 749 | 16 (733,749) | 2.1 | 1.931E-02 | 1.207E-03 | 0.2 |
| 64 | P6 | 758 | 17 (741,758) | 2.2 | 1.911E-02 | 1.124E-03 | 0.2 |
| 65 | P6 | 767 | 26 (749,775) | 3.4 | 1.884E-02 | 7.246E-04 | 0.2 |
| 66 | P7 | 837 | 22 (820,842) | 2.6 | 1.504E-02 | 6.931E-04 | 0.7 |
| 67 | P7 | 846 | 22 (829,851) | 2.6 | 1.612E-02 | 7.361E-04 | 0.8 |
| 68 | P7 | 855 | 22 (837,859) | 2.6 | 1.650E-02 | 7.500E-04 | 1.0 |
| 69 | P7 | 864 | 22 (846,868) | 2.6 | 1.630E-02 | 7.342E-04 | 1.1 |
| 70 | P7 | 873 | 22 (854,877) | 2.6 | 1.625E-02 | 7.222E-04 | 0.8 |
| 71 | P7 | 882 | 23 (863,886) | 2.6 | 1.646E-02 | 7.283E-04 | 0.8 |
| 72 | P7 | 891 | 23 (872,894) | 2.6 | 1.628E-02 | 7.140E-04 | 0.4 |
| 73 | P7 | 900 | 23 (880,903) | 2.6 | 1.592E-02 | 6.892E-04 | 0.4 |
| 74 | P7 | 909 | 23 (889,912) | 2.6 | 1.589E-02 | 6.849E-04 | 0.6 |
| 75 | P7 | 918 | 23 (897,921) | 2.5 | 1.558E-02 | 6.658E-04 | 0.8 |
| 76 | P7 | 927 | 24 (906,930) | 2.5 | 1.512E-02 | 6.407E-04 | 0.9 |
| 77 | P8 | 1002 | 25 (983,1008) | 2.5 | 1.325E-02 | 5.364E-04 | 0.8 |
| 78 | P8 | 1012 | 25 (992,1017) | 2.5 | 1.400E-02 | 5.600E-04 | 1.1 |
| 79 | P8 | 1021 | 25 (1001,1027) | 2.5 | 1.392E-02 | 5.480E-04 | 0.6 |
| 80 | P8 | 1031 | 26 (1010,1036) | 2.5 | 1.444E-02 | 5.619E-04 | 0.4 |
| 81 | P8 | 1040 | 26 (1019,1045) | 2.5 | 1.451E-02 | 5.581E-04 | 0.9 |
| 82 | P8 | 1050 | 26 (1028,1054) | 2.5 | 1.440E-02 | 5.496E-04 | 1.1 |
| 83 | P8 | 1059 | 26 (1037,1064) | 2.5 | 1.376E-02 | 5.192E-04 | 0.9 |
| 84 | P8 | 1069 | 27 (1046,1073) | 2.5 | 1.380E-02 | 5.149E-04 | 0.4 |
| 85 | P8 | 1078 | 27 (1055,1082) | 2.5 | 1.389E-02 | 5.107E-04 | 0.9 |
| 86 | P8 | 1088 | 28 (1064,1092) | 2.5 | 1.370E-02 | 4.982E-04 | 1.2 |

Table 4: Energy/Flux Calibration Factors for the Histogram Electron Channels. Histogram LUT ID (M35-A): 20738.

| CH # | PIX | $E$ [keV] | $\Delta E$ ($E_{lo}$,$E_{hi}$) [keV] | $\Delta E/E$ [%] | $G_0 \Delta E$ [cm$^2$ sr keV] | $G_0$ [cm$^2$ sr] | $\Delta G_0/G_0$ [%] |
|---|---|---|---|---|---|---|---|
| 0 | P1 | 135 | 10 (126,136) | 7.4 | 3.860E-02 | 3.860E-03 | 1.3 |
| 1 | P1 | 144 | 11 (135,146) | 7.6 | 3.940E-02 | 3.582E-03 | 1.3 |
| 2 | P1 | 152 | 12 (144,156) | 7.9 | 3.880E-02 | 3.233E-03 | 1.3 |
| 3 | P1 | 163 | 11 (154,165) | 6.7 | 3.880E-02 | 3.527E-03 | 1.3 |
| 4 | P1 | 173 | 12 (163,175) | 6.9 | 3.730E-02 | 3.108E-03 | 1.3 |
| 5 | P2 | 212 | 12 (200,212) | 5.5 | 3.374E-02 | 2.884E-03 | 0.9 |
| 6 | P2 | 222 | 12 (210,222) | 5.5 | 3.591E-02 | 2.920E-03 | 1.0 |
| 7 | P2 | 230 | 13 (219,232) | 5.7 | 3.581E-02 | 2.755E-03 | 0.9 |
| 8 | P2 | 240 | 13 (230,243) | 5.4 | 3.564E-02 | 2.742E-03 | 0.8 |
| 9 | P2 | 249 | 11 (240,251) | 4.4 | 3.501E-02 | 3.183E-03 | 0.9 |
| 10 | P2 | 260 | 11 (249,260) | 4.2 | 3.483E-02 | 3.166E-03 | 0.8 |
| 11 | P3 | 309 | 10 (298,309) | 3.3 | 2.822E-02 | 2.740E-03 | 0.7 |
| 12 | P3 | 319 | 14 (305,319) | 4.4 | 3.027E-02 | 2.162E-03 | 0.7 |
| 13 | P3 | 327 | 11 (316,327) | 3.4 | 3.038E-02 | 2.762E-03 | 0.6 |
| 14 | P3 | 338 | 15 (323,338) | 4.4 | 3.044E-02 | 2.029E-03 | 0.7 |
| 15 | P3 | 346 | 12 (334,346) | 3.5 | 3.017E-02 | 2.514E-03 | 0.5 |
| 16 | P3 | 354 | 16 (342,358) | 4.5 | 2.951E-02 | 1.844E-03 | 0.7 |
| 17 | P3 | 362 | 16 (350,366) | 4.4 | 2.930E-02 | 1.831E-03 | 0.8 |
| 18 | P3 | 374 | 12 (362,374) | 3.2 | 2.870E-02 | 2.392E-03 | 0.7 |
| 19 | P4 | 429 | 14 (415,429) | 3.3 | 2.527E-02 | 1.805E-03 | 0.8 |
| 20 | P4 | 434 | 10 (424,434) | 2.3 | 2.558E-02 | 2.558E-03 | 0.6 |
| 21 | P4 | 444 | 15 (429,444) | 3.4 | 2.600E-02 | 1.733E-03 | 0.4 |
| 22 | P4 | 454 | 15 (439,454) | 3.3 | 2.580E-02 | 1.720E-03 | 0.5 |
| 23 | P4 | 465 | 16 (449,465) | 3.4 | 2.553E-02 | 1.596E-03 | 0.8 |
| 24 | P4 | 470 | 10 (460,470) | 2.1 | 2.511E-02 | 2.511E-03 | 0.5 |
| 25 | P4 | 481 | 16 (465,481) | 3.3 | 2.526E-02 | 1.579E-03 | 0.5 |
| 26 | P4 | 486 | 17 (475,492) | 3.5 | 2.438E-02 | 1.434E-03 | 0.8 |
| 27 | P5 | 551 | 18 (533,551) | 3.3 | 2.084E-02 | 1.158E-03 | 0.8 |
| 28 | P5 | 558 | 13 (545,558) | 2.3 | 2.211E-02 | 1.701E-03 | 0.5 |
| 29 | P5 | 564 | 13 (551,564) | 2.3 | 2.180E-02 | 1.677E-03 | 0.5 |
| 30 | P5 | 577 | 19 (558,577) | 3.3 | 2.240E-02 | 1.179E-03 | 0.9 |
| 31 | P5 | 584 | 14 (570,584) | 2.4 | 2.220E-02 | 1.586E-03 | 0.5 |
| 32 | P5 | 590 | 13 (577,590) | 2.2 | 2.173E-02 | 1.672E-03 | 0.5 |
| 33 | P5 | 597 | 20 (584,604) | 3.4 | 2.119E-02 | 1.060E-03 | 0.8 |
| 34 | P5 | 611 | 14 (597,611) | 2.3 | 2.141E-02 | 1.529E-03 | 0.6 |

| | | | | | | | |
|---|---|---|---|---|---|---|---|
| 35 | P5 | 618 | 14 (604,618) | 2.3 | 2.101E-02 | 1.501E-03 | 0.3 |
| 36 | P5 | 625 | 14 (611,625) | 2.2 | 2.041E-02 | 1.458E-03 | 0.5 |
| 37 | P6 | 684 | 15 (669,684) | 2.2 | 1.855E-02 | 1.237E-03 | 0.3 |
| 38 | P6 | 692 | 15 (677,692) | 2.2 | 1.964E-02 | 1.309E-03 | 0.4 |
| 39 | P6 | 700 | 24 (684,708) | 3.4 | 1.960E-02 | 8.167E-04 | 0.6 |
| 40 | P6 | 708 | 24 (692,716) | 3.4 | 1.999E-02 | 8.329E-04 | 0.8 |
| 41 | P6 | 724 | 16 (708,724) | 2.2 | 2.038E-02 | 1.274E-03 | 0.9 |
| 42 | P6 | 733 | 17 (716,733) | 2.3 | 1.987E-02 | 1.169E-03 | 0.7 |
| 43 | P6 | 741 | 17 (724,741) | 2.3 | 1.985E-02 | 1.168E-03 | 0.4 |
| 44 | P6 | 749 | 16 (733,749) | 2.1 | 1.931E-02 | 1.207E-03 | 0.2 |
| 45 | P6 | 758 | 17 (741,758) | 2.2 | 1.911E-02 | 1.124E-03 | 0.2 |
| 46 | P6 | 767 | 26 (749,775) | 3.4 | 1.884E-02 | 7.246E-04 | 0.2 |
| 47 | P7 | 837 | 22 (820,842) | 2.6 | 1.504E-02 | 6.931E-04 | 0.7 |
| 48 | P7 | 846 | 22 (829,851) | 2.6 | 1.612E-02 | 7.361E-04 | 0.8 |
| 49 | P7 | 855 | 22 (837,859) | 2.6 | 1.650E-02 | 7.500E-04 | 1.0 |
| 50 | P7 | 864 | 22 (846,868) | 2.6 | 1.630E-02 | 7.342E-04 | 1.1 |
| 51 | P7 | 873 | 22 (854,877) | 2.6 | 1.625E-02 | 7.222E-04 | 0.8 |
| 52 | P7 | 882 | 23 (863,886) | 2.6 | 1.646E-02 | 7.283E-04 | 0.8 |
| 53 | P7 | 891 | 23 (872,894) | 2.6 | 1.628E-02 | 7.140E-04 | 0.4 |
| 54 | P7 | 900 | 23 (880,903) | 2.6 | 1.592E-02 | 6.892E-04 | 0.4 |
| 55 | P7 | 909 | 23 (889,912) | 2.6 | 1.589E-02 | 6.849E-04 | 0.6 |
| 56 | P7 | 918 | 23 (897,921) | 2.5 | 1.558E-02 | 6.658E-04 | 0.8 |
| 57 | P7 | 927 | 24 (906,930) | 2.5 | 1.512E-02 | 6.407E-04 | 0.9 |
| 58 | P8 | 1002 | 25 (983,1008) | 2.5 | 1.325E-02 | 5.364E-04 | 0.8 |
| 59 | P8 | 1012 | 25 (992,1017) | 2.5 | 1.400E-02 | 5.600E-04 | 1.1 |
| 60 | P8 | 1021 | 25 (1001,1027) | 2.5 | 1.392E-02 | 5.480E-04 | 0.6 |
| 61 | P8 | 1031 | 26 (1010,1036) | 2.5 | 1.444E-02 | 5.619E-04 | 0.4 |
| 62 | P8 | 1040 | 26 (1019,1045) | 2.5 | 1.451E-02 | 5.581E-04 | 0.9 |
| 63 | P8 | 1050 | 26 (1028,1054) | 2.5 | 1.440E-02 | 5.496E-04 | 1.1 |
| 64 | P8 | 1059 | 26 (1037,1064) | 2.5 | 1.376E-02 | 5.192E-04 | 0.9 |
| 65 | P8 | 1069 | 27 (1046,1073) | 2.5 | 1.380E-02 | 5.149E-04 | 0.4 |
| 66 | P8 | 1078 | 27 (1055,1082) | 2.5 | 1.389E-02 | 5.107E-04 | 0.9 |
| 67 | P8 | 1088 | 28 (1064,1092) | 2.5 | 1.370E-02 | 4.982E-04 | 1.2 |

**Probe A (M75)**

Table 5: Energy/Flux Calibration Factors for the Histogram Electron Channels. Histogram LUT ID (M75-A): 24833.

| CH # | PIX | $E$ [keV] | $\Delta E$ ($E_{lo}$,$E_{hi}$) [keV] | $\Delta E/E$ [%] | $G_0 \Delta E$ [cm$^2$ sr keV] | $G_0$ [cm$^2$ sr] | $\Delta G_0/G_0$ [%] |
|---|---|---|---|---|---|---|---|
| 0 | P1 | 131 | 6 (125,131) | 4.6 | 1.730E-02 | 2.883E-03 | 1.6 |
| 1 | P1 | 136 | 6 (130,136) | 4.4 | 1.860E-02 | 3.100E-03 | 1.6 |
| 2 | P1 | 139 | 6 (133,139) | 4.3 | 1.870E-02 | 3.117E-03 | 1.5 |
| 3 | P1 | 144 | 6 (138,144) | 4.2 | 1.890E-02 | 3.150E-03 | 1.3 |
| 4 | P1 | 149 | 7 (142,149) | 4.7 | 1.880E-02 | 2.686E-03 | 1.3 |
| 5 | P1 | 154 | 6 (148,154) | 3.9 | 1.890E-02 | 3.150E-03 | 1.4 |
| 6 | P1 | 158 | 7 (151,158) | 4.4 | 1.860E-02 | 2.657E-03 | 1.3 |
| 7 | P1 | 163 | 5 (158,163) | 3.1 | 1.870E-02 | 3.740E-03 | 1.3 |
| 8 | P1 | 167 | 7 (160,167) | 4.2 | 1.850E-02 | 2.643E-03 | 1.1 |
| 9 | P1 | 171 | 6 (165,171) | 3.3 | 1.810E-02 | 3.175E-03 | 1.3 |
| 10 | P1 | 177 | 6 (171,177) | 3.4 | 1.780E-02 | 2.967E-03 | 1.2 |
| 11 | P2 | 214 | 8 (206,214) | 3.5 | 1.630E-02 | 2.173E-03 | 1.0 |
| 12 | P2 | 219 | 8 (211,219) | 3.7 | 1.690E-02 | 2.112E-03 | 1.1 |
| 13 | P2 | 222 | 6 (216,222) | 2.8 | 1.738E-02 | 2.759E-03 | 1.0 |
| 14 | P2 | 227 | 7 (220,227) | 3.0 | 1.747E-02 | 2.569E-03 | 0.9 |
| 15 | P2 | 232 | 7 (225,232) | 3.1 | 1.752E-02 | 2.400E-03 | 0.9 |
| 16 | P2 | 238 | 9 (229,238) | 3.7 | 1.736E-02 | 1.995E-03 | 0.9 |
| 17 | P2 | 240 | 6 (234,240) | 2.6 | 1.720E-02 | 2.774E-03 | 1.1 |
| 18 | P2 | 246 | 7 (238,246) | 3.0 | 1.726E-02 | 2.332E-03 | 0.8 |
| 19 | P2 | 251 | 8 (243,251) | 3.2 | 1.731E-02 | 2.164E-03 | 0.8 |
| 20 | P2 | 254 | 6 (248,254) | 2.6 | 1.697E-02 | 2.611E-03 | 0.9 |
| 21 | P2 | 260 | 8 (252,260) | 3.0 | 1.685E-02 | 2.133E-03 | 0.7 |
| 22 | P2 | 266 | 9 (257,266) | 3.5 | 1.700E-02 | 1.828E-03 | 1.0 |
| 23 | P2 | 269 | 8 (261,269) | 2.9 | 1.649E-02 | 2.114E-03 | 0.7 |
| 24 | P3 | 319 | 8 (311,319) | 2.4 | 1.413E-02 | 1.835E-03 | 0.9 |
| 25 | P3 | 323 | 8 (315,323) | 2.4 | 1.448E-02 | 1.881E-03 | 0.8 |
| 26 | P3 | 327 | 8 (319,327) | 2.4 | 1.452E-02 | 1.886E-03 | 0.6 |
| 27 | P3 | 330 | 7 (323,330) | 2.2 | 1.488E-02 | 2.067E-03 | 0.5 |
| 28 | P3 | 334 | 7 (327,334) | 2.0 | 1.469E-02 | 2.160E-03 | 0.6 |
| 29 | P3 | 338 | 7 (331,338) | 2.0 | 1.458E-02 | 2.144E-03 | 0.8 |
| 30 | P3 | 346 | 11 (335,346) | 3.2 | 1.479E-02 | 1.357E-03 | 0.9 |
| 31 | P3 | 350 | 11 (339,350) | 3.1 | 1.465E-02 | 1.344E-03 | 0.7 |
| 32 | P3 | 354 | 11 (343,354) | 3.1 | 1.453E-02 | 1.333E-03 | 0.6 |
| 33 | P3 | 358 | 11 (347,358) | 3.1 | 1.440E-02 | 1.309E-03 | 0.5 |
| 34 | P3 | 362 | 11 (351,362) | 3.0 | 1.431E-02 | 1.301E-03 | 0.5 |
| 35 | P3 | 366 | 11 (355,366) | 3.0 | 1.397E-02 | 1.270E-03 | 0.5 |
| 36 | P3 | 370 | 11 (359,370) | 3.0 | 1.424E-02 | 1.295E-03 | 0.5 |
| 37 | P3 | 374 | 11 (363,374) | 3.0 | 1.425E-02 | 1.284E-03 | 0.5 |
| 38 | P3 | 379 | 12 (367,379) | 3.2 | 1.383E-02 | 1.143E-03 | 0.5 |
| 39 | P4 | 439 | 15 (424,439) | 3.4 | 2.487E-02 | 1.658E-03 | 0.5 |
| 40 | P4 | 449 | 15 (434,449) | 3.3 | 2.575E-02 | 1.717E-03 | 0.7 |
| 41 | P4 | 454 | 10 (444,454) | 2.2 | 2.577E-02 | 2.577E-03 | 0.6 |
| 42 | P4 | 465 | 16 (449,465) | 3.4 | 2.556E-02 | 1.597E-03 | 0.4 |
| 43 | P4 | 475 | 15 (460,475) | 3.2 | 2.560E-02 | 1.707E-03 | 0.6 |
| 44 | P4 | 481 | 11 (470,481) | 2.3 | 2.509E-02 | 2.281E-03 | 0.6 |
| 45 | P4 | 492 | 17 (475,492) | 3.5 | 2.482E-02 | 1.460E-03 | 0.5 |
| 46 | P4 | 498 | 17 (486,503) | 3.4 | 2.403E-02 | 1.414E-03 | 0.9 |
| 47 | P4 | 509 | 11 (498,509) | 2.2 | 2.382E-02 | 2.165E-03 | 0.4 |
| 48 | P5 | 564 | 13 (551,564) | 2.3 | 2.166E-02 | 1.666E-03 | 0.8 |
| 49 | P5 | 577 | 19 (558,577) | 3.3 | 2.294E-02 | 1.207E-03 | 0.6 |
| 50 | P5 | 584 | 14 (570,584) | 2.4 | 2.275E-02 | 1.625E-03 | 0.4 |
| 51 | P5 | 590 | 20 (577,597) | 3.4 | 2.273E-02 | 1.136E-03 | 0.9 |
| 52 | P5 | 604 | 14 (590,604) | 2.3 | 2.255E-02 | 1.611E-03 | 0.6 |
| 53 | P5 | 611 | 14 (597,611) | 2.3 | 2.231E-02 | 1.594E-03 | 0.5 |
| 54 | P5 | 618 | 21 (604,625) | 3.4 | 2.191E-02 | 1.043E-03 | 0.7 |
| 55 | P5 | 632 | 21 (611,632) | 3.3 | 2.202E-02 | 1.049E-03 | 0.8 |
| 56 | P5 | 639 | 14 (625,639) | 2.2 | 2.124E-02 | 1.517E-03 | 0.4 |
| 57 | P6 | 708 | 16 (692,708) | 2.3 | 1.895E-02 | 1.184E-03 | 0.4 |
| 58 | P6 | 716 | 24 (700,724) | 3.4 | 1.949E-02 | 8.121E-04 | 0.6 |
| 59 | P6 | 724 | 25 (708,733) | 3.5 | 1.973E-02 | 7.892E-04 | 0.8 |
| 60 | P6 | 733 | 25 (716,741) | 3.4 | 1.981E-02 | 7.924E-04 | 0.9 |
| 61 | P6 | 749 | 16 (733,749) | 2.1 | 1.997E-02 | 1.248E-03 | 0.9 |

| | | | | | | | |
|---|---|---|---|---|---|---|---|
| 62 | P6 | 758 | 17 (741,758) | 2.2 | 1.976E-02 | 1.162E-03 | 0.7 |
| 63 | P6 | 767 | 18 (749,767) | 2.3 | 1.937E-02 | 1.076E-03 | 0.5 |
| 64 | P6 | 775 | 17 (758,775) | 2.2 | 1.923E-02 | 1.131E-03 | 0.3 |
| 65 | P6 | 784 | 17 (767,784) | 2.2 | 1.891E-02 | 1.112E-03 | 0.3 |
| 66 | P6 | 793 | 18 (775,793) | 2.3 | 1.801E-02 | 1.001E-03 | 0.3 |
| 67 | P7 | 861 | 22 (842,864) | 2.6 | 1.457E-02 | 6.623E-04 | 0.8 |
| 68 | P7 | 870 | 22 (851,873) | 2.6 | 1.541E-02 | 6.941E-04 | 0.6 |
| 69 | P7 | 879 | 23 (859,882) | 2.6 | 1.591E-02 | 7.040E-04 | 0.4 |
| 70 | P7 | 888 | 23 (868,891) | 2.6 | 1.563E-02 | 6.855E-04 | 0.5 |
| 71 | P7 | 897 | 23 (877,900) | 2.6 | 1.583E-02 | 6.883E-04 | 0.6 |
| 72 | P7 | 906 | 23 (886,909) | 2.6 | 1.581E-02 | 6.785E-04 | 0.9 |
| 73 | P7 | 915 | 24 (894,918) | 2.6 | 1.560E-02 | 6.638E-04 | 1.1 |
| 74 | P7 | 924 | 24 (903,927) | 2.6 | 1.548E-02 | 6.532E-04 | 0.8 |
| 75 | P7 | 933 | 24 (912,936) | 2.6 | 1.518E-02 | 6.299E-04 | 0.5 |
| 76 | P7 | 942 | 24 (921,945) | 2.6 | 1.480E-02 | 6.091E-04 | 0.5 |
| 77 | P7 | 951 | 24 (930,954) | 2.6 | 1.494E-02 | 6.098E-04 | 0.8 |
| 78 | P8 | 1021 | 23 (998,1022) | 2.3 | 1.284E-02 | 5.583E-04 | 0.5 |
| 79 | P8 | 1030 | 24 (1007,1031) | 2.3 | 1.349E-02 | 5.740E-04 | 0.8 |
| 80 | P8 | 1038 | 24 (1016,1040) | 2.3 | 1.347E-02 | 5.660E-04 | 0.9 |
| 81 | P8 | 1047 | 24 (1025,1049) | 2.3 | 1.316E-02 | 5.416E-04 | 1.0 |
| 82 | P8 | 1056 | 25 (1034,1059) | 2.3 | 1.344E-02 | 5.441E-04 | 0.4 |
| 83 | P8 | 1064 | 25 (1043,1068) | 2.4 | 1.332E-02 | 5.307E-04 | 0.9 |
| 84 | P8 | 1073 | 26 (1052,1077) | 2.4 | 1.350E-02 | 5.294E-04 | 1.2 |
| 85 | P8 | 1082 | 26 (1060,1086) | 2.4 | 1.310E-02 | 5.058E-04 | 0.7 |
| 86 | P8 | 1090 | 26 (1069,1096) | 2.4 | 1.304E-02 | 4.958E-04 | 0.6 |
| 87 | P8 | 1099 | 27 (1078,1105) | 2.4 | 1.310E-02 | 4.888E-04 | 1.3 |
| 88 | P8 | 1108 | 27 (1087,1114) | 2.4 | 1.220E-02 | 4.502E-04 | 1.1 |

Table 6: Energy/Flux Calibration Factors for the Histogram Electron Channels. Histogram LUT ID (M75-A): 24834.

| CH # | PIX | $E$ [keV] | $\Delta E$ ($E_{lo}$,$E_{hi}$) [keV] | $\Delta E/E$ [%] | $G_0\Delta E$ [$cm^2$ sr keV] | $G_0$ [$cm^2$ sr] | $\Delta G_0/G_0$ [%] |
|---|---|---|---|---|---|---|---|
| 0 | P1 | 133 | 11 (124,135) | 8.3 | 3.560E-02 | 3.236E-03 | 1.5 |
| 1 | P1 | 142 | 11 (133,144) | 7.7 | 3.780E-02 | 3.436E-03 | 1.4 |
| 2 | P1 | 151 | 10 (142,152) | 6.6 | 3.740E-02 | 3.740E-03 | 1.3 |
| 3 | P1 | 160 | 10 (151,161) | 6.2 | 3.710E-02 | 3.710E-03 | 1.3 |
| 4 | P1 | 169 | 11 (160,171) | 6.5 | 3.660E-02 | 3.327E-03 | 1.2 |
| 5 | P1 | 179 | 12 (169,181) | 6.7 | 3.410E-02 | 2.842E-03 | 1.2 |
| 6 | P2 | 217 | 12 (205,217) | 5.5 | 3.310E-02 | 2.758E-03 | 1.1 |
| 7 | P2 | 224 | 13 (214,227) | 5.8 | 3.488E-02 | 2.683E-03 | 0.9 |
| 8 | P2 | 235 | 11 (224,235) | 4.7 | 3.490E-02 | 3.173E-03 | 0.9 |
| 9 | P2 | 243 | 14 (232,246) | 5.8 | 3.449E-02 | 2.464E-03 | 0.9 |
| 10 | P2 | 254 | 11 (243,254) | 4.3 | 3.450E-02 | 3.136E-03 | 1.0 |
| 11 | P2 | 263 | 12 (251,263) | 4.6 | 3.380E-02 | 2.817E-03 | 0.8 |
| 12 | P2 | 272 | 12 (260,272) | 4.4 | 3.187E-02 | 2.656E-03 | 0.8 |
| 13 | P3 | 319 | 10 (309,319) | 3.1 | 2.842E-02 | 2.842E-03 | 0.6 |
| 14 | P3 | 330 | 15 (316,330) | 4.4 | 2.958E-02 | 2.012E-03 | 0.9 |
| 15 | P3 | 338 | 11 (327,338) | 3.3 | 2.952E-02 | 2.612E-03 | 0.6 |
| 16 | P3 | 346 | 12 (334,346) | 3.5 | 2.924E-02 | 2.437E-03 | 0.6 |
| 17 | P3 | 354 | 12 (342,354) | 3.3 | 2.867E-02 | 2.430E-03 | 0.7 |
| 18 | P3 | 362 | 16 (350,366) | 4.4 | 2.799E-02 | 1.749E-03 | 0.9 |
| 19 | P3 | 374 | 12 (362,374) | 3.2 | 2.866E-02 | 2.388E-03 | 0.8 |
| 20 | P3 | 383 | 13 (370,383) | 3.4 | 2.699E-02 | 2.076E-03 | 0.8 |
| 21 | P4 | 439 | 15 (424,439) | 3.4 | 2.487E-02 | 1.658E-03 | 0.5 |
| 22 | P4 | 449 | 15 (434,449) | 3.3 | 2.575E-02 | 1.717E-03 | 0.7 |
| 23 | P4 | 454 | 10 (444,454) | 2.2 | 2.577E-02 | 2.577E-03 | 0.6 |
| 24 | P4 | 465 | 16 (449,465) | 3.4 | 2.556E-02 | 1.597E-03 | 0.4 |
| 25 | P4 | 475 | 15 (460,475) | 3.2 | 2.560E-02 | 1.707E-03 | 0.6 |
| 26 | P4 | 481 | 11 (470,481) | 2.3 | 2.509E-02 | 2.281E-03 | 0.6 |
| 27 | P4 | 492 | 17 (475,492) | 3.5 | 2.482E-02 | 1.460E-03 | 0.5 |
| 28 | P4 | 498 | 17 (486,503) | 3.4 | 2.403E-02 | 1.414E-03 | 0.9 |
| 29 | P4 | 509 | 11 (498,509) | 2.2 | 2.382E-02 | 2.165E-03 | 0.4 |
| 30 | P5 | 564 | 13 (551,564) | 2.3 | 2.166E-02 | 1.666E-03 | 0.8 |
| 31 | P5 | 577 | 19 (558,577) | 3.3 | 2.294E-02 | 1.207E-03 | 0.6 |
| 32 | P5 | 584 | 14 (570,584) | 2.4 | 2.275E-02 | 1.625E-03 | 0.4 |

| | | | | | | | |
|---|---|---|---|---|---|---|---|
| 33 | P5 | 590 | 20 (577,597) | 3.4 | 2.273E-02 | 1.136E-03 | 0.9 |
| 34 | P5 | 604 | 14 (590,604) | 2.3 | 2.255E-02 | 1.611E-03 | 0.6 |
| 35 | P5 | 611 | 14 (597,611) | 2.3 | 2.231E-02 | 1.594E-03 | 0.5 |
| 36 | P5 | 618 | 21 (604,625) | 3.4 | 2.191E-02 | 1.043E-03 | 0.7 |
| 37 | P5 | 632 | 21 (611,632) | 3.3 | 2.202E-02 | 1.049E-03 | 0.8 |
| 38 | P5 | 639 | 14 (625,639) | 2.2 | 2.124E-02 | 1.517E-03 | 0.4 |
| 39 | P6 | 708 | 16 (692,708) | 2.3 | 1.895E-02 | 1.184E-03 | 0.4 |
| 40 | P6 | 716 | 24 (700,724) | 3.4 | 1.949E-02 | 8.121E-04 | 0.6 |
| 41 | P6 | 724 | 25 (708,733) | 3.5 | 1.973E-02 | 7.892E-04 | 0.8 |
| 42 | P6 | 733 | 25 (716,741) | 3.4 | 1.981E-02 | 7.924E-04 | 0.9 |
| 43 | P6 | 749 | 16 (733,749) | 2.1 | 1.997E-02 | 1.248E-03 | 0.9 |
| 44 | P6 | 758 | 17 (741,758) | 2.2 | 1.976E-02 | 1.162E-03 | 0.7 |
| 45 | P6 | 767 | 18 (749,767) | 2.3 | 1.937E-02 | 1.076E-03 | 0.5 |
| 46 | P6 | 775 | 17 (758,775) | 2.2 | 1.923E-02 | 1.131E-03 | 0.3 |
| 47 | P6 | 784 | 17 (767,784) | 2.2 | 1.891E-02 | 1.112E-03 | 0.3 |
| 48 | P6 | 793 | 18 (775,793) | 2.3 | 1.801E-02 | 1.001E-03 | 0.3 |
| 49 | P7 | 861 | 22 (842,864) | 2.6 | 1.457E-02 | 6.623E-04 | 0.8 |
| 50 | P7 | 870 | 22 (851,873) | 2.6 | 1.541E-02 | 6.941E-04 | 0.6 |
| 51 | P7 | 879 | 23 (859,882) | 2.6 | 1.591E-02 | 7.040E-04 | 0.4 |
| 52 | P7 | 888 | 23 (868,891) | 2.6 | 1.563E-02 | 6.855E-04 | 0.5 |
| 53 | P7 | 897 | 23 (877,900) | 2.6 | 1.583E-02 | 6.883E-04 | 0.6 |
| 54 | P7 | 906 | 23 (886,909) | 2.6 | 1.581E-02 | 6.785E-04 | 0.9 |
| 55 | P7 | 915 | 24 (894,918) | 2.6 | 1.560E-02 | 6.638E-04 | 1.1 |
| 56 | P7 | 924 | 24 (903,927) | 2.6 | 1.548E-02 | 6.532E-04 | 0.8 |
| 57 | P7 | 933 | 24 (912,936) | 2.6 | 1.518E-02 | 6.299E-04 | 0.5 |
| 58 | P7 | 942 | 24 (921,945) | 2.6 | 1.480E-02 | 6.091E-04 | 0.5 |
| 59 | P7 | 951 | 24 (930,954) | 2.6 | 1.494E-02 | 6.098E-04 | 0.8 |
| 60 | P8 | 1021 | 23 (998,1022) | 2.3 | 1.284E-02 | 5.583E-04 | 0.5 |
| 61 | P8 | 1030 | 24 (1007,1031) | 2.3 | 1.349E-02 | 5.740E-04 | 0.8 |
| 62 | P8 | 1038 | 24 (1016,1040) | 2.3 | 1.347E-02 | 5.660E-04 | 0.9 |
| 63 | P8 | 1047 | 24 (1025,1049) | 2.3 | 1.316E-02 | 5.416E-04 | 1.0 |
| 64 | P8 | 1056 | 25 (1034,1059) | 2.3 | 1.344E-02 | 5.441E-04 | 0.4 |
| 65 | P8 | 1064 | 25 (1043,1068) | 2.4 | 1.332E-02 | 5.307E-04 | 0.9 |
| 66 | P8 | 1073 | 26 (1052,1077) | 2.4 | 1.350E-02 | 5.294E-04 | 1.2 |
| 67 | P8 | 1082 | 26 (1060,1086) | 2.4 | 1.310E-02 | 5.058E-04 | 0.7 |
| 68 | P8 | 1090 | 26 (1069,1096) | 2.4 | 1.304E-02 | 4.958E-04 | 0.6 |
| 69 | P8 | 1099 | 27 (1078,1105) | 2.4 | 1.310E-02 | 4.888E-04 | 1.3 |
| 70 | P8 | 1108 | 27 (1087,1114) | 2.4 | 1.220E-02 | 4.502E-04 | 1.1 |

## Probe A (HIGH)

Table 7: Energy/Flux Calibration Factors for the Histogram Electron Channels. Histogram LUT ID (HIGH-A): 29442, for times between 2012/09/01 and 2013/08/03.

| CH # | PIX | $E$ [keV] | $\Delta E$ ($E_{lo}$,$E_{hi}$) [keV] | $\Delta E/E$ [%] | $G_0\Delta E$ [cm$^2$ sr keV] | $G_0$ [cm$^2$ sr] | $\Delta G_0/G_0$ [%] |
|---|---|---|---|---|---|---|---|
| 0 | P0 | 970 | 152 (844,996) | 15.7 | 1.020E-01 | 6.689E-04 | 4.3 |
| 1 | P0 | 992 | 140 (884,1024) | 14.1 | 1.050E-01 | 7.527E-04 | 3.9 |
| 2 | P0 | 1016 | 146 (905,1050) | 14.3 | 1.110E-01 | 7.629E-04 | 3.4 |
| 3 | P0 | 1039 | 130 (948,1078) | 12.5 | 1.200E-01 | 9.266E-04 | 3.7 |
| 4 | P0 | 1088 | 134 (970,1104) | 12.4 | 1.380E-01 | 1.026E-03 | 3.0 |
| 5 | P0 | 1114 | 116 (1016,1132) | 10.4 | 1.270E-01 | 1.100E-03 | 2.6 |
| 6 | P0 | 1140 | 119 (1039,1158) | 10.5 | 1.270E-01 | 1.064E-03 | 1.9 |
| 7 | P0 | 1167 | 97 (1088,1185) | 8.3 | 1.160E-01 | 1.191E-03 | 2.1 |
| 8 | P0 | 1194 | 98 (1114,1212) | 8.2 | 1.110E-01 | 1.128E-03 | 2.5 |
| 9 | P0 | 1250 | 72 (1178,1250) | 5.8 | 1.140E-01 | 1.575E-03 | 2.7 |
| 10 | P0 | 1279 | 72 (1207,1279) | 5.7 | 1.050E-01 | 1.450E-03 | 1.8 |
| 11 | P1 | 1576 | 219 (1407,1626) | 13.9 | 5.450E-02 | 2.491E-04 | 2.7 |
| 12 | P1 | 1607 | 205 (1446,1650) | 12.7 | 6.210E-02 | 3.035E-04 | 1.9 |
| 13 | P1 | 1637 | 190 (1485,1675) | 11.6 | 6.080E-02 | 3.192E-04 | 2.3 |
| 14 | P1 | 1668 | 176 (1524,1700) | 10.6 | 6.060E-02 | 3.437E-04 | 2.1 |
| 15 | P1 | 1699 | 162 (1563,1725) | 9.5 | 6.280E-02 | 3.872E-04 | 1.2 |
| 16 | P1 | 1730 | 148 (1601,1750) | 8.6 | 6.420E-02 | 4.335E-04 | 1.4 |
| 17 | P1 | 1760 | 134 (1640,1774) | 7.6 | 6.350E-02 | 4.739E-04 | 2.2 |
| 18 | P1 | 1791 | 120 (1679,1799) | 6.7 | 5.800E-02 | 4.841E-04 | 2.1 |
| 19 | P1 | 1822 | 106 (1718,1824) | 5.8 | 4.760E-02 | 4.503E-04 | 1.1 |
| 20 | P2 | 2218 | 223 (2012,2235) | 10.1 | 7.690E-02 | 3.450E-04 | 2.2 |
| 21 | P2 | 2251 | 220 (2048,2268) | 9.8 | 8.460E-02 | 3.851E-04 | 1.9 |
| 22 | P2 | 2285 | 216 (2084,2301) | 9.5 | 8.470E-02 | 3.914E-04 | 1.9 |
| 23 | P2 | 2318 | 213 (2121,2334) | 9.2 | 8.930E-02 | 4.189E-04 | 2.1 |
| 24 | P2 | 2352 | 210 (2157,2367) | 8.9 | 9.870E-02 | 4.702E-04 | 1.5 |
| 25 | P2 | 2386 | 207 (2193,2400) | 8.7 | 9.120E-02 | 4.414E-04 | 1.8 |
| 26 | P2 | 2419 | 203 (2230,2433) | 8.4 | 9.070E-02 | 4.459E-04 | 1.4 |
| 27 | P2 | 2453 | 200 (2266,2466) | 8.2 | 9.700E-02 | 4.848E-04 | 1.6 |
| 28 | P2 | 2486 | 197 (2302,2499) | 7.9 | 9.930E-02 | 5.046E-04 | 2.2 |
| 29 | P2 | 2520 | 194 (2338,2532) | 7.7 | 9.720E-02 | 5.021E-04 | 1.6 |
| 30 | P2 | 2554 | 190 (2375,2565) | 7.4 | 9.980E-02 | 5.247E-04 | 1.7 |
| 31 | P2 | 2587 | 187 (2411,2598) | 7.2 | 9.020E-02 | 4.824E-04 | 1.7 |
| 32 | P2 | 2621 | 184 (2447,2631) | 7.0 | 8.820E-02 | 4.801E-04 | 1.5 |
| 33 | P2 | 2654 | 180 (2483,2664) | 6.8 | 8.960E-02 | 4.967E-04 | 2.2 |
| 34 | P2 | 2688 | 177 (2520,2697) | 6.6 | 8.710E-02 | 4.915E-04 | 1.4 |
| 35 | P2 | 2722 | 174 (2556,2730) | 6.4 | 9.260E-02 | 5.325E-04 | 2.2 |
| 36 | P2 | 2755 | 171 (2592,2763) | 6.2 | 7.510E-02 | 4.402E-04 | 1.9 |
| 37 | P2 | 2789 | 167 (2628,2796) | 6.0 | 8.270E-02 | 4.940E-04 | 2.4 |
| 38 | P2 | 2822 | 164 (2665,2829) | 5.8 | 6.750E-02 | 4.113E-04 | 2.0 |
| 39 | P2 | 2856 | 161 (2701,2862) | 5.6 | 7.050E-02 | 4.382E-04 | 1.8 |
| 40 | P3 | 3723 | 353 (3472,3825) | 9.5 | 1.410E-01 | 3.999E-04 | 4.7 |
| 41 | P3 | 3759 | 346 (3510,3855) | 9.2 | 1.310E-01 | 3.791E-04 | 4.1 |
| 42 | P3 | 3794 | 338 (3547,3886) | 8.9 | 1.320E-01 | 3.900E-04 | 3.8 |
| 43 | P3 | 3830 | 331 (3585,3916) | 8.7 | 1.270E-01 | 3.833E-04 | 4.6 |
| 44 | P3 | 3865 | 324 (3622,3946) | 8.4 | 1.490E-01 | 4.596E-04 | 3.7 |
| 45 | P3 | 3901 | 317 (3659,3976) | 8.1 | 1.400E-01 | 4.415E-04 | 3.8 |
| 46 | P3 | 3936 | 310 (3697,4007) | 7.9 | 1.470E-01 | 4.742E-04 | 4.9 |
| 47 | P3 | 3972 | 303 (3734,4037) | 7.6 | 1.680E-01 | 5.546E-04 | 3.3 |
| 48 | P3 | 4007 | 296 (3772,4067) | 7.4 | 1.360E-01 | 4.599E-04 | 3.7 |
| 49 | P3 | 4043 | 289 (3809,4098) | 7.1 | 1.660E-01 | 5.750E-04 | 4.1 |
| 50 | P3 | 4078 | 282 (3846,4128) | 6.9 | 1.390E-01 | 4.938E-04 | 3.3 |
| 51 | P3 | 4114 | 274 (3884,4158) | 6.7 | 1.670E-01 | 6.086E-04 | 4.9 |
| 52 | P3 | 4149 | 267 (3921,4188) | 6.4 | 1.490E-01 | 5.574E-04 | 3.6 |
| 53 | P3 | 4185 | 260 (3958,4218) | 6.2 | 1.350E-01 | 5.188E-04 | 3.5 |
| 54 | P3 | 4220 | 253 (3996,4249) | 6.0 | 1.520E-01 | 6.006E-04 | 5.0 |
| 55 | P3 | 4256 | 246 (4033,4279) | 5.8 | 1.400E-01 | 5.693E-04 | 2.8 |
| 56 | P3 | 4291 | 239 (4070,4309) | 5.6 | 1.150E-01 | 4.816E-04 | 5.1 |
| 57 | P3 | 4327 | 232 (4108,4340) | 5.4 | 1.390E-01 | 5.997E-04 | 4.3 |
| 58 | P3 | 4362 | 225 (4145,4370) | 5.1 | 1.280E-01 | 5.699E-04 | 3.4 |
| 59 | P3 | 4398 | 218 (4183,4400) | 4.9 | 1.150E-01 | 5.287E-04 | 5.1 |
| 60 | P3 | 4433 | 210 (4223,4433) | 4.7 | 1.280E-01 | 6.087E-04 | 3.0 |

| | | | | | | | |
|---|---|---|---|---|---|---|---|
| 61 | P3 | 4469 | 203 (4266,4469) | 4.5 | 1.040E-01 | 5.118E-04 | 3.5 |
| 62 | P3 | 4504 | 196 (4308,4504) | 4.4 | 1.180E-01 | 6.017E-04 | 5.3 |

Table 8: Energy/Flux Calibration Factors for the Histogram Electron Channels. Histogram LUT ID (HIGH-A): 29442, for times between 2013/08/03 and 2019/10/14.

| CH # | PIX | $E$ [keV] | $\Delta E$ ($E_{lo}$,$E_{hi}$) [keV] | $\Delta E/E$ [%] | $G_0 \Delta E$ [cm$^2$ sr keV] | $G_0$ [cm$^2$ sr] | $\Delta G_0/G_0$ [%] |
|---|---|---|---|---|---|---|---|
| 0 | P0 | 970 | 91 (884,975) | 9.4 | 2.400E-01 | 2.646E-03 | 2.5 |
| 1 | P0 | 992 | 82 (926,1008) | 8.2 | 2.520E-01 | 3.088E-03 | 3.1 |
| 2 | P0 | 1039 | 93 (948,1041) | 8.9 | 3.030E-01 | 3.272E-03 | 2.8 |
| 3 | P0 | 1064 | 82 (992,1074) | 7.7 | 3.000E-01 | 3.676E-03 | 1.6 |
| 4 | P0 | 1088 | 91 (1016,1107) | 8.3 | 3.050E-01 | 3.366E-03 | 1.6 |
| 5 | P0 | 1114 | 76 (1064,1140) | 6.8 | 2.870E-01 | 3.796E-03 | 2.5 |
| 6 | P0 | 1167 | 85 (1088,1173) | 7.2 | 3.120E-01 | 3.688E-03 | 2.1 |
| 7 | P0 | 1194 | 92 (1114,1206) | 7.7 | 2.920E-01 | 3.188E-03 | 1.0 |
| 8 | P0 | 1222 | 72 (1167,1239) | 5.9 | 2.530E-01 | 3.534E-03 | 1.3 |
| 9 | P0 | 1250 | 78 (1194,1272) | 6.2 | 2.260E-01 | 2.916E-03 | 2.3 |
| 10 | P1 | 1480 | 161 (1340,1501) | 10.9 | 1.380E-01 | 8.582E-04 | 2.4 |
| 11 | P1 | 1511 | 156 (1375,1530) | 10.3 | 1.450E-01 | 9.325E-04 | 2.3 |
| 12 | P1 | 1542 | 150 (1409,1559) | 9.7 | 1.690E-01 | 1.126E-03 | 1.6 |
| 13 | P1 | 1573 | 145 (1443,1588) | 9.2 | 1.840E-01 | 1.271E-03 | 1.9 |
| 14 | P1 | 1604 | 139 (1477,1616) | 8.7 | 1.900E-01 | 1.363E-03 | 2.3 |
| 15 | P1 | 1635 | 134 (1511,1645) | 8.2 | 1.880E-01 | 1.403E-03 | 1.8 |
| 16 | P1 | 1666 | 129 (1545,1674) | 7.7 | 1.820E-01 | 1.414E-03 | 1.4 |
| 17 | P1 | 1697 | 123 (1580,1703) | 7.3 | 1.869E-01 | 1.516E-03 | 0.9 |
| 18 | P1 | 1728 | 118 (1614,1732) | 6.8 | 1.820E-01 | 1.545E-03 | 1.1 |
| 19 | P1 | 1759 | 112 (1648,1760) | 6.4 | 1.680E-01 | 1.493E-03 | 1.5 |
| 20 | P1 | 1790 | 107 (1683,1790) | 6.0 | 1.510E-01 | 1.409E-03 | 1.8 |
| 21 | P2 | 2309 | 136 (2173,2309) | 5.9 | 1.770E-01 | 1.301E-03 | 1.7 |
| 22 | P2 | 2344 | 138 (2206,2344) | 5.9 | 1.780E-01 | 1.295E-03 | 1.7 |
| 23 | P2 | 2378 | 139 (2239,2378) | 5.8 | 1.940E-01 | 1.395E-03 | 1.5 |
| 24 | P2 | 2413 | 141 (2272,2413) | 5.8 | 1.870E-01 | 1.329E-03 | 2.1 |
| 25 | P2 | 2447 | 142 (2305,2447) | 5.8 | 2.030E-01 | 1.427E-03 | 1.2 |
| 26 | P2 | 2482 | 144 (2338,2482) | 5.8 | 2.310E-01 | 1.606E-03 | 2.1 |
| 27 | P2 | 2516 | 145 (2371,2516) | 5.8 | 2.060E-01 | 1.418E-03 | 1.3 |
| 28 | P2 | 2551 | 147 (2404,2551) | 5.8 | 2.180E-01 | 1.484E-03 | 1.6 |
| 29 | P2 | 2585 | 148 (2437,2585) | 5.7 | 1.850E-01 | 1.246E-03 | 2.3 |
| 30 | P2 | 2620 | 150 (2470,2620) | 5.7 | 2.200E-01 | 1.466E-03 | 1.3 |
| 31 | P2 | 2654 | 152 (2502,2654) | 5.7 | 2.200E-01 | 1.451E-03 | 2.4 |
| 32 | P2 | 2689 | 153 (2536,2689) | 5.7 | 1.960E-01 | 1.280E-03 | 1.2 |
| 33 | P2 | 2723 | 155 (2568,2723) | 5.7 | 2.040E-01 | 1.319E-03 | 1.9 |
| 34 | P2 | 2758 | 156 (2601,2758) | 5.7 | 1.790E-01 | 1.145E-03 | 1.9 |
| 35 | P2 | 2792 | 158 (2634,2792) | 5.7 | 1.840E-01 | 1.165E-03 | 1.5 |
| 36 | P2 | 2826 | 159 (2667,2826) | 5.6 | 1.550E-01 | 9.730E-04 | 2.4 |
| 37 | P2 | 2861 | 161 (2700,2861) | 5.6 | 1.710E-01 | 1.063E-03 | 1.3 |
| 38 | P3 | 3789 | 293 (3536,3829) | 7.7 | 1.160E-01 | 3.963E-04 | 5.4 |
| 39 | P3 | 3825 | 290 (3574,3865) | 7.6 | 1.450E-01 | 4.995E-04 | 5.1 |
| 40 | P3 | 3862 | 288 (3612,3900) | 7.5 | 1.370E-01 | 4.757E-04 | 3.9 |
| 41 | P3 | 3898 | 286 (3650,3936) | 7.3 | 1.280E-01 | 4.480E-04 | 4.1 |
| 42 | P3 | 3935 | 283 (3688,3972) | 7.2 | 1.400E-01 | 4.940E-04 | 3.9 |
| 43 | P3 | 3971 | 281 (3726,4007) | 7.1 | 1.490E-01 | 5.301E-04 | 3.3 |
| 44 | P3 | 4008 | 279 (3764,4043) | 7.0 | 1.380E-01 | 4.950E-04 | 5.2 |
| 45 | P3 | 4044 | 276 (3802,4078) | 6.8 | 1.580E-01 | 5.716E-04 | 3.6 |
| 46 | P3 | 4081 | 274 (3840,4114) | 6.7 | 1.420E-01 | 5.179E-04 | 3.6 |
| 47 | P3 | 4117 | 272 (3878,4150) | 6.6 | 1.600E-01 | 5.887E-04 | 4.3 |
| 48 | P3 | 4154 | 270 (3916,4186) | 6.5 | 1.570E-01 | 5.823E-04 | 3.5 |
| 49 | P3 | 4190 | 267 (3954,4221) | 6.4 | 1.340E-01 | 5.015E-04 | 4.9 |
| 50 | P3 | 4226 | 265 (3992,4257) | 6.3 | 1.590E-01 | 6.002E-04 | 4.5 |
| 51 | P3 | 4263 | 263 (4030,4292) | 6.2 | 1.370E-01 | 5.217E-04 | 3.3 |
| 52 | P3 | 4299 | 260 (4068,4328) | 6.1 | 1.540E-01 | 5.916E-04 | 5.0 |
| 53 | P3 | 4336 | 258 (4106,4364) | 6.0 | 1.410E-01 | 5.465E-04 | 3.7 |
| 54 | P3 | 4372 | 256 (4144,4399) | 5.8 | 1.260E-01 | 4.928E-04 | 4.0 |
| 55 | P3 | 4409 | 253 (4182,4435) | 5.7 | 1.190E-01 | 4.698E-04 | 5.0 |
| 56 | P3 | 4445 | 251 (4220,4471) | 5.6 | 1.240E-01 | 4.938E-04 | 3.1 |

| | | | | | | | |
|---|---|---|---|---|---|---|---|
| 57 | P3 | 4482 | 249 (4258,4506) | 5.5 | 1.160E-01 | 4.664E-04 | 4.6 |
| 58 | P3 | 4518 | 246 (4296,4542) | 5.5 | 1.280E-01 | 5.193E-04 | 5.0 |

# Probe B (LOW)

Table 9: Energy/Flux Calibration Factors for the Histogram Electron Channels. Histogram LUT ID (LOW-B): 17664.

| CH # | PIX | $E$ [keV] | $\Delta E$ ($E_{lo}$,$E_{hi}$) [keV] | $\Delta E/E$ [%] | $G_0\Delta E$ [cm$^2$ sr keV] | $G_0$ [cm$^2$ sr] | $\Delta G_0/G_0$ [%] |
|---|---|---|---|---|---|---|---|
| 0 | P1 | 21.7 | 1.2 (20.7,21.9) | 5.5 | 3.963E-03 | 3.303E-03 | 0.5 |
| 1 | P1 | 22.7 | 1.2 (21.7,22.9) | 5.3 | 3.936E-03 | 3.280E-03 | 0.6 |
| 2 | P1 | 23.7 | 1.3 (22.7,24.0) | 5.5 | 3.930E-03 | 3.023E-03 | 0.8 |
| 3 | P1 | 24.6 | 1.1 (23.7,24.8) | 4.5 | 3.871E-03 | 3.519E-03 | 0.6 |
| 4 | P1 | 25.7 | 1.4 (24.6,26.0) | 5.4 | 3.841E-03 | 2.744E-03 | 0.7 |
| 5 | P2 | 32.3 | 1.4 (31.2,32.6) | 4.3 | 3.373E-03 | 2.409E-03 | 0.4 |
| 6 | P2 | 33.4 | 1.1 (32.3,33.4) | 3.3 | 3.426E-03 | 3.115E-03 | 0.8 |
| 7 | P2 | 34.2 | 1.6 (33.0,34.6) | 4.7 | 3.389E-03 | 2.118E-03 | 0.4 |
| 8 | P2 | 34.9 | 1.1 (34.2,35.3) | 3.2 | 3.363E-03 | 3.057E-03 | 0.8 |
| 9 | P2 | 36.2 | 1.7 (34.9,36.6) | 4.7 | 3.379E-03 | 1.988E-03 | 0.4 |
| 10 | P2 | 37.0 | 1.2 (36.2,37.4) | 3.2 | 3.368E-03 | 2.807E-03 | 0.4 |
| 11 | P2 | 37.8 | 1.3 (37.0,38.3) | 3.4 | 3.298E-03 | 2.537E-03 | 0.5 |
| 12 | P2 | 39.2 | 1.4 (37.8,39.2) | 3.6 | 3.336E-03 | 2.383E-03 | 0.7 |
| 13 | P2 | 40.1 | 1.4 (38.7,40.1) | 3.5 | 3.268E-03 | 2.334E-03 | 0.6 |
| 14 | P2 | 41.0 | 1.4 (39.6,41.0) | 3.4 | 3.209E-03 | 2.292E-03 | 0.5 |
| 15 | P3 | 48.6 | 1.7 (47.5,49.2) | 3.5 | 2.861E-03 | 1.683E-03 | 0.3 |
| 16 | P3 | 49.7 | 1.1 (48.6,49.7) | 2.2 | 2.948E-03 | 2.680E-03 | 0.6 |
| 17 | P3 | 50.3 | 1.7 (49.2,50.9) | 3.4 | 2.929E-03 | 1.723E-03 | 0.5 |
| 18 | P3 | 51.5 | 1.7 (50.3,52.0) | 3.3 | 2.985E-03 | 1.756E-03 | 0.3 |
| 19 | P3 | 52.6 | 1.1 (51.5,52.6) | 2.1 | 2.987E-03 | 2.715E-03 | 0.5 |
| 20 | P3 | 53.2 | 1.8 (52.0,53.8) | 3.4 | 2.967E-03 | 1.648E-03 | 0.4 |
| 21 | P3 | 54.5 | 1.3 (53.2,54.5) | 2.4 | 2.978E-03 | 2.291E-03 | 0.4 |
| 22 | P3 | 55.1 | 1.9 (53.8,55.7) | 3.4 | 2.920E-03 | 1.537E-03 | 0.5 |
| 23 | P3 | 56.4 | 1.3 (55.1,56.4) | 2.3 | 2.930E-03 | 2.254E-03 | 0.3 |
| 24 | P3 | 57.0 | 1.9 (55.7,57.6) | 3.3 | 2.901E-03 | 1.527E-03 | 0.5 |
| 25 | P3 | 58.3 | 1.3 (57.0,58.3) | 2.2 | 2.911E-03 | 2.239E-03 | 0.3 |
| 26 | P3 | 59.0 | 2.1 (57.6,59.7) | 3.6 | 2.872E-03 | 1.368E-03 | 0.4 |
| 27 | P3 | 60.3 | 1.3 (59.0,60.3) | 2.2 | 2.900E-03 | 2.231E-03 | 0.5 |
| 28 | P3 | 61.0 | 2.0 (59.7,61.7) | 3.3 | 2.792E-03 | 1.396E-03 | 0.3 |
| 29 | P4 | 70.7 | 2.4 (69.2,71.6) | 3.4 | 2.502E-03 | 1.043E-03 | 0.2 |
| 30 | P4 | 71.6 | 1.7 (70.7,72.4) | 2.4 | 2.555E-03 | 1.503E-03 | 0.5 |
| 31 | P4 | 72.4 | 1.6 (71.6,73.2) | 2.2 | 2.602E-03 | 1.626E-03 | 0.7 |
| 32 | P4 | 74.0 | 1.6 (72.4,74.0) | 2.2 | 2.620E-03 | 1.638E-03 | 0.4 |
| 33 | P4 | 74.9 | 1.7 (73.2,74.9) | 2.3 | 2.624E-03 | 1.544E-03 | 0.2 |
| 34 | P4 | 75.7 | 2.6 (74.0,76.6) | 3.4 | 2.620E-03 | 1.008E-03 | 0.2 |
| 35 | P4 | 76.6 | 2.6 (74.9,77.5) | 3.4 | 2.566E-03 | 9.869E-04 | 0.3 |
| 36 | P4 | 77.5 | 2.7 (75.7,78.4) | 3.5 | 2.597E-03 | 9.619E-04 | 0.3 |
| 37 | P4 | 78.4 | 1.8 (77.5,79.3) | 2.3 | 2.589E-03 | 1.438E-03 | 0.5 |
| 38 | P4 | 79.3 | 1.8 (78.4,80.2) | 2.3 | 2.545E-03 | 1.414E-03 | 0.6 |
| 39 | P4 | 80.2 | 1.8 (79.3,81.1) | 2.2 | 2.542E-03 | 1.412E-03 | 0.7 |
| 40 | P4 | 82.0 | 1.8 (80.2,82.0) | 2.2 | 2.605E-03 | 1.447E-03 | 0.7 |
| 41 | P4 | 83.0 | 1.9 (81.1,83.0) | 2.3 | 2.567E-03 | 1.351E-03 | 0.6 |
| 42 | P4 | 83.9 | 1.9 (82.0,83.9) | 2.3 | 2.527E-03 | 1.330E-03 | 0.6 |
| 43 | P4 | 84.9 | 1.9 (83.0,84.9) | 2.2 | 2.558E-03 | 1.346E-03 | 0.6 |
| 44 | P4 | 85.8 | 1.9 (83.9,85.8) | 2.2 | 2.564E-03 | 1.349E-03 | 0.5 |
| 45 | P4 | 86.8 | 1.9 (84.9,86.8) | 2.2 | 2.533E-03 | 1.333E-03 | 0.6 |
| 46 | P5 | 94.0 | 3.2 (91.9,95.1) | 3.4 | 4.671E-03 | 1.460E-03 | 0.2 |
| 47 | P5 | 96.2 | 3.3 (94.0,97.3) | 3.4 | 4.810E-03 | 1.458E-03 | 0.3 |
| 48 | P5 | 98.4 | 2.2 (96.2,98.4) | 2.2 | 4.870E-03 | 2.214E-03 | 0.7 |
| 49 | P5 | 99.5 | 3.7 (97.3,101.0) | 3.7 | 4.729E-03 | 1.278E-03 | 0.4 |
| 50 | P5 | 102.0 | 3.5 (99.5,103.0) | 3.4 | 4.676E-03 | 1.336E-03 | 0.2 |
| 51 | P5 | 104.0 | 2.0 (102.0,104.0) | 1.9 | 4.724E-03 | 2.362E-03 | 0.5 |
| 52 | P5 | 105.0 | 4.0 (103.0,107.0) | 3.8 | 4.539E-03 | 1.135E-03 | 0.4 |
| 53 | P5 | 108.0 | 4.0 (105.0,109.0) | 3.7 | 4.606E-03 | 1.151E-03 | 0.2 |
| 54 | P5 | 109.0 | 2.0 (108.0,110.0) | 1.8 | 4.500E-03 | 2.250E-03 | 0.6 |
| 55 | P5 | 112.0 | 4.0 (109.0,113.0) | 3.6 | 4.520E-03 | 1.130E-03 | 0.2 |
| 56 | P6 | 126.0 | 2.0 (124.0,126.0) | 1.6 | 4.229E-03 | 2.115E-03 | 0.8 |
| 57 | P6 | 128.0 | 4.0 (125.0,129.0) | 3.1 | 4.322E-03 | 1.081E-03 | 0.2 |
| 58 | P6 | 129.0 | 3.0 (128.0,131.0) | 2.3 | 4.250E-03 | 1.417E-03 | 0.4 |
| 59 | P6 | 132.0 | 3.0 (129.0,132.0) | 2.3 | 4.253E-03 | 1.418E-03 | 0.7 |
| 60 | P6 | 134.0 | 4.0 (131.0,135.0) | 3.0 | 4.190E-03 | 1.048E-03 | 0.2 |
| 61 | P6 | 135.0 | 5.0 (132.0,137.0) | 3.7 | 4.090E-03 | 8.180E-04 | 0.4 |

| | | | | | | | |
|---|---|---|---|---|---|---|---|
| 62 | P6 | 137.0 | 3.0 (135.0,138.0) | 2.2 | 4.111E-03 | 1.370E-03 | 0.9 |
| 63 | P6 | 140.0 | 3.0 (137.0,140.0) | 2.1 | 4.143E-03 | 1.381E-03 | 0.5 |
| 64 | P6 | 142.0 | 5.0 (138.0,143.0) | 3.5 | 4.051E-03 | 8.102E-04 | 0.2 |
| 65 | P7 | 162.0 | 3.0 (159.0,162.0) | 1.9 | 3.875E-03 | 1.292E-03 | 0.8 |
| 66 | P7 | 164.0 | 6.0 (160.0,166.0) | 3.7 | 3.967E-03 | 6.612E-04 | 0.6 |
| 67 | P7 | 166.0 | 6.0 (162.0,168.0) | 3.6 | 3.968E-03 | 6.613E-04 | 0.4 |
| 68 | P7 | 168.0 | 6.0 (164.0,170.0) | 3.6 | 3.926E-03 | 6.543E-04 | 0.2 |
| 69 | P7 | 170.0 | 4.0 (168.0,172.0) | 2.4 | 3.867E-03 | 9.668E-04 | 0.2 |
| 70 | P7 | 172.0 | 4.0 (170.0,174.0) | 2.3 | 3.907E-03 | 9.767E-04 | 0.4 |
| 71 | P7 | 174.0 | 4.0 (172.0,176.0) | 2.3 | 3.856E-03 | 9.640E-04 | 0.7 |
| 72 | P7 | 176.0 | 4.0 (174.0,178.0) | 2.3 | 3.740E-03 | 9.350E-04 | 0.8 |
| 73 | P7 | 178.0 | 4.0 (176.0,180.0) | 2.2 | 3.749E-03 | 9.373E-04 | 0.9 |
| 74 | P7 | 180.0 | 4.0 (178.0,182.0) | 2.2 | 3.710E-03 | 9.275E-04 | 1.1 |
| 75 | P8 | 201.6 | 5.4 (198.2,203.6) | 2.7 | 3.023E-03 | 5.598E-04 | 0.5 |
| 76 | P8 | 203.6 | 5.4 (200.1,205.5) | 2.7 | 3.080E-03 | 5.704E-04 | 0.2 |
| 77 | P8 | 205.5 | 5.5 (202.0,207.5) | 2.7 | 3.153E-03 | 5.733E-04 | 0.7 |
| 78 | P8 | 207.4 | 5.5 (203.9,209.4) | 2.7 | 3.110E-03 | 5.655E-04 | 1.2 |
| 79 | P8 | 209.4 | 5.6 (205.8,211.4) | 2.7 | 3.104E-03 | 5.543E-04 | 0.9 |
| 80 | P8 | 211.3 | 5.6 (207.7,213.3) | 2.7 | 3.084E-03 | 5.507E-04 | 0.9 |
| 81 | P8 | 213.2 | 5.7 (209.6,215.3) | 2.7 | 3.061E-03 | 5.370E-04 | 0.2 |
| 82 | P8 | 215.2 | 5.6 (211.6,217.2) | 2.6 | 3.090E-03 | 5.518E-04 | 0.7 |
| 83 | P8 | 217.1 | 5.7 (213.5,219.2) | 2.6 | 3.090E-03 | 5.421E-04 | 1.5 |
| 84 | P8 | 219.0 | 5.7 (215.4,221.1) | 2.6 | 2.975E-03 | 5.219E-04 | 0.9 |
| 85 | P8 | 221.0 | 5.8 (217.3,223.1) | 2.6 | 3.009E-03 | 5.188E-04 | 0.7 |
| 86 | P8 | 222.9 | 5.8 (219.2,225.0) | 2.6 | 3.062E-03 | 5.279E-04 | 0.3 |
| 87 | P8 | 224.8 | 5.9 (221.1,227.0) | 2.6 | 2.970E-03 | 5.034E-04 | 1.3 |
| 88 | P8 | 226.8 | 5.9 (223.0,228.9) | 2.6 | 2.940E-03 | 4.983E-04 | 1.0 |
| 89 | P8 | 228.7 | 5.9 (225.0,230.9) | 2.6 | 2.961E-03 | 5.019E-04 | 0.8 |
| 90 | P8 | 230.7 | 5.9 (226.9,232.8) | 2.6 | 2.966E-03 | 5.027E-04 | 0.4 |

Table 10: Energy/Flux Calibration Factors for the Histogram Electron Channels. Histogram LUT ID (LOW-B): 18690.

| CH # | PIX | $E$ [keV] | $\Delta E$ ($E_{lo}$,$E_{hi}$) [keV] | $\Delta E/E$ [%] | $G_0\Delta E$ [cm$^2$ sr keV] | $G_0$ [cm$^2$ sr] | $\Delta G_0/G_0$ [%] |
|---|---|---|---|---|---|---|---|
| 0 | P1 | 21.2 | 2.1 (19.8,21.9) | 9.9 | 7.952E-03 | 3.787E-03 | 0.7 |
| 1 | P1 | 23.2 | 2.3 (21.7,24.0) | 9.9 | 7.874E-03 | 3.423E-03 | 0.8 |
| 2 | P1 | 25.1 | 2.3 (23.7,26.0) | 9.2 | 7.728E-03 | 3.360E-03 | 0.5 |
| 3 | P2 | 31.9 | 2.1 (30.5,32.6) | 6.6 | 6.641E-03 | 3.162E-03 | 0.6 |
| 4 | P2 | 33.8 | 2.3 (32.3,34.6) | 6.8 | 6.820E-03 | 2.965E-03 | 0.5 |
| 5 | P2 | 35.8 | 2.4 (34.2,36.6) | 6.7 | 6.800E-03 | 2.833E-03 | 0.7 |
| 6 | P2 | 37.4 | 2.1 (36.2,38.3) | 5.6 | 6.670E-03 | 3.176E-03 | 0.4 |
| 7 | P2 | 39.6 | 2.3 (37.8,40.1) | 5.8 | 6.605E-03 | 2.872E-03 | 0.7 |
| 8 | P3 | 49.7 | 2.3 (48.6,50.9) | 4.6 | 5.820E-03 | 2.530E-03 | 0.7 |
| 9 | P3 | 52.0 | 2.3 (50.3,52.6) | 4.4 | 5.971E-03 | 2.596E-03 | 0.4 |
| 10 | P3 | 53.8 | 2.5 (52.0,54.5) | 4.6 | 5.951E-03 | 2.380E-03 | 0.3 |
| 11 | P3 | 55.7 | 2.6 (53.8,56.4) | 4.7 | 5.854E-03 | 2.252E-03 | 0.3 |
| 12 | P3 | 57.6 | 2.6 (55.7,58.3) | 4.5 | 5.819E-03 | 2.238E-03 | 0.3 |
| 13 | P3 | 59.7 | 2.7 (57.6,60.3) | 4.5 | 5.775E-03 | 2.139E-03 | 0.3 |
| 14 | P4 | 71.6 | 3.2 (69.2,72.4) | 4.5 | 5.115E-03 | 1.598E-03 | 0.4 |
| 15 | P4 | 73.2 | 2.4 (71.6,74.0) | 3.3 | 5.223E-03 | 2.176E-03 | 0.2 |
| 16 | P4 | 74.9 | 2.5 (73.2,75.7) | 3.3 | 5.181E-03 | 2.072E-03 | 0.6 |
| 17 | P4 | 77.5 | 3.5 (74.9,78.4) | 4.5 | 5.223E-03 | 1.492E-03 | 0.5 |
| 18 | P4 | 79.3 | 2.7 (77.5,80.2) | 3.4 | 5.198E-03 | 1.925E-03 | 0.3 |
| 19 | P4 | 81.1 | 2.7 (79.3,82.0) | 3.3 | 5.154E-03 | 1.909E-03 | 0.2 |
| 20 | P4 | 83.0 | 2.8 (81.1,83.9) | 3.4 | 5.045E-03 | 1.802E-03 | 0.2 |
| 21 | P4 | 84.9 | 2.8 (83.0,85.8) | 3.3 | 5.066E-03 | 1.809E-03 | 0.3 |
| 22 | P5 | 94.0 | 3.2 (91.9,95.1) | 3.4 | 4.671E-03 | 1.460E-03 | 0.2 |
| 23 | P5 | 96.2 | 3.3 (94.0,97.3) | 3.4 | 4.810E-03 | 1.458E-03 | 0.3 |
| 24 | P5 | 98.4 | 2.2 (96.2,98.4) | 2.2 | 4.870E-03 | 2.214E-03 | 0.7 |
| 25 | P5 | 99.5 | 3.7 (97.3,101.0) | 3.7 | 4.729E-03 | 1.278E-03 | 0.4 |
| 26 | P5 | 102.0 | 3.5 (99.5,103.0) | 3.4 | 4.676E-03 | 1.336E-03 | 0.2 |
| 27 | P5 | 104.0 | 2.0 (102.0,104.0) | 1.9 | 4.724E-03 | 2.362E-03 | 0.5 |
| 28 | P5 | 105.0 | 4.0 (103.0,107.0) | 3.8 | 4.539E-03 | 1.135E-03 | 0.4 |
| 29 | P5 | 108.0 | 4.0 (105.0,109.0) | 3.7 | 4.606E-03 | 1.151E-03 | 0.2 |
| 30 | P5 | 109.0 | 2.0 (108.0,110.0) | 1.8 | 4.500E-03 | 2.250E-03 | 0.6 |

| | | | | | | | |
|---|---|---|---|---|---|---|---|
| 31 | P5 | 112.0 | 4.0 (109.0,113.0) | 3.6 | 4.520E-03 | 1.130E-03 | 0.2 |
| 32 | P6 | 126.0 | 2.0 (124.0,126.0) | 1.6 | 4.229E-03 | 2.115E-03 | 0.8 |
| 33 | P6 | 128.0 | 4.0 (125.0,129.0) | 3.1 | 4.322E-03 | 1.081E-03 | 0.2 |
| 34 | P6 | 129.0 | 3.0 (128.0,131.0) | 2.3 | 4.250E-03 | 1.417E-03 | 0.4 |
| 35 | P6 | 132.0 | 3.0 (129.0,132.0) | 2.3 | 4.253E-03 | 1.418E-03 | 0.7 |
| 36 | P6 | 134.0 | 4.0 (131.0,135.0) | 3.0 | 4.190E-03 | 1.048E-03 | 0.2 |
| 37 | P6 | 135.0 | 5.0 (132.0,137.0) | 3.7 | 4.090E-03 | 8.180E-04 | 0.4 |
| 38 | P6 | 137.0 | 3.0 (135.0,138.0) | 2.2 | 4.111E-03 | 1.370E-03 | 0.9 |
| 39 | P6 | 140.0 | 3.0 (137.0,140.0) | 2.1 | 4.143E-03 | 1.381E-03 | 0.5 |
| 40 | P6 | 142.0 | 5.0 (138.0,143.0) | 3.5 | 4.051E-03 | 8.102E-04 | 0.2 |
| 41 | P7 | 162.0 | 3.0 (159.0,162.0) | 1.9 | 3.875E-03 | 1.292E-03 | 0.8 |
| 42 | P7 | 164.0 | 6.0 (160.0,166.0) | 3.7 | 3.967E-03 | 6.612E-04 | 0.6 |
| 43 | P7 | 166.0 | 6.0 (162.0,168.0) | 3.6 | 3.968E-03 | 6.613E-04 | 0.4 |
| 44 | P7 | 168.0 | 6.0 (164.0,170.0) | 3.6 | 3.926E-03 | 6.543E-04 | 0.2 |
| 45 | P7 | 170.0 | 4.0 (168.0,172.0) | 2.4 | 3.867E-03 | 9.668E-04 | 0.2 |
| 46 | P7 | 172.0 | 4.0 (170.0,174.0) | 2.3 | 3.907E-03 | 9.767E-04 | 0.4 |
| 47 | P7 | 174.0 | 4.0 (172.0,176.0) | 2.3 | 3.856E-03 | 9.640E-04 | 0.7 |
| 48 | P7 | 176.0 | 4.0 (174.0,178.0) | 2.3 | 3.740E-03 | 9.350E-04 | 0.8 |
| 49 | P7 | 178.0 | 4.0 (176.0,180.0) | 2.2 | 3.749E-03 | 9.373E-04 | 0.9 |
| 50 | P7 | 180.0 | 4.0 (178.0,182.0) | 2.2 | 3.710E-03 | 9.275E-04 | 1.1 |
| 51 | P8 | 202.0 | 4.7 (199.5,204.2) | 2.3 | 3.040E-03 | 6.468E-04 | 1.3 |
| 52 | P8 | 204.0 | 4.8 (201.4,206.2) | 2.4 | 3.082E-03 | 6.421E-04 | 0.9 |
| 53 | P8 | 206.0 | 4.9 (203.3,208.2) | 2.4 | 3.059E-03 | 6.243E-04 | 0.8 |
| 54 | P8 | 208.0 | 5.0 (205.2,210.2) | 2.4 | 3.097E-03 | 6.194E-04 | 0.3 |
| 55 | P8 | 210.0 | 5.1 (207.1,212.2) | 2.4 | 3.182E-03 | 6.239E-04 | 0.7 |
| 56 | P8 | 212.0 | 5.2 (209.0,214.2) | 2.5 | 3.080E-03 | 5.923E-04 | 1.3 |
| 57 | P8 | 214.0 | 5.3 (210.9,216.2) | 2.5 | 3.039E-03 | 5.734E-04 | 0.9 |
| 58 | P8 | 215.9 | 5.4 (212.8,218.2) | 2.5 | 3.045E-03 | 5.639E-04 | 0.9 |
| 59 | P8 | 217.9 | 5.5 (214.7,220.2) | 2.5 | 3.045E-03 | 5.536E-04 | 0.2 |
| 60 | P8 | 219.9 | 5.7 (216.6,222.3) | 2.6 | 3.020E-03 | 5.298E-04 | 0.9 |
| 61 | P8 | 221.9 | 5.8 (218.5,224.3) | 2.6 | 2.980E-03 | 5.138E-04 | 1.3 |
| 62 | P8 | 223.9 | 5.9 (220.4,226.3) | 2.6 | 2.960E-03 | 5.017E-04 | 1.0 |
| 63 | P8 | 225.9 | 6.0 (222.3,228.3) | 2.7 | 2.997E-03 | 4.995E-04 | 0.4 |
| 64 | P8 | 227.9 | 6.1 (224.2,230.3) | 2.7 | 2.991E-03 | 4.903E-04 | 0.9 |

## Probe B (M35)

Table 11: Energy/Flux Calibration Factors for the Histogram Electron Channels. Histogram LUT ID (M35-B): 39168.

| CH # | PIX | $E$ [keV] | $\Delta E$ ($E_{lo}$,$E_{hi}$) [keV] | $\Delta E/E$ [%] | $G_0\Delta E$ [cm$^2$ sr keV] | $G_0$ [cm$^2$ sr] | $\Delta G_0/G_0$ [%] |
|---|---|---|---|---|---|---|---|
| 0 | P1 | 141 | 6 (135,141) | 4.3 | 1.980E-02 | 3.300E-03 | 1.7 |
| 1 | P1 | 144 | 6 (138,144) | 4.2 | 2.040E-02 | 3.400E-03 | 1.5 |
| 2 | P1 | 149 | 7 (142,149) | 4.7 | 2.100E-02 | 3.000E-03 | 1.3 |
| 3 | P1 | 154 | 7 (147,154) | 4.5 | 2.100E-02 | 3.043E-03 | 1.3 |
| 4 | P1 | 160 | 7 (153,160) | 4.4 | 2.110E-02 | 3.014E-03 | 1.4 |
| 5 | P1 | 163 | 7 (156,163) | 4.3 | 2.070E-02 | 2.957E-03 | 1.2 |
| 6 | P1 | 169 | 6 (163,169) | 3.6 | 2.080E-02 | 3.467E-03 | 1.3 |
| 7 | P1 | 173 | 8 (165,173) | 4.6 | 2.050E-02 | 2.563E-03 | 1.2 |
| 8 | P1 | 179 | 6 (173,179) | 3.4 | 2.060E-02 | 3.433E-03 | 1.3 |
| 9 | P1 | 183 | 8 (175,183) | 4.4 | 2.020E-02 | 2.525E-03 | 1.1 |
| 10 | P1 | 187 | 6 (181,187) | 3.4 | 1.900E-02 | 3.016E-03 | 1.2 |
| 11 | P2 | 227 | 8 (219,227) | 3.5 | 1.740E-02 | 2.203E-03 | 1.2 |
| 12 | P2 | 230 | 6 (224,230) | 2.7 | 1.820E-02 | 2.889E-03 | 1.0 |
| 13 | P2 | 235 | 7 (228,235) | 2.9 | 1.867E-02 | 2.746E-03 | 0.9 |
| 14 | P2 | 240 | 7 (233,240) | 3.0 | 1.888E-02 | 2.622E-03 | 0.9 |
| 15 | P2 | 246 | 9 (237,246) | 3.5 | 1.910E-02 | 2.221E-03 | 1.1 |
| 16 | P2 | 249 | 7 (242,249) | 2.8 | 1.864E-02 | 2.663E-03 | 0.9 |
| 17 | P2 | 254 | 8 (246,254) | 3.0 | 1.872E-02 | 2.496E-03 | 0.8 |
| 18 | P2 | 257 | 6 (251,257) | 2.3 | 1.840E-02 | 3.119E-03 | 1.0 |
| 19 | P2 | 263 | 7 (256,263) | 2.8 | 1.842E-02 | 2.523E-03 | 0.8 |
| 20 | P2 | 269 | 9 (260,269) | 3.2 | 1.835E-02 | 2.109E-03 | 0.9 |
| 21 | P2 | 272 | 7 (265,272) | 2.6 | 1.828E-02 | 2.539E-03 | 0.9 |
| 22 | P2 | 279 | 10 (269,279) | 3.4 | 1.821E-02 | 1.897E-03 | 0.8 |
| 23 | P2 | 282 | 8 (274,282) | 2.8 | 1.785E-02 | 2.231E-03 | 0.8 |
| 24 | P3 | 330 | 9 (321,330) | 2.7 | 1.532E-02 | 1.721E-03 | 0.7 |
| 25 | P3 | 334 | 8 (326,334) | 2.5 | 1.588E-02 | 1.937E-03 | 0.7 |
| 26 | P3 | 338 | 8 (330,338) | 2.2 | 1.608E-02 | 2.116E-03 | 0.9 |
| 27 | P3 | 346 | 11 (335,346) | 3.2 | 1.659E-02 | 1.508E-03 | 0.9 |
| 28 | P3 | 350 | 10 (340,350) | 3.0 | 1.659E-02 | 1.595E-03 | 0.6 |
| 29 | P3 | 354 | 9 (344,354) | 2.7 | 1.659E-02 | 1.765E-03 | 0.6 |
| 30 | P3 | 358 | 9 (349,358) | 2.5 | 1.654E-02 | 1.818E-03 | 0.6 |
| 31 | P3 | 362 | 8 (354,362) | 2.3 | 1.631E-02 | 1.919E-03 | 0.8 |
| 32 | P3 | 370 | 12 (358,370) | 3.2 | 1.649E-02 | 1.386E-03 | 0.9 |
| 33 | P3 | 374 | 11 (363,374) | 3.0 | 1.645E-02 | 1.469E-03 | 0.8 |
| 34 | P3 | 379 | 12 (367,379) | 3.1 | 1.638E-02 | 1.412E-03 | 0.6 |
| 35 | P3 | 383 | 11 (372,383) | 2.9 | 1.627E-02 | 1.479E-03 | 0.6 |
| 36 | P3 | 387 | 10 (377,387) | 2.7 | 1.613E-02 | 1.551E-03 | 0.6 |
| 37 | P3 | 392 | 11 (381,392) | 2.7 | 1.588E-02 | 1.484E-03 | 0.5 |
| 38 | P3 | 396 | 10 (386,396) | 2.6 | 1.577E-02 | 1.561E-03 | 0.6 |
| 39 | P4 | 445 | 14 (431,445) | 3.1 | 1.318E-02 | 9.482E-04 | 0.4 |
| 40 | P4 | 449 | 14 (436,449) | 3.1 | 1.347E-02 | 9.761E-04 | 0.4 |
| 41 | P4 | 454 | 14 (440,454) | 3.0 | 1.388E-02 | 1.021E-03 | 0.5 |
| 42 | P4 | 459 | 14 (445,459) | 2.9 | 1.407E-02 | 1.042E-03 | 0.6 |
| 43 | P4 | 463 | 13 (450,463) | 2.9 | 1.385E-02 | 1.041E-03 | 0.7 |
| 44 | P4 | 468 | 13 (455,468) | 2.8 | 1.394E-02 | 1.056E-03 | 0.8 |
| 45 | P4 | 473 | 13 (460,473) | 2.8 | 1.381E-02 | 1.062E-03 | 0.7 |
| 46 | P4 | 477 | 13 (464,477) | 2.7 | 1.357E-02 | 1.052E-03 | 0.7 |
| 47 | P4 | 482 | 13 (469,482) | 2.7 | 1.356E-02 | 1.059E-03 | 0.5 |
| 48 | P4 | 487 | 13 (474,487) | 2.6 | 1.357E-02 | 1.077E-03 | 0.4 |
| 49 | P4 | 491 | 12 (479,491) | 2.5 | 1.331E-02 | 1.065E-03 | 0.4 |
| 50 | P4 | 496 | 12 (484,496) | 2.5 | 1.342E-02 | 1.091E-03 | 0.6 |
| 51 | P4 | 501 | 12 (488,501) | 2.4 | 1.323E-02 | 1.084E-03 | 0.8 |
| 52 | P4 | 505 | 12 (493,505) | 2.4 | 1.297E-02 | 1.081E-03 | 0.6 |
| 53 | P4 | 510 | 12 (498,510) | 2.3 | 1.320E-02 | 1.109E-03 | 0.5 |
| 54 | P4 | 514 | 12 (503,514) | 2.3 | 1.286E-02 | 1.099E-03 | 0.4 |
| 55 | P5 | 571 | 14 (558,571) | 2.4 | 1.163E-02 | 8.428E-04 | 0.4 |
| 56 | P5 | 576 | 14 (562,576) | 2.4 | 1.221E-02 | 8.721E-04 | 0.4 |
| 57 | P5 | 581 | 14 (567,581) | 2.5 | 1.226E-02 | 8.573E-04 | 0.7 |
| 58 | P5 | 586 | 15 (572,586) | 2.5 | 1.242E-02 | 8.507E-04 | 0.7 |
| 59 | P5 | 591 | 15 (577,591) | 2.5 | 1.235E-02 | 8.401E-04 | 0.4 |
| 60 | P5 | 596 | 15 (582,596) | 2.5 | 1.261E-02 | 8.407E-04 | 0.3 |
| 61 | P5 | 601 | 15 (586,602) | 2.5 | 1.244E-02 | 8.131E-04 | 0.8 |

| | | | | | | | |
|---|---|---|---|---|---|---|---|
| 62 | P5 | 606 | 15 (591,607) | 2.5 | 1.236E-02 | 8.026E-04 | 0.5 |
| 63 | P5 | 611 | 16 (596,612) | 2.6 | 1.208E-02 | 7.694E-04 | 0.4 |
| 64 | P5 | 616 | 16 (601,617) | 2.6 | 1.244E-02 | 7.775E-04 | 0.5 |
| 65 | P5 | 621 | 16 (606,622) | 2.6 | 1.217E-02 | 7.559E-04 | 0.7 |
| 66 | P5 | 626 | 16 (610,627) | 2.6 | 1.206E-02 | 7.354E-04 | 0.3 |
| 67 | P5 | 631 | 17 (615,632) | 2.6 | 1.208E-02 | 7.234E-04 | 0.4 |
| 68 | P5 | 636 | 17 (620,637) | 2.6 | 1.184E-02 | 7.048E-04 | 0.9 |
| 69 | P5 | 641 | 17 (625,642) | 2.7 | 1.167E-02 | 6.825E-04 | 0.4 |
| 70 | P5 | 646 | 17 (630,647) | 2.7 | 1.168E-02 | 6.751E-04 | 0.3 |
| 71 | P5 | 650 | 18 (635,652) | 2.7 | 1.187E-02 | 6.744E-04 | 0.9 |
| 72 | P6 | 698 | 19 (683,701) | 2.7 | 9.911E-03 | 5.328E-04 | 0.3 |
| 73 | P6 | 703 | 19 (688,706) | 2.6 | 9.953E-03 | 5.351E-04 | 0.9 |
| 74 | P6 | 708 | 18 (692,711) | 2.6 | 1.031E-02 | 5.573E-04 | 0.4 |
| 75 | P6 | 713 | 18 (697,716) | 2.6 | 1.032E-02 | 5.609E-04 | 0.4 |
| 76 | P6 | 718 | 18 (702,721) | 2.5 | 1.027E-02 | 5.612E-04 | 0.4 |
| 77 | P6 | 723 | 18 (707,725) | 2.5 | 1.051E-02 | 5.775E-04 | 0.4 |
| 78 | P6 | 728 | 18 (712,730) | 2.5 | 1.039E-02 | 5.709E-04 | 1.0 |
| 79 | P6 | 733 | 18 (717,735) | 2.5 | 1.035E-02 | 5.687E-04 | 0.4 |
| 80 | P6 | 738 | 18 (722,740) | 2.5 | 1.043E-02 | 5.762E-04 | 0.5 |
| 81 | P6 | 743 | 18 (727,745) | 2.4 | 1.033E-02 | 5.739E-04 | 0.4 |
| 82 | P6 | 748 | 18 (732,750) | 2.4 | 1.035E-02 | 5.750E-04 | 0.4 |
| 83 | P6 | 753 | 18 (736,754) | 2.4 | 9.889E-03 | 5.525E-04 | 0.7 |
| 84 | P6 | 758 | 18 (741,759) | 2.3 | 1.015E-02 | 5.702E-04 | 0.5 |
| 85 | P6 | 763 | 18 (746,764) | 2.3 | 9.951E-03 | 5.622E-04 | 0.9 |
| 86 | P6 | 768 | 18 (751,769) | 2.3 | 9.910E-03 | 5.599E-04 | 0.4 |
| 87 | P6 | 773 | 18 (756,774) | 2.3 | 9.792E-03 | 5.532E-04 | 0.6 |
| 88 | P6 | 778 | 18 (761,778) | 2.3 | 9.881E-03 | 5.614E-04 | 0.2 |
| 89 | P6 | 783 | 18 (766,783) | 2.2 | 9.545E-03 | 5.454E-04 | 0.6 |
| 90 | P7 | 857 | 19 (839,857) | 2.2 | 8.439E-03 | 4.489E-04 | 0.7 |
| 91 | P7 | 862 | 19 (843,862) | 2.2 | 8.617E-03 | 4.488E-04 | 0.5 |
| 92 | P7 | 867 | 19 (848,867) | 2.2 | 8.679E-03 | 4.474E-04 | 0.7 |
| 93 | P7 | 871 | 20 (852,872) | 2.3 | 8.529E-03 | 4.329E-04 | 0.5 |
| 94 | P7 | 876 | 20 (857,877) | 2.3 | 8.905E-03 | 4.453E-04 | 0.8 |
| 95 | P7 | 881 | 20 (861,882) | 2.3 | 8.830E-03 | 4.350E-04 | 0.5 |
| 96 | P7 | 885 | 21 (866,887) | 2.3 | 8.784E-03 | 4.264E-04 | 0.9 |
| 97 | P7 | 890 | 21 (871,892) | 2.3 | 8.722E-03 | 4.173E-04 | 0.6 |
| 98 | P7 | 895 | 21 (875,896) | 2.4 | 8.720E-03 | 4.113E-04 | 1.1 |
| 99 | P7 | 899 | 22 (880,901) | 2.4 | 8.559E-03 | 3.981E-04 | 0.7 |
| 100 | P7 | 904 | 22 (884,906) | 2.4 | 8.410E-03 | 3.858E-04 | 1.1 |
| 101 | P7 | 909 | 22 (889,911) | 2.4 | 8.606E-03 | 3.894E-04 | 0.7 |
| 102 | P7 | 913 | 22 (894,916) | 2.5 | 8.354E-03 | 3.729E-04 | 0.9 |
| 103 | P7 | 918 | 23 (898,921) | 2.5 | 8.395E-03 | 3.715E-04 | 0.7 |
| 104 | P7 | 923 | 23 (903,926) | 2.5 | 8.248E-03 | 3.602E-04 | 0.4 |
| 105 | P7 | 927 | 23 (907,931) | 2.5 | 8.166E-03 | 3.520E-04 | 0.7 |
| 106 | P7 | 932 | 24 (912,936) | 2.5 | 8.316E-03 | 3.539E-04 | 0.4 |
| 107 | P7 | 937 | 24 (917,940) | 2.5 | 8.204E-03 | 3.447E-04 | 0.8 |
| 108 | P8 | 1018 | 25 (995,1021) | 2.5 | 6.538E-03 | 2.574E-04 | 0.7 |
| 109 | P8 | 1023 | 25 (1000,1026) | 2.5 | 6.847E-03 | 2.696E-04 | 0.7 |
| 110 | P8 | 1028 | 25 (1005,1030) | 2.5 | 7.065E-03 | 2.781E-04 | 0.8 |
| 111 | P8 | 1033 | 26 (1009,1035) | 2.5 | 7.179E-03 | 2.815E-04 | 0.8 |
| 112 | P8 | 1037 | 25 (1014,1040) | 2.5 | 7.203E-03 | 2.825E-04 | 1.0 |
| 113 | P8 | 1042 | 26 (1019,1044) | 2.4 | 6.935E-03 | 2.720E-04 | 0.8 |
| 114 | P8 | 1047 | 26 (1024,1049) | 2.4 | 7.097E-03 | 2.783E-04 | 0.6 |
| 115 | P8 | 1051 | 26 (1028,1054) | 2.4 | 6.909E-03 | 2.699E-04 | 0.9 |
| 116 | P8 | 1056 | 26 (1033,1058) | 2.4 | 7.094E-03 | 2.771E-04 | 1.0 |
| 117 | P8 | 1061 | 26 (1038,1063) | 2.4 | 7.070E-03 | 2.762E-04 | 1.3 |
| 118 | P8 | 1066 | 26 (1042,1068) | 2.4 | 7.037E-03 | 2.728E-04 | 0.9 |
| 119 | P8 | 1070 | 26 (1047,1073) | 2.4 | 7.028E-03 | 2.724E-04 | 0.3 |
| 120 | P8 | 1075 | 26 (1052,1077) | 2.4 | 7.120E-03 | 2.760E-04 | 1.1 |
| 121 | P8 | 1080 | 26 (1056,1082) | 2.4 | 7.100E-03 | 2.752E-04 | 1.0 |
| 122 | P8 | 1084 | 26 (1061,1087) | 2.4 | 6.850E-03 | 2.645E-04 | 1.1 |
| 123 | P8 | 1089 | 26 (1066,1091) | 2.4 | 6.960E-03 | 2.687E-04 | 1.3 |
| 124 | P8 | 1094 | 26 (1070,1096) | 2.4 | 6.707E-03 | 2.590E-04 | 0.9 |
| 125 | P8 | 1098 | 26 (1075,1101) | 2.4 | 7.110E-03 | 2.735E-04 | 1.3 |
| 126 | P8 | 1103 | 26 (1080,1106) | 2.4 | 6.840E-03 | 2.631E-04 | 1.3 |
| 127 | P8 | 1108 | 26 (1084,1110) | 2.4 | 6.412E-03 | 2.457E-04 | 0.3 |
| 128 | P8 | 1112 | 26 (1089,1115) | 2.3 | 6.500E-03 | 2.490E-04 | 1.3 |

Table 12: Energy/Flux Calibration Factors for the Histogram Electron Channels. Histogram LUT ID (M35-B): 39169.

| CH # | PIX | $E$ [keV] | $\Delta E$ ($E_{lo}$,$E_{hi}$) [keV] | $\Delta E/E$ [%] | $G_0\Delta E$ [cm$^2$ sr keV] | $G_0$ [cm$^2$ sr] | $\Delta G_0/G_0$ [%] |
|---|---|---|---|---|---|---|---|
| 0 | P1 | 142 | 11 (133,144) | 7.7 | 4.030E-02 | 3.664E-03 | 1.5 |
| 1 | P1 | 151 | 12 (142,154) | 7.9 | 4.160E-02 | 3.467E-03 | 1.5 |
| 2 | P1 | 161 | 12 (151,163) | 7.5 | 4.180E-02 | 3.483E-03 | 1.2 |
| 3 | P1 | 171 | 12 (161,173) | 7.0 | 4.140E-02 | 3.450E-03 | 1.2 |
| 4 | P1 | 181 | 12 (171,183) | 6.6 | 4.080E-02 | 3.400E-03 | 1.2 |
| 5 | P2 | 227 | 13 (217,230) | 5.7 | 3.540E-02 | 2.723E-03 | 1.2 |
| 6 | P2 | 238 | 11 (227,238) | 4.6 | 3.753E-02 | 3.412E-03 | 0.9 |
| 7 | P2 | 246 | 14 (235,249) | 5.7 | 3.746E-02 | 2.676E-03 | 1.0 |
| 8 | P2 | 257 | 11 (246,257) | 4.3 | 3.745E-02 | 3.405E-03 | 0.9 |
| 9 | P2 | 266 | 12 (254,266) | 4.5 | 3.680E-02 | 3.067E-03 | 0.8 |
| 10 | P2 | 275 | 12 (263,275) | 4.4 | 3.651E-02 | 3.043E-03 | 0.8 |
| 11 | P2 | 285 | 13 (272,285) | 4.6 | 3.451E-02 | 2.655E-03 | 0.8 |
| 12 | P3 | 338 | 15 (323,338) | 4.4 | 3.228E-02 | 2.152E-03 | 0.7 |
| 13 | P3 | 346 | 12 (334,346) | 3.5 | 3.294E-02 | 2.745E-03 | 0.7 |
| 14 | P3 | 358 | 16 (342,358) | 4.5 | 3.337E-02 | 2.086E-03 | 0.8 |
| 15 | P3 | 366 | 12 (354,366) | 3.3 | 3.287E-02 | 2.739E-03 | 0.6 |
| 16 | P3 | 374 | 12 (362,374) | 3.2 | 3.257E-02 | 2.714E-03 | 0.7 |
| 17 | P3 | 383 | 13 (370,383) | 3.4 | 3.206E-02 | 2.466E-03 | 0.8 |
| 18 | P3 | 396 | 17 (379,396) | 4.3 | 3.184E-02 | 1.873E-03 | 0.9 |
| 19 | P4 | 444 | 10 (434,444) | 2.3 | 2.639E-02 | 2.639E-03 | 0.8 |
| 20 | P4 | 454 | 15 (439,454) | 3.3 | 2.770E-02 | 1.847E-03 | 0.6 |
| 21 | P4 | 465 | 16 (449,465) | 3.4 | 2.759E-02 | 1.724E-03 | 0.4 |
| 22 | P4 | 475 | 15 (460,475) | 3.2 | 2.765E-02 | 1.843E-03 | 0.4 |
| 23 | P4 | 486 | 16 (470,486) | 3.3 | 2.735E-02 | 1.709E-03 | 0.7 |
| 24 | P4 | 492 | 11 (481,492) | 2.2 | 2.650E-02 | 2.409E-03 | 0.6 |
| 25 | P4 | 503 | 17 (486,503) | 3.4 | 2.624E-02 | 1.544E-03 | 0.4 |
| 26 | P4 | 515 | 17 (498,515) | 3.3 | 2.629E-02 | 1.546E-03 | 0.7 |
| 27 | P5 | 577 | 19 (558,577) | 3.3 | 2.404E-02 | 1.265E-03 | 0.8 |
| 28 | P5 | 584 | 14 (570,584) | 2.4 | 2.469E-02 | 1.764E-03 | 0.3 |
| 29 | P5 | 597 | 20 (577,597) | 3.4 | 2.516E-02 | 1.258E-03 | 0.9 |
| 30 | P5 | 604 | 14 (590,604) | 2.3 | 2.482E-02 | 1.773E-03 | 0.4 |
| 31 | P5 | 611 | 21 (597,618) | 3.4 | 2.430E-02 | 1.157E-03 | 0.8 |
| 32 | P5 | 625 | 14 (611,625) | 2.2 | 2.445E-02 | 1.746E-03 | 0.6 |
| 33 | P5 | 632 | 14 (618,632) | 2.2 | 2.390E-02 | 1.707E-03 | 0.4 |
| 34 | P5 | 639 | 21 (625,646) | 3.3 | 2.315E-02 | 1.102E-03 | 0.9 |
| 35 | P5 | 654 | 15 (639,654) | 2.3 | 2.319E-02 | 1.546E-03 | 0.5 |
| 36 | P6 | 700 | 23 (677,700) | 3.3 | 1.930E-02 | 8.391E-04 | 0.9 |
| 37 | P6 | 708 | 16 (692,708) | 2.3 | 2.044E-02 | 1.277E-03 | 0.7 |
| 38 | P6 | 716 | 16 (700,716) | 2.2 | 2.060E-02 | 1.288E-03 | 0.3 |
| 39 | P6 | 724 | 25 (708,733) | 3.5 | 2.088E-02 | 8.352E-04 | 0.4 |
| 40 | P6 | 733 | 25 (716,741) | 3.4 | 2.058E-02 | 8.232E-04 | 0.6 |
| 41 | P6 | 741 | 25 (724,749) | 3.4 | 2.050E-02 | 8.200E-04 | 1.0 |
| 42 | P6 | 758 | 17 (741,758) | 2.2 | 2.022E-02 | 1.189E-03 | 0.9 |
| 43 | P6 | 767 | 18 (749,767) | 2.3 | 1.986E-02 | 1.103E-03 | 0.6 |
| 44 | P6 | 775 | 17 (758,775) | 2.2 | 1.968E-02 | 1.158E-03 | 0.3 |
| 45 | P7 | 861 | 18 (843,861) | 2.1 | 1.706E-02 | 9.425E-04 | 0.6 |
| 46 | P7 | 870 | 19 (851,870) | 2.2 | 1.721E-02 | 9.154E-04 | 0.6 |
| 47 | P7 | 879 | 20 (860,879) | 2.2 | 1.774E-02 | 9.097E-04 | 0.7 |
| 48 | P7 | 888 | 20 (869,889) | 2.3 | 1.750E-02 | 8.621E-04 | 0.7 |
| 49 | P7 | 897 | 21 (878,899) | 2.3 | 1.729E-02 | 8.233E-04 | 0.9 |
| 50 | P7 | 906 | 22 (887,909) | 2.4 | 1.720E-02 | 7.926E-04 | 1.0 |
| 51 | P7 | 915 | 24 (896,920) | 2.6 | 1.690E-02 | 7.191E-04 | 1.2 |
| 52 | P7 | 924 | 24 (906,930) | 2.6 | 1.620E-02 | 6.694E-04 | 1.0 |
| 53 | P7 | 933 | 26 (915,941) | 2.8 | 1.633E-02 | 6.305E-04 | 0.9 |
| 54 | P8 | 1019 | 24 (998,1023) | 2.4 | 1.338E-02 | 5.552E-04 | 0.4 |
| 55 | P8 | 1029 | 24 (1008,1032) | 2.4 | 1.424E-02 | 5.812E-04 | 0.8 |
| 56 | P8 | 1038 | 25 (1017,1042) | 2.4 | 1.414E-02 | 5.656E-04 | 0.9 |
| 57 | P8 | 1047 | 25 (1026,1051) | 2.4 | 1.390E-02 | 5.494E-04 | 1.1 |
| 58 | P8 | 1056 | 26 (1035,1060) | 2.4 | 1.402E-02 | 5.455E-04 | 0.4 |
| 59 | P8 | 1066 | 26 (1044,1070) | 2.5 | 1.407E-02 | 5.370E-04 | 0.6 |
| 60 | P8 | 1075 | 27 (1053,1080) | 2.5 | 1.420E-02 | 5.338E-04 | 1.1 |
| 61 | P8 | 1084 | 27 (1062,1089) | 2.5 | 1.370E-02 | 5.055E-04 | 1.3 |

| | | | | | | | |
|---|---|---|---|---|---|---|---|
| 62 | P8 | 1093 | 27 (1071,1098) | 2.5 | 1.366E-02 | 4.985E-04 | 0.5 |
| 63 | P8 | 1103 | 28 (1080,1108) | 2.5 | 1.326E-02 | 4.770E-04 | 0.7 |
| 64 | P8 | 1112 | 28 (1089,1117) | 2.5 | 1.290E-02 | 4.558E-04 | 1.2 |

## Probe B (M75)

Table 13: Energy/Flux Calibration Factors for the Histogram Electron Channels. Histogram LUT ID (M75-B): 25856.

| CH # | PIX | $E$ [keV] | $\Delta E$ ($E_{lo}$,$E_{hi}$) [keV] | $\Delta E/E$ [%] | $G_0\Delta E$ [cm$^2$ sr keV] | $G_0$ [cm$^2$ sr] | $\Delta G_0/G_0$ [%] |
|---|---|---|---|---|---|---|---|
| 0 | P1 | 139 | 7 (132,139) | 5.0 | 1.960E-02 | 2.800E-03 | 1.5 |
| 1 | P1 | 144 | 6 (138,144) | 4.2 | 2.020E-02 | 3.367E-03 | 1.4 |
| 2 | P1 | 149 | 6 (143,149) | 4.0 | 2.040E-02 | 3.400E-03 | 1.4 |
| 3 | P1 | 154 | 6 (148,154) | 3.9 | 2.040E-02 | 3.400E-03 | 1.5 |
| 4 | P1 | 158 | 7 (151,158) | 4.4 | 2.010E-02 | 2.871E-03 | 1.4 |
| 5 | P1 | 163 | 7 (156,163) | 4.3 | 2.020E-02 | 2.886E-03 | 1.2 |
| 6 | P1 | 169 | 6 (163,169) | 3.6 | 2.010E-02 | 3.350E-03 | 1.4 |
| 7 | P1 | 173 | 8 (165,173) | 4.6 | 1.980E-02 | 2.475E-03 | 1.2 |
| 8 | P1 | 177 | 6 (171,177) | 3.4 | 1.960E-02 | 3.267E-03 | 1.3 |
| 9 | P1 | 183 | 8 (175,183) | 4.4 | 1.900E-02 | 2.375E-03 | 1.2 |
| 10 | P2 | 222 | 7 (215,222) | 3.1 | 1.588E-02 | 2.335E-03 | 0.9 |
| 11 | P2 | 227 | 7 (220,227) | 3.3 | 1.663E-02 | 2.247E-03 | 1.0 |
| 12 | P2 | 232 | 8 (224,232) | 3.4 | 1.700E-02 | 2.152E-03 | 1.1 |
| 13 | P2 | 235 | 6 (228,235) | 2.8 | 1.683E-02 | 2.589E-03 | 0.9 |
| 14 | P2 | 240 | 7 (233,240) | 3.0 | 1.689E-02 | 2.379E-03 | 0.9 |
| 15 | P2 | 243 | 6 (237,243) | 2.3 | 1.690E-02 | 3.018E-03 | 1.1 |
| 16 | P2 | 249 | 7 (242,249) | 2.9 | 1.688E-02 | 2.344E-03 | 0.9 |
| 17 | P2 | 254 | 8 (246,254) | 3.1 | 1.693E-02 | 2.171E-03 | 0.9 |
| 18 | P2 | 257 | 6 (251,257) | 2.5 | 1.659E-02 | 2.592E-03 | 0.9 |
| 19 | P2 | 263 | 8 (255,263) | 3.0 | 1.668E-02 | 2.111E-03 | 0.8 |
| 20 | P2 | 266 | 6 (260,266) | 2.4 | 1.646E-02 | 2.532E-03 | 0.9 |
| 21 | P2 | 272 | 8 (264,272) | 3.0 | 1.654E-02 | 2.042E-03 | 0.8 |
| 22 | P2 | 275 | 7 (268,275) | 2.4 | 1.614E-02 | 2.409E-03 | 0.8 |
| 23 | P2 | 282 | 9 (273,282) | 3.3 | 1.568E-02 | 1.704E-03 | 0.8 |
| 24 | P3 | 323 | 9 (314,323) | 2.8 | 1.336E-02 | 1.452E-03 | 0.7 |
| 25 | P3 | 327 | 9 (318,327) | 2.6 | 1.381E-02 | 1.606E-03 | 0.8 |
| 26 | P3 | 330 | 8 (322,330) | 2.4 | 1.426E-02 | 1.783E-03 | 0.8 |
| 27 | P3 | 338 | 11 (327,338) | 3.4 | 1.453E-02 | 1.275E-03 | 0.9 |
| 28 | P3 | 342 | 11 (331,342) | 3.2 | 1.472E-02 | 1.326E-03 | 0.7 |
| 29 | P3 | 346 | 11 (335,346) | 3.1 | 1.463E-02 | 1.355E-03 | 0.6 |
| 30 | P3 | 350 | 10 (340,350) | 3.0 | 1.458E-02 | 1.389E-03 | 0.6 |
| 31 | P3 | 354 | 10 (344,354) | 2.8 | 1.447E-02 | 1.462E-03 | 0.6 |
| 32 | P3 | 358 | 10 (348,358) | 2.8 | 1.426E-02 | 1.440E-03 | 0.6 |
| 33 | P3 | 362 | 10 (352,362) | 2.7 | 1.432E-02 | 1.476E-03 | 0.6 |
| 34 | P3 | 366 | 9 (357,366) | 2.6 | 1.411E-02 | 1.501E-03 | 0.6 |
| 35 | P3 | 370 | 9 (361,370) | 2.5 | 1.414E-02 | 1.554E-03 | 0.7 |
| 36 | P3 | 374 | 9 (365,374) | 2.4 | 1.417E-02 | 1.610E-03 | 0.6 |
| 37 | P3 | 379 | 10 (370,379) | 2.5 | 1.408E-02 | 1.482E-03 | 0.6 |
| 38 | P3 | 383 | 9 (374,383) | 2.4 | 1.387E-02 | 1.491E-03 | 0.7 |
| 39 | P3 | 387 | 9 (378,387) | 2.3 | 1.352E-02 | 1.502E-03 | 0.7 |
| 40 | P4 | 442 | 10 (432,442) | 2.3 | 1.248E-02 | 1.236E-03 | 0.7 |
| 41 | P4 | 447 | 10 (436,447) | 2.3 | 1.277E-02 | 1.228E-03 | 0.8 |
| 42 | P4 | 452 | 11 (441,452) | 2.4 | 1.298E-02 | 1.213E-03 | 0.8 |
| 43 | P4 | 456 | 11 (445,456) | 2.4 | 1.315E-02 | 1.206E-03 | 0.7 |
| 44 | P4 | 460 | 11 (449,460) | 2.4 | 1.333E-02 | 1.190E-03 | 0.5 |
| 45 | P4 | 465 | 11 (454,465) | 2.5 | 1.314E-02 | 1.153E-03 | 0.4 |
| 46 | P4 | 470 | 12 (458,470) | 2.5 | 1.320E-02 | 1.128E-03 | 0.4 |
| 47 | P4 | 474 | 12 (462,474) | 2.5 | 1.326E-02 | 1.105E-03 | 0.6 |
| 48 | P4 | 479 | 12 (466,479) | 2.5 | 1.314E-02 | 1.077E-03 | 0.7 |
| 49 | P4 | 483 | 12 (471,483) | 2.6 | 1.286E-02 | 1.029E-03 | 0.6 |
| 50 | P4 | 488 | 13 (475,488) | 2.6 | 1.281E-02 | 1.009E-03 | 0.6 |
| 51 | P4 | 492 | 13 (479,492) | 2.6 | 1.266E-02 | 9.814E-04 | 0.4 |
| 52 | P4 | 497 | 13 (483,497) | 2.7 | 1.278E-02 | 9.609E-04 | 0.5 |
| 53 | P4 | 501 | 14 (488,501) | 2.7 | 1.265E-02 | 9.370E-04 | 0.7 |
| 54 | P4 | 506 | 14 (492,506) | 2.7 | 1.231E-02 | 8.920E-04 | 0.6 |
| 55 | P4 | 510 | 14 (496,510) | 2.7 | 1.260E-02 | 9.000E-04 | 0.5 |
| 56 | P4 | 515 | 14 (500,515) | 2.8 | 1.230E-02 | 8.662E-04 | 0.4 |
| 57 | P4 | 519 | 15 (505,519) | 2.8 | 1.205E-02 | 8.310E-04 | 0.6 |
| 58 | P5 | 577 | 19 (558,577) | 3.3 | 2.142E-02 | 1.127E-03 | 0.7 |
| 59 | P5 | 584 | 14 (570,584) | 2.4 | 2.187E-02 | 1.562E-03 | 0.4 |
| 60 | P5 | 590 | 13 (577,590) | 2.2 | 2.215E-02 | 1.704E-03 | 0.7 |
| 61 | P5 | 604 | 20 (584,604) | 3.3 | 2.207E-02 | 1.103E-03 | 0.8 |

| | | | | | | | |
|---|---|---|---|---|---|---|---|
| 62 | P5 | 611 | 14 (597,611) | 2.3 | 2.181E-02 | 1.558E-03 | 0.5 |
| 63 | P5 | 618 | 14 (604,618) | 2.3 | 2.187E-02 | 1.562E-03 | 0.3 |
| 64 | P5 | 625 | 21 (611,632) | 3.4 | 2.138E-02 | 1.018E-03 | 0.7 |
| 65 | P5 | 632 | 21 (618,639) | 3.3 | 2.098E-02 | 9.990E-04 | 0.9 |
| 66 | P5 | 646 | 14 (632,646) | 2.2 | 2.091E-02 | 1.494E-03 | 0.7 |
| 67 | P5 | 654 | 15 (639,654) | 2.3 | 2.041E-02 | 1.361E-03 | 0.4 |
| 68 | P6 | 708 | 16 (692,708) | 2.3 | 1.859E-02 | 1.162E-03 | 0.3 |
| 69 | P6 | 716 | 16 (700,716) | 2.2 | 1.904E-02 | 1.190E-03 | 0.4 |
| 70 | P6 | 724 | 25 (708,733) | 3.5 | 1.966E-02 | 7.864E-04 | 0.5 |
| 71 | P6 | 733 | 25 (716,741) | 3.4 | 1.946E-02 | 7.784E-04 | 0.6 |
| 72 | P6 | 741 | 25 (724,749) | 3.4 | 1.940E-02 | 7.760E-04 | 0.9 |
| 73 | P6 | 749 | 17 (741,758) | 2.3 | 1.920E-02 | 1.129E-03 | 1.0 |
| 74 | P6 | 767 | 18 (749,767) | 2.3 | 1.935E-02 | 1.075E-03 | 0.9 |
| 75 | P6 | 775 | 17 (758,775) | 2.2 | 1.884E-02 | 1.108E-03 | 0.8 |
| 76 | P6 | 784 | 17 (767,784) | 2.2 | 1.892E-02 | 1.113E-03 | 0.8 |
| 77 | P6 | 793 | 18 (775,793) | 2.3 | 1.809E-02 | 1.005E-03 | 0.7 |
| 78 | P7 | 869 | 22 (849,871) | 2.5 | 1.566E-02 | 7.151E-04 | 0.5 |
| 79 | P7 | 878 | 22 (858,880) | 2.5 | 1.639E-02 | 7.383E-04 | 0.7 |
| 80 | P7 | 887 | 23 (867,889) | 2.5 | 1.628E-02 | 7.204E-04 | 0.8 |
| 81 | P7 | 895 | 23 (875,898) | 2.6 | 1.620E-02 | 7.013E-04 | 1.1 |
| 82 | P7 | 904 | 23 (884,907) | 2.6 | 1.590E-02 | 6.795E-04 | 1.0 |
| 83 | P7 | 913 | 24 (892,916) | 2.6 | 1.602E-02 | 6.731E-04 | 0.8 |
| 84 | P7 | 922 | 24 (901,925) | 2.6 | 1.597E-02 | 6.627E-04 | 0.5 |
| 85 | P7 | 931 | 25 (910,934) | 2.6 | 1.557E-02 | 6.329E-04 | 0.4 |
| 86 | P7 | 940 | 25 (918,943) | 2.7 | 1.537E-02 | 6.148E-04 | 0.7 |
| 87 | P7 | 948 | 25 (927,952) | 2.7 | 1.537E-02 | 6.075E-04 | 0.8 |
| 88 | P8 | 1020 | 28 (999,1028) | 2.8 | 1.282E-02 | 4.546E-04 | 0.7 |
| 89 | P8 | 1029 | 28 (1008,1036) | 2.7 | 1.342E-02 | 4.776E-04 | 0.6 |
| 90 | P8 | 1038 | 28 (1016,1044) | 2.7 | 1.325E-02 | 4.749E-04 | 0.8 |
| 91 | P8 | 1047 | 28 (1025,1053) | 2.7 | 1.300E-02 | 4.676E-04 | 1.2 |
| 92 | P8 | 1056 | 28 (1034,1061) | 2.6 | 1.320E-02 | 4.783E-04 | 0.4 |
| 93 | P8 | 1065 | 27 (1042,1070) | 2.6 | 1.319E-02 | 4.814E-04 | 0.8 |
| 94 | P8 | 1074 | 27 (1051,1078) | 2.5 | 1.340E-02 | 4.926E-04 | 1.1 |
| 95 | P8 | 1083 | 27 (1059,1086) | 2.5 | 1.298E-02 | 4.790E-04 | 0.8 |
| 96 | P8 | 1092 | 27 (1068,1095) | 2.5 | 1.290E-02 | 4.796E-04 | 0.5 |
| 97 | P8 | 1101 | 27 (1076,1103) | 2.4 | 1.290E-02 | 4.813E-04 | 1.3 |

Table 14: Energy/Flux Calibration Factors for the Histogram Electron Channels. Histogram LUT ID (M75-B): 26880.

| CH # | PIX | $E$ [keV] | $\Delta E$ ($E_{lo}$,$E_{hi}$) [keV] | $\Delta E/E$ [%] | $G_0\Delta E$ [cm$^2$ sr keV] | $G_0$ [cm$^2$ sr] | $\Delta G_0/G_0$ [%] |
|---|---|---|---|---|---|---|---|
| 0 | P1 | 136 | 11 (127,138) | 8.1 | 3.740E-02 | 3.400E-03 | 1.7 |
| 1 | P1 | 146 | 11 (136,147) | 7.5 | 4.030E-02 | 3.664E-03 | 1.4 |
| 2 | P1 | 156 | 12 (146,158) | 7.7 | 4.050E-02 | 3.375E-03 | 1.3 |
| 3 | P1 | 165 | 11 (156,167) | 6.7 | 4.000E-02 | 3.636E-03 | 1.3 |
| 4 | P1 | 175 | 12 (165,177) | 6.9 | 3.940E-02 | 3.283E-03 | 1.2 |
| 5 | P2 | 224 | 10 (214,224) | 4.5 | 3.247E-02 | 3.247E-03 | 0.9 |
| 6 | P2 | 232 | 13 (222,235) | 5.6 | 3.360E-02 | 2.585E-03 | 1.0 |
| 7 | P2 | 243 | 11 (232,243) | 4.4 | 3.406E-02 | 3.154E-03 | 1.0 |
| 8 | P2 | 251 | 11 (240,251) | 4.4 | 3.381E-02 | 3.074E-03 | 0.8 |
| 9 | P2 | 260 | 11 (249,260) | 4.2 | 3.328E-02 | 3.025E-03 | 0.8 |
| 10 | P2 | 269 | 12 (257,269) | 4.5 | 3.301E-02 | 2.751E-03 | 0.7 |
| 11 | P2 | 279 | 13 (266,279) | 4.7 | 3.182E-02 | 2.448E-03 | 0.7 |
| 12 | P3 | 330 | 15 (316,330) | 4.4 | 2.833E-02 | 1.927E-03 | 0.6 |
| 13 | P3 | 338 | 11 (327,338) | 3.3 | 2.906E-02 | 2.572E-03 | 0.6 |
| 14 | P3 | 346 | 12 (334,346) | 3.5 | 2.890E-02 | 2.408E-03 | 0.8 |
| 15 | P3 | 358 | 16 (342,358) | 4.5 | 2.892E-02 | 1.808E-03 | 0.8 |
| 16 | P3 | 366 | 12 (354,366) | 3.3 | 2.867E-02 | 2.389E-03 | 0.7 |
| 17 | P3 | 374 | 12 (362,374) | 3.2 | 2.855E-02 | 2.379E-03 | 0.6 |
| 18 | P3 | 383 | 13 (370,383) | 3.4 | 2.820E-02 | 2.169E-03 | 0.6 |
| 19 | P3 | 392 | 13 (379,392) | 3.3 | 2.688E-02 | 2.068E-03 | 0.6 |
| 20 | P4 | 444 | 15 (429,444) | 3.4 | 2.508E-02 | 1.672E-03 | 0.5 |
| 21 | P4 | 454 | 15 (439,454) | 3.3 | 2.639E-02 | 1.759E-03 | 0.4 |
| 22 | P4 | 465 | 16 (449,465) | 3.4 | 2.667E-02 | 1.667E-03 | 0.7 |
| 23 | P4 | 470 | 10 (460,470) | 2.1 | 2.622E-02 | 2.622E-03 | 0.6 |

| | | | | | | | |
|---|---|---|---|---|---|---|---|
| 24 | P4 | 481 | 16 (465,481) | 3.3 | 2.603E-02 | 1.627E-03 | 0.4 |
| 25 | P4 | 492 | 17 (475,492) | 3.5 | 2.568E-02 | 1.511E-03 | 0.6 |
| 26 | P4 | 498 | 12 (486,498) | 2.4 | 2.521E-02 | 2.101E-03 | 0.5 |
| 27 | P4 | 509 | 17 (492,509) | 3.3 | 2.513E-02 | 1.478E-03 | 0.5 |
| 28 | P4 | 515 | 12 (503,515) | 2.3 | 2.413E-02 | 2.011E-03 | 0.7 |
| 29 | P5 | 577 | 19 (558,577) | 3.3 | 2.142E-02 | 1.127E-03 | 0.7 |
| 30 | P5 | 584 | 14 (570,584) | 2.4 | 2.187E-02 | 1.562E-03 | 0.4 |
| 31 | P5 | 590 | 13 (577,590) | 2.2 | 2.215E-02 | 1.704E-03 | 0.7 |
| 32 | P5 | 604 | 20 (584,604) | 3.3 | 2.207E-02 | 1.103E-03 | 0.8 |
| 33 | P5 | 611 | 14 (597,611) | 2.3 | 2.181E-02 | 1.558E-03 | 0.5 |
| 34 | P5 | 618 | 14 (604,618) | 2.3 | 2.187E-02 | 1.562E-03 | 0.3 |
| 35 | P5 | 625 | 21 (611,632) | 3.4 | 2.138E-02 | 1.018E-03 | 0.7 |
| 36 | P5 | 632 | 21 (618,639) | 3.3 | 2.098E-02 | 9.990E-04 | 0.9 |
| 37 | P5 | 646 | 14 (632,646) | 2.2 | 2.091E-02 | 1.494E-03 | 0.7 |
| 38 | P5 | 654 | 15 (639,654) | 2.3 | 2.041E-02 | 1.361E-03 | 0.4 |
| 39 | P6 | 708 | 16 (692,708) | 2.3 | 1.859E-02 | 1.162E-03 | 0.3 |
| 40 | P6 | 716 | 16 (700,716) | 2.2 | 1.904E-02 | 1.190E-03 | 0.4 |
| 41 | P6 | 724 | 25 (708,733) | 3.5 | 1.966E-02 | 7.864E-04 | 0.5 |
| 42 | P6 | 733 | 25 (716,741) | 3.4 | 1.946E-02 | 7.784E-04 | 0.6 |
| 43 | P6 | 741 | 25 (724,749) | 3.4 | 1.940E-02 | 7.760E-04 | 0.9 |
| 44 | P6 | 749 | 17 (741,758) | 2.3 | 1.920E-02 | 1.129E-03 | 1.0 |
| 45 | P6 | 767 | 18 (749,767) | 2.3 | 1.935E-02 | 1.075E-03 | 0.9 |
| 46 | P6 | 775 | 17 (758,775) | 2.2 | 1.884E-02 | 1.108E-03 | 0.8 |
| 47 | P6 | 784 | 17 (767,784) | 2.2 | 1.892E-02 | 1.113E-03 | 0.8 |
| 48 | P6 | 793 | 18 (775,793) | 2.3 | 1.809E-02 | 1.005E-03 | 0.7 |
| 49 | P7 | 861 | 26 (841,868) | 3.1 | 1.500E-02 | 5.660E-04 | 1.1 |
| 50 | P7 | 870 | 26 (851,877) | 3.0 | 1.583E-02 | 6.088E-04 | 0.8 |
| 51 | P7 | 880 | 26 (860,885) | 2.9 | 1.621E-02 | 6.332E-04 | 0.6 |
| 52 | P7 | 889 | 25 (869,894) | 2.8 | 1.612E-02 | 6.422E-04 | 0.4 |
| 53 | P7 | 898 | 25 (878,903) | 2.7 | 1.600E-02 | 6.504E-04 | 0.4 |
| 54 | P7 | 907 | 24 (888,912) | 2.7 | 1.624E-02 | 6.711E-04 | 0.6 |
| 55 | P7 | 916 | 24 (897,920) | 2.6 | 1.605E-02 | 6.772E-04 | 0.7 |
| 56 | P7 | 925 | 23 (906,929) | 2.5 | 1.580E-02 | 6.781E-04 | 1.1 |
| 57 | P7 | 934 | 23 (915,938) | 2.4 | 1.560E-02 | 6.842E-04 | 1.1 |
| 58 | P7 | 944 | 22 (924,947) | 2.4 | 1.503E-02 | 6.710E-04 | 0.7 |
| 59 | P7 | 953 | 22 (934,956) | 2.3 | 1.495E-02 | 6.858E-04 | 0.4 |
| 60 | P8 | 1020 | 28 (999,1028) | 2.8 | 1.282E-02 | 4.546E-04 | 0.7 |
| 61 | P8 | 1029 | 28 (1008,1036) | 2.7 | 1.342E-02 | 4.776E-04 | 0.6 |
| 62 | P8 | 1038 | 28 (1016,1044) | 2.7 | 1.325E-02 | 4.749E-04 | 0.8 |
| 63 | P8 | 1047 | 28 (1025,1053) | 2.7 | 1.300E-02 | 4.676E-04 | 1.2 |
| 64 | P8 | 1056 | 28 (1034,1061) | 2.6 | 1.320E-02 | 4.783E-04 | 0.4 |
| 65 | P8 | 1065 | 27 (1042,1070) | 2.6 | 1.319E-02 | 4.814E-04 | 0.8 |
| 66 | P8 | 1074 | 27 (1051,1078) | 2.5 | 1.340E-02 | 4.926E-04 | 1.1 |
| 67 | P8 | 1083 | 27 (1059,1086) | 2.5 | 1.298E-02 | 4.790E-04 | 0.8 |
| 68 | P8 | 1092 | 27 (1068,1095) | 2.5 | 1.290E-02 | 4.796E-04 | 0.5 |
| 69 | P8 | 1101 | 27 (1076,1103) | 2.4 | 1.290E-02 | 4.813E-04 | 1.3 |

## Probe B (HIGH)

Table 15: Energy/Flux Calibration Factors for the Histogram Electron Channels. Histogram LUT ID (HIGH-B): 31490, for times between 2012/09/01 and 2013/08/03.

| CH # | PIX | $E$ [keV] | $\Delta E$ ($E_{lo}$,$E_{hi}$) [keV] | $\Delta E/E$ [%] | $G_0\Delta E$ [cm$^2$ sr keV] | $G_0$ [cm$^2$ sr] | $\Delta G_0/G_0$ [%] |
|---|---|---|---|---|---|---|---|
| 0 | P0 | 970 | 108 (884,992) | 11.1 | 2.960E-01 | 2.741E-03 | 2.4 |
| 1 | P0 | 992 | 90 (926,1016) | 9.1 | 3.120E-01 | 3.467E-03 | 3.1 |
| 2 | P0 | 1039 | 91 (948,1039) | 8.8 | 3.750E-01 | 4.121E-03 | 1.9 |
| 3 | P0 | 1064 | 96 (992,1088) | 9.0 | 3.620E-01 | 3.771E-03 | 2.2 |
| 4 | P0 | 1114 | 75 (1039,1114) | 6.7 | 4.020E-01 | 5.360E-03 | 1.9 |
| 5 | P0 | 1140 | 103 (1064,1167) | 9.0 | 3.790E-01 | 3.680E-03 | 1.3 |
| 6 | P0 | 1194 | 108 (1114,1222) | 9.0 | 3.860E-01 | 3.574E-03 | 2.2 |
| 7 | P0 | 1222 | 110 (1140,1250) | 9.0 | 3.353E-01 | 3.048E-03 | 0.9 |
| 8 | P0 | 1250 | 85 (1194,1279) | 6.8 | 2.860E-01 | 3.365E-03 | 2.3 |
| 9 | P1 | 1579 | 139 (1440,1579) | 8.8 | 3.240E-01 | 2.331E-03 | 1.4 |
| 10 | P1 | 1611 | 108 (1504,1612) | 6.7 | 3.630E-01 | 3.361E-03 | 1.4 |
| 11 | P1 | 1642 | 111 (1539,1650) | 6.8 | 3.980E-01 | 3.586E-03 | 1.9 |
| 12 | P1 | 1674 | 113 (1575,1688) | 6.8 | 4.150E-01 | 3.673E-03 | 2.2 |
| 13 | P1 | 1706 | 116 (1612,1728) | 6.8 | 3.610E-01 | 3.112E-03 | 2.1 |
| 14 | P1 | 1737 | 118 (1650,1768) | 6.8 | 3.520E-01 | 2.983E-03 | 1.8 |
| 15 | P1 | 1769 | 121 (1688,1809) | 6.8 | 3.230E-01 | 2.669E-03 | 1.3 |
| 16 | P1 | 1800 | 124 (1728,1852) | 6.9 | 2.910E-01 | 2.347E-03 | 1.2 |
| 17 | P2 | 2404 | 214 (2194,2408) | 8.9 | 8.510E-02 | 3.969E-04 | 2.5 |
| 18 | P2 | 2442 | 213 (2236,2448) | 8.7 | 9.000E-02 | 4.225E-04 | 1.7 |
| 19 | P2 | 2480 | 212 (2277,2489) | 8.5 | 9.970E-02 | 4.707E-04 | 2.6 |
| 20 | P2 | 2517 | 210 (2318,2529) | 8.4 | 9.300E-02 | 4.418E-04 | 1.7 |
| 21 | P2 | 2555 | 209 (2360,2569) | 8.2 | 1.000E-01 | 4.782E-04 | 1.9 |
| 22 | P2 | 2593 | 208 (2401,2609) | 8.0 | 9.790E-02 | 4.709E-04 | 2.3 |
| 23 | P2 | 2630 | 207 (2443,2649) | 7.9 | 9.410E-02 | 4.555E-04 | 1.6 |
| 24 | P2 | 2668 | 205 (2484,2690) | 7.7 | 1.120E-01 | 5.455E-04 | 1.9 |
| 25 | P2 | 2706 | 204 (2526,2730) | 7.5 | 8.850E-02 | 4.338E-04 | 2.0 |
| 26 | P2 | 2744 | 203 (2567,2770) | 7.4 | 8.810E-02 | 4.344E-04 | 2.0 |
| 27 | P2 | 2782 | 202 (2609,2810) | 7.2 | 9.130E-02 | 4.531E-04 | 2.0 |
| 28 | P2 | 2819 | 200 (2650,2850) | 7.1 | 8.530E-02 | 4.263E-04 | 2.5 |
| 29 | P2 | 2857 | 199 (2692,2890) | 7.0 | 8.680E-02 | 4.364E-04 | 1.7 |
| 30 | P2 | 2895 | 198 (2733,2931) | 6.8 | 7.460E-02 | 3.775E-04 | 2.9 |
| 31 | P2 | 2933 | 196 (2774,2971) | 6.7 | 8.170E-02 | 4.162E-04 | 1.7 |
| 32 | P3 | 3717 | 460 (3393,3853) | 12.4 | 7.090E-02 | 1.542E-04 | 5.1 |
| 33 | P3 | 3756 | 449 (3437,3886) | 12.0 | 8.440E-02 | 1.879E-04 | 5.0 |
| 34 | P3 | 3795 | 438 (3480,3918) | 11.6 | 8.360E-02 | 1.907E-04 | 4.4 |
| 35 | P3 | 3834 | 427 (3524,3951) | 11.1 | 7.580E-02 | 1.774E-04 | 5.0 |
| 36 | P3 | 3873 | 417 (3567,3984) | 10.8 | 8.790E-02 | 2.110E-04 | 5.2 |
| 37 | P3 | 3912 | 406 (3610,4016) | 10.4 | 7.910E-02 | 1.949E-04 | 4.3 |
| 38 | P3 | 3951 | 395 (3654,4049) | 10.0 | 9.380E-02 | 2.375E-04 | 4.8 |
| 39 | P3 | 3990 | 384 (3697,4081) | 9.6 | 9.440E-02 | 2.458E-04 | 3.6 |
| 40 | P3 | 4028 | 373 (3740,4114) | 9.3 | 9.080E-02 | 2.432E-04 | 3.8 |
| 41 | P3 | 4068 | 362 (3784,4146) | 8.9 | 8.240E-02 | 2.273E-04 | 3.8 |
| 42 | P3 | 4106 | 352 (3827,4179) | 8.6 | 8.070E-02 | 2.295E-04 | 4.0 |
| 43 | P3 | 4146 | 341 (3870,4211) | 8.2 | 8.720E-02 | 2.559E-04 | 4.0 |
| 44 | P3 | 4184 | 330 (3914,4244) | 7.9 | 7.720E-02 | 2.339E-04 | 4.1 |
| 45 | P3 | 4224 | 319 (3957,4276) | 7.6 | 8.640E-02 | 2.708E-04 | 4.8 |
| 46 | P3 | 4262 | 308 (4000,4309) | 7.2 | 8.360E-02 | 2.712E-04 | 3.5 |
| 47 | P3 | 4302 | 297 (4044,4341) | 6.9 | 7.850E-02 | 2.639E-04 | 4.8 |
| 48 | P3 | 4340 | 287 (4087,4374) | 6.6 | 8.210E-02 | 2.865E-04 | 3.7 |
| 49 | P3 | 4380 | 276 (4131,4406) | 6.3 | 6.880E-02 | 2.495E-04 | 5.4 |
| 50 | P3 | 4418 | 265 (4174,4439) | 6.0 | 7.140E-02 | 2.694E-04 | 3.6 |

Table 16: Energy/Flux Calibration Factors for the Histogram Electron Channels. Histogram LUT ID (HIGH-B): 31490, for times between 2013/08/03 and 2019/07/16.

| CH # | PIX | $E$ [keV] | $\Delta E$ ($E_{lo}$,$E_{hi}$) [keV] | $\Delta E/E$ [%] | $G_0\Delta E$ [cm$^2$ sr keV] | $G_0$ [cm$^2$ sr] | $\Delta G_0/G_0$ [%] |
|---|---|---|---|---|---|---|---|

| | | | | | | | |
|---|---|---|---|---|---|---|---|
| 0 | P0 | 970 | 108 (884,992) | 11.1 | 2.970E-01 | 2.750E-03 | 2.4 |
| 1 | P0 | 992 | 90 (926,1016) | 9.1 | 3.130E-01 | 3.478E-03 | 3.1 |
| 2 | P0 | 1039 | 91 (948,1039) | 8.8 | 3.770E-01 | 4.143E-03 | 1.9 |
| 3 | P0 | 1064 | 96 (992,1088) | 9.0 | 3.640E-01 | 3.792E-03 | 2.2 |
| 4 | P0 | 1114 | 75 (1039,1114) | 6.7 | 4.040E-01 | 5.387E-03 | 1.9 |
| 5 | P0 | 1140 | 103 (1064,1167) | 9.0 | 3.820E-01 | 3.709E-03 | 1.3 |
| 6 | P0 | 1194 | 108 (1114,1222) | 9.0 | 3.890E-01 | 3.602E-03 | 2.2 |
| 7 | P0 | 1222 | 110 (1140,1250) | 9.0 | 3.389E-01 | 3.081E-03 | 0.9 |
| 8 | P0 | 1250 | 85 (1194,1279) | 6.8 | 2.880E-01 | 3.388E-03 | 2.2 |
| 9 | P1 | 1579 | 139 (1440,1579) | 8.8 | 3.240E-01 | 2.331E-03 | 1.4 |
| 10 | P1 | 1611 | 108 (1504,1612) | 6.7 | 3.630E-01 | 3.361E-03 | 1.4 |
| 11 | P1 | 1642 | 111 (1539,1650) | 6.8 | 3.980E-01 | 3.586E-03 | 1.9 |
| 12 | P1 | 1674 | 113 (1575,1688) | 6.8 | 4.150E-01 | 3.673E-03 | 2.2 |
| 13 | P1 | 1706 | 116 (1612,1728) | 6.8 | 3.610E-01 | 3.112E-03 | 2.1 |
| 14 | P1 | 1737 | 118 (1650,1768) | 6.8 | 3.520E-01 | 2.983E-03 | 1.8 |
| 15 | P1 | 1769 | 121 (1688,1809) | 6.8 | 3.230E-01 | 2.669E-03 | 1.3 |
| 16 | P1 | 1800 | 124 (1728,1852) | 6.9 | 2.910E-01 | 2.347E-03 | 1.2 |
| 17 | P2 | 2267 | 165 (2103,2267) | 7.3 | 2.560E-01 | 1.554E-03 | 1.6 |
| 18 | P2 | 2308 | 164 (2144,2308) | 7.1 | 3.060E-01 | 1.872E-03 | 1.6 |
| 19 | P2 | 2348 | 162 (2186,2348) | 6.9 | 2.930E-01 | 1.805E-03 | 2.1 |
| 20 | P2 | 2389 | 161 (2228,2389) | 6.7 | 3.240E-01 | 2.010E-03 | 1.2 |
| 21 | P2 | 2429 | 160 (2269,2429) | 6.6 | 3.580E-01 | 2.236E-03 | 1.5 |
| 22 | P2 | 2470 | 159 (2312,2470) | 6.4 | 3.310E-01 | 2.083E-03 | 2.3 |
| 23 | P2 | 2510 | 158 (2354,2512) | 6.3 | 3.640E-01 | 2.308E-03 | 1.1 |
| 24 | P2 | 2550 | 157 (2397,2553) | 6.1 | 3.570E-01 | 2.280E-03 | 1.3 |
| 25 | P2 | 2591 | 155 (2439,2595) | 6.0 | 3.380E-01 | 2.175E-03 | 2.4 |
| 26 | P2 | 2631 | 154 (2482,2636) | 5.9 | 3.420E-01 | 2.216E-03 | 1.2 |
| 27 | P2 | 2672 | 153 (2524,2677) | 5.7 | 3.530E-01 | 2.307E-03 | 1.3 |
| 28 | P2 | 2712 | 152 (2567,2719) | 5.6 | 3.560E-01 | 2.344E-03 | 2.4 |
| 29 | P2 | 2752 | 151 (2610,2760) | 5.5 | 3.040E-01 | 2.017E-03 | 1.6 |
| 30 | P2 | 2793 | 150 (2652,2802) | 5.4 | 3.140E-01 | 2.099E-03 | 1.3 |
| 31 | P2 | 2833 | 148 (2695,2843) | 5.2 | 2.790E-01 | 1.880E-03 | 2.4 |
| 32 | P2 | 2874 | 147 (2737,2884) | 5.1 | 2.760E-01 | 1.875E-03 | 1.2 |
| 33 | P2 | 2914 | 146 (2780,2926) | 5.0 | 2.750E-01 | 1.882E-03 | 2.0 |
| 34 | P3 | 3572 | 250 (3348,3597) | 7.0 | 2.750E-01 | 1.102E-03 | 3.4 |
| 35 | P3 | 3616 | 248 (3390,3639) | 6.9 | 2.890E-01 | 1.163E-03 | 3.3 |
| 36 | P3 | 3659 | 247 (3433,3680) | 6.8 | 3.450E-01 | 1.395E-03 | 3.9 |
| 37 | P3 | 3702 | 246 (3476,3722) | 6.6 | 3.240E-01 | 1.317E-03 | 2.8 |
| 38 | P3 | 3745 | 245 (3518,3763) | 6.5 | 3.110E-01 | 1.270E-03 | 4.3 |
| 39 | P3 | 3788 | 244 (3561,3804) | 6.4 | 3.700E-01 | 1.518E-03 | 2.6 |
| 40 | P3 | 3832 | 242 (3604,3846) | 6.3 | 3.290E-01 | 1.357E-03 | 4.2 |
| 41 | P3 | 3875 | 241 (3646,3888) | 6.2 | 3.910E-01 | 1.620E-03 | 2.7 |
| 42 | P3 | 3918 | 240 (3689,3929) | 6.1 | 3.510E-01 | 1.462E-03 | 3.6 |
| 43 | P3 | 3962 | 239 (3731,3970) | 6.0 | 3.770E-01 | 1.577E-03 | 2.9 |
| 44 | P3 | 4005 | 238 (3774,4012) | 5.9 | 3.230E-01 | 1.358E-03 | 3.7 |
| 45 | P3 | 4048 | 237 (3817,4053) | 5.8 | 3.770E-01 | 1.593E-03 | 3.2 |
| 46 | P3 | 4091 | 235 (3859,4095) | 5.8 | 3.420E-01 | 1.453E-03 | 2.9 |
| 47 | P3 | 4134 | 234 (3902,4136) | 5.7 | 3.500E-01 | 1.494E-03 | 2.9 |
| 48 | P3 | 4178 | 233 (3945,4178) | 5.6 | 3.100E-01 | 1.330E-03 | 3.4 |
| 49 | P3 | 4221 | 232 (3989,4221) | 5.5 | 3.340E-01 | 1.441E-03 | 3.8 |
| 50 | P3 | 4264 | 231 (4034,4264) | 5.4 | 3.030E-01 | 1.314E-03 | 2.4 |
| 51 | P3 | 4308 | 230 (4078,4308) | 5.3 | 3.160E-01 | 1.377E-03 | 4.6 |
