# Supplementary material for: The Magnetic Electron Ion Spectrometer: A Review of On-Orbit Sensor Performance, Data, Operations, and Science
Source: Space Sci Rev. 2021 Oct 28;217(8):80. doi: 10.1007/s11214-021-00855-2 (PMC8553741; doi:10.1007/s11214-021-00855-2)
Supplement: Supplementary file 4 — Tables of the calibration (energy channel definitions/flux conversion) factors for all of the LUTs used on orbit for the electron main rates (PDF 107 kB) [file 11214_2021_855_MOESM4_ESM.pdf]

MagEIS Calibration Factors: Electron Main Rates

June 18, 2021

Table 1 through Table 13 provide energy channel definitions and flux conversion factors for the merged main rate electron channels on both Probes. Note the valid time ranges indicated in the table captions. These tables represent all of the unique combinations of LOW, M75 and HIGH unit lookup tables that were used throughout the mission. For completeness, we also provide the analogous set of tables for the M35 unit (Table 14 to Table 17), which was not used in the merged product.

Probe A (LOW/M75/HIGH)

Table 1: Energy/Flux Calibration Factors for the Merged Electron Channels on Probe A. Valid from 19 Sep 2012 00:00:00 to 04 Oct 2012 19:00:00. Main rate LUT IDs (LOW/M75/HIGH): 16385/24577/29697.

CH #	UNIT-PIX	E [keV]	ΔE (E_{lo},E_{hi}) [keV]	$\Delta E/E$ [%]	$G_0 \Delta E$ [cm^2 sr keV]	G_0 [cm^2 sr]	$\Delta G_0/G_0$ [%]
0	LOW-P0	X	X	X	X	X	X
1	LOW-P1	23	10 (19,29)	43	3.610E-02	3.610E-03	1.6
2	LOW-P2	38	9 (34,43)	24	2.639E-02	2.932E-03	0.9
3	LOW-P3	58	13 (52,65)	22	3.721E-02	2.862E-03	0.6
4	LOW-P4	82	16 (74,90)	19	3.848E-02	2.435E-03	0.4
5	LOW-P5	110	20 (101,121)	18	4.498E-02	2.249E-03	0.4
6	M75-P0	X	X	X	X	X	X
7	LOW-P6	145	22 (135,157)	15	3.920E-02	1.782E-03	1.0
8	M75-P1	152	46 (129,175)	30	1.840E-01	4.000E-03	2.1
9	LOW-P7	182	23 (172,195)	13	3.933E-02	1.710E-03	0.9
10	LOW-P8	221	26 (208,234)	12	3.433E-02	1.320E-03	0.9
11	M75-P2	240	59 (210,269)	25	2.040E-01	3.458E-03	1.5
12	M75-P3	338	72 (298,370)	21	2.141E-01	2.974E-03	0.9
13	M75-P4	460	77 (415,492)	17	2.005E-01	2.604E-03	0.7
14	M75-P5	597	87 (545,632)	15	1.913E-01	2.199E-03	1.0
15	M75-P6	741	92 (692,784)	12	1.740E-01	1.891E-03	1.0
16	M75-P7	879	100 (830,930)	11	1.517E-01	1.517E-03	0.9
17	M75-P8	1042	94 (985,1079)	9	1.380E-01	1.468E-03	1.3
18	HIGH-PXX	X	X	X	X	X	X
19	HIGH-PXX	X	X	X	X	X	X
20	HIGH-PXX	X	X	X	X	X	X
21	HIGH-PXX	X	X	X	X	X	X
22	HIGH-PXX	X	X	X	X	X	X
23	HIGH-PXX	X	X	X	X	X	X
24	HIGH-PXX	X	X	X	X	X	X

Table 2: Energy/Flux Calibration Factors for the Merged Electron Channels on Probe A. Valid from 04 Oct 2012 19:00:00 to 24 Feb 2013 00:00:00. Main rate LUT IDs (LOW/M75/HIGH): 16385/24578/29698.

CH #	UNIT-PIX	E [keV]	ΔE (E_{lo},E_{hi}) [keV]	$\Delta E/E$ [%]	$G_0\Delta E$ [cm^2 sr keV]	G_0 [cm^2 sr]	$\Delta G_0/G_0$ [%]
0	LOW-P0	X	X	X	X	X	X
1	LOW-P1	23	10 (19,29)	43	3.610E-02	3.610E-03	1.6
2	LOW-P2	38	9 (34,43)	24	2.639E-02	2.932E-03	0.9
3	LOW-P3	58	13 (52,65)	22	3.721E-02	2.862E-03	0.6
4	LOW-P4	82	16 (74,90)	19	3.848E-02	2.435E-03	0.4
5	LOW-P5	110	20 (101,121)	18	4.498E-02	2.249E-03	0.4
6	LOW-P6	145	22 (135,157)	15	3.920E-02	1.782E-03	1.0
7	LOW-P7	182	23 (172,195)	13	3.933E-02	1.710E-03	0.9
8	LOW-P8	221	26 (208,234)	12	3.433E-02	1.320E-03	0.9
9	M75-P2	222	27 (205,232)	12	8.520E-02	3.156E-03	1.3
10	M75-P2	249	30 (230,260)	12	1.040E-01	3.467E-03	1.1
11	M75-P3	323	36 (298,334)	11	9.929E-02	2.758E-03	0.9
12	M75-P3	354	36 (330,366)	10	1.018E-01	2.828E-03	0.9
13	M75-P4	460	71 (415,486)	15	1.890E-01	2.662E-03	0.9
14	M75-P5	584	86 (539,625)	15	1.844E-01	2.144E-03	0.9
15	M75-P6	741	83 (692,775)	11	1.671E-01	2.013E-03	0.9
16	M75-P7	879	99 (821,920)	11	1.457E-01	1.472E-03	0.9
17	HIGH-P0	1016	414 (926,1340)	41	1.440E+00	3.478E-03	6.1
18	M75-P8	1031	105 (974,1079)	10	1.380E-01	1.314E-03	1.4
19	HIGH-P1	1575	449 (1403,1852)	29	3.690E-01	8.218E-04	2.7
20	HIGH-P1	1728	320 (1575,1895)	19	4.020E-01	1.256E-03	2.0
21	HIGH-P2	2280	481 (2078,2559)	21	1.010E+00	2.100E-03	2.4
22	HIGH-P2	2680	621 (2387,3008)	23	1.190E+00	1.916E-03	2.5
23	HIGH-P3	3703	702 (3455,4157)	19	1.700E+00	2.422E-03	5.9
24	HIGH-P3	4062	962 (3703,4665)	24	2.280E+00	2.370E-03	4.2

Table 3: Energy/Flux Calibration Factors for the Merged Electron Channels on Probe A. Valid from 24 Feb 2013 00:00:00 to 31 Mar 2013 00:00:00. Main rate LUT IDs (LOW/M75/HIGH): 16385/24577/29698.

CH #	UNIT-PIX	E [keV]	ΔE (E_{lo},E_{hi}) [keV]	$\Delta E/E$ [%]	$G_0 \Delta E$ [cm^2 sr keV]	G_0 [cm^2 sr]	$\Delta G_0/G_0$ [%]
0	LOW-P0	X	X	X	X	X	X
1	LOW-P1	23	10 (19,29)	43	3.610E-02	3.610E-03	1.6
2	LOW-P2	38	9 (34,43)	24	2.639E-02	2.932E-03	0.9
3	LOW-P3	58	13 (52,65)	22	3.721E-02	2.862E-03	0.6
4	LOW-P4	82	16 (74,90)	19	3.848E-02	2.435E-03	0.4
5	LOW-P5	110	20 (101,121)	18	4.498E-02	2.249E-03	0.4
6	M75-P0	X	X	X	X	X	X
7	LOW-P6	145	22 (135,157)	15	3.920E-02	1.782E-03	1.0
8	M75-P1	152	46 (129,175)	30	1.840E-01	4.000E-03	2.1
9	LOW-P7	182	23 (172,195)	13	3.933E-02	1.710E-03	0.9
10	LOW-P8	221	26 (208,234)	12	3.433E-02	1.320E-03	0.9
11	M75-P2	240	59 (210,269)	25	2.040E-01	3.458E-03	1.5
12	M75-P3	338	72 (298,370)	21	2.141E-01	2.974E-03	0.9
13	M75-P4	460	77 (415,492)	17	2.005E-01	2.604E-03	0.7
14	M75-P5	597	87 (545,632)	15	1.913E-01	2.199E-03	1.0
15	M75-P6	741	92 (692,784)	12	1.740E-01	1.891E-03	1.0
16	M75-P7	879	100 (830,930)	11	1.517E-01	1.517E-03	0.9
17	HIGH-P0	1016	414 (926,1340)	41	1.440E+00	3.478E-03	6.1
18	M75-P8	1042	94 (985,1079)	9	1.380E-01	1.468E-03	1.3
19	HIGH-P1	1575	449 (1403,1852)	29	3.690E-01	8.218E-04	2.7
20	HIGH-P1	1728	320 (1575,1895)	19	4.020E-01	1.256E-03	2.0
21	HIGH-P2	2280	481 (2078,2559)	21	1.010E+00	2.100E-03	2.4
22	HIGH-P2	2680	621 (2387,3008)	23	1.190E+00	1.916E-03	2.5
23	HIGH-P3	3703	702 (3455,4157)	19	1.700E+00	2.422E-03	5.9
24	HIGH-P3	4062	962 (3703,4665)	24	2.280E+00	2.370E-03	4.2

Table 4: Energy/Flux Calibration Factors for the Merged Electron Channels on Probe A. Valid from 31 Mar 2013 00:00:00 to 03 Aug 2013 00:00:00. Main rate LUT IDs (LOW/M75/HIGH): 16386/24579/29699.

CH #	UNIT-PIX	E [keV]	ΔE (E_{lo},E_{hi}) [keV]	$\Delta E/E$ [%]	$G_0\Delta E$ [cm^2 sr keV]	G_0 [cm^2 sr]	$\Delta G_0/G_0$ [%]
0	LOW-P0	X	X	X	X	X	X
1	LOW-P1	20	9 (16,25)	45	3.210E-02	3.567E-03	1.5
2	LOW-P2	32	11 (27,38)	34	3.630E-02	3.300E-03	1.2
3	LOW-P3	54	12 (48,60)	22	3.625E-02	3.021E-03	0.6
4	LOW-P4	80	14 (73,87)	17	3.291E-02	2.420E-03	0.5
5	LOW-P5	108	22 (97,119)	20	4.953E-02	2.282E-03	0.6
6	M75-P0	X	X	X	X	X	X
7	M75-P1	142	41 (120,161)	29	1.630E-01	3.976E-03	2.2
8	LOW-P6	143	23 (132,155)	16	4.430E-02	1.926E-03	0.5
9	LOW-P7	184	25 (172,197)	14	4.220E-02	1.688E-03	1.3
10	LOW-P8	226	29 (213,242)	13	4.064E-02	1.401E-03	0.8
11	M75-P2	235	55 (205,260)	23	1.880E-01	3.418E-03	1.3
12	M75-P3	346	70 (309,379)	20	2.158E-01	3.083E-03	0.9
13	M75-P4	470	89 (420,509)	19	2.381E-01	2.675E-03	0.9
14	M75-P5	597	94 (545,639)	16	2.104E-01	2.238E-03	0.7
15	M75-P6	749	101 (692,793)	13	2.040E-01	2.020E-03	1.1
16	M75-P7	909	123 (840,963)	14	1.970E-01	1.602E-03	1.3
17	HIGH-P0	1016	383 (926,1309)	38	1.620E+00	4.230E-03	6.5
18	M75-P8	1079	135 (1007,1142)	13	1.690E-01	1.252E-03	1.0
19	HIGH-P1	1575	449 (1403,1852)	29	4.180E-01	9.310E-04	2.9
20	HIGH-P1	1728	364 (1575,1939)	21	4.660E-01	1.280E-03	2.2
21	HIGH-P2	2280	481 (2078,2559)	21	9.480E-01	1.971E-03	2.6
22	HIGH-P2	2680	691 (2387,3078)	26	1.210E+00	1.751E-03	2.4
23	HIGH-P3	3790	621 (3536,4157)	16	1.640E+00	2.641E-03	4.3
24	HIGH-P3	4157	985 (3790,4775)	24	2.080E+00	2.112E-03	5.4

Table 5: Energy/Flux Calibration Factors for the Merged Electron Channels on Probe A. Valid from 03 Aug 2013 00:00:00 to 14 Oct 2019 14:27:00. Main rate LUT IDs (LOW/M75/HIGH): 16386/24579/29699.

CH #	UNIT-PIX	E [keV]	ΔE (E_{lo},E_{hi}) [keV]	$\Delta E/E$ [%]	$G_0\Delta E$ [cm^2 sr keV]	G_0 [cm^2 sr]	$\Delta G_0/G_0$ [%]
0	LOW-P0	X	X	X	X	X	X
1	LOW-P1	20	9 (16,25)	45	3.210E-02	3.567E-03	1.5
2	LOW-P2	32	11 (27,38)	34	3.630E-02	3.300E-03	1.2
3	LOW-P3	54	12 (48,60)	22	3.625E-02	3.021E-03	0.6
4	LOW-P4	80	14 (73,87)	17	3.291E-02	2.420E-03	0.5
5	LOW-P5	108	22 (97,119)	20	4.953E-02	2.282E-03	0.6
6	M75-P0	X	X	X	X	X	X
7	M75-P1	142	41 (120,161)	29	1.630E-01	3.976E-03	2.2
8	LOW-P6	143	23 (132,155)	16	4.430E-02	1.926E-03	0.5
9	LOW-P7	184	25 (172,197)	14	4.220E-02	1.688E-03	1.3
10	LOW-P8	226	29 (213,242)	13	4.064E-02	1.401E-03	0.8
11	M75-P2	235	55 (205,260)	23	1.880E-01	3.418E-03	1.3
12	M75-P3	346	70 (309,379)	20	2.158E-01	3.083E-03	0.9
13	M75-P4	470	89 (420,509)	19	2.381E-01	2.675E-03	0.9
14	M75-P5	597	94 (545,639)	16	2.104E-01	2.238E-03	0.7
15	M75-P6	749	101 (692,793)	13	2.040E-01	2.020E-03	1.1
16	M75-P7	909	123 (840,963)	14	1.970E-01	1.602E-03	1.3
17	HIGH-P0	970	374 (905,1279)	39	3.120E+00	8.342E-03	6.1
18	M75-P8	1079	135 (1007,1142)	13	1.690E-01	1.252E-03	1.0
19	HIGH-P1	1504	317 (1371,1688)	21	1.010E+00	3.186E-03	2.7
20	HIGH-P1	1688	391 (1504,1895)	23	1.570E+00	4.015E-03	1.7
21	HIGH-P2	2333	422 (2078,2500)	18	1.950E+00	4.621E-03	2.7
22	HIGH-P2	2619	745 (2333,3078)	28	2.710E+00	3.638E-03	2.8
23	HIGH-P3	3790	621 (3536,4157)	16	1.410E+00	2.271E-03	5.3
24	HIGH-P3	4254	897 (3878,4775)	21	2.570E+00	2.865E-03	5.9

Probe B (LOW/M75/HIGH)

Table 6: Energy/Flux Calibration Factors for the Merged Electron Channels on Probe B. Valid from 19 Sep 2012 00:00:00 to 28 Sep 2012 16:00:00. Main rate LUT IDs (LOW/M75/HIGH): 17408/25600/31744.

CH #	UNIT-PIX	E [keV]	ΔE (E_{lo},E_{hi}) [keV]	$\Delta E/E$ [%]	$G_0\Delta E$ [cm^2 sr keV]	G_0 [cm^2 sr]	$\Delta G_0/G_0$ [%]
0	LOW-P0	X	X	X	X	X	X
1	LOW-P1	28	5 (25,30)	17	1.617E-02	3.300E-03	0.9
2	LOW-P2	39	7 (35,42)	18	2.268E-02	3.240E-03	0.6
3	LOW-P3	60	9 (55,65)	16	2.555E-02	2.689E-03	0.7
4	LOW-P4	78	11 (72,83)	15	2.856E-02	2.505E-03	0.4
5	LOW-P5	100	15 (92,107)	15	3.296E-02	2.183E-03	0.3
6	LOW-P6	129	16 (121,137)	12	3.137E-02	1.961E-03	0.7
7	M75-P0	X	X	X	X	X	X
8	LOW-P7	166	23 (153,176)	14	3.820E-02	1.661E-03	1.2
9	LOW-P8	201	18 (193,211)	9	2.850E-02	1.583E-03	1.0
10	M75-P1	X	X	X	X	X	X
11	M75-P2	X	X	X	X	X	X
12	M75-P3	X	X	X	X	X	X
13	M75-P4	X	X	X	X	X	X
14	M75-P5	X	X	X	X	X	X
15	M75-P6	X	X	X	X	X	X
16	M75-P7	X	X	X	X	X	X
17	M75-P8	X	X	X	X	X	X
18	HIGH-PXX	X	X	X	X	X	X
19	HIGH-PXX	X	X	X	X	X	X
20	HIGH-PXX	X	X	X	X	X	X
21	HIGH-PXX	X	X	X	X	X	X
22	HIGH-PXX	X	X	X	X	X	X
23	HIGH-PXX	X	X	X	X	X	X
24	HIGH-PXX	X	X	X	X	X	X

Table 7: Energy/Flux Calibration Factors for the Merged Electron Channels on Probe B. Valid from 28 Sep 2012 16:00:00 to 04 Oct 2012 16:30:00. Main rate LUT IDs (LOW/M75/HIGH): 17408/25601/31745.

CH #	UNIT-PIX	E [keV]	ΔE (E_{lo},E_{hi}) [keV]	$\Delta E/E$ [%]	$G_0\Delta E$ [cm^2 sr keV]	G_0 [cm^2 sr]	$\Delta G_0/G_0$ [%]
0	LOW-P0	X	X	X	X	X	X
1	LOW-P1	28	5 (25,30)	17	1.617E-02	3.300E-03	0.9
2	LOW-P2	39	7 (35,42)	18	2.268E-02	3.240E-03	0.6
3	LOW-P3	60	9 (55,65)	16	2.555E-02	2.689E-03	0.7
4	LOW-P4	78	11 (72,83)	15	2.856E-02	2.505E-03	0.4
5	LOW-P5	100	15 (92,107)	15	3.296E-02	2.183E-03	0.3
6	LOW-P6	129	16 (121,137)	12	3.137E-02	1.961E-03	0.7
7	M75-P0	X	X	X	X	X	X
8	LOW-P7	166	23 (153,176)	14	3.820E-02	1.661E-03	1.2
9	M75-P1	169	55 (141,196)	33	2.040E-01	3.709E-03	2.1
10	LOW-P8	201	18 (193,211)	9	2.850E-02	1.583E-03	1.0
11	M75-P2	249	47 (222,269)	19	1.670E-01	3.553E-03	1.3
12	M75-P3	350	62 (312,374)	18	1.870E-01	3.016E-03	1.0
13	M75-P4	465	68 (424,492)	15	1.683E-01	2.475E-03	0.7
14	M75-P5	584	73 (545,618)	13	1.542E-01	2.112E-03	1.0
15	M75-P6	733	75 (692,767)	10	1.531E-01	2.041E-03	0.8
16	M75-P7	869	88 (821,909)	10	1.209E-01	1.374E-03	0.9
17	M75-P8	1031	92 (974,1066)	9	1.160E-01	1.261E-03	1.4
18	HIGH-PXX	X	X	X	X	X	X
19	HIGH-PXX	X	X	X	X	X	X
20	HIGH-PXX	X	X	X	X	X	X
21	HIGH-PXX	X	X	X	X	X	X
22	HIGH-PXX	X	X	X	X	X	X
23	HIGH-PXX	X	X	X	X	X	X
24	HIGH-PXX	X	X	X	X	X	X

Table 8: Energy/Flux Calibration Factors for the Merged Electron Channels on Probe B. Valid from 04 Oct 2012 16:30:00 to 17 Oct 2012 00:00:00. Main rate LUT IDs (LOW/M75/HIGH): 17408/25601/31746.

CH #	UNIT-PIX	E [keV]	ΔE (E_{lo},E_{hi}) [keV]	$\Delta E/E$ [%]	$G_0\Delta E$ [cm^2 sr keV]	G_0 [cm^2 sr]	$\Delta G_0/G_0$ [%]
0	LOW-P0	X	X	X	X	X	X
1	LOW-P1	28	5 (25,30)	17	1.617E-02	3.300E-03	0.9
2	LOW-P2	39	7 (35,42)	18	2.268E-02	3.240E-03	0.6
3	LOW-P3	60	9 (55,65)	16	2.555E-02	2.689E-03	0.7
4	LOW-P4	78	11 (72,83)	15	2.856E-02	2.505E-03	0.4
5	LOW-P5	100	15 (92,107)	15	3.296E-02	2.183E-03	0.3
6	LOW-P6	129	16 (121,137)	12	3.137E-02	1.961E-03	0.7
7	M75-P0	X	X	X	X	X	X
8	LOW-P7	166	23 (153,176)	14	3.820E-02	1.661E-03	1.2
9	M75-P1	169	55 (141,196)	33	2.040E-01	3.709E-03	2.1
10	LOW-P8	201	18 (193,211)	9	2.850E-02	1.583E-03	1.0
11	M75-P2	249	47 (222,269)	19	1.670E-01	3.553E-03	1.3
12	M75-P3	350	62 (312,374)	18	1.870E-01	3.016E-03	1.0
13	M75-P4	465	68 (424,492)	15	1.683E-01	2.475E-03	0.7
14	M75-P5	584	73 (545,618)	13	1.542E-01	2.112E-03	1.0
15	M75-P6	733	75 (692,767)	10	1.531E-01	2.041E-03	0.8
16	M75-P7	869	88 (821,909)	10	1.209E-01	1.374E-03	0.9
17	HIGH-P0	1016	435 (905,1340)	43	3.890E+00	8.943E-03	5.3
18	M75-P8	1031	92 (974,1066)	9	1.160E-01	1.261E-03	1.4
19	HIGH-P1	1575	252 (1436,1688)	16	2.130E+00	8.452E-03	1.8
20	HIGH-P1	1768	327 (1612,1939)	18	1.870E+00	5.719E-03	2.0
21	HIGH-P2	2443	565 (2177,2742)	23	1.000E+00	1.770E-03	2.6
22	HIGH-P2	2806	650 (2500,3150)	23	1.040E+00	1.600E-03	2.6
23	HIGH-P3	3703	702 (3455,4157)	19	7.830E-01	1.115E-03	6.5
24	HIGH-P3	4062	769 (3790,4559)	19	9.740E-01	1.267E-03	5.3

Table 9: Energy/Flux Calibration Factors for the Merged Electron Channels on Probe B. Valid from 17 Oct 2012 00:00:00 to 24 Oct 2012 00:00:00. Main rate LUT IDs (LOW/M75/HIGH): 18433/25601/31746.

CH #	UNIT-PIX	E [keV]	ΔE (E_{lo},E_{hi}) [keV]	$\Delta E/E$ [%]	$G_0 \Delta E$ [cm^2 sr keV]	G_0 [cm^2 sr]	$\Delta G_0/G_0$ [%]
0	LOW-P0	X	X	X	X	X	X
1	LOW-P1	23	7 (20,27)	30	2.688E-02	3.840E-03	1.0
2	LOW-P2	37	9 (32,41)	24	2.980E-02	3.311E-03	0.7
3	LOW-P3	55	9 (50,60)	17	2.647E-02	2.816E-03	0.4
4	LOW-P4	77	11 (71,82)	15	2.864E-02	2.535E-03	0.5
5	LOW-P5	103	14 (96,110)	13	3.232E-02	2.342E-03	0.5
6	M75-P0	X	X	X	X	X	X
7	LOW-P6	134	19 (124,143)	14	3.970E-02	2.089E-03	0.7
8	LOW-P7	168	27 (155,182)	16	4.196E-02	1.554E-03	0.6
9	M75-P1	169	55 (141,196)	33	2.040E-01	3.709E-03	2.1
10	LOW-P8	199	21 (190,211)	11	2.640E-02	1.257E-03	1.4
11	M75-P2	249	47 (222,269)	19	1.670E-01	3.553E-03	1.3
12	M75-P3	350	62 (312,374)	18	1.870E-01	3.016E-03	1.0
13	M75-P4	465	68 (424,492)	15	1.683E-01	2.475E-03	0.7
14	M75-P5	584	73 (545,618)	13	1.542E-01	2.112E-03	1.0
15	M75-P6	733	75 (692,767)	10	1.531E-01	2.041E-03	0.8
16	M75-P7	869	88 (821,909)	10	1.209E-01	1.374E-03	0.9
17	HIGH-P0	1016	435 (905,1340)	43	3.890E+00	8.943E-03	5.3
18	M75-P8	1031	92 (974,1066)	9	1.160E-01	1.261E-03	1.4
19	HIGH-P1	1575	252 (1436,1688)	16	2.130E+00	8.452E-03	1.8
20	HIGH-P1	1768	327 (1612,1939)	18	1.870E+00	5.719E-03	2.0
21	HIGH-P2	2443	565 (2177,2742)	23	1.000E+00	1.770E-03	2.6
22	HIGH-P2	2806	650 (2500,3150)	23	1.040E+00	1.600E-03	2.6
23	HIGH-P3	3703	702 (3455,4157)	19	7.830E-01	1.115E-03	6.5
24	HIGH-P3	4062	769 (3790,4559)	19	9.740E-01	1.267E-03	5.3

Table 10: Energy/Flux Calibration Factors for the Merged Electron Channels on Probe B. Valid from 24 Oct 2012 00:00:00 to 24 Feb 2013 00:00:00. Main rate LUT IDs (LOW/M75/HIGH): 18433/26626/31746.

CH #	UNIT-PIX	E [keV]	ΔE (E_{lo},E_{hi}) [keV]	$\Delta E/E$ [%]	$G_0\Delta E$ [cm^2 sr keV]	G_0 [cm^2 sr]	$\Delta G_0/G_0$ [%]
0	LOW-P0	X	X	X	X	X	X
1	LOW-P1	23	7 (20,27)	30	2.688E-02	3.840E-03	1.0
2	LOW-P2	37	9 (32,41)	24	2.980E-02	3.311E-03	0.7
3	LOW-P3	55	9 (50,60)	17	2.647E-02	2.816E-03	0.4
4	LOW-P4	77	11 (71,82)	15	2.864E-02	2.535E-03	0.5
5	LOW-P5	103	14 (96,110)	13	3.232E-02	2.342E-03	0.5
6	LOW-P6	134	19 (124,143)	14	3.970E-02	2.089E-03	0.7
7	LOW-P7	168	27 (155,182)	16	4.196E-02	1.554E-03	0.6
8	LOW-P8	199	21 (190,211)	11	2.640E-02	1.257E-03	1.4
9	M75-P2	217	26 (198,224)	12	9.250E-02	3.558E-03	1.8
10	M75-P2	249	47 (222,269)	19	1.670E-01	3.553E-03	1.3
11	M75-P3	334	30 (312,342)	9	8.549E-02	2.850E-03	0.8
12	M75-P3	362	36 (338,374)	10	1.003E-01	2.786E-03	0.8
13	M75-P4	465	68 (424,492)	15	1.683E-01	2.475E-03	0.7
14	M75-P5	584	73 (545,618)	13	1.542E-01	2.112E-03	1.0
15	M75-P6	733	75 (692,767)	10	1.531E-01	2.041E-03	0.8
16	M75-P7	869	88 (821,909)	10	1.209E-01	1.374E-03	0.9
17	HIGH-P0	1016	435 (905,1340)	43	3.890E+00	8.943E-03	5.3
18	M75-P8	1031	92 (974,1066)	9	1.160E-01	1.261E-03	1.4
19	HIGH-P1	1575	252 (1436,1688)	16	2.130E+00	8.452E-03	1.8
20	HIGH-P1	1768	327 (1612,1939)	18	1.870E+00	5.719E-03	2.0
21	HIGH-P2	2443	565 (2177,2742)	23	1.000E+00	1.770E-03	2.6
22	HIGH-P2	2806	650 (2500,3150)	23	1.040E+00	1.600E-03	2.6
23	HIGH-P3	3703	702 (3455,4157)	19	7.830E-01	1.115E-03	6.5
24	HIGH-P3	4062	769 (3790,4559)	19	9.740E-01	1.267E-03	5.3

Table 11: Energy/Flux Calibration Factors for the Merged Electron Channels on Probe B. Valid from 24 Feb 2013 00:00:00 to 31 Mar 2013 00:00:00. Main rate LUT IDs (LOW/M75/HIGH): 18433/25601/31746.

CH #	UNIT-PIX	E [keV]	ΔE (E_{lo},E_{hi}) [keV]	$\Delta E/E$ [%]	$G_0\Delta E$ [cm^2 sr keV]	G_0 [cm^2 sr]	$\Delta G_0/G_0$ [%]
0	LOW-P0	X	X	X	X	X	X
1	LOW-P1	23	7 (20,27)	30	2.688E-02	3.840E-03	1.0
2	LOW-P2	37	9 (32,41)	24	2.980E-02	3.311E-03	0.7
3	LOW-P3	55	9 (50,60)	17	2.647E-02	2.816E-03	0.4
4	LOW-P4	77	11 (71,82)	15	2.864E-02	2.535E-03	0.5
5	LOW-P5	103	14 (96,110)	13	3.232E-02	2.342E-03	0.5
6	M75-P0	X	X	X	X	X	X
7	LOW-P6	134	19 (124,143)	14	3.970E-02	2.089E-03	0.7
8	LOW-P7	168	27 (155,182)	16	4.196E-02	1.554E-03	0.6
9	M75-P1	169	55 (141,196)	33	2.040E-01	3.709E-03	2.1
10	LOW-P8	199	21 (190,211)	11	2.640E-02	1.257E-03	1.4
11	M75-P2	249	47 (222,269)	19	1.670E-01	3.553E-03	1.3
12	M75-P3	350	62 (312,374)	18	1.870E-01	3.016E-03	1.0
13	M75-P4	465	68 (424,492)	15	1.683E-01	2.475E-03	0.7
14	M75-P5	584	73 (545,618)	13	1.542E-01	2.112E-03	1.0
15	M75-P6	733	75 (692,767)	10	1.531E-01	2.041E-03	0.8
16	M75-P7	869	88 (821,909)	10	1.209E-01	1.374E-03	0.9
17	HIGH-P0	1016	435 (905,1340)	43	3.890E+00	8.943E-03	5.3
18	M75-P8	1031	92 (974,1066)	9	1.160E-01	1.261E-03	1.4
19	HIGH-P1	1575	252 (1436,1688)	16	2.130E+00	8.452E-03	1.8
20	HIGH-P1	1768	327 (1612,1939)	18	1.870E+00	5.719E-03	2.0
21	HIGH-P2	2443	565 (2177,2742)	23	1.000E+00	1.770E-03	2.6
22	HIGH-P2	2806	650 (2500,3150)	23	1.040E+00	1.600E-03	2.6
23	HIGH-P3	3703	702 (3455,4157)	19	7.830E-01	1.115E-03	6.5
24	HIGH-P3	4062	769 (3790,4559)	19	9.740E-01	1.267E-03	5.3

Table 12: Energy/Flux Calibration Factors for the Merged Electron Channels on Probe B. Valid from 31 Mar 2013 00:00:00 to 03 Aug 2013 00:00:00. Main rate LUT IDs (LOW/M75/HIGH): 18434/26627/31747.

CH #	UNIT-PIX	E [keV]	ΔE (E_{lo},E_{hi}) [keV]	$\Delta E/E$ [%]	$G_0 \Delta E$ [cm^2 sr keV]	G_0 [cm^2 sr]	$\Delta G_0/G_0$ [%]
0	LOW-P0	X	X	X	X	X	X
1	LOW-P1	24	7 (21,28)	29	2.570E-02	3.671E-03	1.0
2	LOW-P2	33	10 (28,38)	30	3.620E-02	3.620E-03	1.2
3	LOW-P3	54	14 (48,62)	26	4.022E-02	2.873E-03	0.8
4	LOW-P4	75	14 (68,82)	18	3.298E-02	2.425E-03	0.8
5	LOW-P5	102	15 (94,109)	15	3.511E-02	2.341E-03	0.4
6	M75-P0	X	X	X	X	X	X
7	LOW-P6	132	21 (122,143)	16	3.936E-02	1.874E-03	0.4
8	M75-P1	154	50 (127,177)	32	1.980E-01	3.960E-03	2.2
9	LOW-P7	168	25 (157,182)	15	4.140E-02	1.656E-03	1.2
10	LOW-P8	208	31 (195,226)	15	4.442E-02	1.433E-03	0.9
11	M75-P2	246	49 (217,266)	20	1.680E-01	3.429E-03	1.2
12	M75-P3	354	75 (312,387)	21	2.274E-01	3.032E-03	0.9
13	M75-P4	470	85 (424,509)	18	2.164E-01	2.546E-03	0.9
14	M75-P5	604	81 (558,639)	13	1.834E-01	2.264E-03	0.8
15	M75-P6	749	101 (692,793)	13	1.923E-01	1.904E-03	0.9
16	M75-P7	899	112 (840,952)	12	1.793E-01	1.601E-03	0.8
17	HIGH-P0	992	374 (905,1279)	38	3.570E+00	9.545E-03	5.2
18	M75-P8	1054	120 (996,1116)	11	1.646E-01	1.372E-03	0.7
19	HIGH-P1	1504	241 (1371,1612)	16	1.360E+00	5.643E-03	3.3
20	HIGH-P1	1688	400 (1539,1939)	24	2.980E+00	7.450E-03	1.6
21	HIGH-P2	2387	442 (2177,2619)	19	7.390E-01	1.672E-03	2.5
22	HIGH-P2	2680	763 (2387,3150)	28	1.230E+00	1.612E-03	2.4
23	HIGH-P3	3703	702 (3455,4157)	19	6.920E-01	9.858E-04	6.5
24	HIGH-P3	4062	985 (3790,4775)	24	1.180E+00	1.198E-03	5.6

Table 13: Energy/Flux Calibration Factors for the Merged Electron Channels on Probe B. Valid from 03 Aug 2013 00:00:00 to 16 Jul 2019 16:58:00. Main rate LUT IDs (LOW/M75/HIGH): 18434/26627/31747.

CH #	UNIT-PIX	E [keV]	ΔE (E_{lo},E_{hi}) [keV]	$\Delta E/E$ [%]	$G_0\Delta E$ [cm^2 sr keV]	G_0 [cm^2 sr]	$\Delta G_0/G_0$ [%]
0	LOW-P0	X	X	X	X	X	X
1	LOW-P1	24	7 (21,28)	29	2.570E-02	3.671E-03	1.0
2	LOW-P2	33	10 (28,38)	30	3.620E-02	3.620E-03	1.2
3	LOW-P3	54	14 (48,62)	26	4.022E-02	2.873E-03	0.8
4	LOW-P4	75	14 (68,82)	18	3.298E-02	2.425E-03	0.8
5	LOW-P5	102	15 (94,109)	15	3.511E-02	2.341E-03	0.4
6	M75-P0	X	X	X	X	X	X
7	LOW-P6	132	21 (122,143)	16	3.936E-02	1.874E-03	0.4
8	M75-P1	154	50 (127,177)	32	1.980E-01	3.960E-03	2.2
9	LOW-P7	168	25 (157,182)	15	4.140E-02	1.656E-03	1.2
10	LOW-P8	208	31 (195,226)	15	4.442E-02	1.433E-03	0.9
11	M75-P2	246	49 (217,266)	20	1.680E-01	3.429E-03	1.2
12	M75-P3	354	75 (312,387)	21	2.274E-01	3.032E-03	0.9
13	M75-P4	470	85 (424,509)	18	2.164E-01	2.546E-03	0.9
14	M75-P5	604	81 (558,639)	13	1.834E-01	2.264E-03	0.8
15	M75-P6	749	101 (692,793)	13	1.923E-01	1.904E-03	0.9
16	M75-P7	899	112 (840,952)	12	1.793E-01	1.601E-03	0.8
17	HIGH-P0	992	374 (905,1279)	38	3.590E+00	9.599E-03	5.2
18	M75-P8	1054	120 (996,1116)	11	1.646E-01	1.372E-03	0.7
19	HIGH-P1	1504	241 (1371,1612)	16	1.360E+00	5.643E-03	3.3
20	HIGH-P1	1688	400 (1539,1939)	24	2.980E+00	7.450E-03	1.6
21	HIGH-P2	2280	365 (2078,2443)	16	2.140E+00	5.863E-03	1.9
22	HIGH-P2	2619	745 (2333,3078)	28	4.710E+00	6.322E-03	2.1
23	HIGH-P3	3618	502 (3376,3878)	14	2.730E+00	5.438E-03	3.8
24	HIGH-P3	3969	941 (3618,4559)	24	4.910E+00	5.218E-03	4.1

Probe A (M35)

Table 14: Energy/Flux Calibration Factors for the Merged Electron Channels on Probe A. Valid from 25 Sep 2012 05:30:00 to 03 Apr 2013 00:00:00. Main rate LUT IDs (M35): 20482.

CH #	UNIT-PIX	E [keV]	ΔE (E_{lo},E_{hi}) [keV]	$\Delta E/E$ [%]	$G_0\Delta E$ [cm^2 sr keV]	G_0 [cm^2 sr]	$\Delta G_0/G_0$ [%]
0	M35-P0	X	X	X	X	X	X
1	M35-P1	156	40 (135,175)	26	1.540E-01	3.850E-03	1.8
2	M35-P2	243	52 (214,266)	21	1.760E-01	3.385E-03	1.2
3	M35-P3	338	57 (305,362)	17	1.659E-01	2.911E-03	0.8
4	M35-P4	449	65 (410,475)	14	1.785E-01	2.746E-03	0.7
5	M35-P5	558	69 (515,584)	12	1.538E-01	2.229E-03	0.7
6	M35-P6	708	88 (661,749)	12	1.636E-01	1.859E-03	0.6
7	M35-P7	859	87 (802,889)	10	1.390E-01	1.598E-03	1.1
8	M35-P8	1019	92 (974,1066)	9	1.260E-01	1.370E-03	1.0

Table 15: Energy/Flux Calibration Factors for the Merged Electron Channels on Probe A. Valid from 03 Apr 2013 00:00:00 to 14 Oct 2019 14:27:00. Main rate LUT IDs (M35): 20483.

CH #	UNIT-PIX	E [keV]	ΔE (E_{lo},E_{hi}) [keV]	$\Delta E/E$ [%]	$G_0\Delta E$ [cm^2 sr keV]	G_0 [cm^2 sr]	$\Delta G_0/G_0$ [%]
0	M35-P0	X	X	X	X	X	X
1	M35-P1	138	40 (116,156)	29	1.490E-01	3.725E-03	2.1
2	M35-P2	224	60 (191,251)	27	2.030E-01	3.383E-03	1.4
3	M35-P3	330	63 (295,358)	19	1.930E-01	3.063E-03	1.1
4	M35-P4	444	80 (401,481)	18	2.097E-01	2.621E-03	0.8
5	M35-P5	584	73 (545,618)	13	1.637E-01	2.242E-03	0.5
6	M35-P6	724	106 (661,767)	15	2.050E-01	1.934E-03	1.1
7	M35-P7	879	109 (821,930)	12	1.669E-01	1.531E-03	0.7
8	M35-P8	1042	118 (985,1103)	11	1.440E-01	1.220E-03	1.1

Probe B (M35)

Table 16: Energy/Flux Calibration Factors for the Merged Electron Channels on Probe B. Valid from 19 Sep 2012 00:00:00 to 03 Apr 2013 00:00:00. Main rate LUT IDs (M35): 38912.

CH #	UNIT-PIX	E [keV]	ΔE (E_{lo},E_{hi}) [keV]	$\Delta E/E$ [%]	$G_0 \Delta E$ [cm^2 sr keV]	G_0 [cm^2 sr]	$\Delta G_0/G_0$ [%]
0	M35-P0	X	X	X	X	X	X
1	M35-P1	167	45 (142,187)	27	1.860E-01	4.133E-03	1.9
2	M35-P2	254	48 (227,275)	19	1.850E-01	3.854E-03	1.2
3	M35-P3	358	60 (323,383)	17	1.950E-01	3.250E-03	1.1
4	M35-P4	475	75 (434,509)	16	2.035E-01	2.713E-03	0.7
5	M35-P5	604	81 (558,639)	13	1.828E-01	2.257E-03	0.6
6	M35-P6	749	84 (700,784)	11	1.540E-01	1.833E-03	1.0
7	M35-P7	889	90 (840,930)	10	1.525E-01	1.694E-03	0.7
8	M35-P8	1054	107 (996,1103)	10	1.440E-01	1.346E-03	1.0

Table 17: Energy/Flux Calibration Factors for the Merged Electron Channels on Probe B. Valid from 03 Apr 2013 00:00:00 to 16 Jul 2019 16:58:00. Main rate LUT IDs (M35): 38913.

CH #	UNIT-PIX	E [keV]	ΔE (E_{lo},E_{hi}) [keV]	$\Delta E/E$ [%]	$G_0 \Delta E$ [cm^2 sr keV]	G_0 [cm^2 sr]	$\Delta G_0/G_0$ [%]
0	M35-P0	X	X	X	X	X	X
1	M35-P1	158	44 (133,177)	28	1.870E-01	4.250E-03	2.1
2	M35-P2	251	53 (222,275)	21	2.030E-01	3.830E-03	1.3
3	M35-P3	358	68 (319,387)	19	2.122E-01	3.121E-03	1.0
4	M35-P4	475	75 (434,509)	16	2.035E-01	2.713E-03	0.7
5	M35-P5	597	74 (558,632)	12	1.580E-01	2.135E-03	1.0
6	M35-P6	741	92 (692,784)	12	1.729E-01	1.879E-03	0.6
7	M35-P7	879	100 (830,930)	11	1.650E-01	1.650E-03	1.1
8	M35-P8	1054	120 (996,1116)	11	1.495E-01	1.246E-03	0.8
