# Supplementary material for: The Magnetic Electron Ion Spectrometer: A Review of On-Orbit Sensor Performance, Data, Operations, and Science
Source: Space Sci Rev. 2021 Oct 28;217(8):80. doi: 10.1007/s11214-021-00855-2 (PMC8553741; doi:10.1007/s11214-021-00855-2)
Supplement: Supplementary file 5 — Tables of the calibration (energy channel definitions/flux conversion) factors for all of the LUTs used on orbit for the ion main rates (PDF 72 kB) [file 11214_2021_855_MOESM5_ESM.pdf]

MagEIS Calibration Factors: Ion Main Rates

June 18, 2021

Table 1 through Table 5 provide energy channel definitions and flux conversion factors for the main rate proton channels on both Probes. Note the valid time ranges indicated in the table captions.

Table 1: Energy/Flux Calibration Factors for the Proton Channels on Probe A. Valid from 06 Sep 2012 00:00:00 to 23 Dec 2012 00:00:00. Main rate LUTID: 29952. Note that channel 20 should be considered invalid (it has PHA 255 junk bin included).

Chan #	E [keV]	ΔE (E_{lo},E_{hi}) [keV]	$\Delta E/E$ [%]	G [cm^2 sr]	ϵ
0	64	5 (61,67)	8	0.00236	1.0
1	75	5 (72,77)	7	0.00236	1.0
2	88	11 (83,94)	12	0.00236	1.0
3	105	11 (99,110)	10	0.00236	1.0
4	121	11 (115,126)	9	0.00236	1.0
5	140	16 (132,148)	12	0.00236	1.0
6	164	22 (153,175)	13	0.00236	1.0
7	191	22 (180,202)	11	0.00236	1.0
8	224	33 (208,240)	15	0.00236	1.0
9	256	22 (246,267)	8	0.00236	1.0
10	294	43 (273,316)	15	0.00236	1.0
11	346	49 (322,370)	14	0.00236	1.0
12	403	54 (376,430)	13	0.00236	1.0
13	468	65 (435,501)	14	0.00236	1.0
14	544	76 (506,582)	14	0.00236	1.0
15	631	87 (587,674)	14	0.00236	1.0
16	734	108 (680,788)	15	0.00236	1.0
17	853	119 (794,913)	14	0.00236	1.0
18	994	152 (918,1070)	15	0.00236	1.0
19	1154	157 (1076,1233)	14	0.00236	1.0
20	1295	114 (1238,1352)	9	0.00236	1.0

Table 2: Energy/Flux Calibration Factors for the Proton Channels on Probe A. Valid from 23 Dec 2012 00:00:00 to 31 Mar 2013 00:00:00. Main rate LUTID: 29953.

Chan #	E [keV]	ΔE (E_{lo},E_{hi}) [keV]	$\Delta E/E$ [%]	G [cm^2 sr]	ϵ
0	64	5 (61,67)	8	0.00236	1.0
1	75	5 (72,77)	7	0.00236	1.0
2	88	11 (83,94)	12	0.00236	1.0
3	105	11 (99,110)	10	0.00236	1.0
4	121	11 (115,126)	9	0.00236	1.0
5	140	16 (132,148)	12	0.00236	1.0
6	164	22 (153,175)	13	0.00236	1.0
7	191	22 (180,202)	11	0.00236	1.0
8	224	33 (208,240)	15	0.00236	1.0
9	256	22 (246,267)	8	0.00236	1.0
10	294	43 (273,316)	15	0.00236	1.0
11	346	49 (322,370)	14	0.00236	1.0
12	403	54 (376,430)	13	0.00236	1.0
13	468	65 (435,501)	14	0.00236	1.0
14	544	76 (506,582)	14	0.00236	1.0
15	631	87 (587,674)	14	0.00236	1.0
16	734	108 (680,788)	15	0.00236	1.0
17	853	119 (794,913)	14	0.00236	1.0
18	994	152 (918,1070)	15	0.00236	1.0
19	1154	157 (1076,1233)	14	0.00236	1.0
20	1295	114 (1238,1352)	9	0.00236	1.0

Table 3: Energy/Flux Calibration Factors for the Proton Channels on Probe A. Valid from 31 Mar 2013 00:00:00 to 14 Oct 2019 14:27:00. Main rate LUTID: 29954.

Chan #	E [keV]	ΔE (E_{lo},E_{hi}) [keV]	$\Delta E/E$ [%]	G [cm^2 sr]	ϵ
0	58	5 (56,61)	9	0.00236	1.0
1	69	5 (67,72)	8	0.00236	1.0
2	83	11 (77,88)	13	0.00236	1.0
3	99	11 (94,105)	11	0.00236	1.0
4	118	16 (110,126)	14	0.00236	1.0
5	140	16 (132,148)	12	0.00236	1.0
6	164	22 (153,175)	13	0.00236	1.0
7	194	27 (180,208)	14	0.00236	1.0
8	229	33 (213,246)	14	0.00236	1.0
9	267	33 (251,284)	12	0.00236	1.0
10	308	38 (289,327)	12	0.00236	1.0
11	357	49 (332,381)	14	0.00236	1.0
12	414	54 (387,441)	13	0.00236	1.0
13	479	65 (446,511)	14	0.00236	1.0
14	555	76 (517,593)	14	0.00236	1.0
15	636	76 (598,674)	12	0.00236	1.0
16	728	98 (680,777)	13	0.00236	1.0
17	840	114 (783,897)	14	0.00236	1.0
18	967	130 (902,1032)	13	0.00236	1.0
19	1111	146 (1038,1184)	13	0.00236	1.0
20	1271	163 (1190,1352)	13	0.00236	1.0

Table 4: Energy/Flux Calibration Factors for the Proton Channels on Probe B. Valid from 06 Sep 2012 00:00:00 to 29 Sep 2012 00:00:00. Main rate LUTID: 32000. Note that channel 20 should be considered invalid (it has PHA 255 junk bin included).

Chan #	E [keV]	ΔE (E_{lo},E_{hi}) [keV]	$\Delta E/E$ [%]	G [cm^2 sr]	ϵ
0	63	5 (60,65)	9	0.00236	1.0
1	74	5 (71,76)	7	0.00236	1.0
2	84	5 (82,87)	6	0.00236	1.0
3	98	11 (93,103)	11	0.00236	1.0
4	114	11 (109,120)	9	0.00236	1.0
5	133	16 (125,141)	12	0.00236	1.0
6	155	16 (147,163)	10	0.00236	1.0
7	179	22 (168,190)	12	0.00236	1.0
8	206	22 (195,217)	11	0.00236	1.0
9	239	33 (223,255)	14	0.00236	1.0
10	277	33 (260,293)	12	0.00236	1.0
11	320	43 (298,342)	14	0.00236	1.0
12	371	49 (347,396)	13	0.00236	1.0
13	431	60 (401,461)	14	0.00236	1.0
14	499	65 (466,531)	13	0.00236	1.0
15	577	81 (537,618)	14	0.00236	1.0
16	669	92 (623,715)	14	0.00236	1.0
17	772	103 (721,824)	13	0.00236	1.0
18	891	125 (829,954)	14	0.00236	1.0
19	1032	146 (959,1105)	14	0.00236	1.0
20	1203	184 (1111,1295)	15	0.00236	1.0

Table 5: Energy/Flux Calibration Factors for the Proton Channels on Probe B. Valid from 29 Sep 2012 00:00:00 to 16 Jul 2019 16:58:00. Main rate LUTID: 32001.

Chan #	E [keV]	ΔE (E_{lo},E_{hi}) [keV]	$\Delta E/E$ [%]	G [cm^2 sr]	ϵ
0	63	5 (60,65)	9	0.00236	1.0
1	74	5 (71,76)	7	0.00236	1.0
2	84	5 (82,87)	6	0.00236	1.0
3	98	11 (93,103)	11	0.00236	1.0
4	114	11 (109,120)	9	0.00236	1.0
5	133	16 (125,141)	12	0.00236	1.0
6	155	16 (147,163)	10	0.00236	1.0
7	179	22 (168,190)	12	0.00236	1.0
8	206	22 (195,217)	11	0.00236	1.0
9	239	32 (223,255)	14	0.00236	1.0
10	277	32 (260,293)	12	0.00236	1.0
11	320	43 (298,342)	14	0.00236	1.0
12	371	49 (347,396)	13	0.00236	1.0
13	431	60 (401,461)	14	0.00236	1.0
14	499	65 (466,531)	13	0.00236	1.0
15	577	81 (537,618)	14	0.00236	1.0
16	669	92 (623,715)	14	0.00236	1.0
17	772	103 (721,824)	13	0.00236	1.0
18	891	125 (829,954)	14	0.00236	1.0
19	1032	146 (959,1105)	14	0.00236	1.0
20	1206	190 (1111,1300)	16	0.00236	1.0
