# Supplementary material for: The Magnetic Electron Ion Spectrometer: A Review of On-Orbit Sensor Performance, Data, Operations, and Science
Source: Space Sci Rev. 2021 Oct 28;217(8):80. doi: 10.1007/s11214-021-00855-2 (PMC8553741; doi:10.1007/s11214-021-00855-2)
Supplement: Supplementary file 6 — Figures indicating the instrument mode (e.g., science, high-rate, or maintenance mode) for all 8 units over the course of the mission (PDF 3.0 MB) [file 11214_2021_855_MOESM6_ESM.pdf]

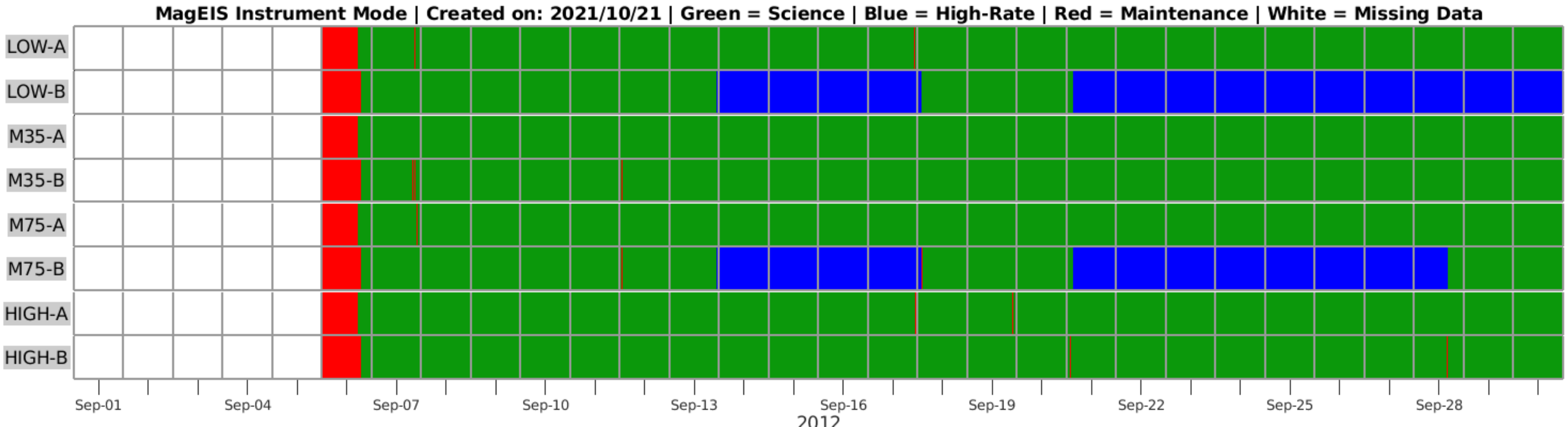
MagEIS Instrument Mode | Created on: 2021/10/21 | Green = Science | Blue = High-Rate | Red = Maintenance | White = Missing Data
LOW-A
LOW-B
M35-A
M35-B
M75-A
M75-B
HIGH-A
HIGH-B
Sep-01
Sep-04
Sep-07
Sep-10
Sep-13
Sep-16
Sep-19
Sep-22
Sep-25
Sep-28
2012

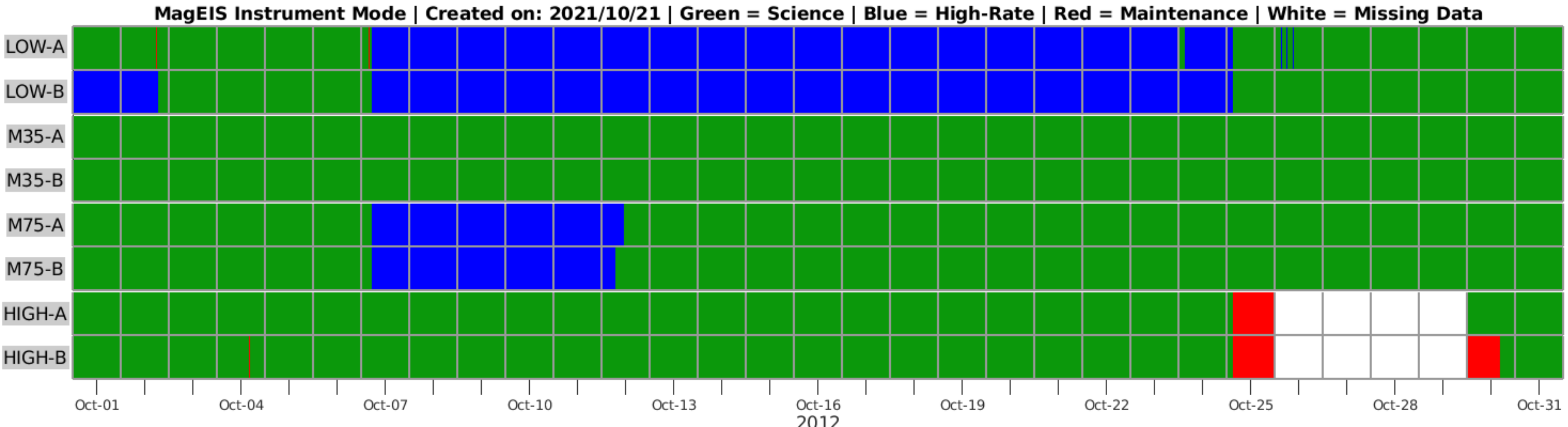

MagEIS Instrument Mode | Created on: 2021/10/21 | Green = Science | Blue = High-Rate | Red = Maintenance | White = Missing Data
LOW-A
LOW-B
M35-A
M35-B
M75-A
M75-B
HIGH-A
HIGH-B
Oct-01
Oct-04
Oct-07
Oct-10
Oct-13
Oct-16
Oct-19
Oct-22
Oct-25
Oct-28
Oct-31
2012

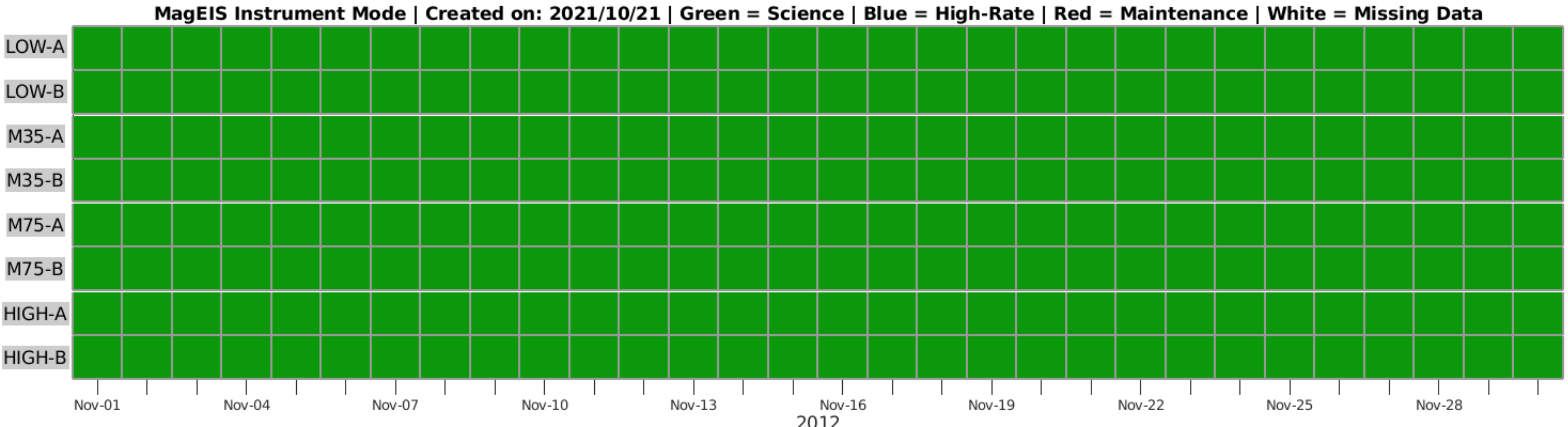
MagEIS Instrument Mode | Created on: 2021/10/21 | Green = Science | Blue = High-Rate | Red = Maintenance | White = Missing Data
LOW-A
LOW-B
M35-A
M35-B
M75-A
M75-B
HIGH-A
HIGH-B
Nov-01
Nov-04
Nov-07
Nov-10
Nov-13
Nov-16
Nov-19
Nov-22
Nov-25
Nov-28
2012

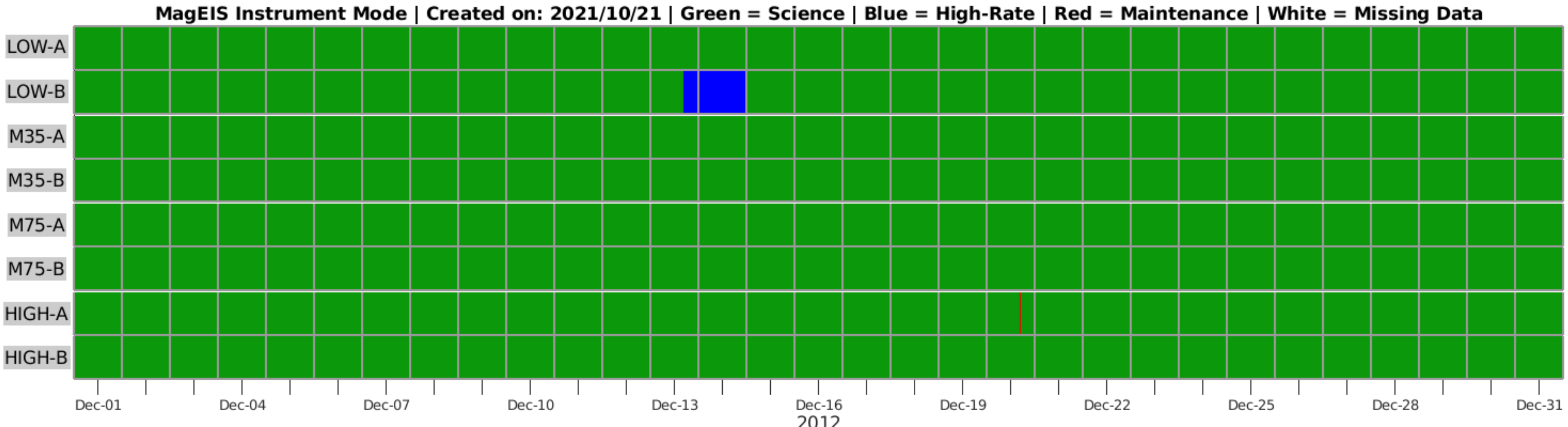
MagEIS Instrument Mode | Created on: 2021/10/21 | Green = Science | Blue = High-Rate | Red = Maintenance | White = Missing Data
LOW-A
LOW-B
M35-A
M35-B
M75-A
M75-B
HIGH-A
HIGH-B
Dec-01
Dec-04
Dec-07
Dec-10
Dec-13
Dec-16
Dec-19
Dec-22
Dec-25
Dec-28
Dec-31
2012

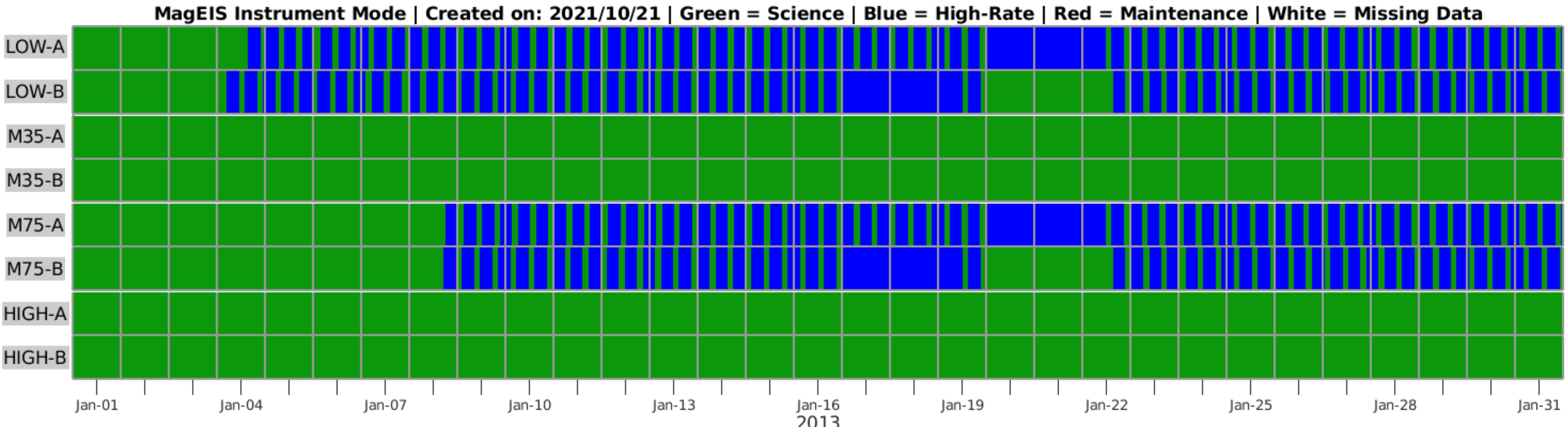

MagEIS Instrument Mode | Created on: 2021/10/21 | Green = Science | Blue = High-Rate | Red = Maintenance | White = Missing Data
LOW-A
LOW-B
M35-A
M35-B
M75-A
M75-B
HIGH-A
HIGH-B
Jan-01
Jan-04
Jan-07
Jan-10
Jan-13
Jan-16
Jan-19
Jan-22
Jan-25
Jan-28
Jan-31
2013

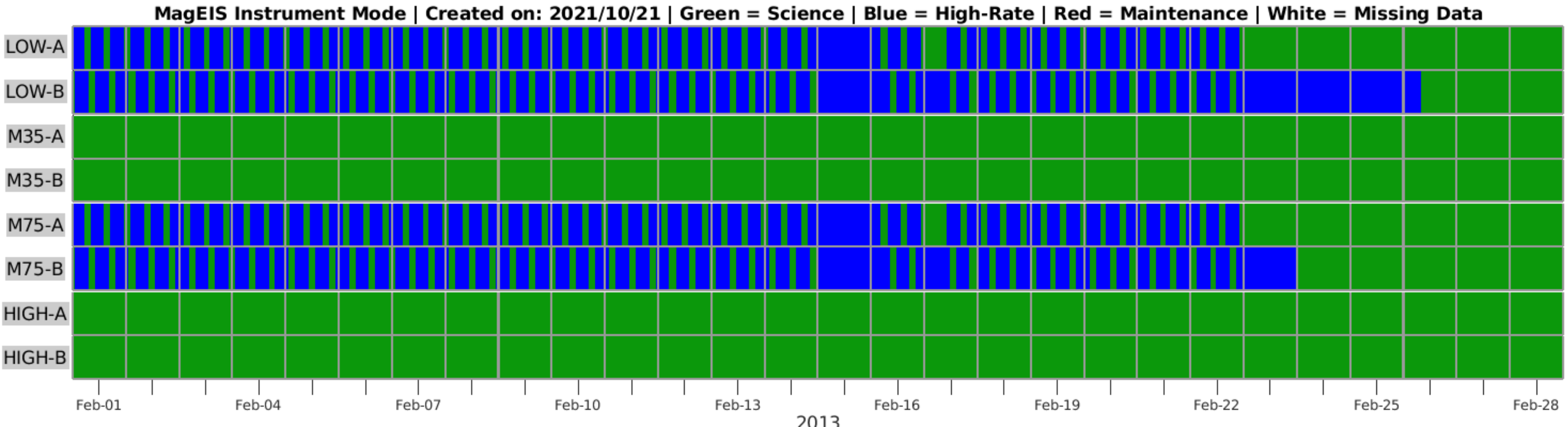

MagEIS Instrument Mode | Created on: 2021/10/21 | Green = Science | Blue = High-Rate | Red = Maintenance | White = Missing Data
LOW-A
LOW-B
M35-A
M35-B
M75-A
M75-B
HIGH-A
HIGH-B
Feb-01
Feb-04
Feb-07
Feb-10
Feb-13
Feb-16
Feb-19
Feb-22
Feb-25
Feb-28
2013

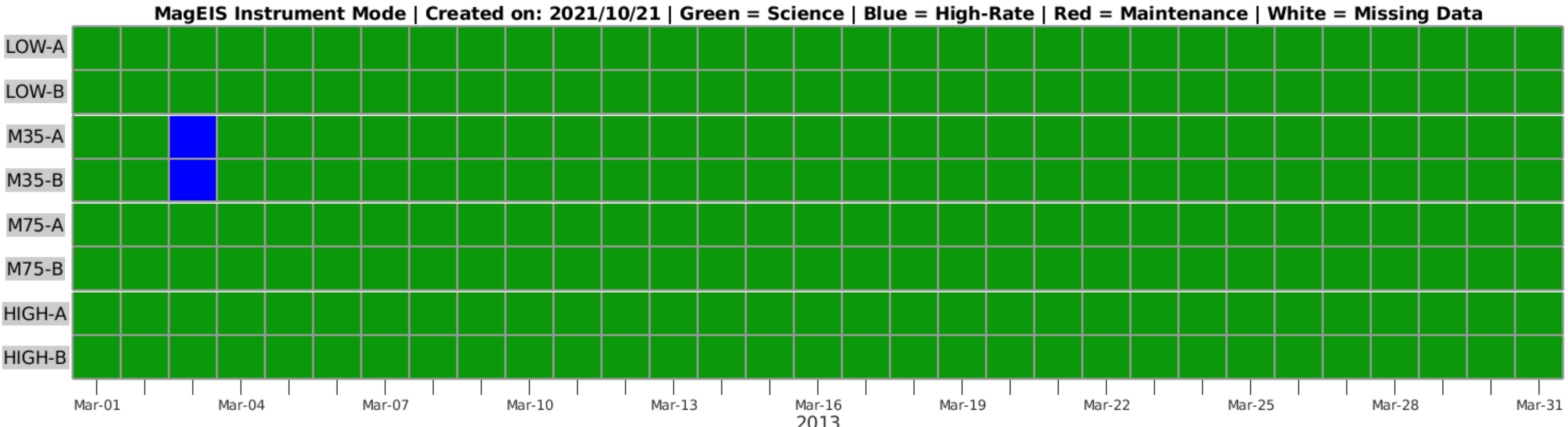

MagEIS Instrument Mode | Created on: 2021/10/21 | Green = Science | Blue = High-Rate | Red = Maintenance | White = Missing Data
LOW-A
LOW-B
M35-A
M35-B
M75-A
M75-B
HIGH-A
HIGH-B
Mar-01
Mar-04
Mar-07
Mar-10
Mar-13
Mar-16
Mar-19
Mar-22
Mar-25
Mar-28
Mar-31
2013

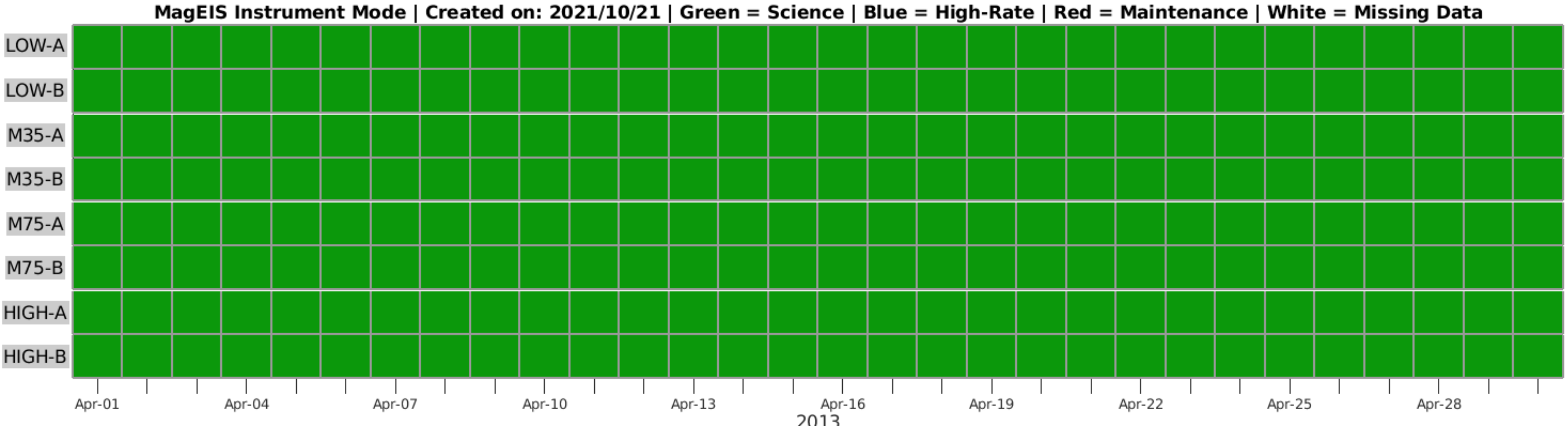

MagEIS Instrument Mode | Created on: 2021/10/21 | Green = Science | Blue = High-Rate | Red = Maintenance | White = Missing Data
LOW-A
LOW-B
M35-A
M35-B
M75-A
M75-B
HIGH-A
HIGH-B
Apr-01
Apr-04
Apr-07
Apr-10
Apr-13
Apr-16
Apr-19
Apr-22
Apr-25
Apr-28
2013

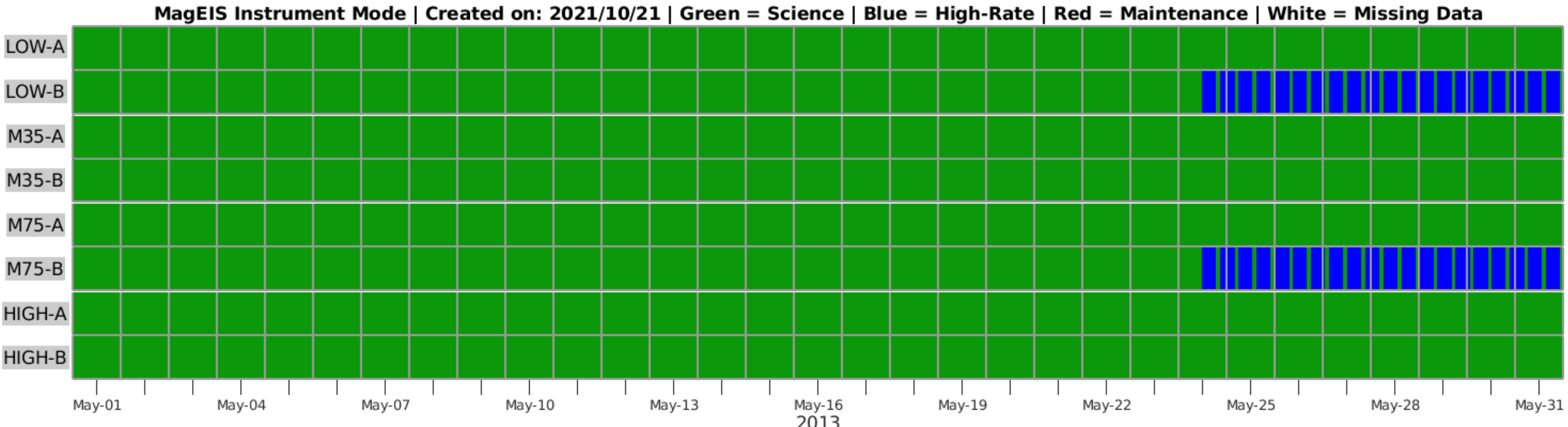

MagEIS Instrument Mode | Created on: 2021/10/21 | Green = Science | Blue = High-Rate | Red = Maintenance | White = Missing Data
LOW-A
LOW-B
M35-A
M35-B
M75-A
M75-B
HIGH-A
HIGH-B
May-01
May-04
May-07
May-10
May-13
May-16
May-19
May-22
May-25
May-28
May-31
2013

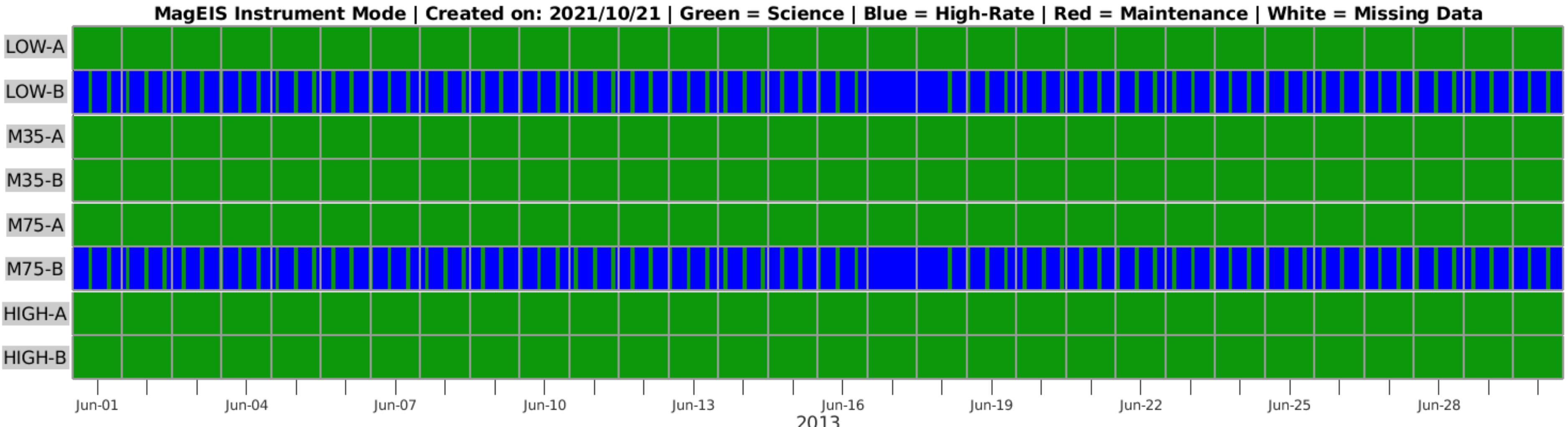
MagEIS Instrument Mode | Created on: 2021/10/21 | Green = Science | Blue = High-Rate | Red = Maintenance | White = Missing Data
LOW-A
LOW-B
M35-A
M35-B
M75-A
M75-B
HIGH-A
HIGH-B
Jun-01
Jun-04
Jun-07
Jun-10
Jun-13
Jun-16
Jun-19
Jun-22
Jun-25
Jun-28
2013

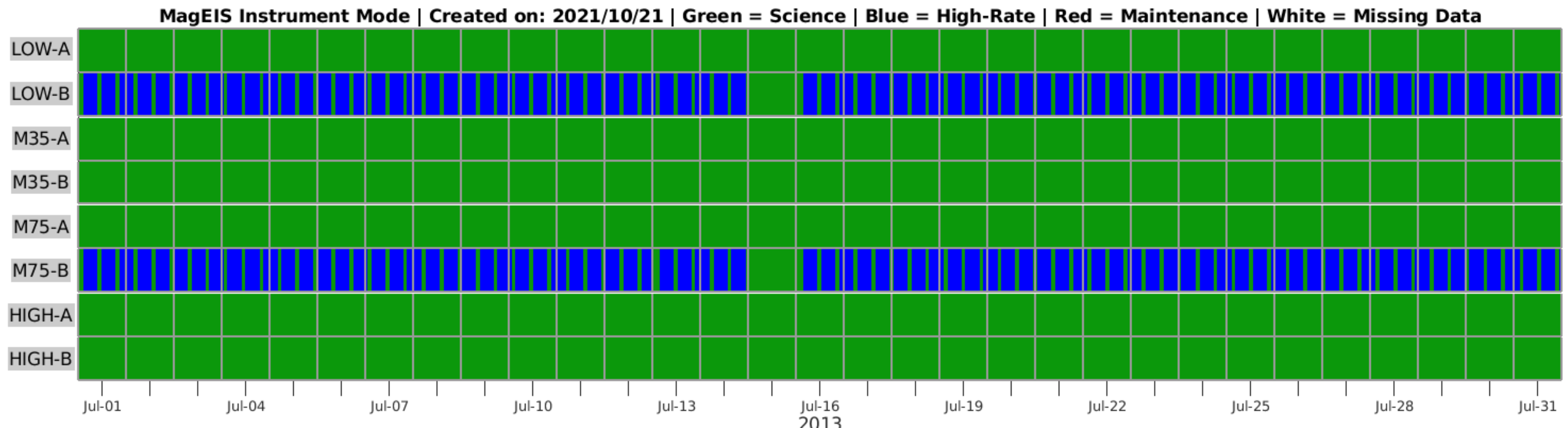
MagEIS Instrument Mode | Created on: 2021/10/21 | Green = Science | Blue = High-Rate | Red = Maintenance | White = Missing Data
LOW-A
LOW-B
M35-A
M35-B
M75-A
M75-B
HIGH-A
HIGH-B
Jul-01
Jul-04
Jul-07
Jul-10
Jul-13
Jul-16
Jul-19
Jul-22
Jul-25
Jul-28
Jul-31
2013

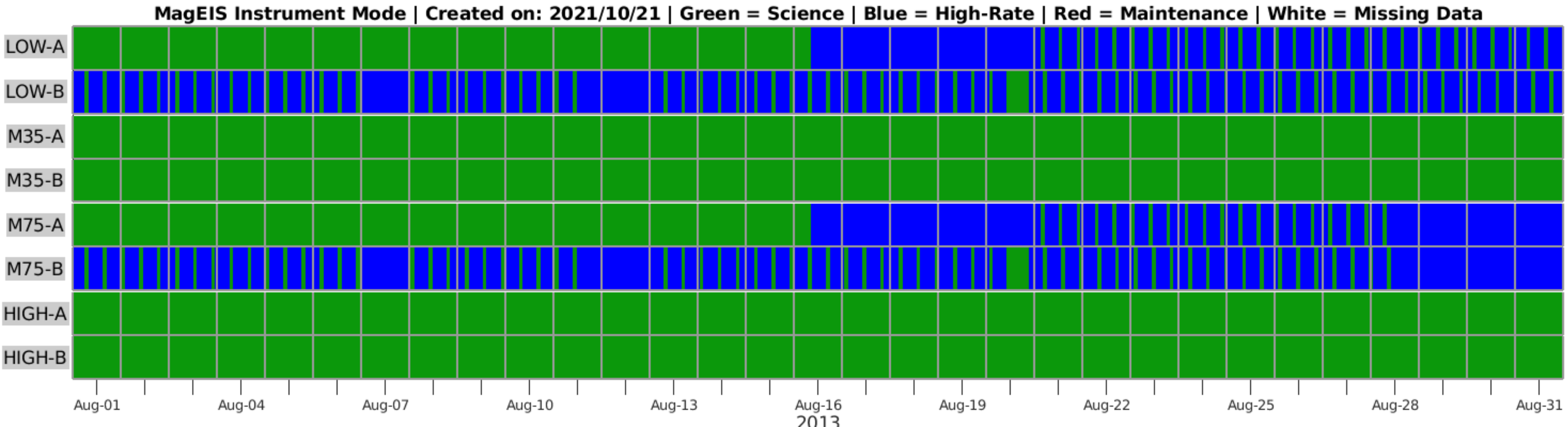

MagEIS Instrument Mode | Created on: 2021/10/21 | Green = Science | Blue = High-Rate | Red = Maintenance | White = Missing Data
LOW-A
LOW-B
M35-A
M35-B
M75-A
M75-B
HIGH-A
HIGH-B
Aug-01
Aug-04
Aug-07
Aug-10
Aug-13
Aug-16
Aug-19
Aug-22
Aug-25
Aug-28
Aug-31
2013

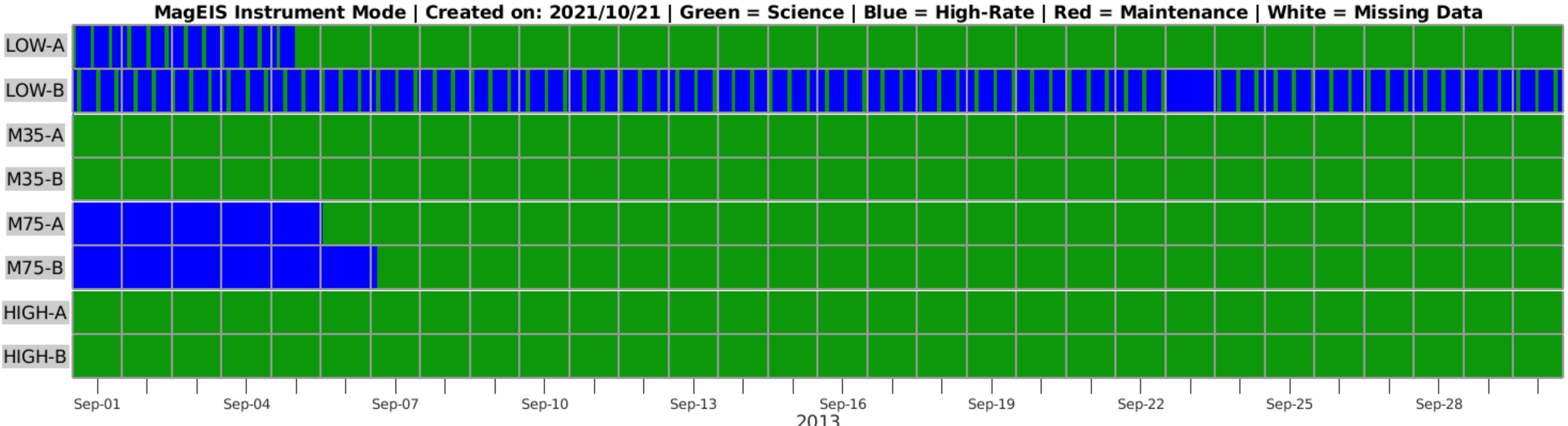
MagEIS Instrument Mode | Created on: 2021/10/21 | Green = Science | Blue = High-Rate | Red = Maintenance | White = Missing Data
LOW-A
LOW-B
M35-A
M35-B
M75-A
M75-B
HIGH-A
HIGH-B
Sep-01
Sep-04
Sep-07
Sep-10
Sep-13
Sep-16
Sep-19
Sep-22
Sep-25
Sep-28
2013

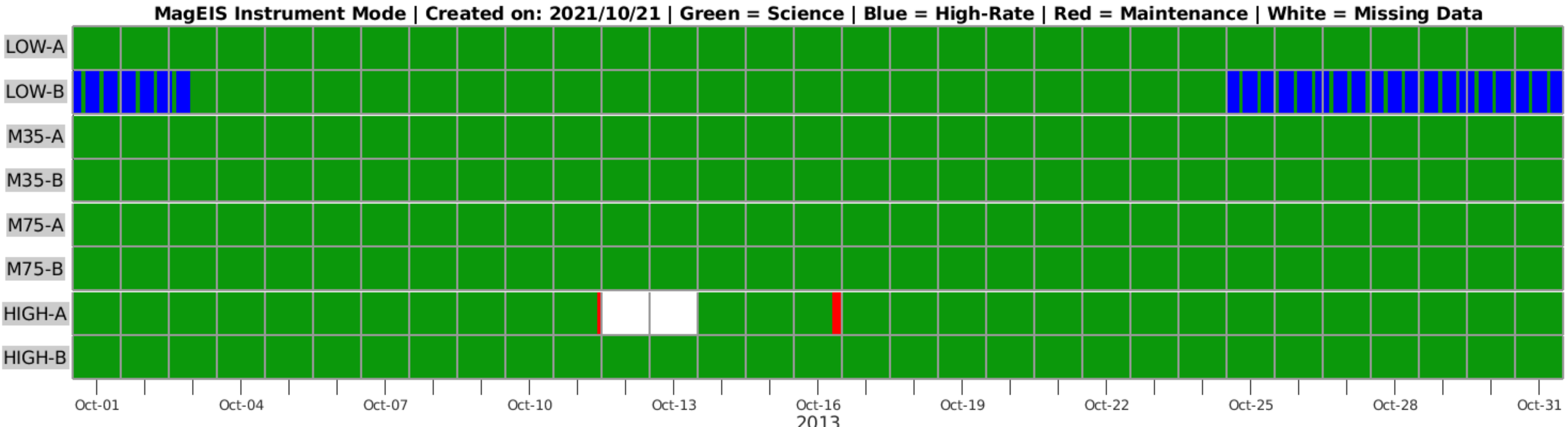
MagEIS Instrument Mode | Created on: 2021/10/21 | Green = Science | Blue = High-Rate | Red = Maintenance | White = Missing Data
LOW-A
LOW-B
M35-A
M35-B
M75-A
M75-B
HIGH-A
HIGH-B
Oct-01
Oct-04
Oct-07
Oct-10
Oct-13
Oct-16
Oct-19
Oct-22
Oct-25
Oct-28
Oct-31
2013

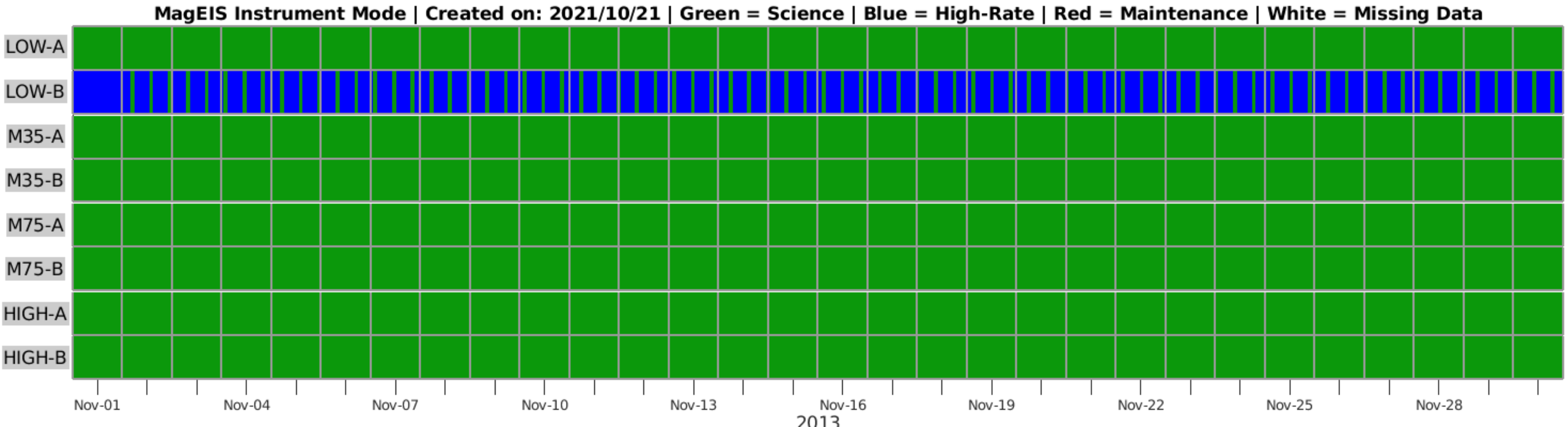
MagEIS Instrument Mode | Created on: 2021/10/21 | Green = Science | Blue = High-Rate | Red = Maintenance | White = Missing Data
LOW-A
LOW-B
M35-A
M35-B
M75-A
M75-B
HIGH-A
HIGH-B
Nov-01
Nov-04
Nov-07
Nov-10
Nov-13
Nov-16
Nov-19
Nov-22
Nov-25
Nov-28
2013

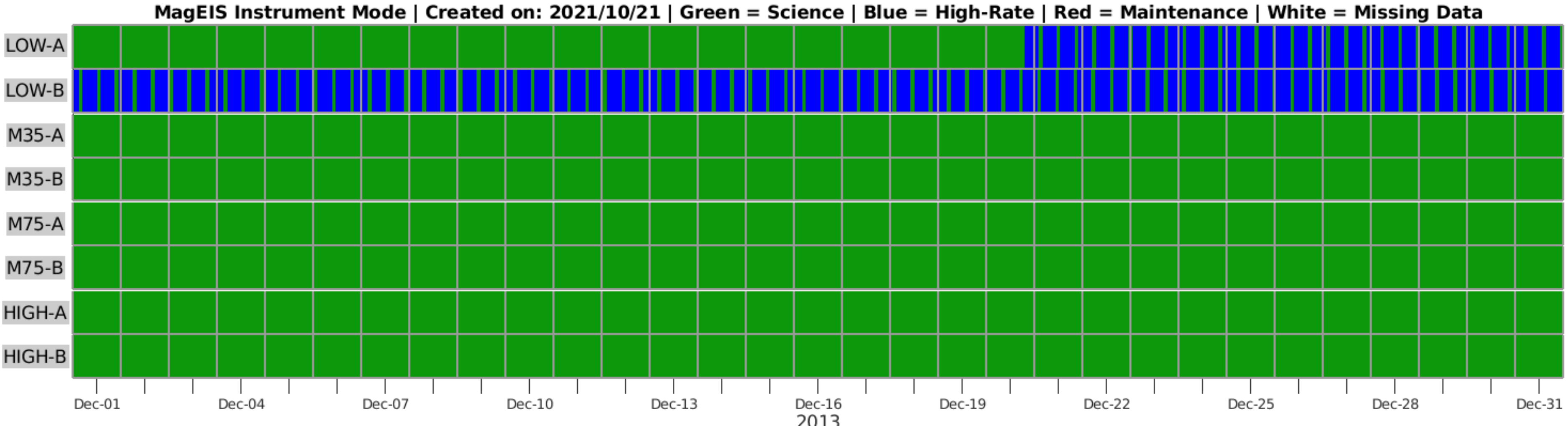
MagEIS Instrument Mode | Created on: 2021/10/21 | Green = Science | Blue = High-Rate | Red = Maintenance | White = Missing Data
LOW-A
LOW-B
M35-A
M35-B
M75-A
M75-B
HIGH-A
HIGH-B
Dec-01
Dec-04
Dec-07
Dec-10
Dec-13
Dec-16
Dec-19
Dec-22
Dec-25
Dec-28
Dec-31
2013

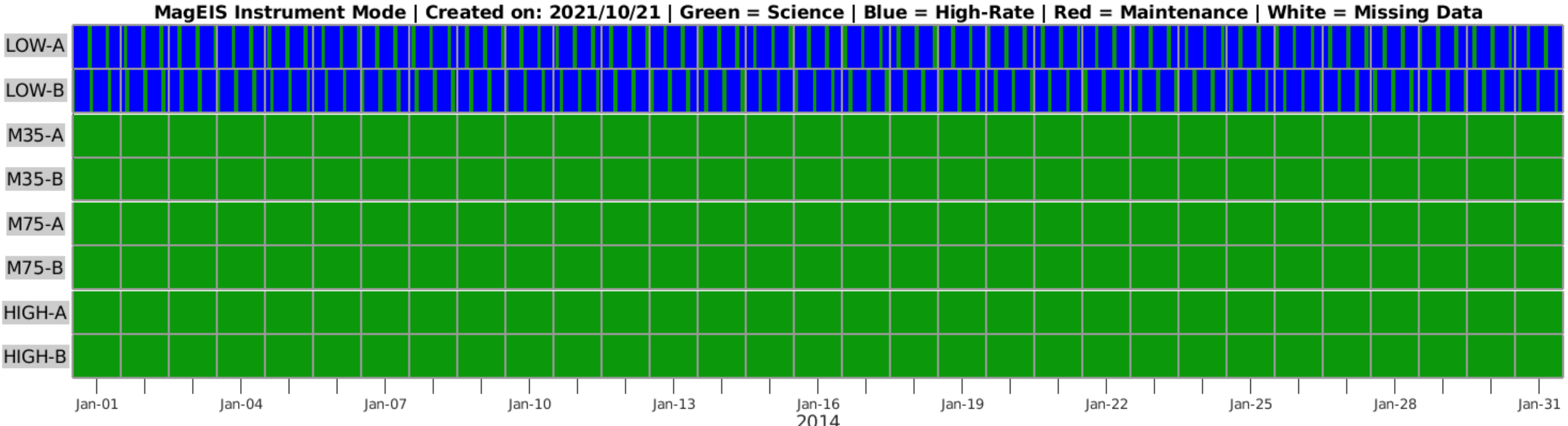
MagEIS Instrument Mode | Created on: 2021/10/21 | Green = Science | Blue = High-Rate | Red = Maintenance | White = Missing Data
LOW-A
LOW-B
M35-A
M35-B
M75-A
M75-B
HIGH-A
HIGH-B
Jan-01
Jan-04
Jan-07
Jan-10
Jan-13
Jan-16
Jan-19
Jan-22
Jan-25
Jan-28
Jan-31
2014

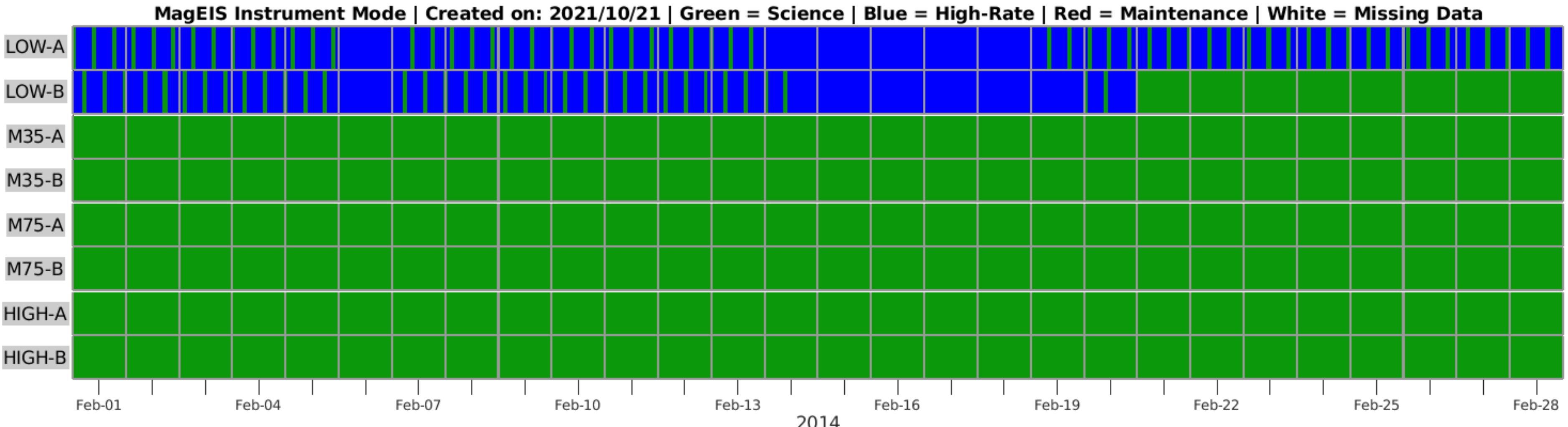
MagEIS Instrument Mode | Created on: 2021/10/21 | Green = Science | Blue = High-Rate | Red = Maintenance | White = Missing Data
LOW-A
LOW-B
M35-A
M35-B
M75-A
M75-B
HIGH-A
HIGH-B
Feb-01
Feb-04
Feb-07
Feb-10
Feb-13
Feb-16
Feb-19
Feb-22
Feb-25
Feb-28
2014

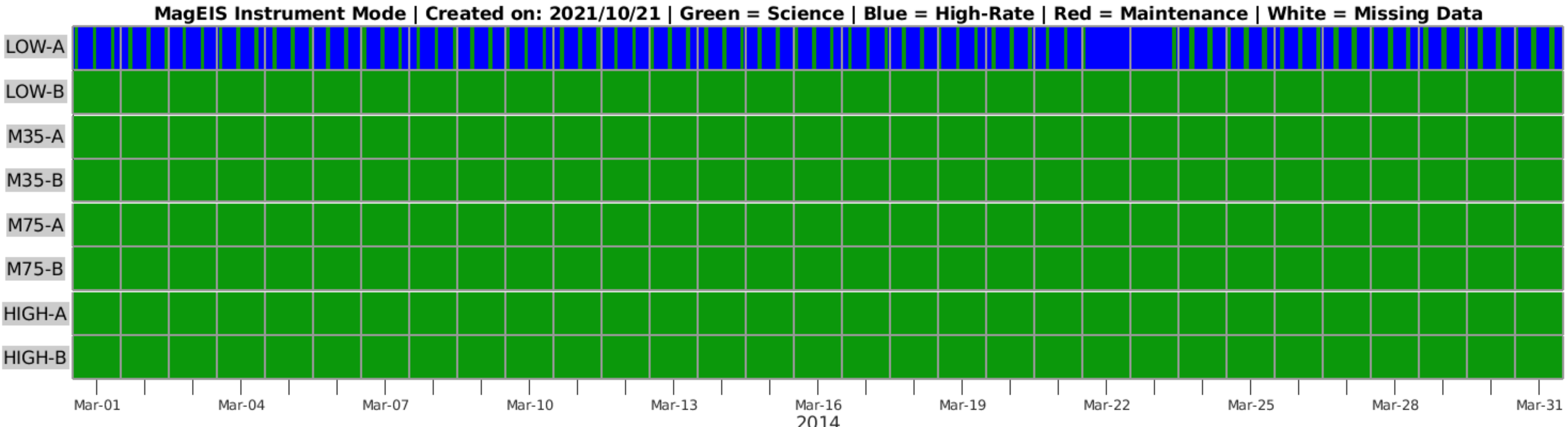

MagEIS Instrument Mode | Created on: 2021/10/21 | Green = Science | Blue = High-Rate | Red = Maintenance | White = Missing Data
LOW-A
LOW-B
M35-A
M35-B
M75-A
M75-B
HIGH-A
HIGH-B
Mar-01
Mar-04
Mar-07
Mar-10
Mar-13
Mar-16
Mar-19
Mar-22
Mar-25
Mar-28
Mar-31
2014

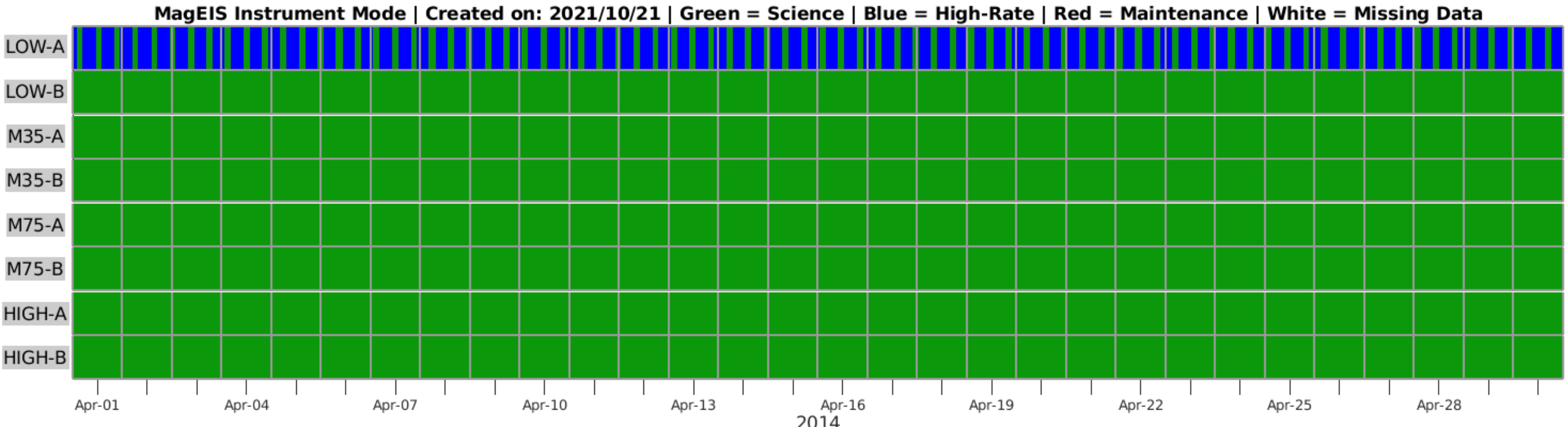
MagEIS Instrument Mode | Created on: 2021/10/21 | Green = Science | Blue = High-Rate | Red = Maintenance | White = Missing Data
LOW-A
LOW-B
M35-A
M35-B
M75-A
M75-B
HIGH-A
HIGH-B
Apr-01
Apr-04
Apr-07
Apr-10
Apr-13
Apr-16
Apr-19
Apr-22
Apr-25
Apr-28
2014

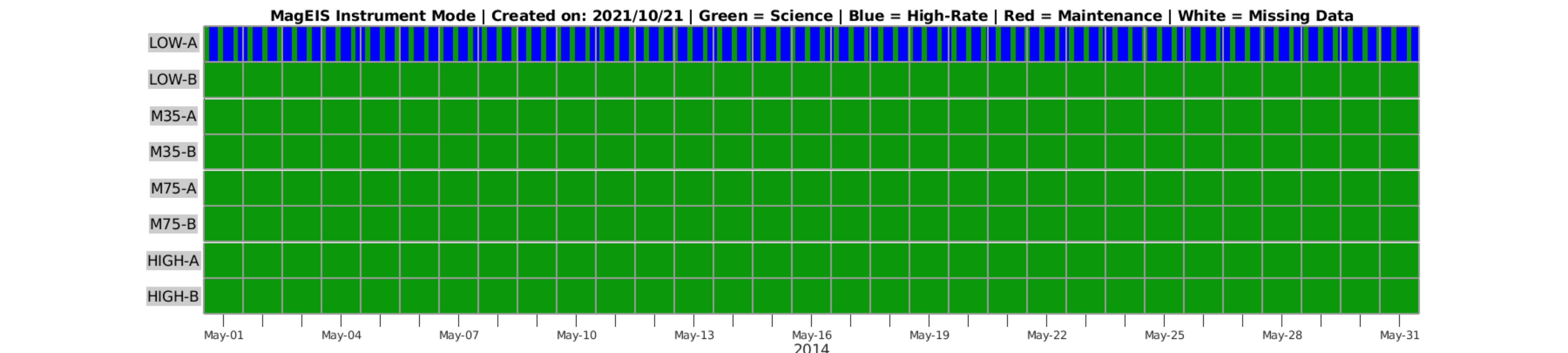

MagEIS Instrument Mode | Created on: 2021/10/21 | Green = Science | Blue = High-Rate | Red = Maintenance | White = Missing Data
LOW-A
LOW-B
M35-A
M35-B
M75-A
M75-B
HIGH-A
HIGH-B
May-01
May-04
May-07
May-10
May-13
May-16
May-19
May-22
May-25
May-28
May-31
2014

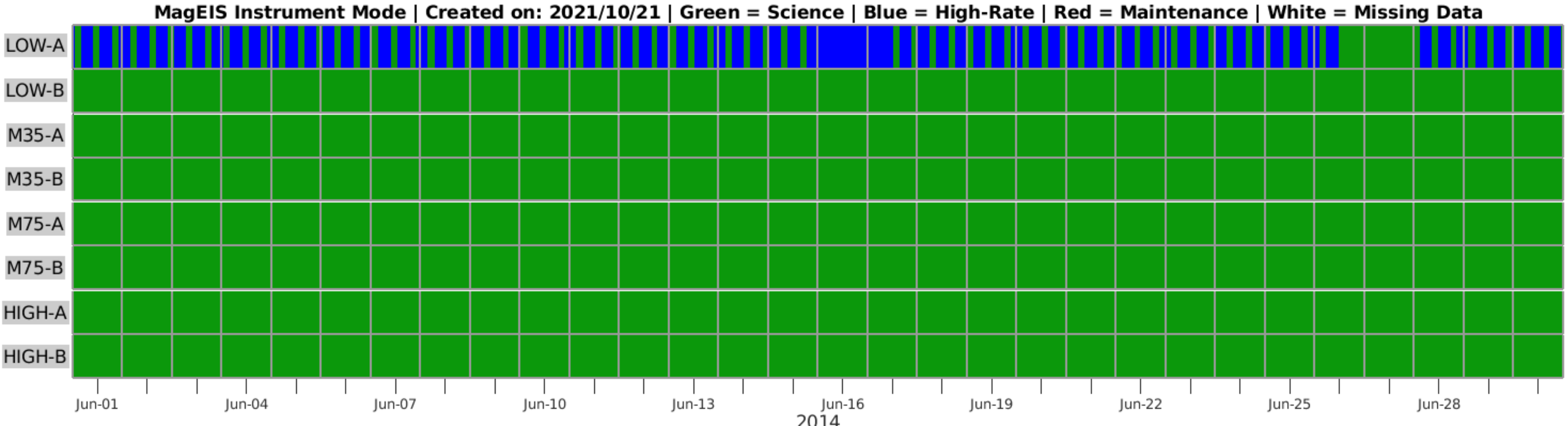
MagEIS Instrument Mode | Created on: 2021/10/21 | Green = Science | Blue = High-Rate | Red = Maintenance | White = Missing Data
LOW-A
LOW-B
M35-A
M35-B
M75-A
M75-B
HIGH-A
HIGH-B
Jun-01
Jun-04
Jun-07
Jun-10
Jun-13
Jun-16
Jun-19
Jun-22
Jun-25
Jun-28
2014

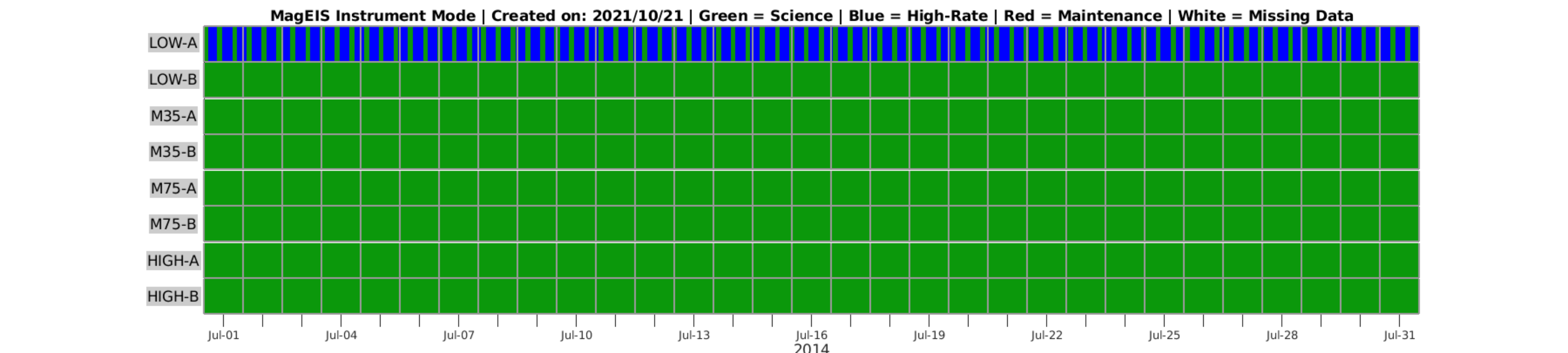
MagEIS Instrument Mode | Created on: 2021/10/21 | Green = Science | Blue = High-Rate | Red = Maintenance | White = Missing Data
LOW-A
LOW-B
M35-A
M35-B
M75-A
M75-B
HIGH-A
HIGH-B
Jul-01
Jul-04
Jul-07
Jul-10
Jul-13
Jul-16
Jul-19
Jul-22
Jul-25
Jul-28
Jul-31
2014

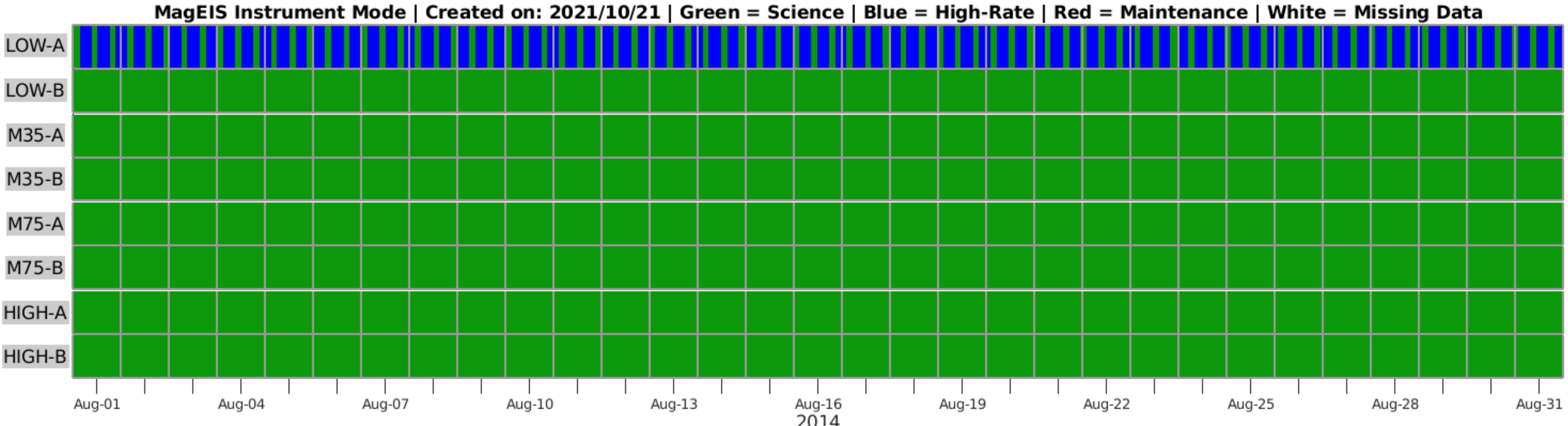
MagEIS Instrument Mode | Created on: 2021/10/21 | Green = Science | Blue = High-Rate | Red = Maintenance | White = Missing Data
LOW-A
LOW-B
M35-A
M35-B
M75-A
M75-B
HIGH-A
HIGH-B
Aug-01
Aug-04
Aug-07
Aug-10
Aug-13
Aug-16
Aug-19
Aug-22
Aug-25
Aug-28
Aug-31
2014

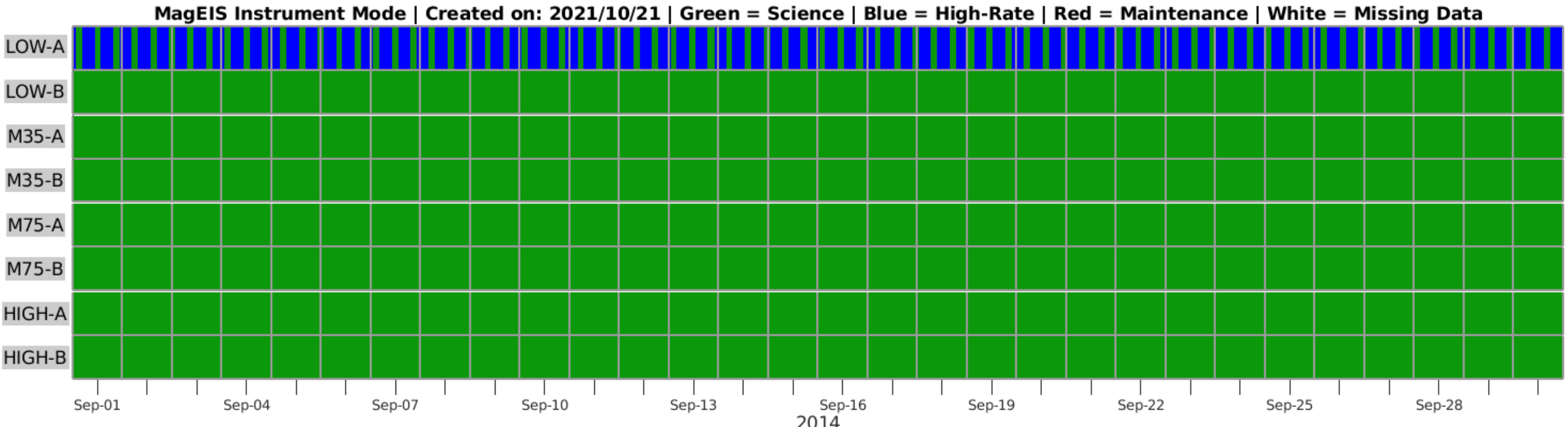
MagEIS Instrument Mode | Created on: 2021/10/21 | Green = Science | Blue = High-Rate | Red = Maintenance | White = Missing Data
LOW-A
LOW-B
M35-A
M35-B
M75-A
M75-B
HIGH-A
HIGH-B
Sep-01
Sep-04
Sep-07
Sep-10
Sep-13
Sep-16
Sep-19
Sep-22
Sep-25
Sep-28
2014

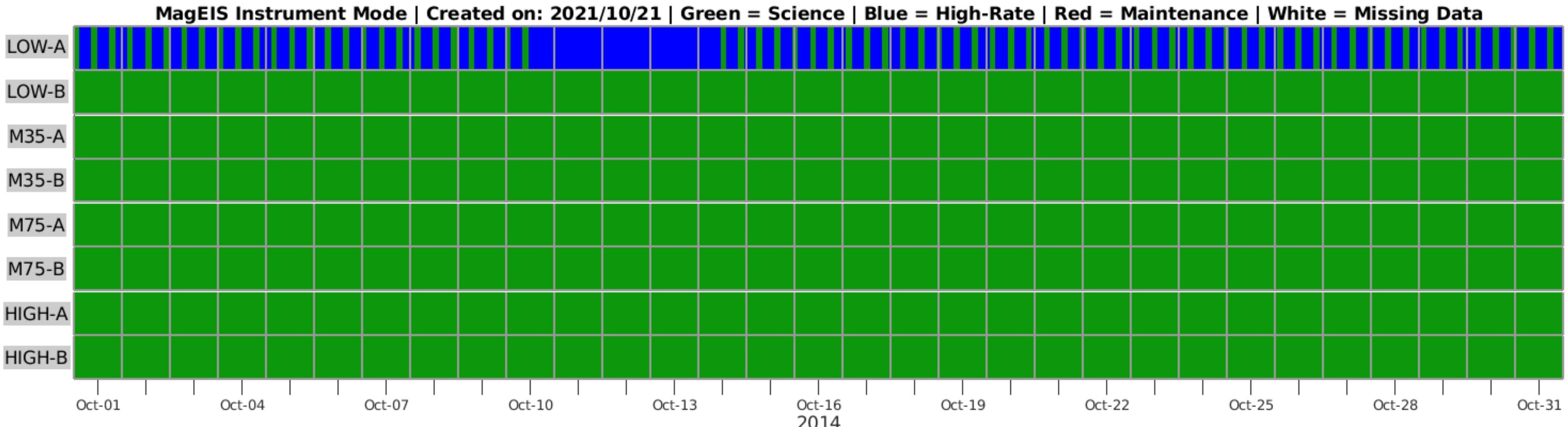
MagEIS Instrument Mode | Created on: 2021/10/21 | Green = Science | Blue = High-Rate | Red = Maintenance | White = Missing Data
LOW-A
LOW-B
M35-A
M35-B
M75-A
M75-B
HIGH-A
HIGH-B
Oct-01
Oct-04
Oct-07
Oct-10
Oct-13
Oct-16
Oct-19
Oct-22
Oct-25
Oct-28
Oct-31
2014

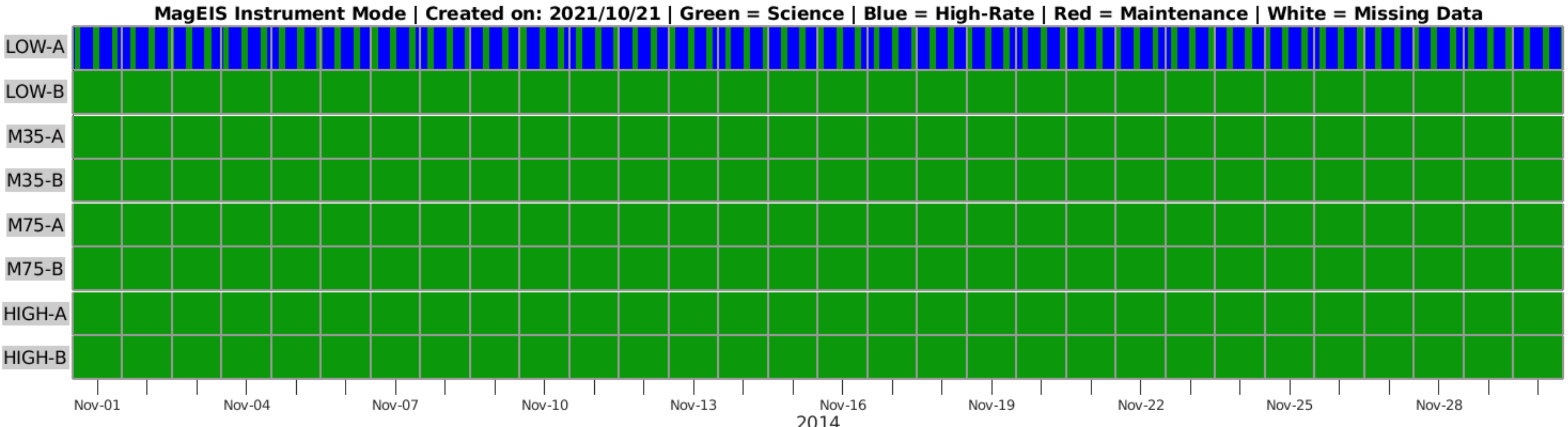
MagEIS Instrument Mode | Created on: 2021/10/21 | Green = Science | Blue = High-Rate | Red = Maintenance | White = Missing Data
LOW-A
LOW-B
M35-A
M35-B
M75-A
M75-B
HIGH-A
HIGH-B
Nov-01
Nov-04
Nov-07
Nov-10
Nov-13
Nov-16
Nov-19
Nov-22
Nov-25
Nov-28
2014

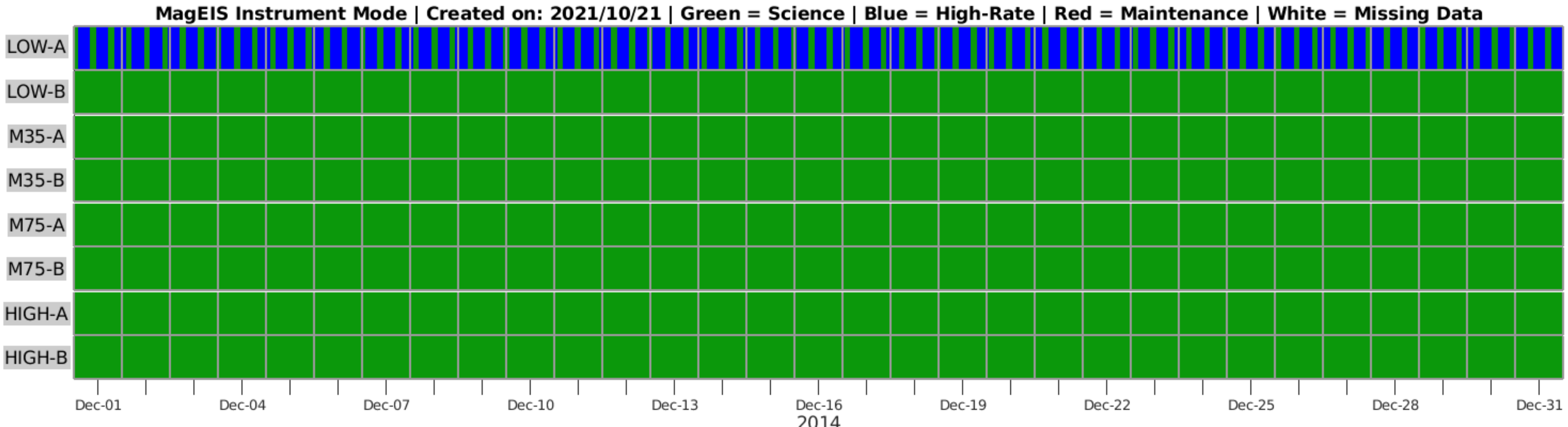

MagEIS Instrument Mode | Created on: 2021/10/21 | Green = Science | Blue = High-Rate | Red = Maintenance | White = Missing Data
LOW-A
LOW-B
M35-A
M35-B
M75-A
M75-B
HIGH-A
HIGH-B
Dec-01
Dec-04
Dec-07
Dec-10
Dec-13
Dec-16
Dec-19
Dec-22
Dec-25
Dec-28
Dec-31
2014

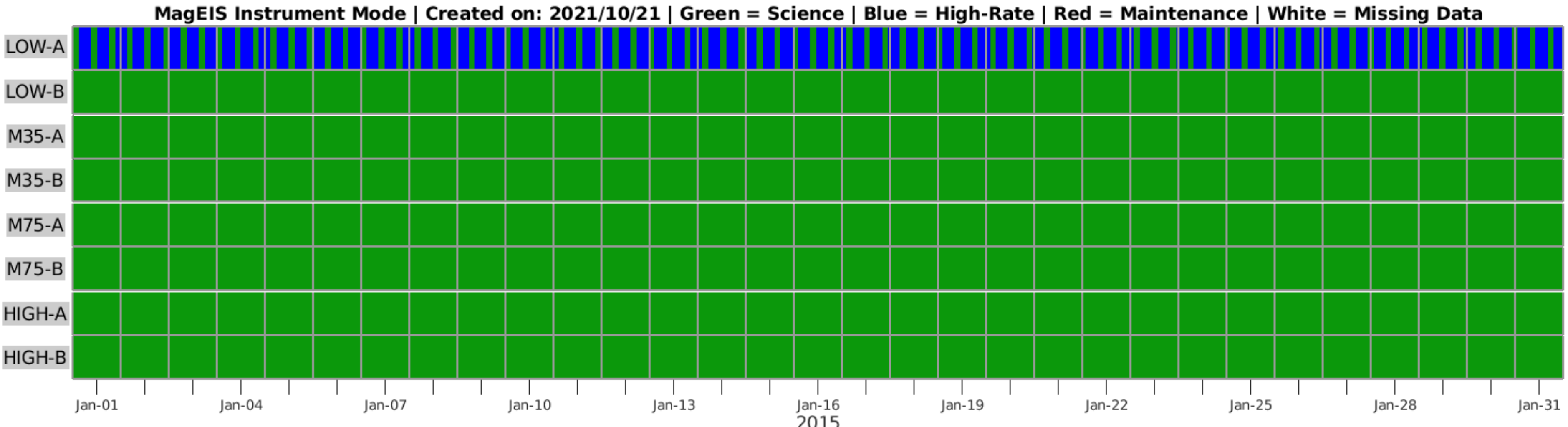
MagEIS Instrument Mode | Created on: 2021/10/21 | Green = Science | Blue = High-Rate | Red = Maintenance | White = Missing Data
LOW-A
LOW-B
M35-A
M35-B
M75-A
M75-B
HIGH-A
HIGH-B
Jan-01
Jan-04
Jan-07
Jan-10
Jan-13
Jan-16
Jan-19
Jan-22
Jan-25
Jan-28
Jan-31
2015

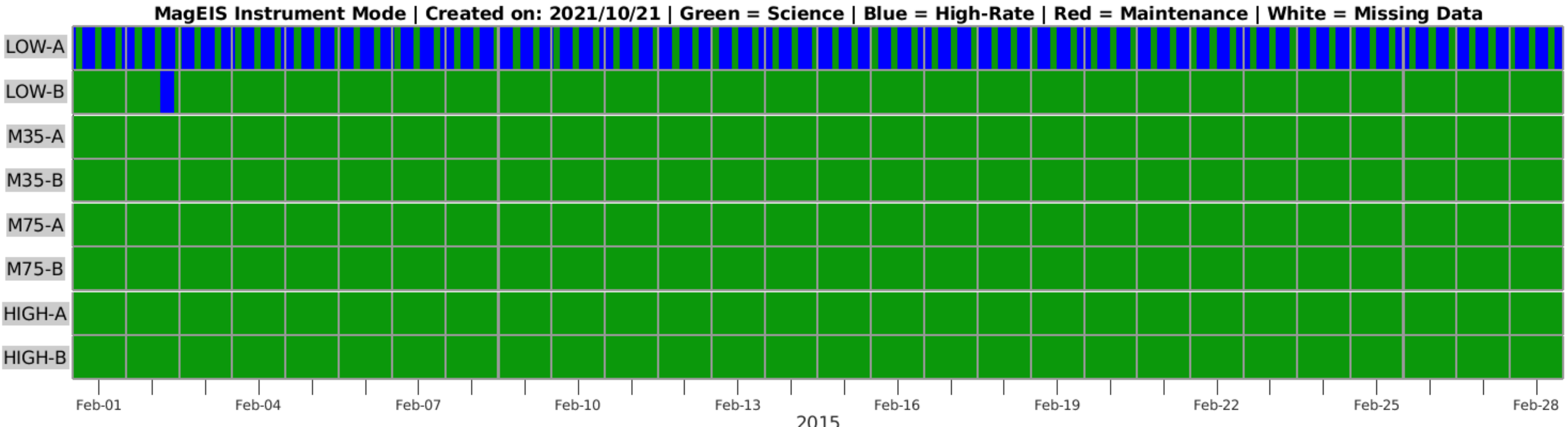
MagEIS Instrument Mode | Created on: 2021/10/21 | Green = Science | Blue = High-Rate | Red = Maintenance | White = Missing Data
LOW-A
LOW-B
M35-A
M35-B
M75-A
M75-B
HIGH-A
HIGH-B
Feb-01
Feb-04
Feb-07
Feb-10
Feb-13
Feb-16
Feb-19
Feb-22
Feb-25
Feb-28
2015

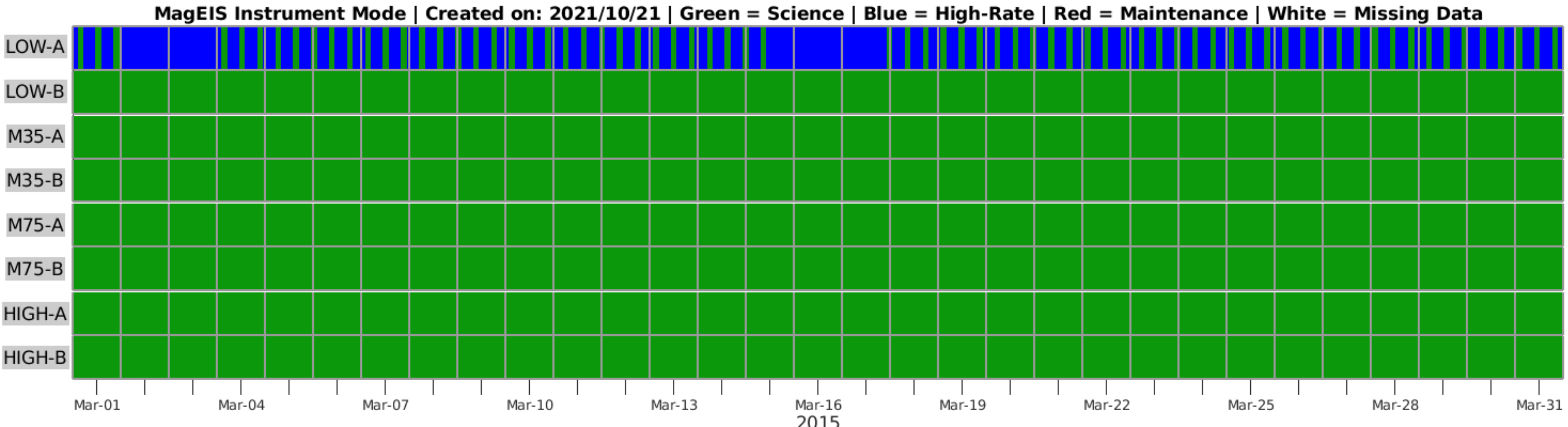

MagEIS Instrument Mode | Created on: 2021/10/21 | Green = Science | Blue = High-Rate | Red = Maintenance | White = Missing Data
LOW-A
LOW-B
M35-A
M35-B
M75-A
M75-B
HIGH-A
HIGH-B
Mar-01
Mar-04
Mar-07
Mar-10
Mar-13
Mar-16
Mar-19
Mar-22
Mar-25
Mar-28
Mar-31
2015

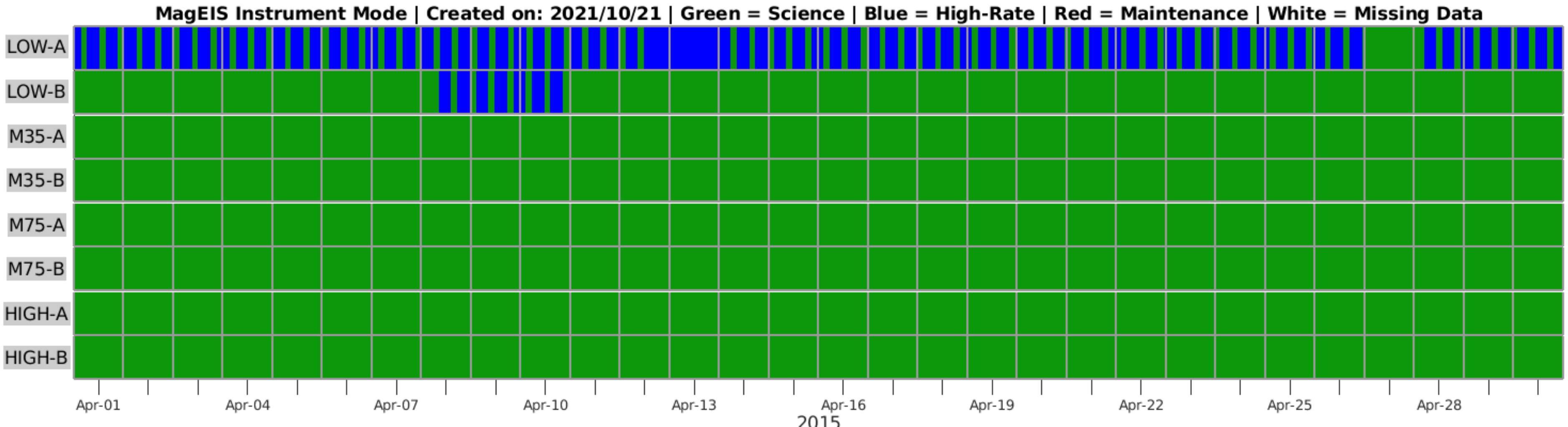
MagEIS Instrument Mode | Created on: 2021/10/21 | Green = Science | Blue = High-Rate | Red = Maintenance | White = Missing Data
LOW-A
LOW-B
M35-A
M35-B
M75-A
M75-B
HIGH-A
HIGH-B
Apr-01
Apr-04
Apr-07
Apr-10
Apr-13
Apr-16
Apr-19
Apr-22
Apr-25
Apr-28
2015

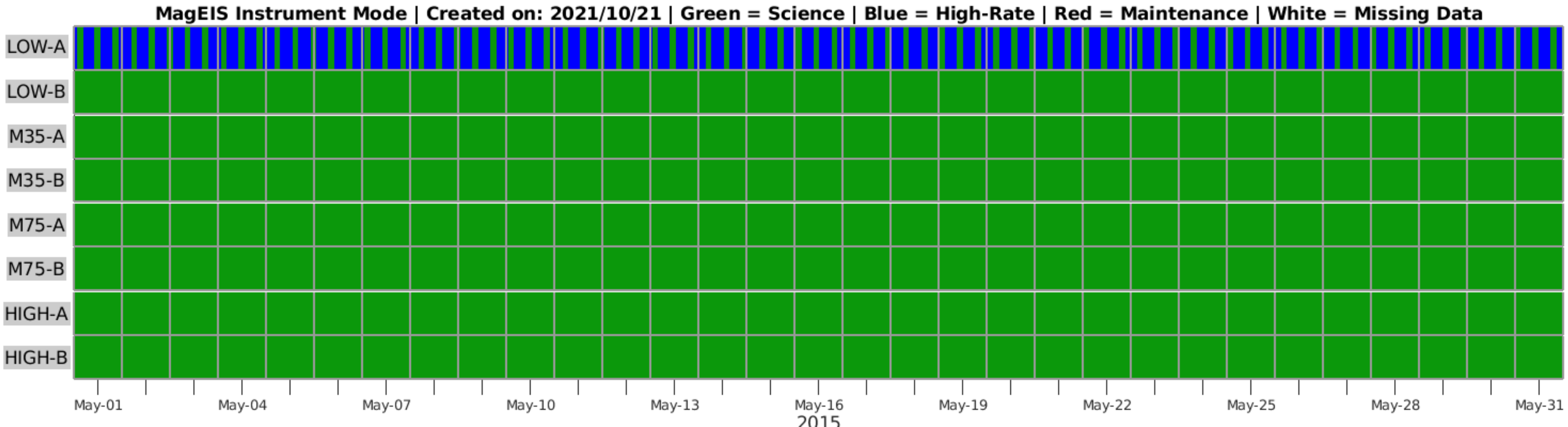

MagEIS Instrument Mode | Created on: 2021/10/21 | Green = Science | Blue = High-Rate | Red = Maintenance | White = Missing Data
LOW-A
LOW-B
M35-A
M35-B
M75-A
M75-B
HIGH-A
HIGH-B
May-01
May-04
May-07
May-10
May-13
May-16
May-19
May-22
May-25
May-28
May-31
2015

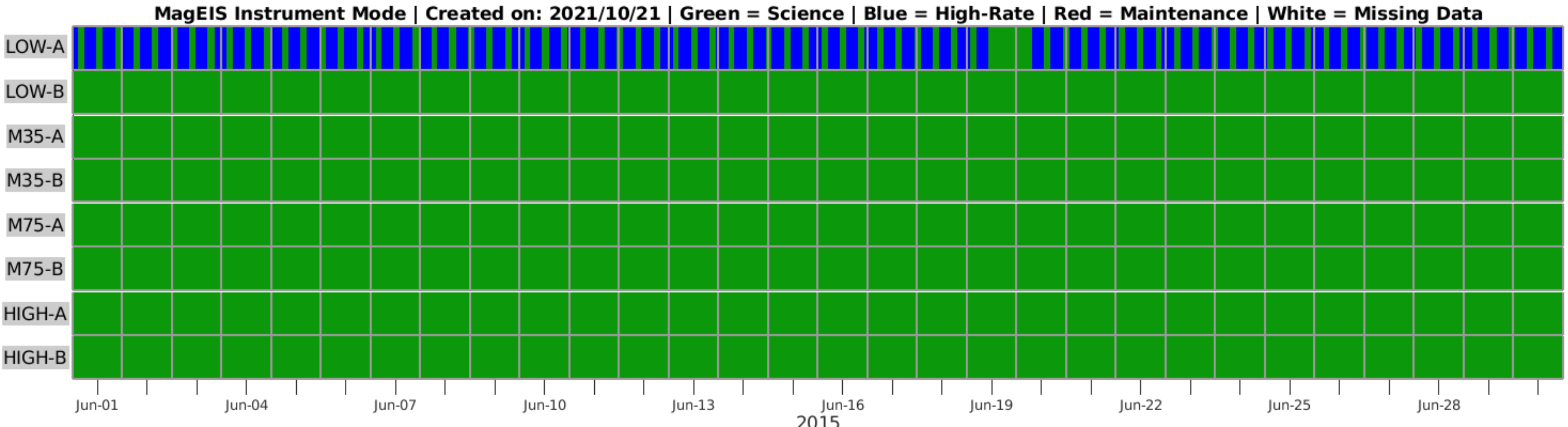
MagEIS Instrument Mode | Created on: 2021/10/21 | Green = Science | Blue = High-Rate | Red = Maintenance | White = Missing Data
LOW-A
LOW-B
M35-A
M35-B
M75-A
M75-B
HIGH-A
HIGH-B
Jun-01
Jun-04
Jun-07
Jun-10
Jun-13
Jun-16
Jun-19
Jun-22
Jun-25
Jun-28
2015

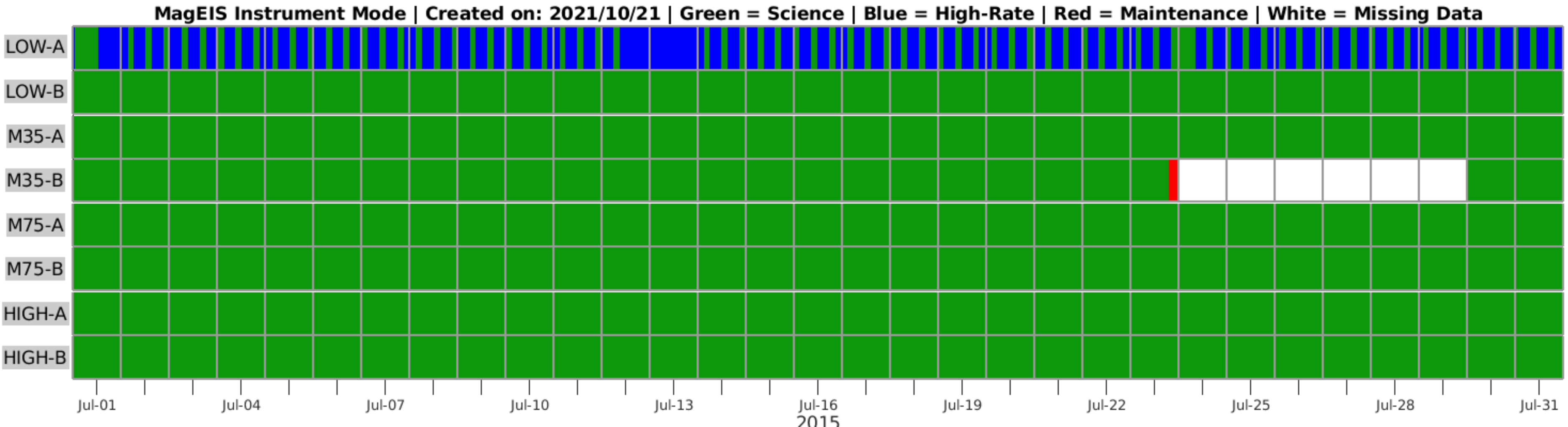
MagEIS Instrument Mode | Created on: 2021/10/21 | Green = Science | Blue = High-Rate | Red = Maintenance | White = Missing Data
LOW-A
LOW-B
M35-A
M35-B
M75-A
M75-B
HIGH-A
HIGH-B
Jul-01
Jul-04
Jul-07
Jul-10
Jul-13
Jul-16
Jul-19
Jul-22
Jul-25
Jul-28
Jul-31
2015

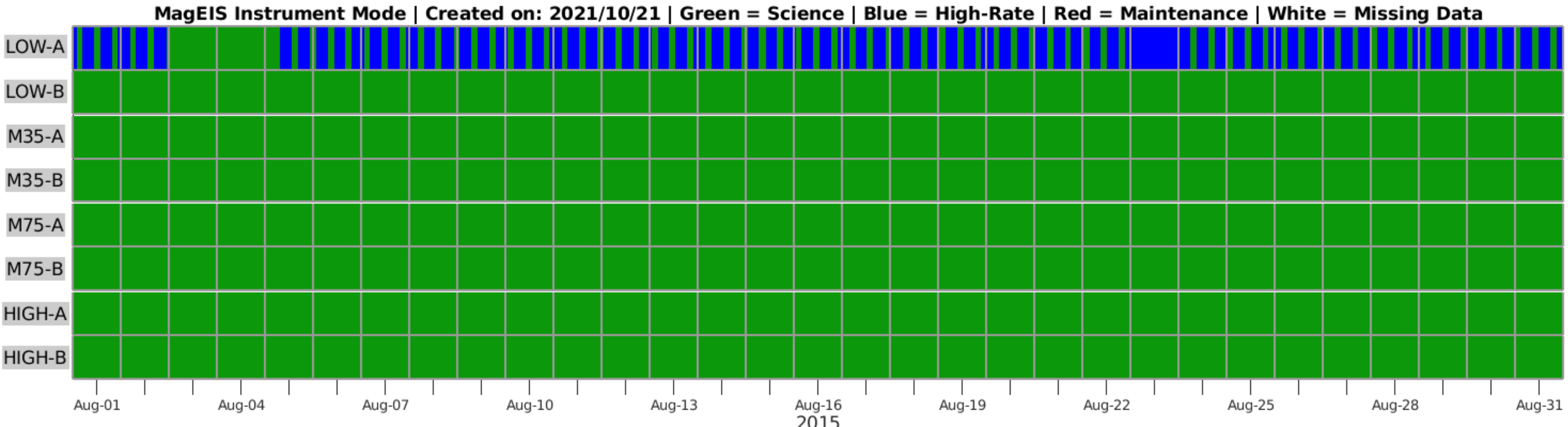
MagEIS Instrument Mode | Created on: 2021/10/21 | Green = Science | Blue = High-Rate | Red = Maintenance | White = Missing Data
LOW-A
LOW-B
M35-A
M35-B
M75-A
M75-B
HIGH-A
HIGH-B
Aug-01
Aug-04
Aug-07
Aug-10
Aug-13
Aug-16
Aug-19
Aug-22
Aug-25
Aug-28
Aug-31
2015

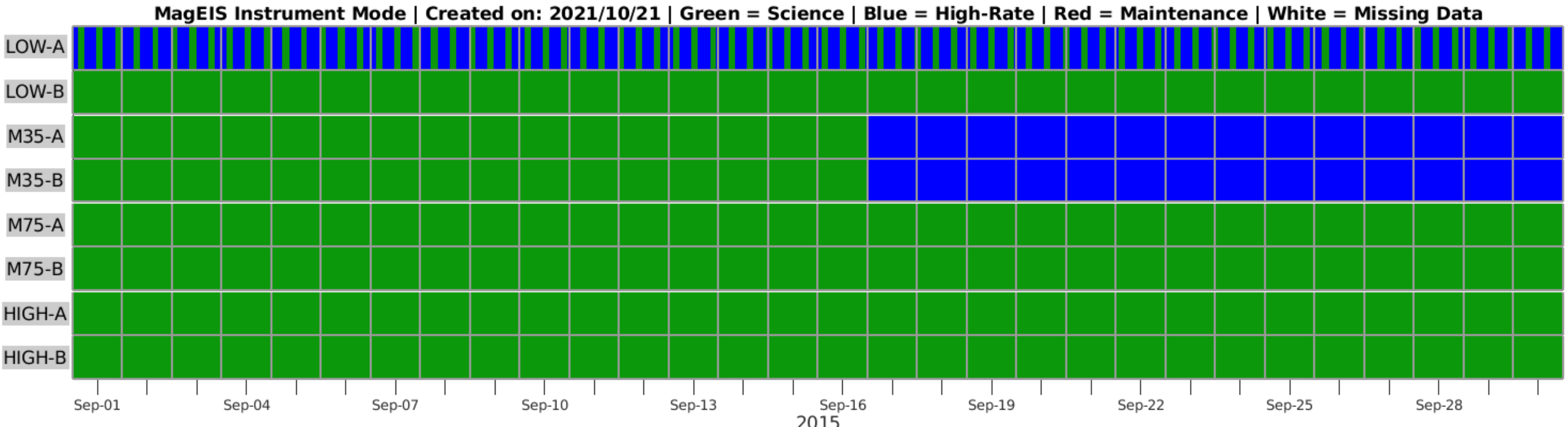

MagEIS Instrument Mode | Created on: 2021/10/21 | Green = Science | Blue = High-Rate | Red = Maintenance | White = Missing Data
LOW-A
LOW-B
M35-A
M35-B
M75-A
M75-B
HIGH-A
HIGH-B
Sep-01
Sep-04
Sep-07
Sep-10
Sep-13
Sep-16
Sep-19
Sep-22
Sep-25
Sep-28
2015

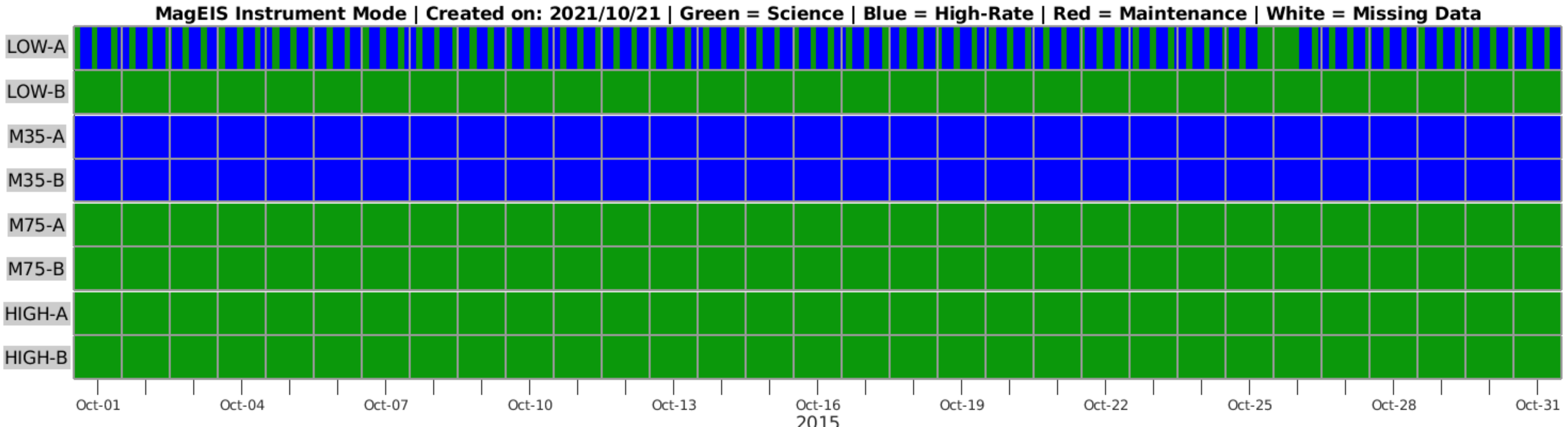

MagEIS Instrument Mode | Created on: 2021/10/21 | Green = Science | Blue = High-Rate | Red = Maintenance | White = Missing Data
LOW-A
LOW-B
M35-A
M35-B
M75-A
M75-B
HIGH-A
HIGH-B
Oct-01
Oct-04
Oct-07
Oct-10
Oct-13
Oct-16
Oct-19
Oct-22
Oct-25
Oct-28
Oct-31
2015

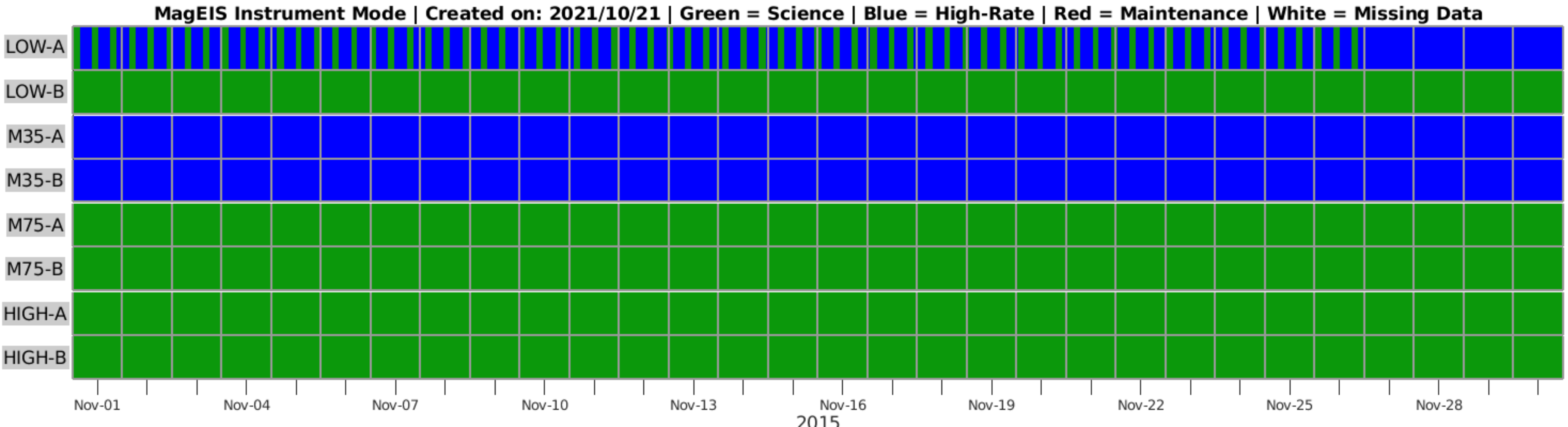

MagEIS Instrument Mode | Created on: 2021/10/21 | Green = Science | Blue = High-Rate | Red = Maintenance | White = Missing Data
LOW-A
LOW-B
M35-A
M35-B
M75-A
M75-B
HIGH-A
HIGH-B
Nov-01
Nov-04
Nov-07
Nov-10
Nov-13
Nov-16
Nov-19
Nov-22
Nov-25
Nov-28
2015

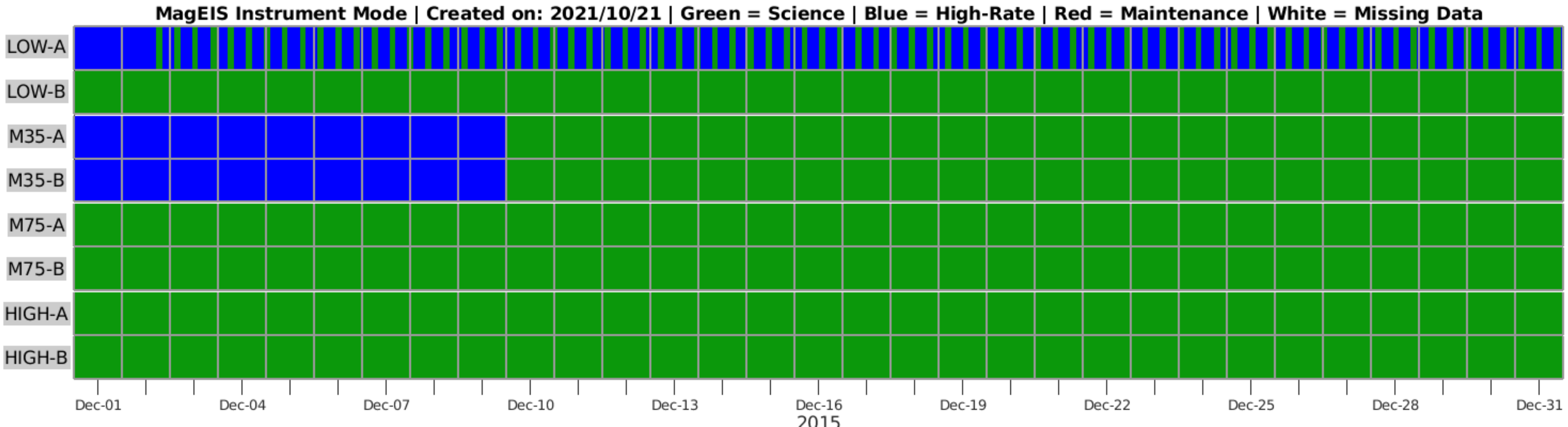
MagEIS Instrument Mode | Created on: 2021/10/21 | Green = Science | Blue = High-Rate | Red = Maintenance | White = Missing Data
LOW-A
LOW-B
M35-A
M35-B
M75-A
M75-B
HIGH-A
HIGH-B
Dec-01
Dec-04
Dec-07
Dec-10
Dec-13
Dec-16
Dec-19
Dec-22
Dec-25
Dec-28
Dec-31
2015

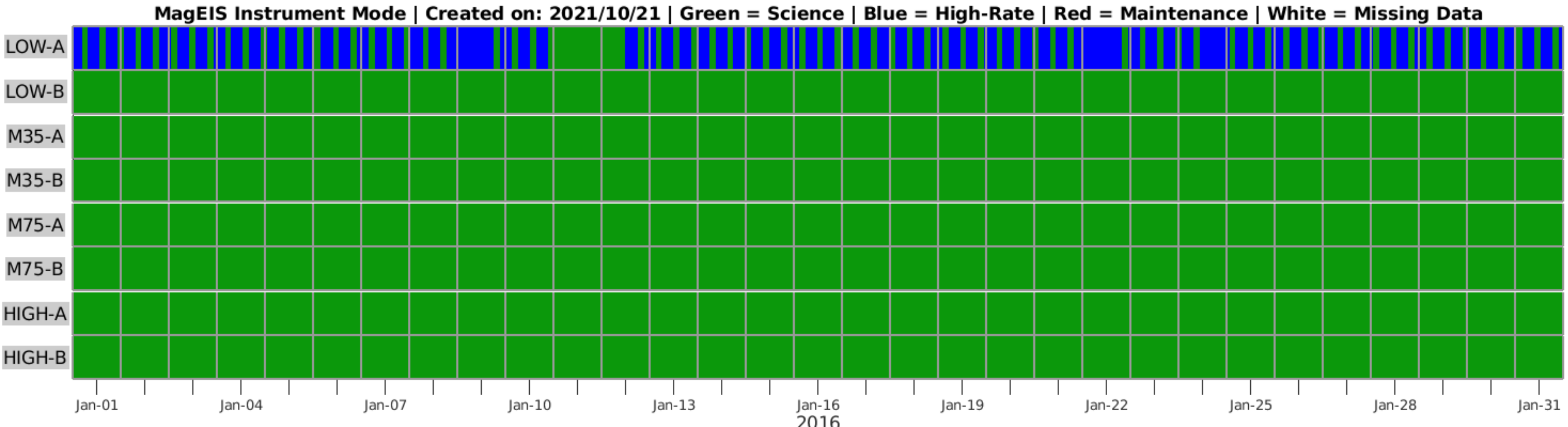
MagEIS Instrument Mode | Created on: 2021/10/21 | Green = Science | Blue = High-Rate | Red = Maintenance | White = Missing Data
LOW-A
LOW-B
M35-A
M35-B
M75-A
M75-B
HIGH-A
HIGH-B
Jan-01
Jan-04
Jan-07
Jan-10
Jan-13
Jan-16
Jan-19
Jan-22
Jan-25
Jan-28
Jan-31
2016

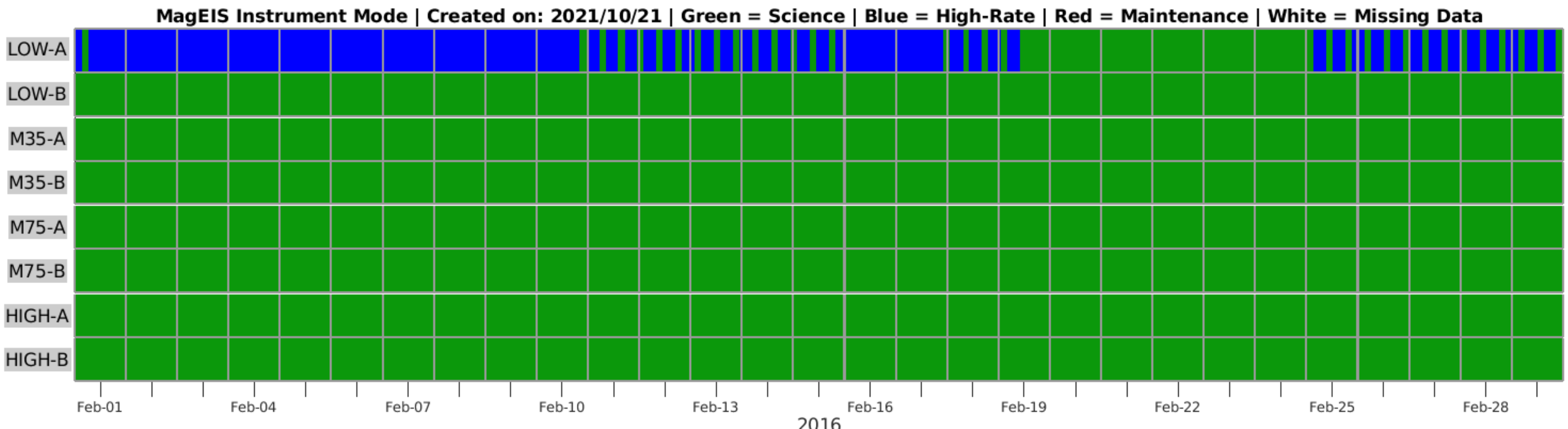
MagEIS Instrument Mode | Created on: 2021/10/21 | Green = Science | Blue = High-Rate | Red = Maintenance | White = Missing Data
LOW-A
LOW-B
M35-A
M35-B
M75-A
M75-B
HIGH-A
HIGH-B
Feb-01
Feb-04
Feb-07
Feb-10
Feb-13
Feb-16
Feb-19
Feb-22
Feb-25
Feb-28
2016

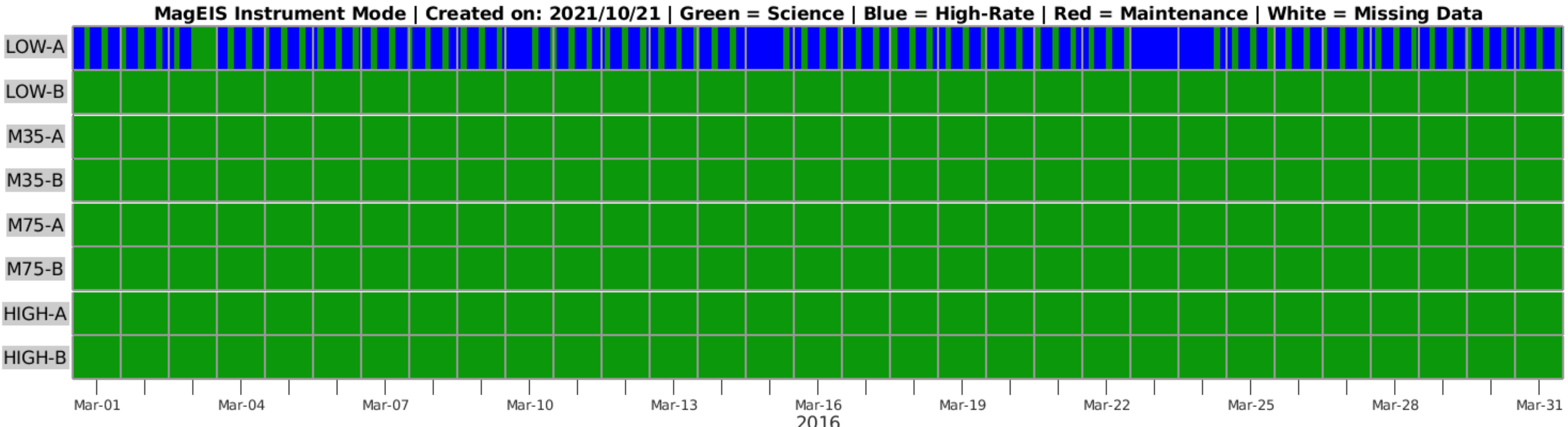
MagEIS Instrument Mode | Created on: 2021/10/21 | Green = Science | Blue = High-Rate | Red = Maintenance | White = Missing Data
LOW-A
LOW-B
M35-A
M35-B
M75-A
M75-B
HIGH-A
HIGH-B
Mar-01
Mar-04
Mar-07
Mar-10
Mar-13
Mar-16
Mar-19
Mar-22
Mar-25
Mar-28
Mar-31
2016

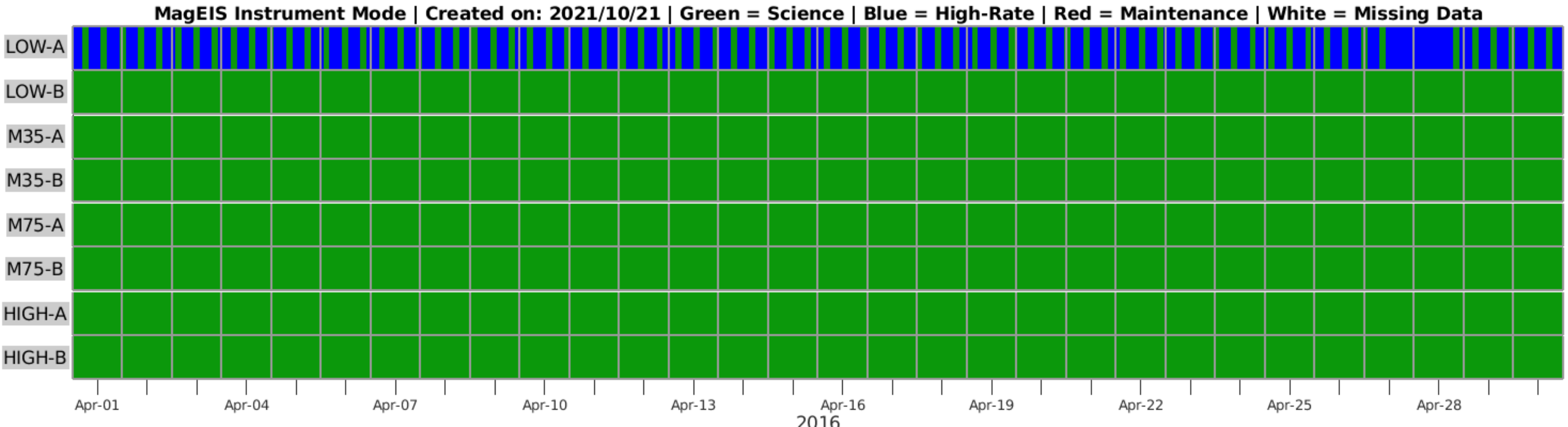
MagEIS Instrument Mode | Created on: 2021/10/21 | Green = Science | Blue = High-Rate | Red = Maintenance | White = Missing Data
LOW-A
LOW-B
M35-A
M35-B
M75-A
M75-B
HIGH-A
HIGH-B
Apr-01
Apr-04
Apr-07
Apr-10
Apr-13
Apr-16
Apr-19
Apr-22
Apr-25
Apr-28
2016

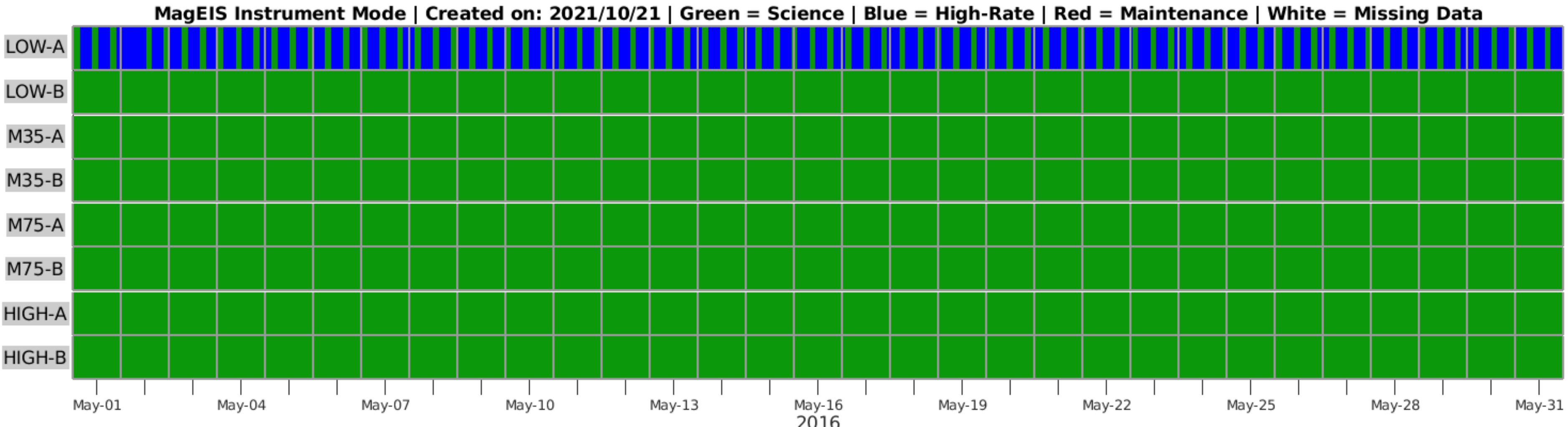
MagEIS Instrument Mode | Created on: 2021/10/21 | Green = Science | Blue = High-Rate | Red = Maintenance | White = Missing Data
LOW-A
LOW-B
M35-A
M35-B
M75-A
M75-B
HIGH-A
HIGH-B
May-01
May-04
May-07
May-10
May-13
May-16
May-19
May-22
May-25
May-28
May-31
2016

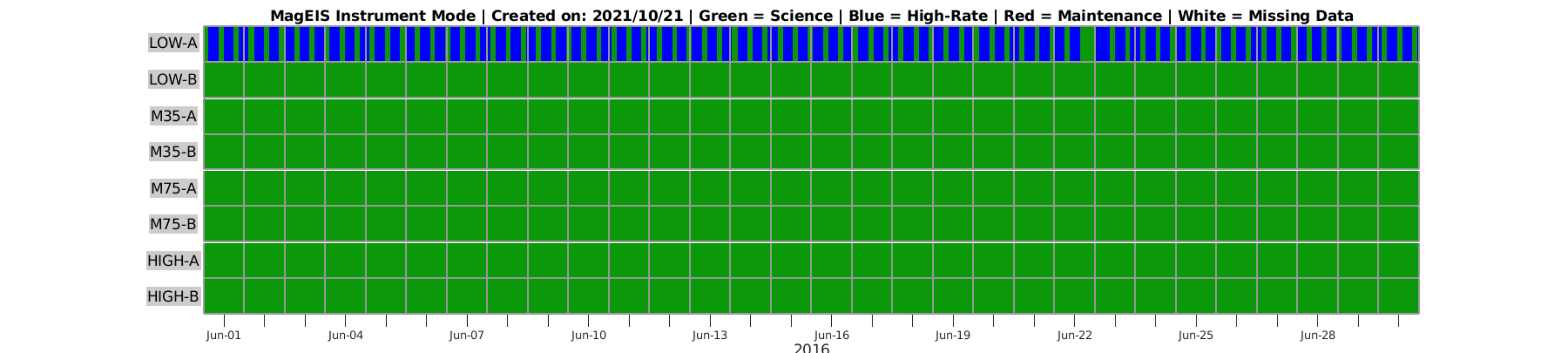
MagEIS Instrument Mode | Created on: 2021/10/21 | Green = Science | Blue = High-Rate | Red = Maintenance | White = Missing Data
LOW-A
LOW-B
M35-A
M35-B
M75-A
M75-B
HIGH-A
HIGH-B
Jun-01
Jun-04
Jun-07
Jun-10
Jun-13
Jun-16
Jun-19
Jun-22
Jun-25
Jun-28
2016

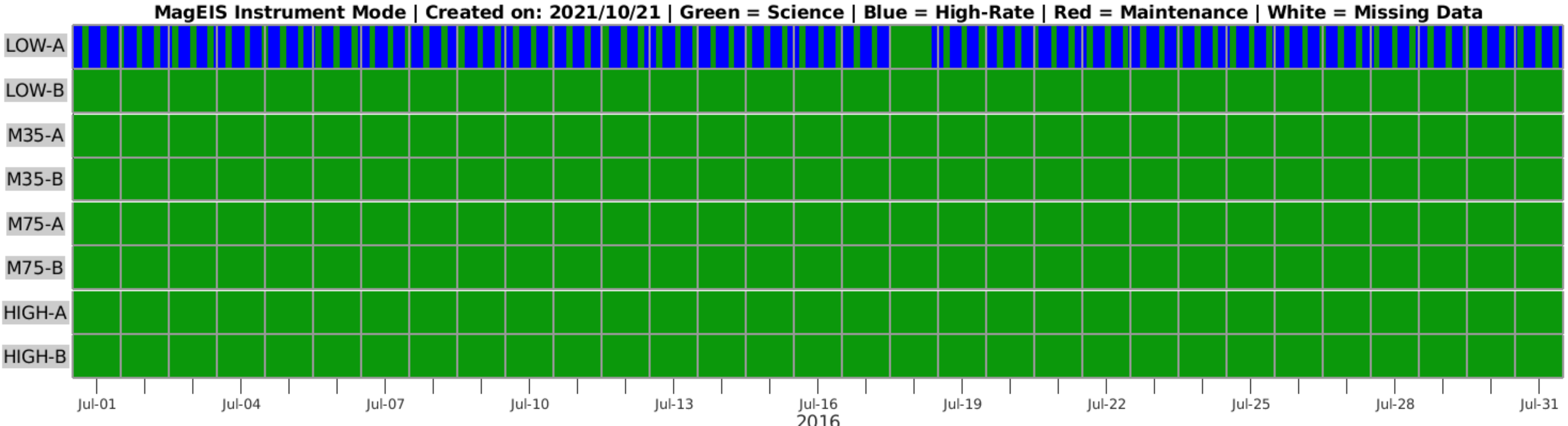
MagEIS Instrument Mode | Created on: 2021/10/21 | Green = Science | Blue = High-Rate | Red = Maintenance | White = Missing Data
LOW-A
LOW-B
M35-A
M35-B
M75-A
M75-B
HIGH-A
HIGH-B
Jul-01
Jul-04
Jul-07
Jul-10
Jul-13
Jul-16
Jul-19
Jul-22
Jul-25
Jul-28
Jul-31
2016

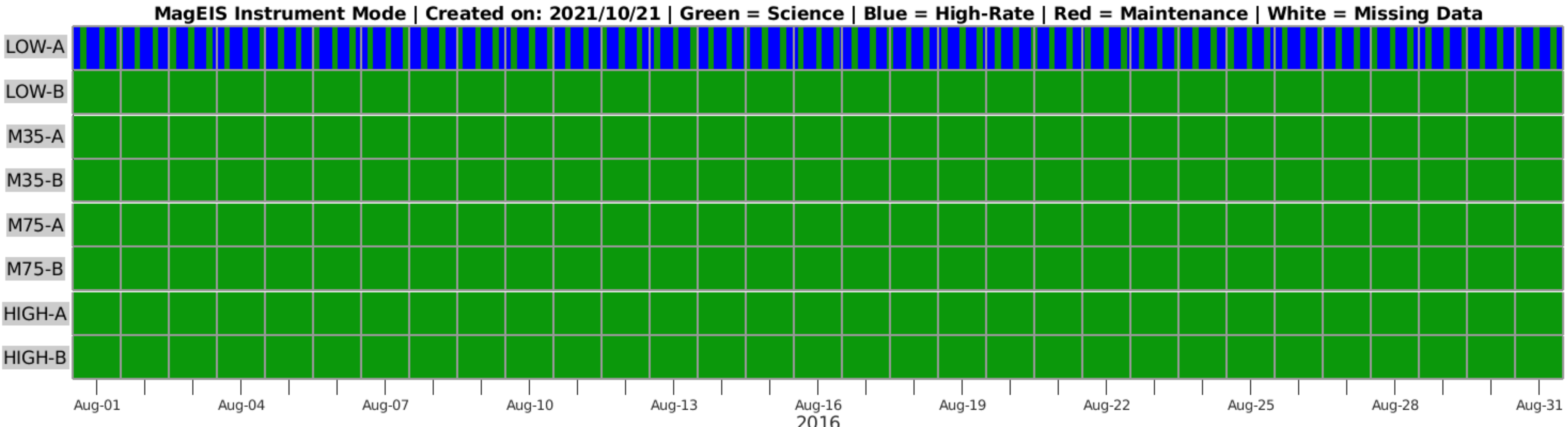
MagEIS Instrument Mode | Created on: 2021/10/21 | Green = Science | Blue = High-Rate | Red = Maintenance | White = Missing Data
LOW-A
LOW-B
M35-A
M35-B
M75-A
M75-B
HIGH-A
HIGH-B
Aug-01
Aug-04
Aug-07
Aug-10
Aug-13
Aug-16
Aug-19
Aug-22
Aug-25
Aug-28
Aug-31
2016

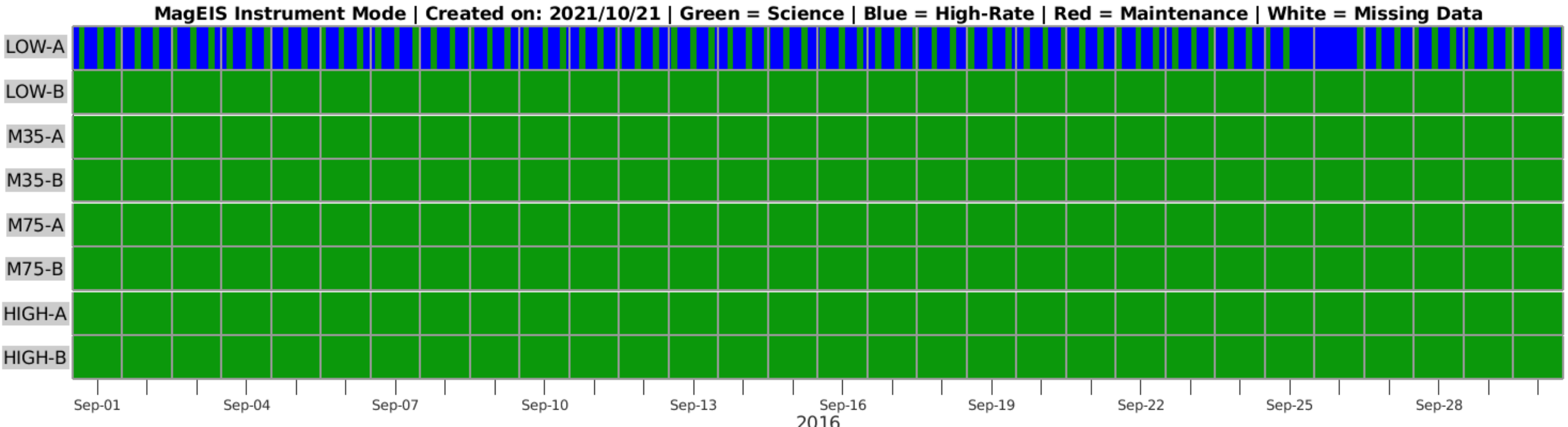
MagEIS Instrument Mode | Created on: 2021/10/21 | Green = Science | Blue = High-Rate | Red = Maintenance | White = Missing Data
LOW-A
LOW-B
M35-A
M35-B
M75-A
M75-B
HIGH-A
HIGH-B
Sep-01
Sep-04
Sep-07
Sep-10
Sep-13
Sep-16
Sep-19
Sep-22
Sep-25
Sep-28
2016

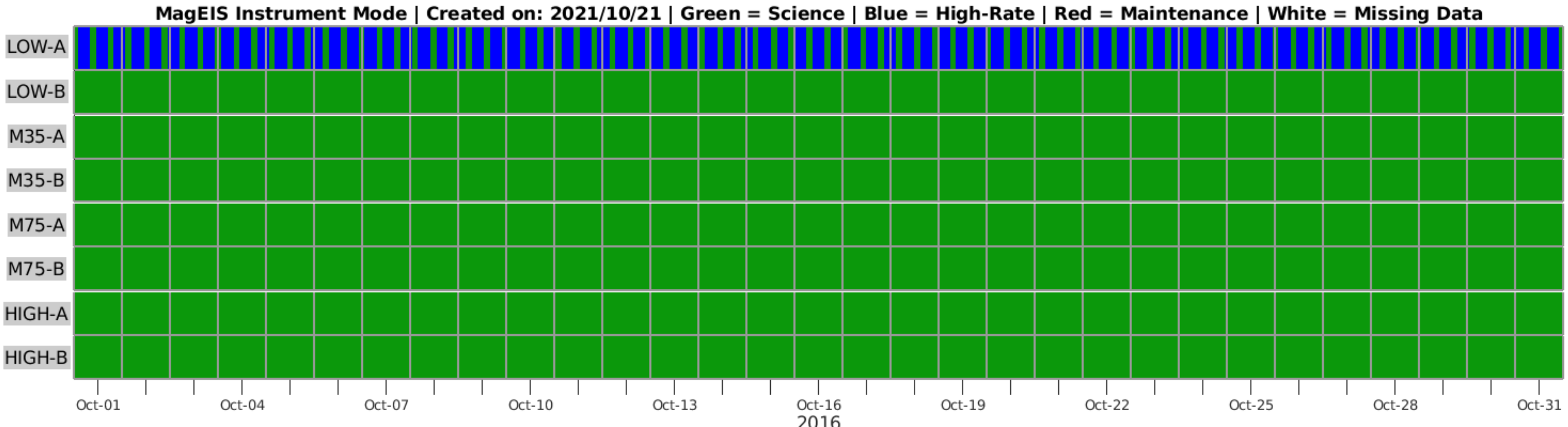
MagEIS Instrument Mode | Created on: 2021/10/21 | Green = Science | Blue = High-Rate | Red = Maintenance | White = Missing Data
LOW-A
LOW-B
M35-A
M35-B
M75-A
M75-B
HIGH-A
HIGH-B
Oct-01
Oct-04
Oct-07
Oct-10
Oct-13
Oct-16
Oct-19
Oct-22
Oct-25
Oct-28
Oct-31
2016

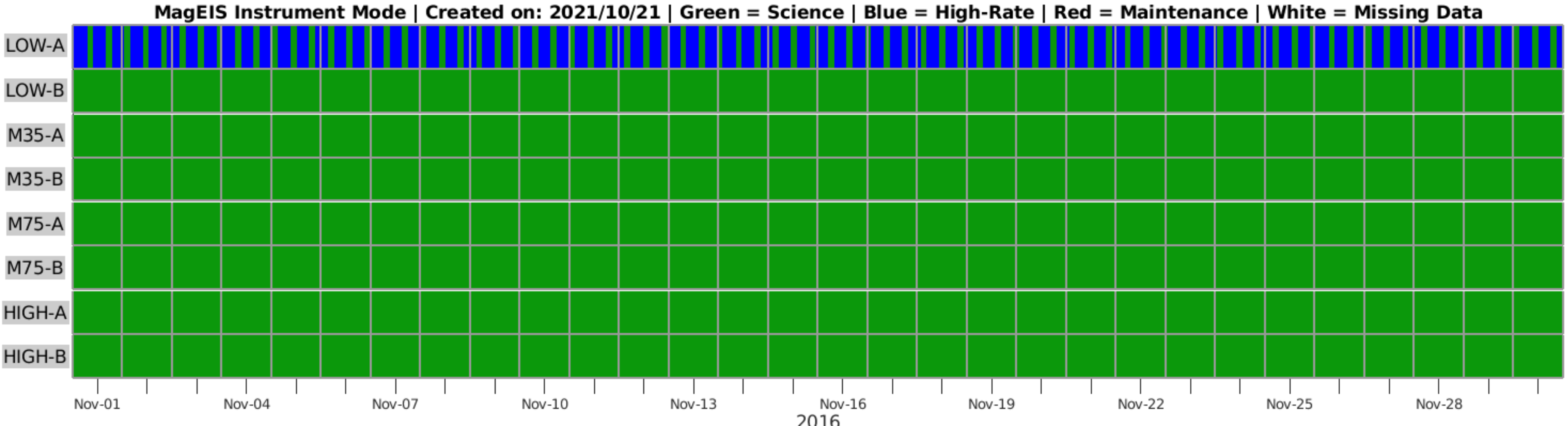
MagEIS Instrument Mode | Created on: 2021/10/21 | Green = Science | Blue = High-Rate | Red = Maintenance | White = Missing Data
LOW-A
LOW-B
M35-A
M35-B
M75-A
M75-B
HIGH-A
HIGH-B
Nov-01
Nov-04
Nov-07
Nov-10
Nov-13
Nov-16
Nov-19
Nov-22
Nov-25
Nov-28
2016

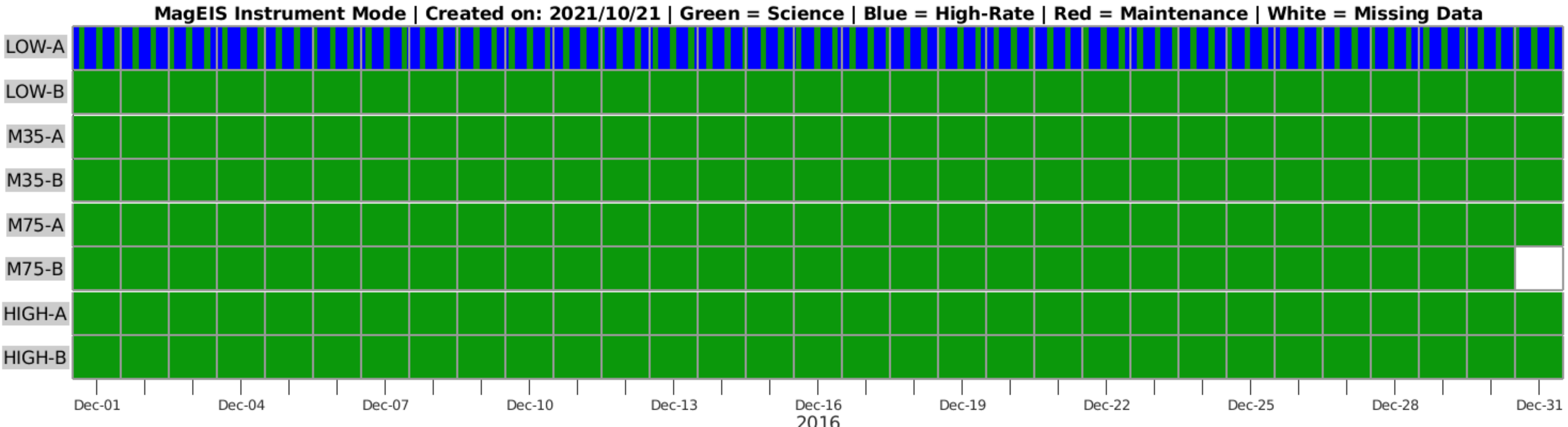
MagEIS Instrument Mode | Created on: 2021/10/21 | Green = Science | Blue = High-Rate | Red = Maintenance | White = Missing Data
LOW-A
LOW-B
M35-A
M35-B
M75-A
M75-B
HIGH-A
HIGH-B
Dec-01
Dec-04
Dec-07
Dec-10
Dec-13
Dec-16
Dec-19
Dec-22
Dec-25
Dec-28
Dec-31
2016

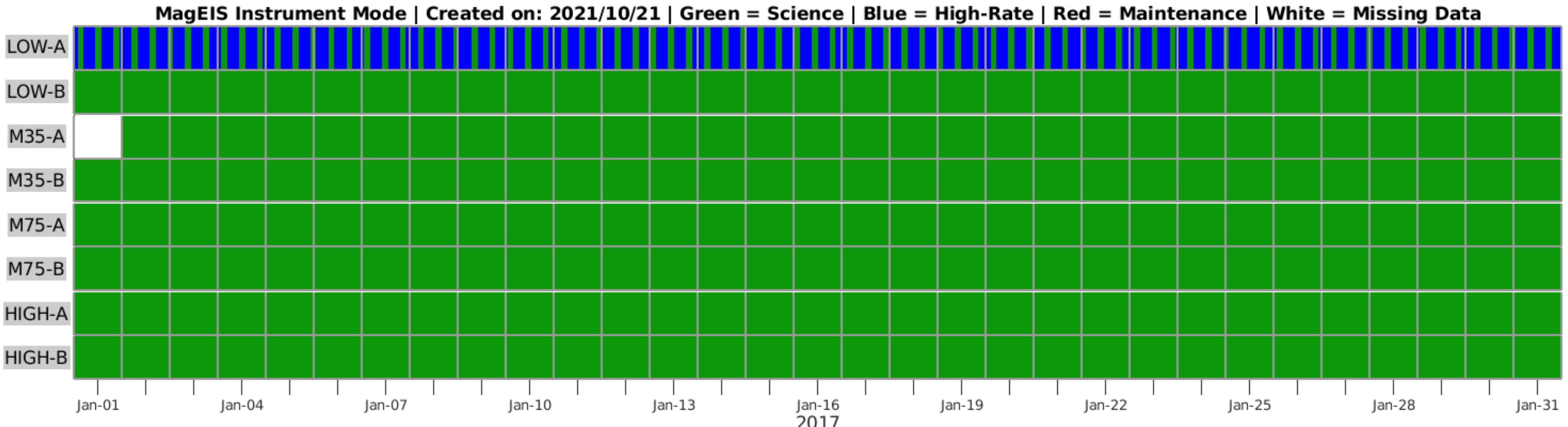
MagEIS Instrument Mode | Created on: 2021/10/21 | Green = Science | Blue = High-Rate | Red = Maintenance | White = Missing Data
LOW-A
LOW-B
M35-A
M35-B
M75-A
M75-B
HIGH-A
HIGH-B
Jan-01
Jan-04
Jan-07
Jan-10
Jan-13
Jan-16
Jan-19
Jan-22
Jan-25
Jan-28
Jan-31
2017

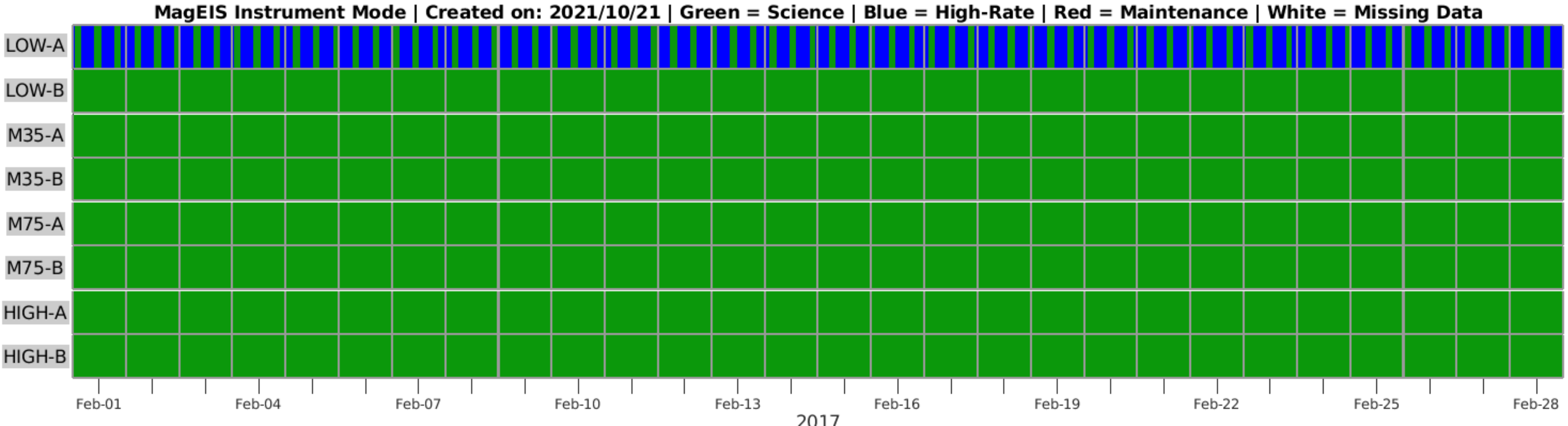
MagEIS Instrument Mode | Created on: 2021/10/21 | Green = Science | Blue = High-Rate | Red = Maintenance | White = Missing Data
LOW-A
LOW-B
M35-A
M35-B
M75-A
M75-B
HIGH-A
HIGH-B
Feb-01
Feb-04
Feb-07
Feb-10
Feb-13
Feb-16
Feb-19
Feb-22
Feb-25
Feb-28
2017

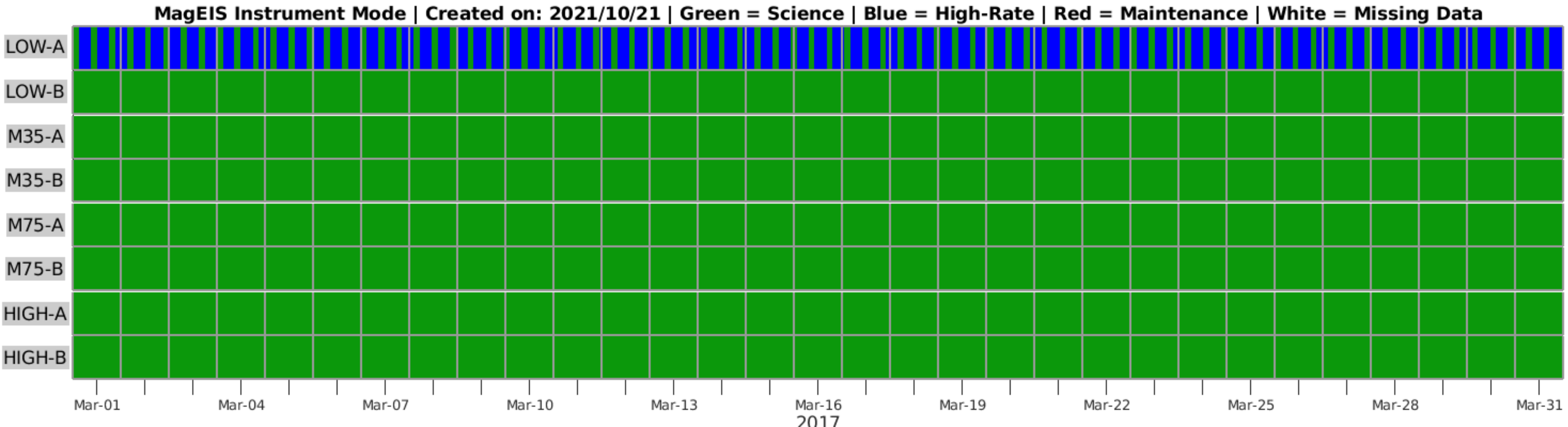
MagEIS Instrument Mode | Created on: 2021/10/21 | Green = Science | Blue = High-Rate | Red = Maintenance | White = Missing Data
LOW-A
LOW-B
M35-A
M35-B
M75-A
M75-B
HIGH-A
HIGH-B
Mar-01
Mar-04
Mar-07
Mar-10
Mar-13
Mar-16
Mar-19
Mar-22
Mar-25
Mar-28
Mar-31
2017

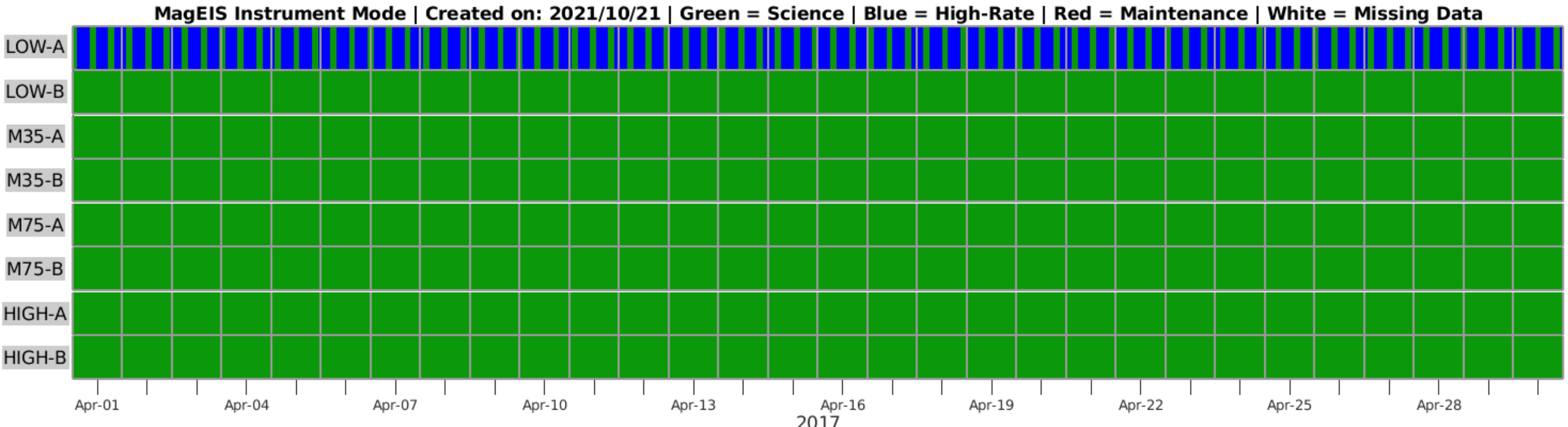
MagEIS Instrument Mode | Created on: 2021/10/21 | Green = Science | Blue = High-Rate | Red = Maintenance | White = Missing Data
LOW-A
LOW-B
M35-A
M35-B
M75-A
M75-B
HIGH-A
HIGH-B
Apr-01
Apr-04
Apr-07
Apr-10
Apr-13
Apr-16
Apr-19
Apr-22
Apr-25
Apr-28
2017

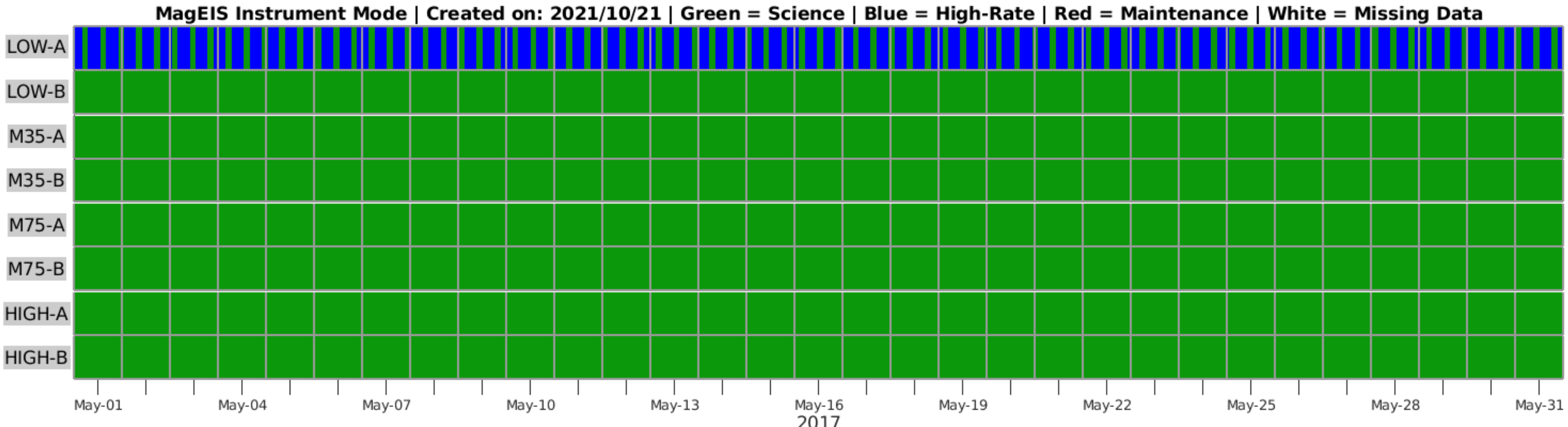
MagEIS Instrument Mode | Created on: 2021/10/21 | Green = Science | Blue = High-Rate | Red = Maintenance | White = Missing Data
LOW-A
LOW-B
M35-A
M35-B
M75-A
M75-B
HIGH-A
HIGH-B
May-01
May-04
May-07
May-10
May-13
May-16
May-19
May-22
May-25
May-28
May-31
2017

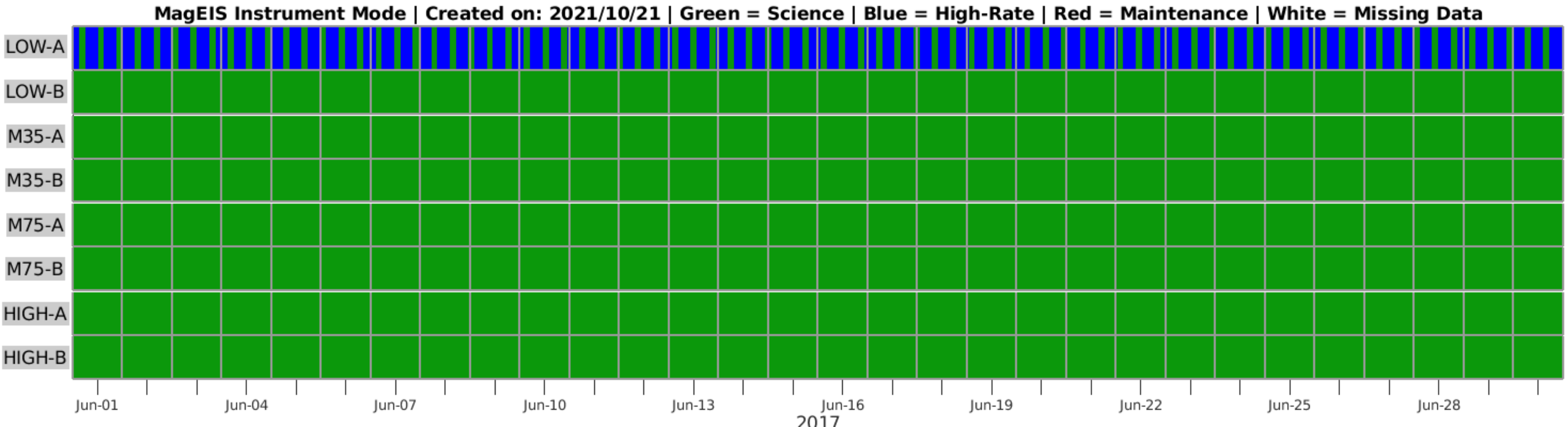
MagEIS Instrument Mode | Created on: 2021/10/21 | Green = Science | Blue = High-Rate | Red = Maintenance | White = Missing Data
LOW-A
LOW-B
M35-A
M35-B
M75-A
M75-B
HIGH-A
HIGH-B
Jun-01
Jun-04
Jun-07
Jun-10
Jun-13
Jun-16
Jun-19
Jun-22
Jun-25
Jun-28
2017

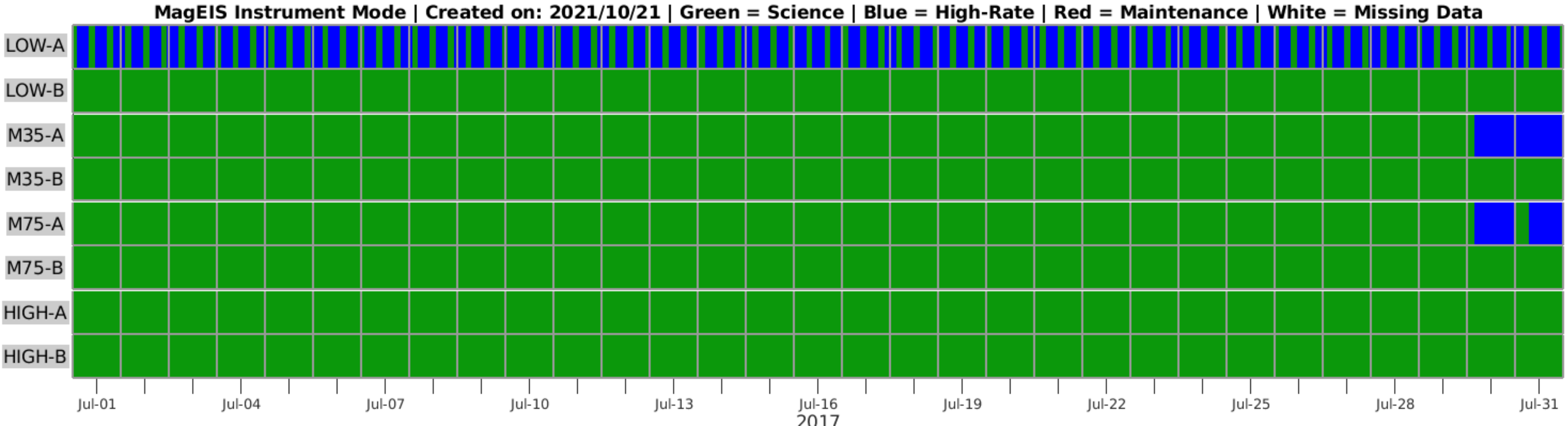
MagEIS Instrument Mode | Created on: 2021/10/21 | Green = Science | Blue = High-Rate | Red = Maintenance | White = Missing Data
LOW-A
LOW-B
M35-A
M35-B
M75-A
M75-B
HIGH-A
HIGH-B
Jul-01
Jul-04
Jul-07
Jul-10
Jul-13
Jul-16
Jul-19
Jul-22
Jul-25
Jul-28
Jul-31
2017

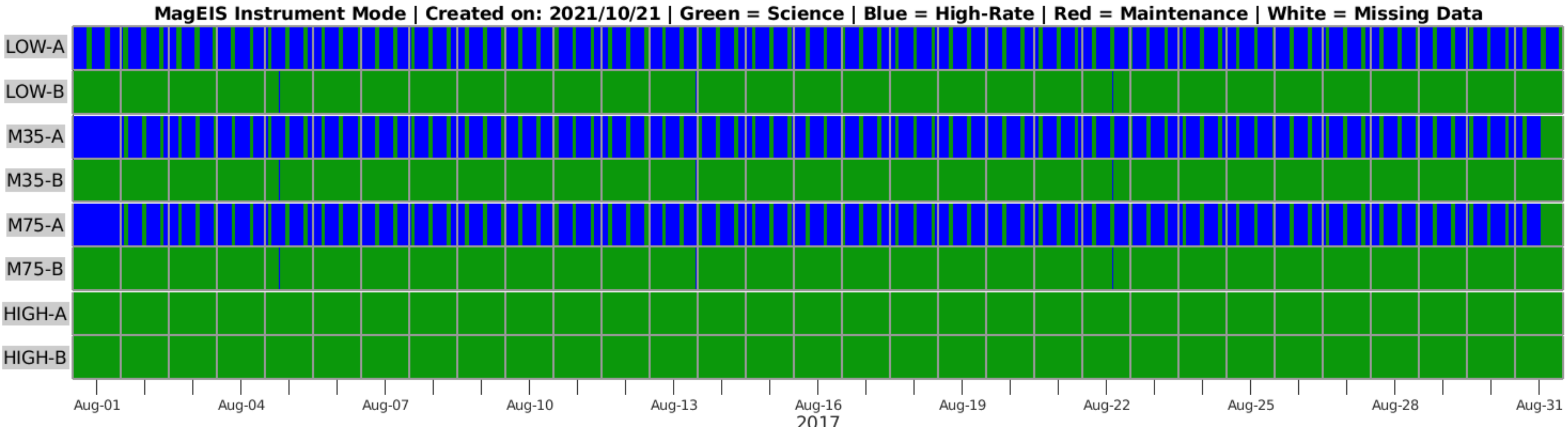
MagEIS Instrument Mode | Created on: 2021/10/21 | Green = Science | Blue = High-Rate | Red = Maintenance | White = Missing Data
LOW-A
LOW-B
M35-A
M35-B
M75-A
M75-B
HIGH-A
HIGH-B
Aug-01
Aug-04
Aug-07
Aug-10
Aug-13
Aug-16
Aug-19
Aug-22
Aug-25
Aug-28
Aug-31
2017

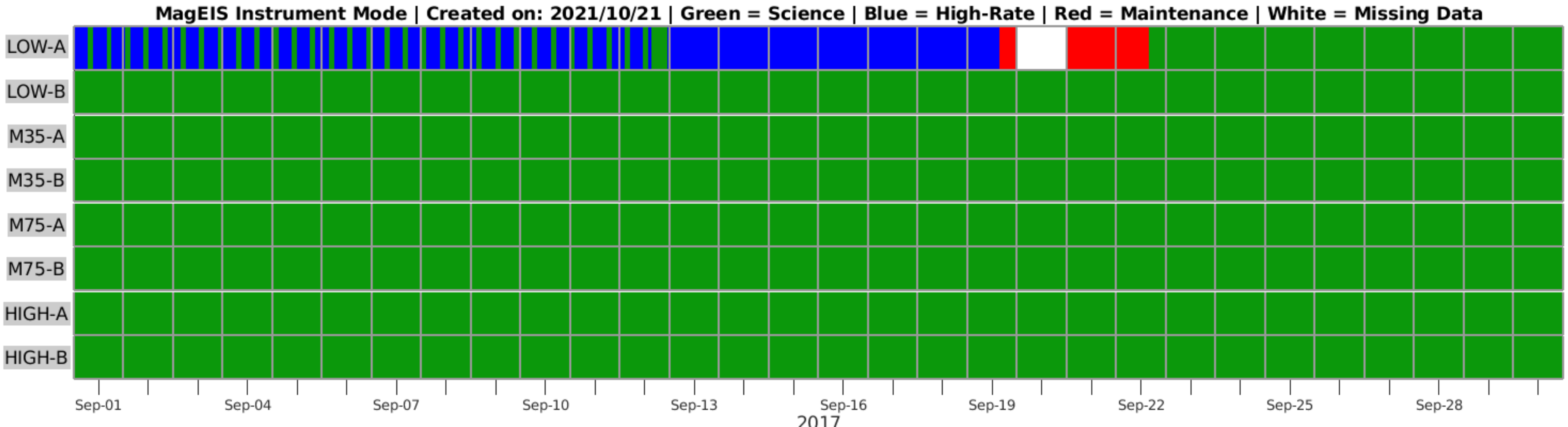

MagEIS Instrument Mode | Created on: 2021/10/21 | Green = Science | Blue = High-Rate | Red = Maintenance | White = Missing Data
LOW-A
LOW-B
M35-A
M35-B
M75-A
M75-B
HIGH-A
HIGH-B
Sep-01
Sep-04
Sep-07
Sep-10
Sep-13
Sep-16
Sep-19
Sep-22
Sep-25
Sep-28
2017

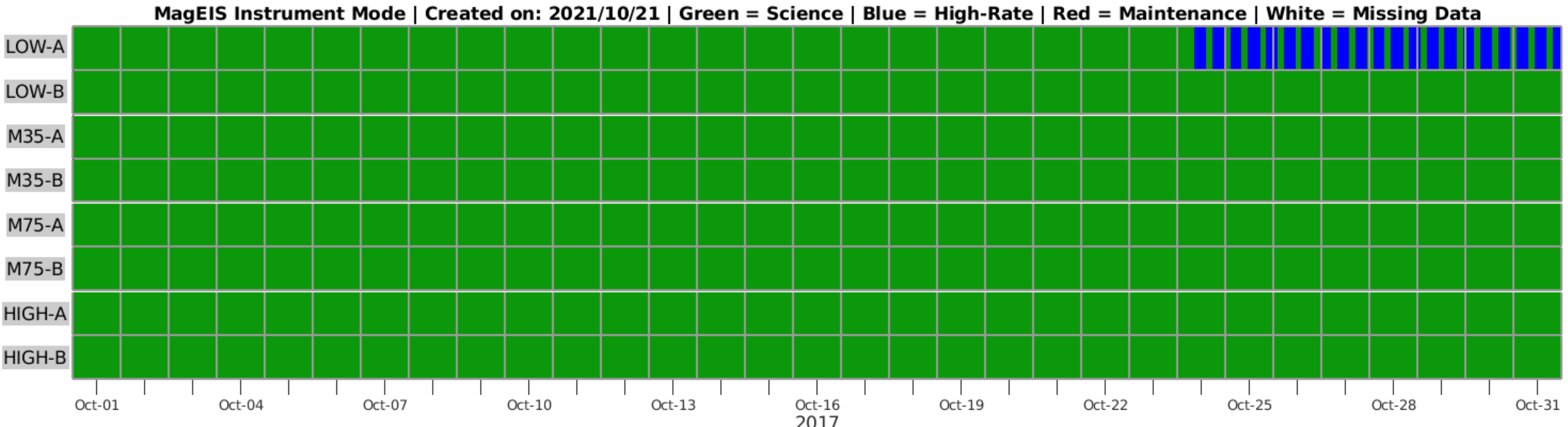
MagEIS Instrument Mode | Created on: 2021/10/21 | Green = Science | Blue = High-Rate | Red = Maintenance | White = Missing Data
LOW-A
LOW-B
M35-A
M35-B
M75-A
M75-B
HIGH-A
HIGH-B
Oct-01
Oct-04
Oct-07
Oct-10
Oct-13
Oct-16
Oct-19
Oct-22
Oct-25
Oct-28
Oct-31
2017

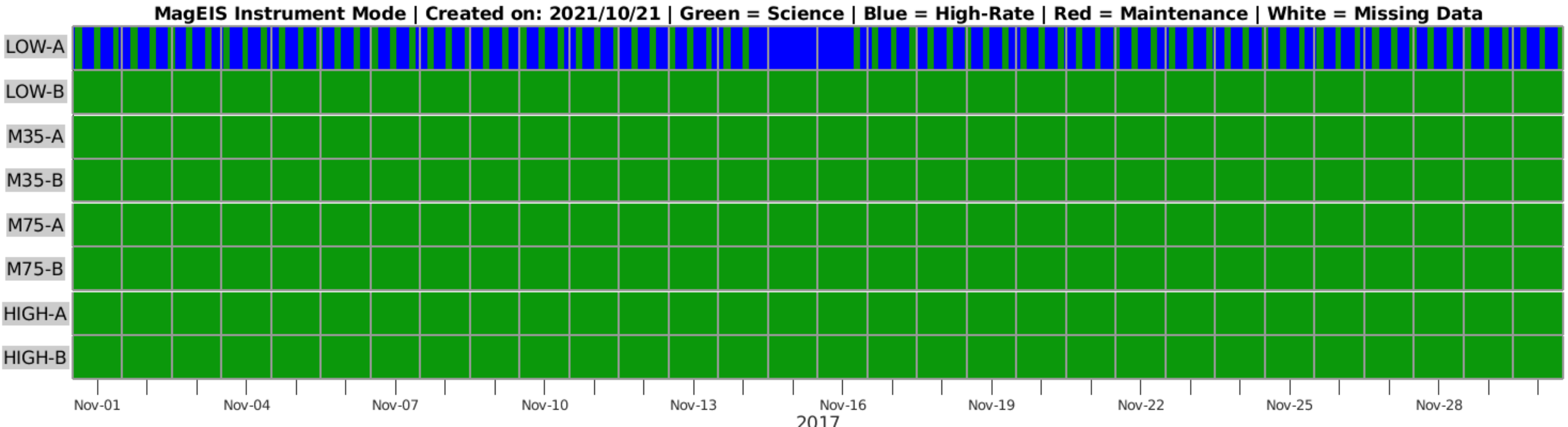
MagEIS Instrument Mode | Created on: 2021/10/21 | Green = Science | Blue = High-Rate | Red = Maintenance | White = Missing Data
LOW-A
LOW-B
M35-A
M35-B
M75-A
M75-B
HIGH-A
HIGH-B
Nov-01
Nov-04
Nov-07
Nov-10
Nov-13
Nov-16
Nov-19
Nov-22
Nov-25
Nov-28
2017

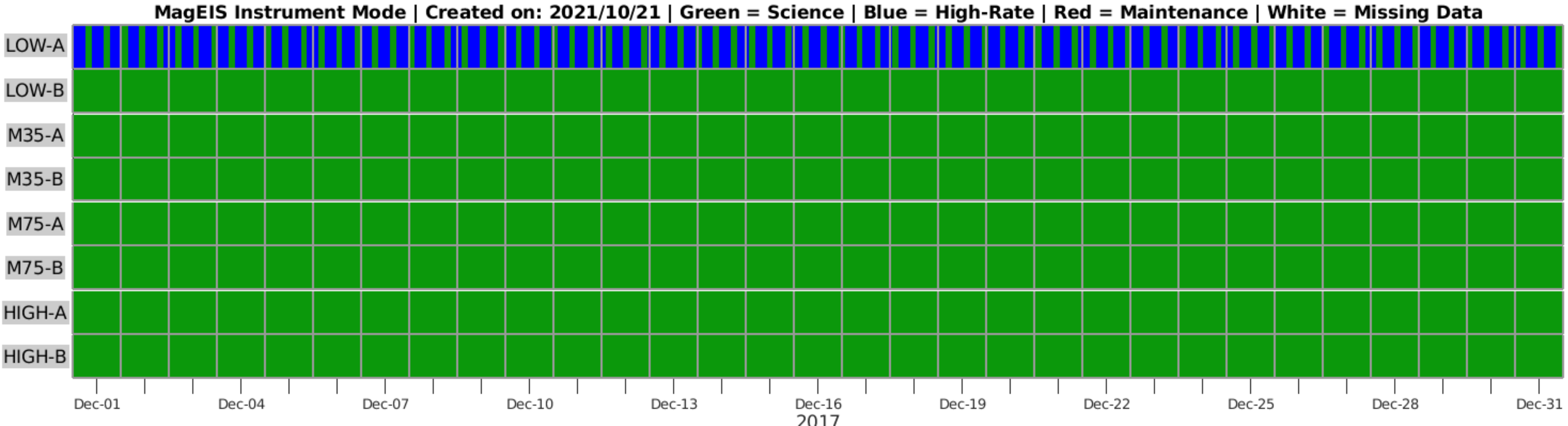
MagEIS Instrument Mode | Created on: 2021/10/21 | Green = Science | Blue = High-Rate | Red = Maintenance | White = Missing Data
LOW-A
LOW-B
M35-A
M35-B
M75-A
M75-B
HIGH-A
HIGH-B
Dec-01
Dec-04
Dec-07
Dec-10
Dec-13
Dec-16
Dec-19
Dec-22
Dec-25
Dec-28
Dec-31
2017

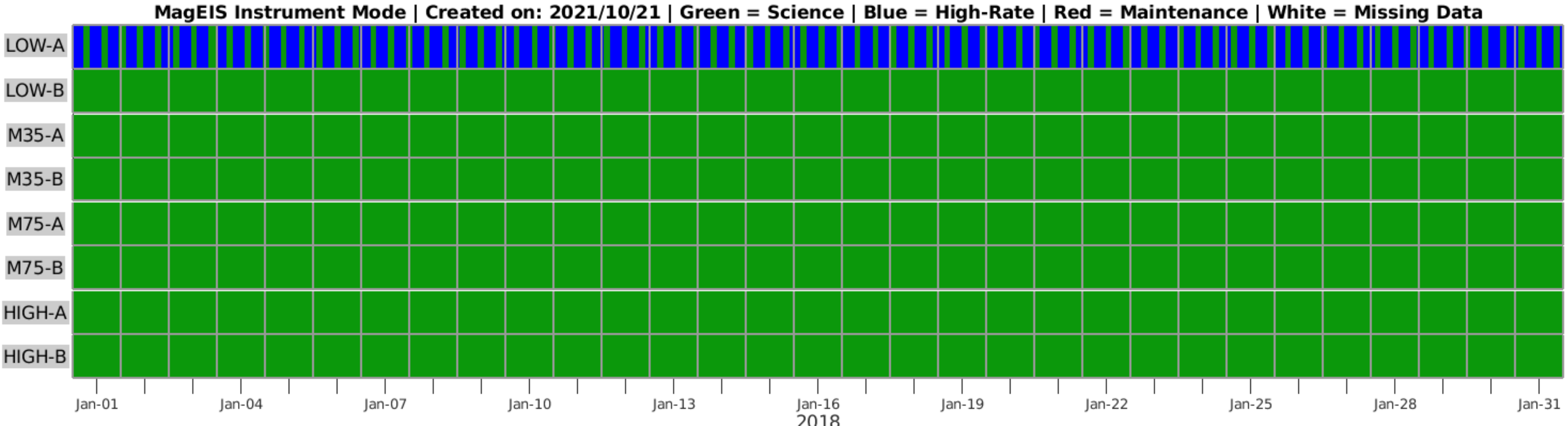
MagEIS Instrument Mode | Created on: 2021/10/21 | Green = Science | Blue = High-Rate | Red = Maintenance | White = Missing Data
LOW-A
LOW-B
M35-A
M35-B
M75-A
M75-B
HIGH-A
HIGH-B
Jan-01
Jan-04
Jan-07
Jan-10
Jan-13
Jan-16
Jan-19
Jan-22
Jan-25
Jan-28
Jan-31
2018

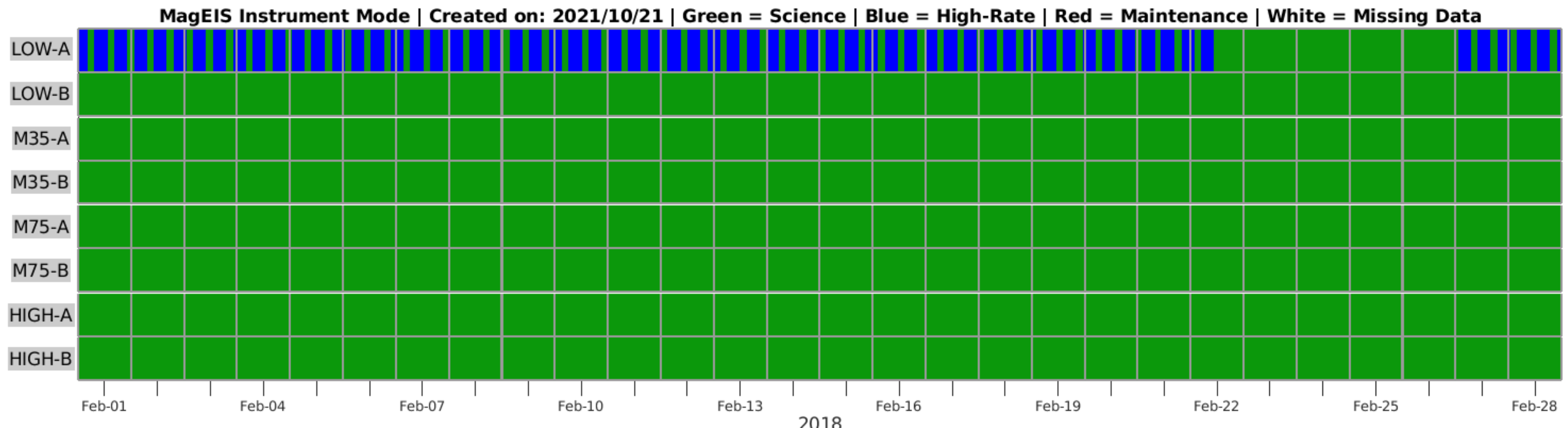

MagEIS Instrument Mode | Created on: 2021/10/21 | Green = Science | Blue = High-Rate | Red = Maintenance | White = Missing Data
LOW-A
LOW-B
M35-A
M35-B
M75-A
M75-B
HIGH-A
HIGH-B
Feb-01
Feb-04
Feb-07
Feb-10
Feb-13
Feb-16
Feb-19
Feb-22
Feb-25
Feb-28
2018

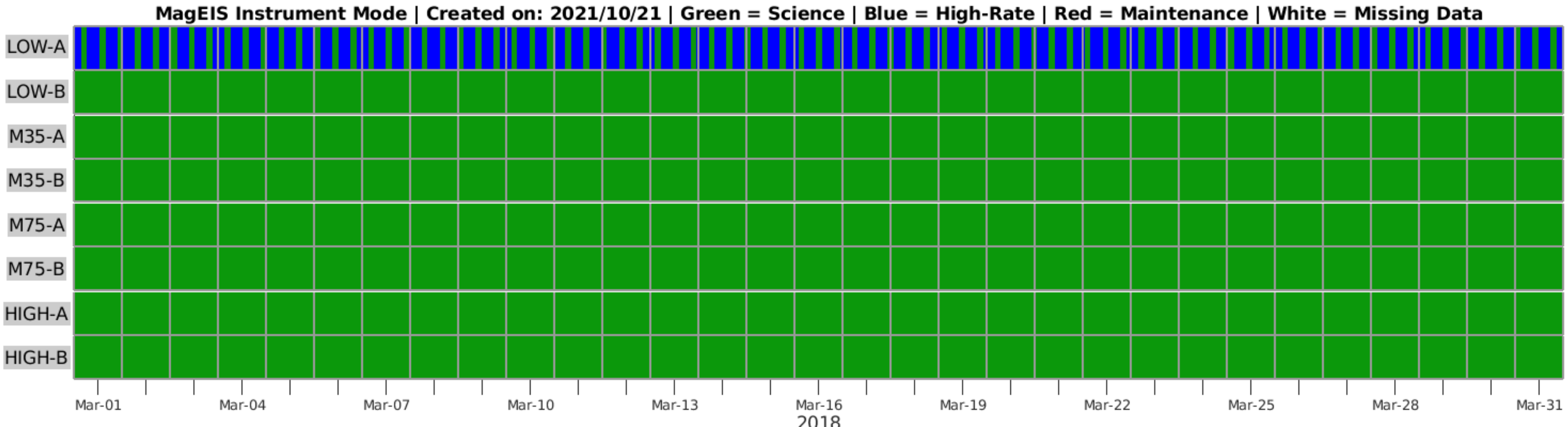
MagEIS Instrument Mode | Created on: 2021/10/21 | Green = Science | Blue = High-Rate | Red = Maintenance | White = Missing Data
LOW-A
LOW-B
M35-A
M35-B
M75-A
M75-B
HIGH-A
HIGH-B
Mar-01
Mar-04
Mar-07
Mar-10
Mar-13
Mar-16
Mar-19
Mar-22
Mar-25
Mar-28
Mar-31
2018

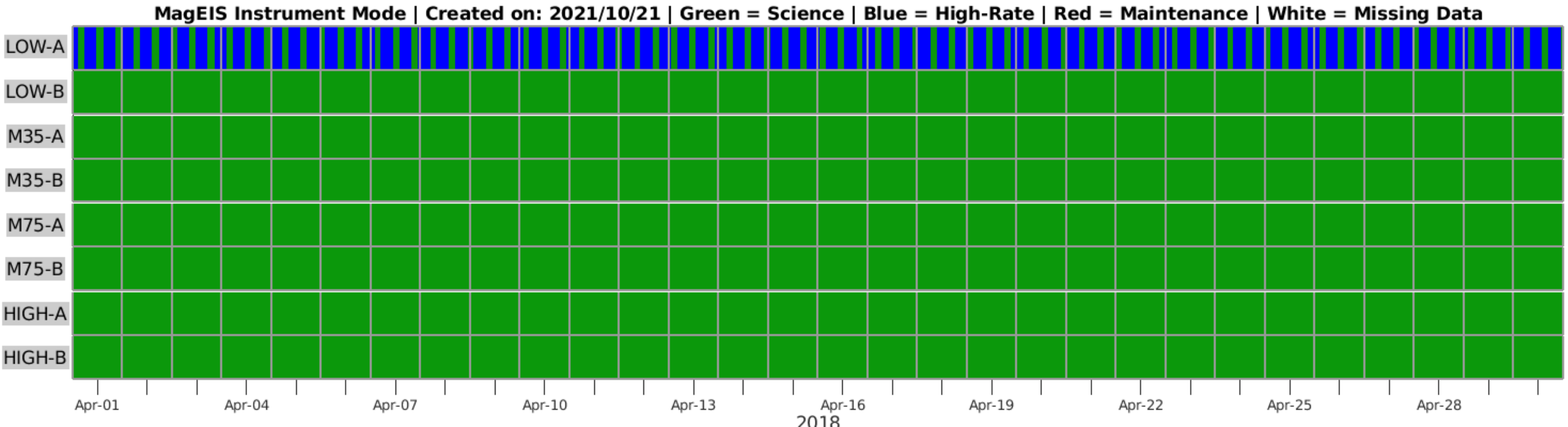

MagEIS Instrument Mode | Created on: 2021/10/21 | Green = Science | Blue = High-Rate | Red = Maintenance | White = Missing Data
LOW-A
LOW-B
M35-A
M35-B
M75-A
M75-B
HIGH-A
HIGH-B
Apr-01
Apr-04
Apr-07
Apr-10
Apr-13
Apr-16
Apr-19
Apr-22
Apr-25
Apr-28
2018

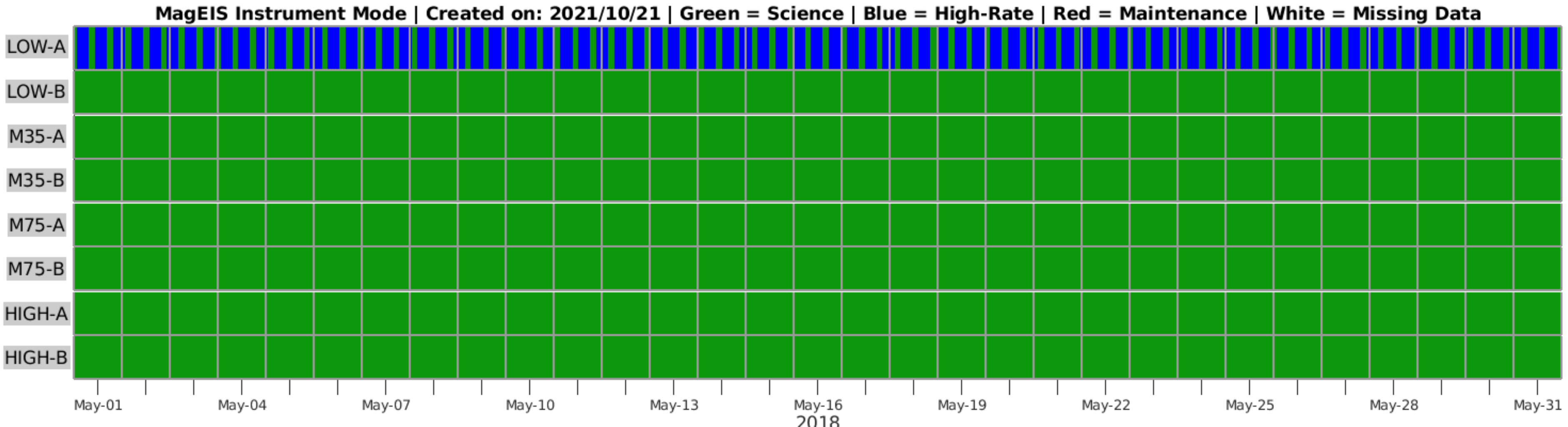
MagEIS Instrument Mode | Created on: 2021/10/21 | Green = Science | Blue = High-Rate | Red = Maintenance | White = Missing Data
LOW-A
LOW-B
M35-A
M35-B
M75-A
M75-B
HIGH-A
HIGH-B
May-01
May-04
May-07
May-10
May-13
May-16
May-19
May-22
May-25
May-28
May-31
2018

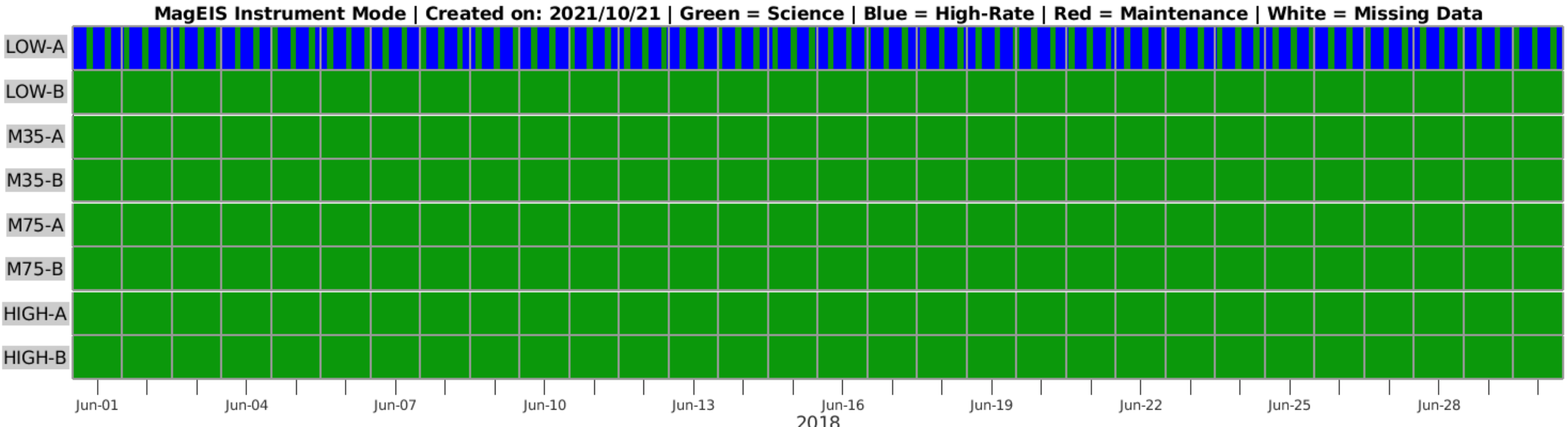
MagEIS Instrument Mode | Created on: 2021/10/21 | Green = Science | Blue = High-Rate | Red = Maintenance | White = Missing Data
LOW-A
LOW-B
M35-A
M35-B
M75-A
M75-B
HIGH-A
HIGH-B
Jun-01
Jun-04
Jun-07
Jun-10
Jun-13
Jun-16
Jun-19
Jun-22
Jun-25
Jun-28
2018

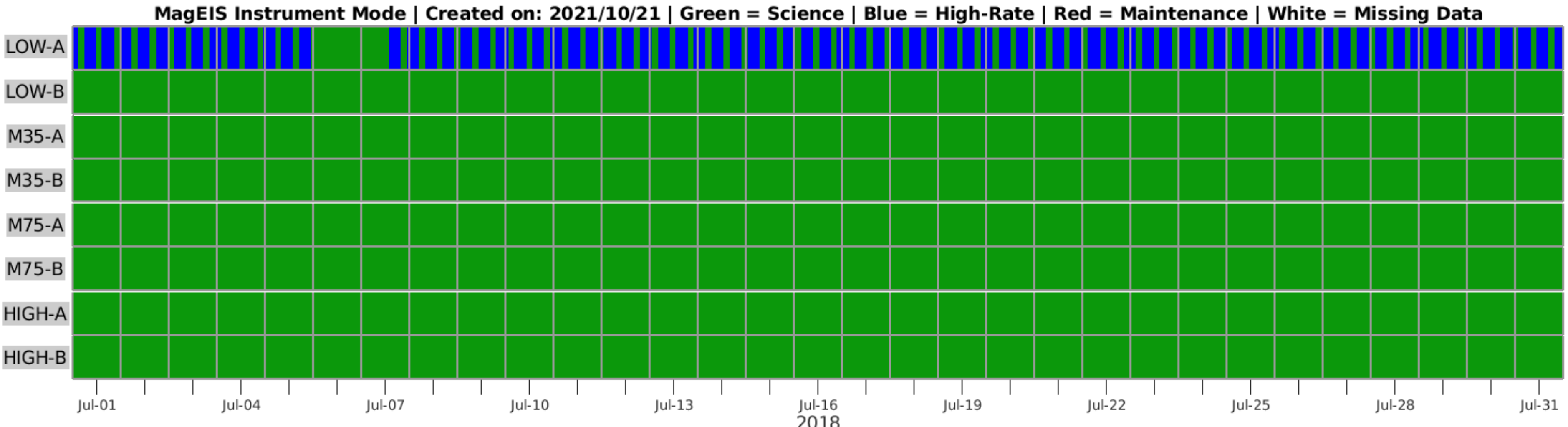
MagEIS Instrument Mode | Created on: 2021/10/21 | Green = Science | Blue = High-Rate | Red = Maintenance | White = Missing Data
LOW-A
LOW-B
M35-A
M35-B
M75-A
M75-B
HIGH-A
HIGH-B
Jul-01
Jul-04
Jul-07
Jul-10
Jul-13
Jul-16
Jul-19
Jul-22
Jul-25
Jul-28
Jul-31
2018

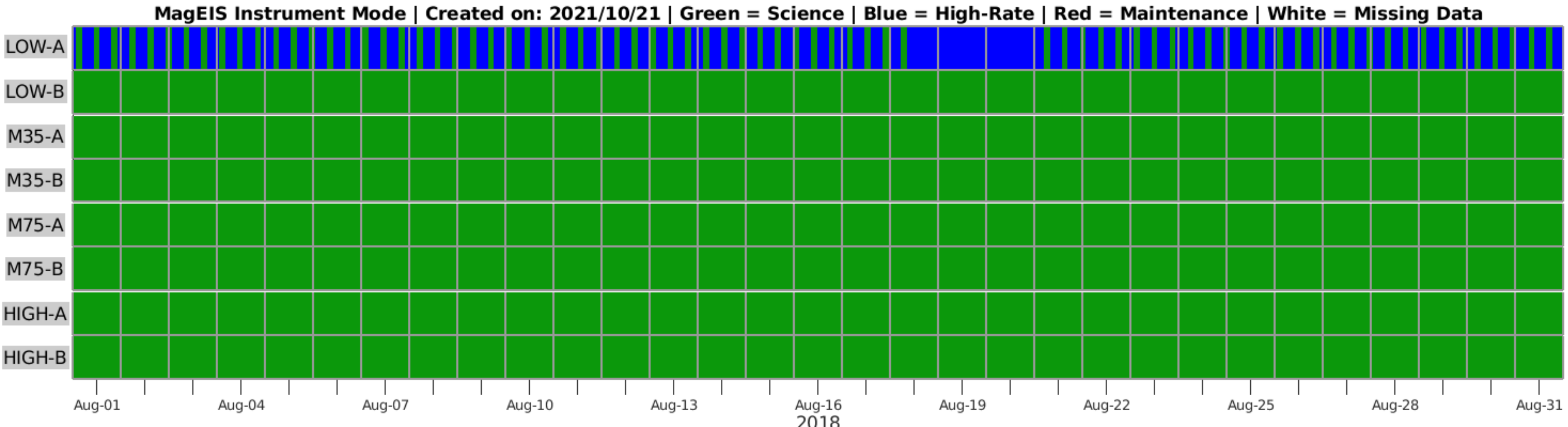
MagEIS Instrument Mode | Created on: 2021/10/21 | Green = Science | Blue = High-Rate | Red = Maintenance | White = Missing Data
LOW-A
LOW-B
M35-A
M35-B
M75-A
M75-B
HIGH-A
HIGH-B
Aug-01
Aug-04
Aug-07
Aug-10
Aug-13
Aug-16
Aug-19
Aug-22
Aug-25
Aug-28
Aug-31
2018

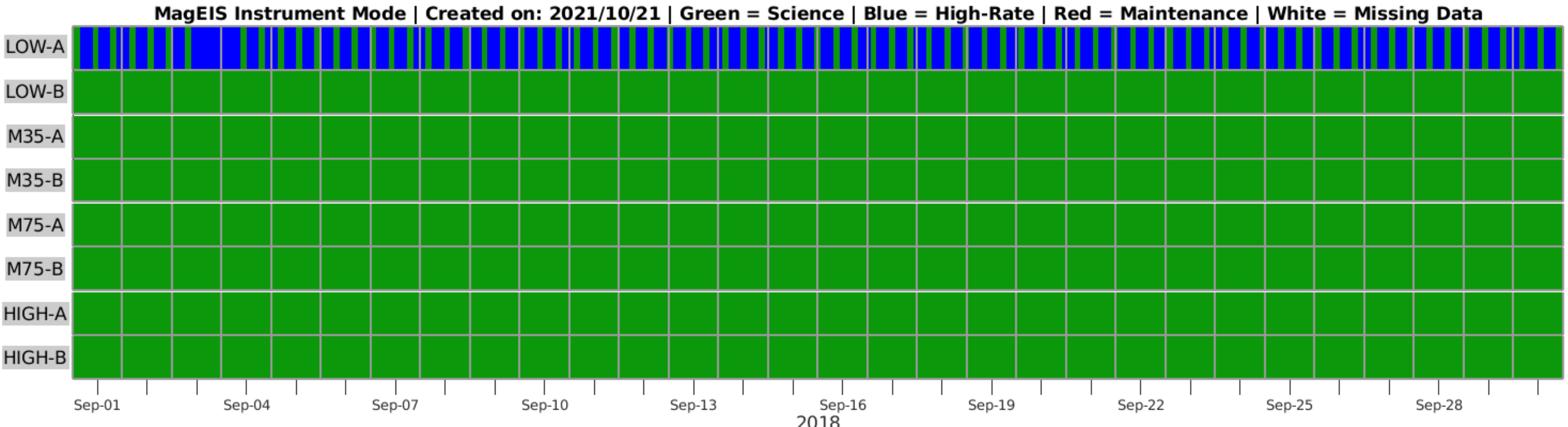
MagEIS Instrument Mode | Created on: 2021/10/21 | Green = Science | Blue = High-Rate | Red = Maintenance | White = Missing Data
LOW-A
LOW-B
M35-A
M35-B
M75-A
M75-B
HIGH-A
HIGH-B
Sep-01
Sep-04
Sep-07
Sep-10
Sep-13
Sep-16
Sep-19
Sep-22
Sep-25
Sep-28
2018

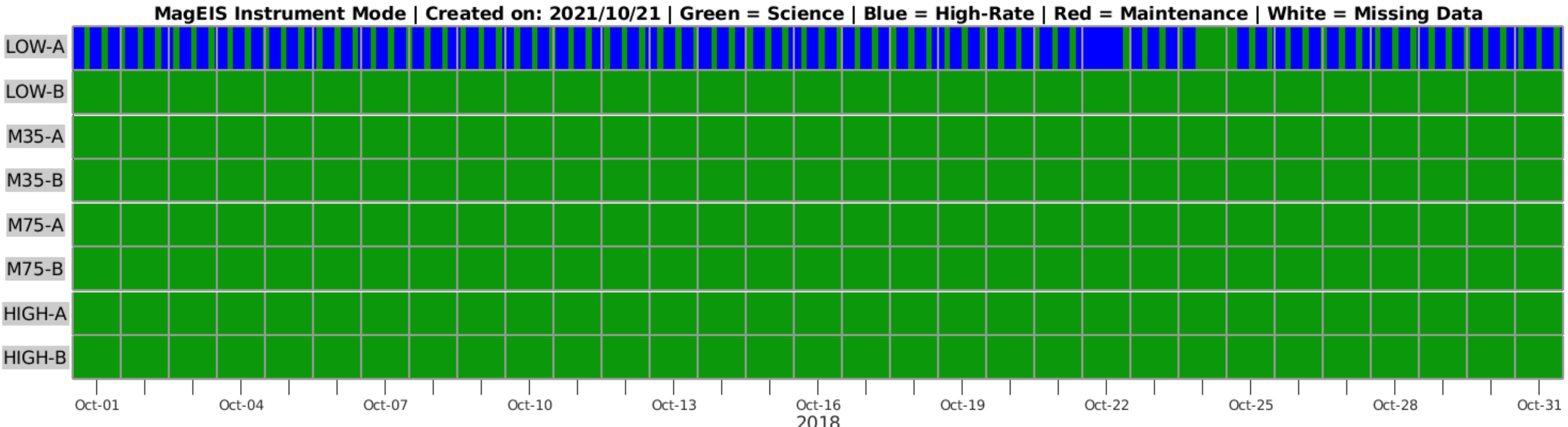
MagEIS Instrument Mode | Created on: 2021/10/21 | Green = Science | Blue = High-Rate | Red = Maintenance | White = Missing Data
LOW-A
LOW-B
M35-A
M35-B
M75-A
M75-B
HIGH-A
HIGH-B
Oct-01
Oct-04
Oct-07
Oct-10
Oct-13
Oct-16
Oct-19
Oct-22
Oct-25
Oct-28
Oct-31
2018

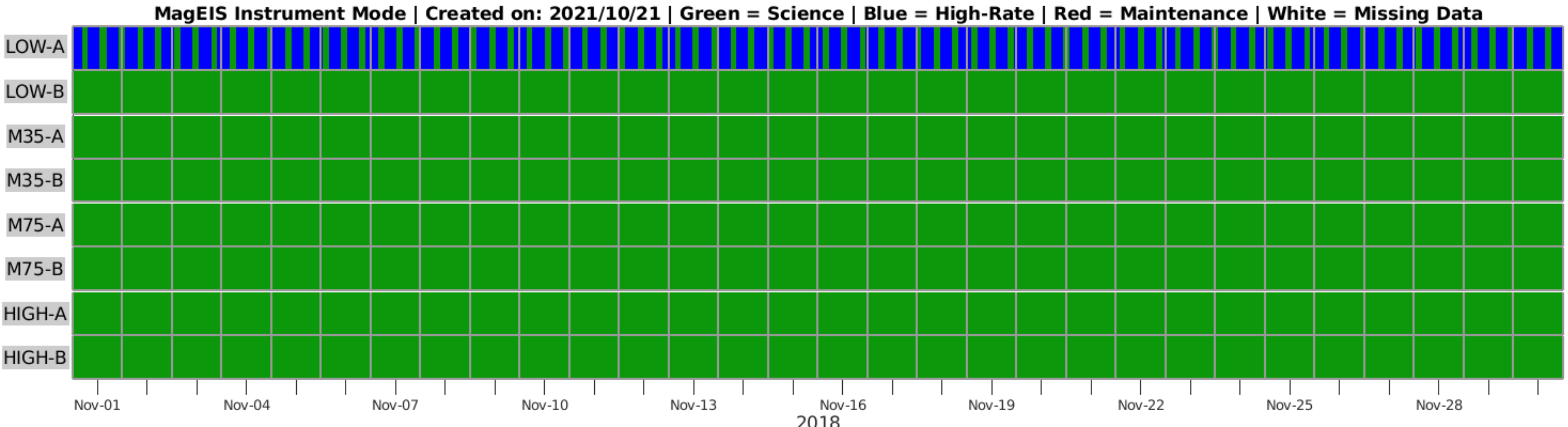
MagEIS Instrument Mode | Created on: 2021/10/21 | Green = Science | Blue = High-Rate | Red = Maintenance | White = Missing Data
LOW-A
LOW-B
M35-A
M35-B
M75-A
M75-B
HIGH-A
HIGH-B
Nov-01
Nov-04
Nov-07
Nov-10
Nov-13
Nov-16
Nov-19
Nov-22
Nov-25
Nov-28
2018

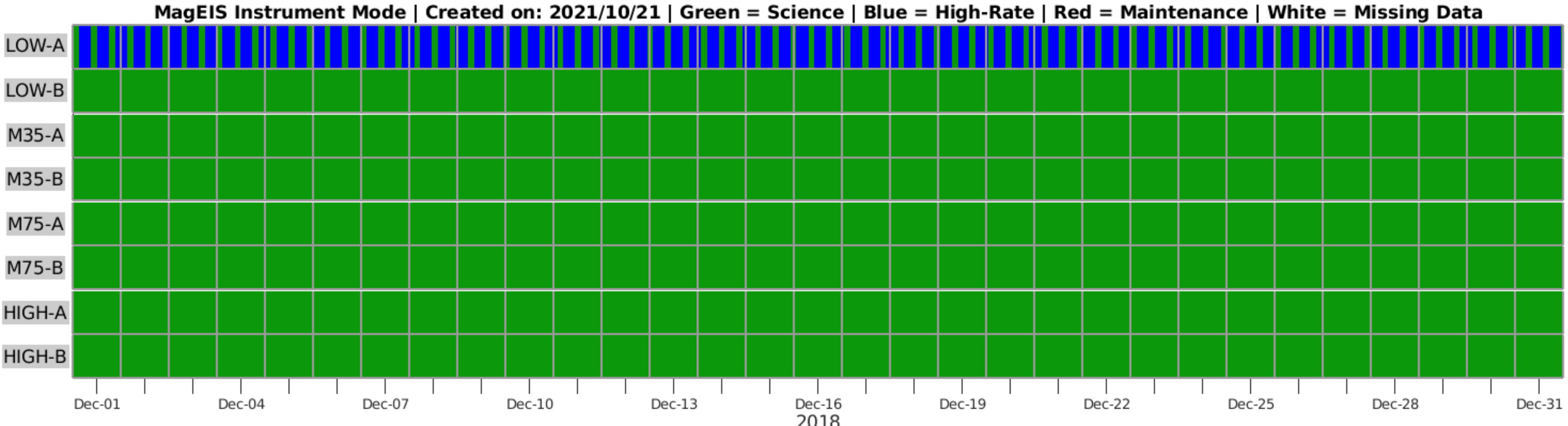
MagEIS Instrument Mode | Created on: 2021/10/21 | Green = Science | Blue = High-Rate | Red = Maintenance | White = Missing Data
LOW-A
LOW-B
M35-A
M35-B
M75-A
M75-B
HIGH-A
HIGH-B
Dec-01
Dec-04
Dec-07
Dec-10
Dec-13
Dec-16
Dec-19
Dec-22
Dec-25
Dec-28
Dec-31
2018

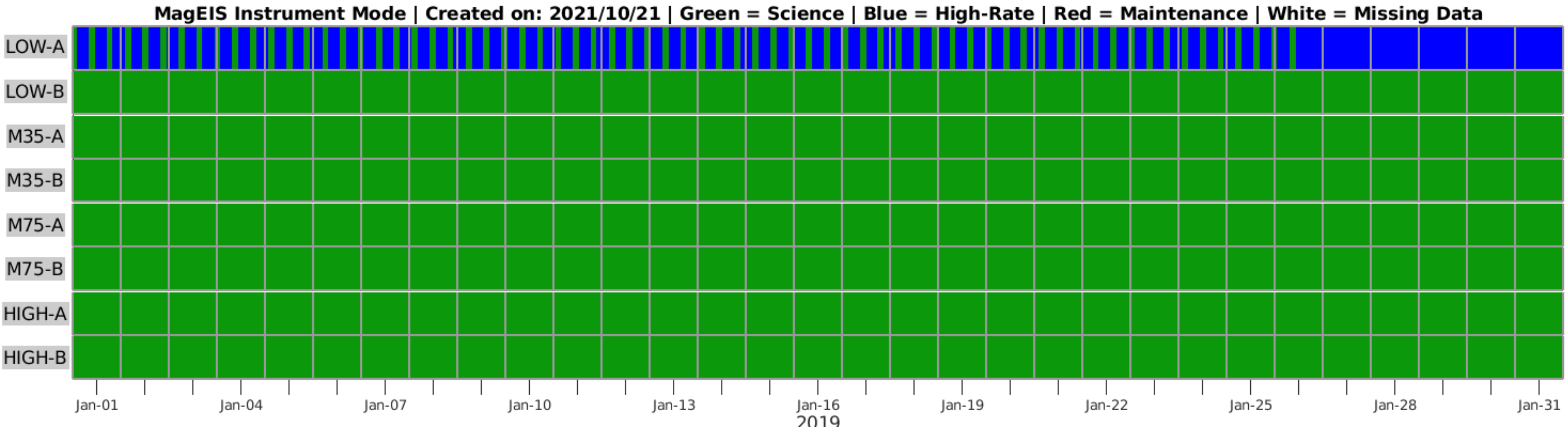
MagEIS Instrument Mode | Created on: 2021/10/21 | Green = Science | Blue = High-Rate | Red = Maintenance | White = Missing Data
LOW-A
LOW-B
M35-A
M35-B
M75-A
M75-B
HIGH-A
HIGH-B
Jan-01
Jan-04
Jan-07
Jan-10
Jan-13
Jan-16
Jan-19
Jan-22
Jan-25
Jan-28
Jan-31
2019

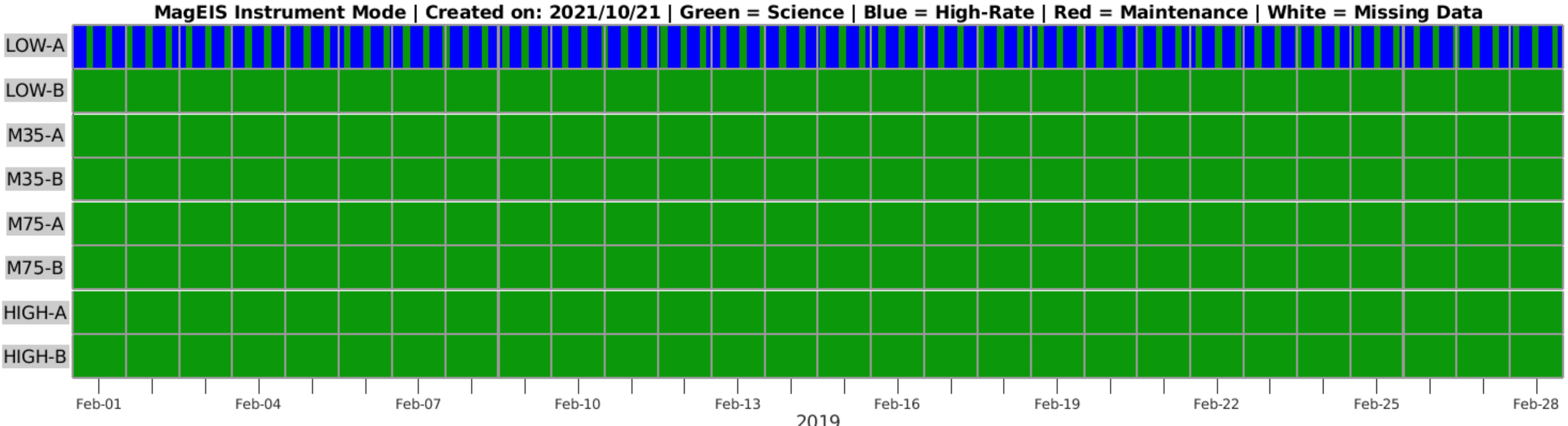
MagEIS Instrument Mode | Created on: 2021/10/21 | Green = Science | Blue = High-Rate | Red = Maintenance | White = Missing Data
LOW-A
LOW-B
M35-A
M35-B
M75-A
M75-B
HIGH-A
HIGH-B
Feb-01
Feb-04
Feb-07
Feb-10
Feb-13
Feb-16
Feb-19
Feb-22
Feb-25
Feb-28
2019

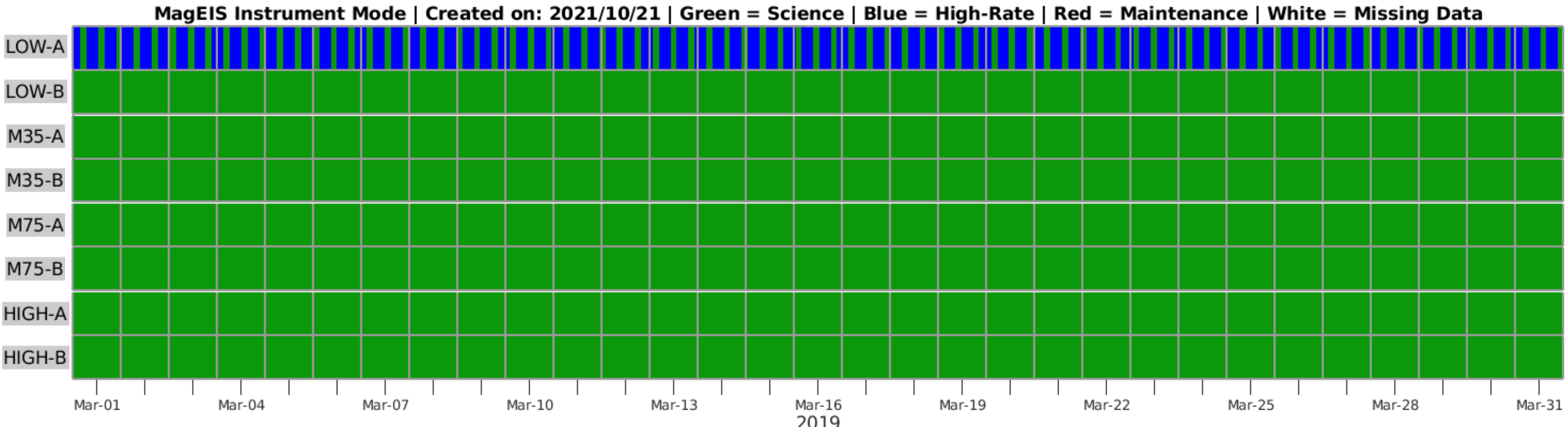
MagEIS Instrument Mode | Created on: 2021/10/21 | Green = Science | Blue = High-Rate | Red = Maintenance | White = Missing Data
LOW-A
LOW-B
M35-A
M35-B
M75-A
M75-B
HIGH-A
HIGH-B
Mar-01
Mar-04
Mar-07
Mar-10
Mar-13
Mar-16
Mar-19
Mar-22
Mar-25
Mar-28
Mar-31
2019

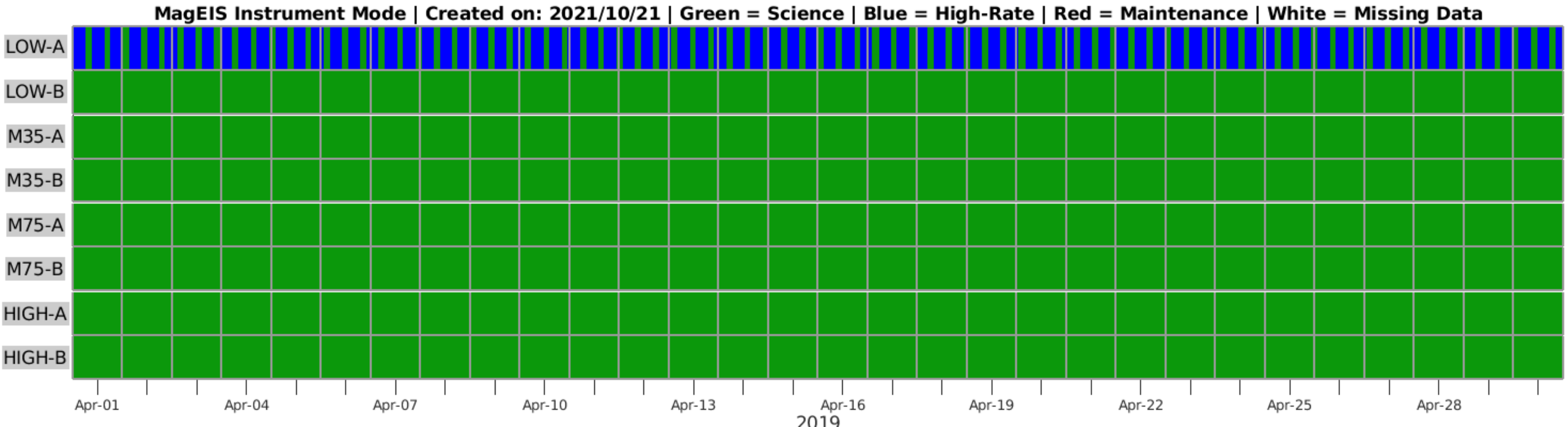
MagEIS Instrument Mode | Created on: 2021/10/21 | Green = Science | Blue = High-Rate | Red = Maintenance | White = Missing Data
LOW-A
LOW-B
M35-A
M35-B
M75-A
M75-B
HIGH-A
HIGH-B
Apr-01
Apr-04
Apr-07
Apr-10
Apr-13
Apr-16
Apr-19
Apr-22
Apr-25
Apr-28
2019

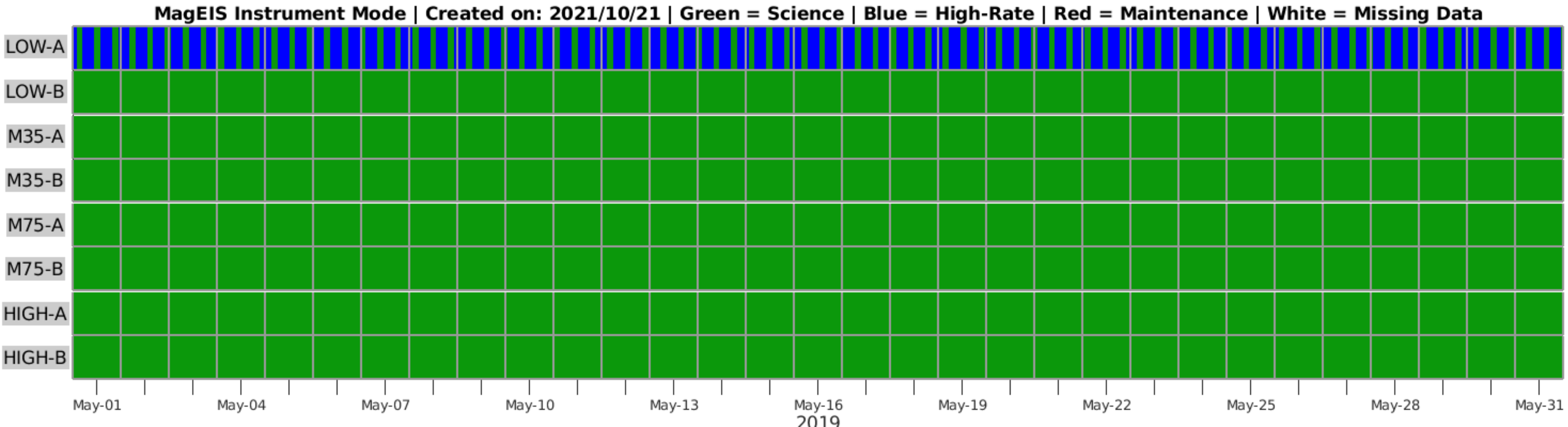
MagEIS Instrument Mode | Created on: 2021/10/21 | Green = Science | Blue = High-Rate | Red = Maintenance | White = Missing Data
LOW-A
LOW-B
M35-A
M35-B
M75-A
M75-B
HIGH-A
HIGH-B
May-01
May-04
May-07
May-10
May-13
May-16
May-19
May-22
May-25
May-28
May-31
2019

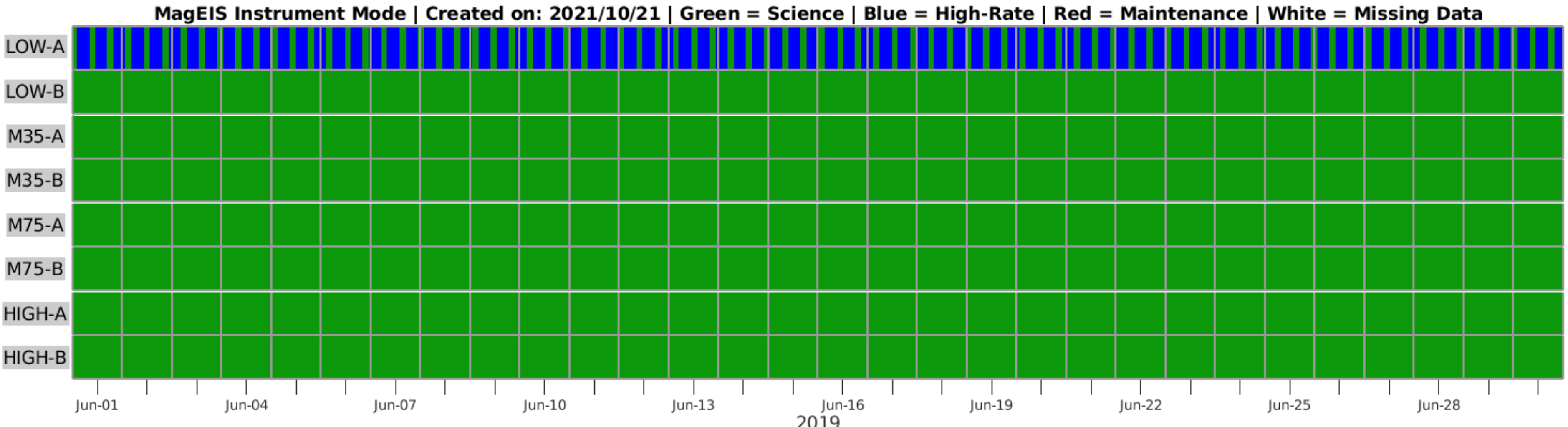
MagEIS Instrument Mode | Created on: 2021/10/21 | Green = Science | Blue = High-Rate | Red = Maintenance | White = Missing Data
LOW-A
LOW-B
M35-A
M35-B
M75-A
M75-B
HIGH-A
HIGH-B
Jun-01
Jun-04
Jun-07
Jun-10
Jun-13
Jun-16
Jun-19
Jun-22
Jun-25
Jun-28
2019

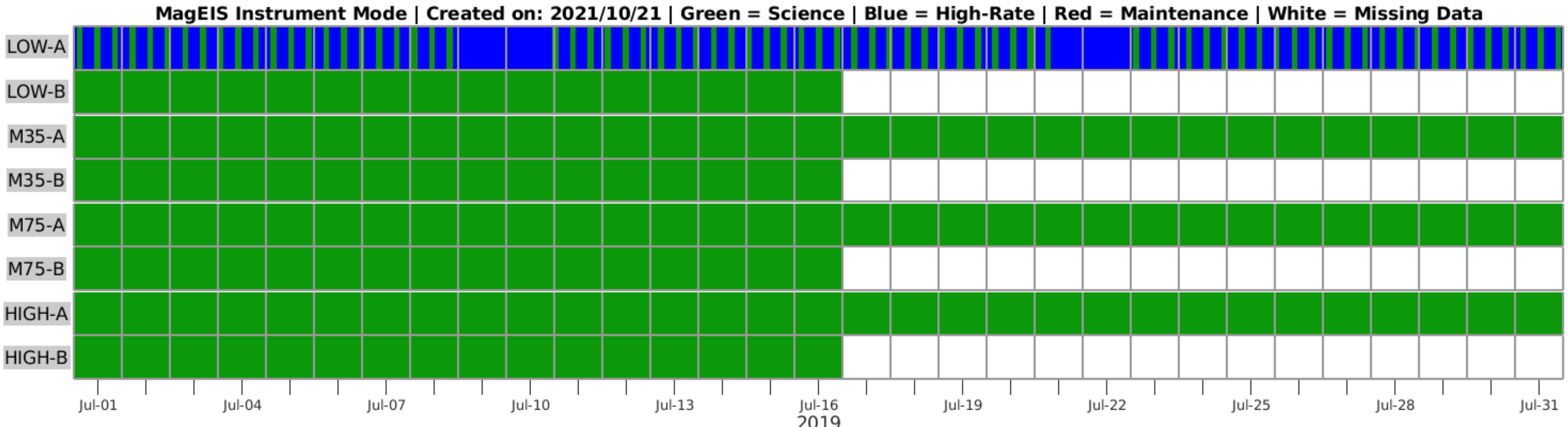
MagEIS Instrument Mode | Created on: 2021/10/21 | Green = Science | Blue = High-Rate | Red = Maintenance | White = Missing Data
LOW-A
LOW-B
M35-A
M35-B
M75-A
M75-B
HIGH-A
HIGH-B
Jul-01
Jul-04
Jul-07
Jul-10
Jul-13
Jul-16
Jul-19
Jul-22
Jul-25
Jul-28
Jul-31
2019

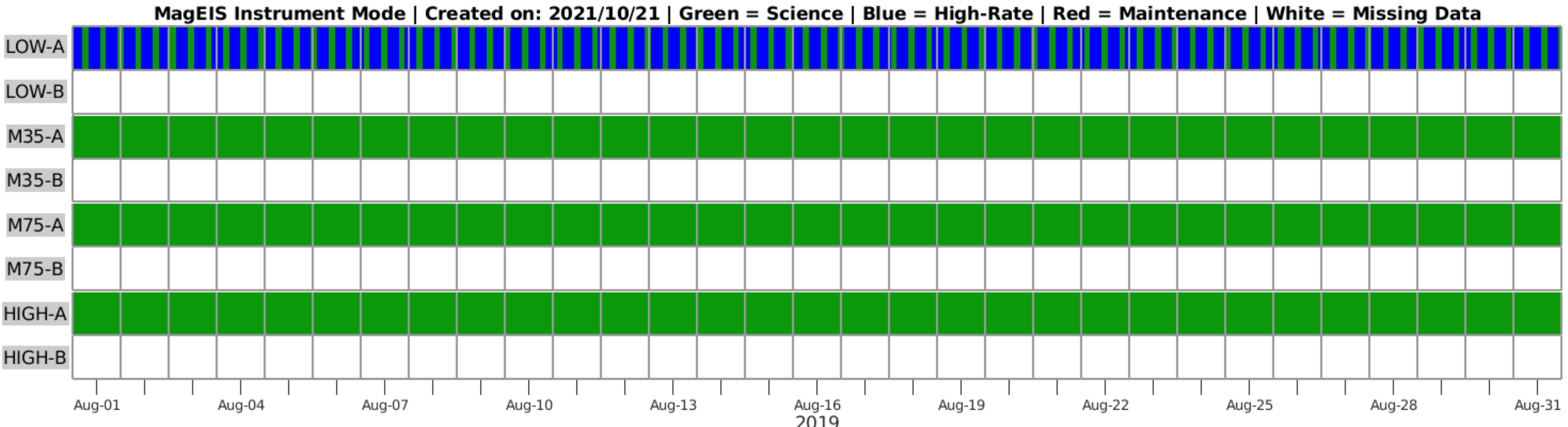
MagEIS Instrument Mode | Created on: 2021/10/21 | Green = Science | Blue = High-Rate | Red = Maintenance | White = Missing Data
LOW-A
LOW-B
M35-A
M35-B
M75-A
M75-B
HIGH-A
HIGH-B
Aug-01
Aug-04
Aug-07
Aug-10
Aug-13
Aug-16
Aug-19
Aug-22
Aug-25
Aug-28
Aug-31
2019

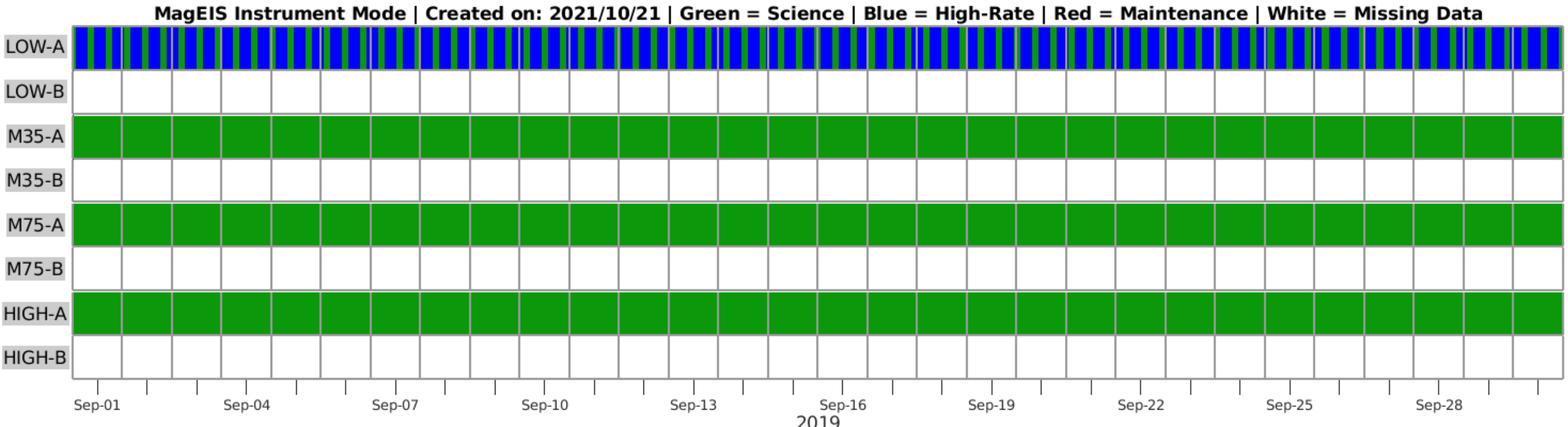

MagEIS Instrument Mode | Created on: 2021/10/21 | Green = Science | Blue = High-Rate | Red = Maintenance | White = Missing Data
LOW-A
LOW-B
M35-A
M35-B
M75-A
M75-B
HIGH-A
HIGH-B
Sep-01
Sep-04
Sep-07
Sep-10
Sep-13
Sep-16
Sep-19
Sep-22
Sep-25
Sep-28
2019

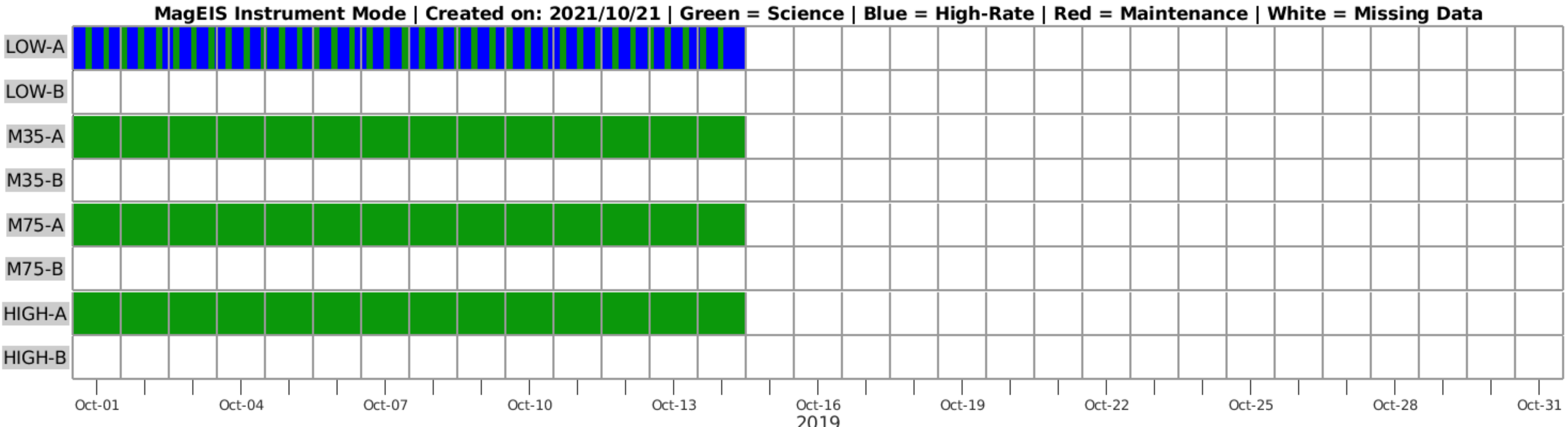
MagEIS Instrument Mode | Created on: 2021/10/21 | Green = Science | Blue = High-Rate | Red = Maintenance | White = Missing Data
LOW-A
LOW-B
M35-A
M35-B
M75-A
M75-B
HIGH-A
HIGH-B
Oct-01
Oct-04
Oct-07
Oct-10
Oct-13
Oct-16
Oct-19
Oct-22
Oct-25
Oct-28
Oct-31
2019
