# Supplementary material for: The Magnetic Electron Ion Spectrometer: A Review of On-Orbit Sensor Performance, Data, Operations, and Science
Source: Space Sci Rev. 2021 Oct 28;217(8):80. doi: 10.1007/s11214-021-00855-2 (PMC8553741; doi:10.1007/s11214-021-00855-2)
Supplement: Supplementary file 7 — Figures indicating the availability of the daily MagEIS CDF data files (levels 1, 2, and 3) for the entire mission (PDF 10.9 MB) [file 11214_2021_855_MOESM7_ESM.pdf]

Note:

At the time of publication, the “MERGE-A-hg” and “MERGE-B-hg” level 3 files had not yet been created. Thus, they are shown as red (file does not exist) in this document. It is anticipated that they will be created eventually, at which time they will become part of the final MagEIS data archive.

MagEIS Data Files | Created on: 2021/10/21 | Green = File Exists | Red = File Does Not Exist
sp=spin-based (science) | ns=non-science (housekeeping & status) | hr=highrate (LOW/MED only) | hg=histogram | de=direct event (HIGH only)

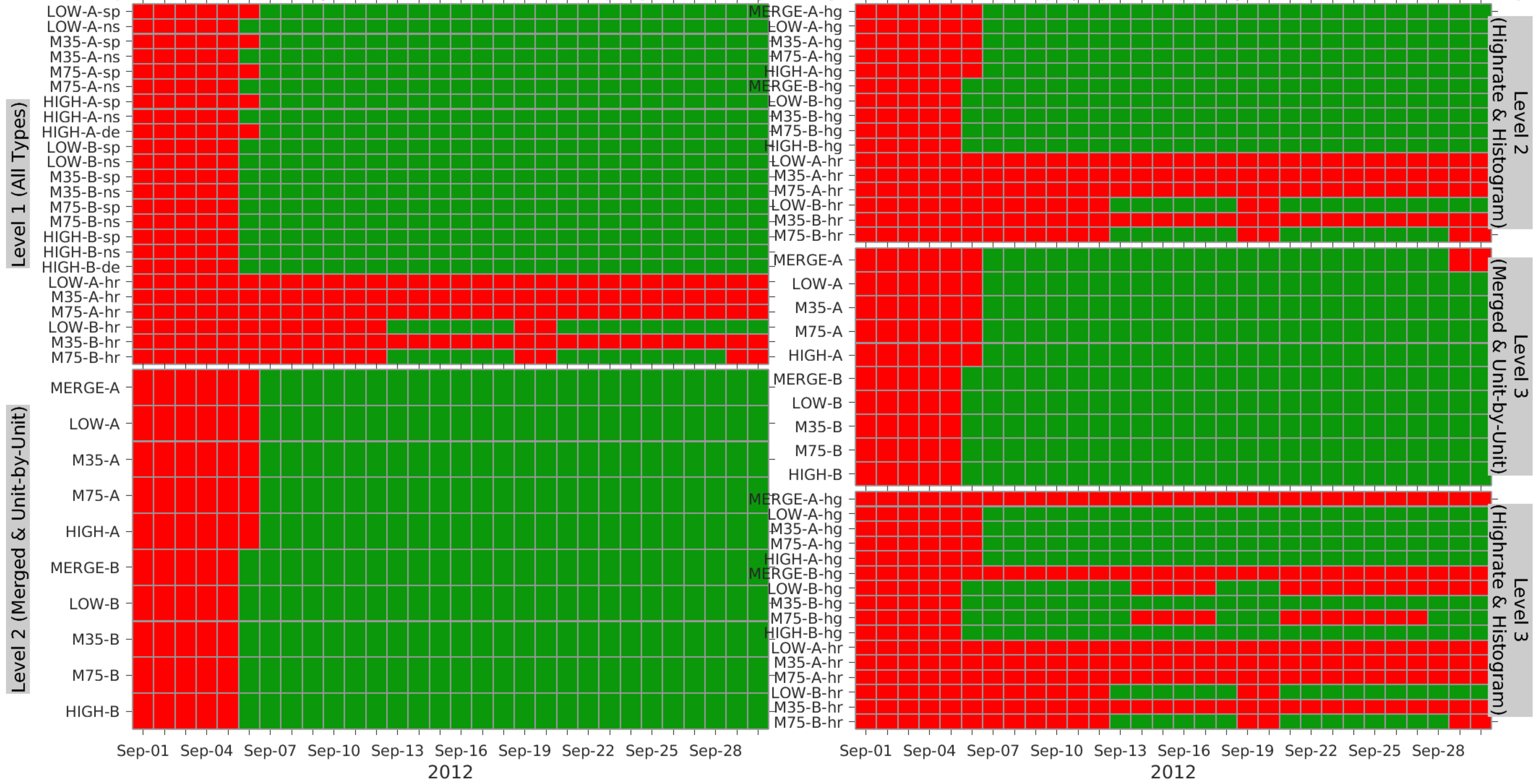

MagEIS Data Files | Created on: 2021/10/21 | Green = File Exists | Red = File Does Not Exist
sp=spin-based (science) | ns=non-science (housekeeping & status) | hr=highrate (LOW/MED only) | hg=histogram | de=direct event (HIGH only)

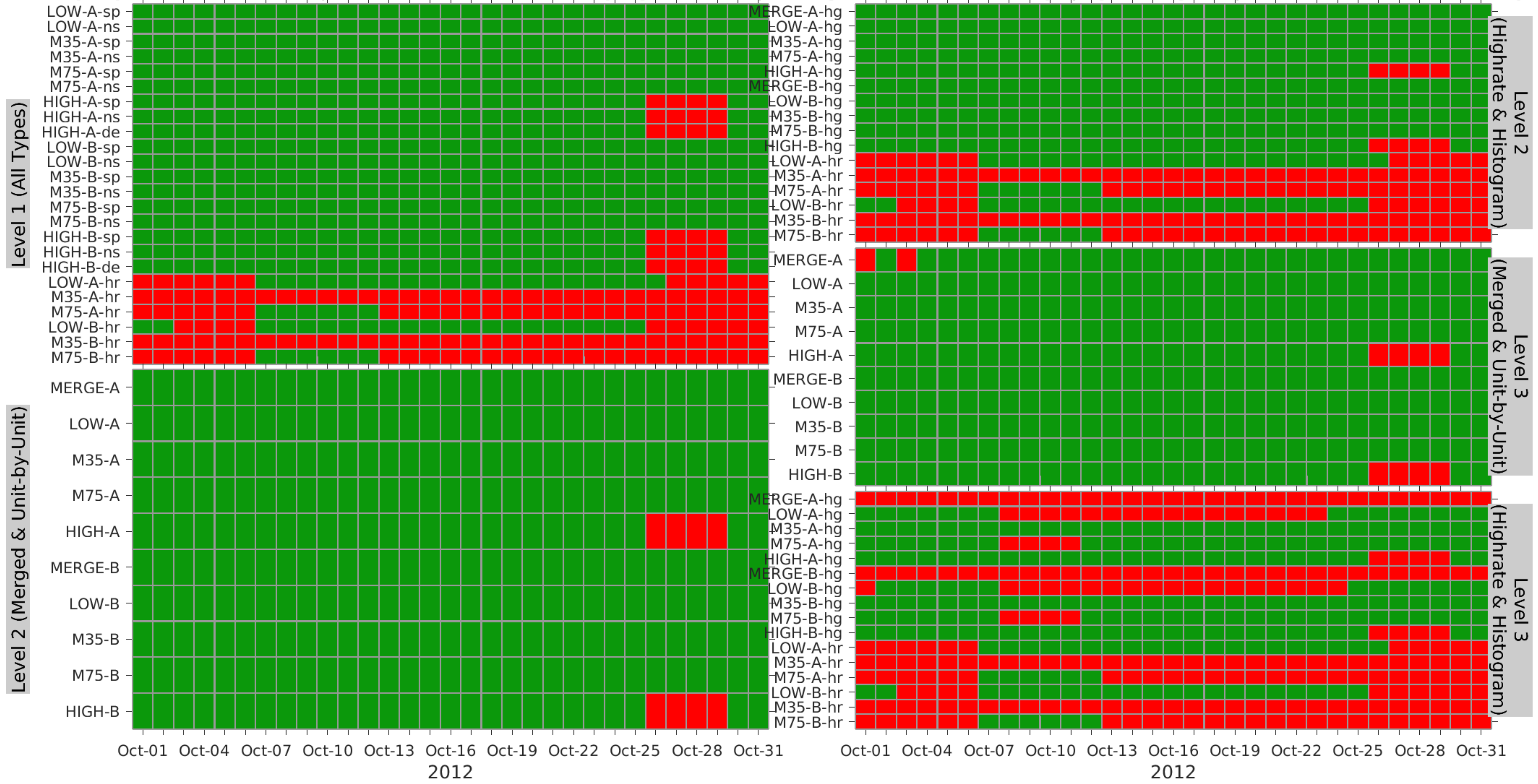

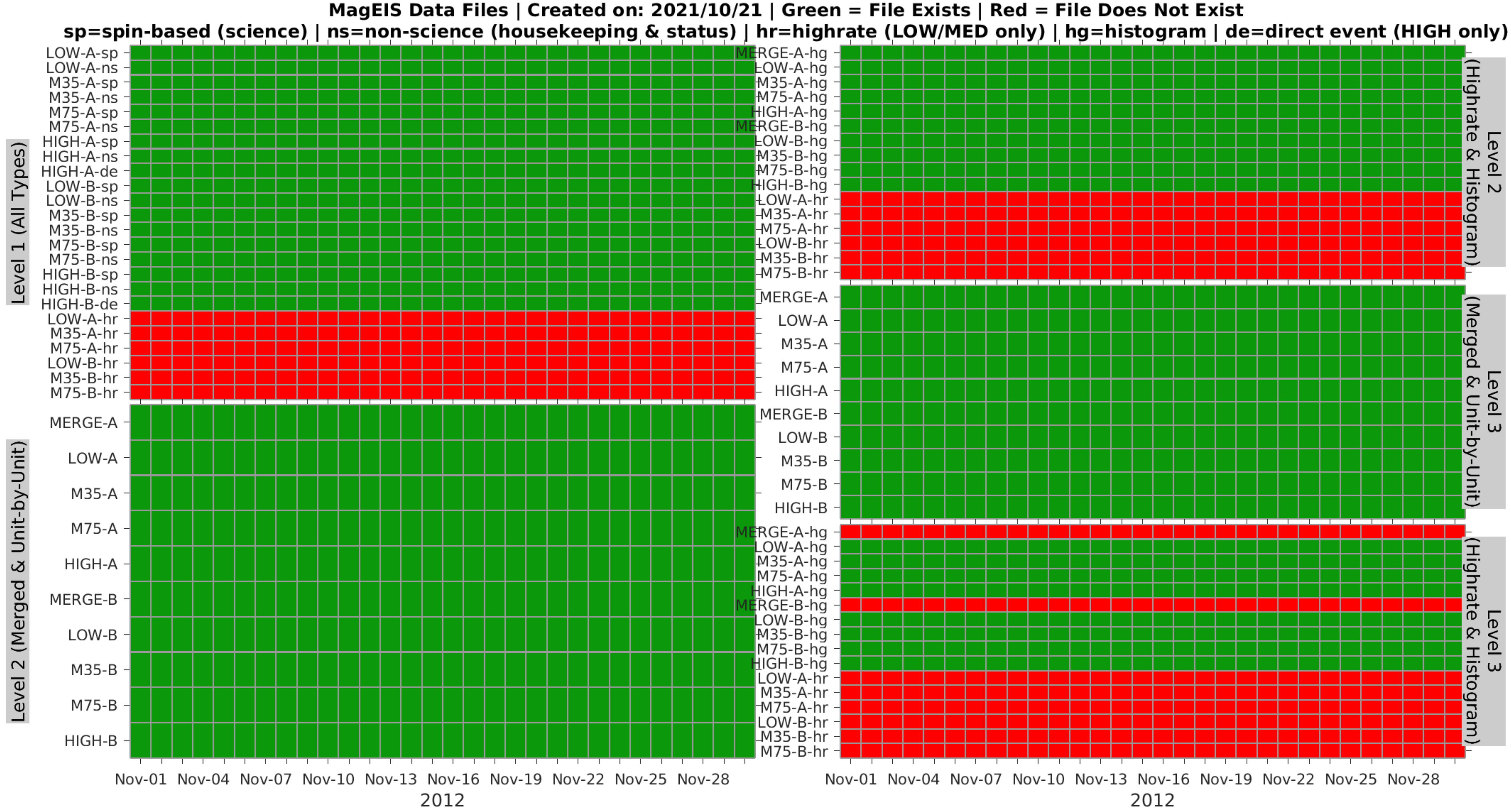
MagEIS Data Files | Created on: 2021/10/21 | Green = File Exists | Red = File Does Not Exist
sp=spin-based (science) | ns=non-science (housekeeping & status) | hr=highrate (LOW/MED only) | hg=histogram | de=direct event (HIGH only)
Level 1 (All Types)
LOW-A-sp
LOW-A-ns
M35-A-sp
M35-A-ns
M75-A-sp
M75-A-ns
HIGH-A-sp
HIGH-A-ns
HIGH-A-de
LOW-B-sp
LOW-B-ns
M35-B-sp
M35-B-ns
M75-B-sp
M75-B-ns
HIGH-B-sp
HIGH-B-ns
HIGH-B-de
LOW-A-hr
M35-A-hr
M75-A-hr
LOW-B-hr
M35-B-hr
M75-B-hr
Level 2 (Merged & Unit-by-Unit)
MERGE-A
LOW-A
M35-A
M75-A
HIGH-A
MERGE-B
LOW-B
M35-B
M75-B
HIGH-B
Level 2 (Highrate & Histogram)
MERGE-A-hg
LOW-A-hg
M35-A-hg
M75-A-hg
HIGH-A-hg
MERGE-B-hg
LOW-B-hg
M35-B-hg
M75-B-hg
HIGH-B-hg
LOW-A-hr
M35-A-hr
M75-A-hr
LOW-B-hr
M35-B-hr
M75-B-hr
Level 3 (Merged & Unit-by-Unit)
MERGE-A
LOW-A
M35-A
M75-A
HIGH-A
MERGE-B
LOW-B
M35-B
M75-B
HIGH-B
Level 3 (Highrate & Histogram)
MERGE-A-hg
LOW-A-hg
M35-A-hg
M75-A-hg
HIGH-A-hg
MERGE-B-hg
LOW-B-hg
M35-B-hg
M75-B-hg
HIGH-B-hg
LOW-A-hr
M35-A-hr
M75-A-hr
LOW-B-hr
M35-B-hr
M75-B-hr
Nov-01 Nov-04 Nov-07 Nov-10 Nov-13 Nov-16 Nov-19 Nov-22 Nov-25 Nov-28
2012

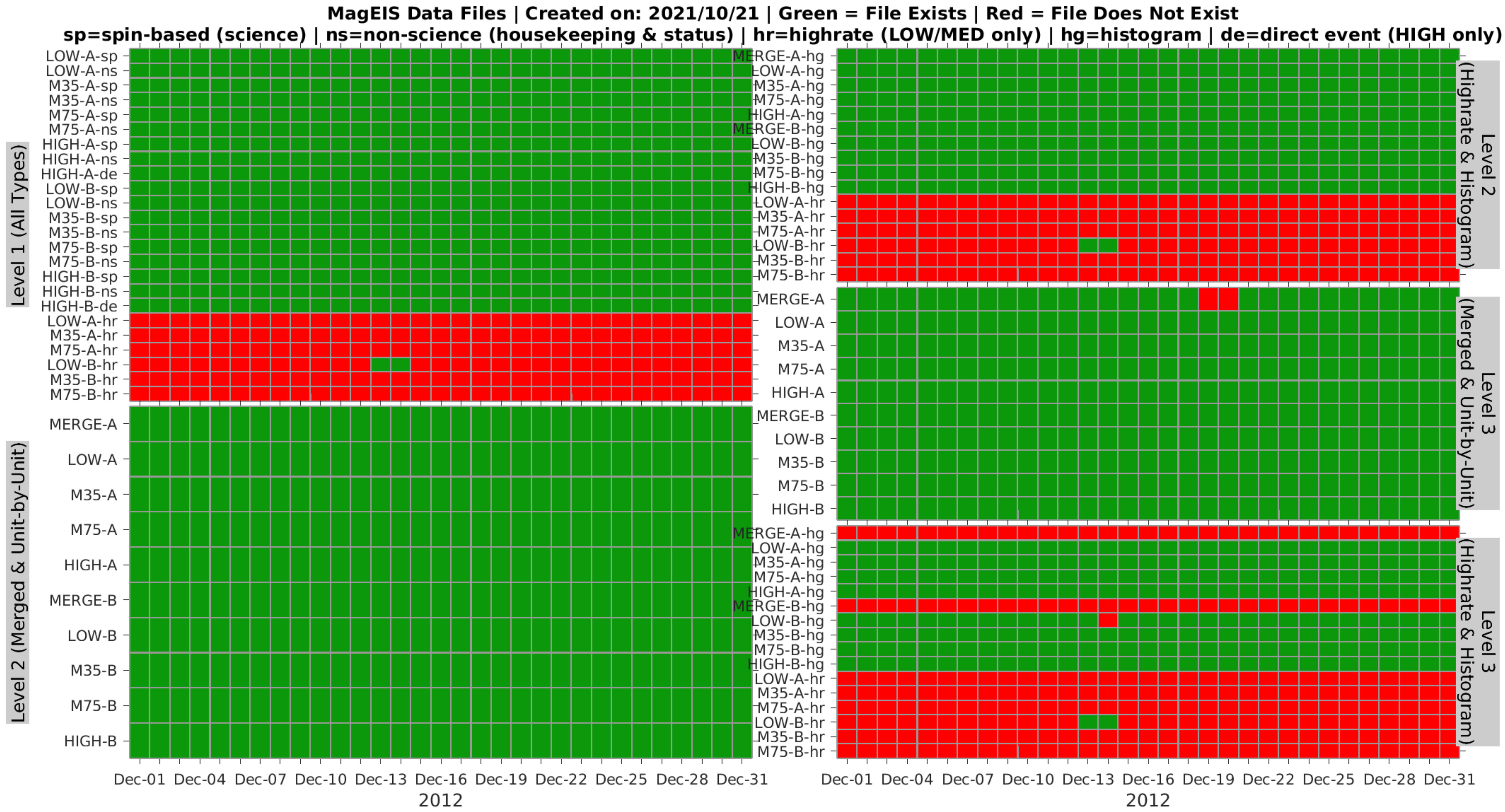

MagEIS Data Files | Created on: 2021/10/21 | Green = File Exists | Red = File Does Not Exist
sp=spin-based (science) | ns=non-science (housekeeping & status) | hr=highrate (LOW/MED only) | hg=histogram | de=direct event (HIGH only)
Level 1 (All Types)
LOW-A-sp
LOW-A-ns
M35-A-sp
M35-A-ns
M75-A-sp
M75-A-ns
HIGH-A-sp
HIGH-A-ns
HIGH-A-de
LOW-B-sp
LOW-B-ns
M35-B-sp
M35-B-ns
M75-B-sp
M75-B-ns
HIGH-B-sp
HIGH-B-ns
HIGH-B-de
LOW-A-hr
M35-A-hr
M75-A-hr
LOW-B-hr
M35-B-hr
M75-B-hr
Level 2 (Merged & Unit-by-Unit)
MERGE-A
LOW-A
M35-A
M75-A
HIGH-A
MERGE-B
LOW-B
M35-B
M75-B
HIGH-B
Level 2 (Highrate & Histogram)
MERGE-A-hg
LOW-A-hg
M35-A-hg
M75-A-hg
HIGH-A-hg
MERGE-B-hg
LOW-B-hg
M35-B-hg
M75-B-hg
HIGH-B-hg
LOW-A-hr
M35-A-hr
M75-A-hr
LOW-B-hr
M35-B-hr
M75-B-hr
Level 3 (Merged & Unit-by-Unit)
MERGE-A
LOW-A
M35-A
M75-A
HIGH-A
MERGE-B
LOW-B
M35-B
M75-B
HIGH-B
Level 3 (Highrate & Histogram)
MERGE-A-hg
LOW-A-hg
M35-A-hg
M75-A-hg
HIGH-A-hg
MERGE-B-hg
LOW-B-hg
M35-B-hg
M75-B-hg
HIGH-B-hg
LOW-A-hr
M35-A-hr
M75-A-hr
LOW-B-hr
M35-B-hr
M75-B-hr
Dec-01
Dec-04
Dec-07
Dec-10
Dec-13
Dec-16
Dec-19
Dec-22
Dec-25
Dec-28
Dec-31
2012

MagEIS Data Files | Created on: 2021/10/21 | Green = File Exists | Red = File Does Not Exist
sp=spin-based (science) | ns=non-science (housekeeping & status) | hr=highrate (LOW/MED only) | hg=histogram | de=direct event (HIGH only)

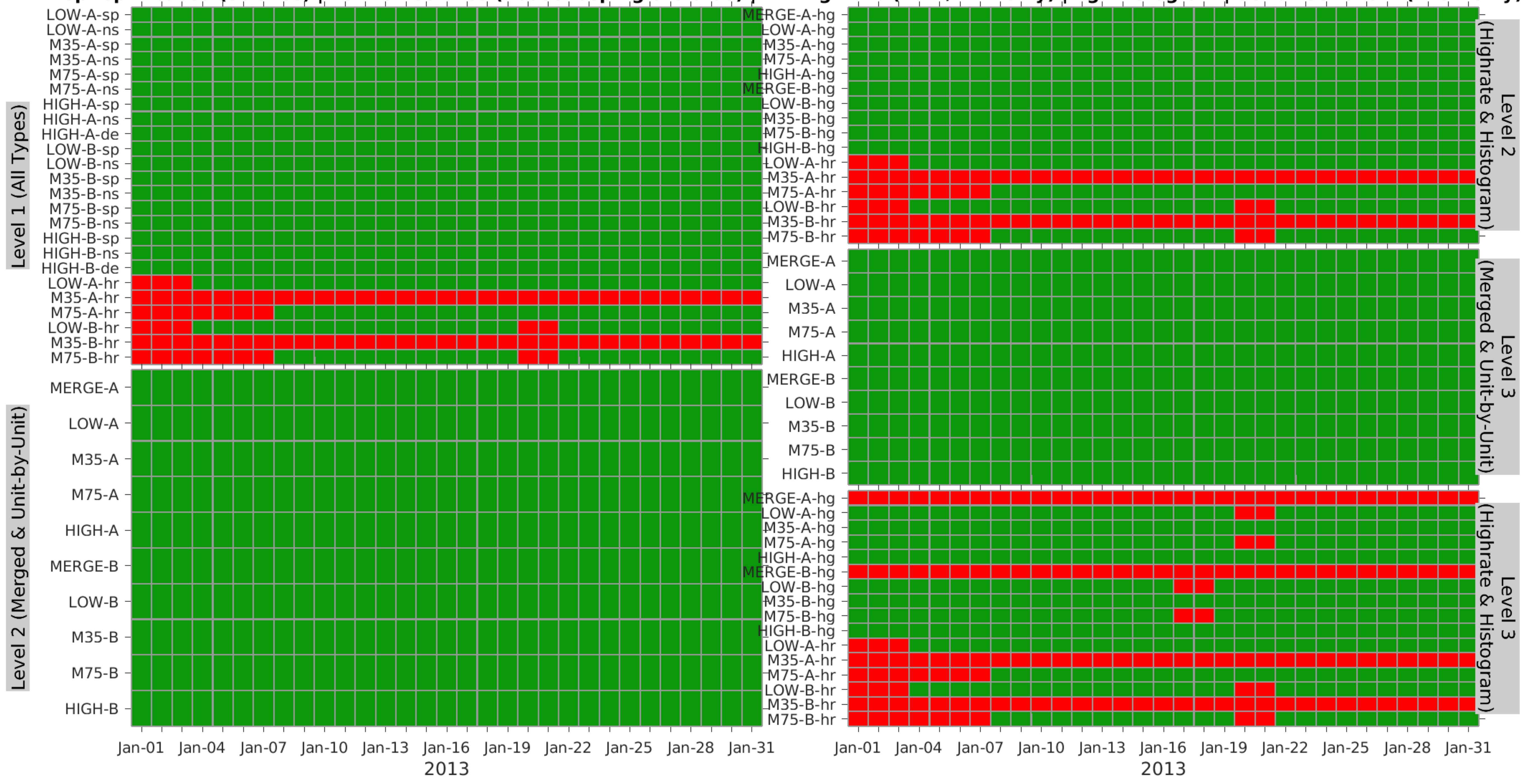

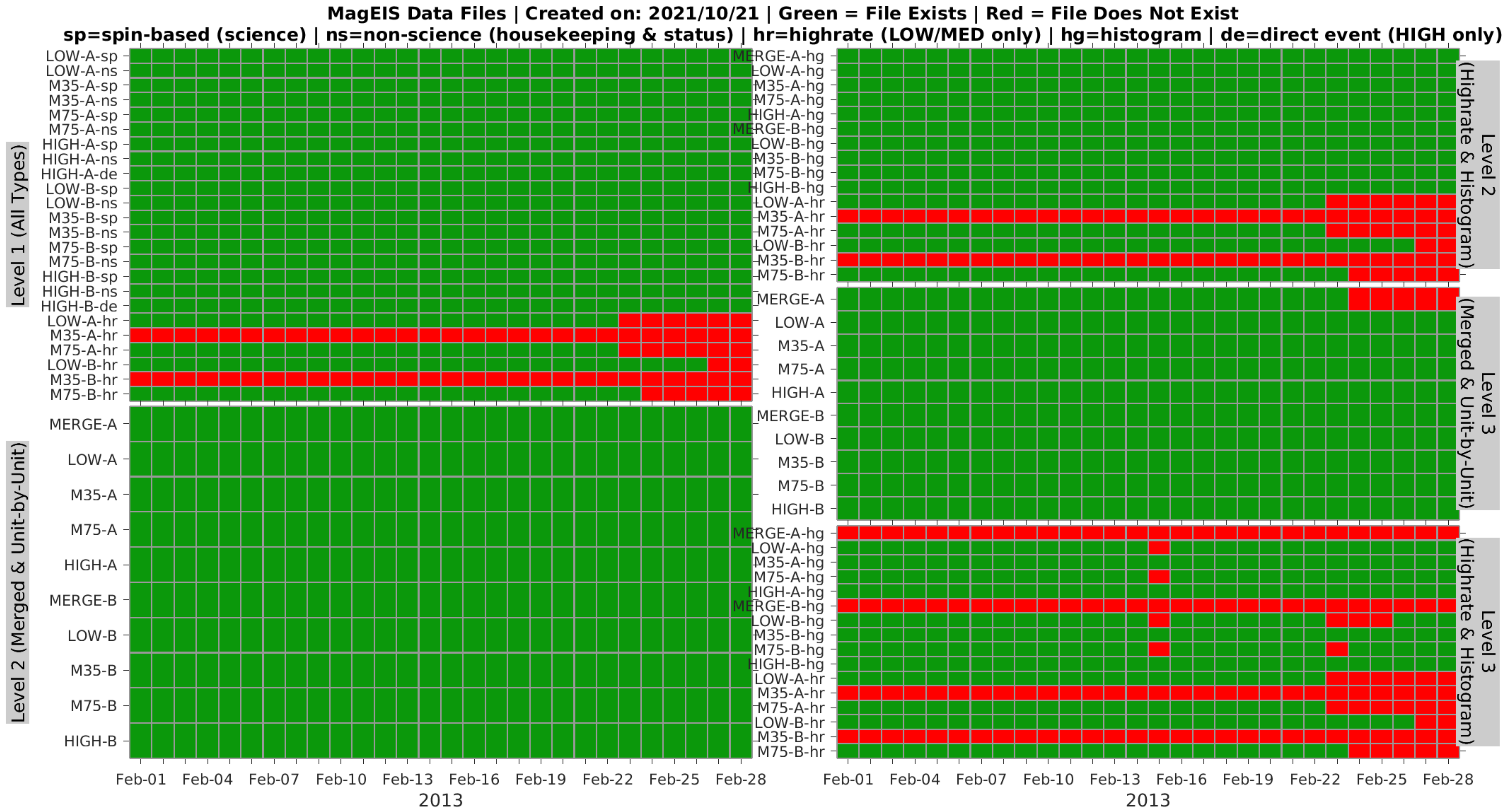
MagEIS Data Files | Created on: 2021/10/21 | Green = File Exists | Red = File Does Not Exist
sp=spin-based (science) | ns=non-science (housekeeping & status) | hr=highrate (LOW/MED only) | hg=histogram | de=direct event (HIGH only)
Level 1 (All Types)
LOW-A-sp
LOW-A-ns
M35-A-sp
M35-A-ns
M75-A-sp
M75-A-ns
HIGH-A-sp
HIGH-A-ns
HIGH-A-de
LOW-B-sp
LOW-B-ns
M35-B-sp
M35-B-ns
M75-B-sp
M75-B-ns
HIGH-B-sp
HIGH-B-ns
HIGH-B-de
LOW-A-hr
M35-A-hr
M75-A-hr
LOW-B-hr
M35-B-hr
M75-B-hr
Level 2 (Merged & Unit-by-Unit)
MERGE-A
LOW-A
M35-A
M75-A
HIGH-A
MERGE-B
LOW-B
M35-B
M75-B
HIGH-B
Level 2 (Highrate & Histogram)
MERGE-A-hg
LOW-A-hg
M35-A-hg
M75-A-hg
HIGH-A-hg
MERGE-B-hg
LOW-B-hg
M35-B-hg
M75-B-hg
HIGH-B-hg
LOW-A-hr
M35-A-hr
M75-A-hr
LOW-B-hr
M35-B-hr
M75-B-hr
Level 3 (Merged & Unit-by-Unit)
MERGE-A
LOW-A
M35-A
M75-A
HIGH-A
MERGE-B
LOW-B
M35-B
M75-B
HIGH-B
Level 3 (Highrate & Histogram)
MERGE-A-hg
LOW-A-hg
M35-A-hg
M75-A-hg
HIGH-A-hg
MERGE-B-hg
LOW-B-hg
M35-B-hg
M75-B-hg
HIGH-B-hg
LOW-A-hr
M35-A-hr
M75-A-hr
LOW-B-hr
M35-B-hr
M75-B-hr
Feb-01
Feb-04
Feb-07
Feb-10
Feb-13
Feb-16
Feb-19
Feb-22
Feb-25
Feb-28
2013

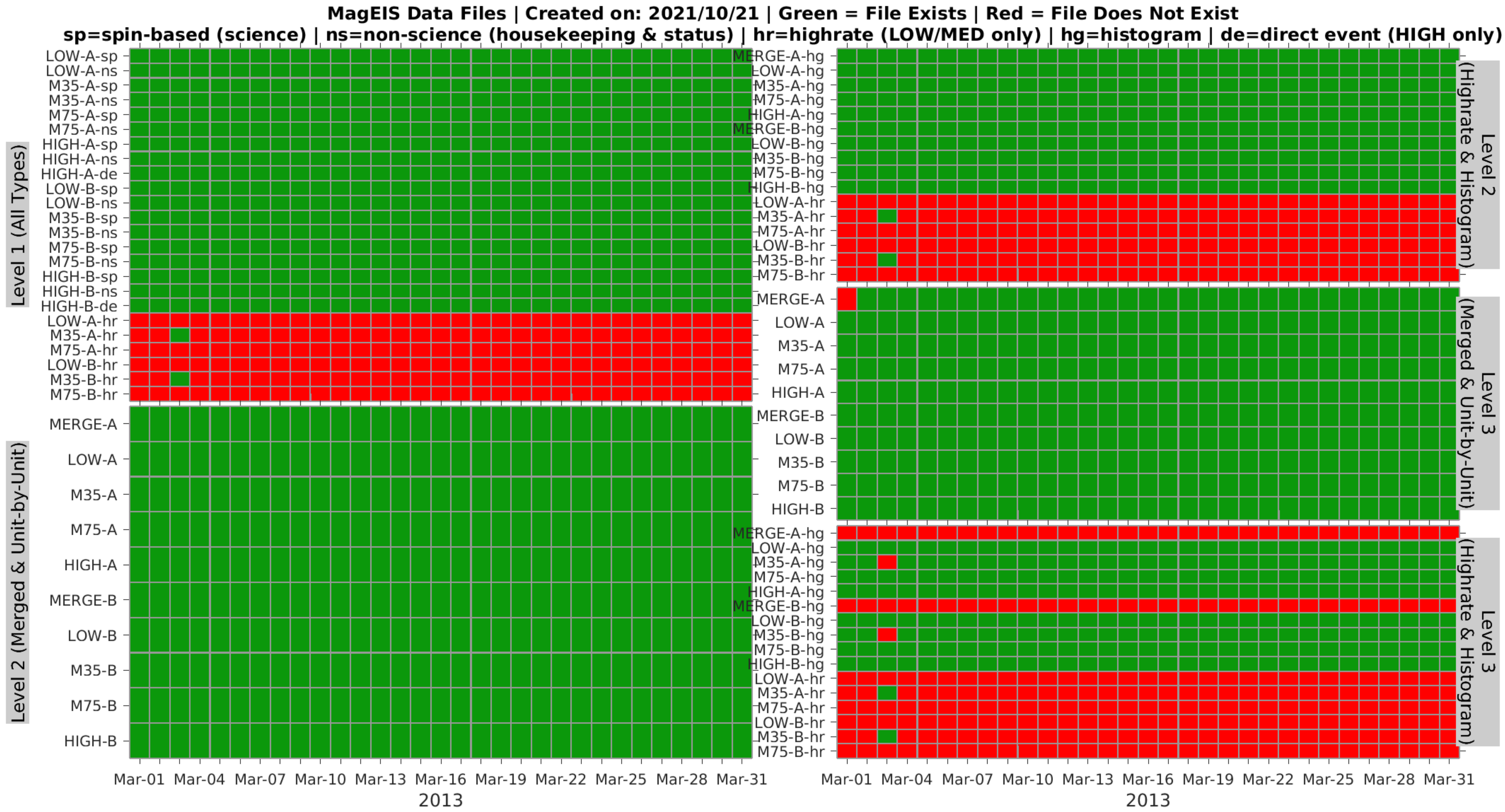

MagEIS Data Files | Created on: 2021/10/21 | Green = File Exists | Red = File Does Not Exist
sp=spin-based (science) | ns=non-science (housekeeping & status) | hr=highrate (LOW/MED only) | hg=histogram | de=direct event (HIGH only)
Level 1 (All Types)
LOW-A-sp
LOW-A-ns
M35-A-sp
M35-A-ns
M75-A-sp
M75-A-ns
HIGH-A-sp
HIGH-A-ns
HIGH-A-de
LOW-B-sp
LOW-B-ns
M35-B-sp
M35-B-ns
M75-B-sp
M75-B-ns
HIGH-B-sp
HIGH-B-ns
HIGH-B-de
LOW-A-hr
M35-A-hr
M75-A-hr
LOW-B-hr
M35-B-hr
M75-B-hr
Level 2 (Merged & Unit-by-Unit)
MERGE-A
LOW-A
M35-A
M75-A
HIGH-A
MERGE-B
LOW-B
M35-B
M75-B
HIGH-B
Level 2 (Highrate & Histogram)
MERGE-A-hg
LOW-A-hg
M35-A-hg
M75-A-hg
HIGH-A-hg
MERGE-B-hg
LOW-B-hg
M35-B-hg
M75-B-hg
HIGH-B-hg
LOW-A-hr
M35-A-hr
M75-A-hr
LOW-B-hr
M35-B-hr
M75-B-hr
Level 3 (Merged & Unit-by-Unit)
MERGE-A
LOW-A
M35-A
M75-A
HIGH-A
MERGE-B
LOW-B
M35-B
M75-B
HIGH-B
Level 3 (Highrate & Histogram)
MERGE-A-hg
LOW-A-hg
M35-A-hg
M75-A-hg
HIGH-A-hg
MERGE-B-hg
LOW-B-hg
M35-B-hg
M75-B-hg
HIGH-B-hg
LOW-A-hr
M35-A-hr
M75-A-hr
LOW-B-hr
M35-B-hr
M75-B-hr
Mar-01
Mar-04
Mar-07
Mar-10
Mar-13
Mar-16
Mar-19
Mar-22
Mar-25
Mar-28
Mar-31
2013

MagEIS Data Files | Created on: 2021/10/21 | Green = File Exists | Red = File Does Not Exist
sp=spin-based (science) | ns=non-science (housekeeping & status) | hr=highrate (LOW/MED only) | hg=histogram | de=direct event (HIGH only)

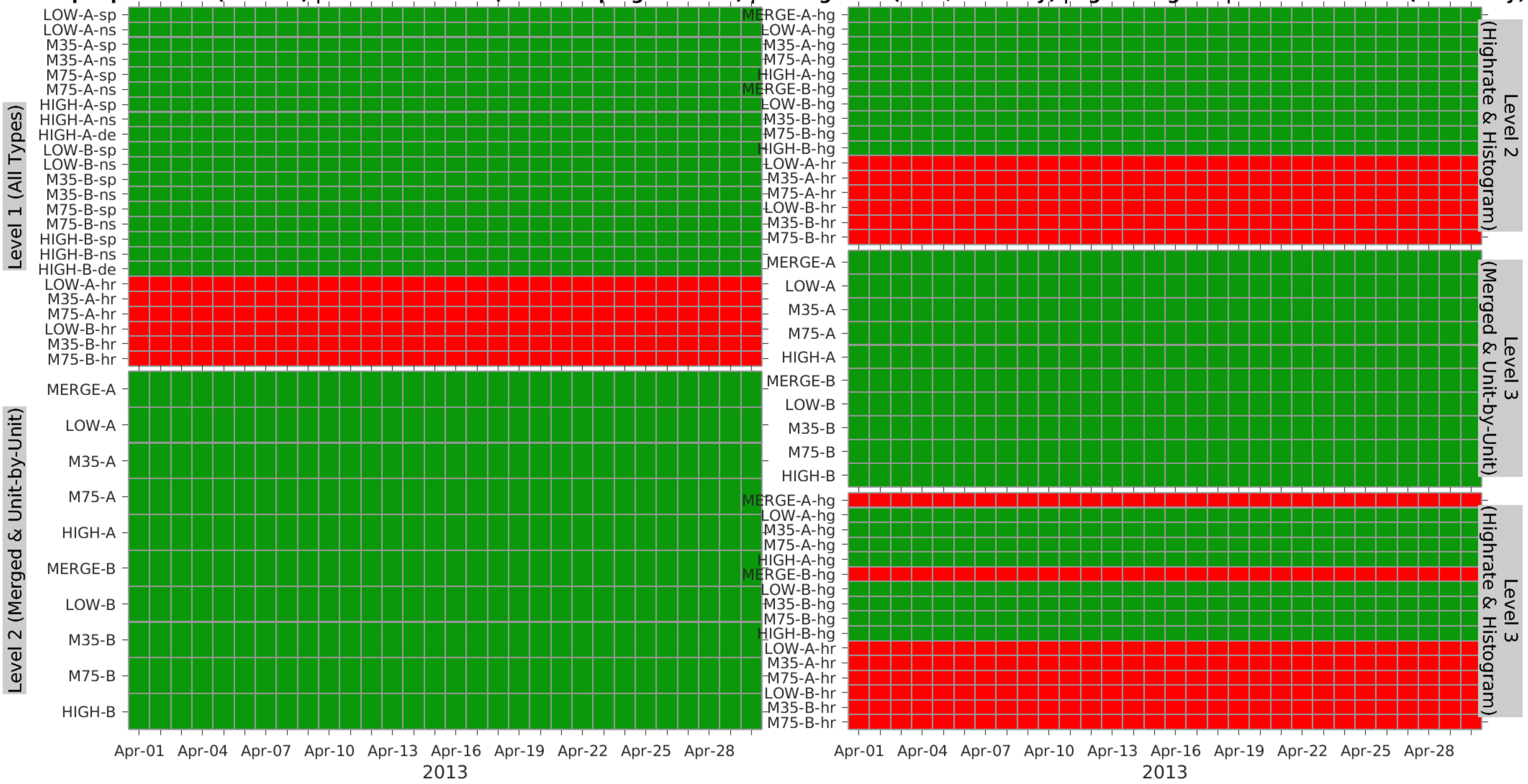

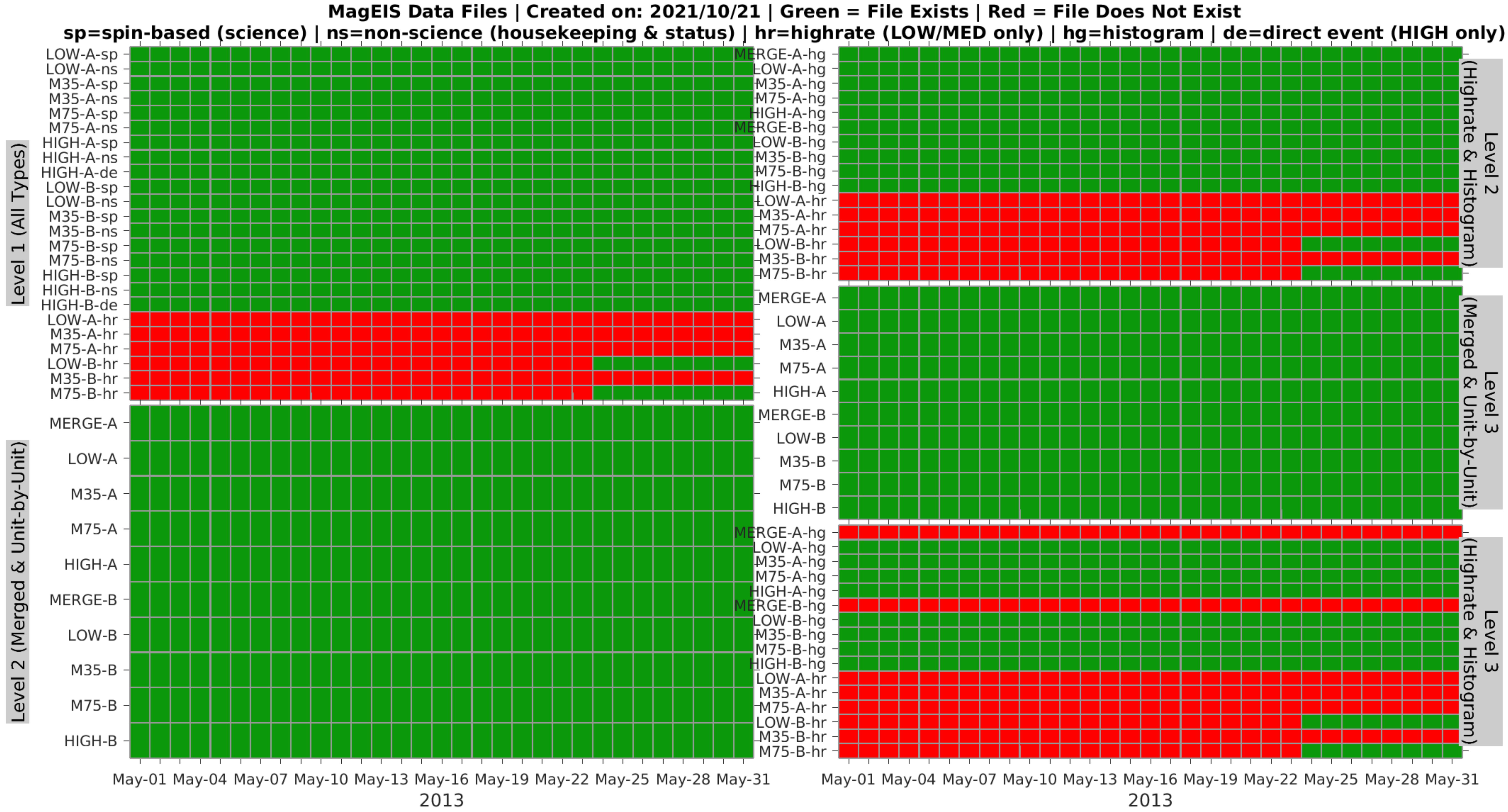
MagEIS Data Files | Created on: 2021/10/21 | Green = File Exists | Red = File Does Not Exist
sp=spin-based (science) | ns=non-science (housekeeping & status) | hr=highrate (LOW/MED only) | hg=histogram | de=direct event (HIGH only)
Level 1 (All Types)
LOW-A-sp
LOW-A-ns
M35-A-sp
M35-A-ns
M75-A-sp
M75-A-ns
HIGH-A-sp
HIGH-A-ns
HIGH-A-de
LOW-B-sp
LOW-B-ns
M35-B-sp
M35-B-ns
M75-B-sp
M75-B-ns
HIGH-B-sp
HIGH-B-ns
HIGH-B-de
LOW-A-hr
M35-A-hr
M75-A-hr
LOW-B-hr
M35-B-hr
M75-B-hr
Level 2 (Merged & Unit-by-Unit)
MERGE-A
LOW-A
M35-A
M75-A
HIGH-A
MERGE-B
LOW-B
M35-B
M75-B
HIGH-B
Level 2 (Highrate & Histogram)
MERGE-A-hg
LOW-A-hg
M35-A-hg
M75-A-hg
HIGH-A-hg
MERGE-B-hg
LOW-B-hg
M35-B-hg
M75-B-hg
HIGH-B-hg
LOW-A-hr
M35-A-hr
M75-A-hr
LOW-B-hr
M35-B-hr
M75-B-hr
Level 3 (Merged & Unit-by-Unit)
Level 3 (Highrate & Histogram)
May-01 May-04 May-07 May-10 May-13 May-16 May-19 May-22 May-25 May-28 May-31
2013

MagEIS Data Files | Created on: 2021/10/21 | Green = File Exists | Red = File Does Not Exist
sp=spin-based (science) | ns=non-science (housekeeping & status) | hr=highrate (LOW/MED only) | hg=histogram | de=direct event (HIGH only)

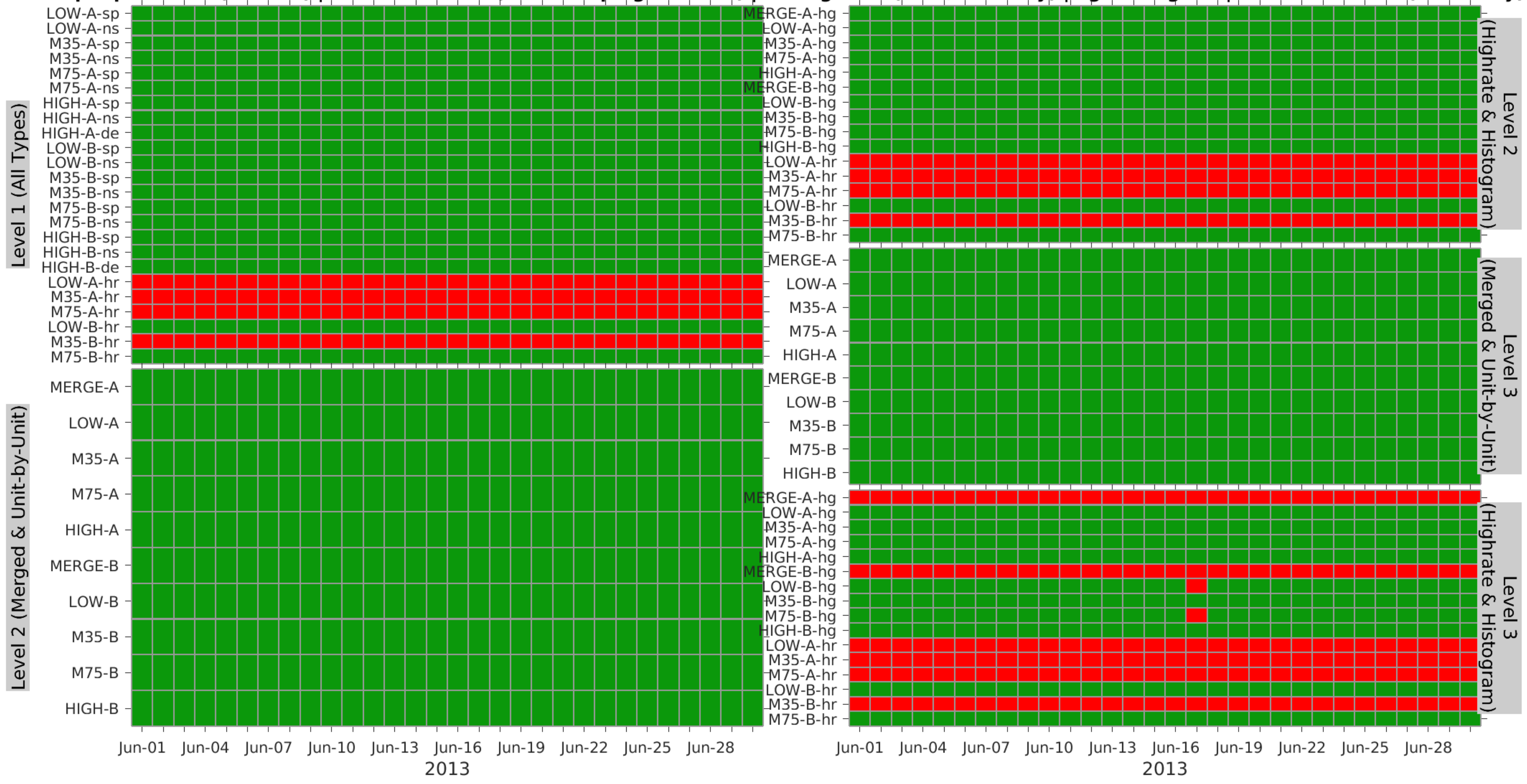

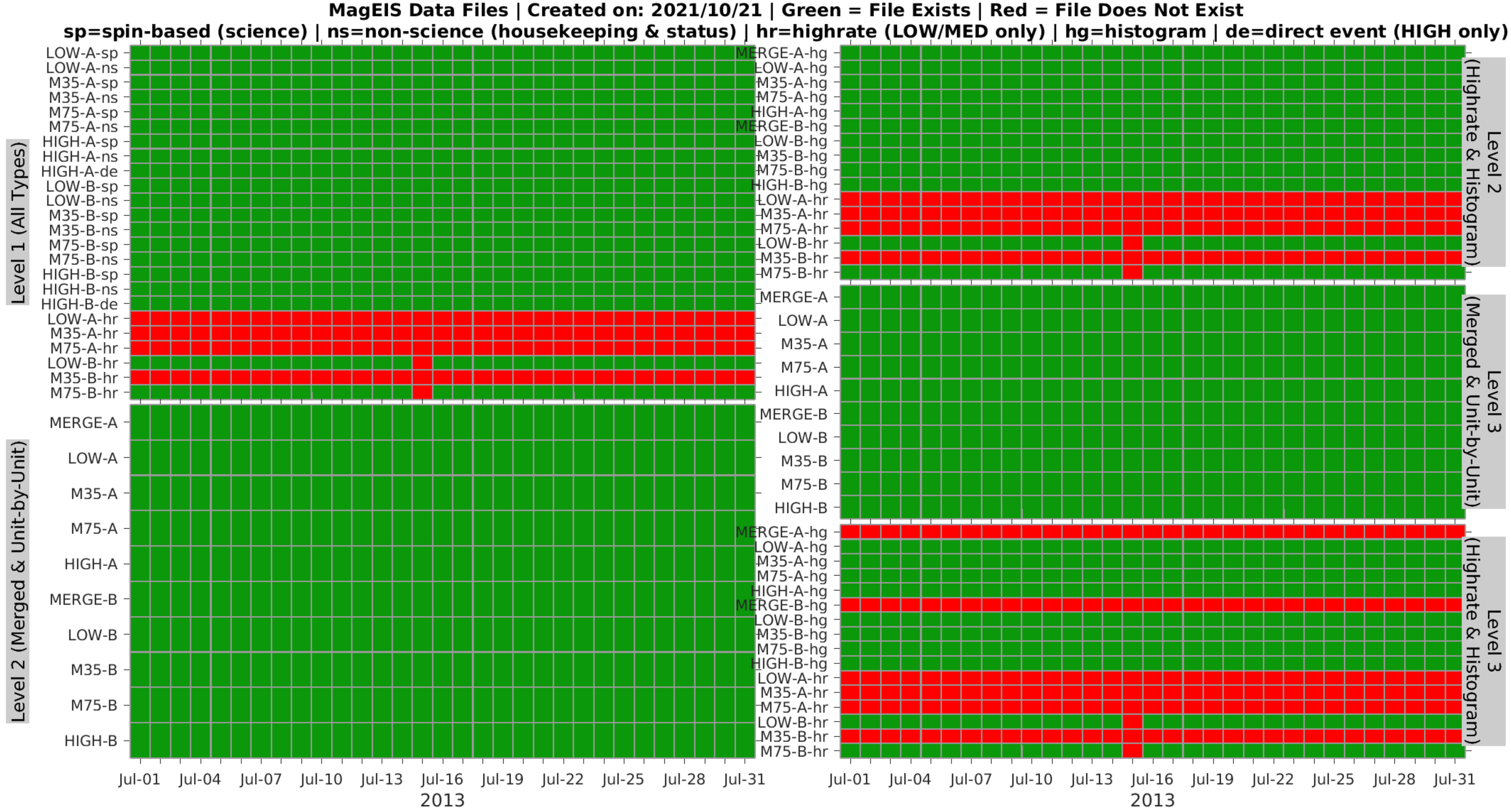
MagEIS Data Files | Created on: 2021/10/21 | Green = File Exists | Red = File Does Not Exist
sp=spin-based (science) | ns=non-science (housekeeping & status) | hr=highrate (LOW/MED only) | hg=histogram | de=direct event (HIGH only)
Level 1 (All Types)
LOW-A-sp
LOW-A-ns
M35-A-sp
M35-A-ns
M75-A-sp
M75-A-ns
HIGH-A-sp
HIGH-A-ns
HIGH-A-de
LOW-B-sp
LOW-B-ns
M35-B-sp
M35-B-ns
M75-B-sp
M75-B-ns
HIGH-B-sp
HIGH-B-ns
HIGH-B-de
LOW-A-hr
M35-A-hr
M75-A-hr
LOW-B-hr
M35-B-hr
M75-B-hr
Level 2 (Merged & Unit-by-Unit)
MERGE-A
LOW-A
M35-A
M75-A
HIGH-A
MERGE-B
LOW-B
M35-B
M75-B
HIGH-B
Level 2 (Highrate & Histogram)
MERGE-A-hg
LOW-A-hg
M35-A-hg
M75-A-hg
HIGH-A-hg
MERGE-B-hg
LOW-B-hg
M35-B-hg
M75-B-hg
HIGH-B-hg
LOW-A-hr
M35-A-hr
M75-A-hr
LOW-B-hr
M35-B-hr
M75-B-hr
Level 3 (Merged & Unit-by-Unit)
MERGE-A
LOW-A
M35-A
M75-A
HIGH-A
MERGE-B
LOW-B
M35-B
M75-B
HIGH-B
Level 3 (Highrate & Histogram)
MERGE-A-hg
LOW-A-hg
M35-A-hg
M75-A-hg
HIGH-A-hg
MERGE-B-hg
LOW-B-hg
M35-B-hg
M75-B-hg
HIGH-B-hg
LOW-A-hr
M35-A-hr
M75-A-hr
LOW-B-hr
M35-B-hr
M75-B-hr
Jul-01
Jul-04
Jul-07
Jul-10
Jul-13
Jul-16
Jul-19
Jul-22
Jul-25
Jul-28
Jul-31
2013

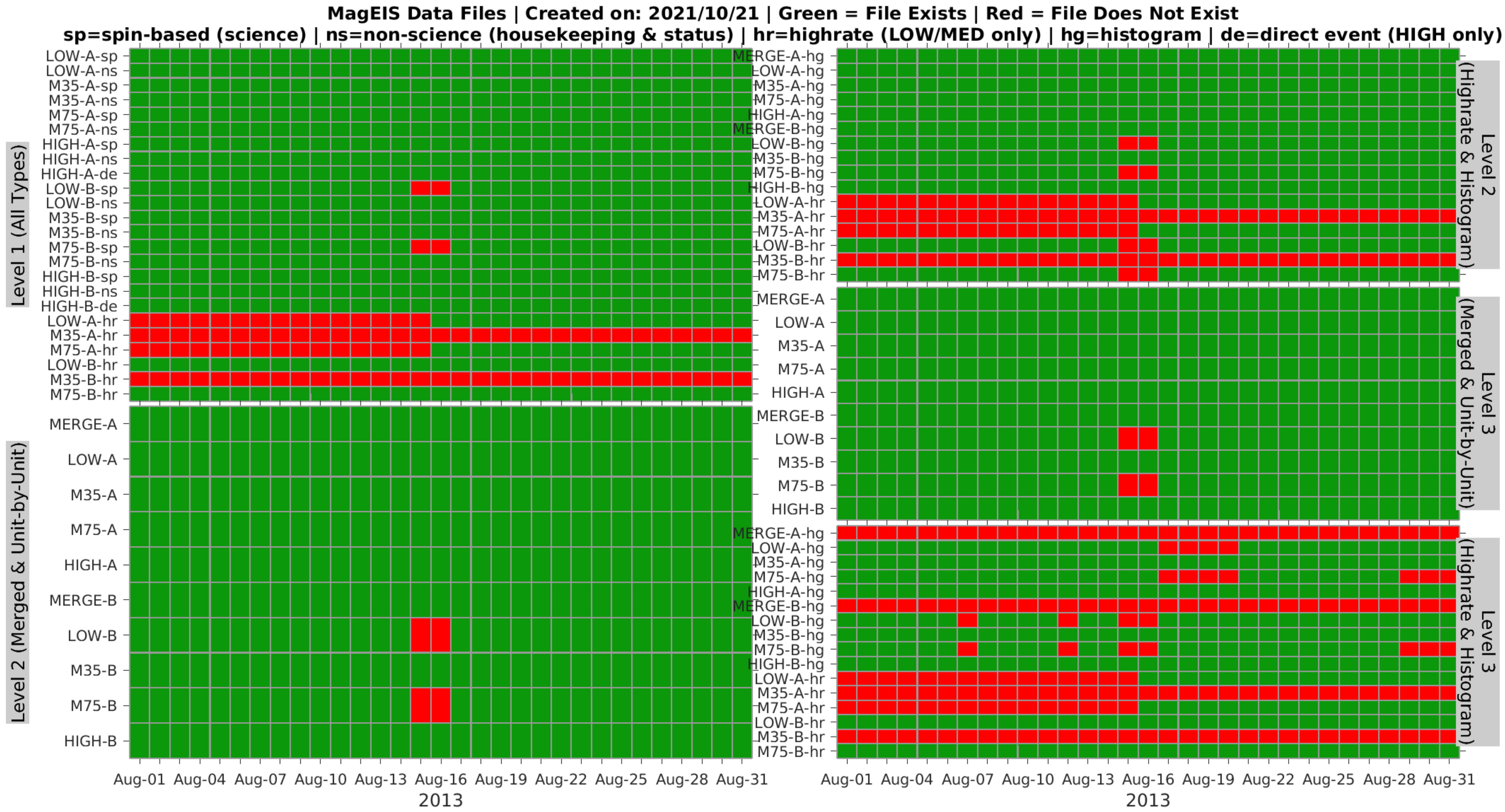
MagEIS Data Files | Created on: 2021/10/21 | Green = File Exists | Red = File Does Not Exist
sp=spin-based (science) | ns=non-science (housekeeping & status) | hr=highrate (LOW/MED only) | hg=histogram | de=direct event (HIGH only)
Level 1 (All Types)
LOW-A-sp
LOW-A-ns
M35-A-sp
M35-A-ns
M75-A-sp
M75-A-ns
HIGH-A-sp
HIGH-A-ns
HIGH-A-de
LOW-B-sp
LOW-B-ns
M35-B-sp
M35-B-ns
M75-B-sp
M75-B-ns
HIGH-B-sp
HIGH-B-ns
HIGH-B-de
LOW-A-hr
M35-A-hr
M75-A-hr
LOW-B-hr
M35-B-hr
M75-B-hr
Level 2 (Merged & Unit-by-Unit)
MERGE-A
LOW-A
M35-A
M75-A
HIGH-A
MERGE-B
LOW-B
M35-B
M75-B
HIGH-B
Level 2 (Highrate & Histogram)
MERGE-A-hg
LOW-A-hg
M35-A-hg
M75-A-hg
HIGH-A-hg
MERGE-B-hg
LOW-B-hg
M35-B-hg
M75-B-hg
HIGH-B-hg
LOW-A-hr
M35-A-hr
M75-A-hr
LOW-B-hr
M35-B-hr
M75-B-hr
Level 3 (Merged & Unit-by-Unit)
MERGE-A
LOW-A
M35-A
M75-A
HIGH-A
MERGE-B
LOW-B
M35-B
M75-B
HIGH-B
Level 3 (Highrate & Histogram)
MERGE-A-hg
LOW-A-hg
M35-A-hg
M75-A-hg
HIGH-A-hg
MERGE-B-hg
LOW-B-hg
M35-B-hg
M75-B-hg
HIGH-B-hg
LOW-A-hr
M35-A-hr
M75-A-hr
LOW-B-hr
M35-B-hr
M75-B-hr
Aug-01 Aug-04 Aug-07 Aug-10 Aug-13 Aug-16 Aug-19 Aug-22 Aug-25 Aug-28 Aug-31
2013

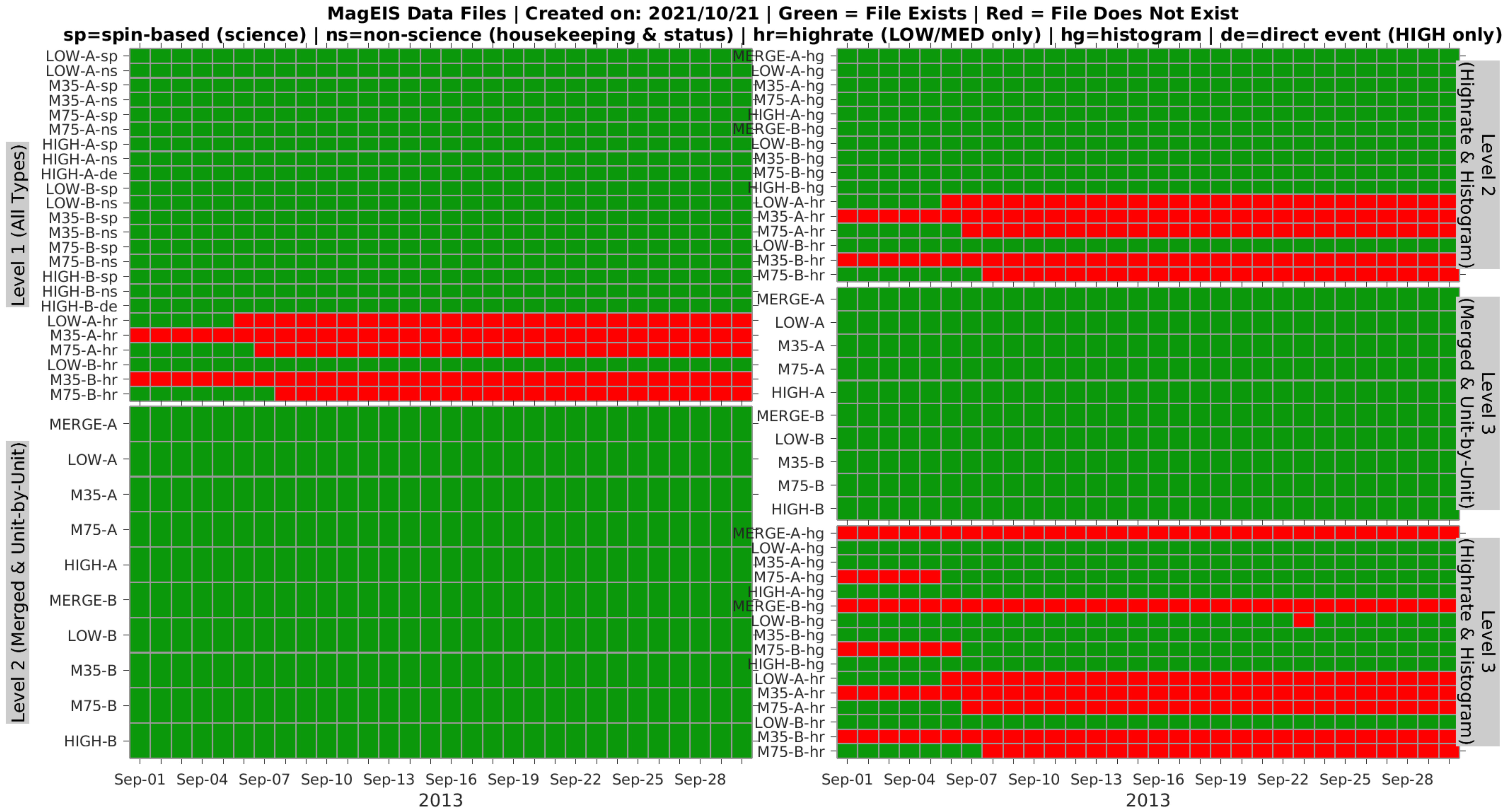
MagEIS Data Files | Created on: 2021/10/21 | Green = File Exists | Red = File Does Not Exist
sp=spin-based (science) | ns=non-science (housekeeping & status) | hr=highrate (LOW/MED only) | hg=histogram | de=direct event (HIGH only)
Level 1 (All Types)
LOW-A-sp
LOW-A-ns
M35-A-sp
M35-A-ns
M75-A-sp
M75-A-ns
HIGH-A-sp
HIGH-A-ns
HIGH-A-de
LOW-B-sp
LOW-B-ns
M35-B-sp
M35-B-ns
M75-B-sp
M75-B-ns
HIGH-B-sp
HIGH-B-ns
HIGH-B-de
LOW-A-hr
M35-A-hr
M75-A-hr
LOW-B-hr
M35-B-hr
M75-B-hr
Level 2 (Merged & Unit-by-Unit)
MERGE-A
LOW-A
M35-A
M75-A
HIGH-A
MERGE-B
LOW-B
M35-B
M75-B
HIGH-B
Level 2 (Highrate & Histogram)
MERGE-A-hg
LOW-A-hg
M35-A-hg
M75-A-hg
HIGH-A-hg
MERGE-B-hg
LOW-B-hg
M35-B-hg
M75-B-hg
HIGH-B-hg
LOW-A-hr
M35-A-hr
M75-A-hr
LOW-B-hr
M35-B-hr
M75-B-hr
Level 3 (Merged & Unit-by-Unit)
MERGE-A
LOW-A
M35-A
M75-A
HIGH-A
MERGE-B
LOW-B
M35-B
M75-B
HIGH-B
Level 3 (Highrate & Histogram)
MERGE-A-hg
LOW-A-hg
M35-A-hg
M75-A-hg
HIGH-A-hg
MERGE-B-hg
LOW-B-hg
M35-B-hg
M75-B-hg
HIGH-B-hg
LOW-A-hr
M35-A-hr
M75-A-hr
LOW-B-hr
M35-B-hr
M75-B-hr
Sep-01 Sep-04 Sep-07 Sep-10 Sep-13 Sep-16 Sep-19 Sep-22 Sep-25 Sep-28
2013

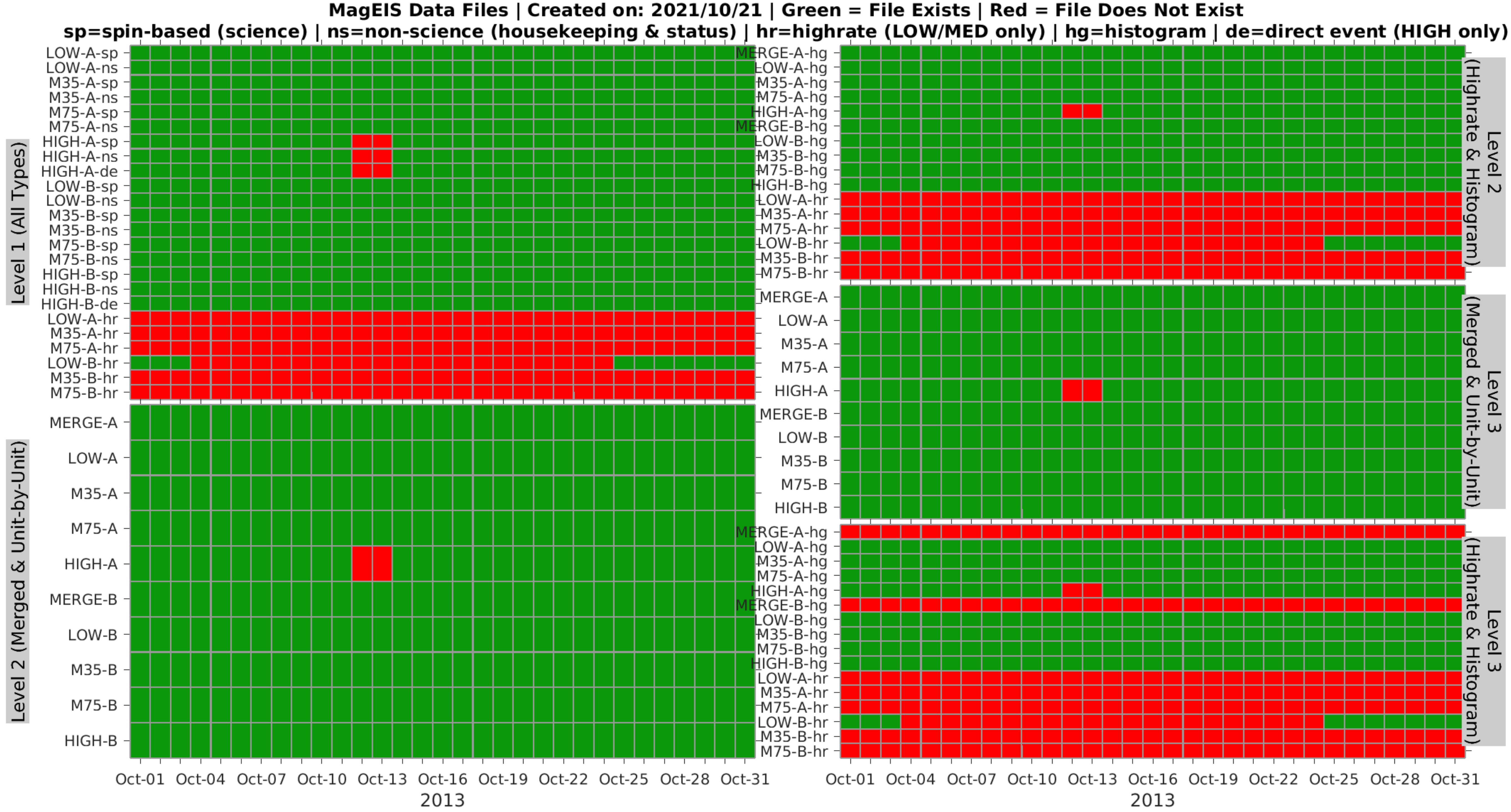
MagEIS Data Files | Created on: 2021/10/21 | Green = File Exists | Red = File Does Not Exist
sp=spin-based (science) | ns=non-science (housekeeping & status) | hr=highrate (LOW/MED only) | hg=histogram | de=direct event (HIGH only)
Level 1 (All Types)
LOW-A-sp
LOW-A-ns
M35-A-sp
M35-A-ns
M75-A-sp
M75-A-ns
HIGH-A-sp
HIGH-A-ns
HIGH-A-de
LOW-B-sp
LOW-B-ns
M35-B-sp
M35-B-ns
M75-B-sp
M75-B-ns
HIGH-B-sp
HIGH-B-ns
HIGH-B-de
LOW-A-hr
M35-A-hr
M75-A-hr
LOW-B-hr
M35-B-hr
M75-B-hr
Level 2 (Merged & Unit-by-Unit)
MERGE-A
LOW-A
M35-A
M75-A
HIGH-A
MERGE-B
LOW-B
M35-B
M75-B
HIGH-B
Level 2 (Highrate & Histogram)
MERGE-A-hg
LOW-A-hg
M35-A-hg
M75-A-hg
HIGH-A-hg
MERGE-B-hg
LOW-B-hg
M35-B-hg
M75-B-hg
HIGH-B-hg
LOW-A-hr
M35-A-hr
M75-A-hr
LOW-B-hr
M35-B-hr
M75-B-hr
Level 3 (Merged & Unit-by-Unit)
MERGE-A
LOW-A
M35-A
M75-A
HIGH-A
MERGE-B
LOW-B
M35-B
M75-B
HIGH-B
Level 3 (Highrate & Histogram)
MERGE-A-hg
LOW-A-hg
M35-A-hg
M75-A-hg
HIGH-A-hg
MERGE-B-hg
LOW-B-hg
M35-B-hg
M75-B-hg
HIGH-B-hg
LOW-A-hr
M35-A-hr
M75-A-hr
LOW-B-hr
M35-B-hr
M75-B-hr
Oct-01 Oct-04 Oct-07 Oct-10 Oct-13 Oct-16 Oct-19 Oct-22 Oct-25 Oct-28 Oct-31
2013

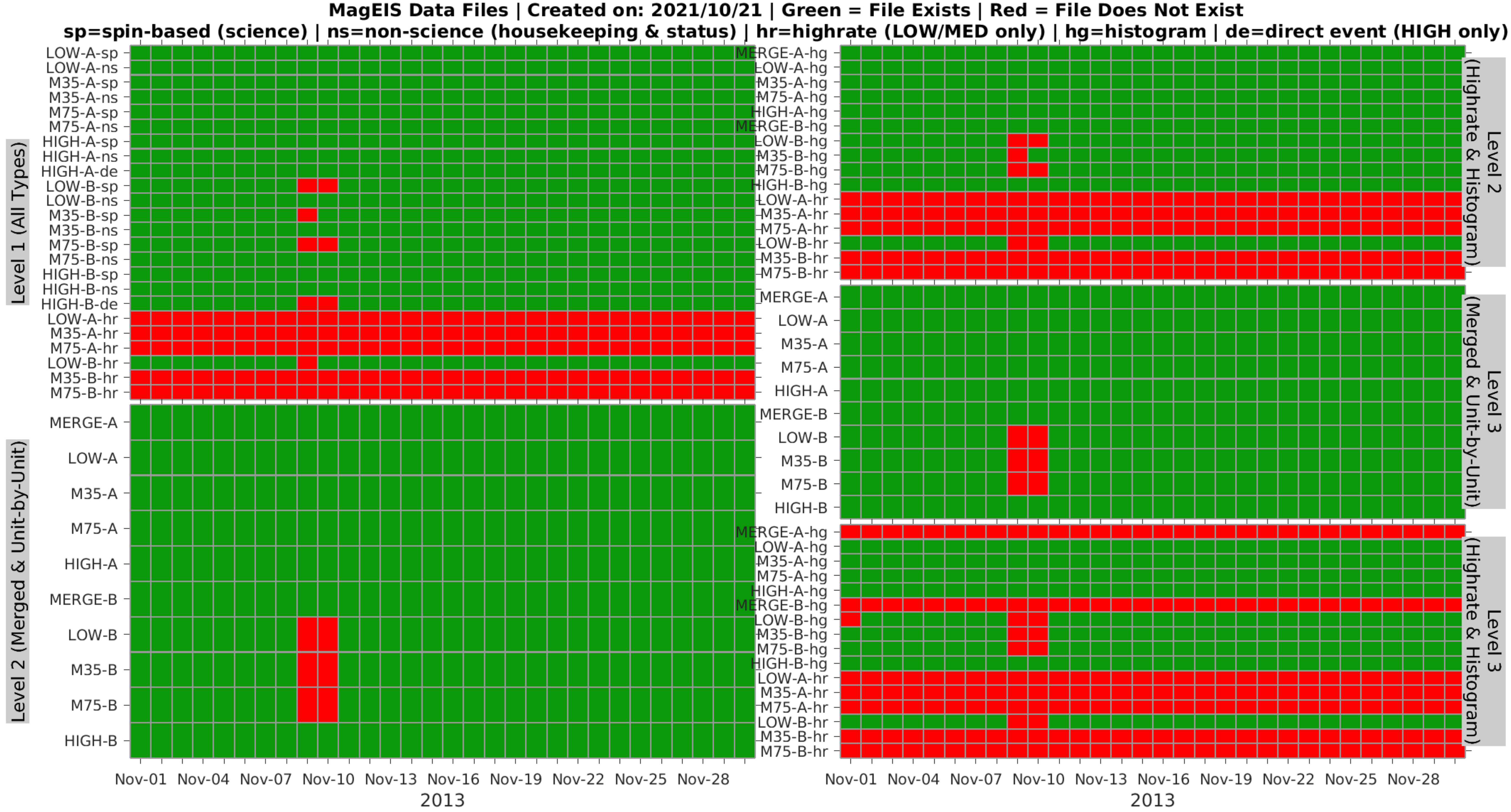

MagEIS Data Files | Created on: 2021/10/21 | Green = File Exists | Red = File Does Not Exist
sp=spin-based (science) | ns=non-science (housekeeping & status) | hr=highrate (LOW/MED only) | hg=histogram | de=direct event (HIGH only)
Level 1 (All Types)
LOW-A-sp
LOW-A-ns
M35-A-sp
M35-A-ns
M75-A-sp
M75-A-ns
HIGH-A-sp
HIGH-A-ns
HIGH-A-de
LOW-B-sp
LOW-B-ns
M35-B-sp
M35-B-ns
M75-B-sp
M75-B-ns
HIGH-B-sp
HIGH-B-ns
HIGH-B-de
LOW-A-hr
M35-A-hr
M75-A-hr
LOW-B-hr
M35-B-hr
M75-B-hr
Level 2 (Merged & Unit-by-Unit)
MERGE-A
LOW-A
M35-A
M75-A
HIGH-A
MERGE-B
LOW-B
M35-B
M75-B
HIGH-B
Level 2 (Highrate & Histogram)
MERGE-A-hg
LOW-A-hg
M35-A-hg
M75-A-hg
HIGH-A-hg
MERGE-B-hg
LOW-B-hg
M35-B-hg
M75-B-hg
HIGH-B-hg
LOW-A-hr
M35-A-hr
M75-A-hr
LOW-B-hr
M35-B-hr
M75-B-hr
Level 3 (Merged & Unit-by-Unit)
MERGE-A
LOW-A
M35-A
M75-A
HIGH-A
MERGE-B
LOW-B
M35-B
M75-B
HIGH-B
Level 3 (Highrate & Histogram)
MERGE-A-hg
LOW-A-hg
M35-A-hg
M75-A-hg
HIGH-A-hg
MERGE-B-hg
LOW-B-hg
M35-B-hg
M75-B-hg
HIGH-B-hg
LOW-A-hr
M35-A-hr
M75-A-hr
LOW-B-hr
M35-B-hr
M75-B-hr
Nov-01
Nov-04
Nov-07
Nov-10
Nov-13
Nov-16
Nov-19
Nov-22
Nov-25
Nov-28
2013

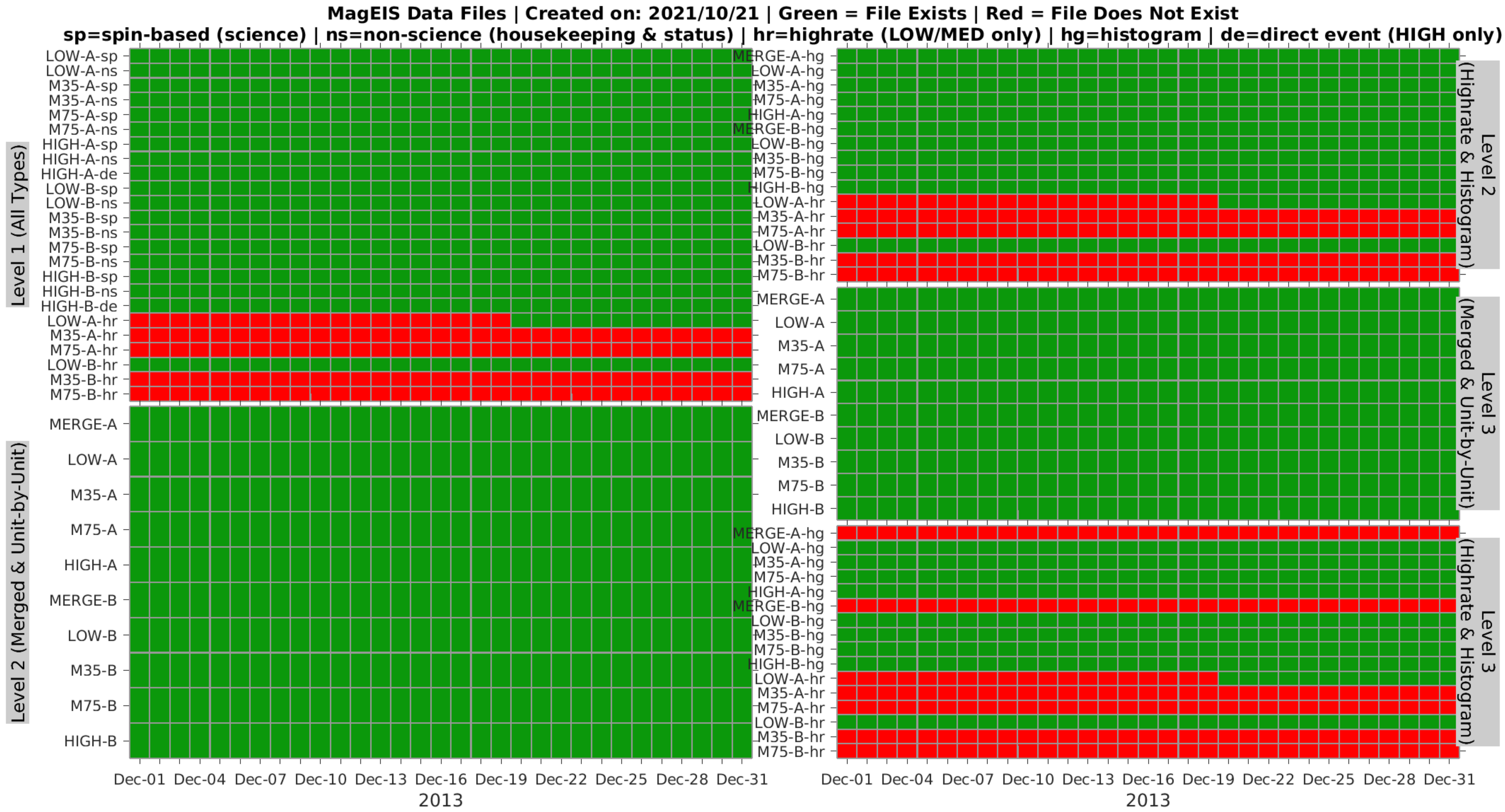
MagEIS Data Files | Created on: 2021/10/21 | Green = File Exists | Red = File Does Not Exist
sp=spin-based (science) | ns=non-science (housekeeping & status) | hr=highrate (LOW/MED only) | hg=histogram | de=direct event (HIGH only)
Level 1 (All Types)
LOW-A-sp
LOW-A-ns
M35-A-sp
M35-A-ns
M75-A-sp
M75-A-ns
HIGH-A-sp
HIGH-A-ns
HIGH-A-de
LOW-B-sp
LOW-B-ns
M35-B-sp
M35-B-ns
M75-B-sp
M75-B-ns
HIGH-B-sp
HIGH-B-ns
HIGH-B-de
LOW-A-hr
M35-A-hr
M75-A-hr
LOW-B-hr
M35-B-hr
M75-B-hr
Level 2 (Merged & Unit-by-Unit)
MERGE-A
LOW-A
M35-A
M75-A
HIGH-A
MERGE-B
LOW-B
M35-B
M75-B
HIGH-B
Level 2 (Highrate & Histogram)
MERGE-A-hg
LOW-A-hg
M35-A-hg
M75-A-hg
HIGH-A-hg
MERGE-B-hg
LOW-B-hg
M35-B-hg
M75-B-hg
HIGH-B-hg
LOW-A-hr
M35-A-hr
M75-A-hr
LOW-B-hr
M35-B-hr
M75-B-hr
Level 3 (Merged & Unit-by-Unit)
MERGE-A
LOW-A
M35-A
M75-A
HIGH-A
MERGE-B
LOW-B
M35-B
M75-B
HIGH-B
Level 3 (Highrate & Histogram)
MERGE-A-hg
LOW-A-hg
M35-A-hg
M75-A-hg
HIGH-A-hg
MERGE-B-hg
LOW-B-hg
M35-B-hg
M75-B-hg
HIGH-B-hg
LOW-A-hr
M35-A-hr
M75-A-hr
LOW-B-hr
M35-B-hr
M75-B-hr
Dec-01 Dec-04 Dec-07 Dec-10 Dec-13 Dec-16 Dec-19 Dec-22 Dec-25 Dec-28 Dec-31
2013
Dec-01 Dec-04 Dec-07 Dec-10 Dec-13 Dec-16 Dec-19 Dec-22 Dec-25 Dec-28 Dec-31
2013

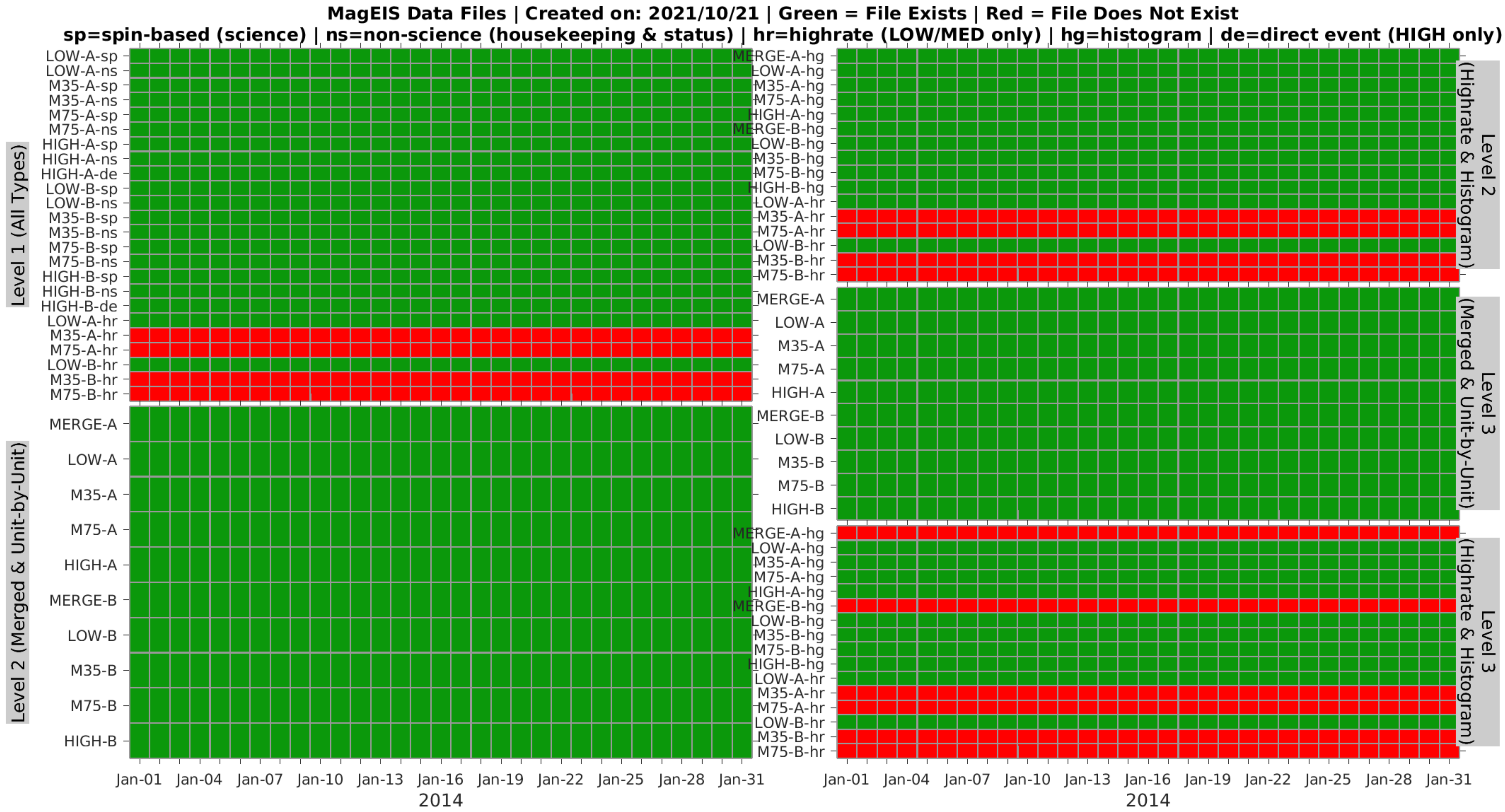
MagEIS Data Files | Created on: 2021/10/21 | Green = File Exists | Red = File Does Not Exist
sp=spin-based (science) | ns=non-science (housekeeping & status) | hr=highrate (LOW/MED only) | hg=histogram | de=direct event (HIGH only)
Level 1 (All Types)
LOW-A-sp
LOW-A-ns
M35-A-sp
M35-A-ns
M75-A-sp
M75-A-ns
HIGH-A-sp
HIGH-A-ns
HIGH-A-de
LOW-B-sp
LOW-B-ns
M35-B-sp
M35-B-ns
M75-B-sp
M75-B-ns
HIGH-B-sp
HIGH-B-ns
HIGH-B-de
LOW-A-hr
M35-A-hr
M75-A-hr
LOW-B-hr
M35-B-hr
M75-B-hr
Level 2 (Merged & Unit-by-Unit)
MERGE-A
LOW-A
M35-A
M75-A
HIGH-A
MERGE-B
LOW-B
M35-B
M75-B
HIGH-B
Level 2 (Highrate & Histogram)
MERGE-A-hg
LOW-A-hg
M35-A-hg
M75-A-hg
HIGH-A-hg
MERGE-B-hg
LOW-B-hg
M35-B-hg
M75-B-hg
HIGH-B-hg
LOW-A-hr
M35-A-hr
M75-A-hr
LOW-B-hr
M35-B-hr
M75-B-hr
Level 3 (Merged & Unit-by-Unit)
MERGE-A
LOW-A
M35-A
M75-A
HIGH-A
MERGE-B
LOW-B
M35-B
M75-B
HIGH-B
Level 3 (Highrate & Histogram)
MERGE-A-hg
LOW-A-hg
M35-A-hg
M75-A-hg
HIGH-A-hg
MERGE-B-hg
LOW-B-hg
M35-B-hg
M75-B-hg
HIGH-B-hg
LOW-A-hr
M35-A-hr
M75-A-hr
LOW-B-hr
M35-B-hr
M75-B-hr
Jan-01
Jan-04
Jan-07
Jan-10
Jan-13
Jan-16
Jan-19
Jan-22
Jan-25
Jan-28
Jan-31
2014

MagEIS Data Files | Created on: 2021/10/21 | Green = File Exists | Red = File Does Not Exist
sp=spin-based (science) | ns=non-science (housekeeping & status) | hr=highrate (LOW/MED only) | hg=histogram | de=direct event (HIGH only)

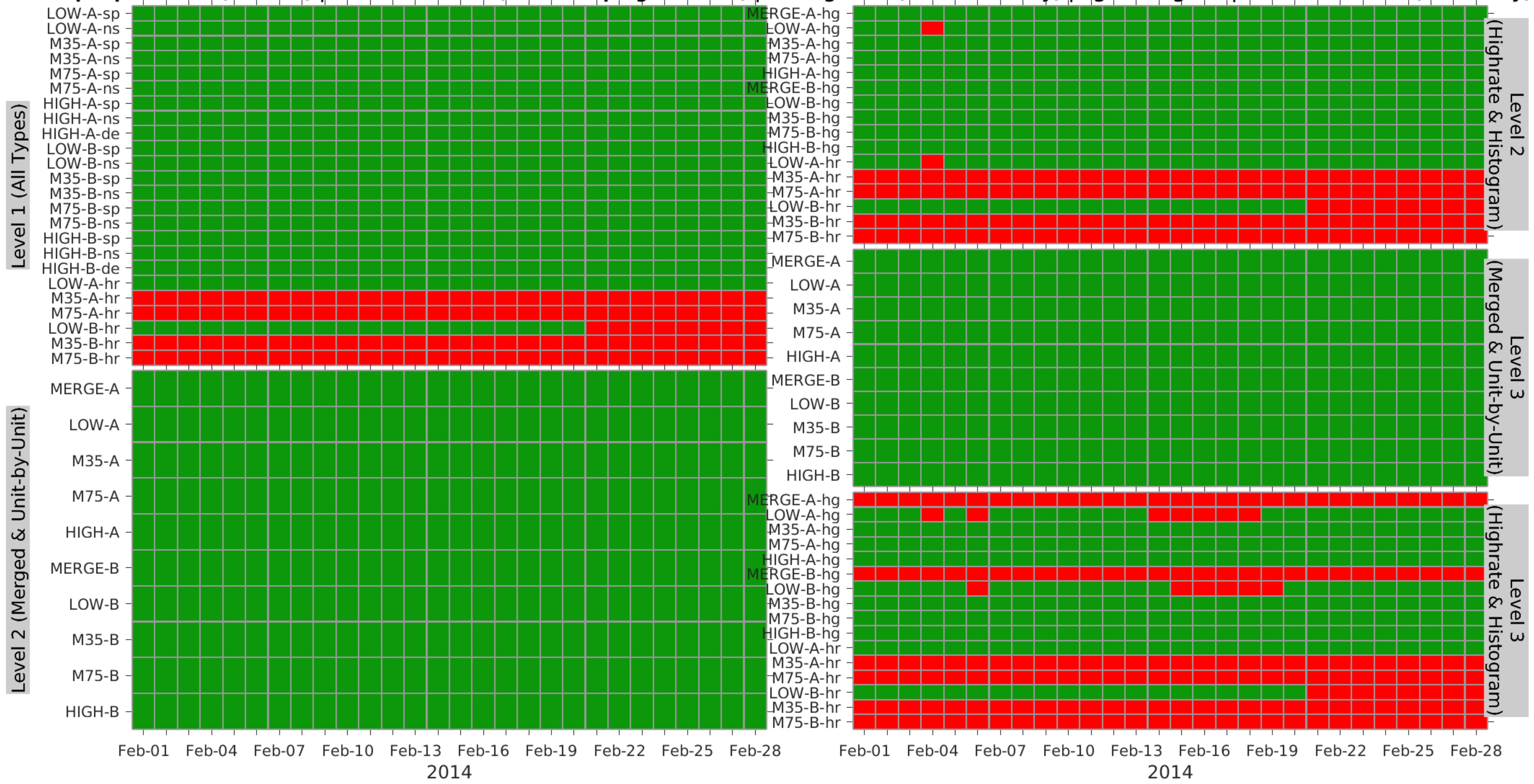

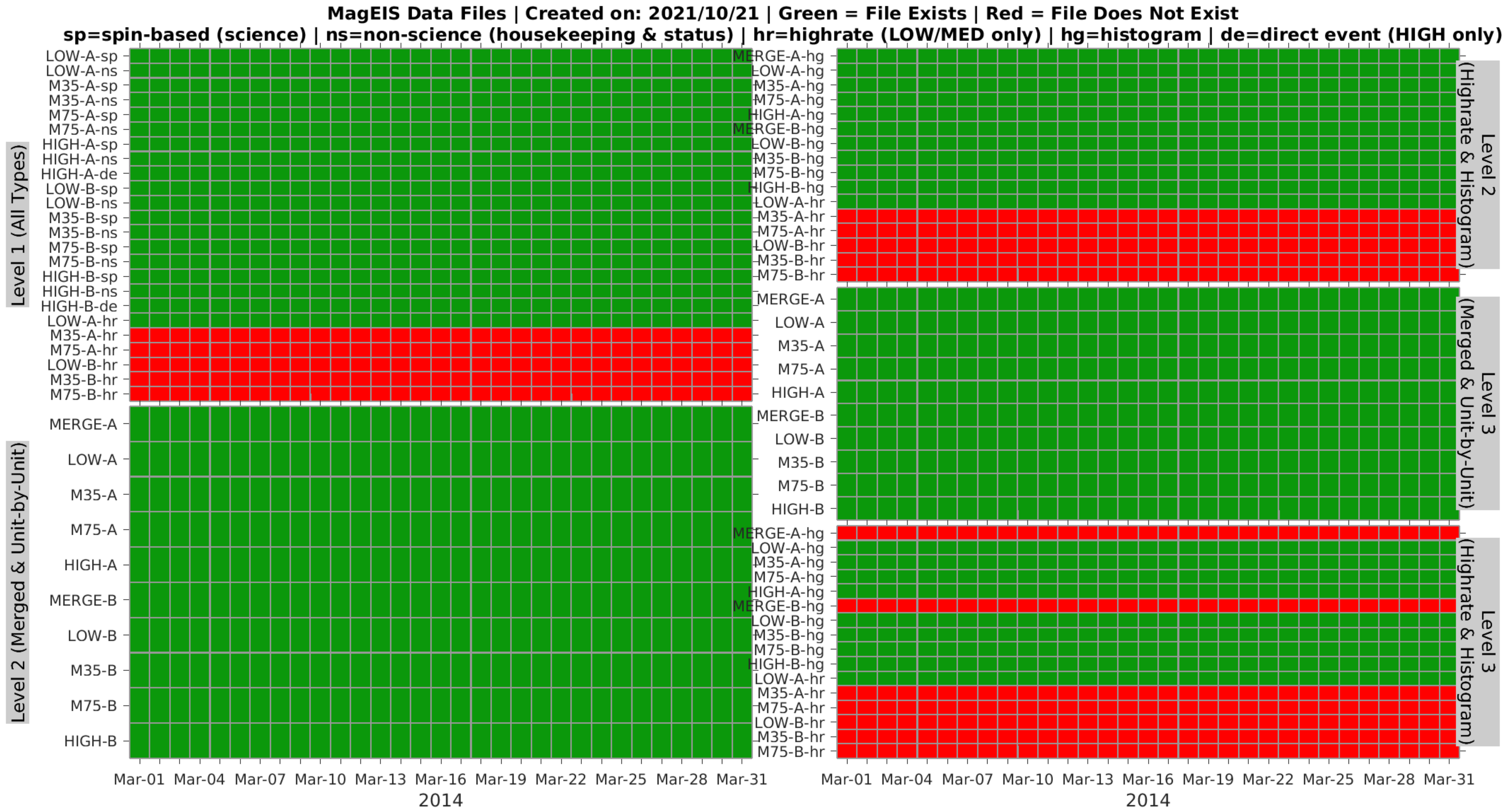

MagEIS Data Files | Created on: 2021/10/21 | Green = File Exists | Red = File Does Not Exist
sp=spin-based (science) | ns=non-science (housekeeping & status) | hr=highrate (LOW/MED only) | hg=histogram | de=direct event (HIGH only)
Level 1 (All Types)
LOW-A-sp
LOW-A-ns
M35-A-sp
M35-A-ns
M75-A-sp
M75-A-ns
HIGH-A-sp
HIGH-A-ns
HIGH-A-de
LOW-B-sp
LOW-B-ns
M35-B-sp
M35-B-ns
M75-B-sp
M75-B-ns
HIGH-B-sp
HIGH-B-ns
HIGH-B-de
LOW-A-hr
M35-A-hr
M75-A-hr
LOW-B-hr
M35-B-hr
M75-B-hr
Level 2 (Merged & Unit-by-Unit)
MERGE-A
LOW-A
M35-A
M75-A
HIGH-A
MERGE-B
LOW-B
M35-B
M75-B
HIGH-B
Level 2 (Highrate & Histogram)
MERGE-A-hg
LOW-A-hg
M35-A-hg
M75-A-hg
HIGH-A-hg
MERGE-B-hg
LOW-B-hg
M35-B-hg
M75-B-hg
HIGH-B-hg
LOW-A-hr
M35-A-hr
M75-A-hr
LOW-B-hr
M35-B-hr
M75-B-hr
Level 3 (Merged & Unit-by-Unit)
MERGE-A
LOW-A
M35-A
M75-A
HIGH-A
MERGE-B
LOW-B
M35-B
M75-B
HIGH-B
Level 3 (Highrate & Histogram)
MERGE-A-hg
LOW-A-hg
M35-A-hg
M75-A-hg
HIGH-A-hg
MERGE-B-hg
LOW-B-hg
M35-B-hg
M75-B-hg
HIGH-B-hg
LOW-A-hr
M35-A-hr
M75-A-hr
LOW-B-hr
M35-B-hr
M75-B-hr
Mar-01
Mar-04
Mar-07
Mar-10
Mar-13
Mar-16
Mar-19
Mar-22
Mar-25
Mar-28
Mar-31
2014

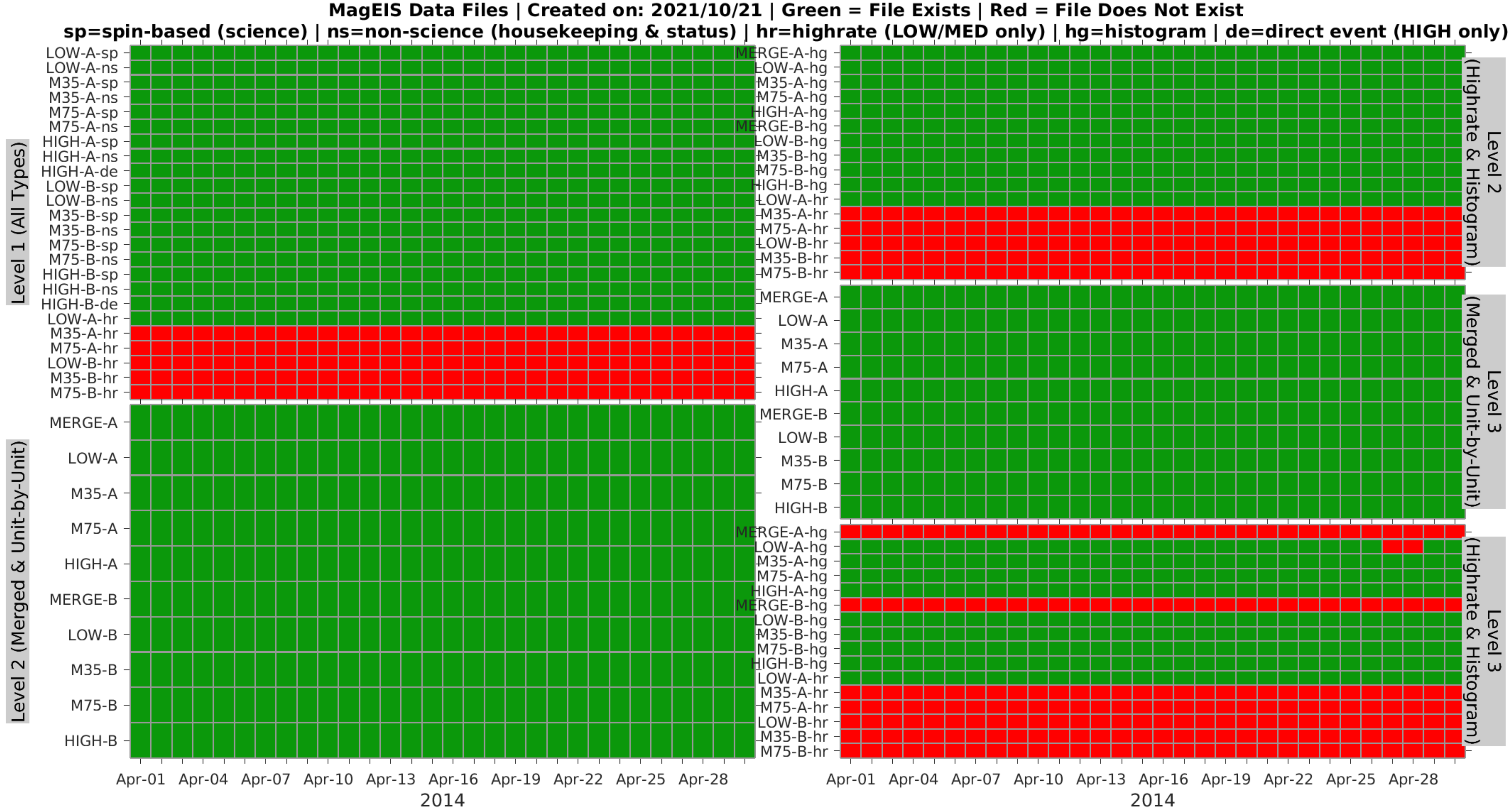

MagEIS Data Files | Created on: 2021/10/21 | Green = File Exists | Red = File Does Not Exist
sp=spin-based (science) | ns=non-science (housekeeping & status) | hr=highrate (LOW/MED only) | hg=histogram | de=direct event (HIGH only)
Level 1 (All Types)
LOW-A-sp
LOW-A-ns
M35-A-sp
M35-A-ns
M75-A-sp
M75-A-ns
HIGH-A-sp
HIGH-A-ns
HIGH-A-de
LOW-B-sp
LOW-B-ns
M35-B-sp
M35-B-ns
M75-B-sp
M75-B-ns
HIGH-B-sp
HIGH-B-ns
HIGH-B-de
LOW-A-hr
M35-A-hr
M75-A-hr
LOW-B-hr
M35-B-hr
M75-B-hr
Level 2 (Merged & Unit-by-Unit)
MERGE-A
LOW-A
M35-A
M75-A
HIGH-A
MERGE-B
LOW-B
M35-B
M75-B
HIGH-B
Level 2 (Highrate & Histogram)
MERGE-A-hg
LOW-A-hg
M35-A-hg
M75-A-hg
HIGH-A-hg
MERGE-B-hg
LOW-B-hg
M35-B-hg
M75-B-hg
HIGH-B-hg
LOW-A-hr
M35-A-hr
M75-A-hr
LOW-B-hr
M35-B-hr
M75-B-hr
Level 3 (Merged & Unit-by-Unit)
MERGE-A
LOW-A
M35-A
M75-A
HIGH-A
MERGE-B
LOW-B
M35-B
M75-B
HIGH-B
Level 3 (Highrate & Histogram)
MERGE-A-hg
LOW-A-hg
M35-A-hg
M75-A-hg
HIGH-A-hg
MERGE-B-hg
LOW-B-hg
M35-B-hg
M75-B-hg
HIGH-B-hg
LOW-A-hr
M35-A-hr
M75-A-hr
LOW-B-hr
M35-B-hr
M75-B-hr
Apr-01
Apr-04
Apr-07
Apr-10
Apr-13
Apr-16
Apr-19
Apr-22
Apr-25
Apr-28
2014

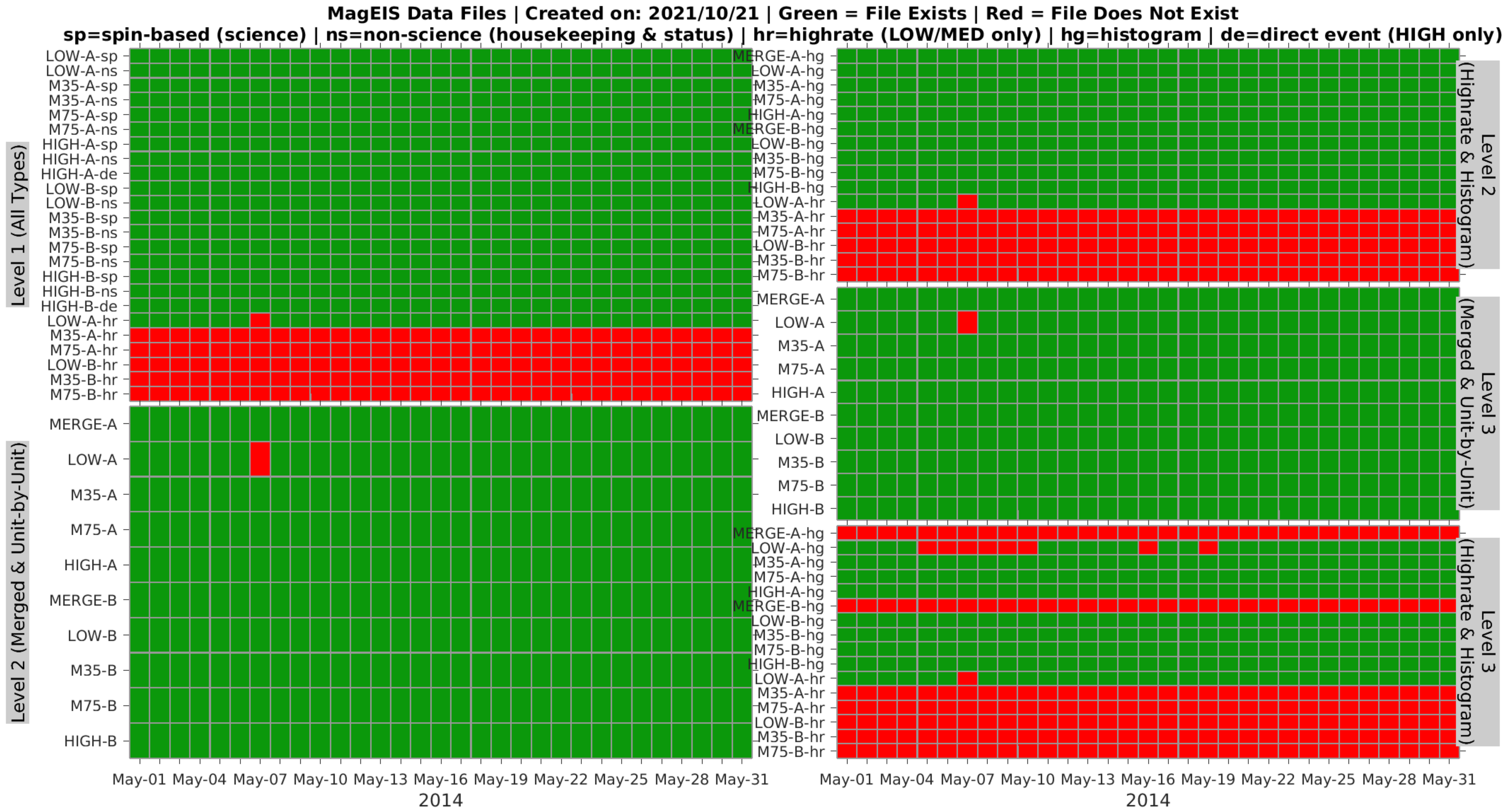

MagEIS Data Files | Created on: 2021/10/21 | Green = File Exists | Red = File Does Not Exist
sp=spin-based (science) | ns=non-science (housekeeping & status) | hr=highrate (LOW/MED only) | hg=histogram | de=direct event (HIGH only)
Level 1 (All Types)
LOW-A-sp
LOW-A-ns
M35-A-sp
M35-A-ns
M75-A-sp
M75-A-ns
HIGH-A-sp
HIGH-A-ns
HIGH-A-de
LOW-B-sp
LOW-B-ns
M35-B-sp
M35-B-ns
M75-B-sp
M75-B-ns
HIGH-B-sp
HIGH-B-ns
HIGH-B-de
LOW-A-hr
M35-A-hr
M75-A-hr
LOW-B-hr
M35-B-hr
M75-B-hr
Level 2 (Merged & Unit-by-Unit)
MERGE-A
LOW-A
M35-A
M75-A
HIGH-A
MERGE-B
LOW-B
M35-B
M75-B
HIGH-B
May-01
May-04
May-07
May-10
May-13
May-16
May-19
May-22
May-25
May-28
May-31
2014
Level 2 (Highrate & Histogram)
MERGE-A-hg
LOW-A-hg
M35-A-hg
M75-A-hg
HIGH-A-hg
MERGE-B-hg
LOW-B-hg
M35-B-hg
M75-B-hg
HIGH-B-hg
LOW-A-hr
M35-A-hr
M75-A-hr
LOW-B-hr
M35-B-hr
M75-B-hr
Level 3 (Merged & Unit-by-Unit)
MERGE-A
LOW-A
M35-A
M75-A
HIGH-A
MERGE-B
LOW-B
M35-B
M75-B
HIGH-B
Level 3 (Highrate & Histogram)
MERGE-A-hg
LOW-A-hg
M35-A-hg
M75-A-hg
HIGH-A-hg
MERGE-B-hg
LOW-B-hg
M35-B-hg
M75-B-hg
HIGH-B-hg
LOW-A-hr
M35-A-hr
M75-A-hr
LOW-B-hr
M35-B-hr
M75-B-hr
May-01
May-04
May-07
May-10
May-13
May-16
May-19
May-22
May-25
May-28
May-31
2014

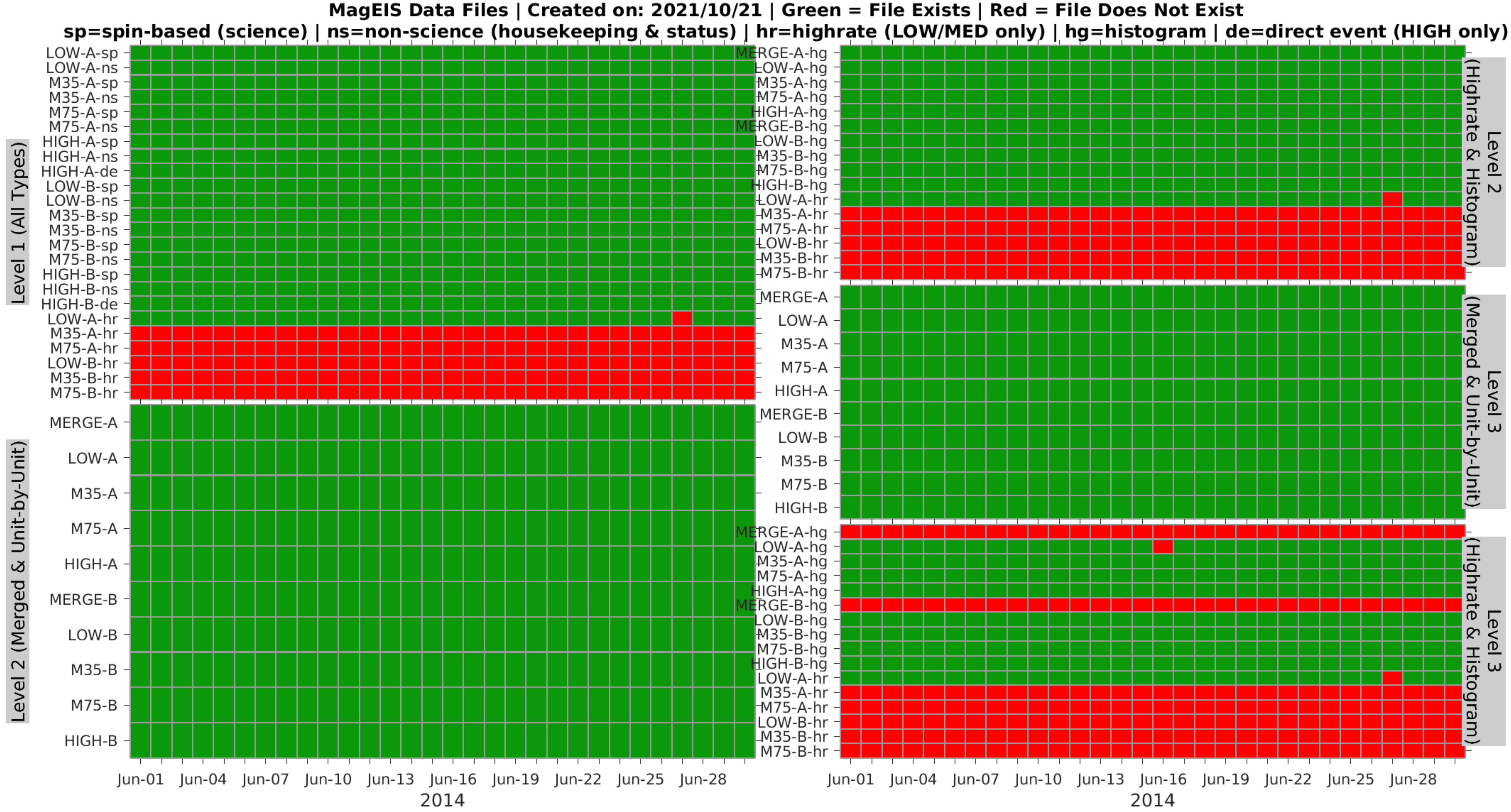
MagEIS Data Files | Created on: 2021/10/21 | Green = File Exists | Red = File Does Not Exist
sp=spin-based (science) | ns=non-science (housekeeping & status) | hr=highrate (LOW/MED only) | hg=histogram | de=direct event (HIGH only)
Level 1 (All Types)
LOW-A-sp
LOW-A-ns
M35-A-sp
M35-A-ns
M75-A-sp
M75-A-ns
HIGH-A-sp
HIGH-A-ns
HIGH-A-de
LOW-B-sp
LOW-B-ns
M35-B-sp
M35-B-ns
M75-B-sp
M75-B-ns
HIGH-B-sp
HIGH-B-ns
HIGH-B-de
LOW-A-hr
M35-A-hr
M75-A-hr
LOW-B-hr
M35-B-hr
M75-B-hr
Level 2 (Merged & Unit-by-Unit)
MERGE-A
LOW-A
M35-A
M75-A
HIGH-A
MERGE-B
LOW-B
M35-B
M75-B
HIGH-B
Jun-01
Jun-04
Jun-07
Jun-10
Jun-13
Jun-16
Jun-19
Jun-22
Jun-25
Jun-28
2014
Level 2 (Highrate & Histogram)
MERGE-A-hg
LOW-A-hg
M35-A-hg
M75-A-hg
HIGH-A-hg
MERGE-B-hg
LOW-B-hg
M35-B-hg
M75-B-hg
HIGH-B-hg
LOW-A-hr
M35-A-hr
M75-A-hr
LOW-B-hr
M35-B-hr
M75-B-hr
Level 3 (Merged & Unit-by-Unit)
MERGE-A
LOW-A
M35-A
M75-A
HIGH-A
MERGE-B
LOW-B
M35-B
M75-B
HIGH-B
Level 3 (Highrate & Histogram)
MERGE-A-hg
LOW-A-hg
M35-A-hg
M75-A-hg
HIGH-A-hg
MERGE-B-hg
LOW-B-hg
M35-B-hg
M75-B-hg
HIGH-B-hg
LOW-A-hr
M35-A-hr
M75-A-hr
LOW-B-hr
M35-B-hr
M75-B-hr
Jun-01
Jun-04
Jun-07
Jun-10
Jun-13
Jun-16
Jun-19
Jun-22
Jun-25
Jun-28
2014

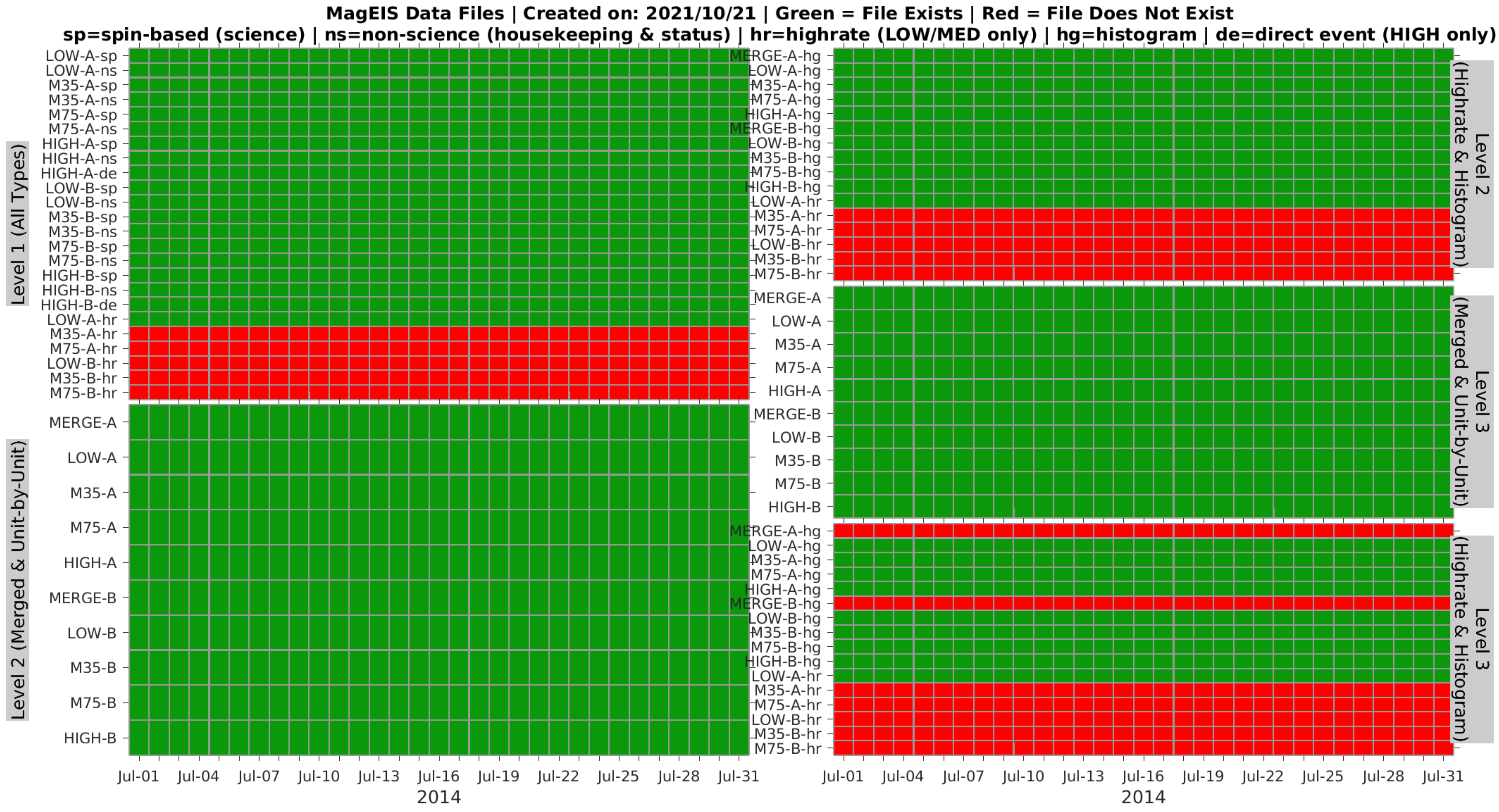
MagEIS Data Files | Created on: 2021/10/21 | Green = File Exists | Red = File Does Not Exist
sp=spin-based (science) | ns=non-science (housekeeping & status) | hr=highrate (LOW/MED only) | hg=histogram | de=direct event (HIGH only)
Level 1 (All Types)
LOW-A-sp
LOW-A-ns
M35-A-sp
M35-A-ns
M75-A-sp
M75-A-ns
HIGH-A-sp
HIGH-A-ns
HIGH-A-de
LOW-B-sp
LOW-B-ns
M35-B-sp
M35-B-ns
M75-B-sp
M75-B-ns
HIGH-B-sp
HIGH-B-ns
HIGH-B-de
LOW-A-hr
M35-A-hr
M75-A-hr
LOW-B-hr
M35-B-hr
M75-B-hr
Level 2 (Merged & Unit-by-Unit)
MERGE-A
LOW-A
M35-A
M75-A
HIGH-A
MERGE-B
LOW-B
M35-B
M75-B
HIGH-B
Jul-01
Jul-04
Jul-07
Jul-10
Jul-13
Jul-16
Jul-19
Jul-22
Jul-25
Jul-28
Jul-31
2014
Level 2 (Highrate & Histogram)
MERGE-A-hg
LOW-A-hg
M35-A-hg
M75-A-hg
HIGH-A-hg
MERGE-B-hg
LOW-B-hg
M35-B-hg
M75-B-hg
HIGH-B-hg
LOW-A-hr
M35-A-hr
M75-A-hr
LOW-B-hr
M35-B-hr
M75-B-hr
Level 3 (Merged & Unit-by-Unit)
MERGE-A
LOW-A
M35-A
M75-A
HIGH-A
MERGE-B
LOW-B
M35-B
M75-B
HIGH-B
Level 3 (Highrate & Histogram)
MERGE-A-hg
LOW-A-hg
M35-A-hg
M75-A-hg
HIGH-A-hg
MERGE-B-hg
LOW-B-hg
M35-B-hg
M75-B-hg
HIGH-B-hg
LOW-A-hr
M35-A-hr
M75-A-hr
LOW-B-hr
M35-B-hr
M75-B-hr
Jul-01
Jul-04
Jul-07
Jul-10
Jul-13
Jul-16
Jul-19
Jul-22
Jul-25
Jul-28
Jul-31
2014

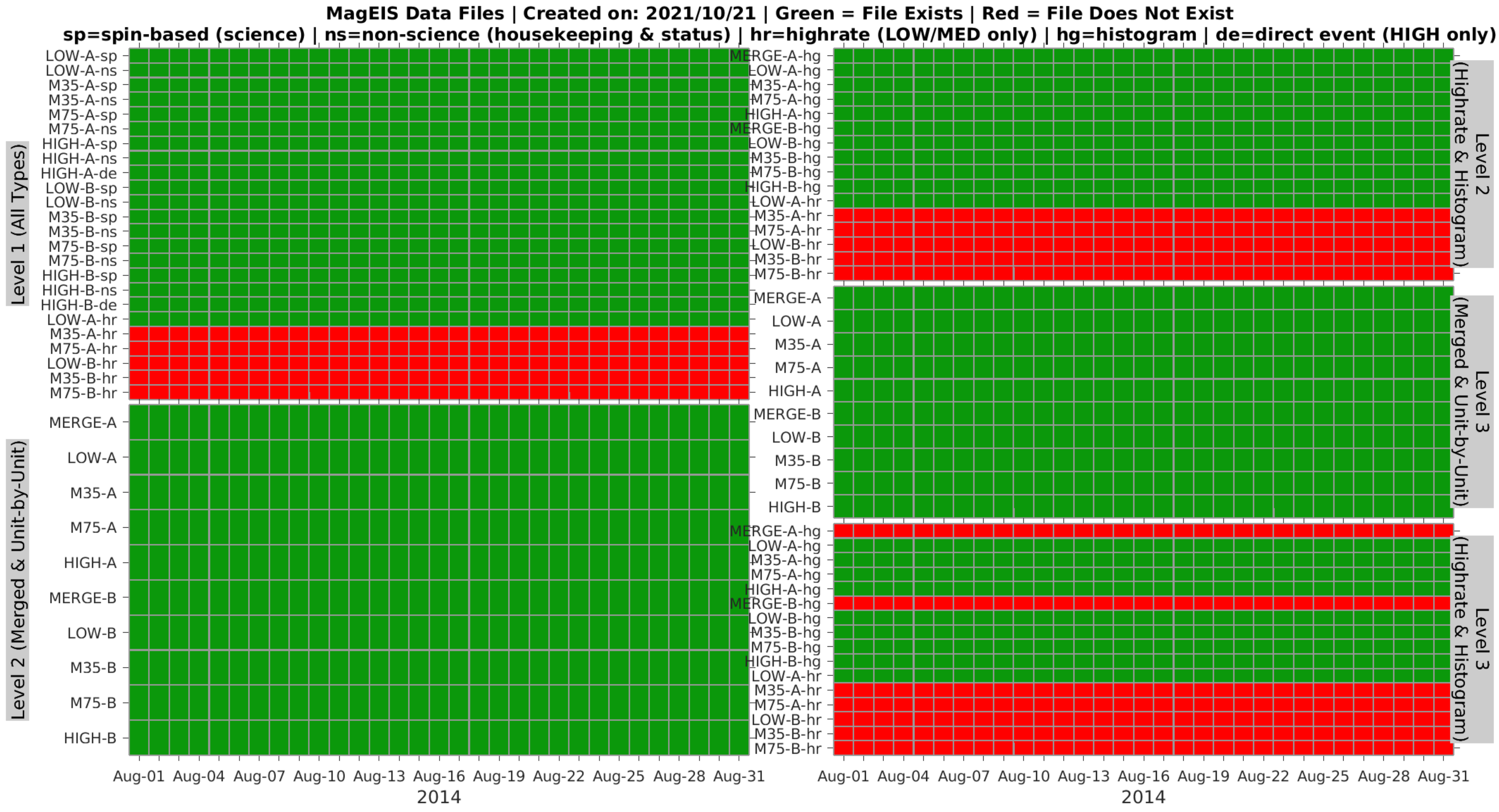
MagEIS Data Files | Created on: 2021/10/21 | Green = File Exists | Red = File Does Not Exist
sp=spin-based (science) | ns=non-science (housekeeping & status) | hr=highrate (LOW/MED only) | hg=histogram | de=direct event (HIGH only)
Level 1 (All Types)
LOW-A-sp
LOW-A-ns
M35-A-sp
M35-A-ns
M75-A-sp
M75-A-ns
HIGH-A-sp
HIGH-A-ns
HIGH-A-de
LOW-B-sp
LOW-B-ns
M35-B-sp
M35-B-ns
M75-B-sp
M75-B-ns
HIGH-B-sp
HIGH-B-ns
HIGH-B-de
LOW-A-hr
M35-A-hr
M75-A-hr
LOW-B-hr
M35-B-hr
M75-B-hr
Level 2 (Merged & Unit-by-Unit)
MERGE-A
LOW-A
M35-A
M75-A
HIGH-A
MERGE-B
LOW-B
M35-B
M75-B
HIGH-B
Level 2 (Highrate & Histogram)
MERGE-A-hg
LOW-A-hg
M35-A-hg
M75-A-hg
HIGH-A-hg
MERGE-B-hg
LOW-B-hg
M35-B-hg
M75-B-hg
HIGH-B-hg
LOW-A-hr
M35-A-hr
M75-A-hr
LOW-B-hr
M35-B-hr
M75-B-hr
Level 3 (Merged & Unit-by-Unit)
MERGE-A
LOW-A
M35-A
M75-A
HIGH-A
MERGE-B
LOW-B
M35-B
M75-B
HIGH-B
Level 3 (Highrate & Histogram)
MERGE-A-hg
LOW-A-hg
M35-A-hg
M75-A-hg
HIGH-A-hg
MERGE-B-hg
LOW-B-hg
M35-B-hg
M75-B-hg
HIGH-B-hg
LOW-A-hr
M35-A-hr
M75-A-hr
LOW-B-hr
M35-B-hr
M75-B-hr
Aug-01
Aug-04
Aug-07
Aug-10
Aug-13
Aug-16
Aug-19
Aug-22
Aug-25
Aug-28
Aug-31
2014

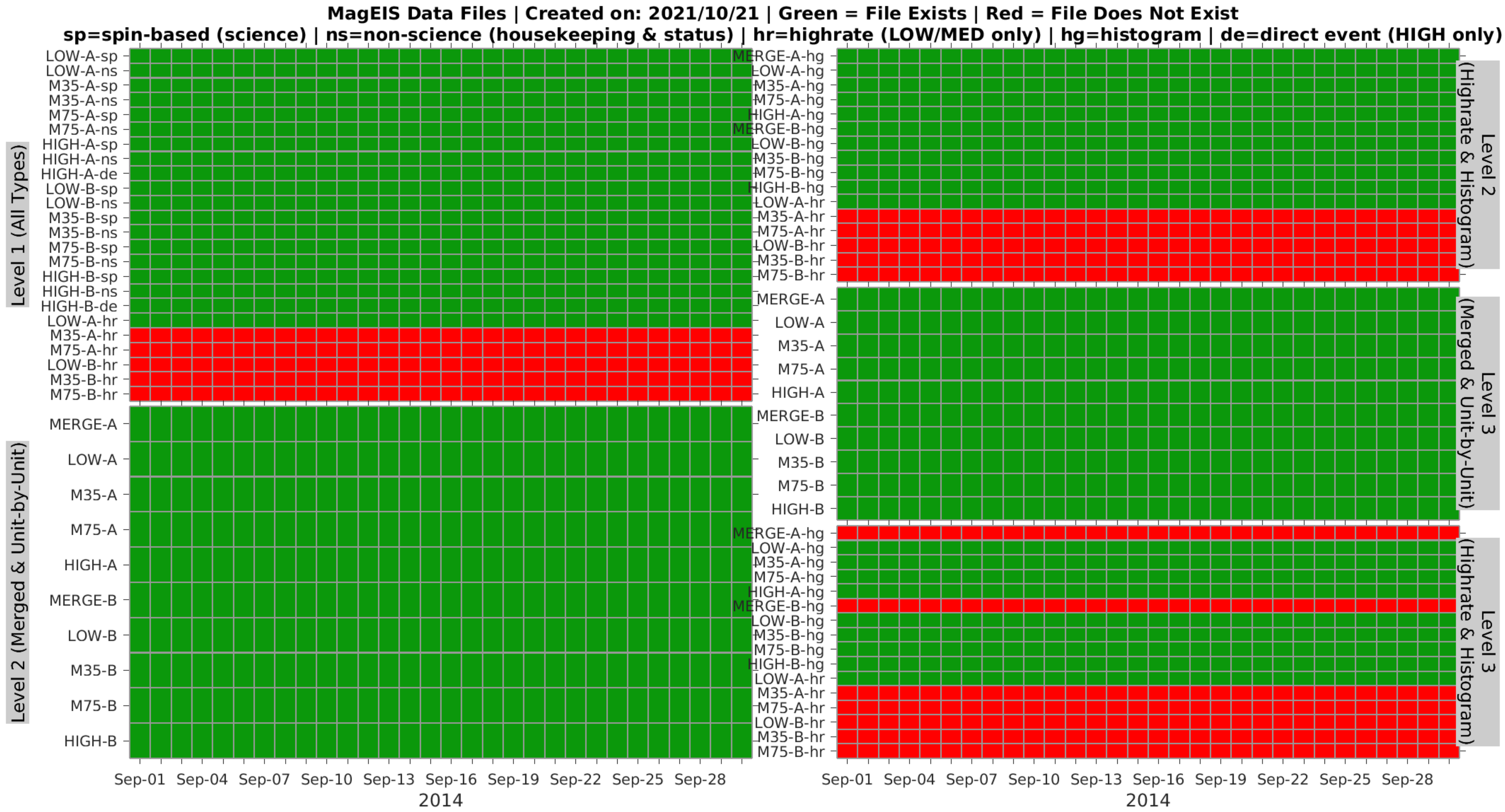
MagEIS Data Files | Created on: 2021/10/21 | Green = File Exists | Red = File Does Not Exist
sp=spin-based (science) | ns=non-science (housekeeping & status) | hr=highrate (LOW/MED only) | hg=histogram | de=direct event (HIGH only)
Level 1 (All Types)
LOW-A-sp
LOW-A-ns
M35-A-sp
M35-A-ns
M75-A-sp
M75-A-ns
HIGH-A-sp
HIGH-A-ns
HIGH-A-de
LOW-B-sp
LOW-B-ns
M35-B-sp
M35-B-ns
M75-B-sp
M75-B-ns
HIGH-B-sp
HIGH-B-ns
HIGH-B-de
LOW-A-hr
M35-A-hr
M75-A-hr
LOW-B-hr
M35-B-hr
M75-B-hr
Level 2 (Merged & Unit-by-Unit)
MERGE-A
LOW-A
M35-A
M75-A
HIGH-A
MERGE-B
LOW-B
M35-B
M75-B
HIGH-B
Level 2 (Highrate & Histogram)
MERGE-A-hg
LOW-A-hg
M35-A-hg
M75-A-hg
HIGH-A-hg
MERGE-B-hg
LOW-B-hg
M35-B-hg
M75-B-hg
HIGH-B-hg
LOW-A-hr
M35-A-hr
M75-A-hr
LOW-B-hr
M35-B-hr
M75-B-hr
Level 3 (Merged & Unit-by-Unit)
MERGE-A
LOW-A
M35-A
M75-A
HIGH-A
MERGE-B
LOW-B
M35-B
M75-B
HIGH-B
Level 3 (Highrate & Histogram)
MERGE-A-hg
LOW-A-hg
M35-A-hg
M75-A-hg
HIGH-A-hg
MERGE-B-hg
LOW-B-hg
M35-B-hg
M75-B-hg
HIGH-B-hg
LOW-A-hr
M35-A-hr
M75-A-hr
LOW-B-hr
M35-B-hr
M75-B-hr
Sep-01
Sep-04
Sep-07
Sep-10
Sep-13
Sep-16
Sep-19
Sep-22
Sep-25
Sep-28
2014

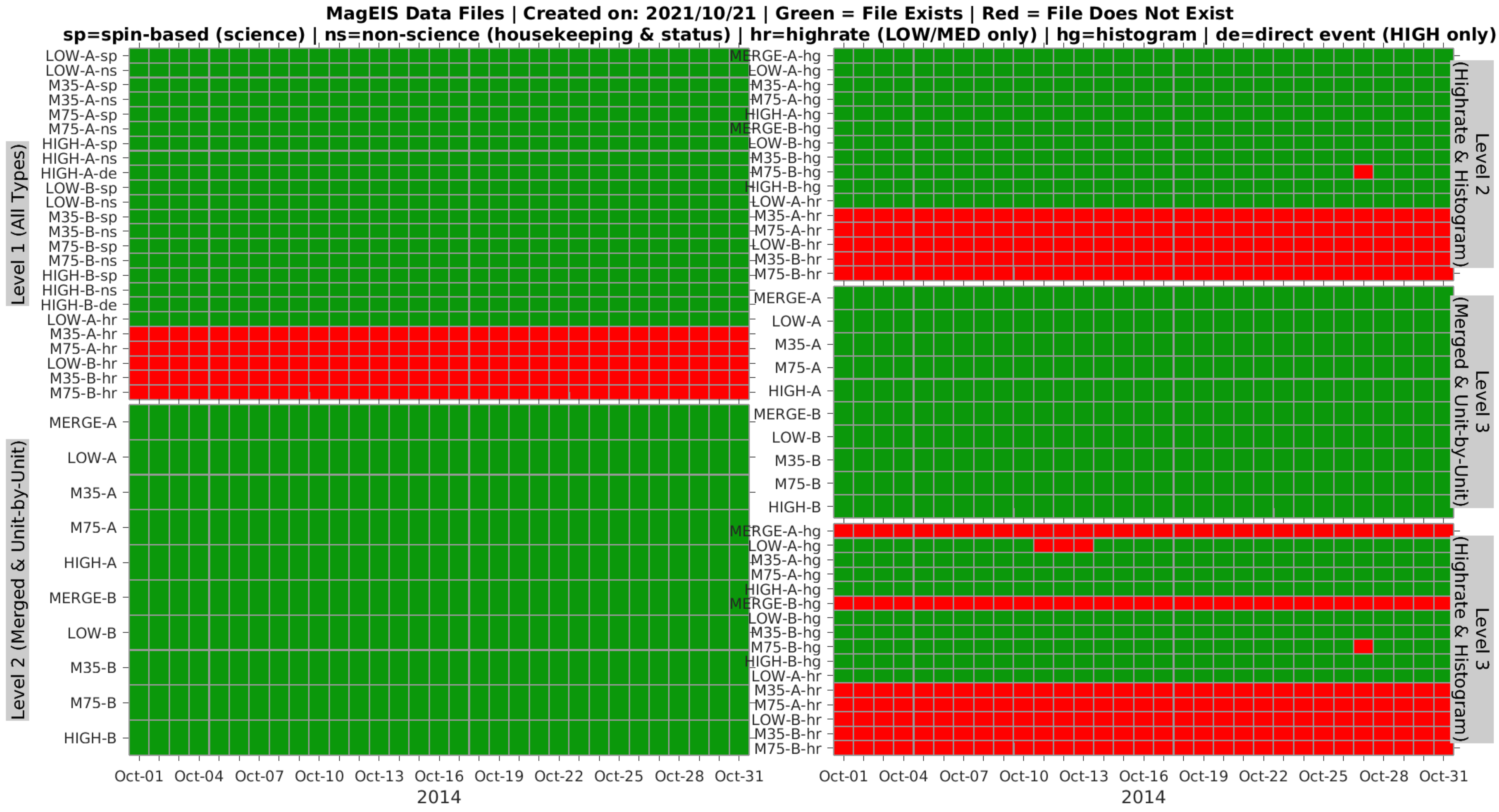
MagEIS Data Files | Created on: 2021/10/21 | Green = File Exists | Red = File Does Not Exist
sp=spin-based (science) | ns=non-science (housekeeping & status) | hr=highrate (LOW/MED only) | hg=histogram | de=direct event (HIGH only)
Level 1 (All Types)
LOW-A-sp
LOW-A-ns
M35-A-sp
M35-A-ns
M75-A-sp
M75-A-ns
HIGH-A-sp
HIGH-A-ns
HIGH-A-de
LOW-B-sp
LOW-B-ns
M35-B-sp
M35-B-ns
M75-B-sp
M75-B-ns
HIGH-B-sp
HIGH-B-ns
HIGH-B-de
LOW-A-hr
M35-A-hr
M75-A-hr
LOW-B-hr
M35-B-hr
M75-B-hr
Level 2 (Merged & Unit-by-Unit)
MERGE-A
LOW-A
M35-A
M75-A
HIGH-A
MERGE-B
LOW-B
M35-B
M75-B
HIGH-B
Level 2 (Highrate & Histogram)
MERGE-A-hg
LOW-A-hg
M35-A-hg
M75-A-hg
HIGH-A-hg
MERGE-B-hg
LOW-B-hg
M35-B-hg
M75-B-hg
HIGH-B-hg
LOW-A-hr
M35-A-hr
M75-A-hr
LOW-B-hr
M35-B-hr
M75-B-hr
Level 3 (Merged & Unit-by-Unit)
MERGE-A
LOW-A
M35-A
M75-A
HIGH-A
MERGE-B
LOW-B
M35-B
M75-B
HIGH-B
Level 3 (Highrate & Histogram)
MERGE-A-hg
LOW-A-hg
M35-A-hg
M75-A-hg
HIGH-A-hg
MERGE-B-hg
LOW-B-hg
M35-B-hg
M75-B-hg
HIGH-B-hg
LOW-A-hr
M35-A-hr
M75-A-hr
LOW-B-hr
M35-B-hr
M75-B-hr
Oct-01 Oct-04 Oct-07 Oct-10 Oct-13 Oct-16 Oct-19 Oct-22 Oct-25 Oct-28 Oct-31
2014

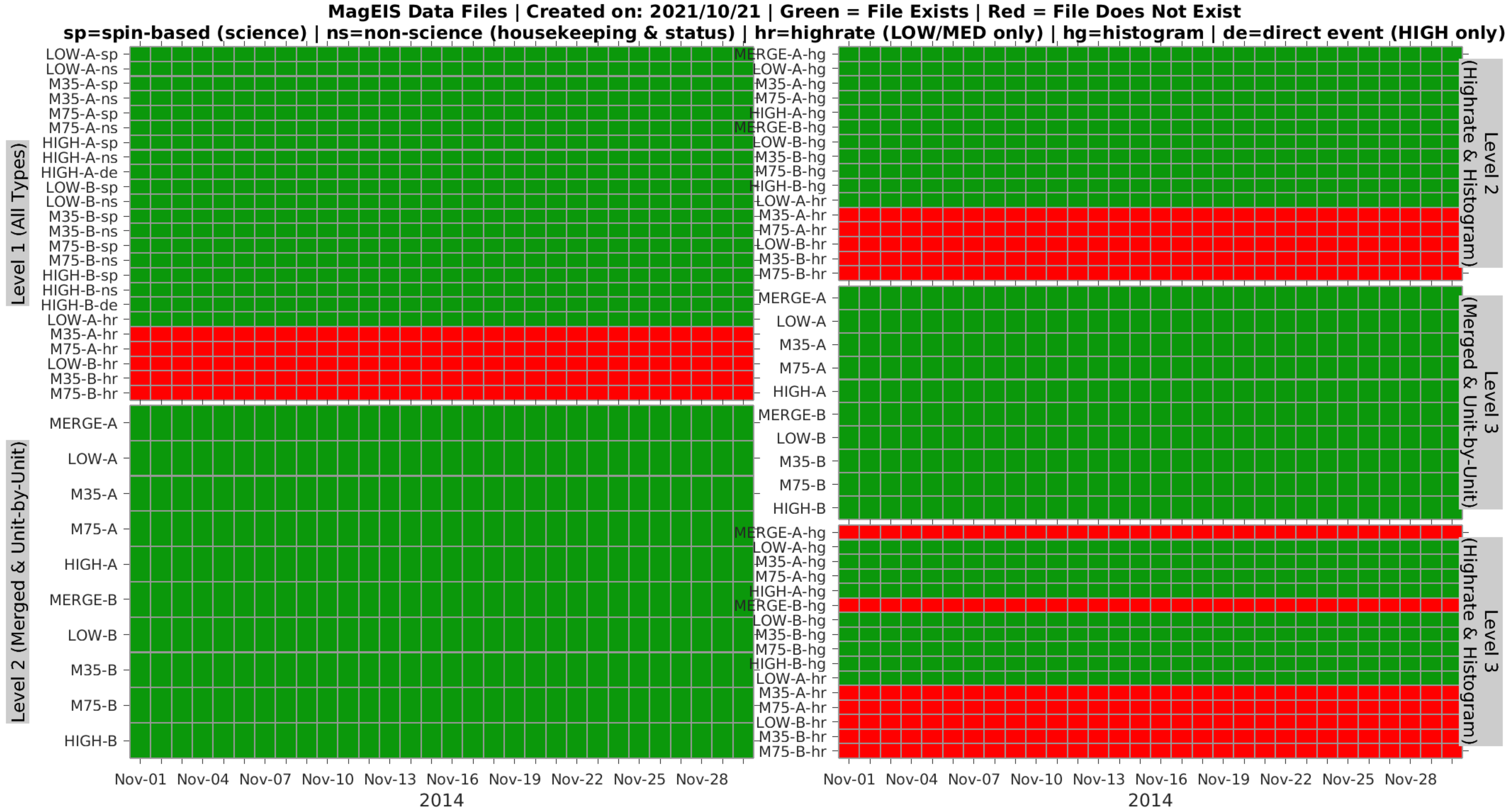

MagEIS Data Files | Created on: 2021/10/21 | Green = File Exists | Red = File Does Not Exist
sp=spin-based (science) | ns=non-science (housekeeping & status) | hr=highrate (LOW/MED only) | hg=histogram | de=direct event (HIGH only)
Level 1 (All Types)
LOW-A-sp
LOW-A-ns
M35-A-sp
M35-A-ns
M75-A-sp
M75-A-ns
HIGH-A-sp
HIGH-A-ns
HIGH-A-de
LOW-B-sp
LOW-B-ns
M35-B-sp
M35-B-ns
M75-B-sp
M75-B-ns
HIGH-B-sp
HIGH-B-ns
HIGH-B-de
LOW-A-hr
M35-A-hr
M75-A-hr
LOW-B-hr
M35-B-hr
M75-B-hr
Level 2 (Merged & Unit-by-Unit)
MERGE-A
LOW-A
M35-A
M75-A
HIGH-A
MERGE-B
LOW-B
M35-B
M75-B
HIGH-B
Level 2 (Highrate & Histogram)
MERGE-A-hg
LOW-A-hg
M35-A-hg
M75-A-hg
HIGH-A-hg
MERGE-B-hg
LOW-B-hg
M35-B-hg
M75-B-hg
HIGH-B-hg
LOW-A-hr
M35-A-hr
M75-A-hr
LOW-B-hr
M35-B-hr
M75-B-hr
Level 3 (Merged & Unit-by-Unit)
MERGE-A
LOW-A
M35-A
M75-A
HIGH-A
MERGE-B
LOW-B
M35-B
M75-B
HIGH-B
Level 3 (Highrate & Histogram)
MERGE-A-hg
LOW-A-hg
M35-A-hg
M75-A-hg
HIGH-A-hg
MERGE-B-hg
LOW-B-hg
M35-B-hg
M75-B-hg
HIGH-B-hg
LOW-A-hr
M35-A-hr
M75-A-hr
LOW-B-hr
M35-B-hr
M75-B-hr
Nov-01
Nov-04
Nov-07
Nov-10
Nov-13
Nov-16
Nov-19
Nov-22
Nov-25
Nov-28
2014

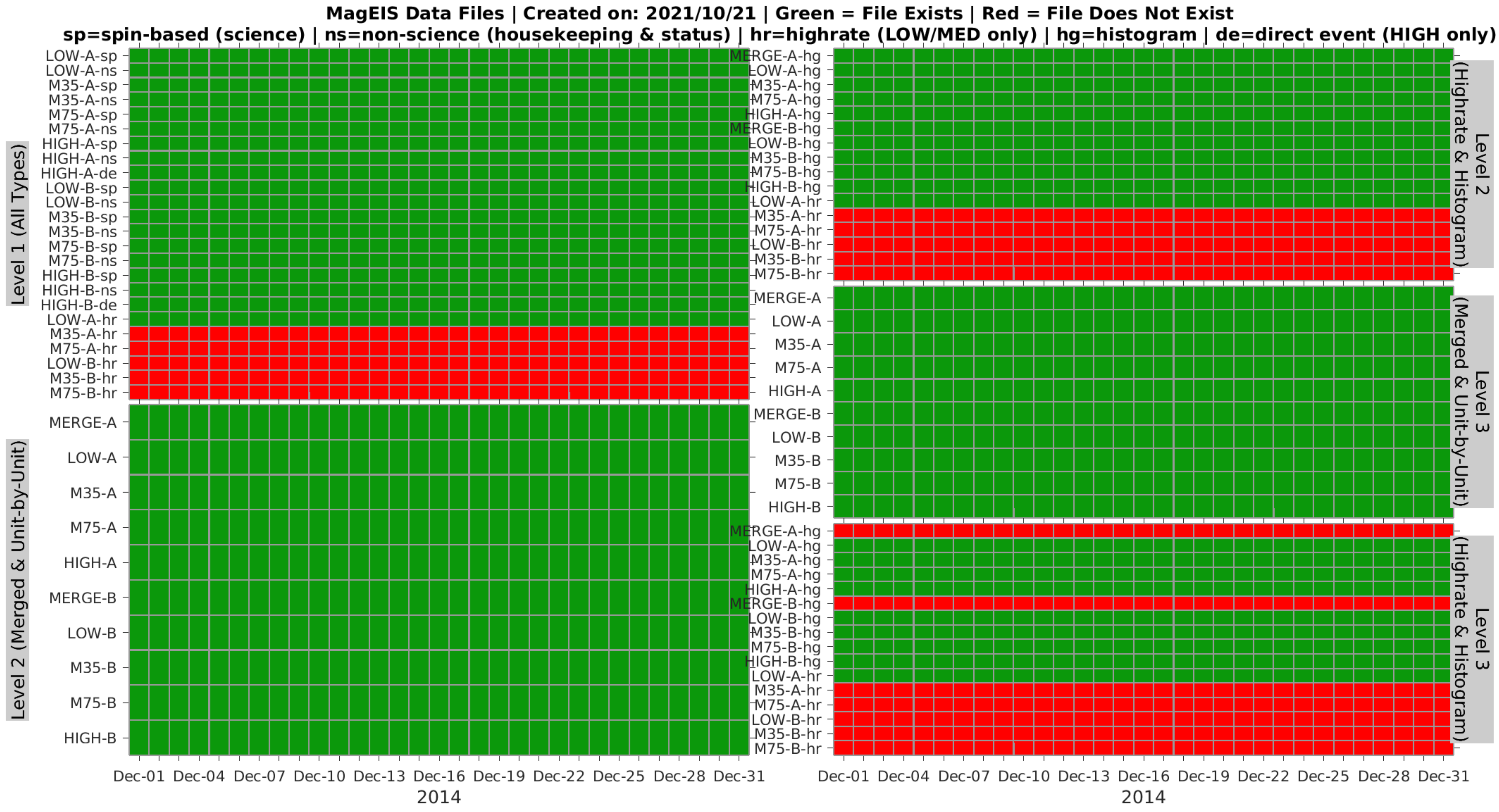
MagEIS Data Files | Created on: 2021/10/21 | Green = File Exists | Red = File Does Not Exist
sp=spin-based (science) | ns=non-science (housekeeping & status) | hr=highrate (LOW/MED only) | hg=histogram | de=direct event (HIGH only)
Level 1 (All Types)
LOW-A-sp
LOW-A-ns
M35-A-sp
M35-A-ns
M75-A-sp
M75-A-ns
HIGH-A-sp
HIGH-A-ns
HIGH-A-de
LOW-B-sp
LOW-B-ns
M35-B-sp
M35-B-ns
M75-B-sp
M75-B-ns
HIGH-B-sp
HIGH-B-ns
HIGH-B-de
LOW-A-hr
M35-A-hr
M75-A-hr
LOW-B-hr
M35-B-hr
M75-B-hr
Level 2 (Merged & Unit-by-Unit)
MERGE-A
LOW-A
M35-A
M75-A
HIGH-A
MERGE-B
LOW-B
M35-B
M75-B
HIGH-B
Level 2 (Highrate & Histogram)
MERGE-A-hg
LOW-A-hg
M35-A-hg
M75-A-hg
HIGH-A-hg
MERGE-B-hg
LOW-B-hg
M35-B-hg
M75-B-hg
HIGH-B-hg
LOW-A-hr
M35-A-hr
M75-A-hr
LOW-B-hr
M35-B-hr
M75-B-hr
Level 3 (Merged & Unit-by-Unit)
MERGE-A
LOW-A
M35-A
M75-A
HIGH-A
MERGE-B
LOW-B
M35-B
M75-B
HIGH-B
Level 3 (Highrate & Histogram)
MERGE-A-hg
LOW-A-hg
M35-A-hg
M75-A-hg
HIGH-A-hg
MERGE-B-hg
LOW-B-hg
M35-B-hg
M75-B-hg
HIGH-B-hg
LOW-A-hr
M35-A-hr
M75-A-hr
LOW-B-hr
M35-B-hr
M75-B-hr
Dec-01 Dec-04 Dec-07 Dec-10 Dec-13 Dec-16 Dec-19 Dec-22 Dec-25 Dec-28 Dec-31
2014
Dec-01 Dec-04 Dec-07 Dec-10 Dec-13 Dec-16 Dec-19 Dec-22 Dec-25 Dec-28 Dec-31
2014

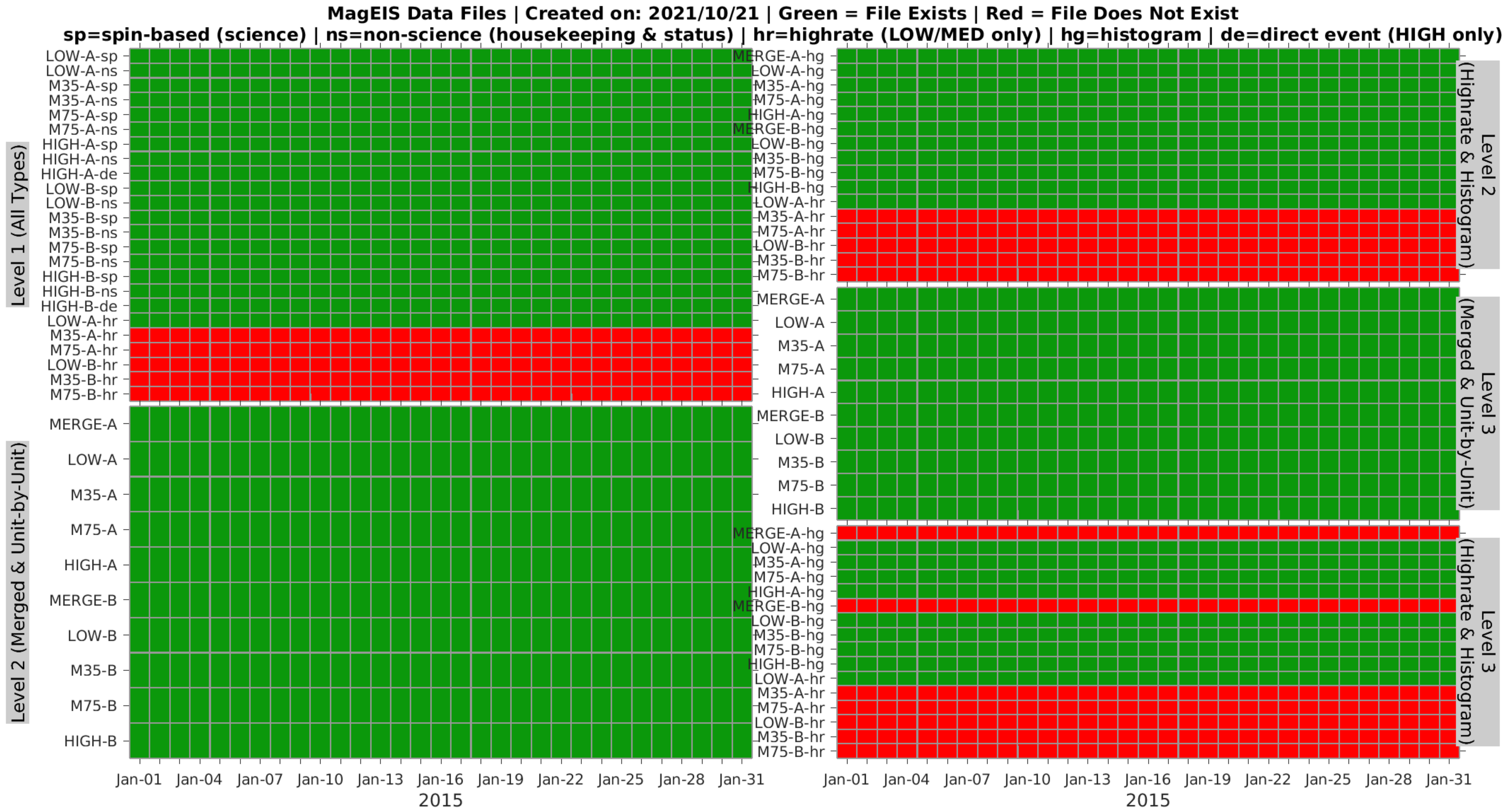
MagEIS Data Files | Created on: 2021/10/21 | Green = File Exists | Red = File Does Not Exist
sp=spin-based (science) | ns=non-science (housekeeping & status) | hr=highrate (LOW/MED only) | hg=histogram | de=direct event (HIGH only)
Level 1 (All Types)
LOW-A-sp
LOW-A-ns
M35-A-sp
M35-A-ns
M75-A-sp
M75-A-ns
HIGH-A-sp
HIGH-A-ns
HIGH-A-de
LOW-B-sp
LOW-B-ns
M35-B-sp
M35-B-ns
M75-B-sp
M75-B-ns
HIGH-B-sp
HIGH-B-ns
HIGH-B-de
LOW-A-hr
M35-A-hr
M75-A-hr
LOW-B-hr
M35-B-hr
M75-B-hr
Level 2 (Merged & Unit-by-Unit)
MERGE-A
LOW-A
M35-A
M75-A
HIGH-A
MERGE-B
LOW-B
M35-B
M75-B
HIGH-B
Level 2 (Highrate & Histogram)
MERGE-A-hg
LOW-A-hg
M35-A-hg
M75-A-hg
HIGH-A-hg
MERGE-B-hg
LOW-B-hg
M35-B-hg
M75-B-hg
HIGH-B-hg
LOW-A-hr
M35-A-hr
M75-A-hr
LOW-B-hr
M35-B-hr
M75-B-hr
Level 3 (Merged & Unit-by-Unit)
MERGE-A
LOW-A
M35-A
M75-A
HIGH-A
MERGE-B
LOW-B
M35-B
M75-B
HIGH-B
Level 3 (Highrate & Histogram)
MERGE-A-hg
LOW-A-hg
M35-A-hg
M75-A-hg
HIGH-A-hg
MERGE-B-hg
LOW-B-hg
M35-B-hg
M75-B-hg
HIGH-B-hg
LOW-A-hr
M35-A-hr
M75-A-hr
LOW-B-hr
M35-B-hr
M75-B-hr
Jan-01
Jan-04
Jan-07
Jan-10
Jan-13
Jan-16
Jan-19
Jan-22
Jan-25
Jan-28
Jan-31
2015

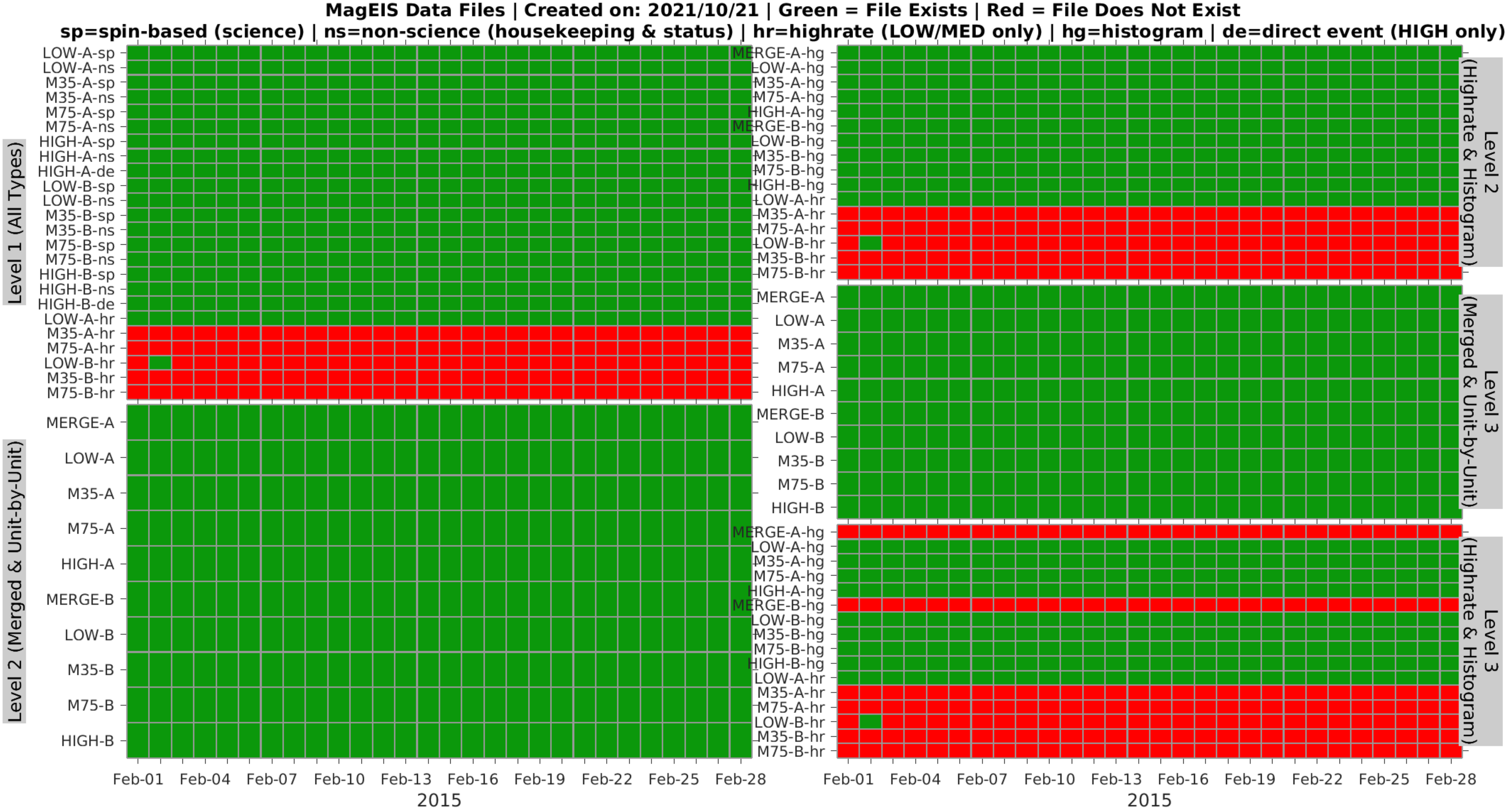
MagEIS Data Files | Created on: 2021/10/21 | Green = File Exists | Red = File Does Not Exist
sp=spin-based (science) | ns=non-science (housekeeping & status) | hr=highrate (LOW/MED only) | hg=histogram | de=direct event (HIGH only)
Level 1 (All Types)
LOW-A-sp
LOW-A-ns
M35-A-sp
M35-A-ns
M75-A-sp
M75-A-ns
HIGH-A-sp
HIGH-A-ns
HIGH-A-de
LOW-B-sp
LOW-B-ns
M35-B-sp
M35-B-ns
M75-B-sp
M75-B-ns
HIGH-B-sp
HIGH-B-ns
HIGH-B-de
LOW-A-hr
M35-A-hr
M75-A-hr
LOW-B-hr
M35-B-hr
M75-B-hr
Level 2 (Merged & Unit-by-Unit)
MERGE-A
LOW-A
M35-A
M75-A
HIGH-A
MERGE-B
LOW-B
M35-B
M75-B
HIGH-B
Level 2 (Highrate & Histogram)
MERGE-A-hg
LOW-A-hg
M35-A-hg
M75-A-hg
HIGH-A-hg
MERGE-B-hg
LOW-B-hg
M35-B-hg
M75-B-hg
HIGH-B-hg
LOW-A-hr
M35-A-hr
M75-A-hr
LOW-B-hr
M35-B-hr
M75-B-hr
Level 3 (Merged & Unit-by-Unit)
MERGE-A
LOW-A
M35-A
M75-A
HIGH-A
MERGE-B
LOW-B
M35-B
M75-B
HIGH-B
Level 3 (Highrate & Histogram)
MERGE-A-hg
LOW-A-hg
M35-A-hg
M75-A-hg
HIGH-A-hg
MERGE-B-hg
LOW-B-hg
M35-B-hg
M75-B-hg
HIGH-B-hg
LOW-A-hr
M35-A-hr
M75-A-hr
LOW-B-hr
M35-B-hr
M75-B-hr
Feb-01
Feb-04
Feb-07
Feb-10
Feb-13
Feb-16
Feb-19
Feb-22
Feb-25
Feb-28
2015

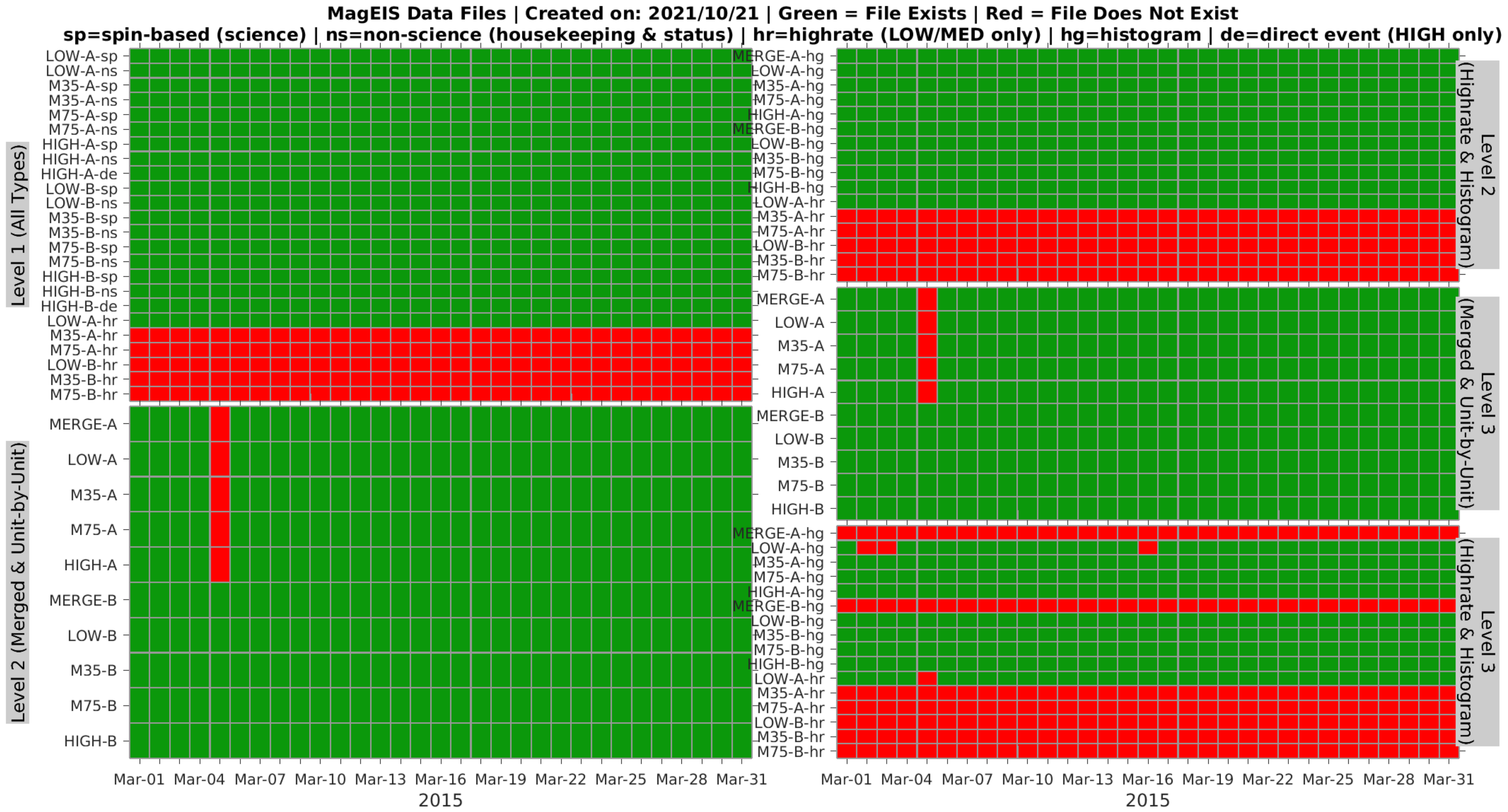
MagEIS Data Files | Created on: 2021/10/21 | Green = File Exists | Red = File Does Not Exist
sp=spin-based (science) | ns=non-science (housekeeping & status) | hr=highrate (LOW/MED only) | hg=histogram | de=direct event (HIGH only)
Level 1 (All Types)
LOW-A-sp
LOW-A-ns
M35-A-sp
M35-A-ns
M75-A-sp
M75-A-ns
HIGH-A-sp
HIGH-A-ns
HIGH-A-de
LOW-B-sp
LOW-B-ns
M35-B-sp
M35-B-ns
M75-B-sp
M75-B-ns
HIGH-B-sp
HIGH-B-ns
HIGH-B-de
LOW-A-hr
M35-A-hr
M75-A-hr
LOW-B-hr
M35-B-hr
M75-B-hr
Level 2 (Merged & Unit-by-Unit)
MERGE-A
LOW-A
M35-A
M75-A
HIGH-A
MERGE-B
LOW-B
M35-B
M75-B
HIGH-B
Level 2 (Highrate & Histogram)
MERGE-A-hg
LOW-A-hg
M35-A-hg
M75-A-hg
HIGH-A-hg
MERGE-B-hg
LOW-B-hg
M35-B-hg
M75-B-hg
HIGH-B-hg
LOW-A-hr
M35-A-hr
M75-A-hr
LOW-B-hr
M35-B-hr
M75-B-hr
Level 3 (Merged & Unit-by-Unit)
MERGE-A
LOW-A
M35-A
M75-A
HIGH-A
MERGE-B
LOW-B
M35-B
M75-B
HIGH-B
Level 3 (Highrate & Histogram)
MERGE-A-hg
LOW-A-hg
M35-A-hg
M75-A-hg
HIGH-A-hg
MERGE-B-hg
LOW-B-hg
M35-B-hg
M75-B-hg
HIGH-B-hg
LOW-A-hr
M35-A-hr
M75-A-hr
LOW-B-hr
M35-B-hr
M75-B-hr
Mar-01 Mar-04 Mar-07 Mar-10 Mar-13 Mar-16 Mar-19 Mar-22 Mar-25 Mar-28 Mar-31
2015
Mar-01 Mar-04 Mar-07 Mar-10 Mar-13 Mar-16 Mar-19 Mar-22 Mar-25 Mar-28 Mar-31
2015

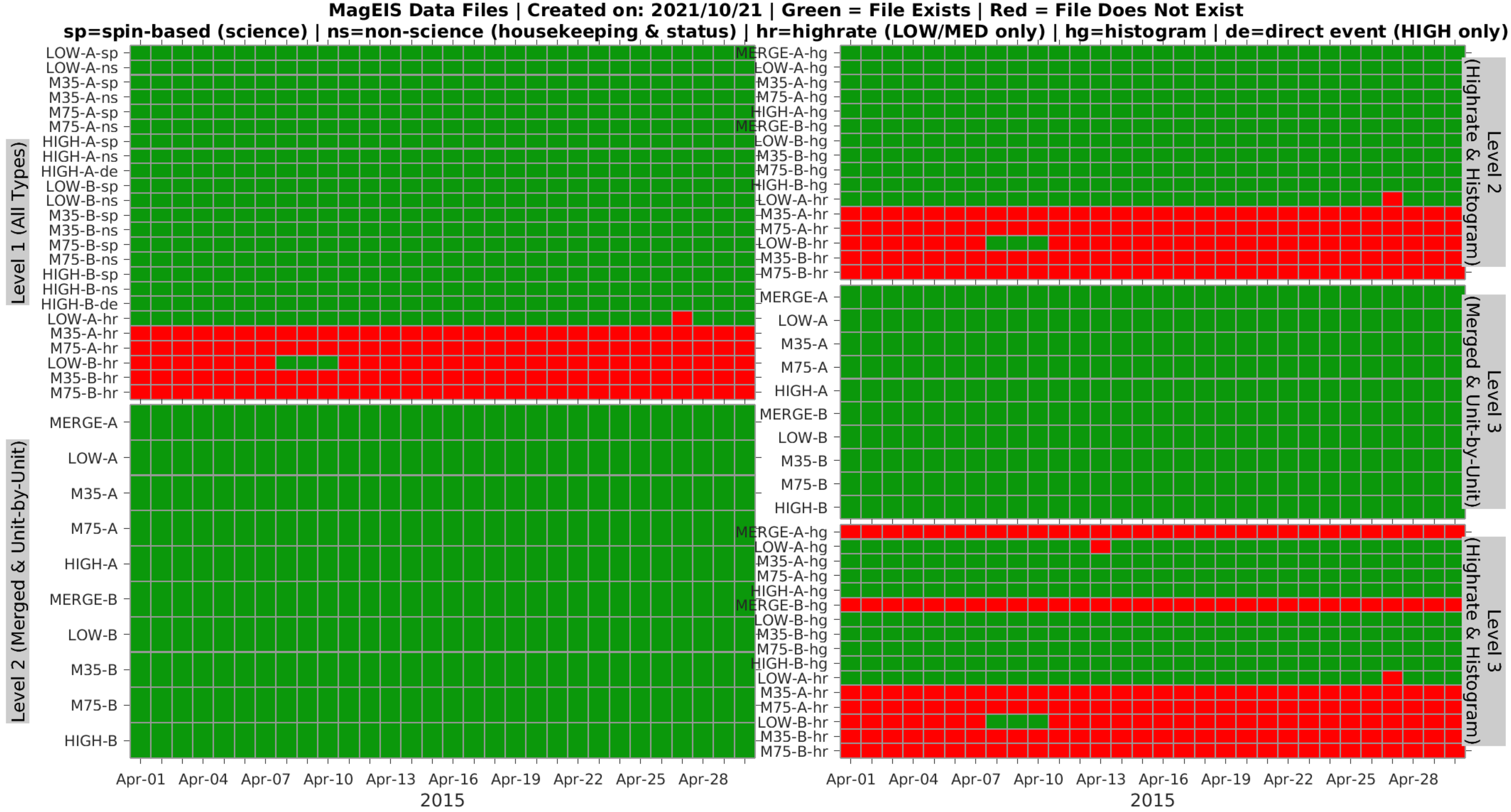

MagEIS Data Files | Created on: 2021/10/21 | Green = File Exists | Red = File Does Not Exist
sp=spin-based (science) | ns=non-science (housekeeping & status) | hr=highrate (LOW/MED only) | hg=histogram | de=direct event (HIGH only)
Level 1 (All Types)
LOW-A-sp
LOW-A-ns
M35-A-sp
M35-A-ns
M75-A-sp
M75-A-ns
HIGH-A-sp
HIGH-A-ns
HIGH-A-de
LOW-B-sp
LOW-B-ns
M35-B-sp
M35-B-ns
M75-B-sp
M75-B-ns
HIGH-B-sp
HIGH-B-ns
HIGH-B-de
LOW-A-hr
M35-A-hr
M75-A-hr
LOW-B-hr
M35-B-hr
M75-B-hr
Level 2 (Merged & Unit-by-Unit)
MERGE-A
LOW-A
M35-A
M75-A
HIGH-A
MERGE-B
LOW-B
M35-B
M75-B
HIGH-B
Level 2 (Highrate & Histogram)
MERGE-A-hg
LOW-A-hg
M35-A-hg
M75-A-hg
HIGH-A-hg
MERGE-B-hg
LOW-B-hg
M35-B-hg
M75-B-hg
HIGH-B-hg
LOW-A-hr
M35-A-hr
M75-A-hr
LOW-B-hr
M35-B-hr
M75-B-hr
Level 3 (Merged & Unit-by-Unit)
MERGE-A
LOW-A
M35-A
M75-A
HIGH-A
MERGE-B
LOW-B
M35-B
M75-B
HIGH-B
Level 3 (Highrate & Histogram)
MERGE-A-hg
LOW-A-hg
M35-A-hg
M75-A-hg
HIGH-A-hg
MERGE-B-hg
LOW-B-hg
M35-B-hg
M75-B-hg
HIGH-B-hg
LOW-A-hr
M35-A-hr
M75-A-hr
LOW-B-hr
M35-B-hr
M75-B-hr
Apr-01
Apr-04
Apr-07
Apr-10
Apr-13
Apr-16
Apr-19
Apr-22
Apr-25
Apr-28
2015

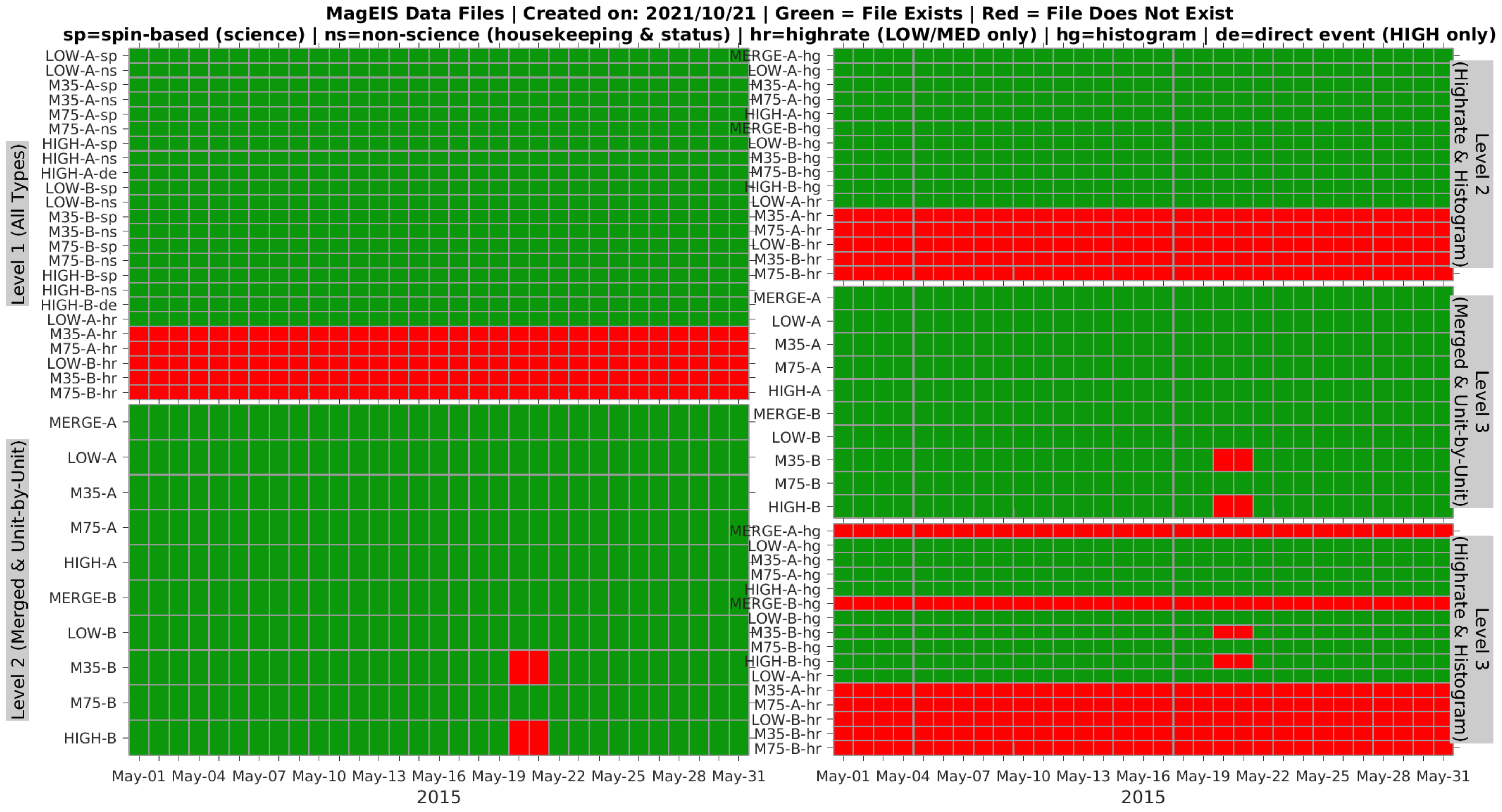

MagEIS Data Files | Created on: 2021/10/21 | Green = File Exists | Red = File Does Not Exist
sp=spin-based (science) | ns=non-science (housekeeping & status) | hr=highrate (LOW/MED only) | hg=histogram | de=direct event (HIGH only)
Level 1 (All Types)
LOW-A-sp
LOW-A-ns
M35-A-sp
M35-A-ns
M75-A-sp
M75-A-ns
HIGH-A-sp
HIGH-A-ns
HIGH-A-de
LOW-B-sp
LOW-B-ns
M35-B-sp
M35-B-ns
M75-B-sp
M75-B-ns
HIGH-B-sp
HIGH-B-ns
HIGH-B-de
LOW-A-hr
M35-A-hr
M75-A-hr
LOW-B-hr
M35-B-hr
M75-B-hr
Level 2 (Merged & Unit-by-Unit)
MERGE-A
LOW-A
M35-A
M75-A
HIGH-A
MERGE-B
LOW-B
M35-B
M75-B
HIGH-B
Level 2 (Highrate & Histogram)
MERGE-A-hg
LOW-A-hg
M35-A-hg
M75-A-hg
HIGH-A-hg
MERGE-B-hg
LOW-B-hg
M35-B-hg
M75-B-hg
HIGH-B-hg
LOW-A-hr
M35-A-hr
M75-A-hr
LOW-B-hr
M35-B-hr
M75-B-hr
Level 3 (Merged & Unit-by-Unit)
MERGE-A
LOW-A
M35-A
M75-A
HIGH-A
MERGE-B
LOW-B
M35-B
M75-B
HIGH-B
Level 3 (Highrate & Histogram)
MERGE-A-hg
LOW-A-hg
M35-A-hg
M75-A-hg
HIGH-A-hg
MERGE-B-hg
LOW-B-hg
M35-B-hg
M75-B-hg
HIGH-B-hg
LOW-A-hr
M35-A-hr
M75-A-hr
LOW-B-hr
M35-B-hr
M75-B-hr
May-01 May-04 May-07 May-10 May-13 May-16 May-19 May-22 May-25 May-28 May-31
2015

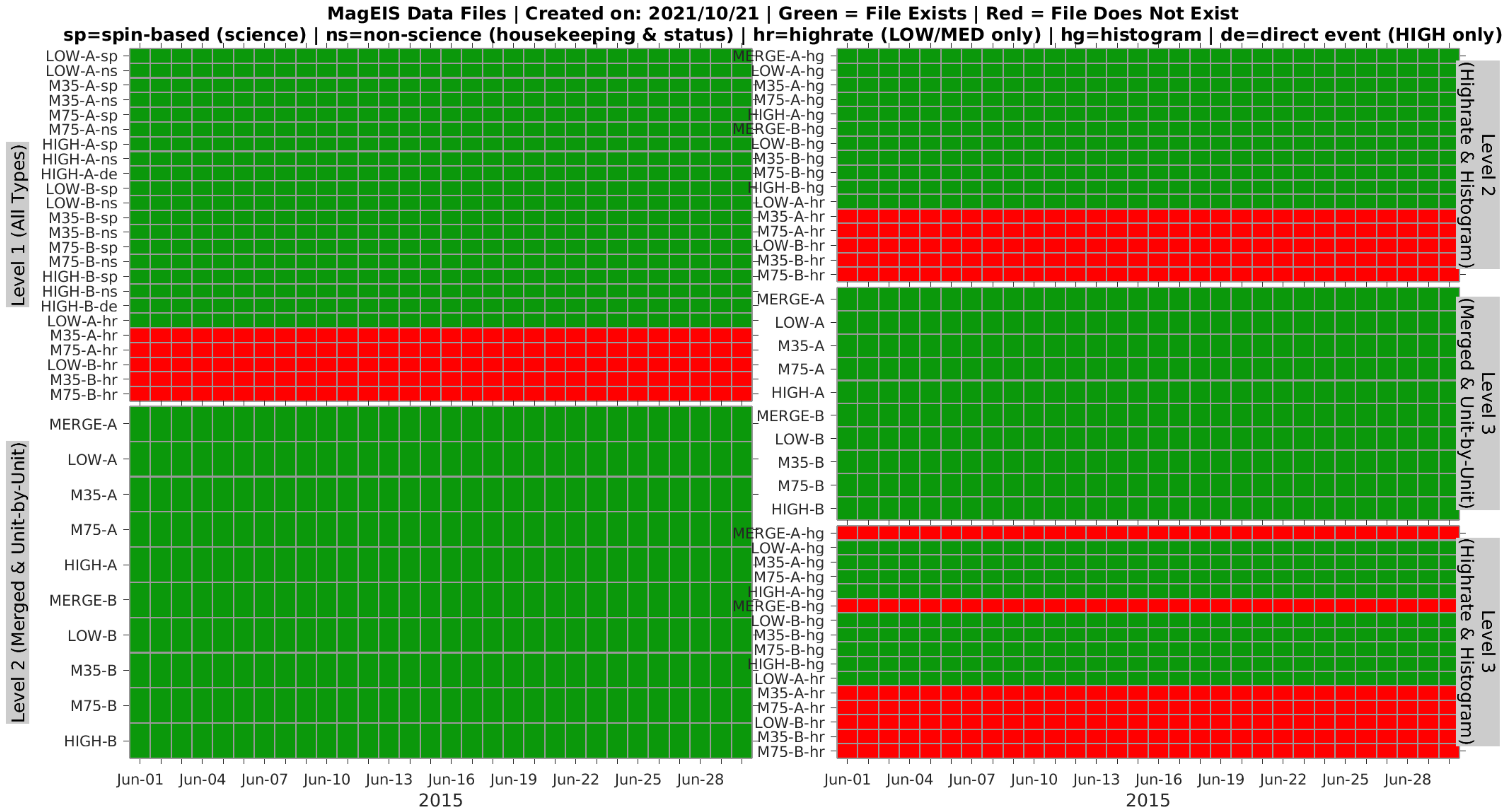
MagEIS Data Files | Created on: 2021/10/21 | Green = File Exists | Red = File Does Not Exist
sp=spin-based (science) | ns=non-science (housekeeping & status) | hr=highrate (LOW/MED only) | hg=histogram | de=direct event (HIGH only)
Level 1 (All Types)
LOW-A-sp
LOW-A-ns
M35-A-sp
M35-A-ns
M75-A-sp
M75-A-ns
HIGH-A-sp
HIGH-A-ns
HIGH-A-de
LOW-B-sp
LOW-B-ns
M35-B-sp
M35-B-ns
M75-B-sp
M75-B-ns
HIGH-B-sp
HIGH-B-ns
HIGH-B-de
LOW-A-hr
M35-A-hr
M75-A-hr
LOW-B-hr
M35-B-hr
M75-B-hr
Level 2 (Merged & Unit-by-Unit)
MERGE-A
LOW-A
M35-A
M75-A
HIGH-A
MERGE-B
LOW-B
M35-B
M75-B
HIGH-B
Jun-01
Jun-04
Jun-07
Jun-10
Jun-13
Jun-16
Jun-19
Jun-22
Jun-25
Jun-28
2015
Level 2 (Highrate & Histogram)
MERGE-A-hg
LOW-A-hg
M35-A-hg
M75-A-hg
HIGH-A-hg
MERGE-B-hg
LOW-B-hg
M35-B-hg
M75-B-hg
HIGH-B-hg
LOW-A-hr
M35-A-hr
M75-A-hr
LOW-B-hr
M35-B-hr
M75-B-hr
Level 3 (Merged & Unit-by-Unit)
MERGE-A
LOW-A
M35-A
M75-A
HIGH-A
MERGE-B
LOW-B
M35-B
M75-B
HIGH-B
Level 3 (Highrate & Histogram)
MERGE-A-hg
LOW-A-hg
M35-A-hg
M75-A-hg
HIGH-A-hg
MERGE-B-hg
LOW-B-hg
M35-B-hg
M75-B-hg
HIGH-B-hg
LOW-A-hr
M35-A-hr
M75-A-hr
LOW-B-hr
M35-B-hr
M75-B-hr
Jun-01
Jun-04
Jun-07
Jun-10
Jun-13
Jun-16
Jun-19
Jun-22
Jun-25
Jun-28
2015

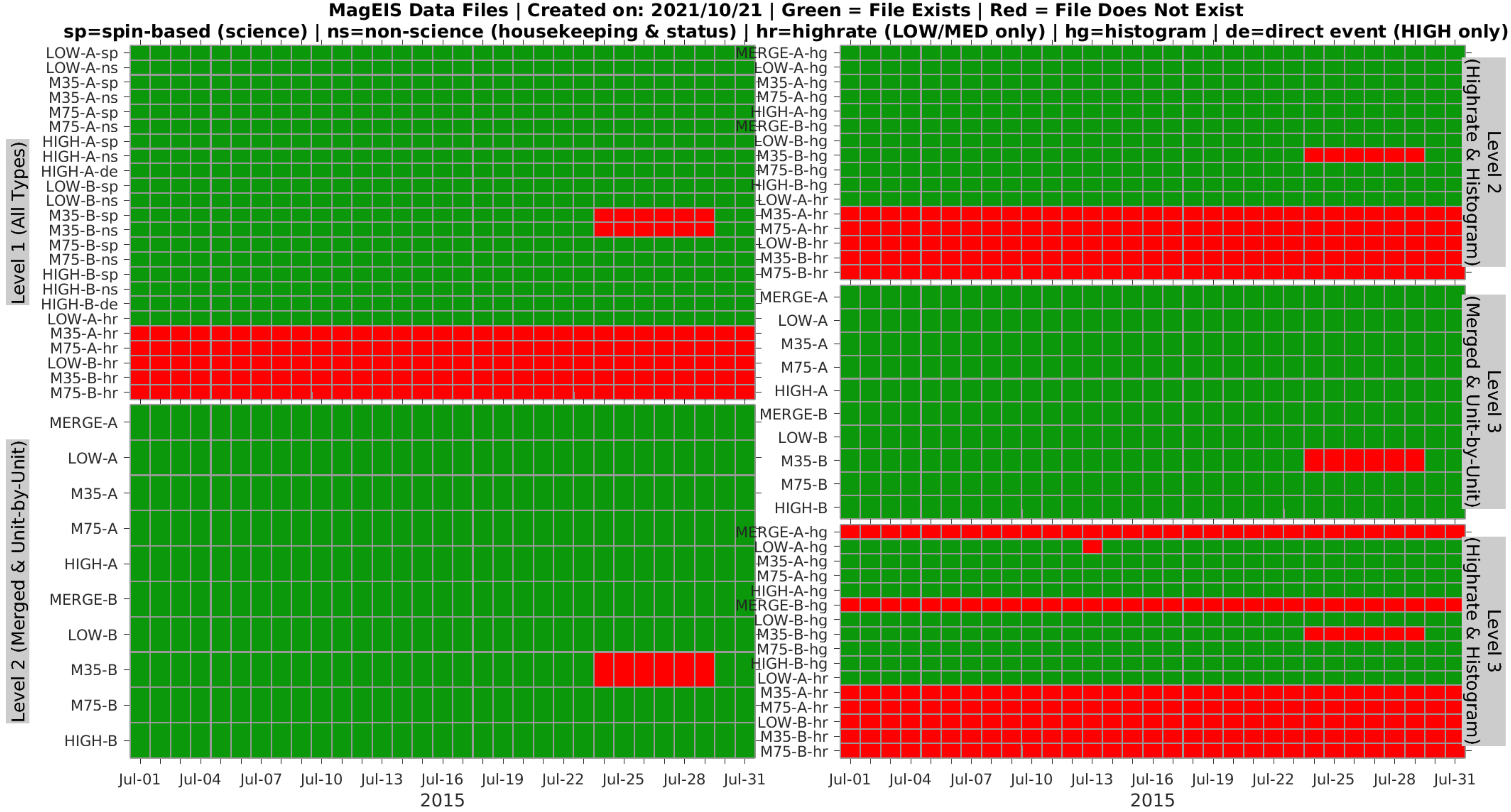

MagEIS Data Files | Created on: 2021/10/21 | Green = File Exists | Red = File Does Not Exist
sp=spin-based (science) | ns=non-science (housekeeping & status) | hr=highrate (LOW/MED only) | hg=histogram | de=direct event (HIGH only)
Level 1 (All Types)
LOW-A-sp
LOW-A-ns
M35-A-sp
M35-A-ns
M75-A-sp
M75-A-ns
HIGH-A-sp
HIGH-A-ns
HIGH-A-de
LOW-B-sp
LOW-B-ns
M35-B-sp
M35-B-ns
M75-B-sp
M75-B-ns
HIGH-B-sp
HIGH-B-ns
HIGH-B-de
LOW-A-hr
M35-A-hr
M75-A-hr
LOW-B-hr
M35-B-hr
M75-B-hr
Level 2 (Merged & Unit-by-Unit)
MERGE-A
LOW-A
M35-A
M75-A
HIGH-A
MERGE-B
LOW-B
M35-B
M75-B
HIGH-B
Level 2 (Highrate & Histogram)
MERGE-A-hg
LOW-A-hg
M35-A-hg
M75-A-hg
HIGH-A-hg
MERGE-B-hg
LOW-B-hg
M35-B-hg
M75-B-hg
HIGH-B-hg
LOW-A-hr
M35-A-hr
M75-A-hr
LOW-B-hr
M35-B-hr
M75-B-hr
Level 3 (Merged & Unit-by-Unit)
MERGE-A
LOW-A
M35-A
M75-A
HIGH-A
MERGE-B
LOW-B
M35-B
M75-B
HIGH-B
Level 3 (Highrate & Histogram)
MERGE-A-hg
LOW-A-hg
M35-A-hg
M75-A-hg
HIGH-A-hg
MERGE-B-hg
LOW-B-hg
M35-B-hg
M75-B-hg
HIGH-B-hg
LOW-A-hr
M35-A-hr
M75-A-hr
LOW-B-hr
M35-B-hr
M75-B-hr
Jul-01
Jul-04
Jul-07
Jul-10
Jul-13
Jul-16
Jul-19
Jul-22
Jul-25
Jul-28
Jul-31
2015

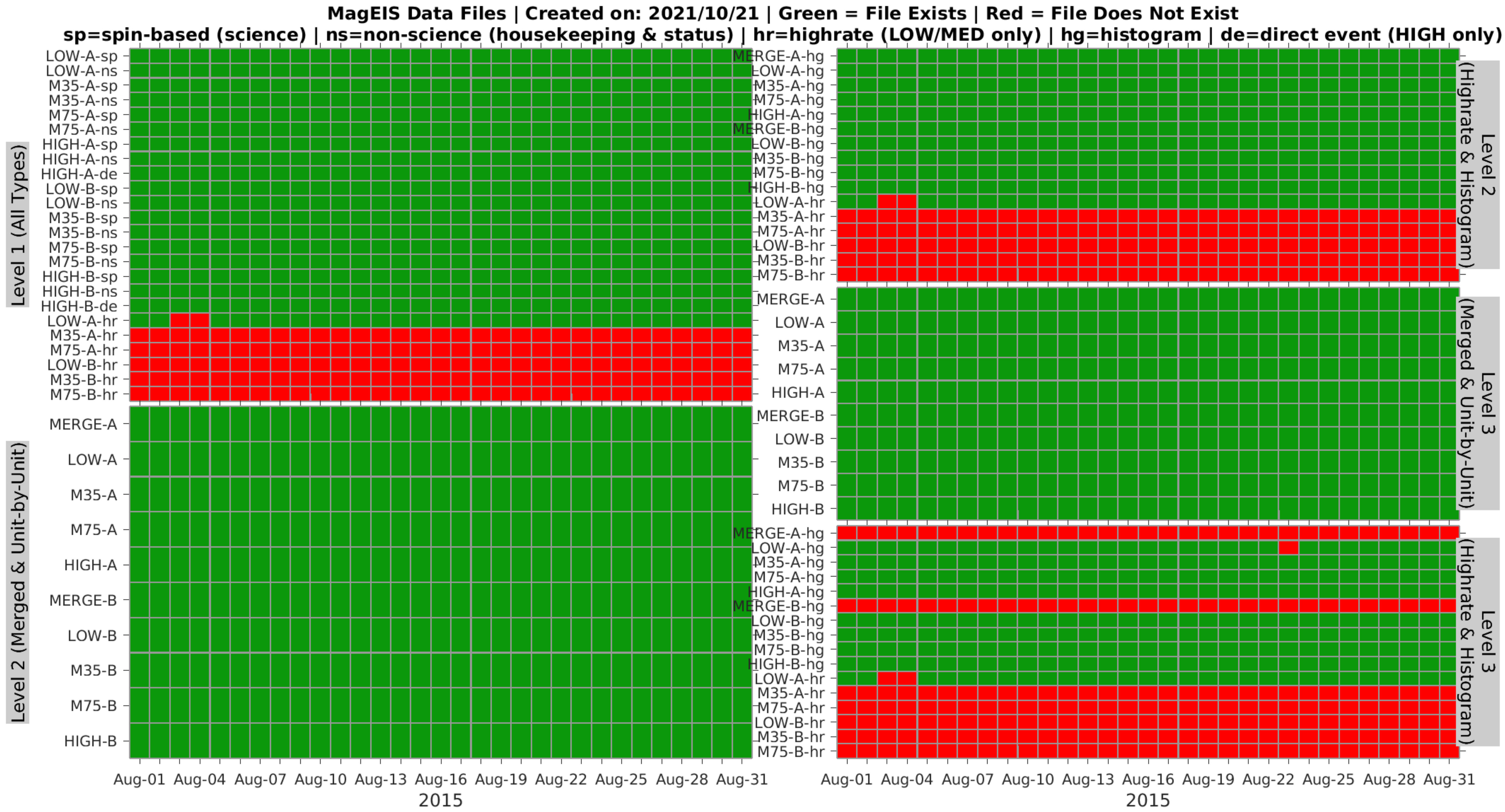
MagEIS Data Files | Created on: 2021/10/21 | Green = File Exists | Red = File Does Not Exist
sp=spin-based (science) | ns=non-science (housekeeping & status) | hr=highrate (LOW/MED only) | hg=histogram | de=direct event (HIGH only)
Level 1 (All Types)
LOW-A-sp
LOW-A-ns
M35-A-sp
M35-A-ns
M75-A-sp
M75-A-ns
HIGH-A-sp
HIGH-A-ns
HIGH-A-de
LOW-B-sp
LOW-B-ns
M35-B-sp
M35-B-ns
M75-B-sp
M75-B-ns
HIGH-B-sp
HIGH-B-ns
HIGH-B-de
LOW-A-hr
M35-A-hr
M75-A-hr
LOW-B-hr
M35-B-hr
M75-B-hr
Level 2 (Merged & Unit-by-Unit)
MERGE-A
LOW-A
M35-A
M75-A
HIGH-A
MERGE-B
LOW-B
M35-B
M75-B
HIGH-B
Level 2 (Highrate & Histogram)
MERGE-A-hg
LOW-A-hg
M35-A-hg
M75-A-hg
HIGH-A-hg
MERGE-B-hg
LOW-B-hg
M35-B-hg
M75-B-hg
HIGH-B-hg
LOW-A-hr
M35-A-hr
M75-A-hr
LOW-B-hr
M35-B-hr
M75-B-hr
Level 3 (Merged & Unit-by-Unit)
MERGE-A
LOW-A
M35-A
M75-A
HIGH-A
MERGE-B
LOW-B
M35-B
M75-B
HIGH-B
Level 3 (Highrate & Histogram)
MERGE-A-hg
LOW-A-hg
M35-A-hg
M75-A-hg
HIGH-A-hg
MERGE-B-hg
LOW-B-hg
M35-B-hg
M75-B-hg
HIGH-B-hg
LOW-A-hr
M35-A-hr
M75-A-hr
LOW-B-hr
M35-B-hr
M75-B-hr
Aug-01 Aug-04 Aug-07 Aug-10 Aug-13 Aug-16 Aug-19 Aug-22 Aug-25 Aug-28 Aug-31
2015
Aug-01 Aug-04 Aug-07 Aug-10 Aug-13 Aug-16 Aug-19 Aug-22 Aug-25 Aug-28 Aug-31
2015

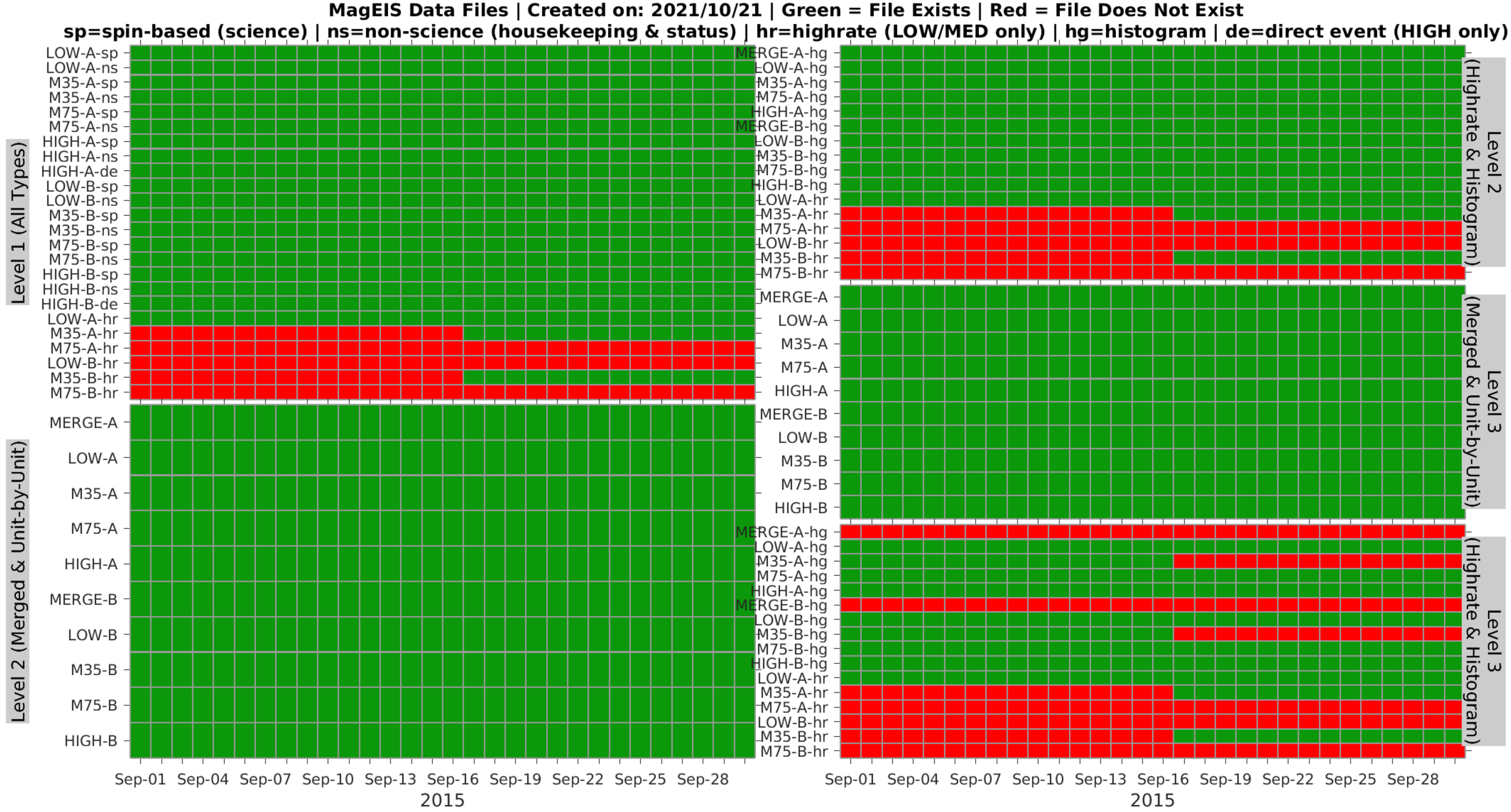
MagEIS Data Files | Created on: 2021/10/21 | Green = File Exists | Red = File Does Not Exist
sp=spin-based (science) | ns=non-science (housekeeping & status) | hr=highrate (LOW/MED only) | hg=histogram | de=direct event (HIGH only)
Level 1 (All Types)
LOW-A-sp
LOW-A-ns
M35-A-sp
M35-A-ns
M75-A-sp
M75-A-ns
HIGH-A-sp
HIGH-A-ns
HIGH-A-de
LOW-B-sp
LOW-B-ns
M35-B-sp
M35-B-ns
M75-B-sp
M75-B-ns
HIGH-B-sp
HIGH-B-ns
HIGH-B-de
LOW-A-hr
M35-A-hr
M75-A-hr
LOW-B-hr
M35-B-hr
M75-B-hr
Level 2 (Merged & Unit-by-Unit)
MERGE-A
LOW-A
M35-A
M75-A
HIGH-A
MERGE-B
LOW-B
M35-B
M75-B
HIGH-B
Level 2 (Highrate & Histogram)
MERGE-A-hg
LOW-A-hg
M35-A-hg
M75-A-hg
HIGH-A-hg
MERGE-B-hg
LOW-B-hg
M35-B-hg
M75-B-hg
HIGH-B-hg
LOW-A-hr
M35-A-hr
M75-A-hr
LOW-B-hr
M35-B-hr
M75-B-hr
Level 3 (Merged & Unit-by-Unit)
MERGE-A
LOW-A
M35-A
M75-A
HIGH-A
MERGE-B
LOW-B
M35-B
M75-B
HIGH-B
Level 3 (Highrate & Histogram)
MERGE-A-hg
LOW-A-hg
M35-A-hg
M75-A-hg
HIGH-A-hg
MERGE-B-hg
LOW-B-hg
M35-B-hg
M75-B-hg
HIGH-B-hg
LOW-A-hr
M35-A-hr
M75-A-hr
LOW-B-hr
M35-B-hr
M75-B-hr
Sep-01 Sep-04 Sep-07 Sep-10 Sep-13 Sep-16 Sep-19 Sep-22 Sep-25 Sep-28
2015
Sep-01 Sep-04 Sep-07 Sep-10 Sep-13 Sep-16 Sep-19 Sep-22 Sep-25 Sep-28
2015

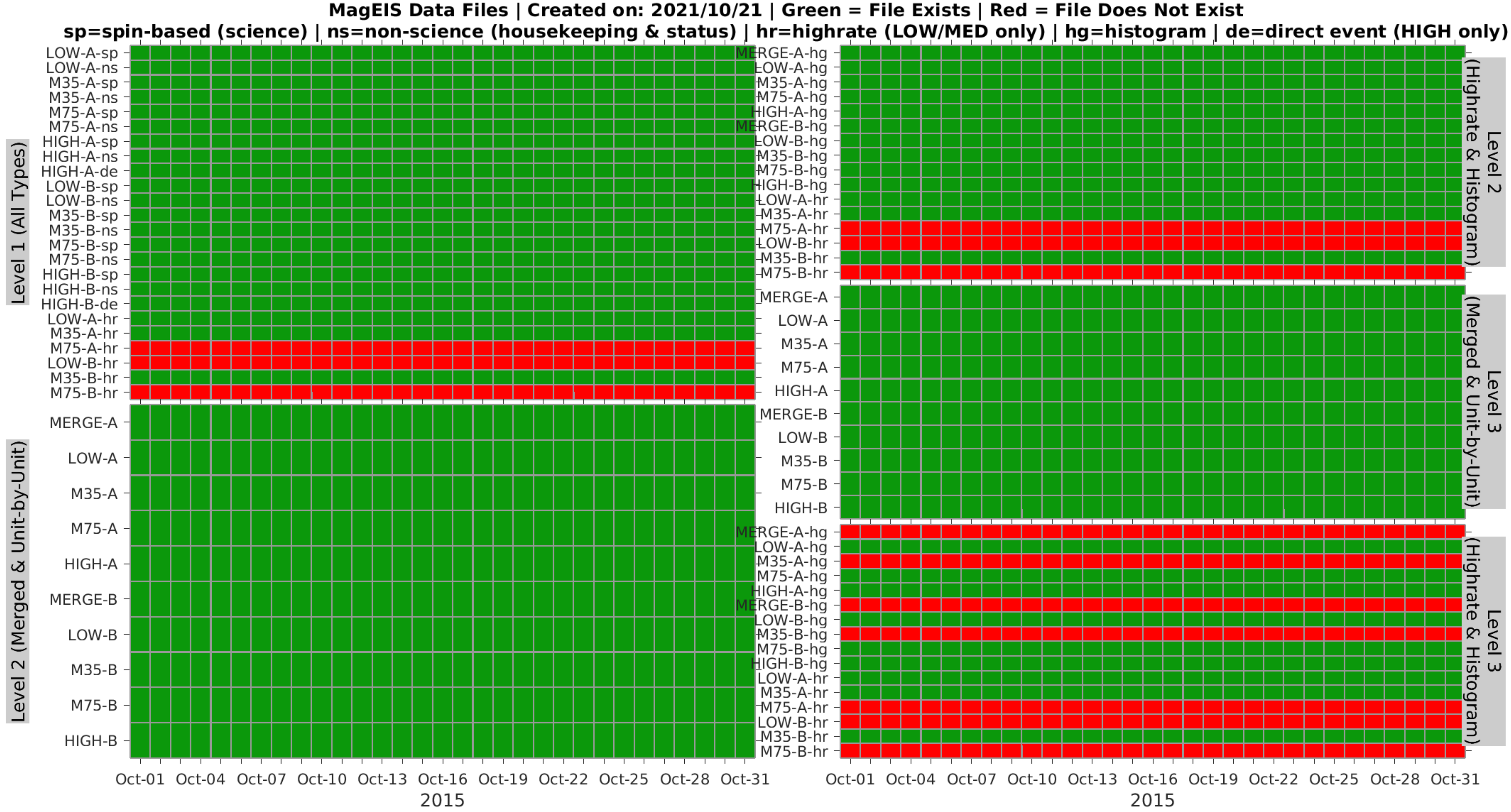
MagEIS Data Files | Created on: 2021/10/21 | Green = File Exists | Red = File Does Not Exist
sp=spin-based (science) | ns=non-science (housekeeping & status) | hr=highrate (LOW/MED only) | hg=histogram | de=direct event (HIGH only)
Level 1 (All Types)
LOW-A-sp
LOW-A-ns
M35-A-sp
M35-A-ns
M75-A-sp
M75-A-ns
HIGH-A-sp
HIGH-A-ns
HIGH-A-de
LOW-B-sp
LOW-B-ns
M35-B-sp
M35-B-ns
M75-B-sp
M75-B-ns
HIGH-B-sp
HIGH-B-ns
HIGH-B-de
LOW-A-hr
M35-A-hr
M75-A-hr
LOW-B-hr
M35-B-hr
M75-B-hr
Level 2 (Merged & Unit-by-Unit)
MERGE-A
LOW-A
M35-A
M75-A
HIGH-A
MERGE-B
LOW-B
M35-B
M75-B
HIGH-B
Level 2 (Highrate & Histogram)
MERGE-A-hg
LOW-A-hg
M35-A-hg
M75-A-hg
HIGH-A-hg
MERGE-B-hg
LOW-B-hg
M35-B-hg
M75-B-hg
HIGH-B-hg
LOW-A-hr
M35-A-hr
M75-A-hr
LOW-B-hr
M35-B-hr
M75-B-hr
Level 3 (Merged & Unit-by-Unit)
Level 3 (Highrate & Histogram)
Oct-01
Oct-04
Oct-07
Oct-10
Oct-13
Oct-16
Oct-19
Oct-22
Oct-25
Oct-28
Oct-31
2015

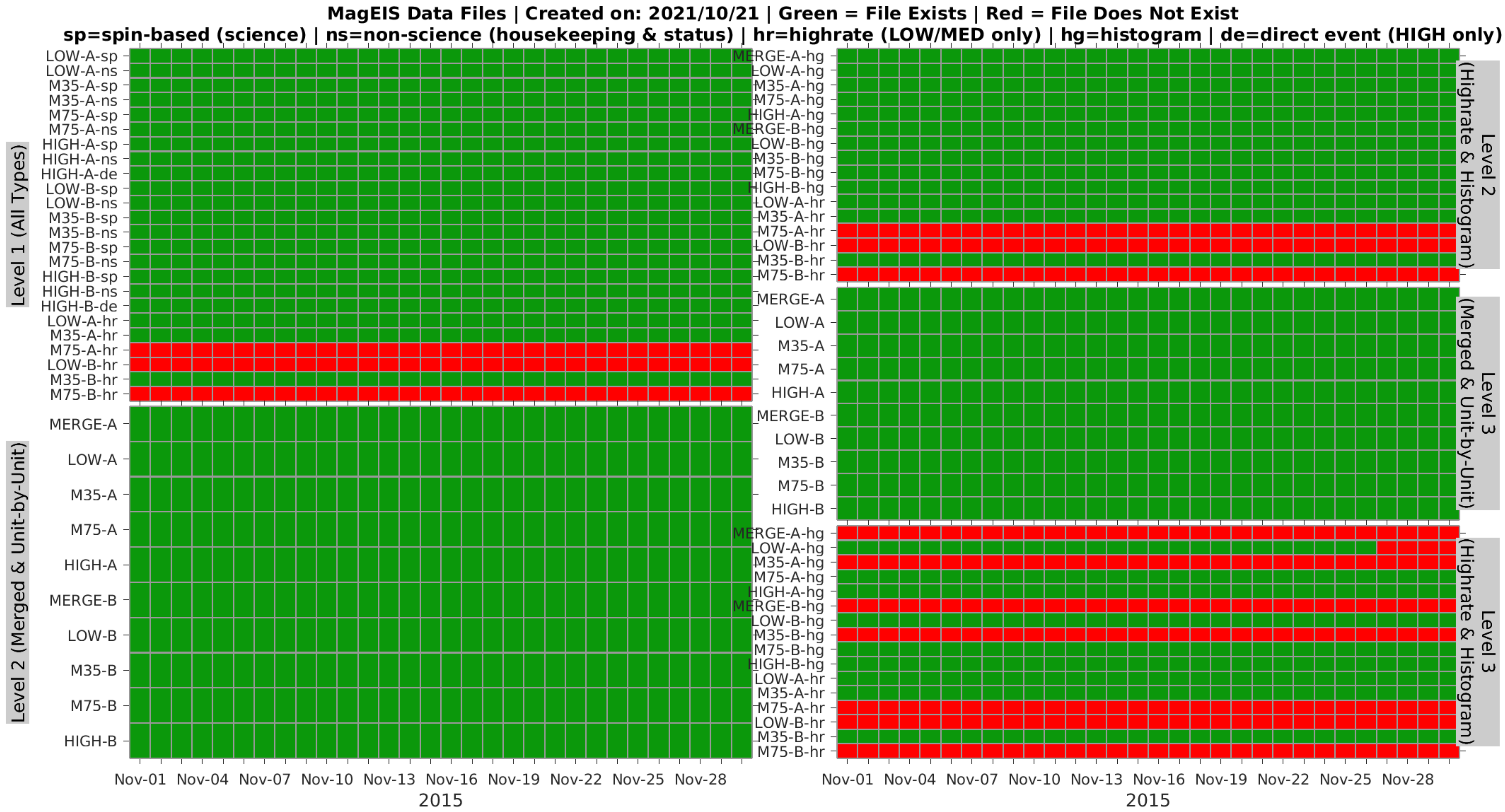
MagEIS Data Files | Created on: 2021/10/21 | Green = File Exists | Red = File Does Not Exist
sp=spin-based (science) | ns=non-science (housekeeping & status) | hr=highrate (LOW/MED only) | hg=histogram | de=direct event (HIGH only)
Level 1 (All Types)
LOW-A-sp
LOW-A-ns
M35-A-sp
M35-A-ns
M75-A-sp
M75-A-ns
HIGH-A-sp
HIGH-A-ns
HIGH-A-de
LOW-B-sp
LOW-B-ns
M35-B-sp
M35-B-ns
M75-B-sp
M75-B-ns
HIGH-B-sp
HIGH-B-ns
HIGH-B-de
LOW-A-hr
M35-A-hr
M75-A-hr
LOW-B-hr
M35-B-hr
M75-B-hr
Level 2 (Merged & Unit-by-Unit)
MERGE-A
LOW-A
M35-A
M75-A
HIGH-A
MERGE-B
LOW-B
M35-B
M75-B
HIGH-B
Level 2 (Highrate & Histogram)
MERGE-A-hg
LOW-A-hg
M35-A-hg
M75-A-hg
HIGH-A-hg
MERGE-B-hg
LOW-B-hg
M35-B-hg
M75-B-hg
HIGH-B-hg
LOW-A-hr
M35-A-hr
M75-A-hr
LOW-B-hr
M35-B-hr
M75-B-hr
Level 3 (Merged & Unit-by-Unit)
MERGE-A
LOW-A
M35-A
M75-A
HIGH-A
MERGE-B
LOW-B
M35-B
M75-B
HIGH-B
Level 3 (Highrate & Histogram)
MERGE-A-hg
LOW-A-hg
M35-A-hg
M75-A-hg
HIGH-A-hg
MERGE-B-hg
LOW-B-hg
M35-B-hg
M75-B-hg
HIGH-B-hg
LOW-A-hr
M35-A-hr
M75-A-hr
LOW-B-hr
M35-B-hr
M75-B-hr
Nov-01 Nov-04 Nov-07 Nov-10 Nov-13 Nov-16 Nov-19 Nov-22 Nov-25 Nov-28
2015

MagEIS Data Files | Created on: 2021/10/21 | Green = File Exists | Red = File Does Not Exist
sp=spin-based (science) | ns=non-science (housekeeping & status) | hr=highrate (LOW/MED only) | hg=histogram | de=direct event (HIGH only)

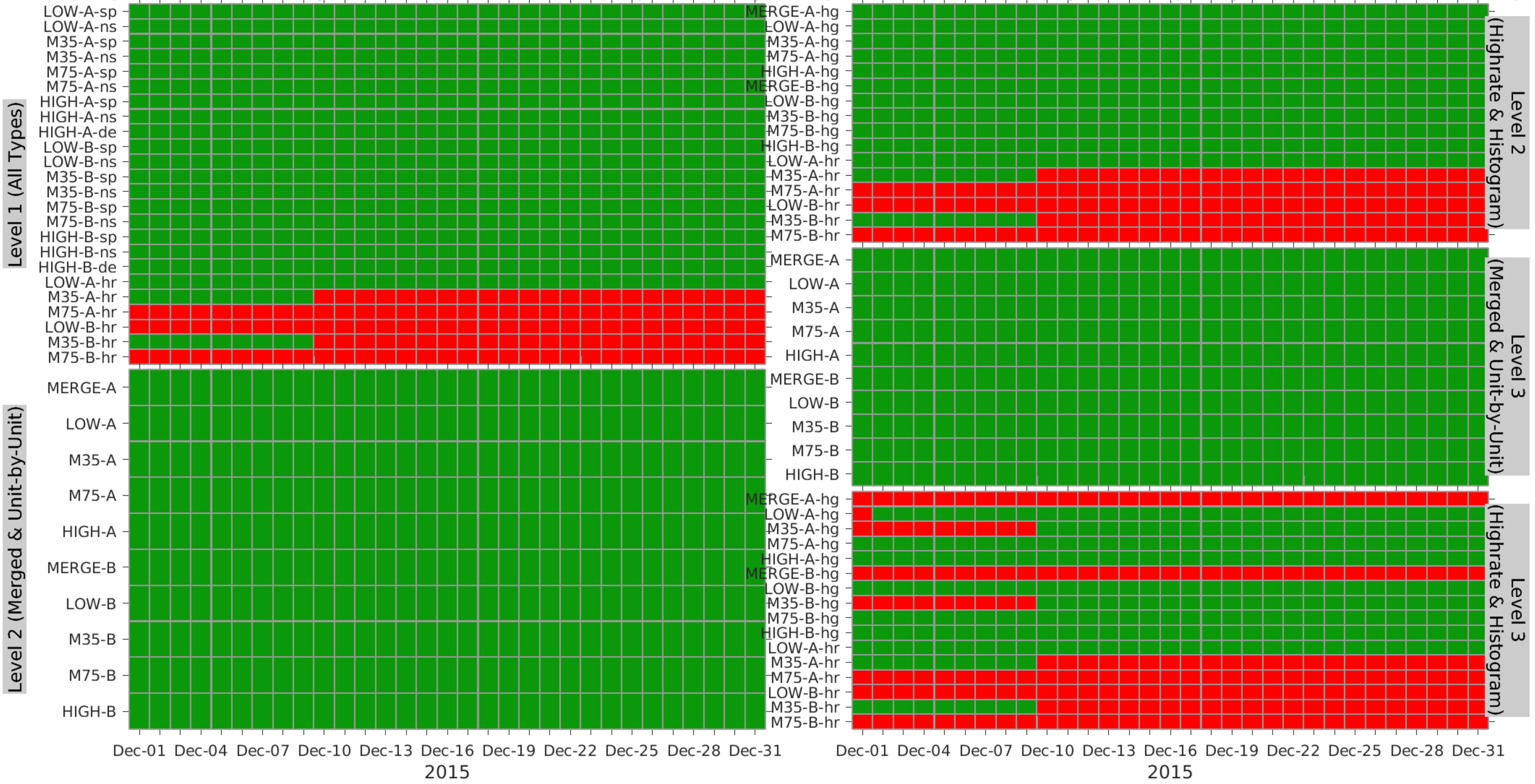

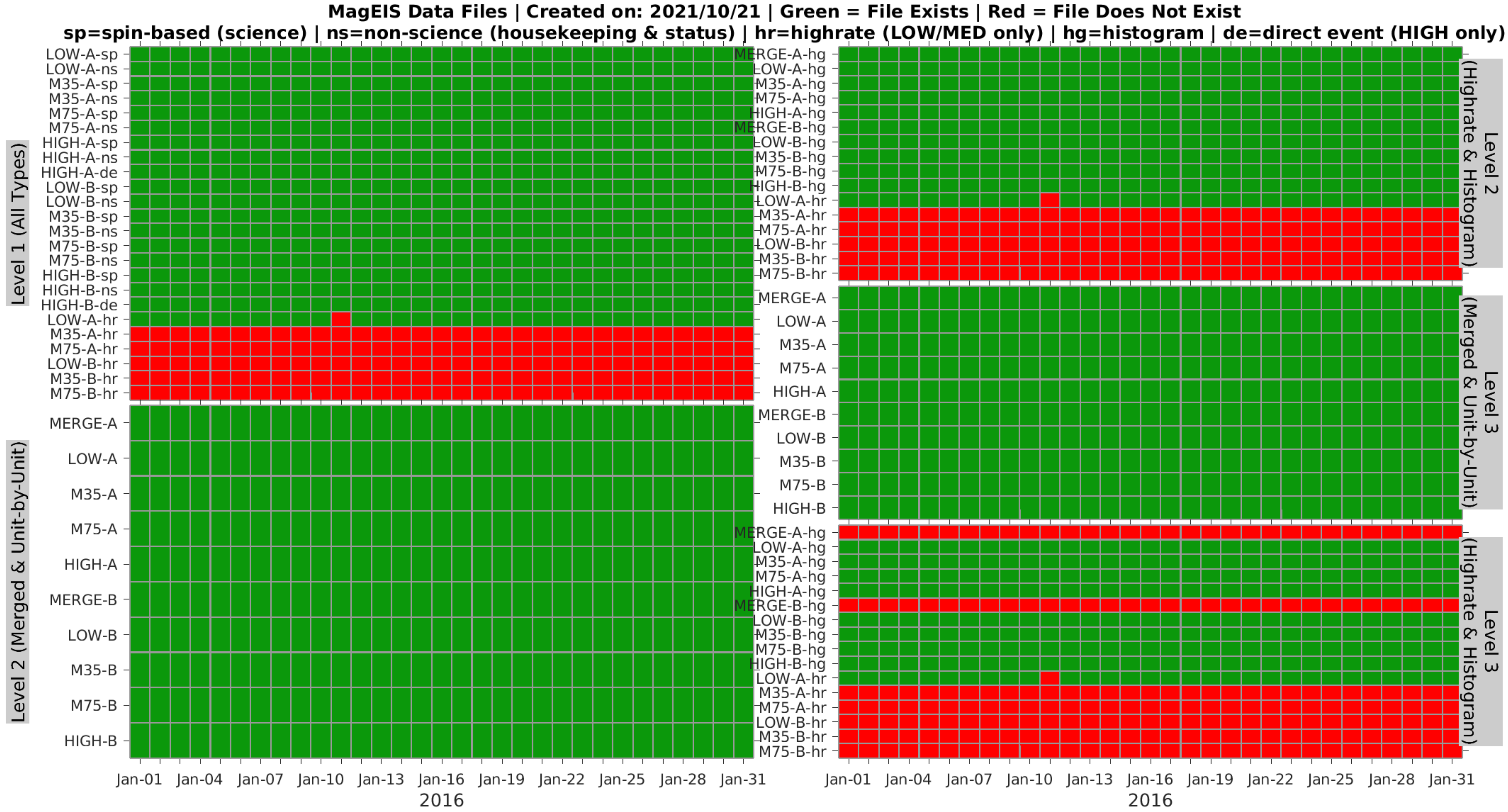

MagEIS Data Files | Created on: 2021/10/21 | Green = File Exists | Red = File Does Not Exist
sp=spin-based (science) | ns=non-science (housekeeping & status) | hr=highrate (LOW/MED only) | hg=histogram | de=direct event (HIGH only)
Level 1 (All Types)
LOW-A-sp
LOW-A-ns
M35-A-sp
M35-A-ns
M75-A-sp
M75-A-ns
HIGH-A-sp
HIGH-A-ns
HIGH-A-de
LOW-B-sp
LOW-B-ns
M35-B-sp
M35-B-ns
M75-B-sp
M75-B-ns
HIGH-B-sp
HIGH-B-ns
HIGH-B-de
LOW-A-hr
M35-A-hr
M75-A-hr
LOW-B-hr
M35-B-hr
M75-B-hr
Level 2 (Merged & Unit-by-Unit)
MERGE-A
LOW-A
M35-A
M75-A
HIGH-A
MERGE-B
LOW-B
M35-B
M75-B
HIGH-B
Level 2 (Highrate & Histogram)
MERGE-A-hg
LOW-A-hg
M35-A-hg
M75-A-hg
HIGH-A-hg
MERGE-B-hg
LOW-B-hg
M35-B-hg
M75-B-hg
HIGH-B-hg
LOW-A-hr
M35-A-hr
M75-A-hr
LOW-B-hr
M35-B-hr
M75-B-hr
Level 3 (Merged & Unit-by-Unit)
MERGE-A
LOW-A
M35-A
M75-A
HIGH-A
MERGE-B
LOW-B
M35-B
M75-B
HIGH-B
Level 3 (Highrate & Histogram)
MERGE-A-hg
LOW-A-hg
M35-A-hg
M75-A-hg
HIGH-A-hg
MERGE-B-hg
LOW-B-hg
M35-B-hg
M75-B-hg
HIGH-B-hg
LOW-A-hr
M35-A-hr
M75-A-hr
LOW-B-hr
M35-B-hr
M75-B-hr
Jan-01
Jan-04
Jan-07
Jan-10
Jan-13
Jan-16
Jan-19
Jan-22
Jan-25
Jan-28
Jan-31
2016

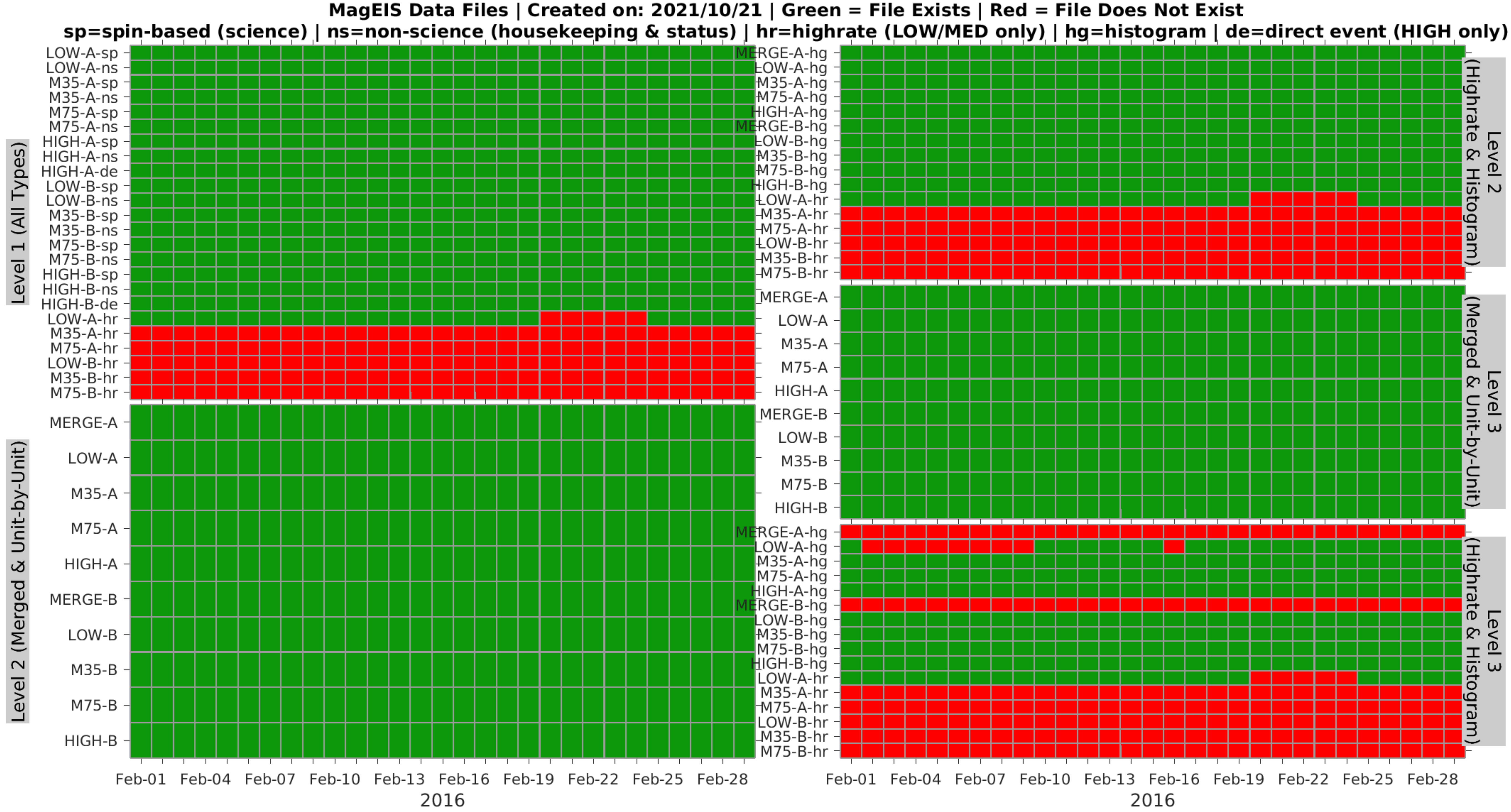
MagEIS Data Files | Created on: 2021/10/21 | Green = File Exists | Red = File Does Not Exist
sp=spin-based (science) | ns=non-science (housekeeping & status) | hr=highrate (LOW/MED only) | hg=histogram | de=direct event (HIGH only)
Level 1 (All Types)
LOW-A-sp
LOW-A-ns
M35-A-sp
M35-A-ns
M75-A-sp
M75-A-ns
HIGH-A-sp
HIGH-A-ns
HIGH-A-de
LOW-B-sp
LOW-B-ns
M35-B-sp
M35-B-ns
M75-B-sp
M75-B-ns
HIGH-B-sp
HIGH-B-ns
HIGH-B-de
LOW-A-hr
M35-A-hr
M75-A-hr
LOW-B-hr
M35-B-hr
M75-B-hr
Level 2 (Merged & Unit-by-Unit)
MERGE-A
LOW-A
M35-A
M75-A
HIGH-A
MERGE-B
LOW-B
M35-B
M75-B
HIGH-B
Level 2 (Highrate & Histogram)
MERGE-A-hg
LOW-A-hg
M35-A-hg
M75-A-hg
HIGH-A-hg
MERGE-B-hg
LOW-B-hg
M35-B-hg
M75-B-hg
HIGH-B-hg
LOW-A-hr
M35-A-hr
M75-A-hr
LOW-B-hr
M35-B-hr
M75-B-hr
Level 3 (Merged & Unit-by-Unit)
MERGE-A
LOW-A
M35-A
M75-A
HIGH-A
MERGE-B
LOW-B
M35-B
M75-B
HIGH-B
Level 3 (Highrate & Histogram)
MERGE-A-hg
LOW-A-hg
M35-A-hg
M75-A-hg
HIGH-A-hg
MERGE-B-hg
LOW-B-hg
M35-B-hg
M75-B-hg
HIGH-B-hg
LOW-A-hr
M35-A-hr
M75-A-hr
LOW-B-hr
M35-B-hr
M75-B-hr
Feb-01
Feb-04
Feb-07
Feb-10
Feb-13
Feb-16
Feb-19
Feb-22
Feb-25
Feb-28
2016

MagEIS Data Files | Created on: 2021/10/21 | Green = File Exists | Red = File Does Not Exist
sp=spin-based (science) | ns=non-science (housekeeping & status) | hr=highrate (LOW/MED only) | hg=histogram | de=direct event (HIGH only)

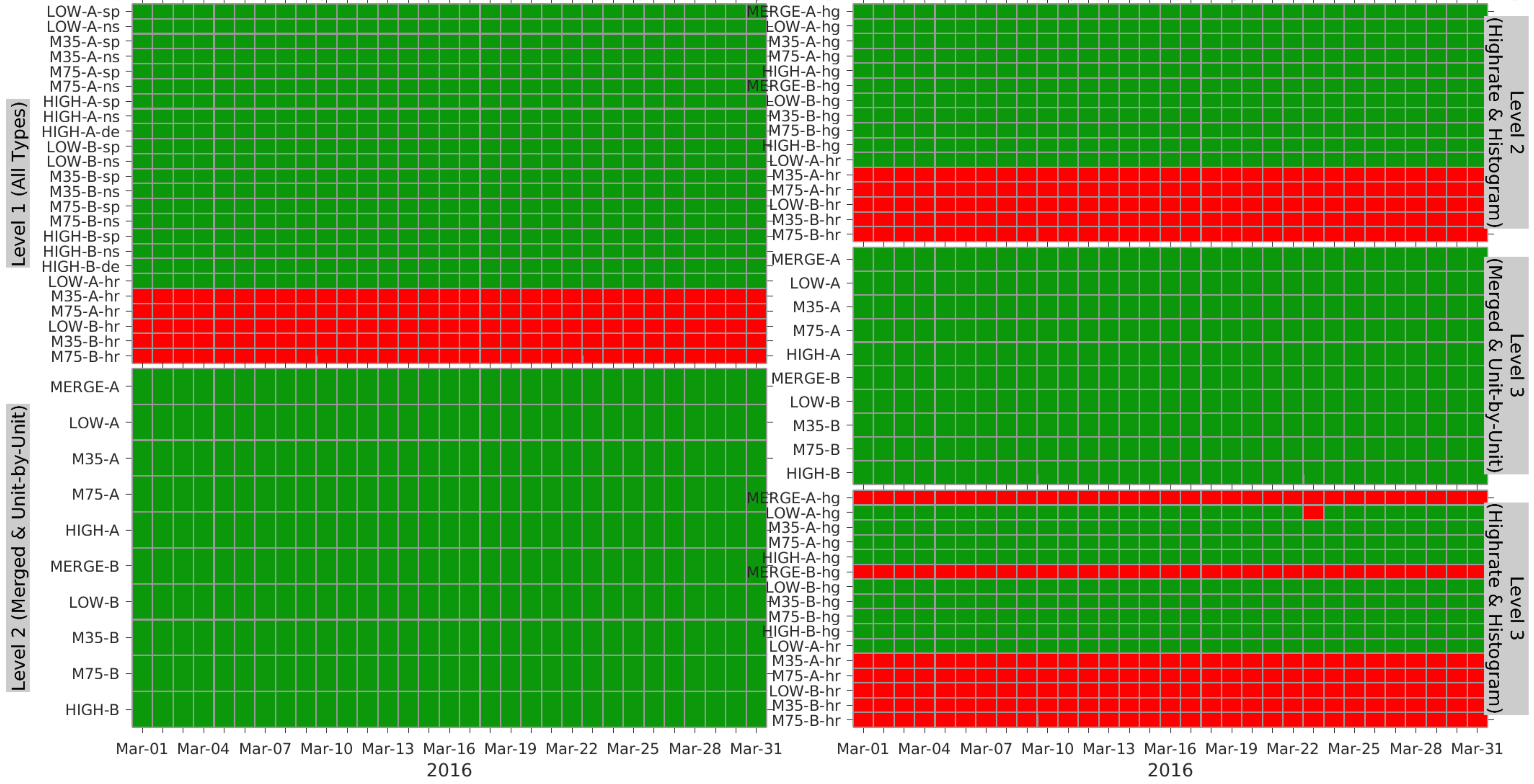

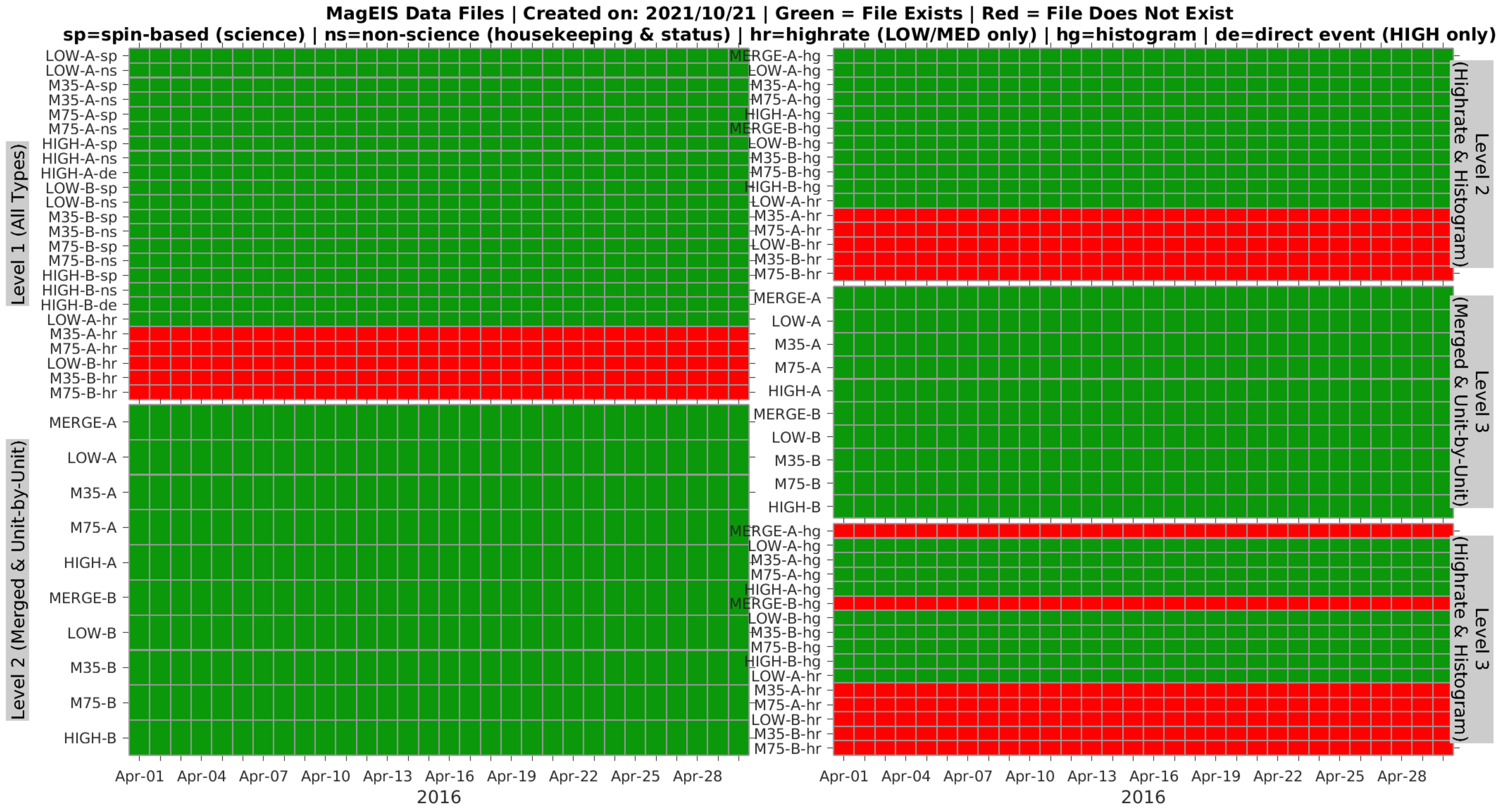
MagEIS Data Files | Created on: 2021/10/21 | Green = File Exists | Red = File Does Not Exist
sp=spin-based (science) | ns=non-science (housekeeping & status) | hr=highrate (LOW/MED only) | hg=histogram | de=direct event (HIGH only)
Level 1 (All Types)
LOW-A-sp
LOW-A-ns
M35-A-sp
M35-A-ns
M75-A-sp
M75-A-ns
HIGH-A-sp
HIGH-A-ns
HIGH-A-de
LOW-B-sp
LOW-B-ns
M35-B-sp
M35-B-ns
M75-B-sp
M75-B-ns
HIGH-B-sp
HIGH-B-ns
HIGH-B-de
LOW-A-hr
M35-A-hr
M75-A-hr
LOW-B-hr
M35-B-hr
M75-B-hr
Level 2 (Merged & Unit-by-Unit)
MERGE-A
LOW-A
M35-A
M75-A
HIGH-A
MERGE-B
LOW-B
M35-B
M75-B
HIGH-B
Level 2 (Highrate & Histogram)
MERGE-A-hg
LOW-A-hg
M35-A-hg
M75-A-hg
HIGH-A-hg
MERGE-B-hg
LOW-B-hg
M35-B-hg
M75-B-hg
HIGH-B-hg
LOW-A-hr
M35-A-hr
M75-A-hr
LOW-B-hr
M35-B-hr
M75-B-hr
Level 3 (Merged & Unit-by-Unit)
MERGE-A
LOW-A
M35-A
M75-A
HIGH-A
MERGE-B
LOW-B
M35-B
M75-B
HIGH-B
Level 3 (Highrate & Histogram)
MERGE-A-hg
LOW-A-hg
M35-A-hg
M75-A-hg
HIGH-A-hg
MERGE-B-hg
LOW-B-hg
M35-B-hg
M75-B-hg
HIGH-B-hg
LOW-A-hr
M35-A-hr
M75-A-hr
LOW-B-hr
M35-B-hr
M75-B-hr
Apr-01 Apr-04 Apr-07 Apr-10 Apr-13 Apr-16 Apr-19 Apr-22 Apr-25 Apr-28
2016
Apr-01 Apr-04 Apr-07 Apr-10 Apr-13 Apr-16 Apr-19 Apr-22 Apr-25 Apr-28
2016

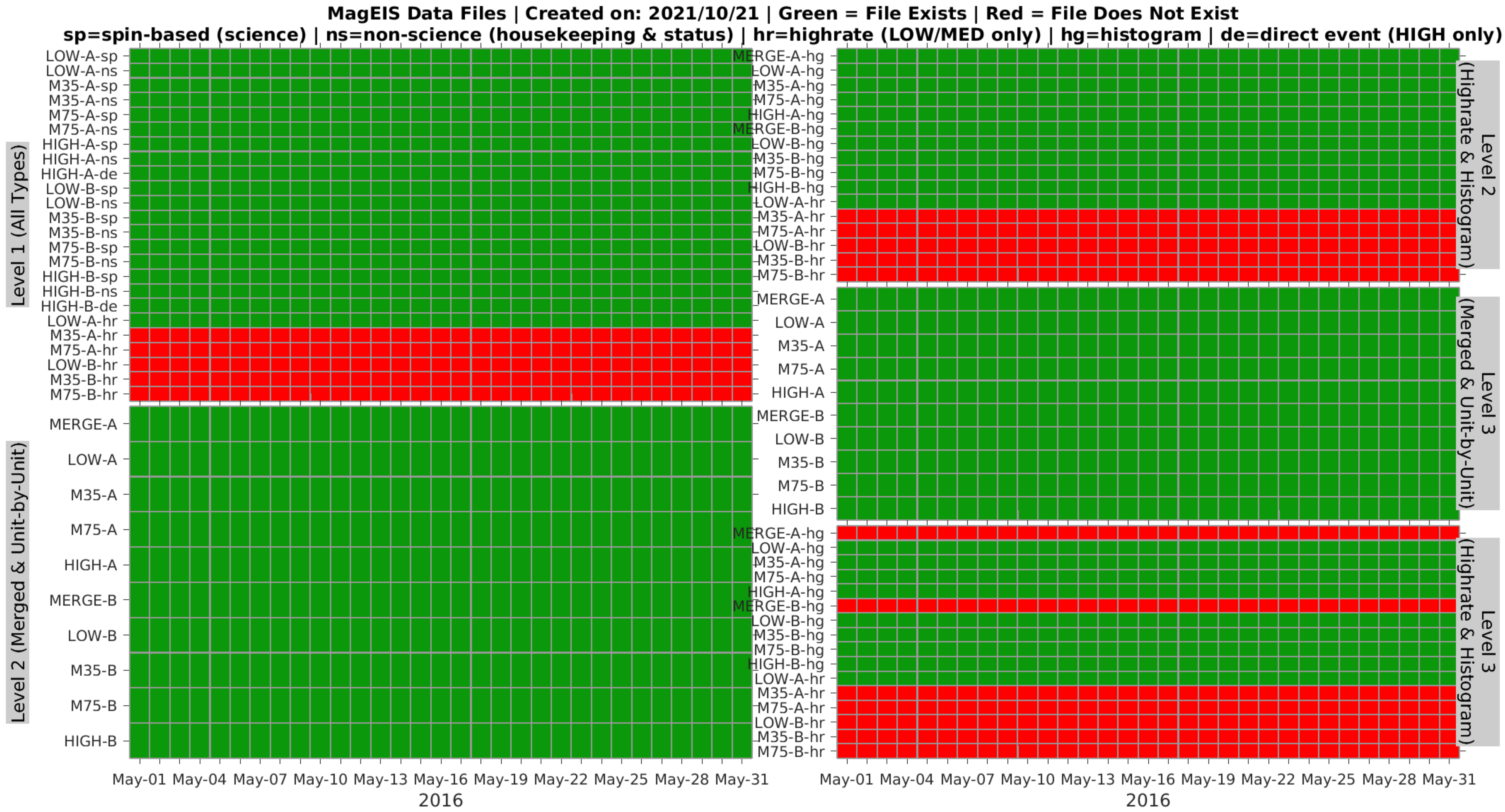
MagEIS Data Files | Created on: 2021/10/21 | Green = File Exists | Red = File Does Not Exist
sp=spin-based (science) | ns=non-science (housekeeping & status) | hr=highrate (LOW/MED only) | hg=histogram | de=direct event (HIGH only)
Level 1 (All Types)
LOW-A-sp
LOW-A-ns
M35-A-sp
M35-A-ns
M75-A-sp
M75-A-ns
HIGH-A-sp
HIGH-A-ns
HIGH-A-de
LOW-B-sp
LOW-B-ns
M35-B-sp
M35-B-ns
M75-B-sp
M75-B-ns
HIGH-B-sp
HIGH-B-ns
HIGH-B-de
LOW-A-hr
M35-A-hr
M75-A-hr
LOW-B-hr
M35-B-hr
M75-B-hr
Level 2 (Merged & Unit-by-Unit)
MERGE-A
LOW-A
M35-A
M75-A
HIGH-A
MERGE-B
LOW-B
M35-B
M75-B
HIGH-B
May-01 May-04 May-07 May-10 May-13 May-16 May-19 May-22 May-25 May-28 May-31
2016
Level 2 (Highrate & Histogram)
MERGE-A-hg
LOW-A-hg
M35-A-hg
M75-A-hg
HIGH-A-hg
MERGE-B-hg
LOW-B-hg
M35-B-hg
M75-B-hg
HIGH-B-hg
LOW-A-hr
M35-A-hr
M75-A-hr
LOW-B-hr
M35-B-hr
M75-B-hr
Level 3 (Merged & Unit-by-Unit)
MERGE-A
LOW-A
M35-A
M75-A
HIGH-A
MERGE-B
LOW-B
M35-B
M75-B
HIGH-B
Level 3 (Highrate & Histogram)
MERGE-A-hg
LOW-A-hg
M35-A-hg
M75-A-hg
HIGH-A-hg
MERGE-B-hg
LOW-B-hg
M35-B-hg
M75-B-hg
HIGH-B-hg
LOW-A-hr
M35-A-hr
M75-A-hr
LOW-B-hr
M35-B-hr
M75-B-hr
May-01 May-04 May-07 May-10 May-13 May-16 May-19 May-22 May-25 May-28 May-31
2016

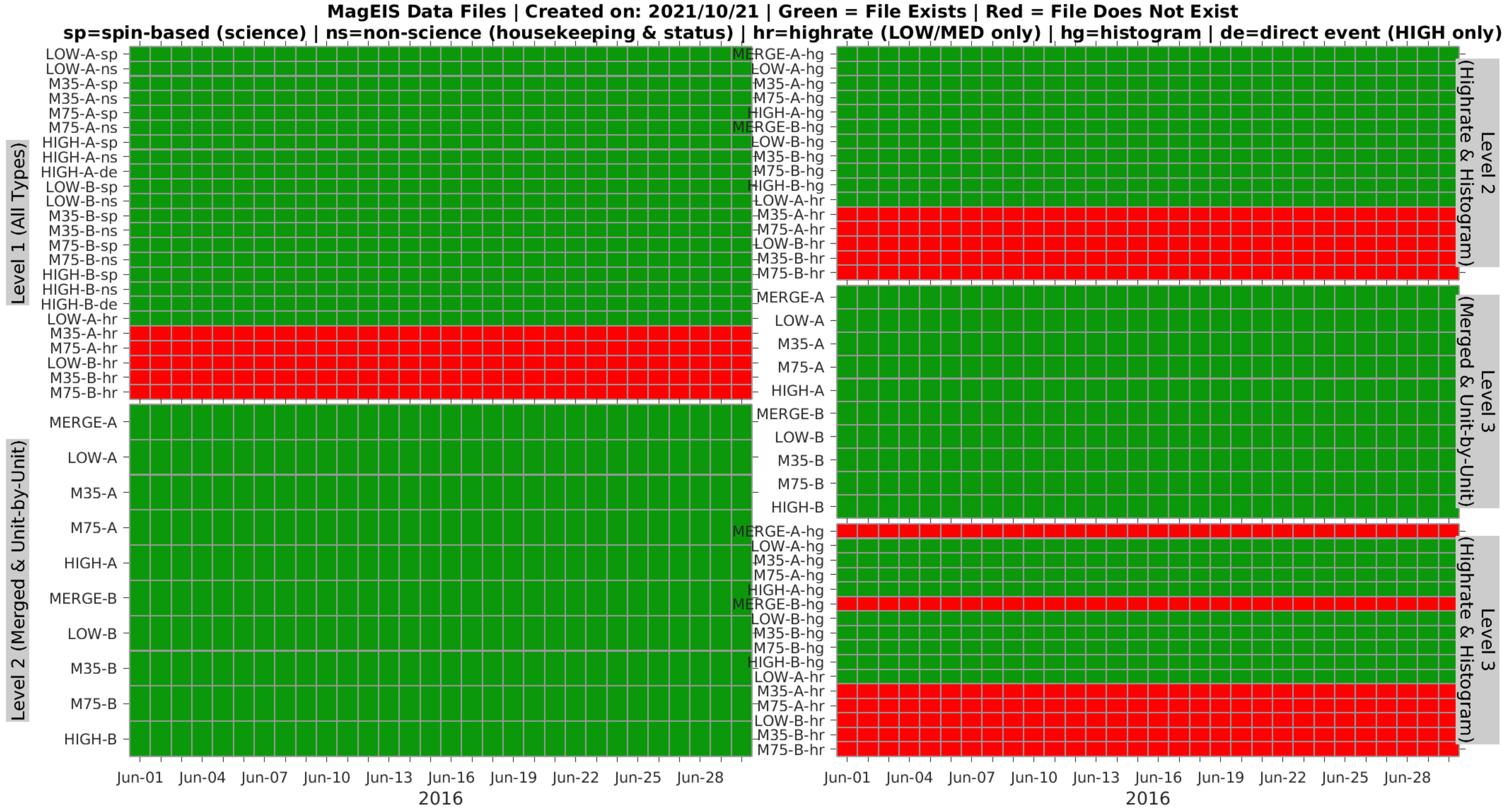
MagEIS Data Files | Created on: 2021/10/21 | Green = File Exists | Red = File Does Not Exist
sp=spin-based (science) | ns=non-science (housekeeping & status) | hr=highrate (LOW/MED only) | hg=histogram | de=direct event (HIGH only)
Level 1 (All Types)
LOW-A-sp
LOW-A-ns
M35-A-sp
M35-A-ns
M75-A-sp
M75-A-ns
HIGH-A-sp
HIGH-A-ns
HIGH-A-de
LOW-B-sp
LOW-B-ns
M35-B-sp
M35-B-ns
M75-B-sp
M75-B-ns
HIGH-B-sp
HIGH-B-ns
HIGH-B-de
LOW-A-hr
M35-A-hr
M75-A-hr
LOW-B-hr
M35-B-hr
M75-B-hr
Level 2 (Merged & Unit-by-Unit)
MERGE-A
LOW-A
M35-A
M75-A
HIGH-A
MERGE-B
LOW-B
M35-B
M75-B
HIGH-B
Jun-01
Jun-04
Jun-07
Jun-10
Jun-13
Jun-16
Jun-19
Jun-22
Jun-25
Jun-28
2016
Level 2 (Highrate & Histogram)
MERGE-A-hg
LOW-A-hg
M35-A-hg
M75-A-hg
HIGH-A-hg
MERGE-B-hg
LOW-B-hg
M35-B-hg
M75-B-hg
HIGH-B-hg
LOW-A-hr
M35-A-hr
M75-A-hr
LOW-B-hr
M35-B-hr
M75-B-hr
Level 3 (Merged & Unit-by-Unit)
MERGE-A
LOW-A
M35-A
M75-A
HIGH-A
MERGE-B
LOW-B
M35-B
M75-B
HIGH-B
Level 3 (Highrate & Histogram)
MERGE-A-hg
LOW-A-hg
M35-A-hg
M75-A-hg
HIGH-A-hg
MERGE-B-hg
LOW-B-hg
M35-B-hg
M75-B-hg
HIGH-B-hg
LOW-A-hr
M35-A-hr
M75-A-hr
LOW-B-hr
M35-B-hr
M75-B-hr
Jun-01
Jun-04
Jun-07
Jun-10
Jun-13
Jun-16
Jun-19
Jun-22
Jun-25
Jun-28
2016

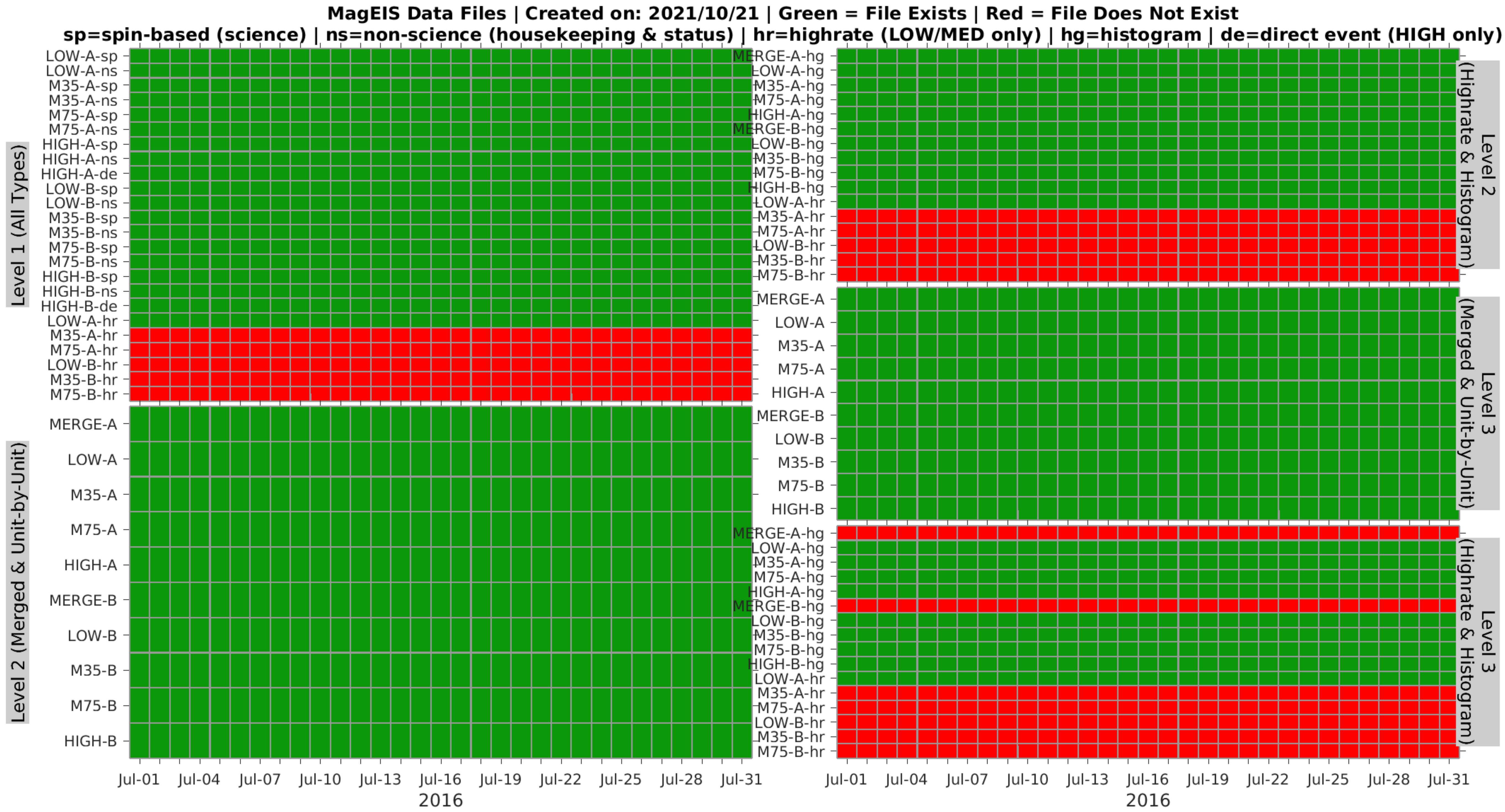

MagEIS Data Files | Created on: 2021/10/21 | Green = File Exists | Red = File Does Not Exist
sp=spin-based (science) | ns=non-science (housekeeping & status) | hr=highrate (LOW/MED only) | hg=histogram | de=direct event (HIGH only)
Level 1 (All Types)
LOW-A-sp
LOW-A-ns
M35-A-sp
M35-A-ns
M75-A-sp
M75-A-ns
HIGH-A-sp
HIGH-A-ns
HIGH-A-de
LOW-B-sp
LOW-B-ns
M35-B-sp
M35-B-ns
M75-B-sp
M75-B-ns
HIGH-B-sp
HIGH-B-ns
HIGH-B-de
LOW-A-hr
M35-A-hr
M75-A-hr
LOW-B-hr
M35-B-hr
M75-B-hr
Level 2 (Merged & Unit-by-Unit)
MERGE-A
LOW-A
M35-A
M75-A
HIGH-A
MERGE-B
LOW-B
M35-B
M75-B
HIGH-B
Level 2 (Highrate & Histogram)
MERGE-A-hg
LOW-A-hg
M35-A-hg
M75-A-hg
HIGH-A-hg
MERGE-B-hg
LOW-B-hg
M35-B-hg
M75-B-hg
HIGH-B-hg
LOW-A-hr
M35-A-hr
M75-A-hr
LOW-B-hr
M35-B-hr
M75-B-hr
Level 3 (Merged & Unit-by-Unit)
MERGE-A
LOW-A
M35-A
M75-A
HIGH-A
MERGE-B
LOW-B
M35-B
M75-B
HIGH-B
Level 3 (Highrate & Histogram)
MERGE-A-hg
LOW-A-hg
M35-A-hg
M75-A-hg
HIGH-A-hg
MERGE-B-hg
LOW-B-hg
M35-B-hg
M75-B-hg
HIGH-B-hg
LOW-A-hr
M35-A-hr
M75-A-hr
LOW-B-hr
M35-B-hr
M75-B-hr
Jul-01
Jul-04
Jul-07
Jul-10
Jul-13
Jul-16
Jul-19
Jul-22
Jul-25
Jul-28
Jul-31
2016

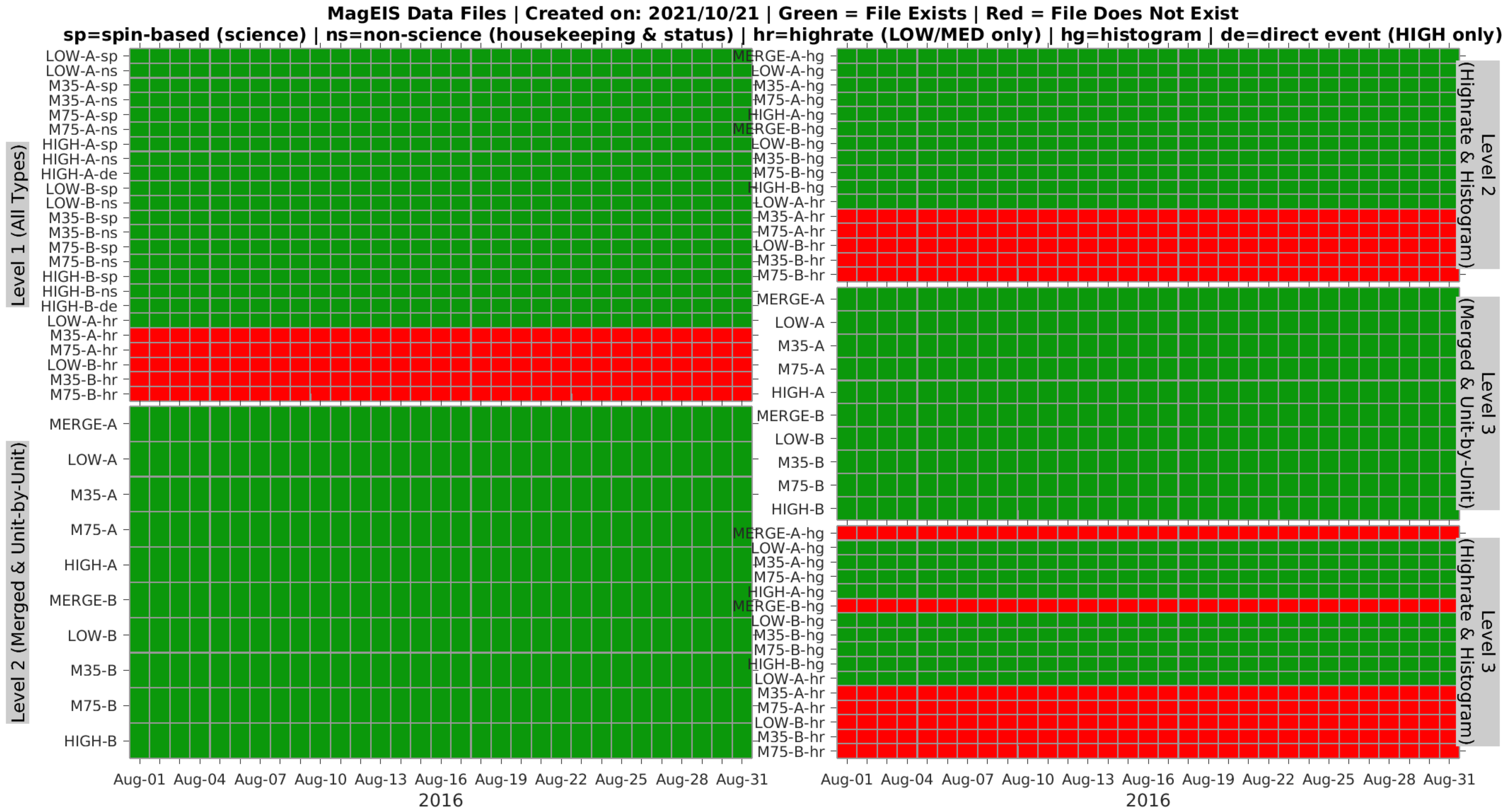
MagEIS Data Files | Created on: 2021/10/21 | Green = File Exists | Red = File Does Not Exist
sp=spin-based (science) | ns=non-science (housekeeping & status) | hr=highrate (LOW/MED only) | hg=histogram | de=direct event (HIGH only)
Level 1 (All Types)
LOW-A-sp
LOW-A-ns
M35-A-sp
M35-A-ns
M75-A-sp
M75-A-ns
HIGH-A-sp
HIGH-A-ns
HIGH-A-de
LOW-B-sp
LOW-B-ns
M35-B-sp
M35-B-ns
M75-B-sp
M75-B-ns
HIGH-B-sp
HIGH-B-ns
HIGH-B-de
LOW-A-hr
M35-A-hr
M75-A-hr
LOW-B-hr
M35-B-hr
M75-B-hr
Level 2 (Merged & Unit-by-Unit)
MERGE-A
LOW-A
M35-A
M75-A
HIGH-A
MERGE-B
LOW-B
M35-B
M75-B
HIGH-B
Level 2 (Highrate & Histogram)
MERGE-A-hg
LOW-A-hg
M35-A-hg
M75-A-hg
HIGH-A-hg
MERGE-B-hg
LOW-B-hg
M35-B-hg
M75-B-hg
HIGH-B-hg
LOW-A-hr
M35-A-hr
M75-A-hr
LOW-B-hr
M35-B-hr
M75-B-hr
Level 3 (Merged & Unit-by-Unit)
MERGE-A
LOW-A
M35-A
M75-A
HIGH-A
MERGE-B
LOW-B
M35-B
M75-B
HIGH-B
Level 3 (Highrate & Histogram)
MERGE-A-hg
LOW-A-hg
M35-A-hg
M75-A-hg
HIGH-A-hg
MERGE-B-hg
LOW-B-hg
M35-B-hg
M75-B-hg
HIGH-B-hg
LOW-A-hr
M35-A-hr
M75-A-hr
LOW-B-hr
M35-B-hr
M75-B-hr
Aug-01
Aug-04
Aug-07
Aug-10
Aug-13
Aug-16
Aug-19
Aug-22
Aug-25
Aug-28
Aug-31
2016

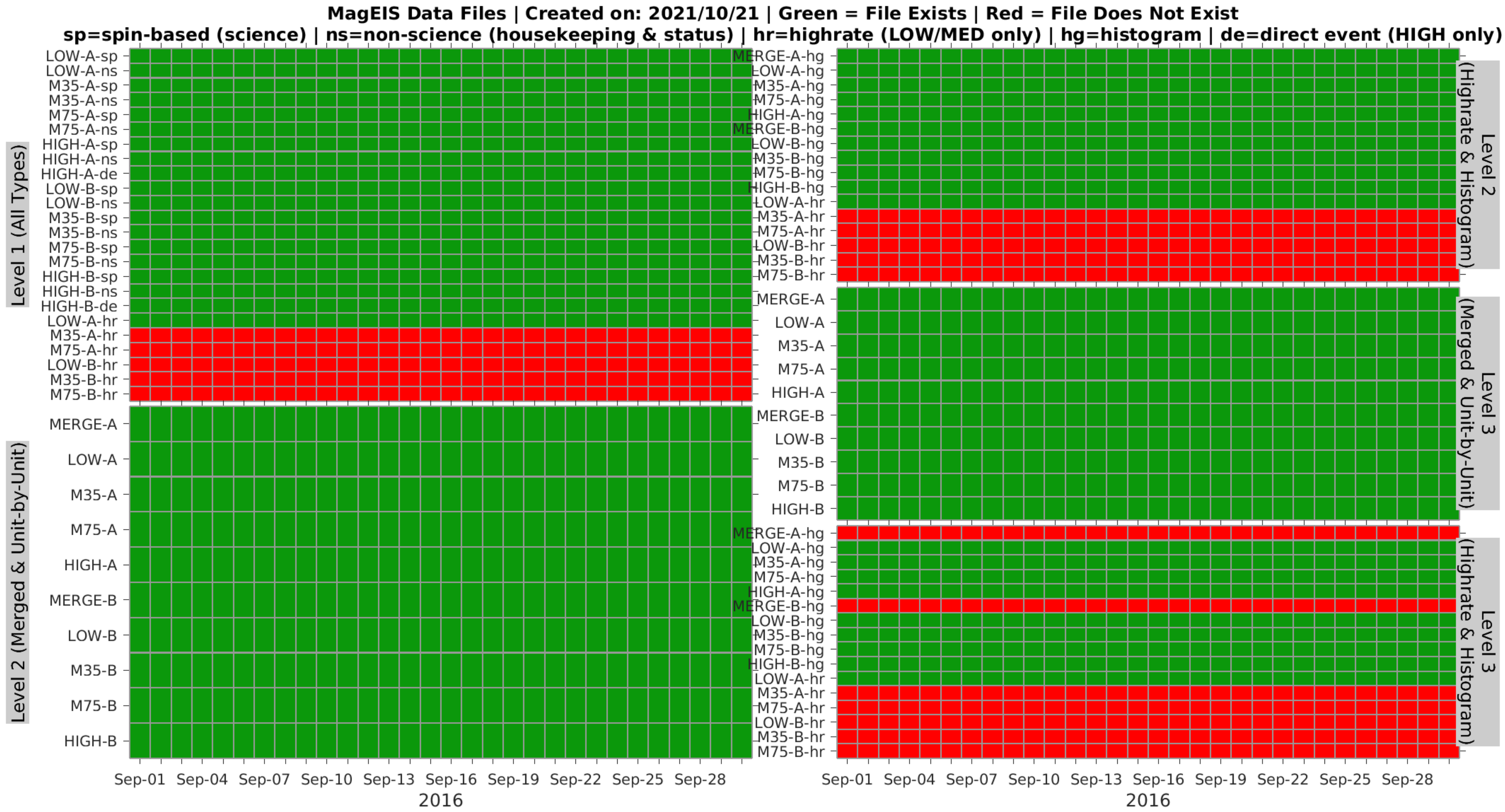
MagEIS Data Files | Created on: 2021/10/21 | Green = File Exists | Red = File Does Not Exist
sp=spin-based (science) | ns=non-science (housekeeping & status) | hr=highrate (LOW/MED only) | hg=histogram | de=direct event (HIGH only)
Level 1 (All Types)
LOW-A-sp
LOW-A-ns
M35-A-sp
M35-A-ns
M75-A-sp
M75-A-ns
HIGH-A-sp
HIGH-A-ns
HIGH-A-de
LOW-B-sp
LOW-B-ns
M35-B-sp
M35-B-ns
M75-B-sp
M75-B-ns
HIGH-B-sp
HIGH-B-ns
HIGH-B-de
LOW-A-hr
M35-A-hr
M75-A-hr
LOW-B-hr
M35-B-hr
M75-B-hr
Level 2 (Merged & Unit-by-Unit)
MERGE-A
LOW-A
M35-A
M75-A
HIGH-A
MERGE-B
LOW-B
M35-B
M75-B
HIGH-B
Level 2 (Highrate & Histogram)
MERGE-A-hg
LOW-A-hg
M35-A-hg
M75-A-hg
HIGH-A-hg
MERGE-B-hg
LOW-B-hg
M35-B-hg
M75-B-hg
HIGH-B-hg
LOW-A-hr
M35-A-hr
M75-A-hr
LOW-B-hr
M35-B-hr
M75-B-hr
Level 3 (Merged & Unit-by-Unit)
MERGE-A
LOW-A
M35-A
M75-A
HIGH-A
MERGE-B
LOW-B
M35-B
M75-B
HIGH-B
Level 3 (Highrate & Histogram)
MERGE-A-hg
LOW-A-hg
M35-A-hg
M75-A-hg
HIGH-A-hg
MERGE-B-hg
LOW-B-hg
M35-B-hg
M75-B-hg
HIGH-B-hg
LOW-A-hr
M35-A-hr
M75-A-hr
LOW-B-hr
M35-B-hr
M75-B-hr
Sep-01
Sep-04
Sep-07
Sep-10
Sep-13
Sep-16
Sep-19
Sep-22
Sep-25
Sep-28
2016

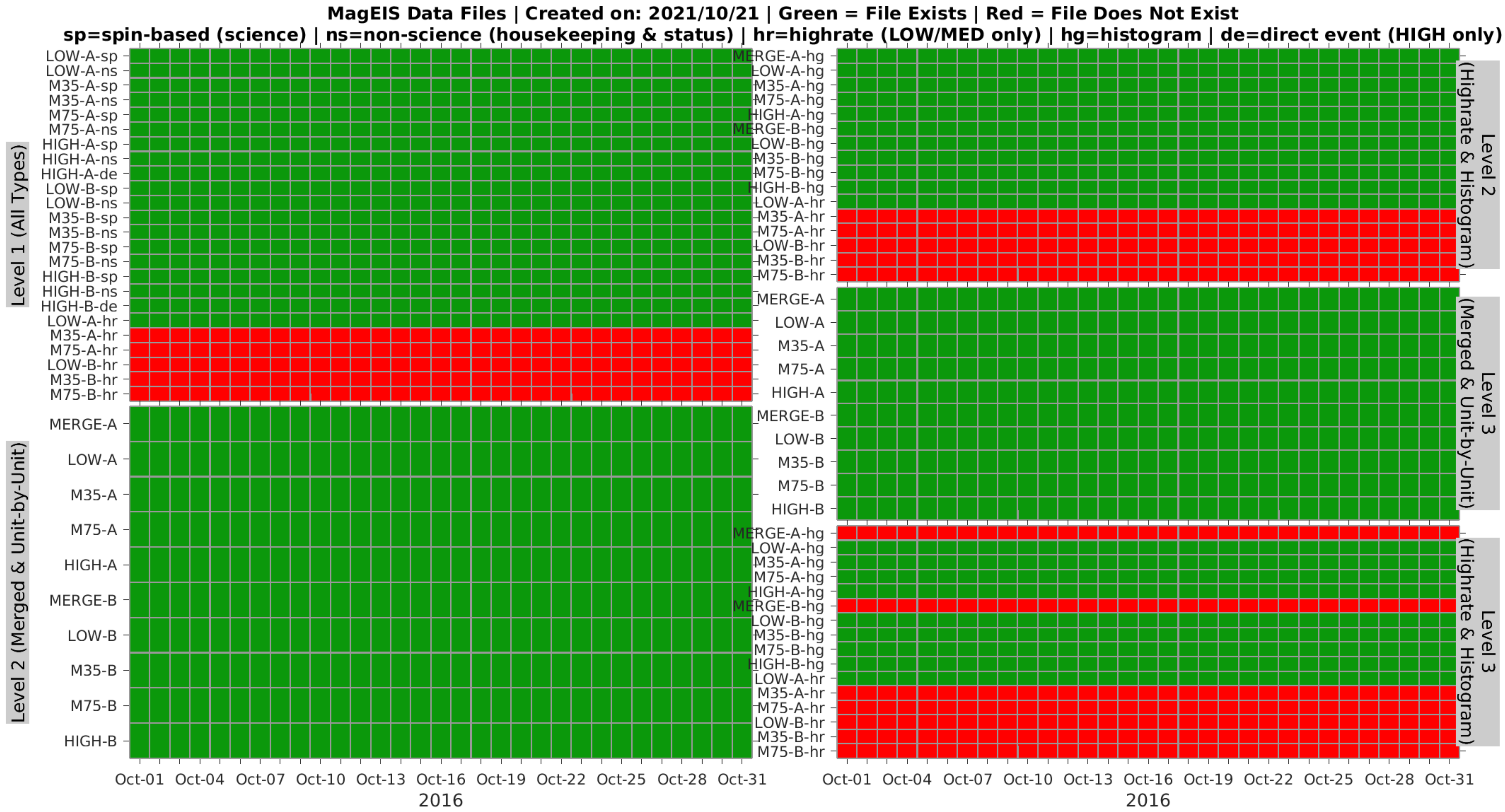

MagEIS Data Files | Created on: 2021/10/21 | Green = File Exists | Red = File Does Not Exist
sp=spin-based (science) | ns=non-science (housekeeping & status) | hr=highrate (LOW/MED only) | hg=histogram | de=direct event (HIGH only)
Level 1 (All Types)
LOW-A-sp
LOW-A-ns
M35-A-sp
M35-A-ns
M75-A-sp
M75-A-ns
HIGH-A-sp
HIGH-A-ns
HIGH-A-de
LOW-B-sp
LOW-B-ns
M35-B-sp
M35-B-ns
M75-B-sp
M75-B-ns
HIGH-B-sp
HIGH-B-ns
HIGH-B-de
LOW-A-hr
M35-A-hr
M75-A-hr
LOW-B-hr
M35-B-hr
M75-B-hr
Level 2 (Merged & Unit-by-Unit)
MERGE-A
LOW-A
M35-A
M75-A
HIGH-A
MERGE-B
LOW-B
M35-B
M75-B
HIGH-B
Oct-01
Oct-04
Oct-07
Oct-10
Oct-13
Oct-16
Oct-19
Oct-22
Oct-25
Oct-28
Oct-31
2016
Level 2 (Highrate & Histogram)
MERGE-A-hg
LOW-A-hg
M35-A-hg
M75-A-hg
HIGH-A-hg
MERGE-B-hg
LOW-B-hg
M35-B-hg
M75-B-hg
HIGH-B-hg
LOW-A-hr
M35-A-hr
M75-A-hr
LOW-B-hr
M35-B-hr
M75-B-hr
Level 3 (Merged & Unit-by-Unit)
MERGE-A
LOW-A
M35-A
M75-A
HIGH-A
MERGE-B
LOW-B
M35-B
M75-B
HIGH-B
Level 3 (Highrate & Histogram)
MERGE-A-hg
LOW-A-hg
M35-A-hg
M75-A-hg
HIGH-A-hg
MERGE-B-hg
LOW-B-hg
M35-B-hg
M75-B-hg
HIGH-B-hg
LOW-A-hr
M35-A-hr
M75-A-hr
LOW-B-hr
M35-B-hr
M75-B-hr
Oct-01
Oct-04
Oct-07
Oct-10
Oct-13
Oct-16
Oct-19
Oct-22
Oct-25
Oct-28
Oct-31
2016

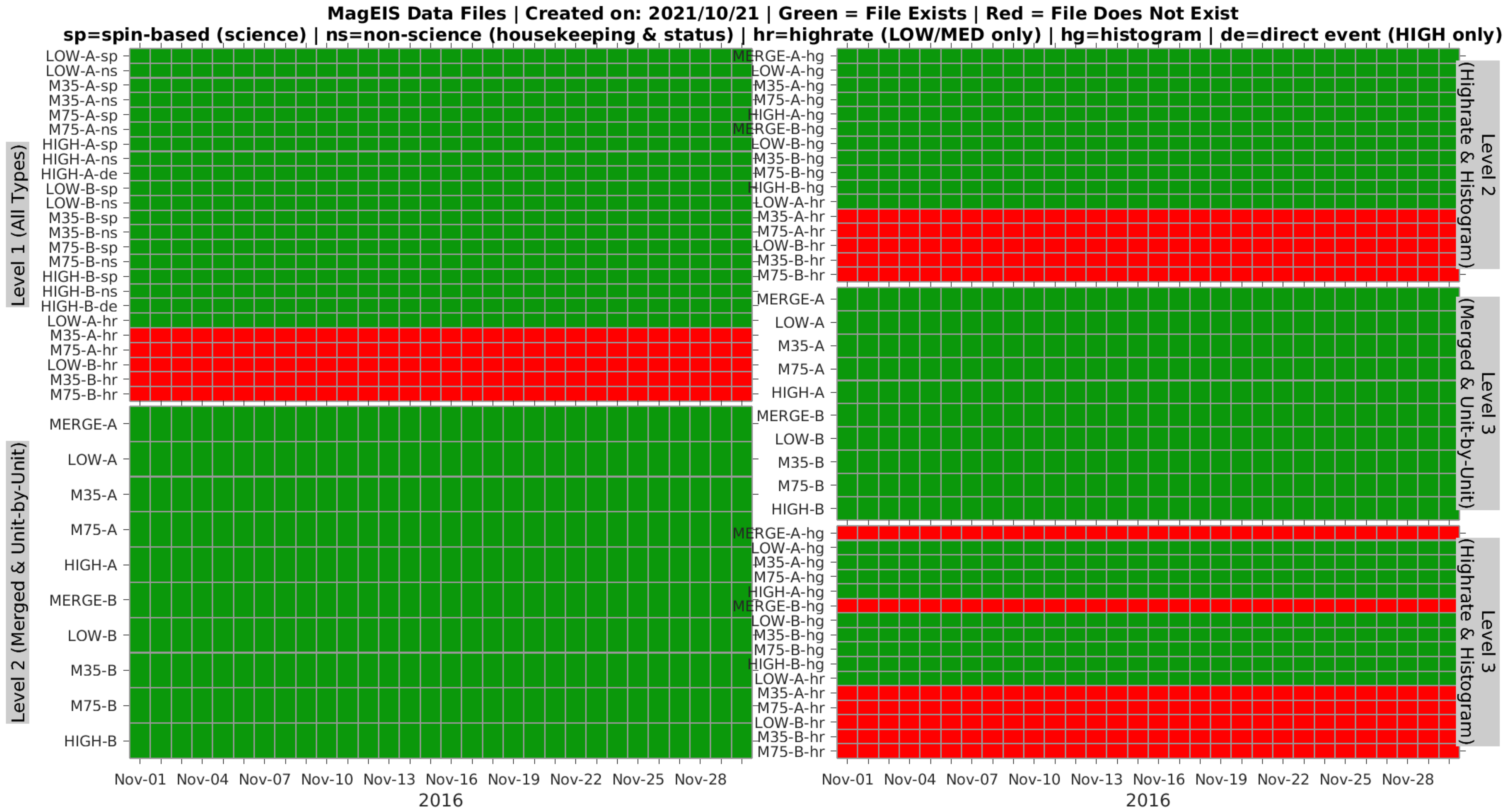

MagEIS Data Files | Created on: 2021/10/21 | Green = File Exists | Red = File Does Not Exist
sp=spin-based (science) | ns=non-science (housekeeping & status) | hr=highrate (LOW/MED only) | hg=histogram | de=direct event (HIGH only)
Level 1 (All Types)
LOW-A-sp
LOW-A-ns
M35-A-sp
M35-A-ns
M75-A-sp
M75-A-ns
HIGH-A-sp
HIGH-A-ns
HIGH-A-de
LOW-B-sp
LOW-B-ns
M35-B-sp
M35-B-ns
M75-B-sp
M75-B-ns
HIGH-B-sp
HIGH-B-ns
HIGH-B-de
LOW-A-hr
M35-A-hr
M75-A-hr
LOW-B-hr
M35-B-hr
M75-B-hr
Level 2 (Merged & Unit-by-Unit)
MERGE-A
LOW-A
M35-A
M75-A
HIGH-A
MERGE-B
LOW-B
M35-B
M75-B
HIGH-B
Level 2 (Highrate & Histogram)
MERGE-A-hg
LOW-A-hg
M35-A-hg
M75-A-hg
HIGH-A-hg
MERGE-B-hg
LOW-B-hg
M35-B-hg
M75-B-hg
HIGH-B-hg
LOW-A-hr
M35-A-hr
M75-A-hr
LOW-B-hr
M35-B-hr
M75-B-hr
Level 3 (Merged & Unit-by-Unit)
MERGE-A
LOW-A
M35-A
M75-A
HIGH-A
MERGE-B
LOW-B
M35-B
M75-B
HIGH-B
Level 3 (Highrate & Histogram)
MERGE-A-hg
LOW-A-hg
M35-A-hg
M75-A-hg
HIGH-A-hg
MERGE-B-hg
LOW-B-hg
M35-B-hg
M75-B-hg
HIGH-B-hg
LOW-A-hr
M35-A-hr
M75-A-hr
LOW-B-hr
M35-B-hr
M75-B-hr
Nov-01
Nov-04
Nov-07
Nov-10
Nov-13
Nov-16
Nov-19
Nov-22
Nov-25
Nov-28
2016

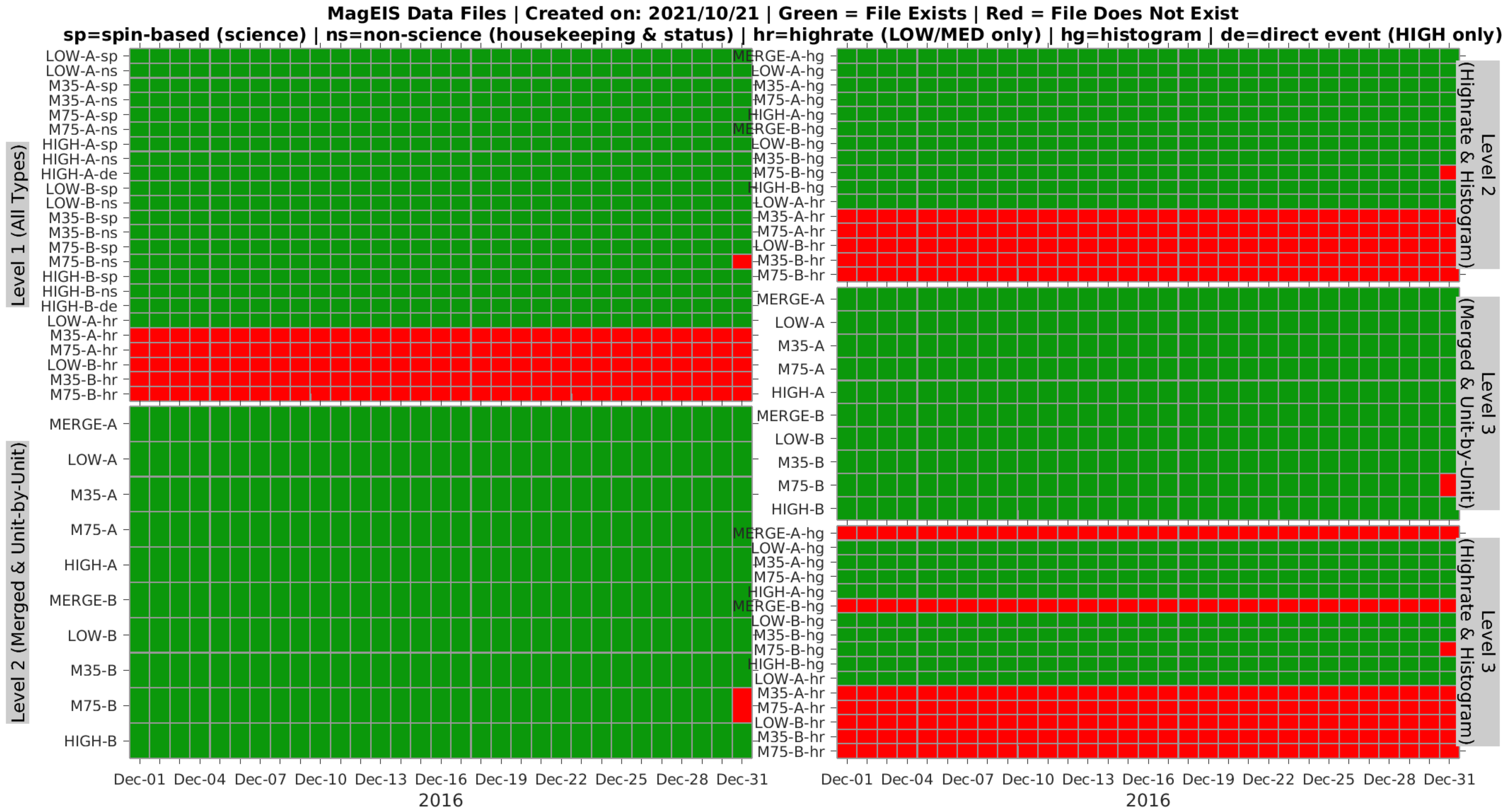
MagEIS Data Files | Created on: 2021/10/21 | Green = File Exists | Red = File Does Not Exist
sp=spin-based (science) | ns=non-science (housekeeping & status) | hr=highrate (LOW/MED only) | hg=histogram | de=direct event (HIGH only)
Level 1 (All Types)
LOW-A-sp
LOW-A-ns
M35-A-sp
M35-A-ns
M75-A-sp
M75-A-ns
HIGH-A-sp
HIGH-A-ns
HIGH-A-de
LOW-B-sp
LOW-B-ns
M35-B-sp
M35-B-ns
M75-B-sp
M75-B-ns
HIGH-B-sp
HIGH-B-ns
HIGH-B-de
LOW-A-hr
M35-A-hr
M75-A-hr
LOW-B-hr
M35-B-hr
M75-B-hr
Level 2 (Merged & Unit-by-Unit)
MERGE-A
LOW-A
M35-A
M75-A
HIGH-A
MERGE-B
LOW-B
M35-B
M75-B
HIGH-B
Level 2 (Highrate & Histogram)
MERGE-A-hg
LOW-A-hg
M35-A-hg
M75-A-hg
HIGH-A-hg
MERGE-B-hg
LOW-B-hg
M35-B-hg
M75-B-hg
HIGH-B-hg
LOW-A-hr
M35-A-hr
M75-A-hr
LOW-B-hr
M35-B-hr
M75-B-hr
Level 3 (Merged & Unit-by-Unit)
MERGE-A
LOW-A
M35-A
M75-A
HIGH-A
MERGE-B
LOW-B
M35-B
M75-B
HIGH-B
Level 3 (Highrate & Histogram)
MERGE-A-hg
LOW-A-hg
M35-A-hg
M75-A-hg
HIGH-A-hg
MERGE-B-hg
LOW-B-hg
M35-B-hg
M75-B-hg
HIGH-B-hg
LOW-A-hr
M35-A-hr
M75-A-hr
LOW-B-hr
M35-B-hr
M75-B-hr
Dec-01 Dec-04 Dec-07 Dec-10 Dec-13 Dec-16 Dec-19 Dec-22 Dec-25 Dec-28 Dec-31
2016

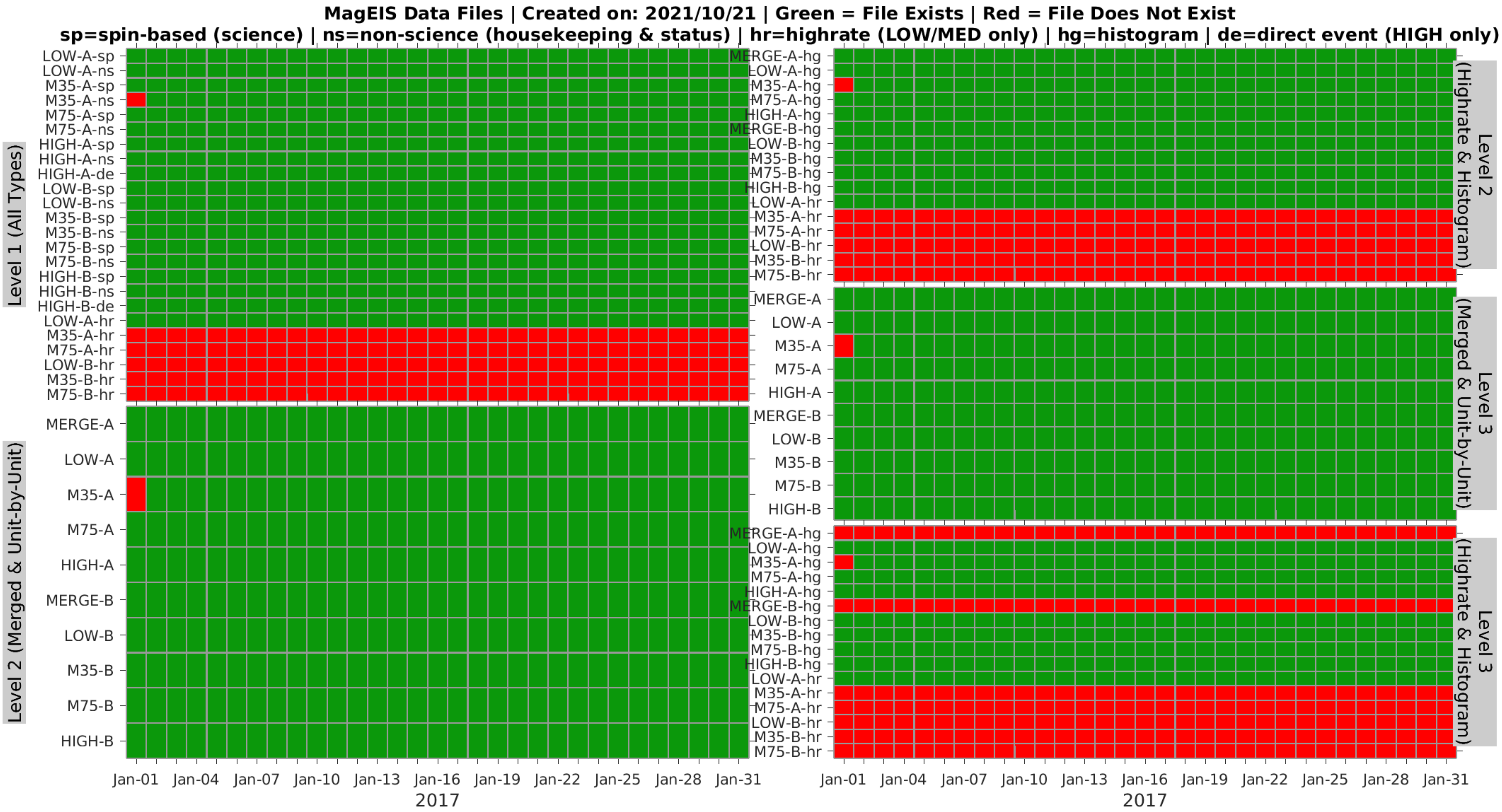
MagEIS Data Files | Created on: 2021/10/21 | Green = File Exists | Red = File Does Not Exist
sp=spin-based (science) | ns=non-science (housekeeping & status) | hr=highrate (LOW/MED only) | hg=histogram | de=direct event (HIGH only)
Level 1 (All Types)
LOW-A-sp
LOW-A-ns
M35-A-sp
M35-A-ns
M75-A-sp
M75-A-ns
HIGH-A-sp
HIGH-A-ns
HIGH-A-de
LOW-B-sp
LOW-B-ns
M35-B-sp
M35-B-ns
M75-B-sp
M75-B-ns
HIGH-B-sp
HIGH-B-ns
HIGH-B-de
LOW-A-hr
M35-A-hr
M75-A-hr
LOW-B-hr
M35-B-hr
M75-B-hr
Level 2 (Merged & Unit-by-Unit)
MERGE-A
LOW-A
M35-A
M75-A
HIGH-A
MERGE-B
LOW-B
M35-B
M75-B
HIGH-B
Level 2 (Highrate & Histogram)
MERGE-A-hg
LOW-A-hg
M35-A-hg
M75-A-hg
HIGH-A-hg
MERGE-B-hg
LOW-B-hg
M35-B-hg
M75-B-hg
HIGH-B-hg
LOW-A-hr
M35-A-hr
M75-A-hr
LOW-B-hr
M35-B-hr
M75-B-hr
Level 3 (Merged & Unit-by-Unit)
MERGE-A
LOW-A
M35-A
M75-A
HIGH-A
MERGE-B
LOW-B
M35-B
M75-B
HIGH-B
Level 3 (Highrate & Histogram)
MERGE-A-hg
LOW-A-hg
M35-A-hg
M75-A-hg
HIGH-A-hg
MERGE-B-hg
LOW-B-hg
M35-B-hg
M75-B-hg
HIGH-B-hg
LOW-A-hr
M35-A-hr
M75-A-hr
LOW-B-hr
M35-B-hr
M75-B-hr
Jan-01
Jan-04
Jan-07
Jan-10
Jan-13
Jan-16
Jan-19
Jan-22
Jan-25
Jan-28
Jan-31
2017

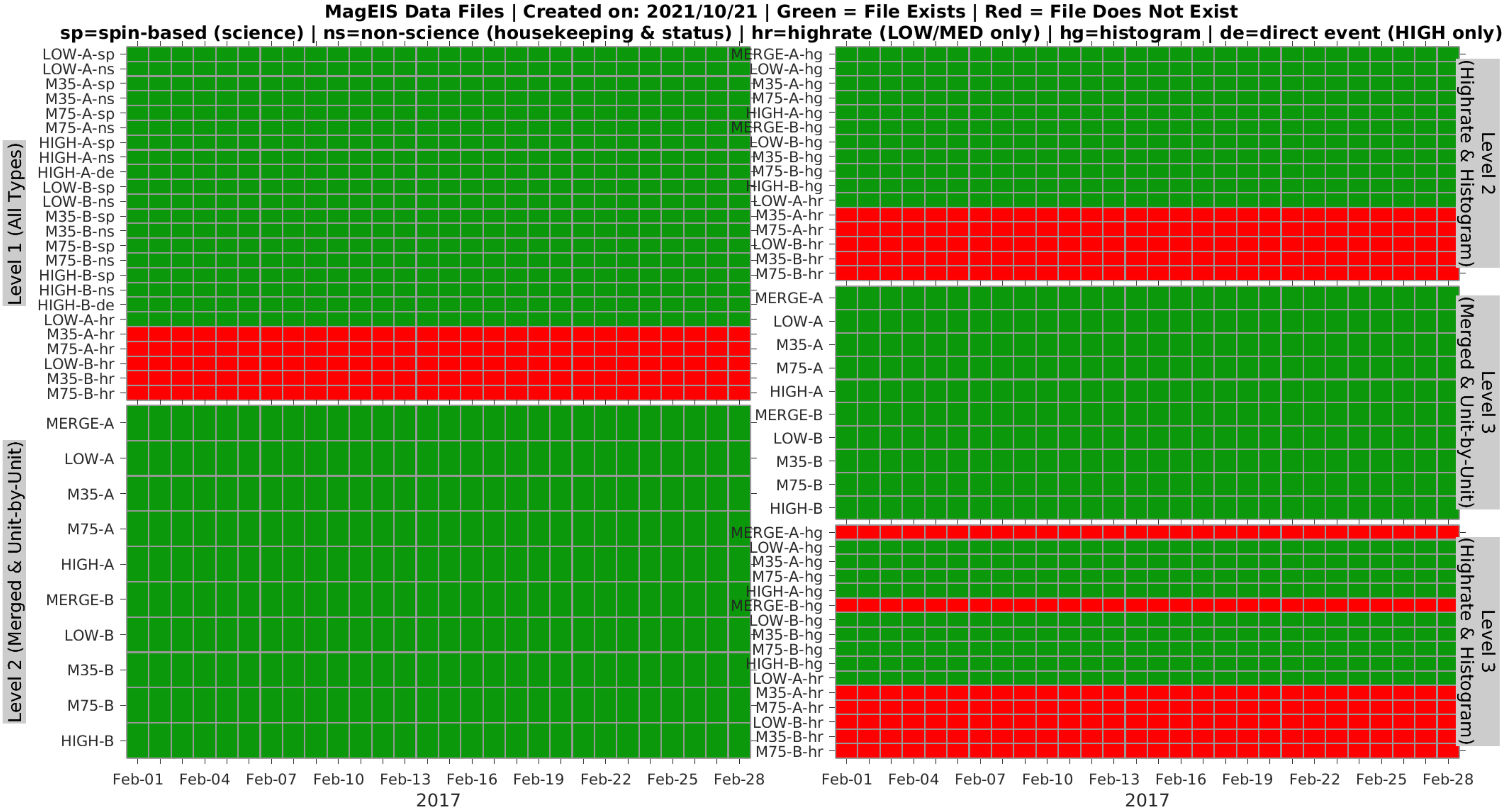
MagEIS Data Files | Created on: 2021/10/21 | Green = File Exists | Red = File Does Not Exist
sp=spin-based (science) | ns=non-science (housekeeping & status) | hr=highrate (LOW/MED only) | hg=histogram | de=direct event (HIGH only)
Level 1 (All Types)
LOW-A-sp
LOW-A-ns
M35-A-sp
M35-A-ns
M75-A-sp
M75-A-ns
HIGH-A-sp
HIGH-A-ns
HIGH-A-de
LOW-B-sp
LOW-B-ns
M35-B-sp
M35-B-ns
M75-B-sp
M75-B-ns
HIGH-B-sp
HIGH-B-ns
HIGH-B-de
LOW-A-hr
M35-A-hr
M75-A-hr
LOW-B-hr
M35-B-hr
M75-B-hr
Level 2 (Merged & Unit-by-Unit)
MERGE-A
LOW-A
M35-A
M75-A
HIGH-A
MERGE-B
LOW-B
M35-B
M75-B
HIGH-B
Level 2 (Highrate & Histogram)
MERGE-A-hg
LOW-A-hg
M35-A-hg
M75-A-hg
HIGH-A-hg
MERGE-B-hg
LOW-B-hg
M35-B-hg
M75-B-hg
HIGH-B-hg
LOW-A-hr
M35-A-hr
M75-A-hr
LOW-B-hr
M35-B-hr
M75-B-hr
Level 3 (Merged & Unit-by-Unit)
MERGE-A
LOW-A
M35-A
M75-A
HIGH-A
MERGE-B
LOW-B
M35-B
M75-B
HIGH-B
Level 3 (Highrate & Histogram)
MERGE-A-hg
LOW-A-hg
M35-A-hg
M75-A-hg
HIGH-A-hg
MERGE-B-hg
LOW-B-hg
M35-B-hg
M75-B-hg
HIGH-B-hg
LOW-A-hr
M35-A-hr
M75-A-hr
LOW-B-hr
M35-B-hr
M75-B-hr
Feb-01
Feb-04
Feb-07
Feb-10
Feb-13
Feb-16
Feb-19
Feb-22
Feb-25
Feb-28
2017

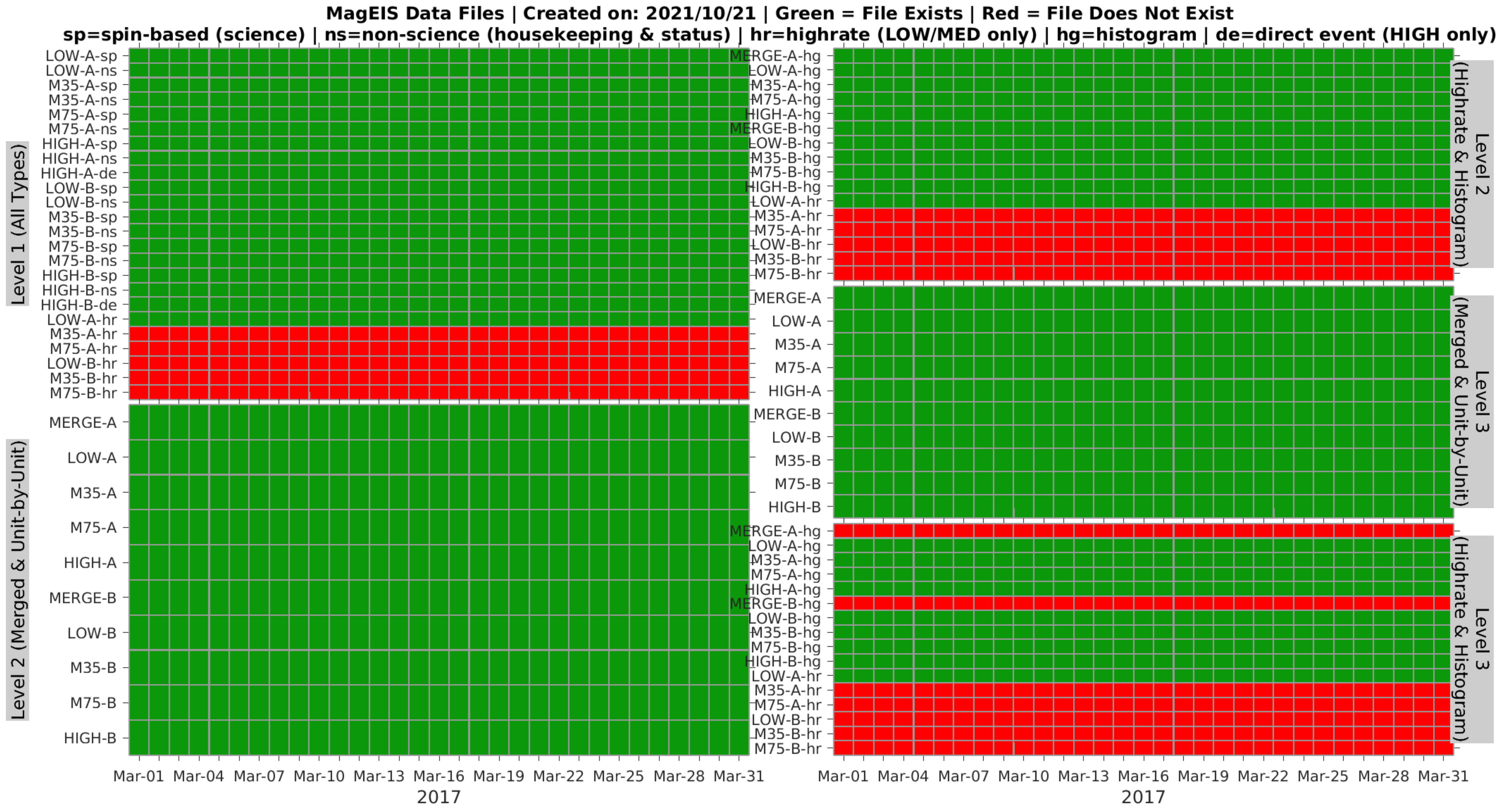
MagEIS Data Files | Created on: 2021/10/21 | Green = File Exists | Red = File Does Not Exist
sp=spin-based (science) | ns=non-science (housekeeping & status) | hr=highrate (LOW/MED only) | hg=histogram | de=direct event (HIGH only)
Level 1 (All Types)
LOW-A-sp
LOW-A-ns
M35-A-sp
M35-A-ns
M75-A-sp
M75-A-ns
HIGH-A-sp
HIGH-A-ns
HIGH-A-de
LOW-B-sp
LOW-B-ns
M35-B-sp
M35-B-ns
M75-B-sp
M75-B-ns
HIGH-B-sp
HIGH-B-ns
HIGH-B-de
LOW-A-hr
M35-A-hr
M75-A-hr
LOW-B-hr
M35-B-hr
M75-B-hr
Level 2 (Merged & Unit-by-Unit)
MERGE-A
LOW-A
M35-A
M75-A
HIGH-A
MERGE-B
LOW-B
M35-B
M75-B
HIGH-B
Level 2 (Highrate & Histogram)
MERGE-A-hg
LOW-A-hg
M35-A-hg
M75-A-hg
HIGH-A-hg
MERGE-B-hg
LOW-B-hg
M35-B-hg
M75-B-hg
HIGH-B-hg
LOW-A-hr
M35-A-hr
M75-A-hr
LOW-B-hr
M35-B-hr
M75-B-hr
Level 3 (Merged & Unit-by-Unit)
MERGE-A
LOW-A
M35-A
M75-A
HIGH-A
MERGE-B
LOW-B
M35-B
M75-B
HIGH-B
Level 3 (Highrate & Histogram)
MERGE-A-hg
LOW-A-hg
M35-A-hg
M75-A-hg
HIGH-A-hg
MERGE-B-hg
LOW-B-hg
M35-B-hg
M75-B-hg
HIGH-B-hg
LOW-A-hr
M35-A-hr
M75-A-hr
LOW-B-hr
M35-B-hr
M75-B-hr
Mar-01
Mar-04
Mar-07
Mar-10
Mar-13
Mar-16
Mar-19
Mar-22
Mar-25
Mar-28
Mar-31
2017

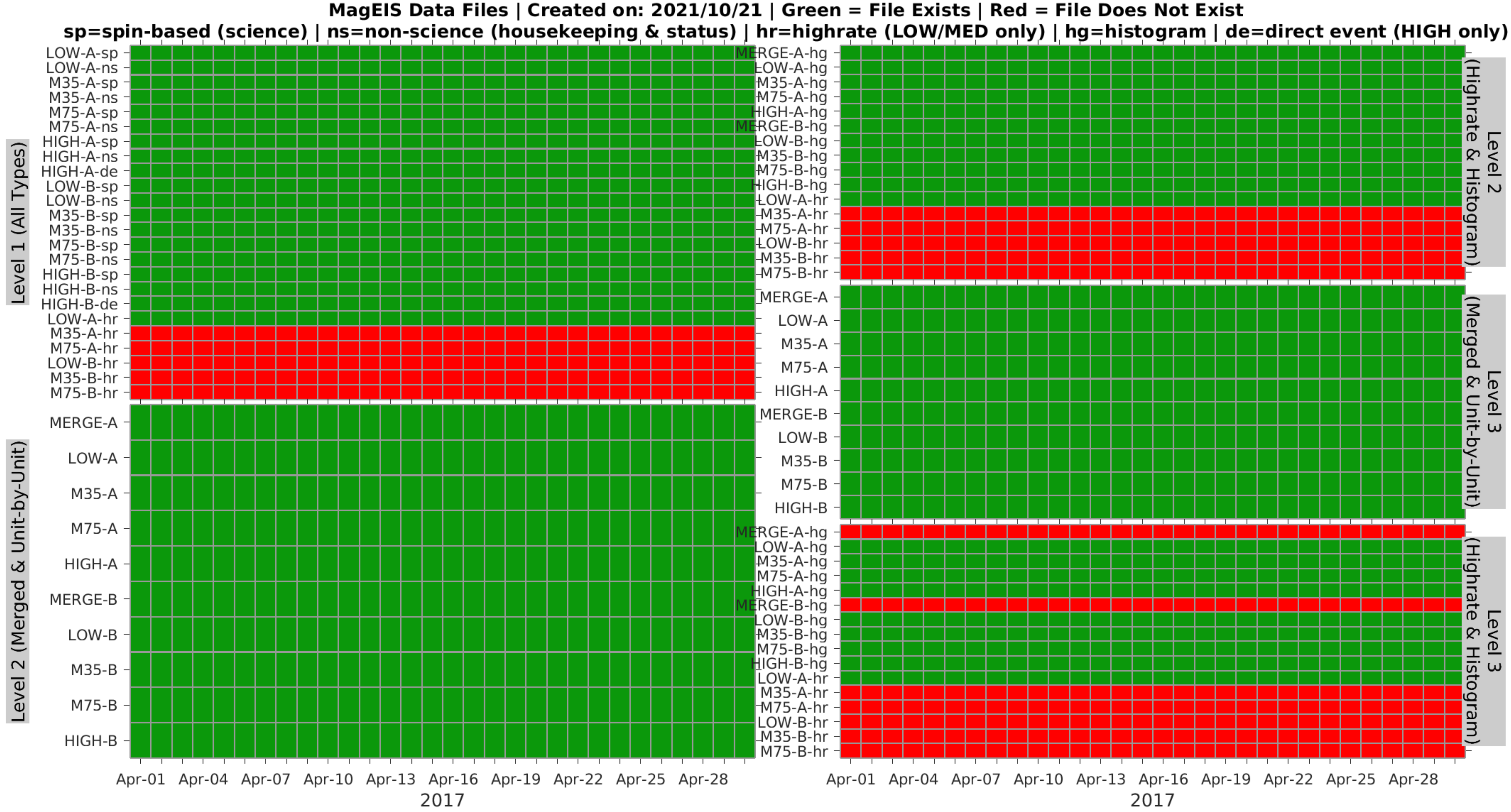

MagEIS Data Files | Created on: 2021/10/21 | Green = File Exists | Red = File Does Not Exist
sp=spin-based (science) | ns=non-science (housekeeping & status) | hr=highrate (LOW/MED only) | hg=histogram | de=direct event (HIGH only)
Level 1 (All Types)
LOW-A-sp
LOW-A-ns
M35-A-sp
M35-A-ns
M75-A-sp
M75-A-ns
HIGH-A-sp
HIGH-A-ns
HIGH-A-de
LOW-B-sp
LOW-B-ns
M35-B-sp
M35-B-ns
M75-B-sp
M75-B-ns
HIGH-B-sp
HIGH-B-ns
HIGH-B-de
LOW-A-hr
M35-A-hr
M75-A-hr
LOW-B-hr
M35-B-hr
M75-B-hr
Level 2 (Merged & Unit-by-Unit)
MERGE-A
LOW-A
M35-A
M75-A
HIGH-A
MERGE-B
LOW-B
M35-B
M75-B
HIGH-B
Level 2 (Highrate & Histogram)
MERGE-A-hg
LOW-A-hg
M35-A-hg
M75-A-hg
HIGH-A-hg
MERGE-B-hg
LOW-B-hg
M35-B-hg
M75-B-hg
HIGH-B-hg
LOW-A-hr
M35-A-hr
M75-A-hr
LOW-B-hr
M35-B-hr
M75-B-hr
Level 3 (Merged & Unit-by-Unit)
MERGE-A
LOW-A
M35-A
M75-A
HIGH-A
MERGE-B
LOW-B
M35-B
M75-B
HIGH-B
Level 3 (Highrate & Histogram)
MERGE-A-hg
LOW-A-hg
M35-A-hg
M75-A-hg
HIGH-A-hg
MERGE-B-hg
LOW-B-hg
M35-B-hg
M75-B-hg
HIGH-B-hg
LOW-A-hr
M35-A-hr
M75-A-hr
LOW-B-hr
M35-B-hr
M75-B-hr
Apr-01
Apr-04
Apr-07
Apr-10
Apr-13
Apr-16
Apr-19
Apr-22
Apr-25
Apr-28
2017

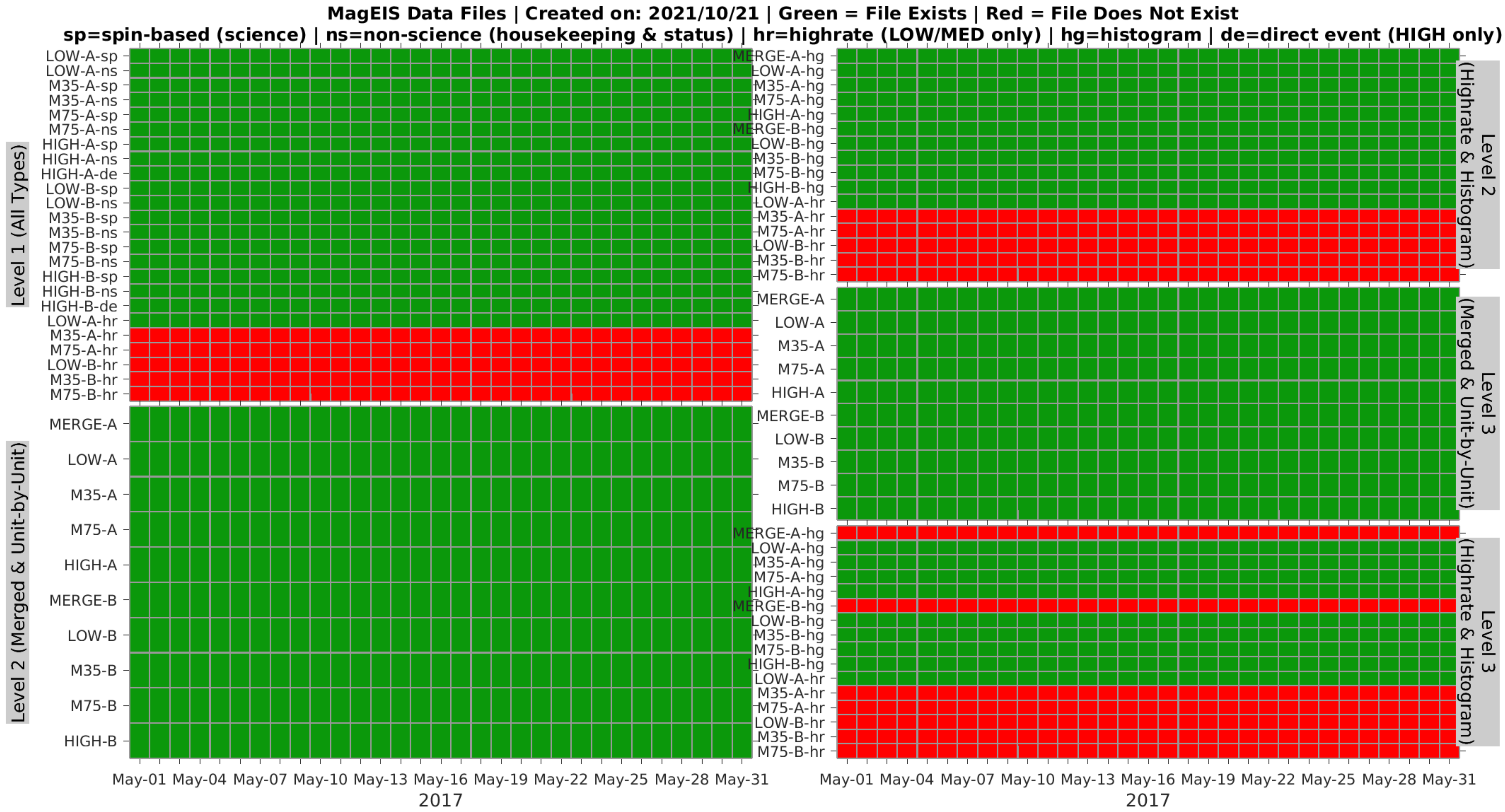

MagEIS Data Files | Created on: 2021/10/21 | Green = File Exists | Red = File Does Not Exist
sp=spin-based (science) | ns=non-science (housekeeping & status) | hr=highrate (LOW/MED only) | hg=histogram | de=direct event (HIGH only)
Level 1 (All Types)
LOW-A-sp
LOW-A-ns
M35-A-sp
M35-A-ns
M75-A-sp
M75-A-ns
HIGH-A-sp
HIGH-A-ns
HIGH-A-de
LOW-B-sp
LOW-B-ns
M35-B-sp
M35-B-ns
M75-B-sp
M75-B-ns
HIGH-B-sp
HIGH-B-ns
HIGH-B-de
LOW-A-hr
M35-A-hr
M75-A-hr
LOW-B-hr
M35-B-hr
M75-B-hr
Level 2 (Merged & Unit-by-Unit)
MERGE-A
LOW-A
M35-A
M75-A
HIGH-A
MERGE-B
LOW-B
M35-B
M75-B
HIGH-B
Level 2 (Highrate & Histogram)
MERGE-A-hg
LOW-A-hg
M35-A-hg
M75-A-hg
HIGH-A-hg
MERGE-B-hg
LOW-B-hg
M35-B-hg
M75-B-hg
HIGH-B-hg
LOW-A-hr
M35-A-hr
M75-A-hr
LOW-B-hr
M35-B-hr
M75-B-hr
Level 3 (Merged & Unit-by-Unit)
MERGE-A
LOW-A
M35-A
M75-A
HIGH-A
MERGE-B
LOW-B
M35-B
M75-B
HIGH-B
Level 3 (Highrate & Histogram)
MERGE-A-hg
LOW-A-hg
M35-A-hg
M75-A-hg
HIGH-A-hg
MERGE-B-hg
LOW-B-hg
M35-B-hg
M75-B-hg
HIGH-B-hg
LOW-A-hr
M35-A-hr
M75-A-hr
LOW-B-hr
M35-B-hr
M75-B-hr
May-01 May-04 May-07 May-10 May-13 May-16 May-19 May-22 May-25 May-28 May-31
2017

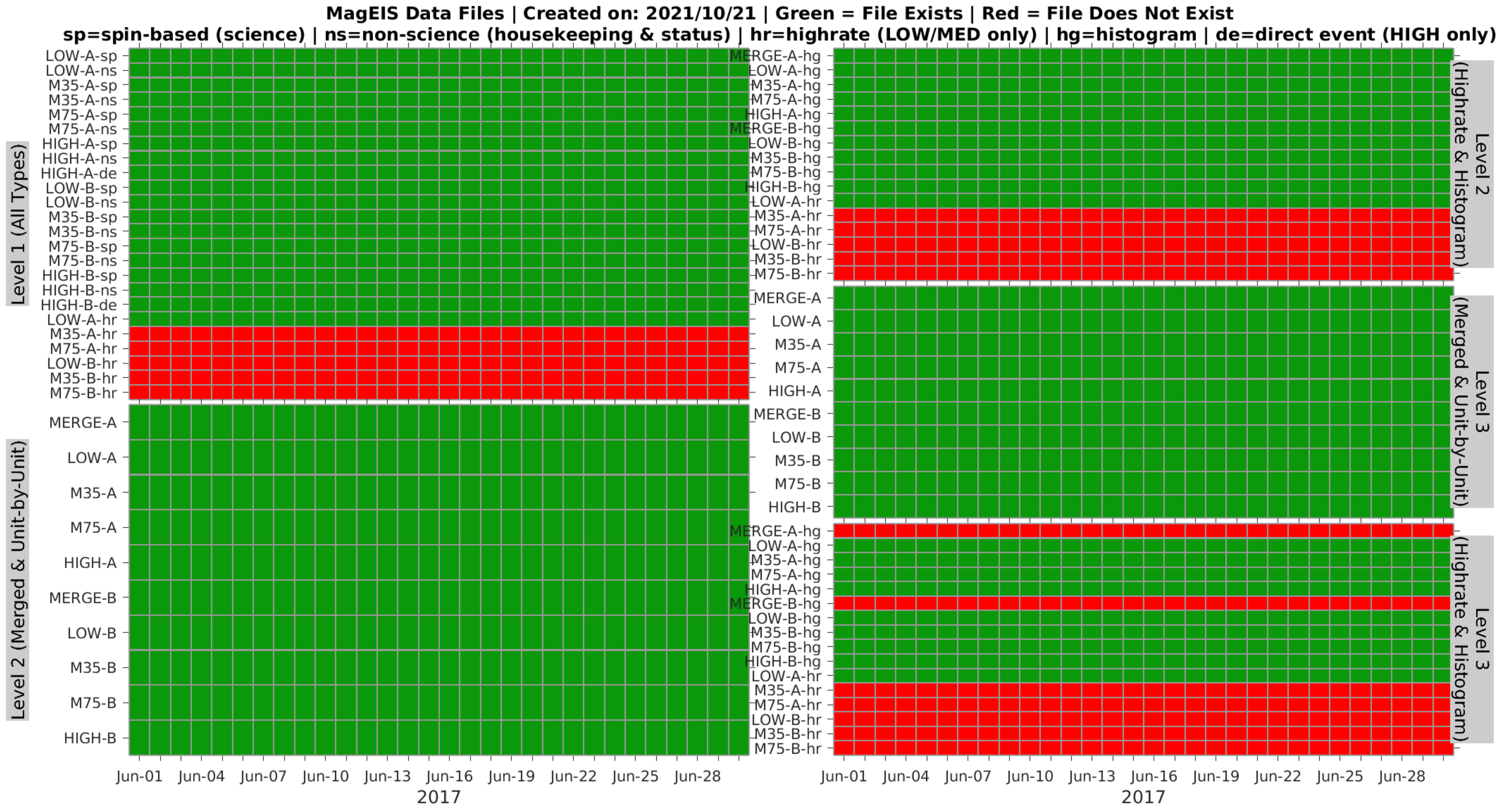
MagEIS Data Files | Created on: 2021/10/21 | Green = File Exists | Red = File Does Not Exist
sp=spin-based (science) | ns=non-science (housekeeping & status) | hr=highrate (LOW/MED only) | hg=histogram | de=direct event (HIGH only)
Level 1 (All Types)
LOW-A-sp
LOW-A-ns
M35-A-sp
M35-A-ns
M75-A-sp
M75-A-ns
HIGH-A-sp
HIGH-A-ns
HIGH-A-de
LOW-B-sp
LOW-B-ns
M35-B-sp
M35-B-ns
M75-B-sp
M75-B-ns
HIGH-B-sp
HIGH-B-ns
HIGH-B-de
LOW-A-hr
M35-A-hr
M75-A-hr
LOW-B-hr
M35-B-hr
M75-B-hr
Level 2 (Merged & Unit-by-Unit)
MERGE-A
LOW-A
M35-A
M75-A
HIGH-A
MERGE-B
LOW-B
M35-B
M75-B
HIGH-B
Level 2 (Highrate & Histogram)
MERGE-A-hg
LOW-A-hg
M35-A-hg
M75-A-hg
HIGH-A-hg
MERGE-B-hg
LOW-B-hg
M35-B-hg
M75-B-hg
HIGH-B-hg
LOW-A-hr
M35-A-hr
M75-A-hr
LOW-B-hr
M35-B-hr
M75-B-hr
Level 3 (Merged & Unit-by-Unit)
MERGE-A
LOW-A
M35-A
M75-A
HIGH-A
MERGE-B
LOW-B
M35-B
M75-B
HIGH-B
Level 3 (Highrate & Histogram)
MERGE-A-hg
LOW-A-hg
M35-A-hg
M75-A-hg
HIGH-A-hg
MERGE-B-hg
LOW-B-hg
M35-B-hg
M75-B-hg
HIGH-B-hg
LOW-A-hr
M35-A-hr
M75-A-hr
LOW-B-hr
M35-B-hr
M75-B-hr
Jun-01
Jun-04
Jun-07
Jun-10
Jun-13
Jun-16
Jun-19
Jun-22
Jun-25
Jun-28
2017

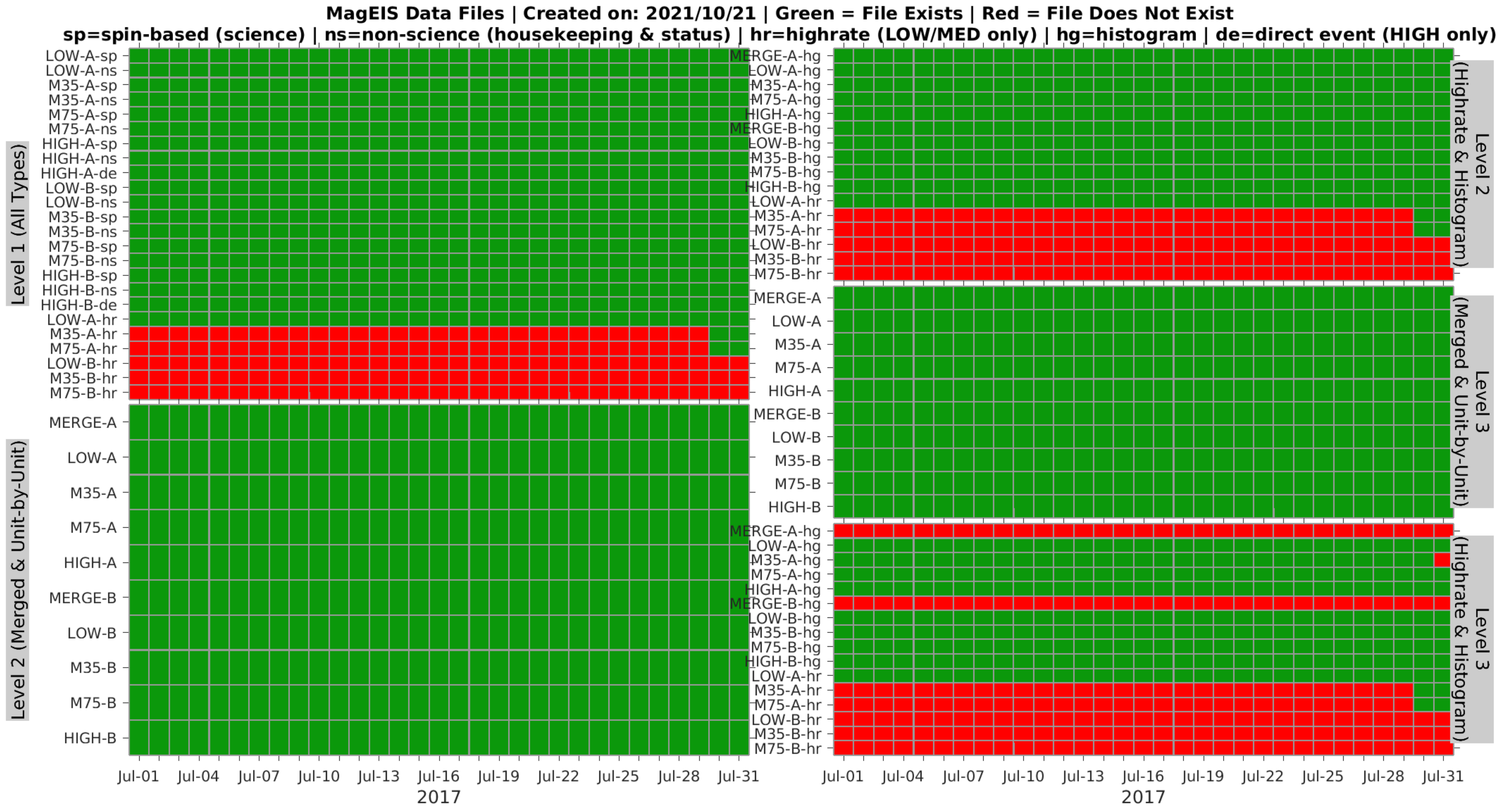

MagEIS Data Files | Created on: 2021/10/21 | Green = File Exists | Red = File Does Not Exist
sp=spin-based (science) | ns=non-science (housekeeping & status) | hr=highrate (LOW/MED only) | hg=histogram | de=direct event (HIGH only)
Level 1 (All Types)
LOW-A-sp
LOW-A-ns
M35-A-sp
M35-A-ns
M75-A-sp
M75-A-ns
HIGH-A-sp
HIGH-A-ns
HIGH-A-de
LOW-B-sp
LOW-B-ns
M35-B-sp
M35-B-ns
M75-B-sp
M75-B-ns
HIGH-B-sp
HIGH-B-ns
HIGH-B-de
LOW-A-hr
M35-A-hr
M75-A-hr
LOW-B-hr
M35-B-hr
M75-B-hr
Level 2 (Merged & Unit-by-Unit)
MERGE-A
LOW-A
M35-A
M75-A
HIGH-A
MERGE-B
LOW-B
M35-B
M75-B
HIGH-B
Level 2 (Highrate & Histogram)
MERGE-A-hg
LOW-A-hg
M35-A-hg
M75-A-hg
HIGH-A-hg
MERGE-B-hg
LOW-B-hg
M35-B-hg
M75-B-hg
HIGH-B-hg
LOW-A-hr
M35-A-hr
M75-A-hr
LOW-B-hr
M35-B-hr
M75-B-hr
Level 3 (Merged & Unit-by-Unit)
MERGE-A
LOW-A
M35-A
M75-A
HIGH-A
MERGE-B
LOW-B
M35-B
M75-B
HIGH-B
Level 3 (Highrate & Histogram)
MERGE-A-hg
LOW-A-hg
M35-A-hg
M75-A-hg
HIGH-A-hg
MERGE-B-hg
LOW-B-hg
M35-B-hg
M75-B-hg
HIGH-B-hg
LOW-A-hr
M35-A-hr
M75-A-hr
LOW-B-hr
M35-B-hr
M75-B-hr
Jul-01
Jul-04
Jul-07
Jul-10
Jul-13
Jul-16
Jul-19
Jul-22
Jul-25
Jul-28
Jul-31
2017

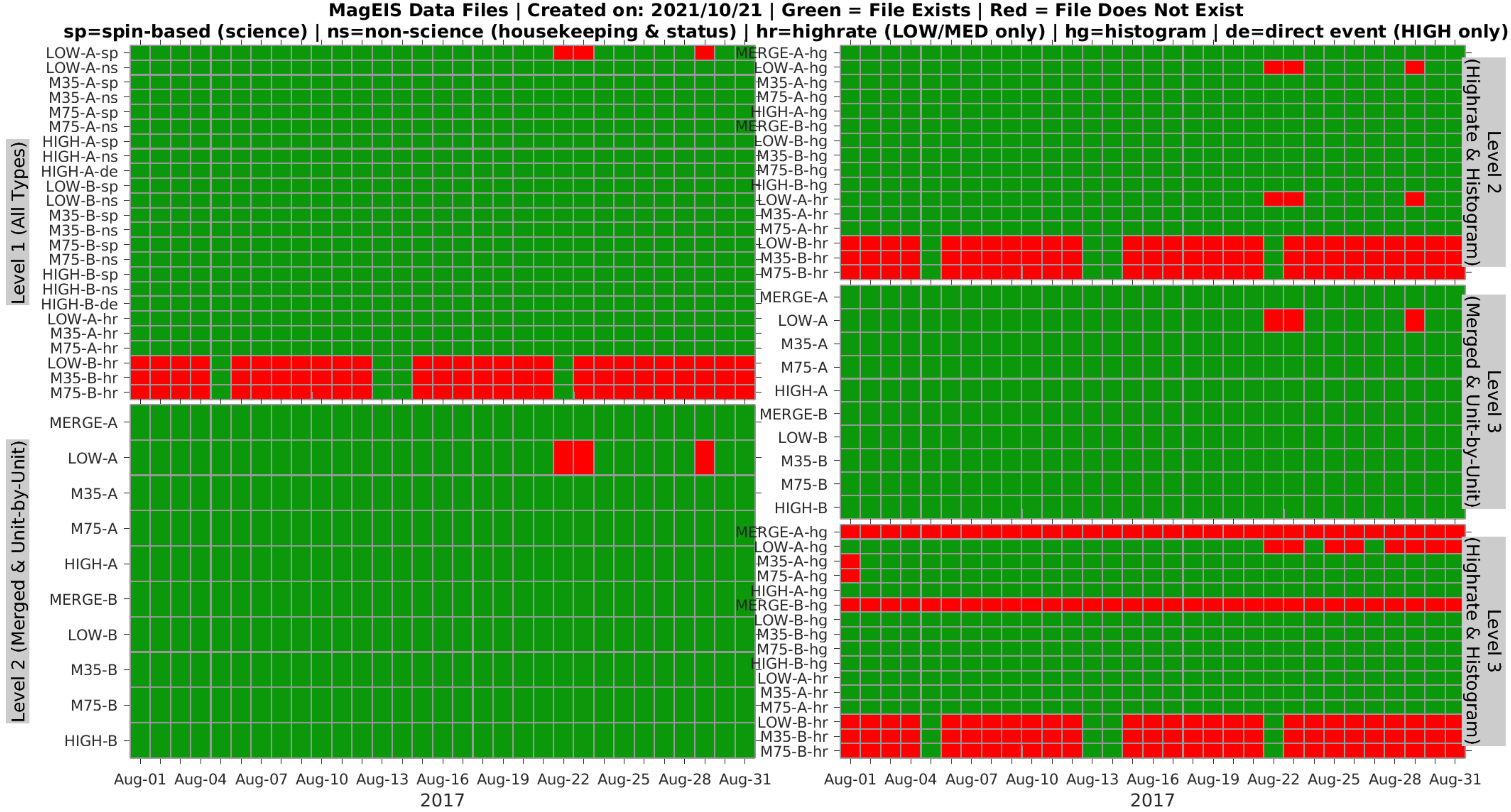
MagEIS Data Files | Created on: 2021/10/21 | Green = File Exists | Red = File Does Not Exist
sp=spin-based (science) | ns=non-science (housekeeping & status) | hr=highrate (LOW/MED only) | hg=histogram | de=direct event (HIGH only)
Level 1 (All Types)
LOW-A-sp
LOW-A-ns
M35-A-sp
M35-A-ns
M75-A-sp
M75-A-ns
HIGH-A-sp
HIGH-A-ns
HIGH-A-de
LOW-B-sp
LOW-B-ns
M35-B-sp
M35-B-ns
M75-B-sp
M75-B-ns
HIGH-B-sp
HIGH-B-ns
HIGH-B-de
LOW-A-hr
M35-A-hr
M75-A-hr
LOW-B-hr
M35-B-hr
M75-B-hr
Level 2 (Merged & Unit-by-Unit)
MERGE-A
LOW-A
M35-A
M75-A
HIGH-A
MERGE-B
LOW-B
M35-B
M75-B
HIGH-B
Level 2 (Highrate & Histogram)
MERGE-A-hg
LOW-A-hg
M35-A-hg
M75-A-hg
HIGH-A-hg
MERGE-B-hg
LOW-B-hg
M35-B-hg
M75-B-hg
HIGH-B-hg
LOW-A-hr
M35-A-hr
M75-A-hr
LOW-B-hr
M35-B-hr
M75-B-hr
Level 3 (Merged & Unit-by-Unit)
MERGE-A
LOW-A
M35-A
M75-A
HIGH-A
MERGE-B
LOW-B
M35-B
M75-B
HIGH-B
Level 3 (Highrate & Histogram)
MERGE-A-hg
LOW-A-hg
M35-A-hg
M75-A-hg
HIGH-A-hg
MERGE-B-hg
LOW-B-hg
M35-B-hg
M75-B-hg
HIGH-B-hg
LOW-A-hr
M35-A-hr
M75-A-hr
LOW-B-hr
M35-B-hr
M75-B-hr
Aug-01 Aug-04 Aug-07 Aug-10 Aug-13 Aug-16 Aug-19 Aug-22 Aug-25 Aug-28 Aug-31
2017

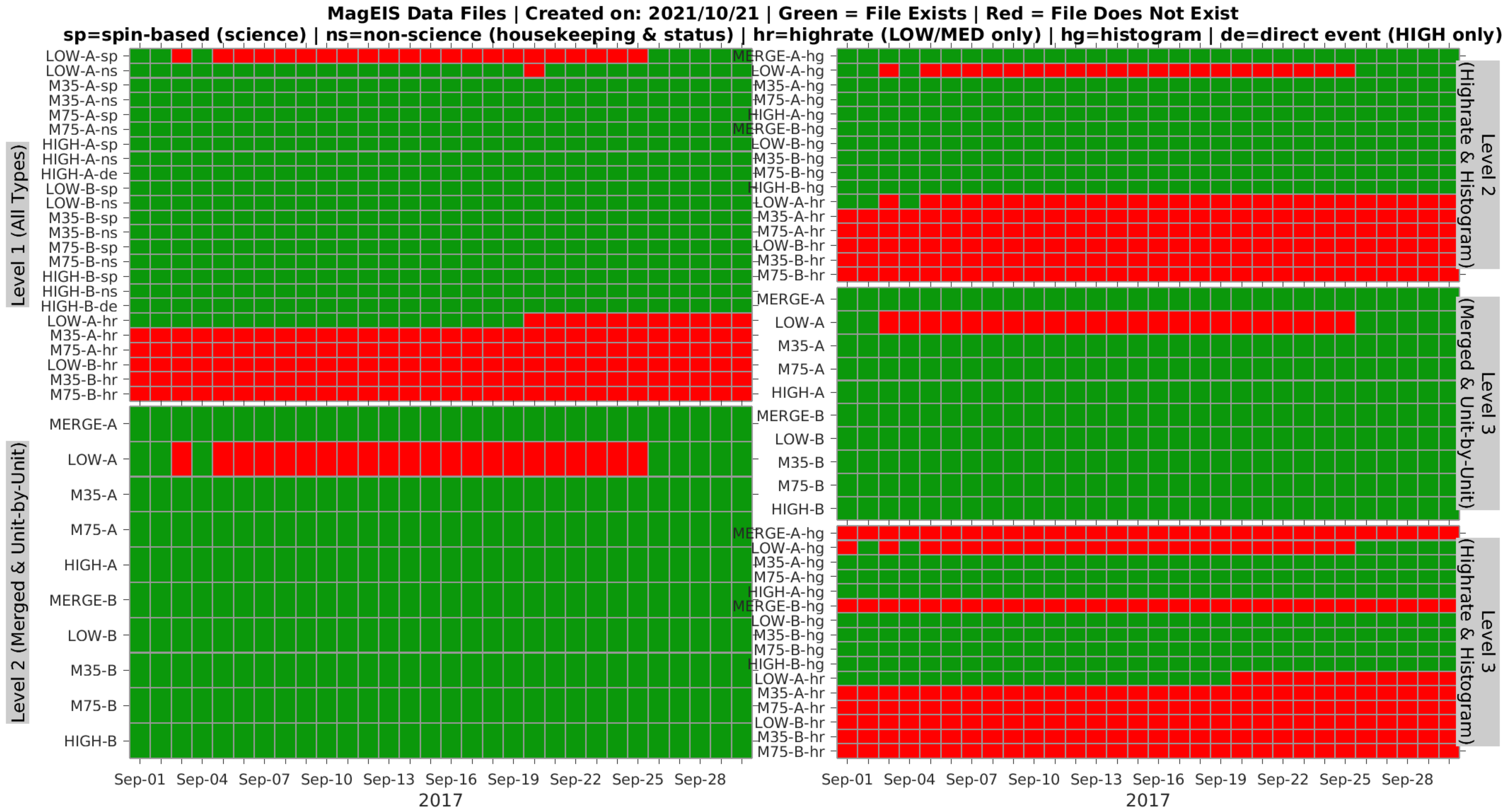
MagEIS Data Files | Created on: 2021/10/21 | Green = File Exists | Red = File Does Not Exist
sp=spin-based (science) | ns=non-science (housekeeping & status) | hr=highrate (LOW/MED only) | hg=histogram | de=direct event (HIGH only)
Level 1 (All Types)
LOW-A-sp
LOW-A-ns
M35-A-sp
M35-A-ns
M75-A-sp
M75-A-ns
HIGH-A-sp
HIGH-A-ns
HIGH-A-de
LOW-B-sp
LOW-B-ns
M35-B-sp
M35-B-ns
M75-B-sp
M75-B-ns
HIGH-B-sp
HIGH-B-ns
HIGH-B-de
LOW-A-hr
M35-A-hr
M75-A-hr
LOW-B-hr
M35-B-hr
M75-B-hr
Level 2 (Merged & Unit-by-Unit)
MERGE-A
LOW-A
M35-A
M75-A
HIGH-A
MERGE-B
LOW-B
M35-B
M75-B
HIGH-B
Level 2 (Highrate & Histogram)
MERGE-A-hg
LOW-A-hg
M35-A-hg
M75-A-hg
HIGH-A-hg
MERGE-B-hg
LOW-B-hg
M35-B-hg
M75-B-hg
HIGH-B-hg
LOW-A-hr
M35-A-hr
M75-A-hr
LOW-B-hr
M35-B-hr
M75-B-hr
Level 3 (Merged & Unit-by-Unit)
MERGE-A
LOW-A
M35-A
M75-A
HIGH-A
MERGE-B
LOW-B
M35-B
M75-B
HIGH-B
Level 3 (Highrate & Histogram)
MERGE-A-hg
LOW-A-hg
M35-A-hg
M75-A-hg
HIGH-A-hg
MERGE-B-hg
LOW-B-hg
M35-B-hg
M75-B-hg
HIGH-B-hg
LOW-A-hr
M35-A-hr
M75-A-hr
LOW-B-hr
M35-B-hr
M75-B-hr
Sep-01
Sep-04
Sep-07
Sep-10
Sep-13
Sep-16
Sep-19
Sep-22
Sep-25
Sep-28
2017

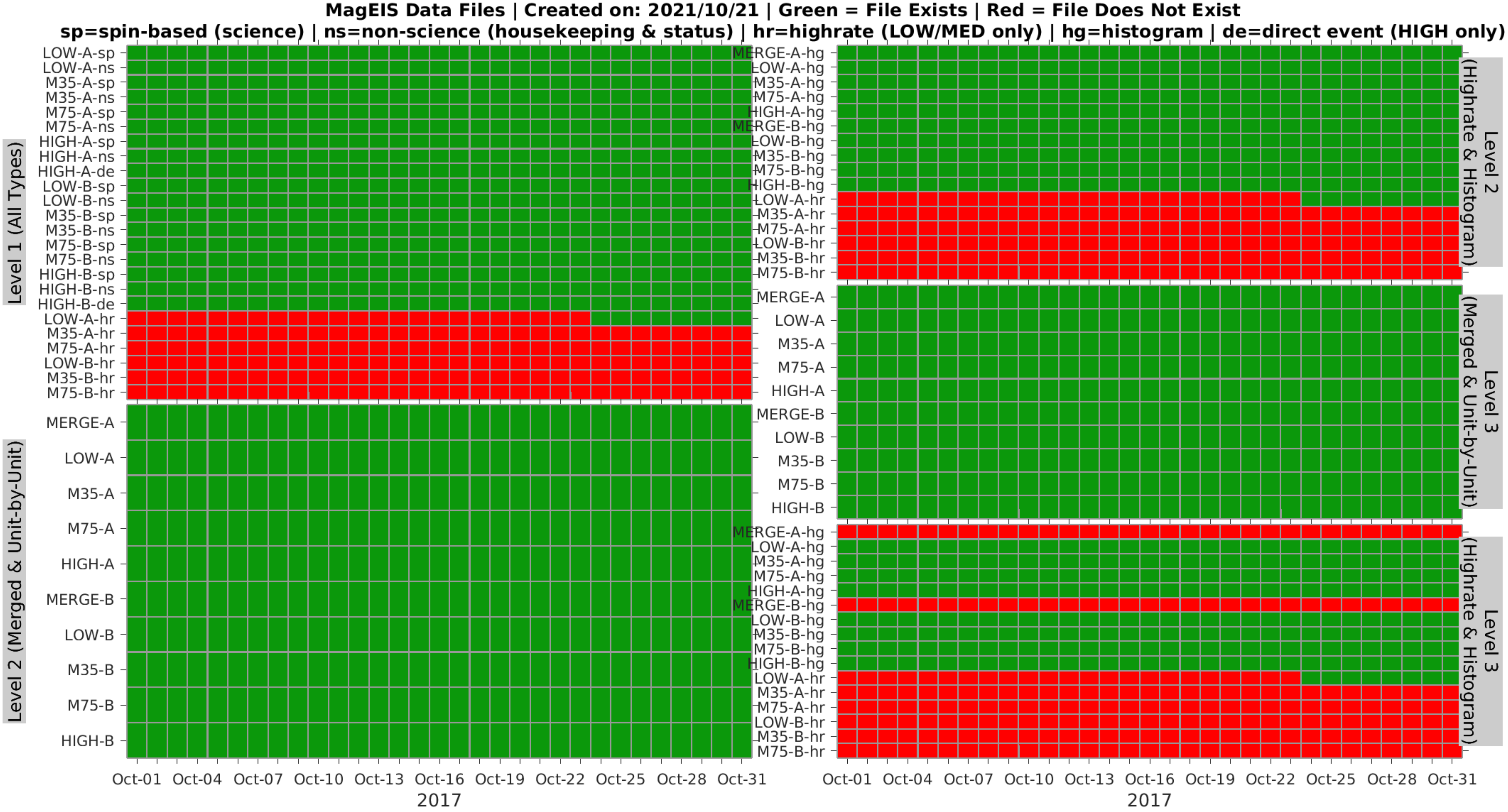
MagEIS Data Files | Created on: 2021/10/21 | Green = File Exists | Red = File Does Not Exist
sp=spin-based (science) | ns=non-science (housekeeping & status) | hr=highrate (LOW/MED only) | hg=histogram | de=direct event (HIGH only)
Level 1 (All Types)
LOW-A-sp
LOW-A-ns
M35-A-sp
M35-A-ns
M75-A-sp
M75-A-ns
HIGH-A-sp
HIGH-A-ns
HIGH-A-de
LOW-B-sp
LOW-B-ns
M35-B-sp
M35-B-ns
M75-B-sp
M75-B-ns
HIGH-B-sp
HIGH-B-ns
HIGH-B-de
LOW-A-hr
M35-A-hr
M75-A-hr
LOW-B-hr
M35-B-hr
M75-B-hr
Level 2 (Merged & Unit-by-Unit)
MERGE-A
LOW-A
M35-A
M75-A
HIGH-A
MERGE-B
LOW-B
M35-B
M75-B
HIGH-B
Level 2 (Highrate & Histogram)
MERGE-A-hg
LOW-A-hg
M35-A-hg
M75-A-hg
HIGH-A-hg
MERGE-B-hg
LOW-B-hg
M35-B-hg
M75-B-hg
HIGH-B-hg
LOW-A-hr
M35-A-hr
M75-A-hr
LOW-B-hr
M35-B-hr
M75-B-hr
Level 3 (Merged & Unit-by-Unit)
MERGE-A
LOW-A
M35-A
M75-A
HIGH-A
MERGE-B
LOW-B
M35-B
M75-B
HIGH-B
Level 3 (Highrate & Histogram)
MERGE-A-hg
LOW-A-hg
M35-A-hg
M75-A-hg
HIGH-A-hg
MERGE-B-hg
LOW-B-hg
M35-B-hg
M75-B-hg
HIGH-B-hg
LOW-A-hr
M35-A-hr
M75-A-hr
LOW-B-hr
M35-B-hr
M75-B-hr
Oct-01
Oct-04
Oct-07
Oct-10
Oct-13
Oct-16
Oct-19
Oct-22
Oct-25
Oct-28
Oct-31
2017

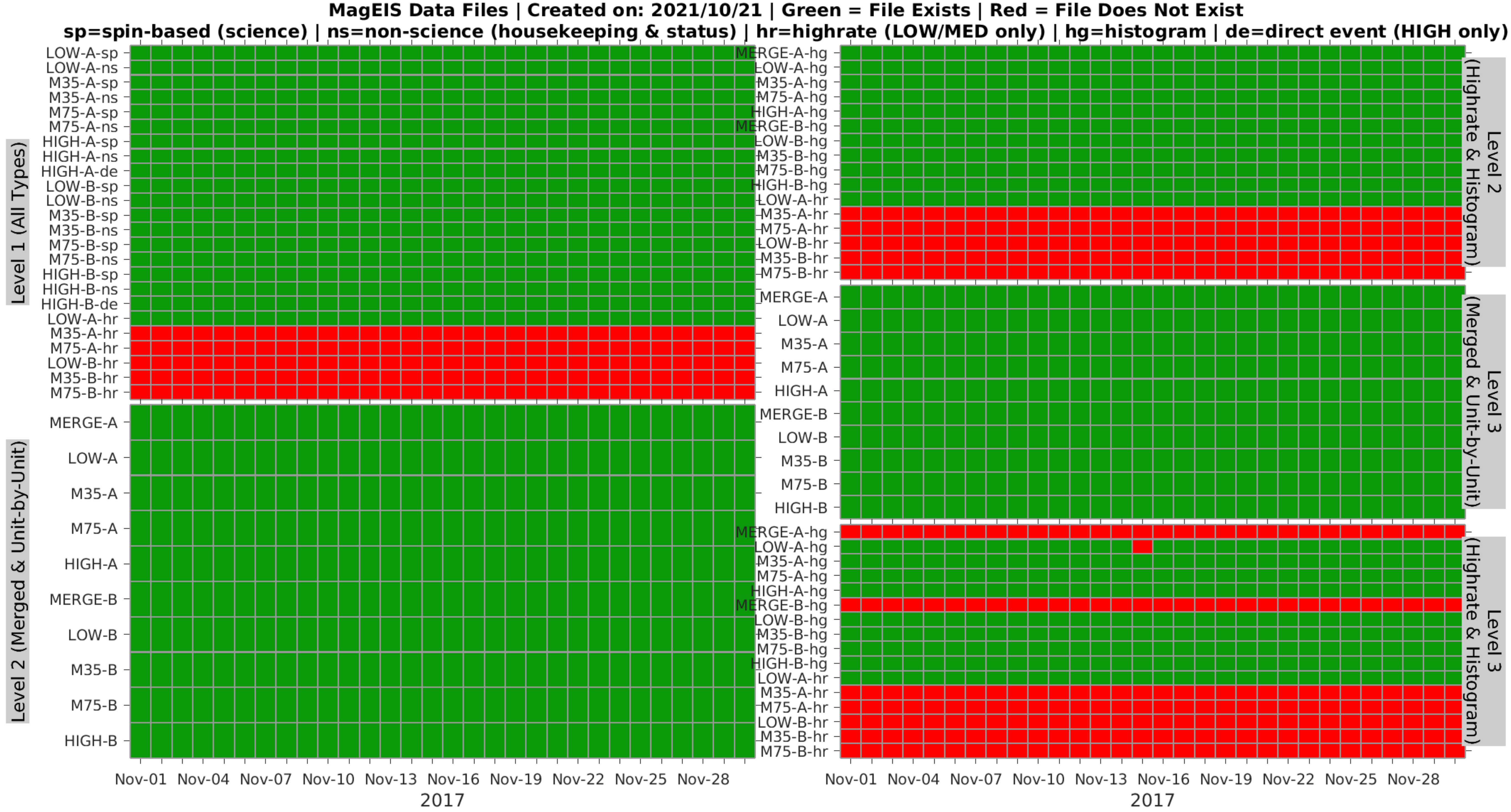
MagEIS Data Files | Created on: 2021/10/21 | Green = File Exists | Red = File Does Not Exist
sp=spin-based (science) | ns=non-science (housekeeping & status) | hr=highrate (LOW/MED only) | hg=histogram | de=direct event (HIGH only)
Level 1 (All Types)
Level 2 (Merged & Unit-by-Unit)
Level 2 (Highrate & Histogram)
Level 3 (Merged & Unit-by-Unit)
Level 3 (Highrate & Histogram)
LOW-A-sp
LOW-A-ns
M35-A-sp
M35-A-ns
M75-A-sp
M75-A-ns
HIGH-A-sp
HIGH-A-ns
HIGH-A-de
LOW-B-sp
LOW-B-ns
M35-B-sp
M35-B-ns
M75-B-sp
M75-B-ns
HIGH-B-sp
HIGH-B-ns
HIGH-B-de
LOW-A-hr
M35-A-hr
M75-A-hr
LOW-B-hr
M35-B-hr
M75-B-hr
MERGE-A
LOW-A
M35-A
M75-A
HIGH-A
MERGE-B
LOW-B
M35-B
M75-B
HIGH-B
MERGE-A-hg
LOW-A-hg
M35-A-hg
M75-A-hg
HIGH-A-hg
MERGE-B-hg
LOW-B-hg
M35-B-hg
M75-B-hg
HIGH-B-hg
Nov-01
Nov-04
Nov-07
Nov-10
Nov-13
Nov-16
Nov-19
Nov-22
Nov-25
Nov-28
2017

MagEIS Data Files | Created on: 2021/10/21 | Green = File Exists | Red = File Does Not Exist
sp=spin-based (science) | ns=non-science (housekeeping & status) | hr=highrate (LOW/MED only) | hg=histogram | de=direct event (HIGH only)

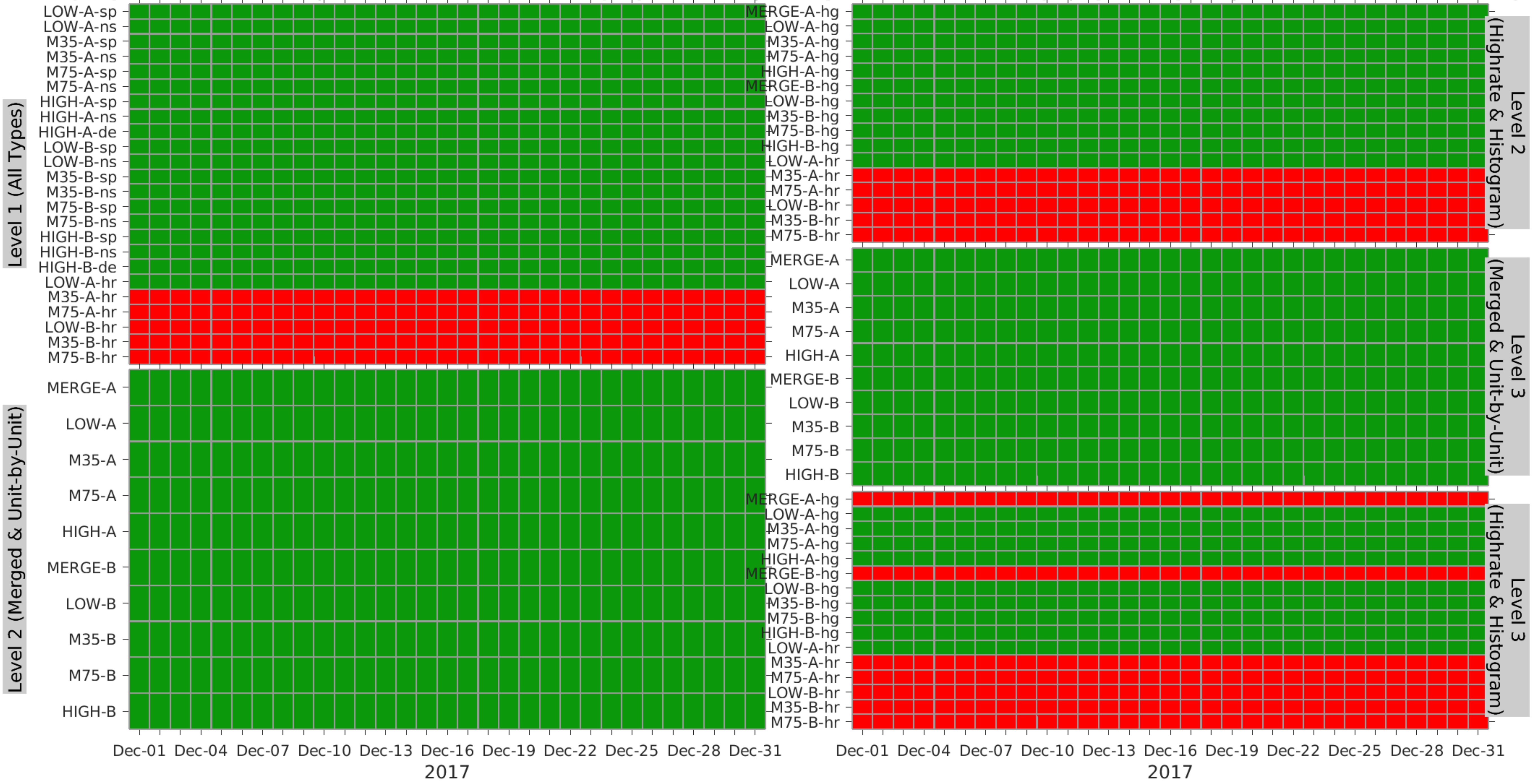

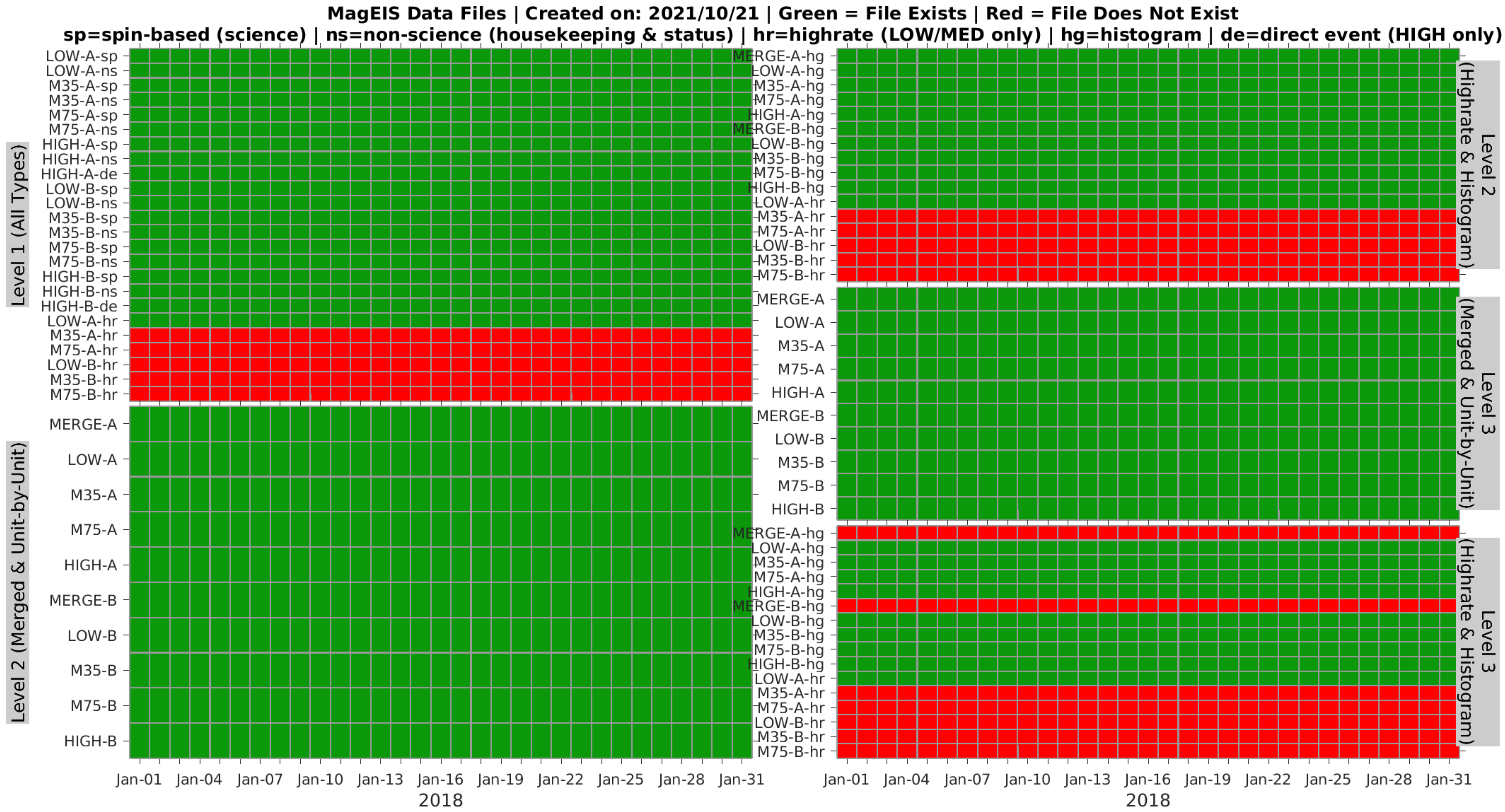
MagEIS Data Files | Created on: 2021/10/21 | Green = File Exists | Red = File Does Not Exist
sp=spin-based (science) | ns=non-science (housekeeping & status) | hr=highrate (LOW/MED only) | hg=histogram | de=direct event (HIGH only)
Level 1 (All Types)
LOW-A-sp
LOW-A-ns
M35-A-sp
M35-A-ns
M75-A-sp
M75-A-ns
HIGH-A-sp
HIGH-A-ns
HIGH-A-de
LOW-B-sp
LOW-B-ns
M35-B-sp
M35-B-ns
M75-B-sp
M75-B-ns
HIGH-B-sp
HIGH-B-ns
HIGH-B-de
LOW-A-hr
M35-A-hr
M75-A-hr
LOW-B-hr
M35-B-hr
M75-B-hr
Level 2 (Merged & Unit-by-Unit)
MERGE-A
LOW-A
M35-A
M75-A
HIGH-A
MERGE-B
LOW-B
M35-B
M75-B
HIGH-B
Level 2 (Highrate & Histogram)
MERGE-A-hg
LOW-A-hg
M35-A-hg
M75-A-hg
HIGH-A-hg
MERGE-B-hg
LOW-B-hg
M35-B-hg
M75-B-hg
HIGH-B-hg
LOW-A-hr
M35-A-hr
M75-A-hr
LOW-B-hr
M35-B-hr
M75-B-hr
Level 3 (Merged & Unit-by-Unit)
MERGE-A
LOW-A
M35-A
M75-A
HIGH-A
MERGE-B
LOW-B
M35-B
M75-B
HIGH-B
Level 3 (Highrate & Histogram)
MERGE-A-hg
LOW-A-hg
M35-A-hg
M75-A-hg
HIGH-A-hg
MERGE-B-hg
LOW-B-hg
M35-B-hg
M75-B-hg
HIGH-B-hg
LOW-A-hr
M35-A-hr
M75-A-hr
LOW-B-hr
M35-B-hr
M75-B-hr
Jan-01
Jan-04
Jan-07
Jan-10
Jan-13
Jan-16
Jan-19
Jan-22
Jan-25
Jan-28
Jan-31
2018

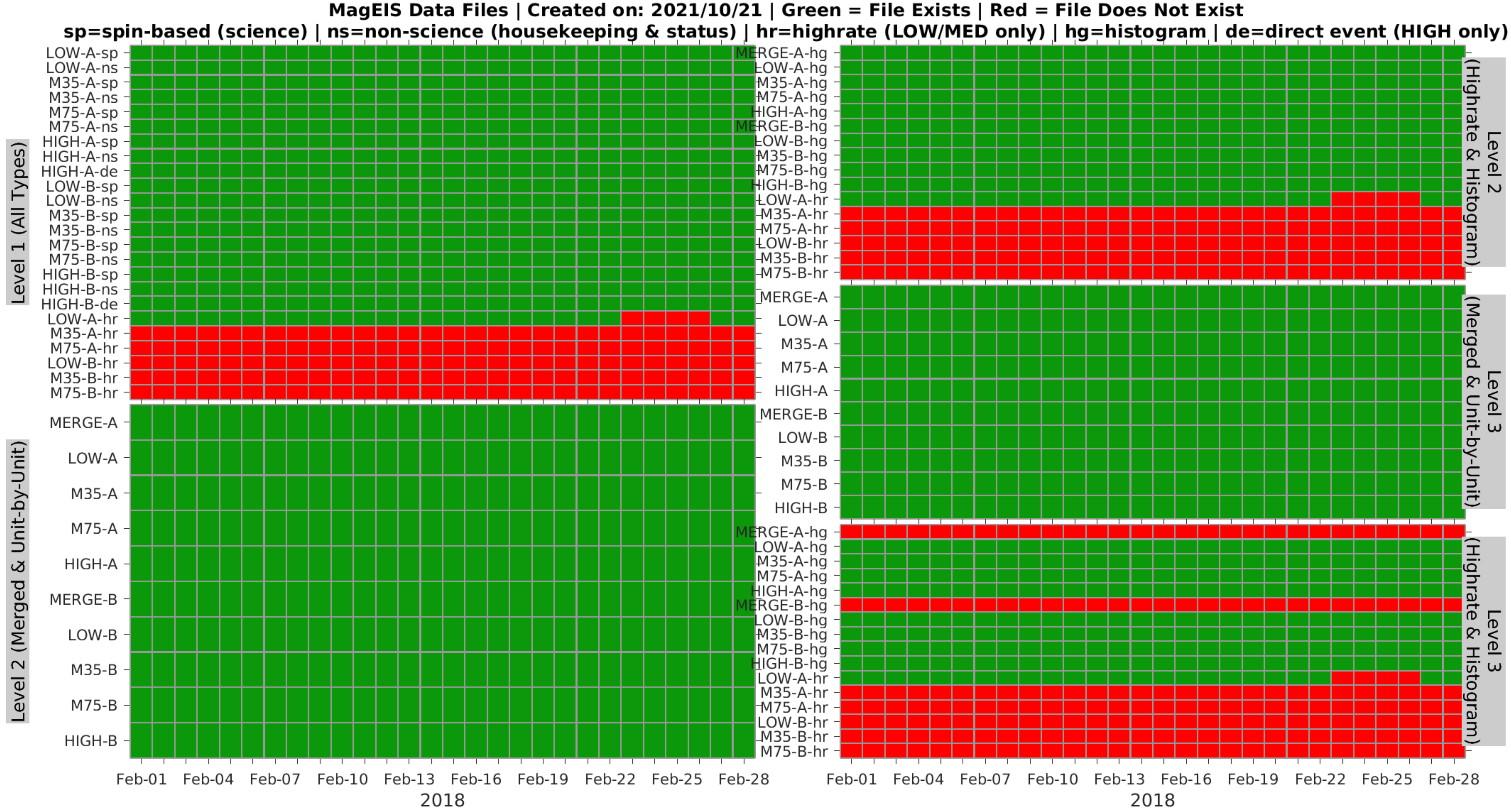
MagEIS Data Files | Created on: 2021/10/21 | Green = File Exists | Red = File Does Not Exist
sp=spin-based (science) | ns=non-science (housekeeping & status) | hr=highrate (LOW/MED only) | hg=histogram | de=direct event (HIGH only)
Level 1 (All Types)
LOW-A-sp
LOW-A-ns
M35-A-sp
M35-A-ns
M75-A-sp
M75-A-ns
HIGH-A-sp
HIGH-A-ns
HIGH-A-de
LOW-B-sp
LOW-B-ns
M35-B-sp
M35-B-ns
M75-B-sp
M75-B-ns
HIGH-B-sp
HIGH-B-ns
HIGH-B-de
LOW-A-hr
M35-A-hr
M75-A-hr
LOW-B-hr
M35-B-hr
M75-B-hr
Level 2 (Merged & Unit-by-Unit)
MERGE-A
LOW-A
M35-A
M75-A
HIGH-A
MERGE-B
LOW-B
M35-B
M75-B
HIGH-B
Level 2 (Highrate & Histogram)
MERGE-A-hg
LOW-A-hg
M35-A-hg
M75-A-hg
HIGH-A-hg
MERGE-B-hg
LOW-B-hg
M35-B-hg
M75-B-hg
HIGH-B-hg
LOW-A-hr
M35-A-hr
M75-A-hr
LOW-B-hr
M35-B-hr
M75-B-hr
Level 3 (Merged & Unit-by-Unit)
MERGE-A
LOW-A
M35-A
M75-A
HIGH-A
MERGE-B
LOW-B
M35-B
M75-B
HIGH-B
Level 3 (Highrate & Histogram)
MERGE-A-hg
LOW-A-hg
M35-A-hg
M75-A-hg
HIGH-A-hg
MERGE-B-hg
LOW-B-hg
M35-B-hg
M75-B-hg
HIGH-B-hg
LOW-A-hr
M35-A-hr
M75-A-hr
LOW-B-hr
M35-B-hr
M75-B-hr
Feb-01 Feb-04 Feb-07 Feb-10 Feb-13 Feb-16 Feb-19 Feb-22 Feb-25 Feb-28
2018

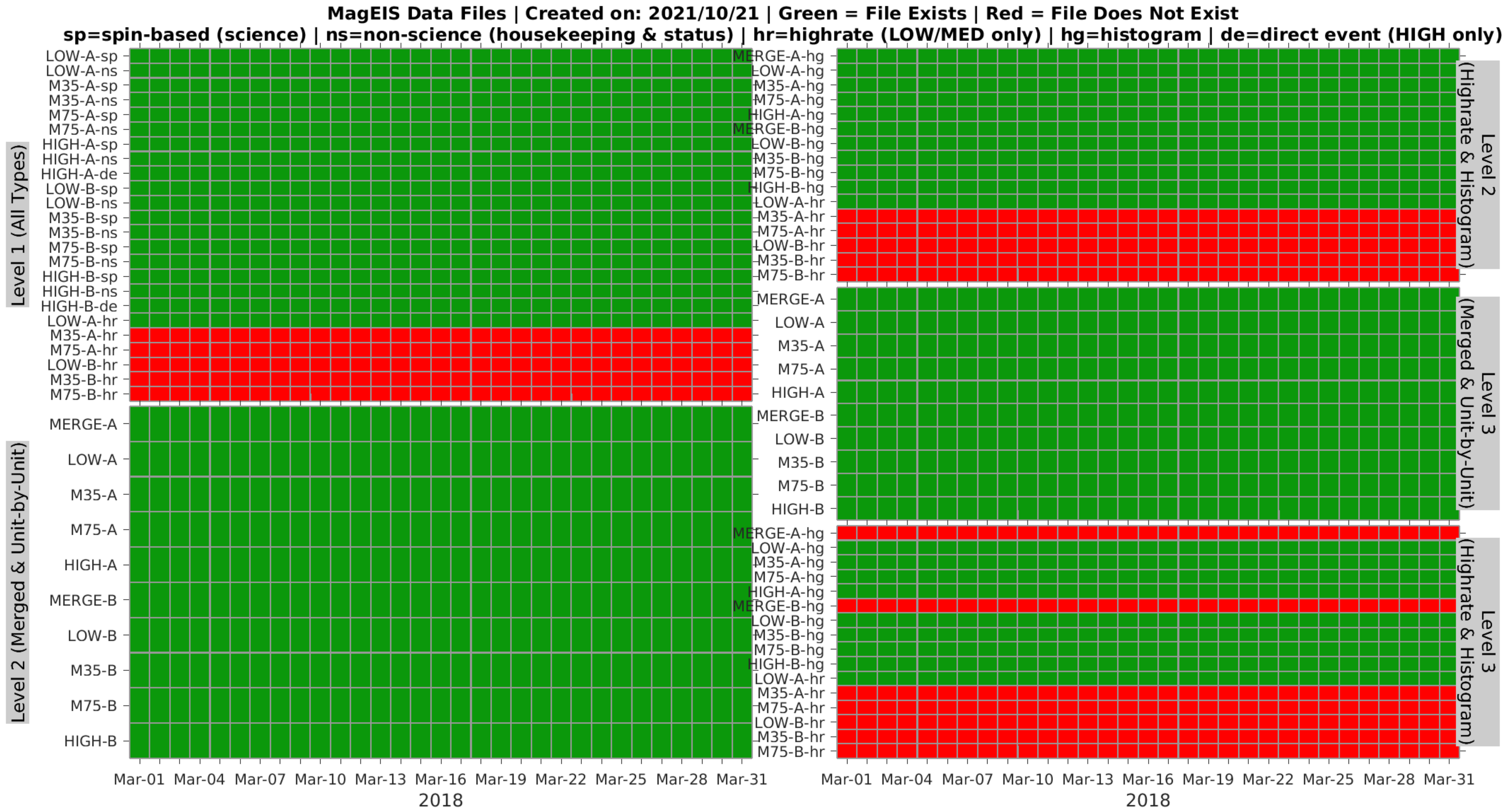

MagEIS Data Files | Created on: 2021/10/21 | Green = File Exists | Red = File Does Not Exist
sp=spin-based (science) | ns=non-science (housekeeping & status) | hr=highrate (LOW/MED only) | hg=histogram | de=direct event (HIGH only)
Level 1 (All Types)
LOW-A-sp
LOW-A-ns
M35-A-sp
M35-A-ns
M75-A-sp
M75-A-ns
HIGH-A-sp
HIGH-A-ns
HIGH-A-de
LOW-B-sp
LOW-B-ns
M35-B-sp
M35-B-ns
M75-B-sp
M75-B-ns
HIGH-B-sp
HIGH-B-ns
HIGH-B-de
LOW-A-hr
M35-A-hr
M75-A-hr
LOW-B-hr
M35-B-hr
M75-B-hr
Level 2 (Merged & Unit-by-Unit)
MERGE-A
LOW-A
M35-A
M75-A
HIGH-A
MERGE-B
LOW-B
M35-B
M75-B
HIGH-B
Mar-01
Mar-04
Mar-07
Mar-10
Mar-13
Mar-16
Mar-19
Mar-22
Mar-25
Mar-28
Mar-31
2018
Level 2 (Highrate & Histogram)
MERGE-A-hg
LOW-A-hg
M35-A-hg
M75-A-hg
HIGH-A-hg
MERGE-B-hg
LOW-B-hg
M35-B-hg
M75-B-hg
HIGH-B-hg
LOW-A-hr
M35-A-hr
M75-A-hr
LOW-B-hr
M35-B-hr
M75-B-hr
Level 3 (Merged & Unit-by-Unit)
MERGE-A
LOW-A
M35-A
M75-A
HIGH-A
MERGE-B
LOW-B
M35-B
M75-B
HIGH-B
Level 3 (Highrate & Histogram)
MERGE-A-hg
LOW-A-hg
M35-A-hg
M75-A-hg
HIGH-A-hg
MERGE-B-hg
LOW-B-hg
M35-B-hg
M75-B-hg
HIGH-B-hg
LOW-A-hr
M35-A-hr
M75-A-hr
LOW-B-hr
M35-B-hr
M75-B-hr
Mar-01
Mar-04
Mar-07
Mar-10
Mar-13
Mar-16
Mar-19
Mar-22
Mar-25
Mar-28
Mar-31
2018

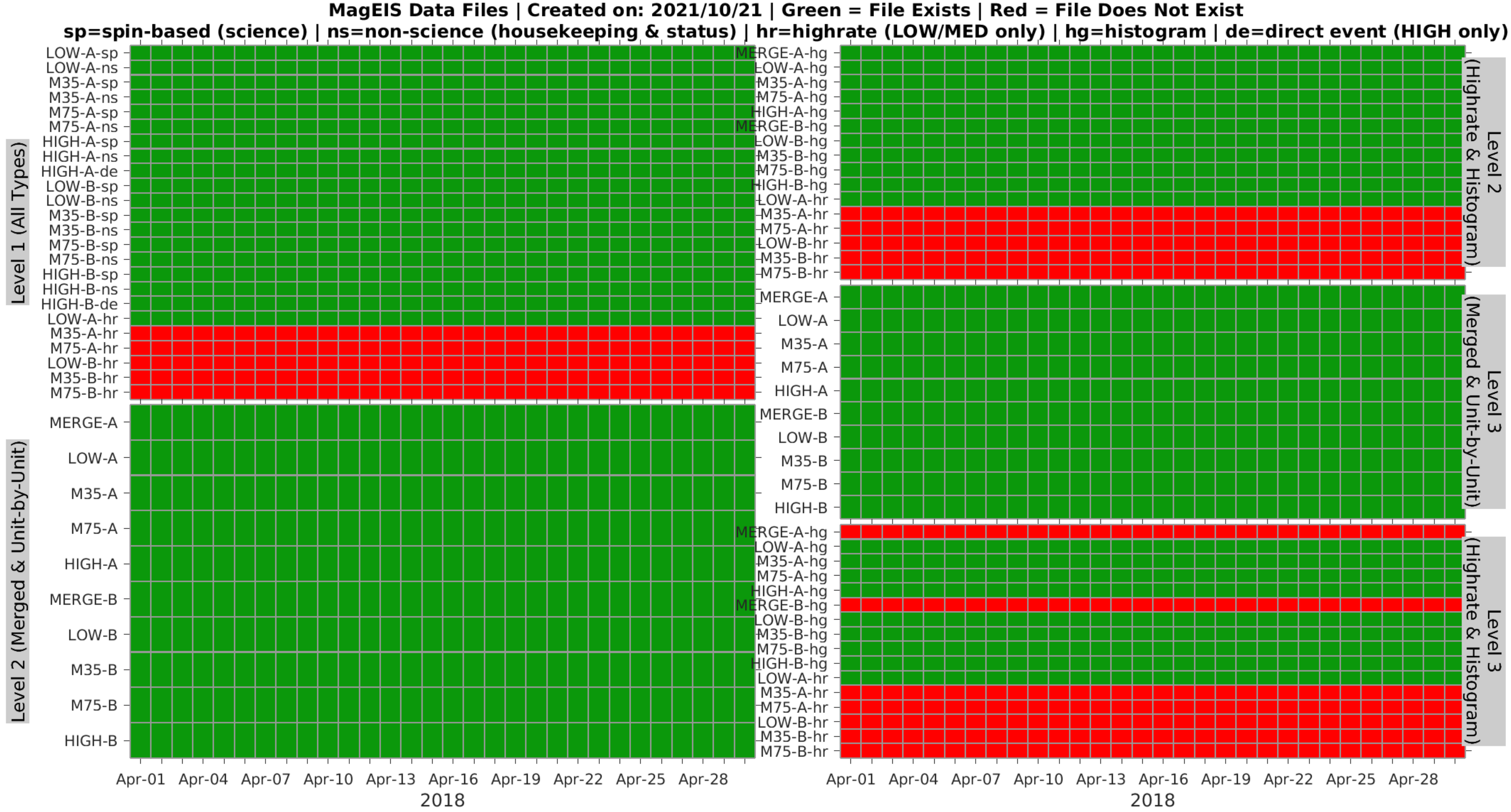
MagEIS Data Files | Created on: 2021/10/21 | Green = File Exists | Red = File Does Not Exist
sp=spin-based (science) | ns=non-science (housekeeping & status) | hr=highrate (LOW/MED only) | hg=histogram | de=direct event (HIGH only)
Level 1 (All Types)
LOW-A-sp
LOW-A-ns
M35-A-sp
M35-A-ns
M75-A-sp
M75-A-ns
HIGH-A-sp
HIGH-A-ns
HIGH-A-de
LOW-B-sp
LOW-B-ns
M35-B-sp
M35-B-ns
M75-B-sp
M75-B-ns
HIGH-B-sp
HIGH-B-ns
HIGH-B-de
LOW-A-hr
M35-A-hr
M75-A-hr
LOW-B-hr
M35-B-hr
M75-B-hr
Level 2 (Merged & Unit-by-Unit)
MERGE-A
LOW-A
M35-A
M75-A
HIGH-A
MERGE-B
LOW-B
M35-B
M75-B
HIGH-B
Level 2 (Highrate & Histogram)
MERGE-A-hg
LOW-A-hg
M35-A-hg
M75-A-hg
HIGH-A-hg
MERGE-B-hg
LOW-B-hg
M35-B-hg
M75-B-hg
HIGH-B-hg
LOW-A-hr
M35-A-hr
M75-A-hr
LOW-B-hr
M35-B-hr
M75-B-hr
Level 3 (Merged & Unit-by-Unit)
Level 3 (Highrate & Histogram)
Apr-01
Apr-04
Apr-07
Apr-10
Apr-13
Apr-16
Apr-19
Apr-22
Apr-25
Apr-28
2018

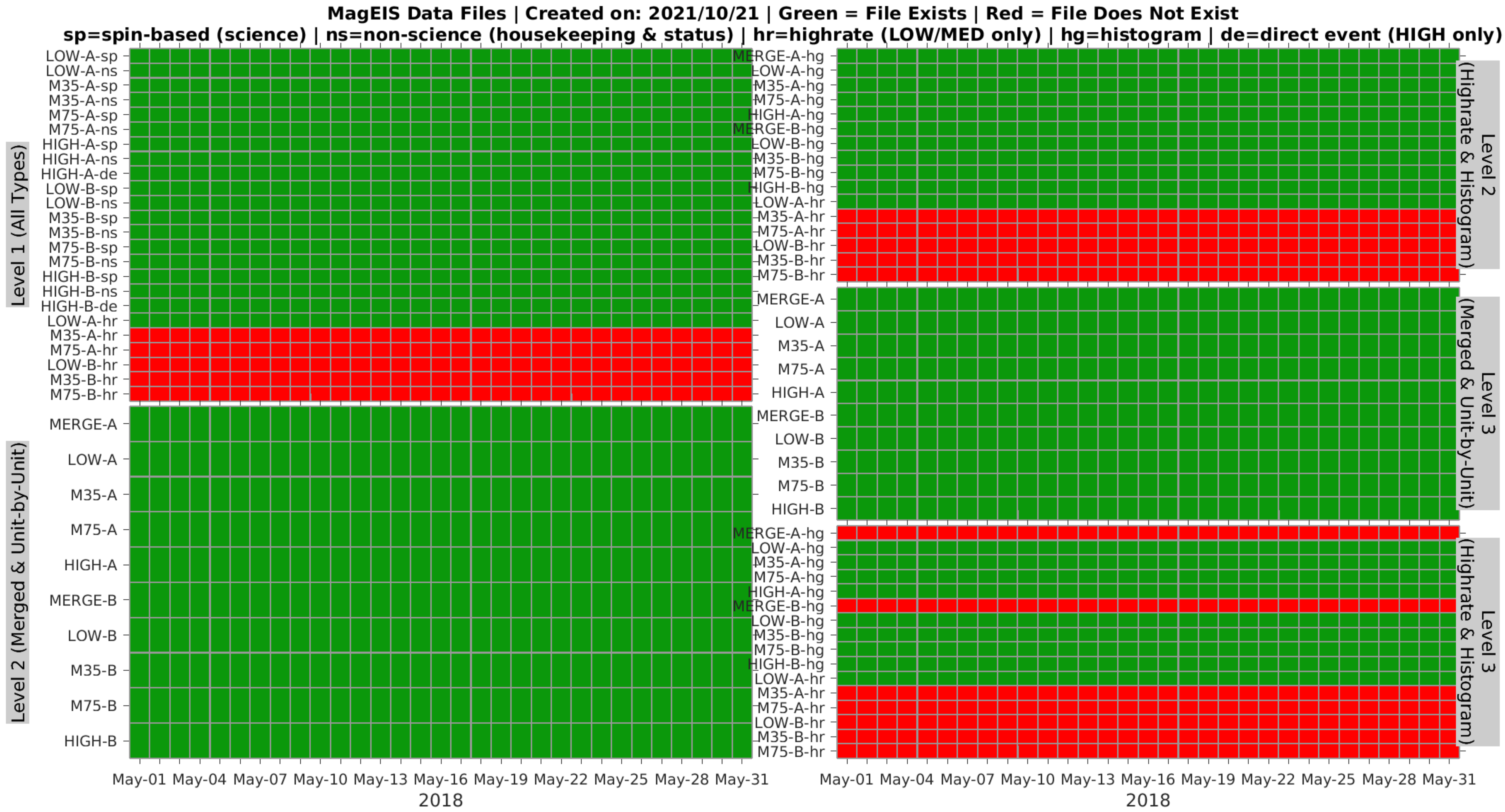

MagEIS Data Files | Created on: 2021/10/21 | Green = File Exists | Red = File Does Not Exist
sp=spin-based (science) | ns=non-science (housekeeping & status) | hr=highrate (LOW/MED only) | hg=histogram | de=direct event (HIGH only)
Level 1 (All Types)
LOW-A-sp
LOW-A-ns
M35-A-sp
M35-A-ns
M75-A-sp
M75-A-ns
HIGH-A-sp
HIGH-A-ns
HIGH-A-de
LOW-B-sp
LOW-B-ns
M35-B-sp
M35-B-ns
M75-B-sp
M75-B-ns
HIGH-B-sp
HIGH-B-ns
HIGH-B-de
LOW-A-hr
M35-A-hr
M75-A-hr
LOW-B-hr
M35-B-hr
M75-B-hr
Level 2 (Merged & Unit-by-Unit)
MERGE-A
LOW-A
M35-A
M75-A
HIGH-A
MERGE-B
LOW-B
M35-B
M75-B
HIGH-B
Level 2 (Highrate & Histogram)
MERGE-A-hg
LOW-A-hg
M35-A-hg
M75-A-hg
HIGH-A-hg
MERGE-B-hg
LOW-B-hg
M35-B-hg
M75-B-hg
HIGH-B-hg
LOW-A-hr
M35-A-hr
M75-A-hr
LOW-B-hr
M35-B-hr
M75-B-hr
Level 3 (Merged & Unit-by-Unit)
MERGE-A
LOW-A
M35-A
M75-A
HIGH-A
MERGE-B
LOW-B
M35-B
M75-B
HIGH-B
Level 3 (Highrate & Histogram)
MERGE-A-hg
LOW-A-hg
M35-A-hg
M75-A-hg
HIGH-A-hg
MERGE-B-hg
LOW-B-hg
M35-B-hg
M75-B-hg
HIGH-B-hg
LOW-A-hr
M35-A-hr
M75-A-hr
LOW-B-hr
M35-B-hr
M75-B-hr
May-01 May-04 May-07 May-10 May-13 May-16 May-19 May-22 May-25 May-28 May-31
2018

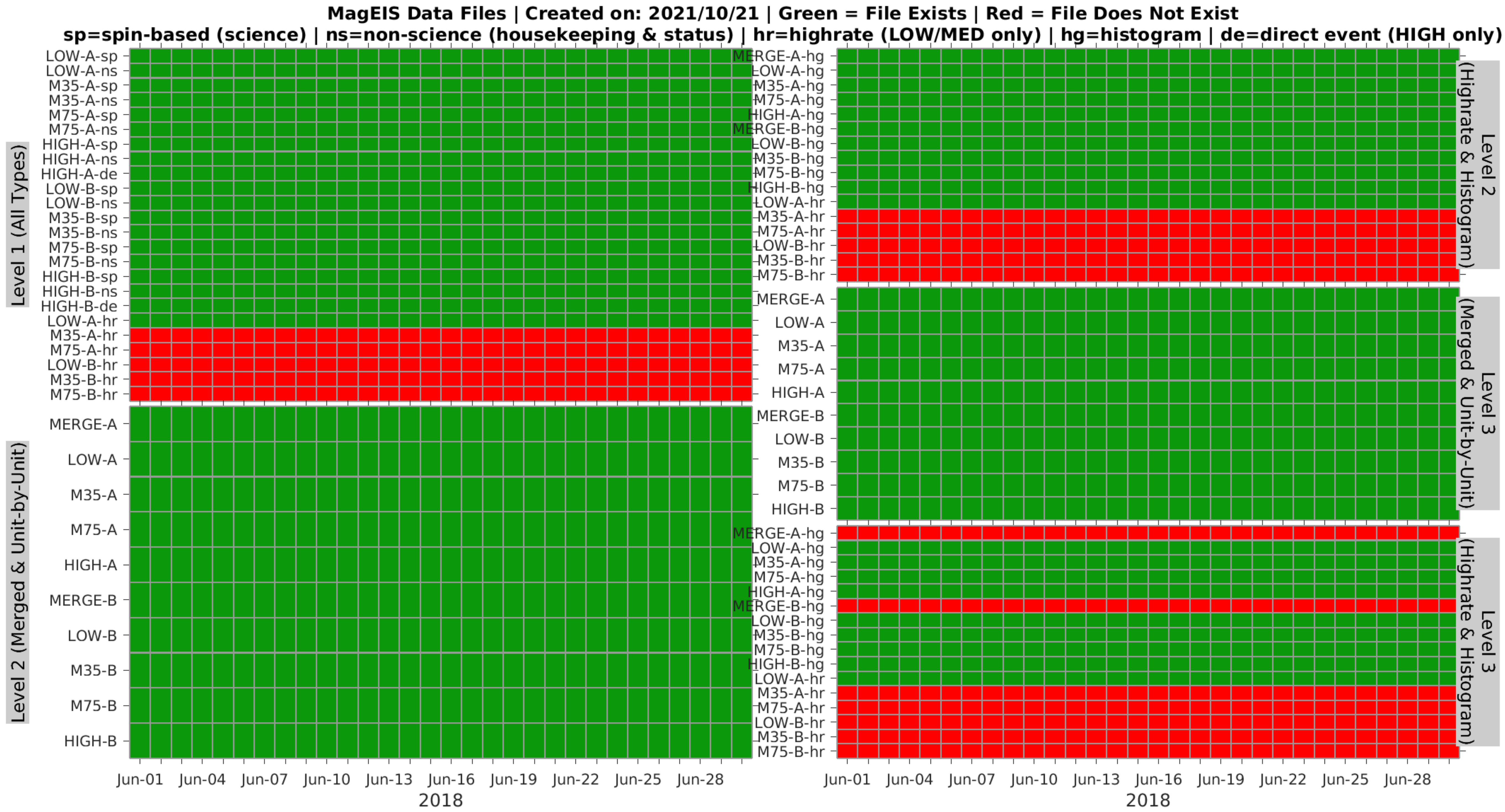
MagEIS Data Files | Created on: 2021/10/21 | Green = File Exists | Red = File Does Not Exist
sp=spin-based (science) | ns=non-science (housekeeping & status) | hr=highrate (LOW/MED only) | hg=histogram | de=direct event (HIGH only)
Level 1 (All Types)
LOW-A-sp
LOW-A-ns
M35-A-sp
M35-A-ns
M75-A-sp
M75-A-ns
HIGH-A-sp
HIGH-A-ns
HIGH-A-de
LOW-B-sp
LOW-B-ns
M35-B-sp
M35-B-ns
M75-B-sp
M75-B-ns
HIGH-B-sp
HIGH-B-ns
HIGH-B-de
LOW-A-hr
M35-A-hr
M75-A-hr
LOW-B-hr
M35-B-hr
M75-B-hr
Level 2 (Merged & Unit-by-Unit)
MERGE-A
LOW-A
M35-A
M75-A
HIGH-A
MERGE-B
LOW-B
M35-B
M75-B
HIGH-B
Level 2 (Highrate & Histogram)
MERGE-A-hg
LOW-A-hg
M35-A-hg
M75-A-hg
HIGH-A-hg
MERGE-B-hg
LOW-B-hg
M35-B-hg
M75-B-hg
HIGH-B-hg
LOW-A-hr
M35-A-hr
M75-A-hr
LOW-B-hr
M35-B-hr
M75-B-hr
Level 3 (Merged & Unit-by-Unit)
MERGE-A
LOW-A
M35-A
M75-A
HIGH-A
MERGE-B
LOW-B
M35-B
M75-B
HIGH-B
Level 3 (Highrate & Histogram)
MERGE-A-hg
LOW-A-hg
M35-A-hg
M75-A-hg
HIGH-A-hg
MERGE-B-hg
LOW-B-hg
M35-B-hg
M75-B-hg
HIGH-B-hg
LOW-A-hr
M35-A-hr
M75-A-hr
LOW-B-hr
M35-B-hr
M75-B-hr
Jun-01
Jun-04
Jun-07
Jun-10
Jun-13
Jun-16
Jun-19
Jun-22
Jun-25
Jun-28
2018

MagEIS Data Files | Created on: 2021/10/21 | Green = File Exists | Red = File Does Not Exist
sp=spin-based (science) | ns=non-science (housekeeping & status) | hr=highrate (LOW/MED only) | hg=histogram | de=direct event (HIGH only)

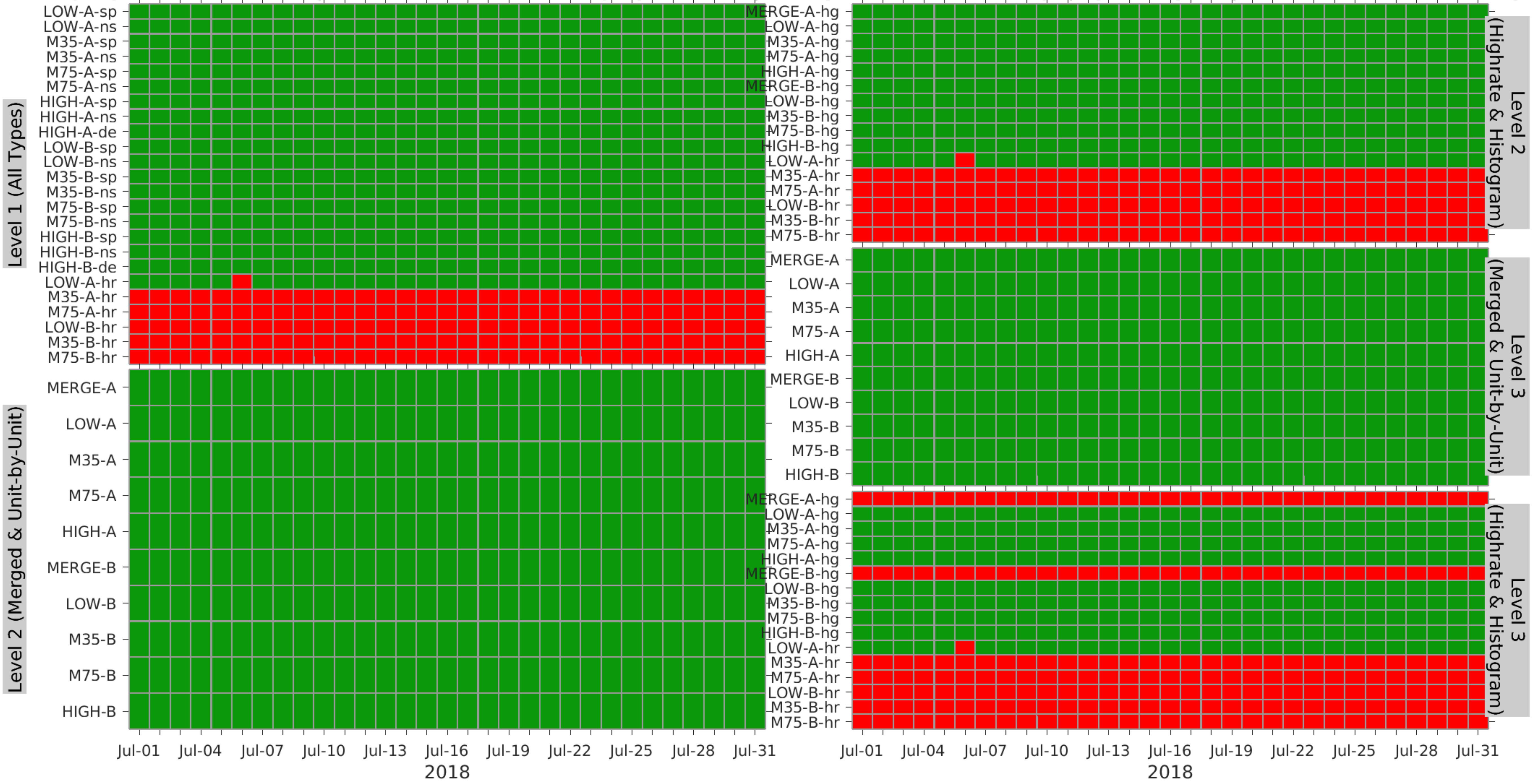

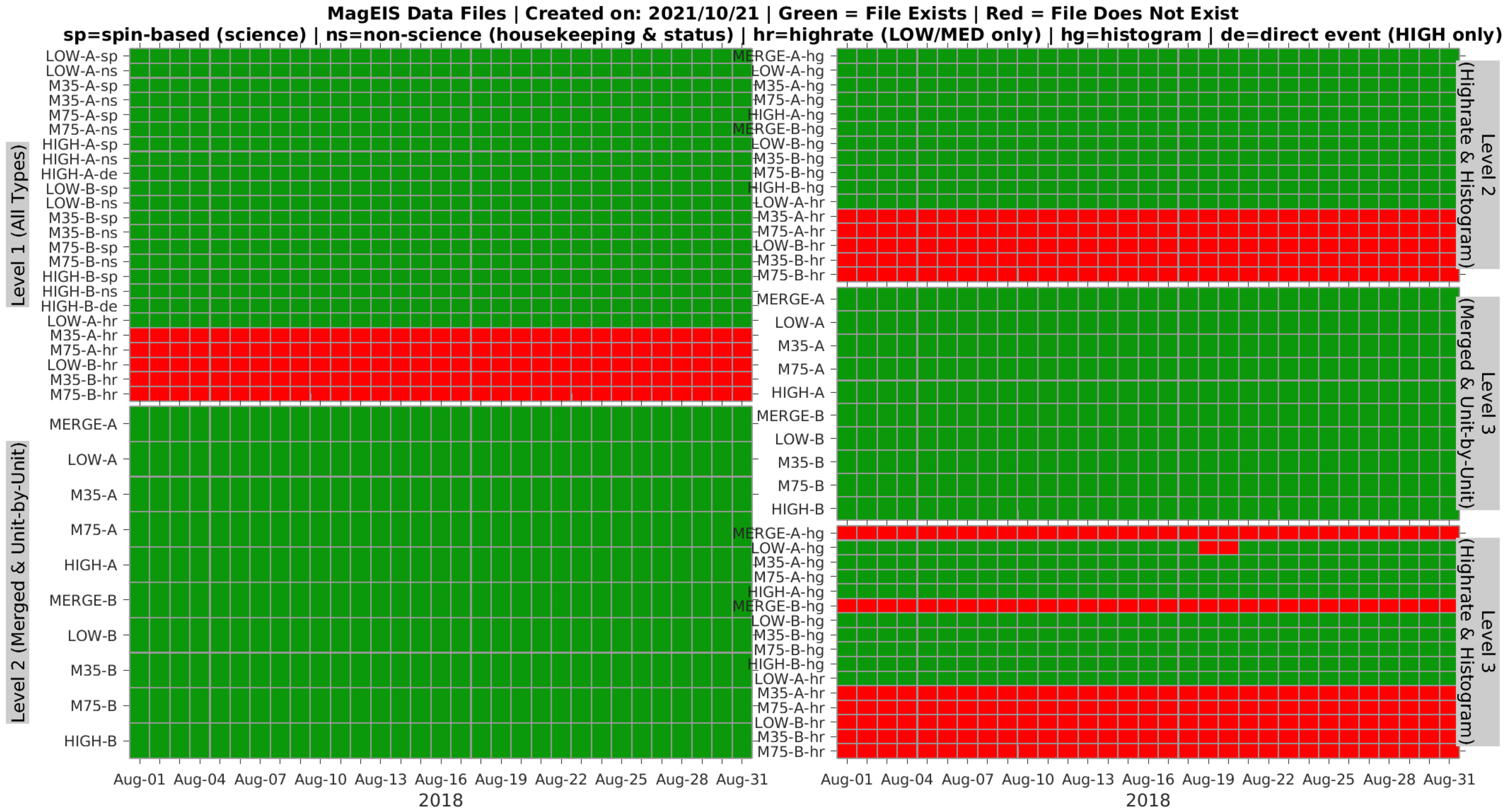
MagEIS Data Files | Created on: 2021/10/21 | Green = File Exists | Red = File Does Not Exist
sp=spin-based (science) | ns=non-science (housekeeping & status) | hr=highrate (LOW/MED only) | hg=histogram | de=direct event (HIGH only)
Level 1 (All Types)
LOW-A-sp
LOW-A-ns
M35-A-sp
M35-A-ns
M75-A-sp
M75-A-ns
HIGH-A-sp
HIGH-A-ns
HIGH-A-de
LOW-B-sp
LOW-B-ns
M35-B-sp
M35-B-ns
M75-B-sp
M75-B-ns
HIGH-B-sp
HIGH-B-ns
HIGH-B-de
LOW-A-hr
M35-A-hr
M75-A-hr
LOW-B-hr
M35-B-hr
M75-B-hr
Level 2 (Merged & Unit-by-Unit)
MERGE-A
LOW-A
M35-A
M75-A
HIGH-A
MERGE-B
LOW-B
M35-B
M75-B
HIGH-B
Level 2 (Highrate & Histogram)
MERGE-A-hg
LOW-A-hg
M35-A-hg
M75-A-hg
HIGH-A-hg
MERGE-B-hg
LOW-B-hg
M35-B-hg
M75-B-hg
HIGH-B-hg
LOW-A-hr
M35-A-hr
M75-A-hr
LOW-B-hr
M35-B-hr
M75-B-hr
Level 3 (Merged & Unit-by-Unit)
MERGE-A
LOW-A
M35-A
M75-A
HIGH-A
MERGE-B
LOW-B
M35-B
M75-B
HIGH-B
Level 3 (Highrate & Histogram)
MERGE-A-hg
LOW-A-hg
M35-A-hg
M75-A-hg
HIGH-A-hg
MERGE-B-hg
LOW-B-hg
M35-B-hg
M75-B-hg
HIGH-B-hg
LOW-A-hr
M35-A-hr
M75-A-hr
LOW-B-hr
M35-B-hr
M75-B-hr
Aug-01 Aug-04 Aug-07 Aug-10 Aug-13 Aug-16 Aug-19 Aug-22 Aug-25 Aug-28 Aug-31
2018
Aug-01 Aug-04 Aug-07 Aug-10 Aug-13 Aug-16 Aug-19 Aug-22 Aug-25 Aug-28 Aug-31
2018

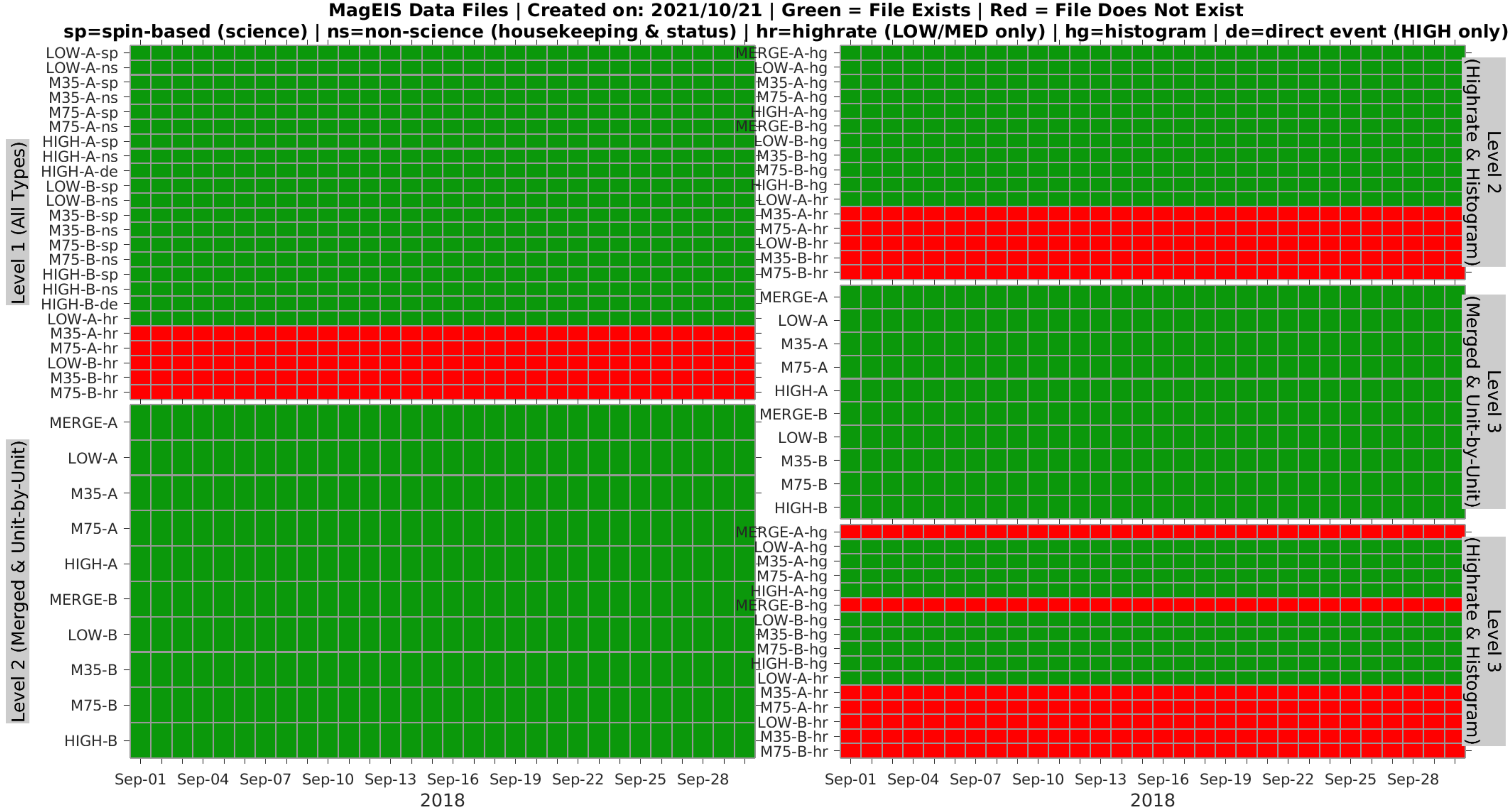
MagEIS Data Files | Created on: 2021/10/21 | Green = File Exists | Red = File Does Not Exist
sp=spin-based (science) | ns=non-science (housekeeping & status) | hr=highrate (LOW/MED only) | hg=histogram | de=direct event (HIGH only)
Level 1 (All Types)
LOW-A-sp
LOW-A-ns
M35-A-sp
M35-A-ns
M75-A-sp
M75-A-ns
HIGH-A-sp
HIGH-A-ns
HIGH-A-de
LOW-B-sp
LOW-B-ns
M35-B-sp
M35-B-ns
M75-B-sp
M75-B-ns
HIGH-B-sp
HIGH-B-ns
HIGH-B-de
LOW-A-hr
M35-A-hr
M75-A-hr
LOW-B-hr
M35-B-hr
M75-B-hr
Level 2 (Merged & Unit-by-Unit)
MERGE-A
LOW-A
M35-A
M75-A
HIGH-A
MERGE-B
LOW-B
M35-B
M75-B
HIGH-B
Level 2 (Highrate & Histogram)
MERGE-A-hg
LOW-A-hg
M35-A-hg
M75-A-hg
HIGH-A-hg
MERGE-B-hg
LOW-B-hg
M35-B-hg
M75-B-hg
HIGH-B-hg
LOW-A-hr
M35-A-hr
M75-A-hr
LOW-B-hr
M35-B-hr
M75-B-hr
Level 3 (Merged & Unit-by-Unit)
MERGE-A
LOW-A
M35-A
M75-A
HIGH-A
MERGE-B
LOW-B
M35-B
M75-B
HIGH-B
Level 3 (Highrate & Histogram)
MERGE-A-hg
LOW-A-hg
M35-A-hg
M75-A-hg
HIGH-A-hg
MERGE-B-hg
LOW-B-hg
M35-B-hg
M75-B-hg
HIGH-B-hg
LOW-A-hr
M35-A-hr
M75-A-hr
LOW-B-hr
M35-B-hr
M75-B-hr
Sep-01 Sep-04 Sep-07 Sep-10 Sep-13 Sep-16 Sep-19 Sep-22 Sep-25 Sep-28
2018
Sep-01 Sep-04 Sep-07 Sep-10 Sep-13 Sep-16 Sep-19 Sep-22 Sep-25 Sep-28
2018

MagEIS Data Files | Created on: 2021/10/21 | Green = File Exists | Red = File Does Not Exist
sp=spin-based (science) | ns=non-science (housekeeping & status) | hr=highrate (LOW/MED only) | hg=histogram | de=direct event (HIGH only)

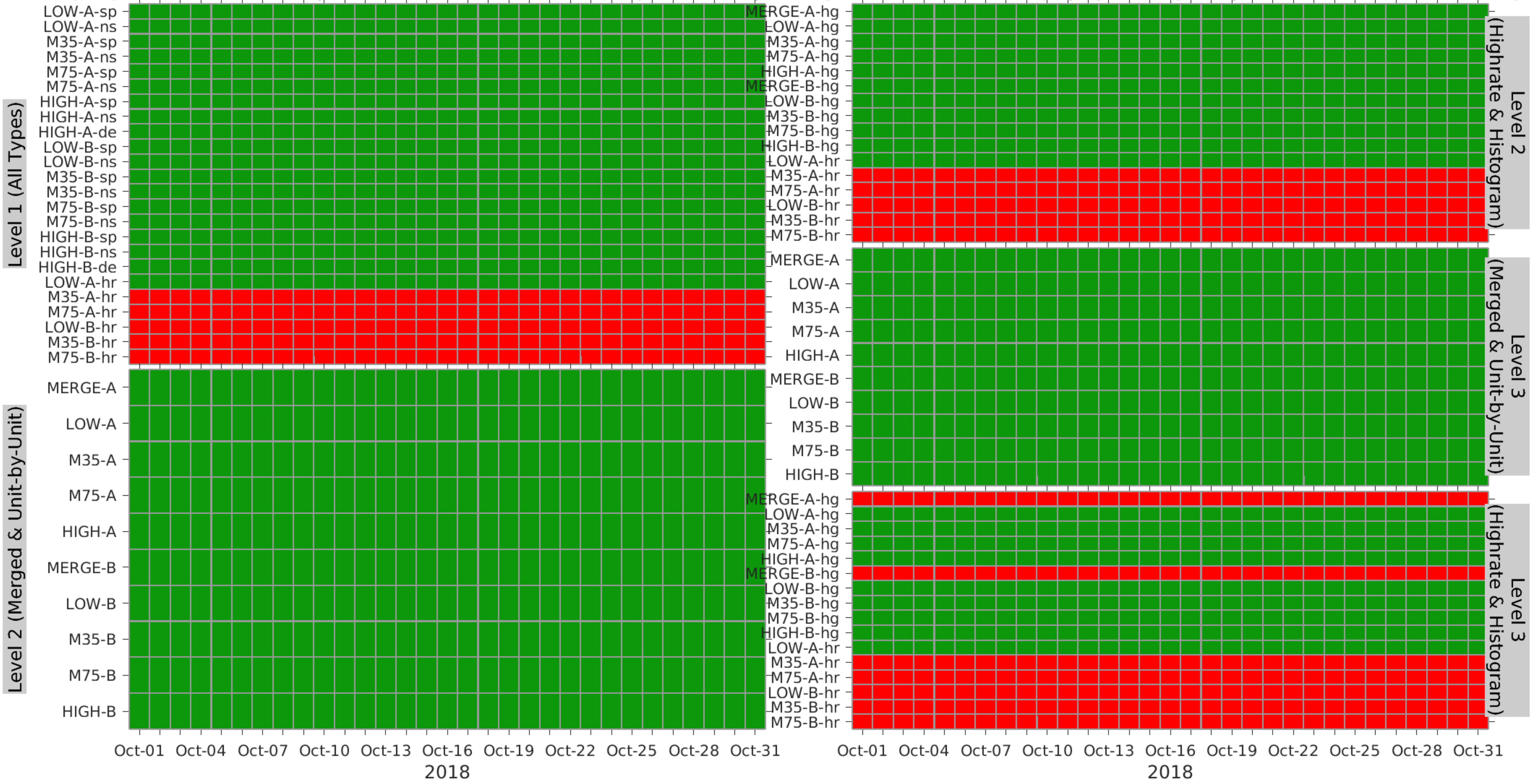

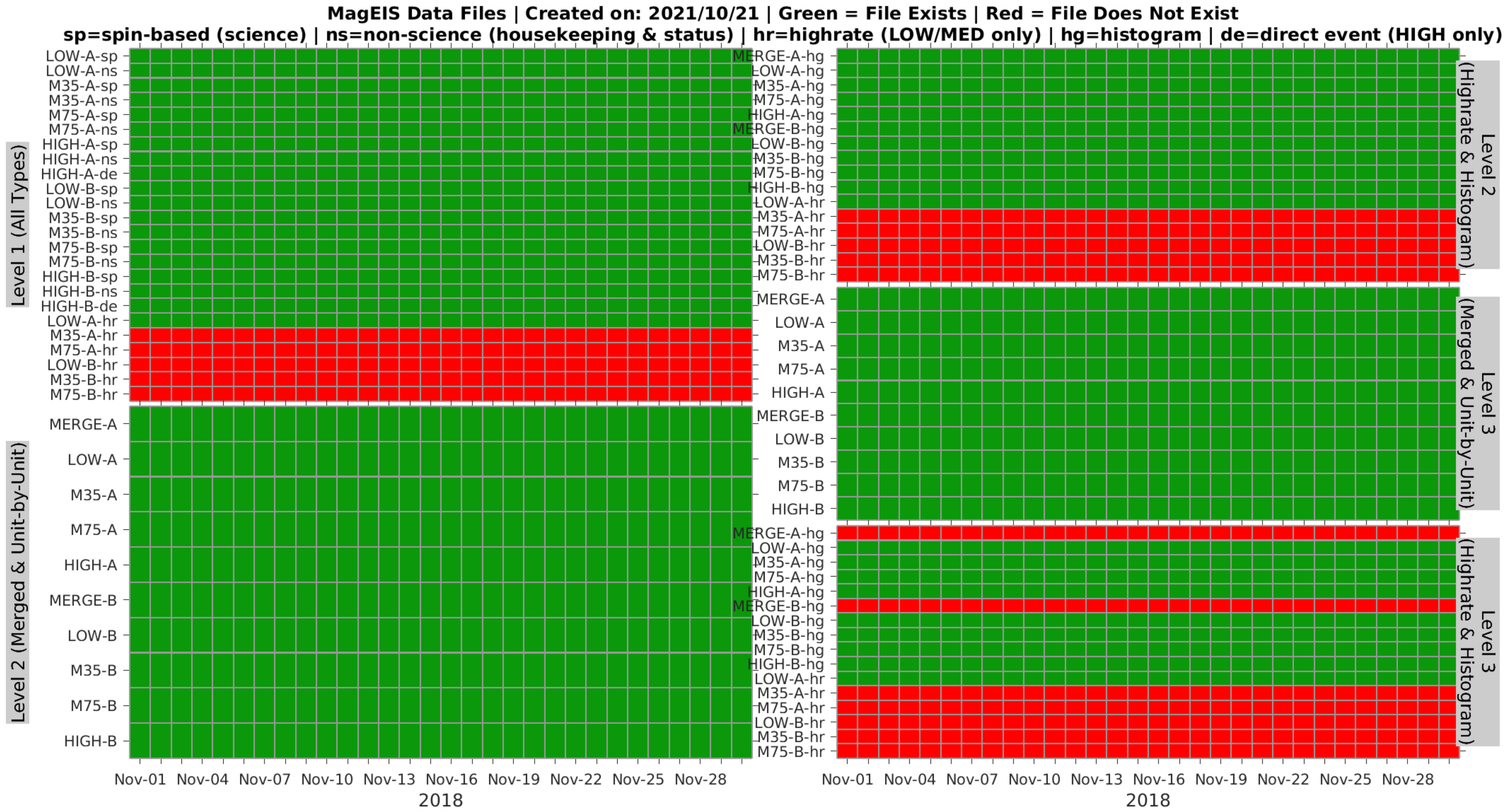
MagEIS Data Files | Created on: 2021/10/21 | Green = File Exists | Red = File Does Not Exist
sp=spin-based (science) | ns=non-science (housekeeping & status) | hr=highrate (LOW/MED only) | hg=histogram | de=direct event (HIGH only)
Level 1 (All Types)
LOW-A-sp
LOW-A-ns
M35-A-sp
M35-A-ns
M75-A-sp
M75-A-ns
HIGH-A-sp
HIGH-A-ns
HIGH-A-de
LOW-B-sp
LOW-B-ns
M35-B-sp
M35-B-ns
M75-B-sp
M75-B-ns
HIGH-B-sp
HIGH-B-ns
HIGH-B-de
LOW-A-hr
M35-A-hr
M75-A-hr
LOW-B-hr
M35-B-hr
M75-B-hr
Level 2 (Merged & Unit-by-Unit)
MERGE-A
LOW-A
M35-A
M75-A
HIGH-A
MERGE-B
LOW-B
M35-B
M75-B
HIGH-B
Level 2 (Highrate & Histogram)
MERGE-A-hg
LOW-A-hg
M35-A-hg
M75-A-hg
HIGH-A-hg
MERGE-B-hg
LOW-B-hg
M35-B-hg
M75-B-hg
HIGH-B-hg
LOW-A-hr
M35-A-hr
M75-A-hr
LOW-B-hr
M35-B-hr
M75-B-hr
Level 3 (Merged & Unit-by-Unit)
MERGE-A
LOW-A
M35-A
M75-A
HIGH-A
MERGE-B
LOW-B
M35-B
M75-B
HIGH-B
Level 3 (Highrate & Histogram)
MERGE-A-hg
LOW-A-hg
M35-A-hg
M75-A-hg
HIGH-A-hg
MERGE-B-hg
LOW-B-hg
M35-B-hg
M75-B-hg
HIGH-B-hg
LOW-A-hr
M35-A-hr
M75-A-hr
LOW-B-hr
M35-B-hr
M75-B-hr
Nov-01 Nov-04 Nov-07 Nov-10 Nov-13 Nov-16 Nov-19 Nov-22 Nov-25 Nov-28
2018

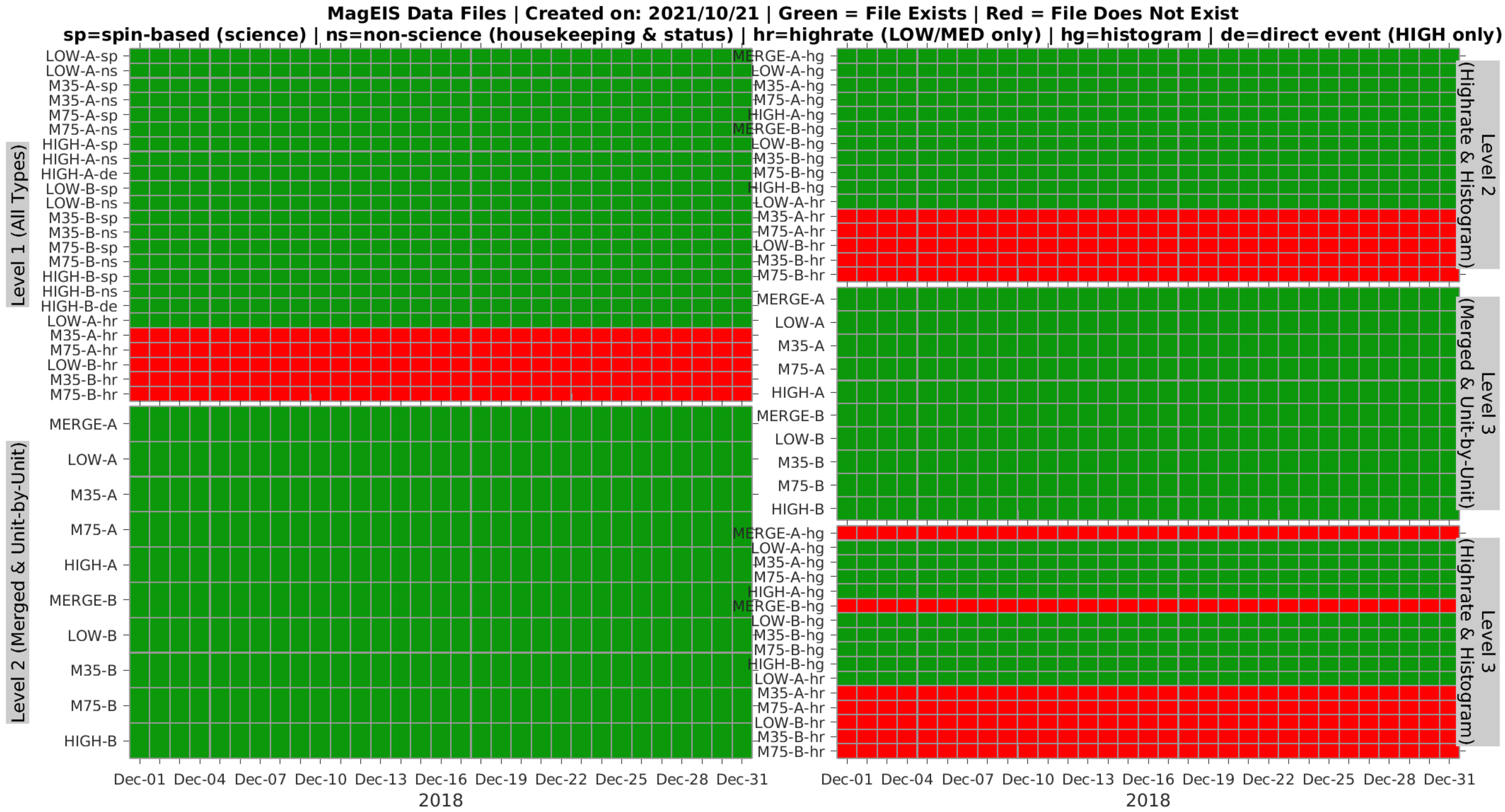
MagEIS Data Files | Created on: 2021/10/21 | Green = File Exists | Red = File Does Not Exist
sp=spin-based (science) | ns=non-science (housekeeping & status) | hr=highrate (LOW/MED only) | hg=histogram | de=direct event (HIGH only)
Level 1 (All Types)
LOW-A-sp
LOW-A-ns
M35-A-sp
M35-A-ns
M75-A-sp
M75-A-ns
HIGH-A-sp
HIGH-A-ns
HIGH-A-de
LOW-B-sp
LOW-B-ns
M35-B-sp
M35-B-ns
M75-B-sp
M75-B-ns
HIGH-B-sp
HIGH-B-ns
HIGH-B-de
LOW-A-hr
M35-A-hr
M75-A-hr
LOW-B-hr
M35-B-hr
M75-B-hr
Level 2 (Merged & Unit-by-Unit)
MERGE-A
LOW-A
M35-A
M75-A
HIGH-A
MERGE-B
LOW-B
M35-B
M75-B
HIGH-B
Dec-01
Dec-04
Dec-07
Dec-10
Dec-13
Dec-16
Dec-19
Dec-22
Dec-25
Dec-28
Dec-31
2018
Level 2 (Highrate & Histogram)
MERGE-A-hg
LOW-A-hg
M35-A-hg
M75-A-hg
HIGH-A-hg
MERGE-B-hg
LOW-B-hg
M35-B-hg
M75-B-hg
HIGH-B-hg
LOW-A-hr
M35-A-hr
M75-A-hr
LOW-B-hr
M35-B-hr
M75-B-hr
Level 3 (Merged & Unit-by-Unit)
MERGE-A
LOW-A
M35-A
M75-A
HIGH-A
MERGE-B
LOW-B
M35-B
M75-B
HIGH-B
Level 3 (Highrate & Histogram)
MERGE-A-hg
LOW-A-hg
M35-A-hg
M75-A-hg
HIGH-A-hg
MERGE-B-hg
LOW-B-hg
M35-B-hg
M75-B-hg
HIGH-B-hg
LOW-A-hr
M35-A-hr
M75-A-hr
LOW-B-hr
M35-B-hr
M75-B-hr
Dec-01
Dec-04
Dec-07
Dec-10
Dec-13
Dec-16
Dec-19
Dec-22
Dec-25
Dec-28
Dec-31
2018

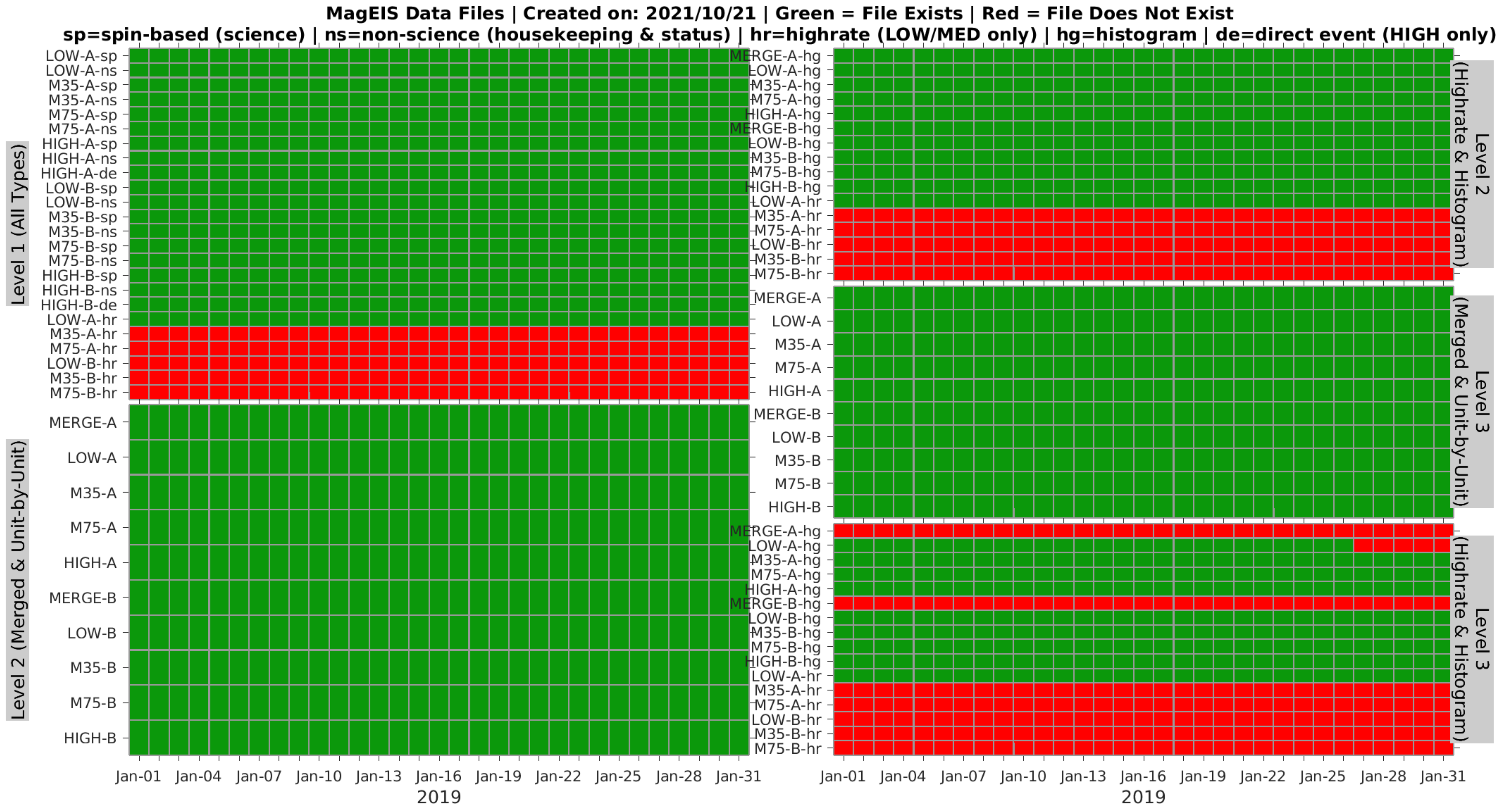

MagEIS Data Files | Created on: 2021/10/21 | Green = File Exists | Red = File Does Not Exist
sp=spin-based (science) | ns=non-science (housekeeping & status) | hr=highrate (LOW/MED only) | hg=histogram | de=direct event (HIGH only)
Level 1 (All Types)
LOW-A-sp
LOW-A-ns
M35-A-sp
M35-A-ns
M75-A-sp
M75-A-ns
HIGH-A-sp
HIGH-A-ns
HIGH-A-de
LOW-B-sp
LOW-B-ns
M35-B-sp
M35-B-ns
M75-B-sp
M75-B-ns
HIGH-B-sp
HIGH-B-ns
HIGH-B-de
LOW-A-hr
M35-A-hr
M75-A-hr
LOW-B-hr
M35-B-hr
M75-B-hr
Level 2 (Merged & Unit-by-Unit)
MERGE-A
LOW-A
M35-A
M75-A
HIGH-A
MERGE-B
LOW-B
M35-B
M75-B
HIGH-B
Level 2 (Highrate & Histogram)
MERGE-A-hg
LOW-A-hg
M35-A-hg
M75-A-hg
HIGH-A-hg
MERGE-B-hg
LOW-B-hg
M35-B-hg
M75-B-hg
HIGH-B-hg
LOW-A-hr
M35-A-hr
M75-A-hr
LOW-B-hr
M35-B-hr
M75-B-hr
Level 3 (Merged & Unit-by-Unit)
Level 3 (Highrate & Histogram)
Jan-01
Jan-04
Jan-07
Jan-10
Jan-13
Jan-16
Jan-19
Jan-22
Jan-25
Jan-28
Jan-31
2019

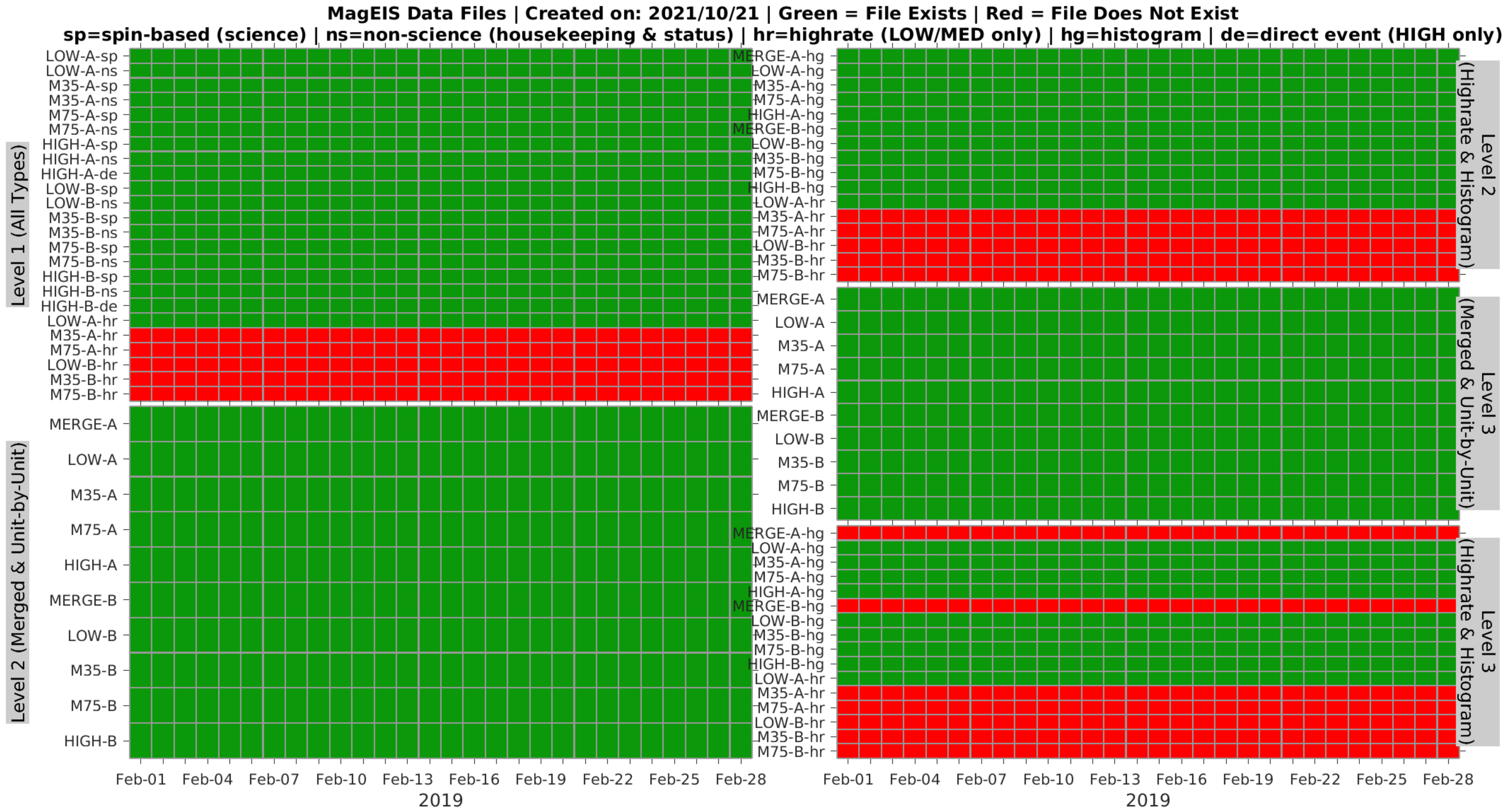
MagEIS Data Files | Created on: 2021/10/21 | Green = File Exists | Red = File Does Not Exist
sp=spin-based (science) | ns=non-science (housekeeping & status) | hr=highrate (LOW/MED only) | hg=histogram | de=direct event (HIGH only)
Level 1 (All Types)
LOW-A-sp
LOW-A-ns
M35-A-sp
M35-A-ns
M75-A-sp
M75-A-ns
HIGH-A-sp
HIGH-A-ns
HIGH-A-de
LOW-B-sp
LOW-B-ns
M35-B-sp
M35-B-ns
M75-B-sp
M75-B-ns
HIGH-B-sp
HIGH-B-ns
HIGH-B-de
LOW-A-hr
M35-A-hr
M75-A-hr
LOW-B-hr
M35-B-hr
M75-B-hr
Level 2 (Merged & Unit-by-Unit)
MERGE-A
LOW-A
M35-A
M75-A
HIGH-A
MERGE-B
LOW-B
M35-B
M75-B
HIGH-B
Level 2 (Highrate & Histogram)
MERGE-A-hg
LOW-A-hg
M35-A-hg
M75-A-hg
HIGH-A-hg
MERGE-B-hg
LOW-B-hg
M35-B-hg
M75-B-hg
HIGH-B-hg
LOW-A-hr
M35-A-hr
M75-A-hr
LOW-B-hr
M35-B-hr
M75-B-hr
Level 3 (Merged & Unit-by-Unit)
MERGE-A
LOW-A
M35-A
M75-A
HIGH-A
MERGE-B
LOW-B
M35-B
M75-B
HIGH-B
Level 3 (Highrate & Histogram)
MERGE-A-hg
LOW-A-hg
M35-A-hg
M75-A-hg
HIGH-A-hg
MERGE-B-hg
LOW-B-hg
M35-B-hg
M75-B-hg
HIGH-B-hg
LOW-A-hr
M35-A-hr
M75-A-hr
LOW-B-hr
M35-B-hr
M75-B-hr
Feb-01
Feb-04
Feb-07
Feb-10
Feb-13
Feb-16
Feb-19
Feb-22
Feb-25
Feb-28
2019

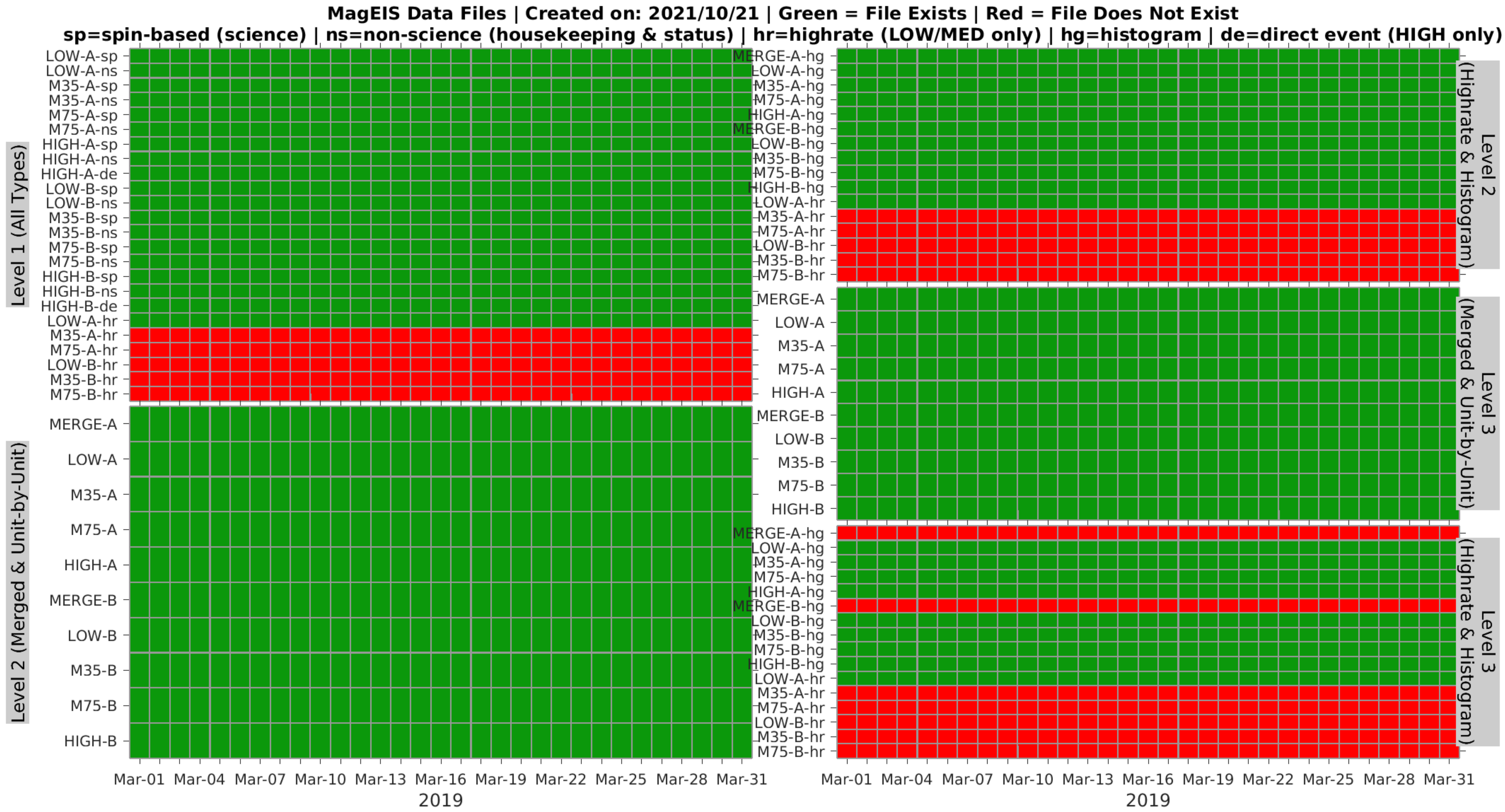

MagEIS Data Files | Created on: 2021/10/21 | Green = File Exists | Red = File Does Not Exist
sp=spin-based (science) | ns=non-science (housekeeping & status) | hr=highrate (LOW/MED only) | hg=histogram | de=direct event (HIGH only)
Level 1 (All Types)
LOW-A-sp
LOW-A-ns
M35-A-sp
M35-A-ns
M75-A-sp
M75-A-ns
HIGH-A-sp
HIGH-A-ns
HIGH-A-de
LOW-B-sp
LOW-B-ns
M35-B-sp
M35-B-ns
M75-B-sp
M75-B-ns
HIGH-B-sp
HIGH-B-ns
HIGH-B-de
LOW-A-hr
M35-A-hr
M75-A-hr
LOW-B-hr
M35-B-hr
M75-B-hr
Level 2 (Merged & Unit-by-Unit)
MERGE-A
LOW-A
M35-A
M75-A
HIGH-A
MERGE-B
LOW-B
M35-B
M75-B
HIGH-B
Level 2 (Highrate & Histogram)
MERGE-A-hg
LOW-A-hg
M35-A-hg
M75-A-hg
HIGH-A-hg
MERGE-B-hg
LOW-B-hg
M35-B-hg
M75-B-hg
HIGH-B-hg
LOW-A-hr
M35-A-hr
M75-A-hr
LOW-B-hr
M35-B-hr
M75-B-hr
Level 3 (Merged & Unit-by-Unit)
MERGE-A
LOW-A
M35-A
M75-A
HIGH-A
MERGE-B
LOW-B
M35-B
M75-B
HIGH-B
Level 3 (Highrate & Histogram)
MERGE-A-hg
LOW-A-hg
M35-A-hg
M75-A-hg
HIGH-A-hg
MERGE-B-hg
LOW-B-hg
M35-B-hg
M75-B-hg
HIGH-B-hg
LOW-A-hr
M35-A-hr
M75-A-hr
LOW-B-hr
M35-B-hr
M75-B-hr
Mar-01 Mar-04 Mar-07 Mar-10 Mar-13 Mar-16 Mar-19 Mar-22 Mar-25 Mar-28 Mar-31
2019

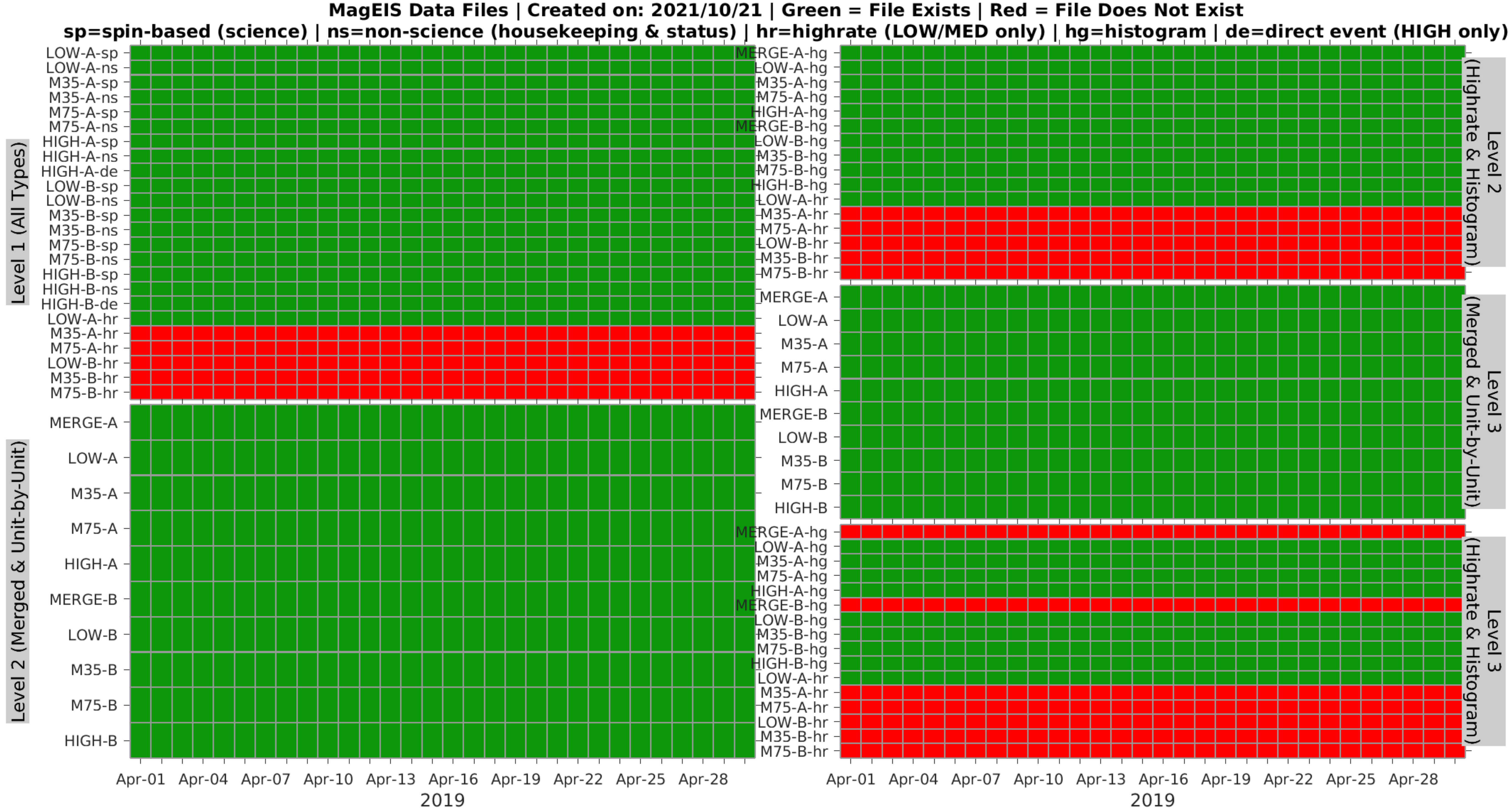

MagEIS Data Files | Created on: 2021/10/21 | Green = File Exists | Red = File Does Not Exist
sp=spin-based (science) | ns=non-science (housekeeping & status) | hr=highrate (LOW/MED only) | hg=histogram | de=direct event (HIGH only)
Level 1 (All Types)
LOW-A-sp
LOW-A-ns
M35-A-sp
M35-A-ns
M75-A-sp
M75-A-ns
HIGH-A-sp
HIGH-A-ns
HIGH-A-de
LOW-B-sp
LOW-B-ns
M35-B-sp
M35-B-ns
M75-B-sp
M75-B-ns
HIGH-B-sp
HIGH-B-ns
HIGH-B-de
LOW-A-hr
M35-A-hr
M75-A-hr
LOW-B-hr
M35-B-hr
M75-B-hr
Level 2 (Merged & Unit-by-Unit)
MERGE-A
LOW-A
M35-A
M75-A
HIGH-A
MERGE-B
LOW-B
M35-B
M75-B
HIGH-B
Level 2 (Highrate & Histogram)
MERGE-A-hg
LOW-A-hg
M35-A-hg
M75-A-hg
HIGH-A-hg
MERGE-B-hg
LOW-B-hg
M35-B-hg
M75-B-hg
HIGH-B-hg
LOW-A-hr
M35-A-hr
M75-A-hr
LOW-B-hr
M35-B-hr
M75-B-hr
Level 3 (Merged & Unit-by-Unit)
MERGE-A
LOW-A
M35-A
M75-A
HIGH-A
MERGE-B
LOW-B
M35-B
M75-B
HIGH-B
Level 3 (Highrate & Histogram)
MERGE-A-hg
LOW-A-hg
M35-A-hg
M75-A-hg
HIGH-A-hg
MERGE-B-hg
LOW-B-hg
M35-B-hg
M75-B-hg
HIGH-B-hg
LOW-A-hr
M35-A-hr
M75-A-hr
LOW-B-hr
M35-B-hr
M75-B-hr
Apr-01 Apr-04 Apr-07 Apr-10 Apr-13 Apr-16 Apr-19 Apr-22 Apr-25 Apr-28
2019
Apr-01 Apr-04 Apr-07 Apr-10 Apr-13 Apr-16 Apr-19 Apr-22 Apr-25 Apr-28
2019

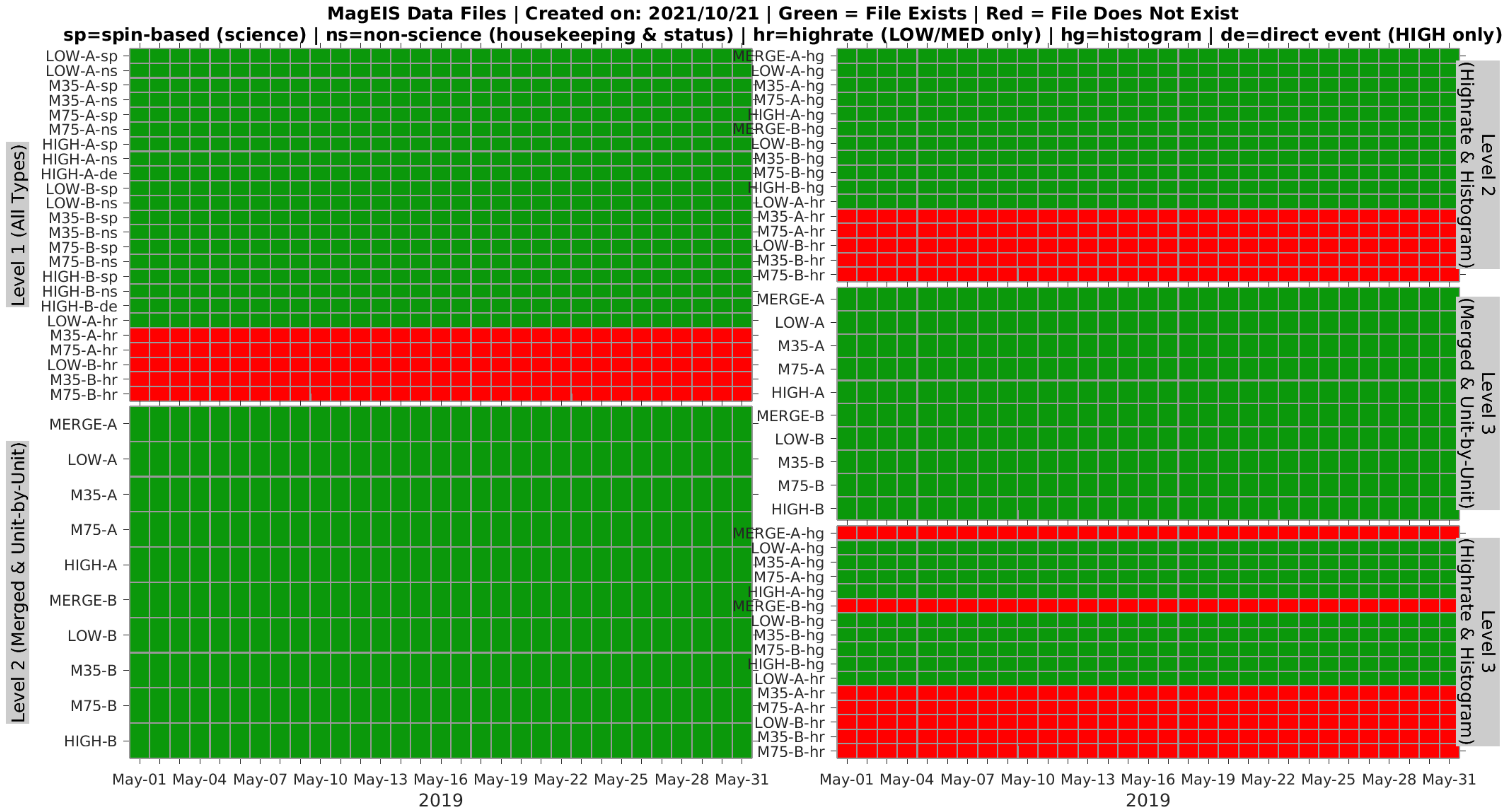
MagEIS Data Files | Created on: 2021/10/21 | Green = File Exists | Red = File Does Not Exist
sp=spin-based (science) | ns=non-science (housekeeping & status) | hr=highrate (LOW/MED only) | hg=histogram | de=direct event (HIGH only)
Level 1 (All Types)
LOW-A-sp
LOW-A-ns
M35-A-sp
M35-A-ns
M75-A-sp
M75-A-ns
HIGH-A-sp
HIGH-A-ns
HIGH-A-de
LOW-B-sp
LOW-B-ns
M35-B-sp
M35-B-ns
M75-B-sp
M75-B-ns
HIGH-B-sp
HIGH-B-ns
HIGH-B-de
LOW-A-hr
M35-A-hr
M75-A-hr
LOW-B-hr
M35-B-hr
M75-B-hr
Level 2 (Merged & Unit-by-Unit)
MERGE-A
LOW-A
M35-A
M75-A
HIGH-A
MERGE-B
LOW-B
M35-B
M75-B
HIGH-B
Level 2 (Highrate & Histogram)
MERGE-A-hg
LOW-A-hg
M35-A-hg
M75-A-hg
HIGH-A-hg
MERGE-B-hg
LOW-B-hg
M35-B-hg
M75-B-hg
HIGH-B-hg
LOW-A-hr
M35-A-hr
M75-A-hr
LOW-B-hr
M35-B-hr
M75-B-hr
Level 3 (Merged & Unit-by-Unit)
MERGE-A
LOW-A
M35-A
M75-A
HIGH-A
MERGE-B
LOW-B
M35-B
M75-B
HIGH-B
Level 3 (Highrate & Histogram)
MERGE-A-hg
LOW-A-hg
M35-A-hg
M75-A-hg
HIGH-A-hg
MERGE-B-hg
LOW-B-hg
M35-B-hg
M75-B-hg
HIGH-B-hg
LOW-A-hr
M35-A-hr
M75-A-hr
LOW-B-hr
M35-B-hr
M75-B-hr
May-01 May-04 May-07 May-10 May-13 May-16 May-19 May-22 May-25 May-28 May-31
2019

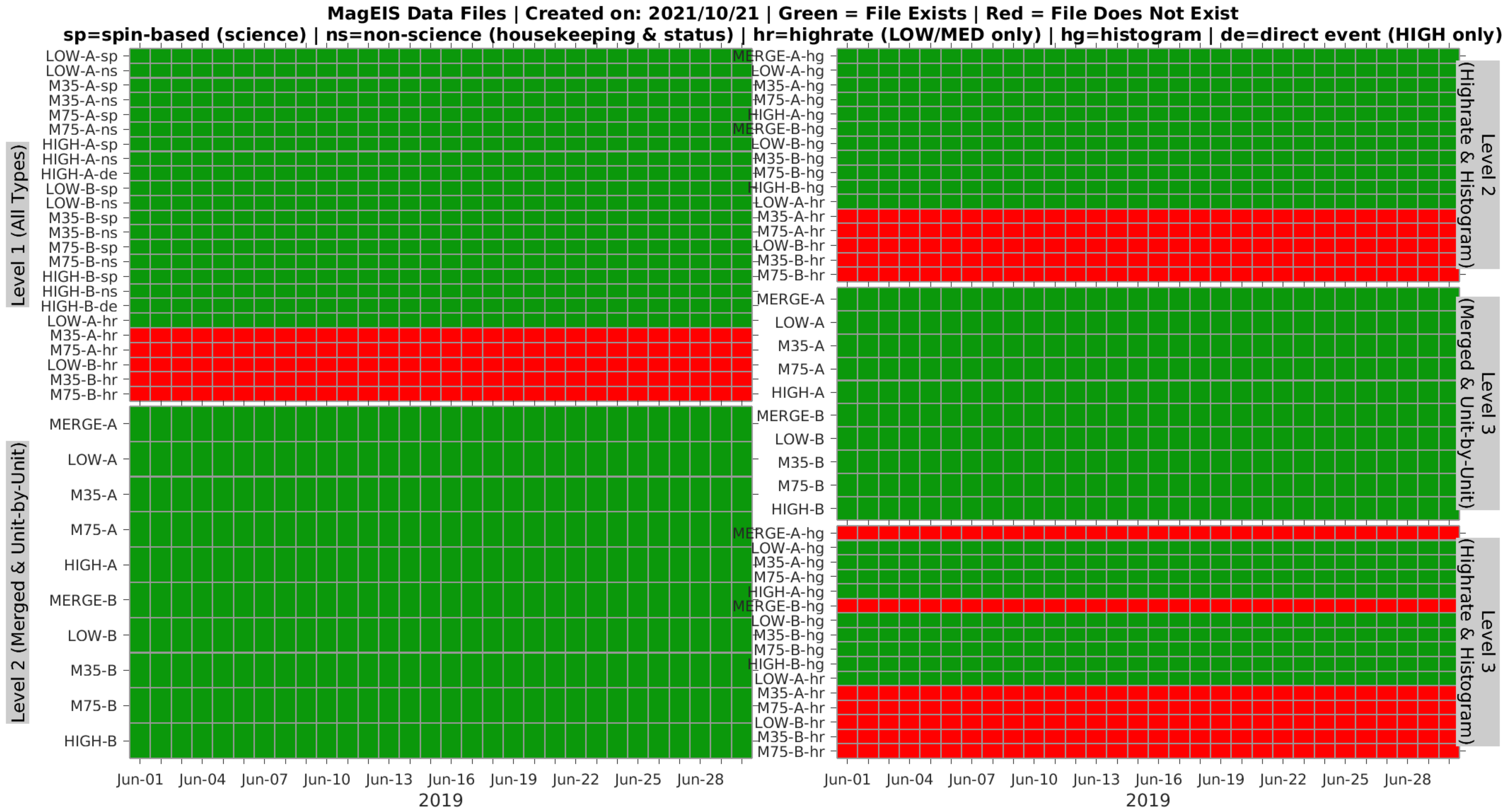
MagEIS Data Files | Created on: 2021/10/21 | Green = File Exists | Red = File Does Not Exist
sp=spin-based (science) | ns=non-science (housekeeping & status) | hr=highrate (LOW/MED only) | hg=histogram | de=direct event (HIGH only)
Level 1 (All Types)
LOW-A-sp
LOW-A-ns
M35-A-sp
M35-A-ns
M75-A-sp
M75-A-ns
HIGH-A-sp
HIGH-A-ns
HIGH-A-de
LOW-B-sp
LOW-B-ns
M35-B-sp
M35-B-ns
M75-B-sp
M75-B-ns
HIGH-B-sp
HIGH-B-ns
HIGH-B-de
LOW-A-hr
M35-A-hr
M75-A-hr
LOW-B-hr
M35-B-hr
M75-B-hr
Level 2 (Merged & Unit-by-Unit)
MERGE-A
LOW-A
M35-A
M75-A
HIGH-A
MERGE-B
LOW-B
M35-B
M75-B
HIGH-B
Level 2 (Highrate & Histogram)
MERGE-A-hg
LOW-A-hg
M35-A-hg
M75-A-hg
HIGH-A-hg
MERGE-B-hg
LOW-B-hg
M35-B-hg
M75-B-hg
HIGH-B-hg
LOW-A-hr
M35-A-hr
M75-A-hr
LOW-B-hr
M35-B-hr
M75-B-hr
Level 3 (Merged & Unit-by-Unit)
MERGE-A
LOW-A
M35-A
M75-A
HIGH-A
MERGE-B
LOW-B
M35-B
M75-B
HIGH-B
Level 3 (Highrate & Histogram)
MERGE-A-hg
LOW-A-hg
M35-A-hg
M75-A-hg
HIGH-A-hg
MERGE-B-hg
LOW-B-hg
M35-B-hg
M75-B-hg
HIGH-B-hg
LOW-A-hr
M35-A-hr
M75-A-hr
LOW-B-hr
M35-B-hr
M75-B-hr
Jun-01
Jun-04
Jun-07
Jun-10
Jun-13
Jun-16
Jun-19
Jun-22
Jun-25
Jun-28
2019

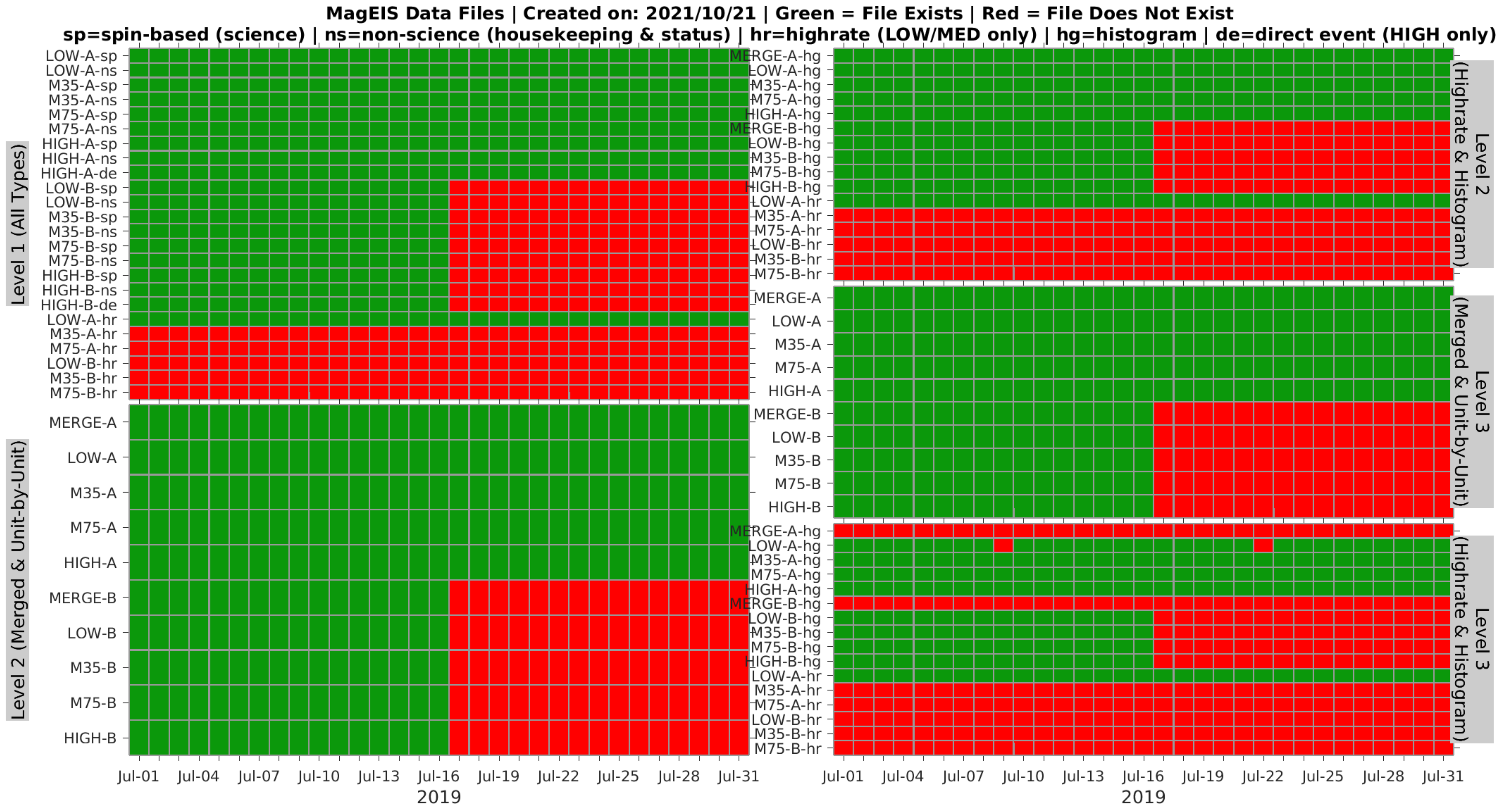
MagEIS Data Files | Created on: 2021/10/21 | Green = File Exists | Red = File Does Not Exist
sp=spin-based (science) | ns=non-science (housekeeping & status) | hr=highrate (LOW/MED only) | hg=histogram | de=direct event (HIGH only)
Level 1 (All Types)
LOW-A-sp
LOW-A-ns
M35-A-sp
M35-A-ns
M75-A-sp
M75-A-ns
HIGH-A-sp
HIGH-A-ns
HIGH-A-de
LOW-B-sp
LOW-B-ns
M35-B-sp
M35-B-ns
M75-B-sp
M75-B-ns
HIGH-B-sp
HIGH-B-ns
HIGH-B-de
LOW-A-hr
M35-A-hr
M75-A-hr
LOW-B-hr
M35-B-hr
M75-B-hr
Level 2 (Merged & Unit-by-Unit)
MERGE-A
LOW-A
M35-A
M75-A
HIGH-A
MERGE-B
LOW-B
M35-B
M75-B
HIGH-B
Level 2 (Highrate & Histogram)
MERGE-A-hg
LOW-A-hg
M35-A-hg
M75-A-hg
HIGH-A-hg
MERGE-B-hg
LOW-B-hg
M35-B-hg
M75-B-hg
HIGH-B-hg
LOW-A-hr
M35-A-hr
M75-A-hr
LOW-B-hr
M35-B-hr
M75-B-hr
Level 3 (Merged & Unit-by-Unit)
MERGE-A
LOW-A
M35-A
M75-A
HIGH-A
MERGE-B
LOW-B
M35-B
M75-B
HIGH-B
Level 3 (Highrate & Histogram)
MERGE-A-hg
LOW-A-hg
M35-A-hg
M75-A-hg
HIGH-A-hg
MERGE-B-hg
LOW-B-hg
M35-B-hg
M75-B-hg
HIGH-B-hg
LOW-A-hr
M35-A-hr
M75-A-hr
LOW-B-hr
M35-B-hr
M75-B-hr
Jul-01
Jul-04
Jul-07
Jul-10
Jul-13
Jul-16
Jul-19
Jul-22
Jul-25
Jul-28
Jul-31
2019

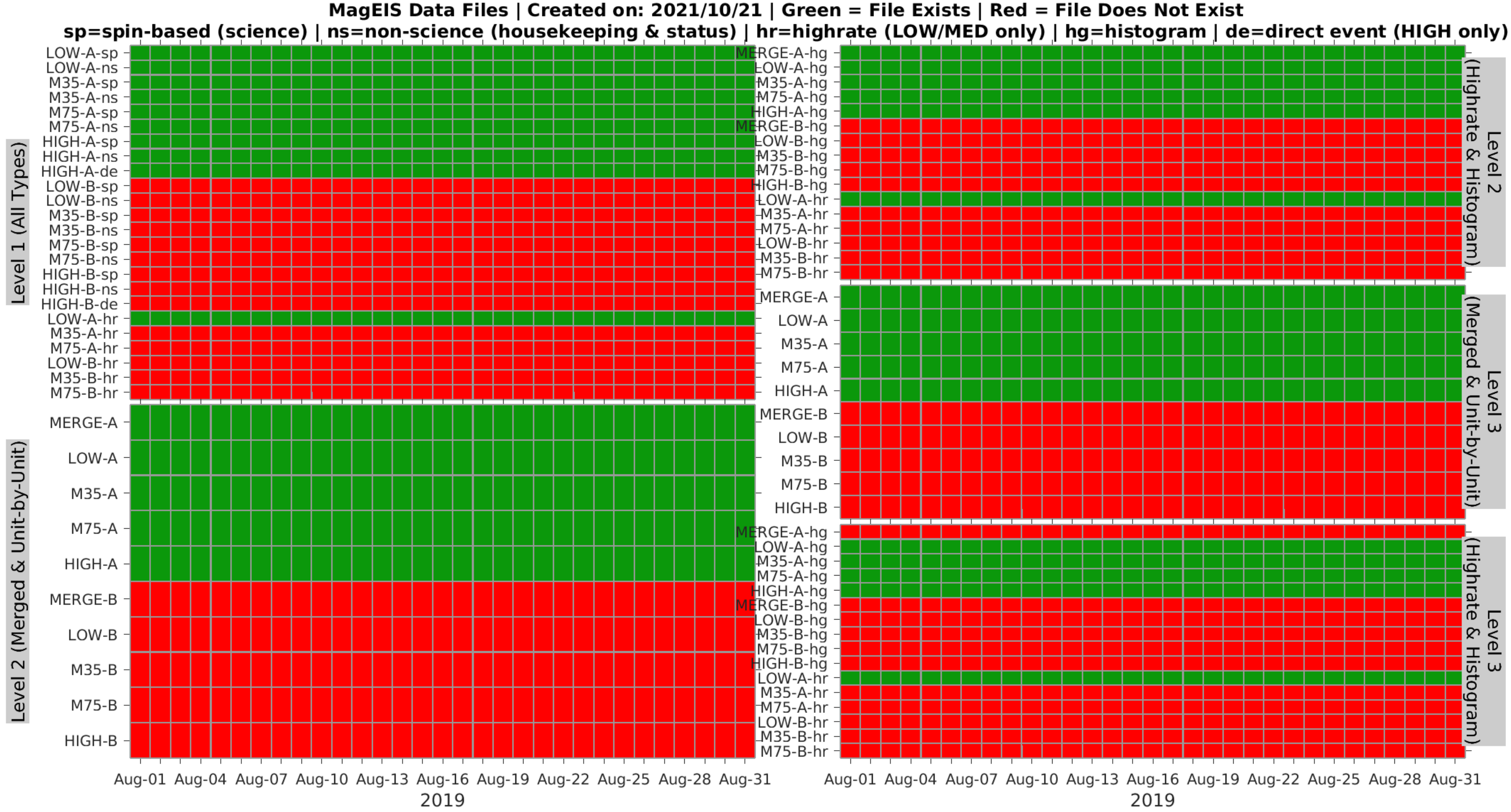
MagEIS Data Files | Created on: 2021/10/21 | Green = File Exists | Red = File Does Not Exist
sp=spin-based (science) | ns=non-science (housekeeping & status) | hr=highrate (LOW/MED only) | hg=histogram | de=direct event (HIGH only)
Level 1 (All Types)
LOW-A-sp
LOW-A-ns
M35-A-sp
M35-A-ns
M75-A-sp
M75-A-ns
HIGH-A-sp
HIGH-A-ns
HIGH-A-de
LOW-B-sp
LOW-B-ns
M35-B-sp
M35-B-ns
M75-B-sp
M75-B-ns
HIGH-B-sp
HIGH-B-ns
HIGH-B-de
LOW-A-hr
M35-A-hr
M75-A-hr
LOW-B-hr
M35-B-hr
M75-B-hr
Level 2 (Merged & Unit-by-Unit)
MERGE-A
LOW-A
M35-A
M75-A
HIGH-A
MERGE-B
LOW-B
M35-B
M75-B
HIGH-B
Level 2 (Highrate & Histogram)
MERGE-A-hg
LOW-A-hg
M35-A-hg
M75-A-hg
HIGH-A-hg
MERGE-B-hg
LOW-B-hg
M35-B-hg
M75-B-hg
HIGH-B-hg
LOW-A-hr
M35-A-hr
M75-A-hr
LOW-B-hr
M35-B-hr
M75-B-hr
Level 3 (Merged & Unit-by-Unit)
MERGE-A
LOW-A
M35-A
M75-A
HIGH-A
MERGE-B
LOW-B
M35-B
M75-B
HIGH-B
Level 3 (Highrate & Histogram)
MERGE-A-hg
LOW-A-hg
M35-A-hg
M75-A-hg
HIGH-A-hg
MERGE-B-hg
LOW-B-hg
M35-B-hg
M75-B-hg
HIGH-B-hg
LOW-A-hr
M35-A-hr
M75-A-hr
LOW-B-hr
M35-B-hr
M75-B-hr
Aug-01
Aug-04
Aug-07
Aug-10
Aug-13
Aug-16
Aug-19
Aug-22
Aug-25
Aug-28
Aug-31
2019

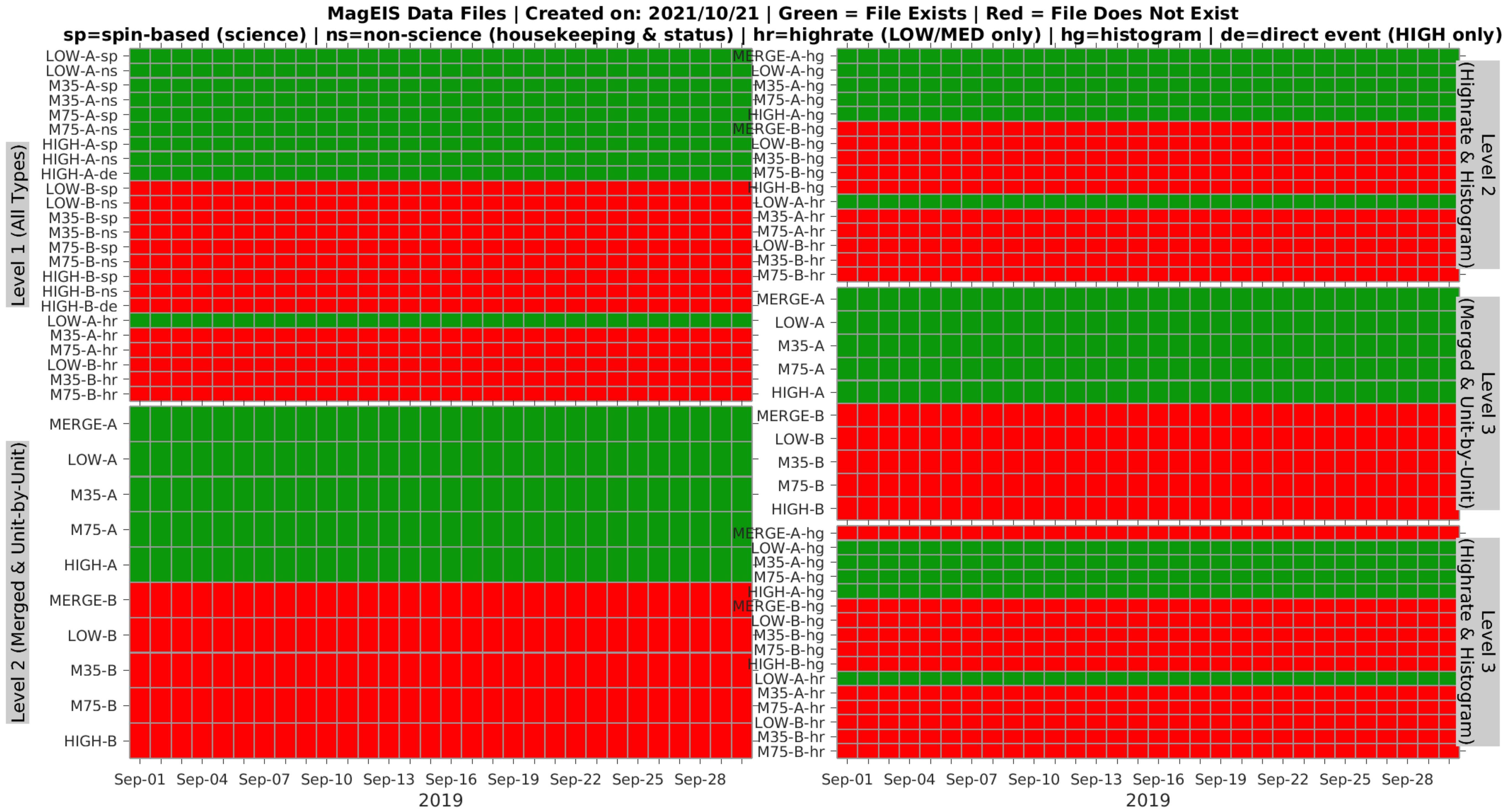
MagEIS Data Files | Created on: 2021/10/21 | Green = File Exists | Red = File Does Not Exist
sp=spin-based (science) | ns=non-science (housekeeping & status) | hr=highrate (LOW/MED only) | hg=histogram | de=direct event (HIGH only)
Level 1 (All Types)
LOW-A-sp
LOW-A-ns
M35-A-sp
M35-A-ns
M75-A-sp
M75-A-ns
HIGH-A-sp
HIGH-A-ns
HIGH-A-de
LOW-B-sp
LOW-B-ns
M35-B-sp
M35-B-ns
M75-B-sp
M75-B-ns
HIGH-B-sp
HIGH-B-ns
HIGH-B-de
LOW-A-hr
M35-A-hr
M75-A-hr
LOW-B-hr
M35-B-hr
M75-B-hr
Level 2 (Merged & Unit-by-Unit)
MERGE-A
LOW-A
M35-A
M75-A
HIGH-A
MERGE-B
LOW-B
M35-B
M75-B
HIGH-B
Level 2 (Highrate & Histogram)
MERGE-A-hg
LOW-A-hg
M35-A-hg
M75-A-hg
HIGH-A-hg
MERGE-B-hg
LOW-B-hg
M35-B-hg
M75-B-hg
HIGH-B-hg
LOW-A-hr
M35-A-hr
M75-A-hr
LOW-B-hr
M35-B-hr
M75-B-hr
Level 3 (Merged & Unit-by-Unit)
MERGE-A
LOW-A
M35-A
M75-A
HIGH-A
MERGE-B
LOW-B
M35-B
M75-B
HIGH-B
Level 3 (Highrate & Histogram)
MERGE-A-hg
LOW-A-hg
M35-A-hg
M75-A-hg
HIGH-A-hg
MERGE-B-hg
LOW-B-hg
M35-B-hg
M75-B-hg
HIGH-B-hg
LOW-A-hr
M35-A-hr
M75-A-hr
LOW-B-hr
M35-B-hr
M75-B-hr
Sep-01
Sep-04
Sep-07
Sep-10
Sep-13
Sep-16
Sep-19
Sep-22
Sep-25
Sep-28
2019

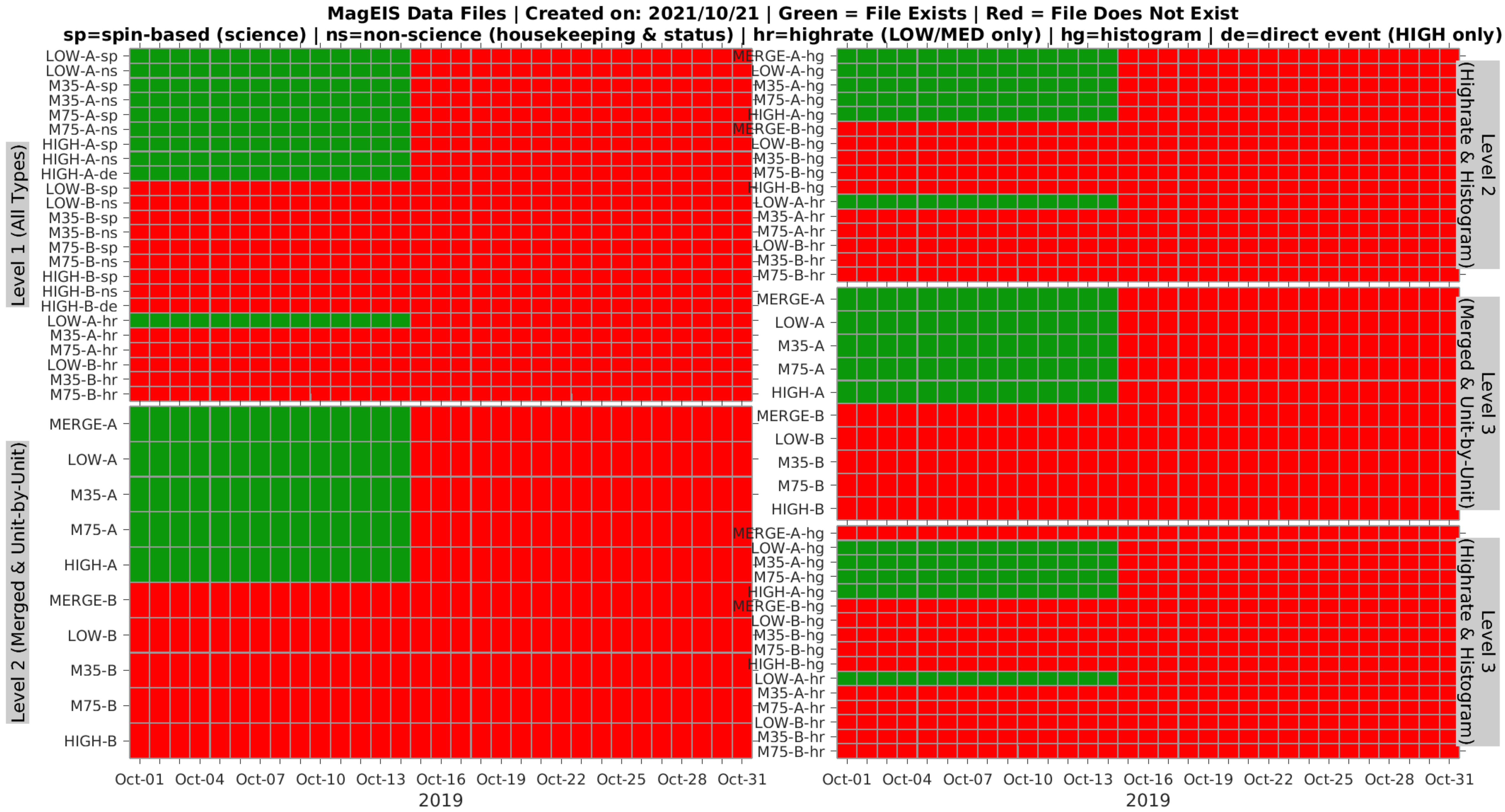
MagEIS Data Files | Created on: 2021/10/21 | Green = File Exists | Red = File Does Not Exist
sp=spin-based (science) | ns=non-science (housekeeping & status) | hr=highrate (LOW/MED only) | hg=histogram | de=direct event (HIGH only)
Level 1 (All Types)
LOW-A-sp
LOW-A-ns
M35-A-sp
M35-A-ns
M75-A-sp
M75-A-ns
HIGH-A-sp
HIGH-A-ns
HIGH-A-de
LOW-B-sp
LOW-B-ns
M35-B-sp
M35-B-ns
M75-B-sp
M75-B-ns
HIGH-B-sp
HIGH-B-ns
HIGH-B-de
LOW-A-hr
M35-A-hr
M75-A-hr
LOW-B-hr
M35-B-hr
M75-B-hr
Level 2 (Merged & Unit-by-Unit)
MERGE-A
LOW-A
M35-A
M75-A
HIGH-A
MERGE-B
LOW-B
M35-B
M75-B
HIGH-B
Level 2 (Highrate & Histogram)
MERGE-A-hg
LOW-A-hg
M35-A-hg
M75-A-hg
HIGH-A-hg
MERGE-B-hg
LOW-B-hg
M35-B-hg
M75-B-hg
HIGH-B-hg
LOW-A-hr
M35-A-hr
M75-A-hr
LOW-B-hr
M35-B-hr
M75-B-hr
Level 3 (Merged & Unit-by-Unit)
MERGE-A
LOW-A
M35-A
M75-A
HIGH-A
MERGE-B
LOW-B
M35-B
M75-B
HIGH-B
Level 3 (Highrate & Histogram)
MERGE-A-hg
LOW-A-hg
M35-A-hg
M75-A-hg
HIGH-A-hg
MERGE-B-hg
LOW-B-hg
M35-B-hg
M75-B-hg
HIGH-B-hg
LOW-A-hr
M35-A-hr
M75-A-hr
LOW-B-hr
M35-B-hr
M75-B-hr
Oct-01
Oct-04
Oct-07
Oct-10
Oct-13
Oct-16
Oct-19
Oct-22
Oct-25
Oct-28
Oct-31
2019
