# Supplementary material for: The Magnetic Electron Ion Spectrometer: A Review of On-Orbit Sensor Performance, Data, Operations, and Science
Source: Space Sci Rev. 2021 Oct 28;217(8):80. doi: 10.1007/s11214-021-00855-2 (PMC8553741; doi:10.1007/s11214-021-00855-2)
Supplement: Supplementary file 8 — A chronological listing of configuration changes (e.g., LUT changes, accumulation parameter changes) and known instrumental/spacecraft issues that led to missing data files and/or large data gaps (PDF 60 kB) [file 11214_2021_855_MOESM8_ESM.pdf]

Van Allen Probes ECT/MagEIS: Major Configuration Changes

Contact: Seth Claudepierre, The Aerospace Corporation (seth@aero.org)
Last update: 2020/05/04

NOTES:

- MagEIS turn-on was 2012/09/06. Changes are only noted below for dates after 2012/10/05, as configuration changes were too frequent to document between turn-on and this date. Many of those changes were made during real-time contacts. And prior to 2012/12/21, MagEIS data should be used with extreme caution, as all 8 MagEIS units had numerous configuration changes and issues. In particular, the HIGH unit electron look-up-tables (LUTs) were invalid prior to 2012/10/04, making the data essentially unusable prior to this time. Prior to 2012/12/21, the HIGH units also had extensive changes in coincidence settings and the LOW/MED units were run in a different bias state. Please contact the MagEIS team if you wish to use this early data for scientific studies.
- Some changes below are of minor/little significance, while other are not (e.g., new energy channels definitions via LUT uploads). Also important are changes to sectoring and/or time cadence parameters (only major reconfigurations are noted below).
- With regard to detectors bias: “Low-bias” = 40V; “High-bias” = 125V (LOW), 375V (MED); “Normal-bias” = 75V (LOW), ~200V (MED), 320V (HIGH). The LOW/MED units were commanded into the normal bias state in Dec 2012 (HIGH was not modified from its pre-launch configuration).
- Commanding issues, anomalies, data loss (e.g., instrument in maintenance mode), threshold tuning, coincidence tuning are all noted.

2012/10/05: All 8 MagEIS units in the nominal telemetry mode. Main rate parameters: 1 spin cadence; 27 sectors/spin (LOW/MED), 16 sectors/spin (HIGH elec), 27 sectors/spin (HIGH prot). Histogram parameters: 12 spin cadence; 27 sectors/spin (LOW/MED); 16 sectors/spin (HIGH elec); 27 sectors/spin (HIGH prot).

2012/10/12:

- The M75-A unit bias reduced to the low-bias state. It was restored to the high-bias state on 2012/10/16.
- The M75-B unit bias reduced to the low-bias state. It was restored to the high-bias state on 2012/10/31.
- The M35-A unit bias reduced to the low-bias state. It was restored to the normal-bias state on 2012/12/20.
- The M35-B unit bias reduced to the low-bias state. It was restored to the normal-bias state on 2012/12/21.
- Data from these units during the low-bias time intervals indicated should only be used qualitatively.

2012/10/15: LOW-B new main rate and histogram LUTs uploaded. This changed the energy channel definitions (centroids and/or channel widths).

2012/10/16: The M75-A unit bias restored to the high-bias state.

2012/10/17: LOW-B new main rate LUT uploaded. This changed the energy channel definitions (centroids and/or channel widths).

2012/10/18: M75-A new histogram LUT uploaded. This changed the energy channel definitions (centroids and/or channel widths).

2012/10/24:

- M75-B new main rate LUT uploaded. This changed the energy channel definitions (centroids and/or channel widths).

- The coincidence window was changed on the HIGH-A, HIGH-B electron spectrometers from 20 microseconds to 10 microseconds.

2012/10/25: HIGH-A, HIGH-B rebooted into maintenance mode. Both units in maintenance mode until 2012/10/30 (no science data).
2012/10/28: End of RBSP 60 day commissioning period.
2012/10/30:

- Incorrect threshold settings on HIGH-A proton telescope. Proton data are invalid until 2012/11/06.
- HIGH-A, HIGH-B rebooted into science mode.

2012/10/31: The M75-B unit bias restored to the high-bias state.
2012/11/06: New (corrected) threshold settings on HIGH-A proton telescope.
2012/11/28: A new proton histogram LUT was uploaded to the HIGH-B unit. This LUT removes the LP data from the histogram and gives 128 histogram channels each to the MPA and MSD detectors. Note that this does not change/affect the main proton channels.
2012/12/13: New flight software uploaded to M75-B to allow for bias tuning. The bias was lowered on the M75-B unit to the normal-bias state.
2012/12/14: New flight software uploaded to M75-A to allow for bias tuning. The bias was lowered on the M75-A unit to the normal-bias state.
2012/12/20:

- New flight software uploaded to LOW-A, M35-A, HIGH-A to allow for bias tuning. The bias was lowered on the LOW-A, M35-A, and M75-A units to the normal-bias state.
- The M35-A unit bias restored to the normal-bias state.

2012/12/21:

- New flight software uploaded to LOW-B, M35-B, HIGH-B to allow for bias tuning. The bias was lowered on the LOW-B, M35-B, and M75-B units to the normal-bias state.
- The M35-B unit bias restored to the normal-bias state.
- The coincidence window was changed on the HIGH-A electron spectrometer from 10 microseconds to 20 microseconds.

2012/12/22: The coincidence window was changed on the HIGH-B electron spectrometer from 10 microseconds to 20 microseconds.
2012/12/23: A new proton main rate LUT was uploaded to the HIGH-A unit. This changed the energy channel definitions (centroids and/or channel widths).
2013/02/13: A new proton histogram LUT was uploaded to the HIGH-A unit. Note that this does not change/affect the main proton channels.
2013/02/24: M75-A, M75-B new main rate LUTs uploaded. This changed the energy channel definitions (centroids and/or channel widths).
2013/03/25: Threshold changes on LOW-B, M35-B, M75-B.
2013/03/26: Threshold changes on LOW-A, M35-A, M75-A.
2013/03/31:

- At ~0000UTC new main rate LUTs were uploaded to all four MagEIS units (LOW, M35, M75, HIGH) on both spacecraft. This changed the energy channel definitions (centroids and/or channel widths). The commands to M35-A and M35-B were not successful and this resulted in an undesired instrument configuration (the LUT for M35-B was inadvertently uploaded to M35-A). LUTs were corrected on 2013/04/03.
- The coincidence window was changed on the HIGH-A electron spectrometer from 20 microseconds to 10 microseconds.
- The coincidence window was changed on the HIGH-B electron spectrometer from 20 microseconds to 10 microseconds.

2013/04/03:

- At ~0000UTC the correct main rate LUTs were uploaded to M35-A and M35-B (see note from 2013/03/31).
- The M35-B unit bias reduced to the low-bias state. It was not restored to the normal-bias state until 2013/05/29. Data from this unit during this time should only be used qualitatively.

2013/04/04:

- The coincidence window was changed on the HIGH-B electron spectrometer from 10 microseconds to 5 microseconds.
- A new proton histogram LUT was uploaded to the HIGH-A unit. This LUT increased the resolution for the 2 micron and 9 micron detectors, while retaining some of the MPA detector. Note that this does not change/affect the main proton channels.
- New HR LUTs were uploaded to all 6 LOW/MED units. These LUTs removed noisy pixels 0 and 1 from the HR channels

2013/04/28: The space weather data channels changed.

2013/05/29: The M35-B unit bias restored to the normal-bias state.

2013/07/03: HIGH-A, HIGH-B electron threshold changes. Any step function increases in HIGH unit electron flux around this time are likely non-physical.

2013/07/18: HIGH-A, HIGH-B electron threshold changes. Any step function increases in HIGH unit electron flux around this time are likely non-physical.

2013/07/24: A new proton histogram LUT was uploaded to the HIGH-A unit. This LUT excluded the MPA detector and used the entire histogram for the 2 micron and 9 micron detectors. Note that this does not change/affect the main proton channels.

2013/07/25: HIGH-B electron threshold changes. Any step function increases in HIGH unit electron flux around this time are likely non-physical.

2013/07/26: HIGH-A electron threshold changes. Any step function increases in HIGH unit electron flux around this time are likely non-physical.

2013/08/03: HIGH-A, HIGH-B electron threshold changes. Any step function increases in HIGH unit electron flux around this time are likely non-physical.

2013/08/14:

- HIGH-A electron threshold changes. Any step function increases in HIGH unit electron flux around this time are likely non-physical.
- The coincidence window was changed on the HIGH-A electron spectrometer from 10 microseconds to 5 microseconds.

2013/08/21: HIGH-A electron threshold changes. Any step function increases in HIGH unit electron flux around this time are likely non-physical.

2013/08/23: HIGH-A, HIGH-B perigee pulser sweeps no longer switch to the engineering LUTs for protons. The science mode LUT is used throughout the pulser sweep.

2013/09/26:

- A new proton histogram LUT was uploaded to the HIGH-A unit. This LUT includes data from all three proton detectors (MPA, 2 micron, and 9 micron). Note that this does not change/affect the main proton channels.
- HIGH-A electron threshold changes. Any step function increases in HIGH unit electron flux around this time are likely non-physical.

2013/10/02: HIGH-A electron pixel 0 anomaly. At ~1125 UTC, pixel 0 (~900 keV) on HIGH-A failed and no longer produces valid data.

2013/10/04: LOW-A, LOW-B commanding to make use of 50% increase in telemetry. Not fully successful until 2013/10/10. For parameters, see 2013/10/10 entry.

2013/10/05: M35-A, M35-B “ ”.

2013/10/06: M75-A, M75-B “ ”.

2013/10/07: HIGH-A, HIGH-B “ ”.

2013/10/10: All 8 MagEIS units now in the high-telemetry mode. Main rate parameters: 1 spin cadence; 50 sectors/spin (LOW/MED), 32 sectors/spin (HIGH elec & prot). Histogram parameters: 6 spin cadence; 25 sectors/spin (LOW/MED); 16 sectors/spin (HIGH elec); 32 sectors/spin (HIGH prot).

2013/10/11: HIGH-A hard reboot at ~2145UTC. Unit left in maintenance mode (no science data) until 2013/10/14.

2013/10/14: HIGH-A rebooted back into science mode.

2013/10/16: HIGH-A electron threshold changes. Any step function increases in HIGH unit electron flux around this time are likely non-physical.

2013/10/25: LOW-B high-rate mode L>3 on every orbit. Parameters: 360 sectors, 7 channels (pixels 2-8).

2013/11/09: Missing data, L1 RBSP-B. Must be a SOC issue. My code processed all L0 data fine locally.

2013/12/20: LOW-A and LOW-B high-rate mode L>3 on every orbit. Parameters: 500 sectors, 7 channels (pixels 2-8). LOW-B ends on 2014/02/20. LOW-A remains indefinitely.

2014/01/17: HIGH-A, HIGH-B increased electron sectoring (50 for main rates, 25 for histogram) to match LOW/MED.

2014/02/21: LOW-B high-rate mode L>3 on every orbit ends (began 2013/12/20).

2014/04/03: LOW-A high-rate mode changed from L>3 on every orbit to L>4.

2014/05/07: Missing data, L1 RBSP-A. Must be a SOC issue. My code processed all L0 data fine locally.

2014/05/23: HIGH-A, HIGH-B coincidence toggle (on/off) testing. Coincidence left off until 2014/05/28. Any step function increases in HIGH unit electron flux around this time are likely non-physical.

2014/05/29:

- HIGH-A, HIGH-B LUT upload. Engineering histogram LUT that is PHA channels 0-63 only (uploaded into slot). Used to take high resolution histogram data from the front detectors. Note that this does not affect the main electron channels.
- HIGH-B bad heater set point. Temperature oscillation in pixel 3 until 2014/09/18. Data is fill.
- HIGH-A bad threshold on pixel 0. Caused noise in other pixels until 2014/05/31.

2014/06/01: HIGH-A, HIGH-B coincidence toggle (on/off) testing. Front detector histograms. Any step function increases in HIGH unit electron flux around this time are likely non-physical.

2014/06/04: HIGH-A coincidence toggle (on/off) testing. Front detector histograms. Any step function increases in HIGH unit electron flux around this time are likely non-physical.

2014/06/05: HIGH-A, HIGH-B coincidence toggle (on/off) testing. Front detector histograms. Any step function increases in HIGH unit electron flux around this time are likely non-physical.

2014/06/27: LOW, M35, M75, HIGH on both RBSP-A and RBSP-B commanded into very-high-telemetry mode. Main rate parameters: 1 spin cadence; 64 sectors/spin (LOW/MED); 32 sectors/spin (HIGH elec & prot). Histogram parameters: 2 spin cadence; 32 sectors/spin (LOW/MED/HIGH elec & prot).

2014/08/06: HIGH-A bad heater set point. Temperature oscillation in pixel 1 until 2014/11/07. Data is fill.

2014/10/15: HIGH-A and HIGH-B proton thresholds raised to remove low channel noise from data stream. Results in loss of science data from proton channels 0-5 (~60-150 keV) from this point forward.

2015/02/02: LOW-B high-rate mode for close approach event. Parameters: 500 sectors, 7 channels (pixels 2-8).

2015/04/08: LOW-B high-rate mode for L>4 on every orbit for close approach event. Parameters: 500 sectors, 7 channels (pixels 2-8). Ends on 2015/04/10.

2015/05/20: Missing data, L1 RBSP-B. Must be a SOC issue. My code processed all L0 data fine

locally.
2015/07/01: Missing data, L1 RBSP-A. Must be a SOC issue. My code processed all L0 data fine locally.
2015/07/15: M35-B anomaly. Likely SEU. No science data until 2015/07/30. Unit is nominal after 2015/07/30.
2015/07/24: LOW, M35, M75 (RBSP-A and RBSP-B) derived channel time cadence changed from 2 spins to 1 spin.
2015/09/15: MOC (APL) issue results in complete, unrecoverable data loss for ~19 hours.
2015/09/16: M35-A and M35-B new high-rate mode LUTs uploaded.
2015/09/17:

- M35-A and M35-B high-rate mode over entire orbit (continuous). Parameters: 180 sectors, 7 channels (pixels 2-8).
- MagEIS-A&B bad magnetic ephemeris files used to generate perigee pulser sweep commands. Resulted in perigee pulser sweeps being executed not at perigee (bad). Issues was corrected on 2015/09/30. Pulser sweeps executed in inner zone during this time, progressively moving to higher L until 2015/09/30.

2015/09/30: MagEIS-A&B "perigee" pulser sweep commands terminated. Were incorrectly being executed in the inner zone since 2015/09/17. Were not restarted again until 2016/02/25.
2015/10/06: M35-A and M35-B high-rate mode over entire orbit (continuous) sectoring increased from 180 to 2048 (still 7 channels (pixels 2-8)).
2015/10/15: M35-A and M35-B high-rate mode over entire orbit (continuous) sectoring decreased from 2048 to 1960 (still 7 channels (pixels 2-8)).
2015/12/10: M35-A and M35-B high-rate mode over entire orbit (continuous): Campaign ends.
2016/01/11: MOC (APL) issue results in complete, unrecoverable data loss on RBSP-A (~1 day).
2016/01/12: MOC (APL) issue results in complete, unrecoverable data loss on RBSP-A (~1 day).
2016/01/31: SOC commanding issue results in LOW-A stuck in high-rate (sample) mode from 2/1-2/10.
2016/02/25: MagEIS-A&B perigee pulser sweep commands restarted (not conducted since 2015/09/30). However, these pulser sweeps were done at the highest pulser setting of 10 kHz (all previous pulser sweeps were conducted at 1 kHz). The 10 kHz pulser campaign lasted until 2016/03/12.
2016/03/11: HIGH-B heater set point lowered from 28 to 26 (which raised the yoke temperature). Done because downward trend in yoke temp was accelerating.
2016/03/12: MagEIS-A&B perigee pulser sweep commands returned to 1 kHz (10 kHz pulser was run from 2/25 to 3/12).
2016/03/18: HIGH-B heater set point lowered from 26 to 24 (which raised the yoke temperature). Done because downward trend in yoke temp was accelerating. This was the final adjustment for now – yoke temp back into a good range.
2016/05/15: LOW-A high-rate mode changed from 500 sectors to 1000 sectors. All other parameters the same as before (L>4; 7 channels (pixels 2-8).)
2016/06/22: MOC (APL) issue results in complete, unrecoverable data loss on RBSP-A (~1 day).
2016/06/23: MOC (APL) issue results in complete, unrecoverable data loss on RBSP-A (~1 day).
2016/11/26: HIGH-A temperature oscillation in pixel 1 until 2016/12/06, likely due to seasonal change in thermal environment. Data from this pixel is set to fill for this time interval.
2016/12/06: HIGH-A heater set point lowered from 28 to 26 (which raised the yoke temperature). Done to combat thermal oscillation in pixel 1 first noted on 2016/11/26.
2016/12/26: Missing data, L1 RBSP-A. Must be a SOC issue. My code processed all L0 data fine locally.
2017/07/28: LOW-A perigee pulser sweeps stop until 2017/11/01.

2017/07/28: LOW-A: Normal operation of L>4 high-rate mode stops. No high-rate mode data through 2017/07/29.

2017/07/30: LOW-A: high-rate mode L>4 INBOUND/L>3 OUTBOUND (through 2017/08/04); M35-A: high-rate mode over entire orbit (through 2017/08/01); M75-A: high-rate mode over entire orbit with old high-rate mode LUT (5 channels).

2017/07/31: M75-A: Bad main rate/histogram nsectors/nspins parameters from 00:00-06:00; M75-A: Sample mode over entire orbit through 2017/08/01 (New high-rate mode LUT - 7 channels); M75-B: Bad main rate/histogram nsectors/nspins parameters from 00:00-01:30; M75-A and M75-B new high-rate mode LUTs uploaded at 00:00:00 UTC.

2017/08/02: M35-A: Sample mode L>3 through 2017/08/31; M75-A: Sample mode L>3 through 2017/08/31 (New high-rate mode LUT - 7 channels).

2017/08/05: LOW-A: Sample mode L>3 INBOUND and OUTBOUND through 2017/08/31; 07:00-07:15: LOW-B: Sample mode, but nsectors = 500 (1000 intended); M35-B: Sample mode, but nsectors = 180 (1000 intended); M75-B: Sample mode, but nsectors = 180 (1000 intended)

2017/08/13 23:00-23:20: LOW-B: Sample mode, but nsectors = 500 (1000 intended); M35-B: Sample mode, but nsectors = 180 (1000 intended); M75-B: Sample mode, but nsectors = 180 (1000 intended)

2017/08/14 08:20-08:42: LOW-B: Sample mode, but nsectors = 500 (1000 intended); M35-B: Sample mode, but nsectors = 180 (1000 intended); M75-B: Sample mode, but nsectors = 180 (1000 intended)

2017/08/21 LOW-A commanding anomaly. Data lost until 9/26

2017/08/22 15:10-15:40: LOW-B: Sample mode, but nsectors = 500 (1000 intended); M35-B: Sample mode, but nsectors = 180 (1000 intended); M75-B: Sample mode, but nsectors = 180 (1000 intended)

2017/09/01: LOW-A: Sample mode L>4 INBOUND/L>3 OUTBOUND through 2017/09/12

2017/09/07: All 8 units all standing request commanding stops, including perigee pulser sweeps, which do not restart again until 2017/11/01.

2017/09/13: LOW-A: Sample mode entire orbit through 2017/09/19

2017/09/26: LOW-A: science mode data restored (lost since 8/21 due to anomaly).

2017/10/01: LOW-A command script to put into science mode ran at 00 UTC but had no effect since unit was already in science mode.

2017/10/04: HIGH-A pixel 01 temperature oscillation starts. Ends on 10/12.

2017/10/05: HIGH-A pixel 01 buried detector threshold (DAC0) raise from 2250->2450 to zero out the counts, as the noise on this detector has gotten too bad (noise began trending up in ~August 2016). Note that this and the subsequent raises did not fully zero things out until 11/2.

2017/10/11: HIGH-A pixel 01 change heater set point 26->24 to raise yoke temperature to combat thermal oscillation that began on 10/4.

2017/10/12: HIGH-A pixel 01 temperature oscillation ends. Began on 10/4. Data is set to fill during this time interval..

2017/10/12: HIGH-A pixel 01 buried detector threshold (DAC0) raise from 2450->2600 to zero out the counts.

2017/10/18: HIGH-A pixel 01 buried detector threshold (DAC0) raise from 2600->2700 to zero out the counts.

2017/10/24: HIGH-A pixel 01 buried detector threshold (DAC0) raise from 2700->2800 to zero out the counts.

2017/10/24: LOW-A high rate (sample mode) data collections begin again at L>4 (they were stopped around the time of the August 2017 anomaly). 1000 sectors, 7 channels.

2017/11/01: All 8 units perigee pulser sweeps restarted but now occur only on the first perigee of each month.

2017/11/02: HIGH-A pixel 01 buried detector threshold (DAC0) raise from 2800->4000 to zero out the counts. Wiped out all of the counts except for sporadic 1 counts that occur in random sectors at random times. It is unclear why we get them but it is nothing to worry about.

2017/11/14: MOC (APL) issue results in complete, unrecoverable data loss on RBSP-A (~1 day).
2017/11/15: MOC (APL) issue results in complete, unrecoverable data loss on RBSP-A (~1 day).
2018/01/03: HIGH-A pixel 01 change heater set point 24->22 to raise yoke temperature to combat thermal oscillation that began on 12/27. Data is set to fill during this time interval.
2018/02/22: LOW-A high rate (sample mode) commanding issues. No high rate data from 2/22 – 2/26.
2018/04/11: HIGH-A pixel 01 change heater set point 22->20 to raise yoke temperature to combat thermal oscillation that began on 3/24. Data is set to fill during this time interval.
2018/04/27: HIGH-A pixel 01 change heater set point 20->18 to raise yoke temperature to combat thermal oscillation that began on 3/24. Data is set to fill during this time interval.
2018/06/22: MOC (APL) issue results in complete, unrecoverable data loss for ~4 hours on RBSP-B.
2018/08/18-20: SOC (UNH) scheduler failure results in LOW-A being in highrate mode for 3 consecutive days (should go into highrate mode only above L=4).
2018/10/22: LOW-A high rate (sample mode) commanding issues (MOC disk failure). Stuck in high rate mode for most of the day.
2018/10/24: LOW-A high rate (sample mode) commanding issues (MOC disk failure). No high rate data for most of this day.
2019/01/26: LOW-A high rate (sample mode) commanding issues (SOC failed to generate predictive magnetic ephemeris files). Stuck in high rate mode until 2/1.
2019/02/01: LOW-A high rate (sample mode) commanding issues from 1/26 fixed. Regular high rate mode commanding resumes (highrate mode only above L=4).
2019/07/16: At ~16:58:00 UTC the science instruments on RBSP-B shut down as part of the end-of-mission passivation activities.
2019/10/14: At ~14:27:00 UTC the science instruments on RBSP-A shut down as part of the end-of-mission passivation activities.
